# Ketenimines as Aza-Dienophiles

**DOI:** 10.1021/jacs.4c17174

**Published:** 2025-02-07

**Authors:** Christopher J. DeAngelis, Geeta Goyal, Marshall J. Liss, Jessica E. Budwitz, Mary Stuart Herlihy, Audrey V. Conner, Steven E. Wheeler, Pengchen Ma, Miranda Li, K. N. Houk, Christopher G. Newton

**Affiliations:** † Department of Chemistry, University of Georgia, Athens, Georgia 30602, United States; ‡ Department of Chemistry and Biochemistry, University of California, Los Angeles, California 90095, United States

## Abstract

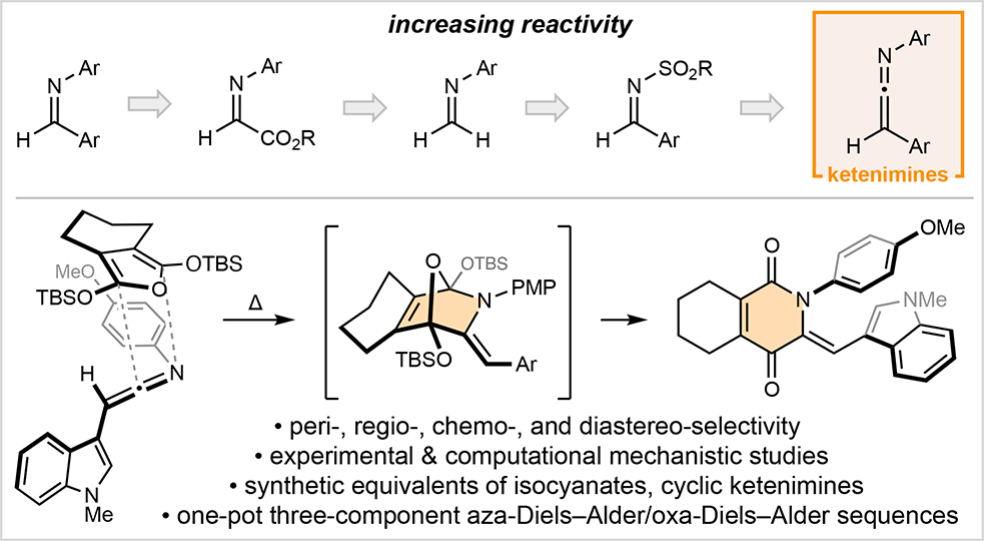

*N*-Aryl ketenimines have been established as highly reactive aza-dienophiles. Intermolecular cycloadditions are achieved upon heating in the presence of 2,5-bis(silyloxy)furans and proceed with high levels of peri-, regio-, chemo- and diastereo-selectivity. Spontaneous C–O cleavage yields oxygenated pyridone derivatives in a highly convergent and redox-neutral manner. Combined experimental and computational studies demonstrate *N*-aryl ketenimines to be significantly more reactive than imino dienophiles, as a consequence of less distorted transition states. Derivatization studies include the development of isocyanate and cyclic ketenimine equivalents as aza-dienophiles, while extension to a one-pot aza-Diels–Alder/oxa-Diels–Alder sequence provides a three-component approach to complex fused pyridone/pyran systems.

## Introduction

**Figure 1:**
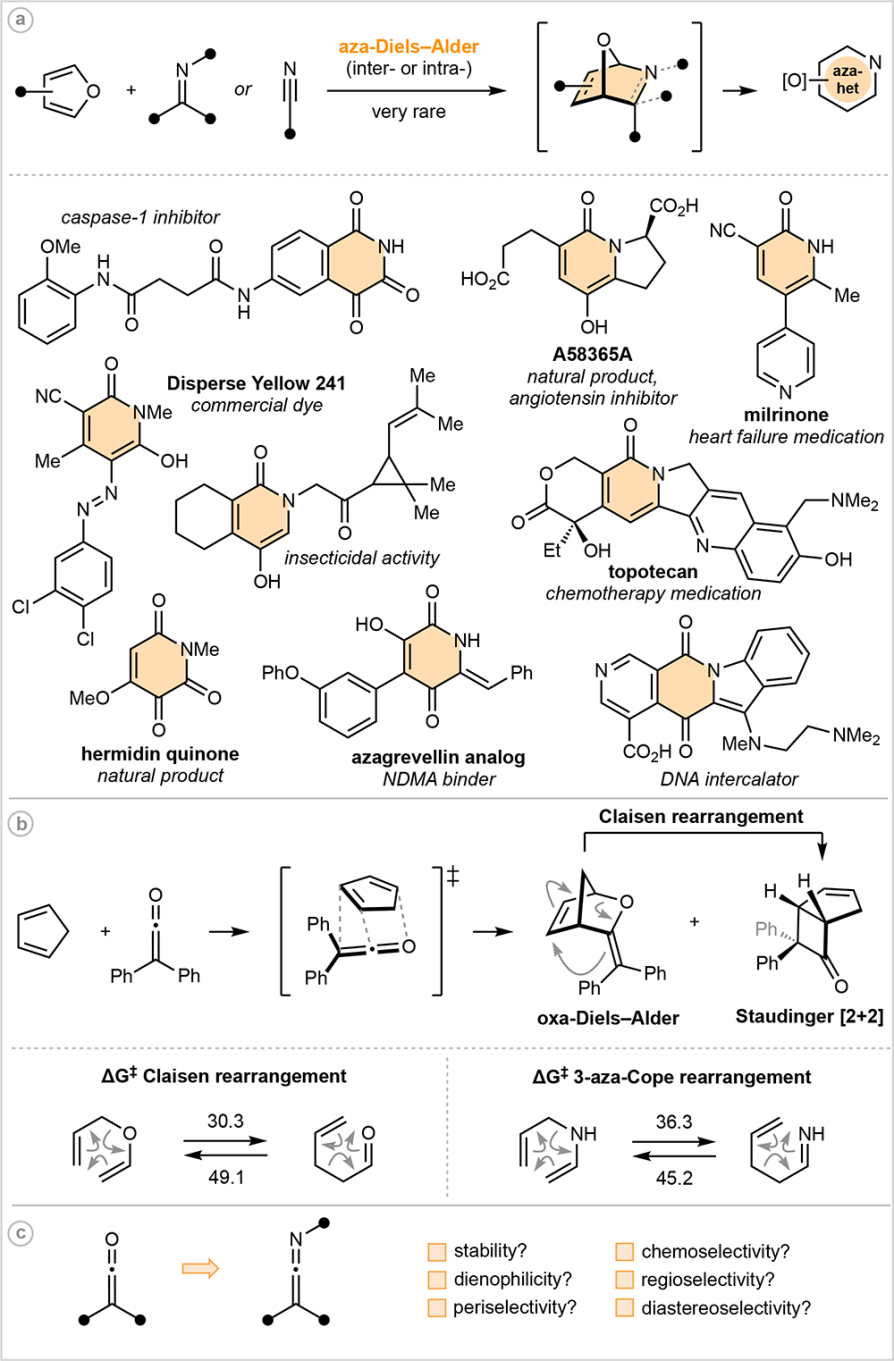
Aza-Diels–Alder reactions of ketenimines for the synthesis of oxygenated aza-heterocycles. Computations: CCSD(T)/cc-pVTZ//M06-2*X*/6-311+G(d,p) level of theory, Δ*G*^⧧^ values are given in kcal·mol^–1^.

Oxygenated pyridine derivatives feature within a broad array of natural products,^1^ pharmaceuticals,^2^ drug candidates,^3^ agrochemicals,^4^ and reagents.^5^ Aza-Diels–Alder reactions of furans, if partnered with controlled cleavage of the resulting bridging ether, would provide a convergent and redox-neutral approach to such motifs (Figure 1a). However, cycloadditions of this nature are remarkably rare (full details in the Supporting Information).^6^ It is perhaps unsurprising this reactivity profile remains problematic given Diels–Alder reactions of furans are typically restricted to highly reactive dienophiles.^6c,6f,6g^ While Brønsted and Lewis acids can be employed to enhance the reactivity of aza-dienophiles,^6b,6e,6h−6j^ the sensitivity of furans and their aza-cycloadducts,^7^ coupled with competitive Mannich-type reactivity,^8^ precludes their implementation in this context. Motivated to address this long-standing challenge in the pericyclic space, we set out to establish a new and general class of highly reactive aza-dienophiles.

In 1907, Staudinger disclosed a thermally promoted [2 + 2] addition of cyclopentadiene across the C=C bond of diphenylketene to generate a fused cyclobutanone.^9^ While several mechanisms were later proposed,^10^ it was not until 2006 that Singleton demonstrated cyclopentadiene reacts with diphenylketene through a bifurcating ambimodal transition state to give a mixture of both the [4 + 2] and [2 + 2] adducts.^11^ While the oxa-Diels–Alder adduct is favored, rapid Claisen rearrangement exclusively yields the Staudinger [2 + 2] adduct (Figure 1b).^12^ Given that 3-aza-Cope rearrangements tend to be significantly less favorable than analogous Claisen rearrangements,^13,14^ we anticipated ketenimine aza-Diels–Alder cycloadducts to be recalcitrant toward [3,3] sigmatropic rearrangement. Furthermore, considering diphenylketene reacts with cyclopentadiene at temperatures as low as −20 °C,^10a,10b^ it stood to reason that ketenimines may also be highly reactive heterodienophiles (Figure 1c).

## Results and Discussion

Instances of ketenimines participating as aza-dienophiles in [4 + 2] processes are scant. To the best of our knowledge, only two reports with carbo-dienes have been disclosed: a single mechanistically speculative reaction reported within a control study from Sarpong^15^ and a brief communication from Lectka describing ketenimine Diels–Alder reactivity with a large excess of the highly reactive cyclopentadiene, followed by cleavage of the newly formed *N*-heterocycle upon workup.^16^ This dearth of interest may be attributable to several reports describing ketenimines as difficult to prepare, purify, or handle.^16,17^ Contrary to these reports, in the present study, we have established that many ketenimines exhibit an excellent stability profile even when held neat or exposed to aqueous media. As far as we can discern, decomposition primarily occurs via acid-catalyzed hydrolysis to the amide; thus, provided exposure to acid is limited, long-term storage is well tolerated (see the Supporting Information for a detailed stability assessment).^18^

**Figure 2:**
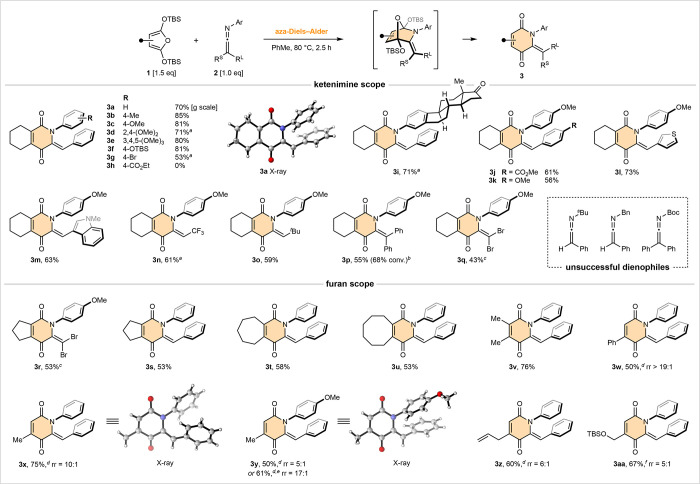
Scope of the ketenimine aza-Diels–Alder reactions. ^*a*^NMR yield. ^*b*^Reaction was run in the microwave at 200 °C for 3 h; conversion determined by ^1^H NMR. ^*c*^Reaction run for 17 h. ^*d*^Yield of the major regioisomer. ^*e*^Reaction run in CCl_4_. ^*f*^Yield of both regioisomers.

**Figure 3:**
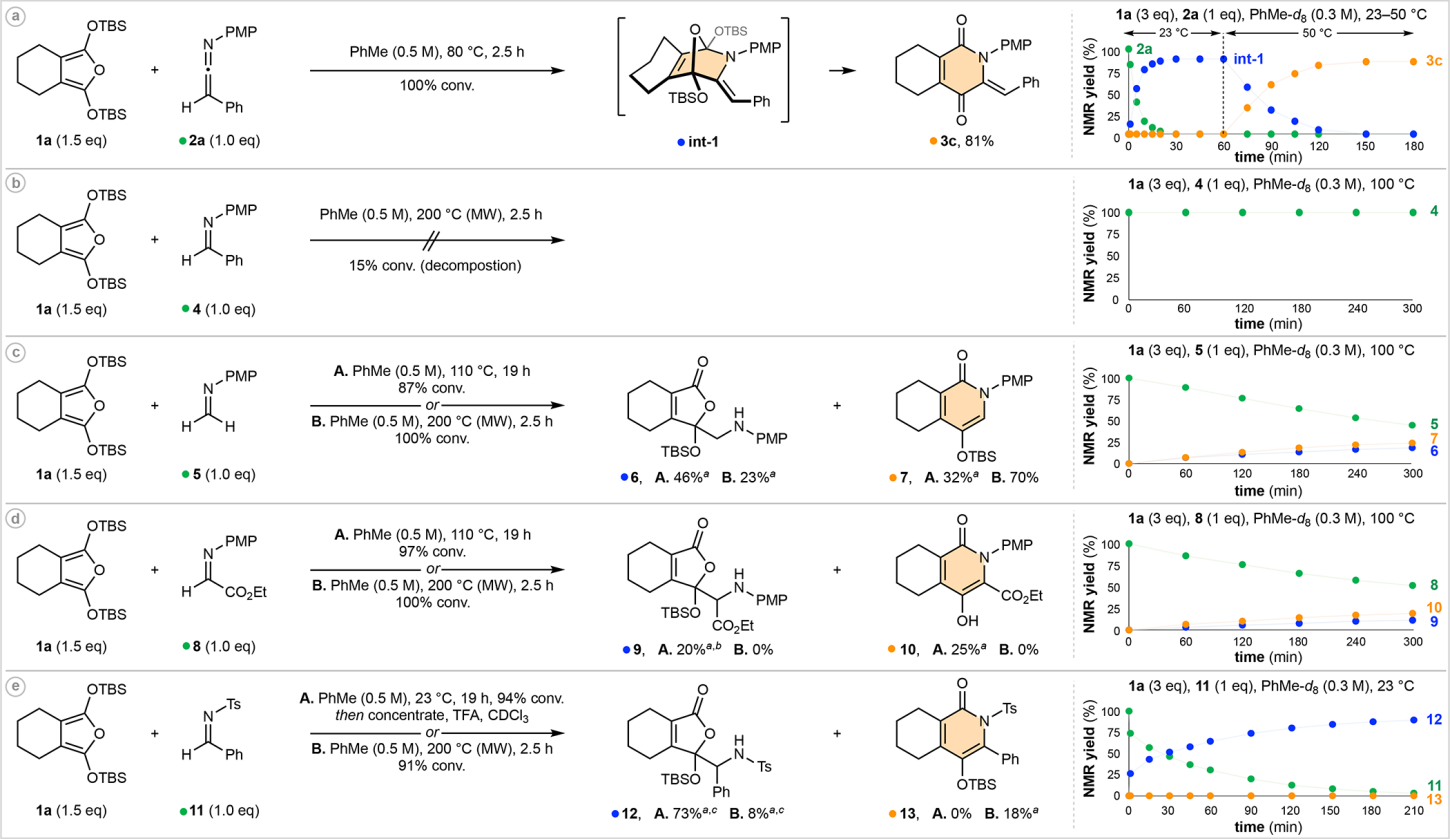
Imines vs ketenimines as aza-dienophiles. ^*a*^NMR yield. ^*b*^dr = 1.3:1. ^*c*^dr > 19:1.

Cycloaddition studies were initiated with our recently reported family of 2,5-bis(*tert*-butyldimethylsilyloxy)furans as vicinal bisketene equivalents (Figure 2).^19^ A brief optimization study revealed simply heating a solution of both cycloaddition partners **1** and **2** in anhydrous toluene at 80 °C for 2.5 h directly provided the desired pyridine-2,5-diones **3**. Overall, the reaction is highly (i) periselective (no evidence for [2 + 2] reactivity), (ii) chemoselective (no evidence for Povarov or Mukaiyama–Mannich reactivity), and (iii) diastereoselective (no evidence for the generation of (*E*)-**3a**, although care must be taken during purification to avoid isomerization). On gram scale purification was achieved via a simple trituration procedure and proceeded in 70% isolated yield. While several diene/dienophile combinations underwent Diels–Alder reaction rapidly at ambient temperature (e.g., see Figure 3a), reactions were primarily conducted at 80 °C to maximize operational simplicity and generality.

Ketenimine scope studies revealed that precise electronic matching of the diene and dienophile is not critical. For example, ketenimines bearing electron-rich aromatics on nitrogen (**3b**–**3f**, **3i**) performed exceptionally well, with silylated phenol **3f** also serving to highlight the chemoselectivity of the desilylative ring-opening process. Electronically deactivated ketenimines were also well-behaved in the Diels–Alder reaction; however, reduced stability of the direct cycloadduct and/or ring-opened aza-heterocycle resulted in lower yields (**3g**). This came to a head in the case of *para*-CO_2_Et functionality (**3h**), for which the cycloadduct was generated in 91% NMR yield, but all attempts to isolate the ring-opened product were unsuccessful.^20^ Despite this limitation, we anticipate that electron-poor ketenimines will find success in alternate Diels–Alder contexts. With respect to ketenimine *C*-substitution, functional group tolerance is excellent (**3j**–**3q**). Highlights include incorporation of heteroaromatics (**3l**, **3m**), (fluoro)alkyl substituents (**3n**, **3o**), and sterically demanding derivatives (**3o**–**3q**). Unfortunately, attempts to move away from *N*-aryl ketenimines were unsuccessful (dashed boxed, details in the Supporting Information).

Furan scope was primarily explored using 1,3-diphenylketenimine as the dienophile. With respect to symmetric furans, aza-heterocycles fused to 5-, 7-, and 8-membered rings were all smoothly accessed (**3r**–**3u**), as too was dimethyl derivative **3v**. Unsymmetric furans required an additional level of control during the cycloaddition event. Pleasingly, regioselectivity tended to be excellent (**3w**), even when a simple methyl group served as a director (**3x**). Although a reduction in regioselectivity was observed with more electron-rich ketenimines (e.g., compare **3x** with **3y**), a simple solvent change improved the regioisomeric ratio (rr) of **3y** from 5:1 to 17:1. More functionalized unsymmetric furans were also successfully screened under our standard conditions (**3z** and **3aa**).

To better contextualize the reactivity of *N*-aryl ketenimines, a range of more traditional aza-dienophiles were screened against the same class of furan (Figure 3). Using our standard reaction stoichiometry, each aza-dienophile was mixed with cyclohexyl-furyl derivative **1a** across various temperature and time ranges (lefthand column). While these experiments are informative about overall reaction efficacy, the results are not a measure of dienophilicity given the ether ring-opening process may be rate-determining. Thus, each aza-dienophile was also separately mixed with a 3-fold excess of furan in deuterated toluene, and reaction progress was closely monitored by ^1^H NMR analysis (righthand column). *para*-Methoxyphenyl ketenimine **2a** was selected as our standard of comparison (Figure 3a). This aza-Diels–Alder reaction was both fast and high-yielding at ambient temperature, reaching completion in less than 30 min. Ring opening of the direct cycloadduct required gentle warming, and at 50 °C, this process took approximately 2 h. In contrast, imine **4** (which can be regarded as a truncated derivative of ketenimine **2a**) was unreactive, even at temperatures as high as 200 °C (Figure 3b). While the less sterically hindered formaldimine **5** was productive, mixtures of furanone **6** and aza-Diels–Alder adduct **7** were observed, with high temperatures necessary for good yields, albeit those of less functionalized products (Figure 3c). Attempts to observe the direct aza-cycloadduct were unsuccessful. With respect to the mechanistic origins of **6**, it could arise via an aza-Diels–Alder/C–N bond cleavage or a Mukaiyama–Mannich reaction. It is also worth highlighting that, in contrast to ketenimine **2a**, formaldimine **5** is prone to polymerization upon concentration.

Incorporation of electron-withdrawing substituents had a deleterious impact on chemoselectivity (dienophiles **8** and **11**, Figure 3d and 3e). In the case of ester **8**, mixtures of furanone **9** and nonsilylated pyridone **10** were observed, suggestive of a stepwise [4 + 2] process supported by the conversion of **9** into **10** with NaH (see the Supporting Information). *N*-Tosyl imine **11** proved more challenging to assess: our results suggest a highly unstable aza-cycloadduct forms at room temperature (ca. ten times slower than the reaction of ketenimine **2a**); however, high temperatures and nonacidic conditions are necessary to promote the desired C–O bond cleavage, highlighting the importance of an available *N*-lone pair for this process. In terms of monitoring the rate of reaction, we resorted to measuring the consumption of **11** at ambient temperature, followed by acidification of NMR aliquots to measure the yield of **12**. In summary, Figure 3 serves to highlight the unique reactivity of *N*-aryl ketenimines, in terms of both their dienophilicty and the mild and chemoselective nature of their subsequent ether cleavage.

**Figure 4:**
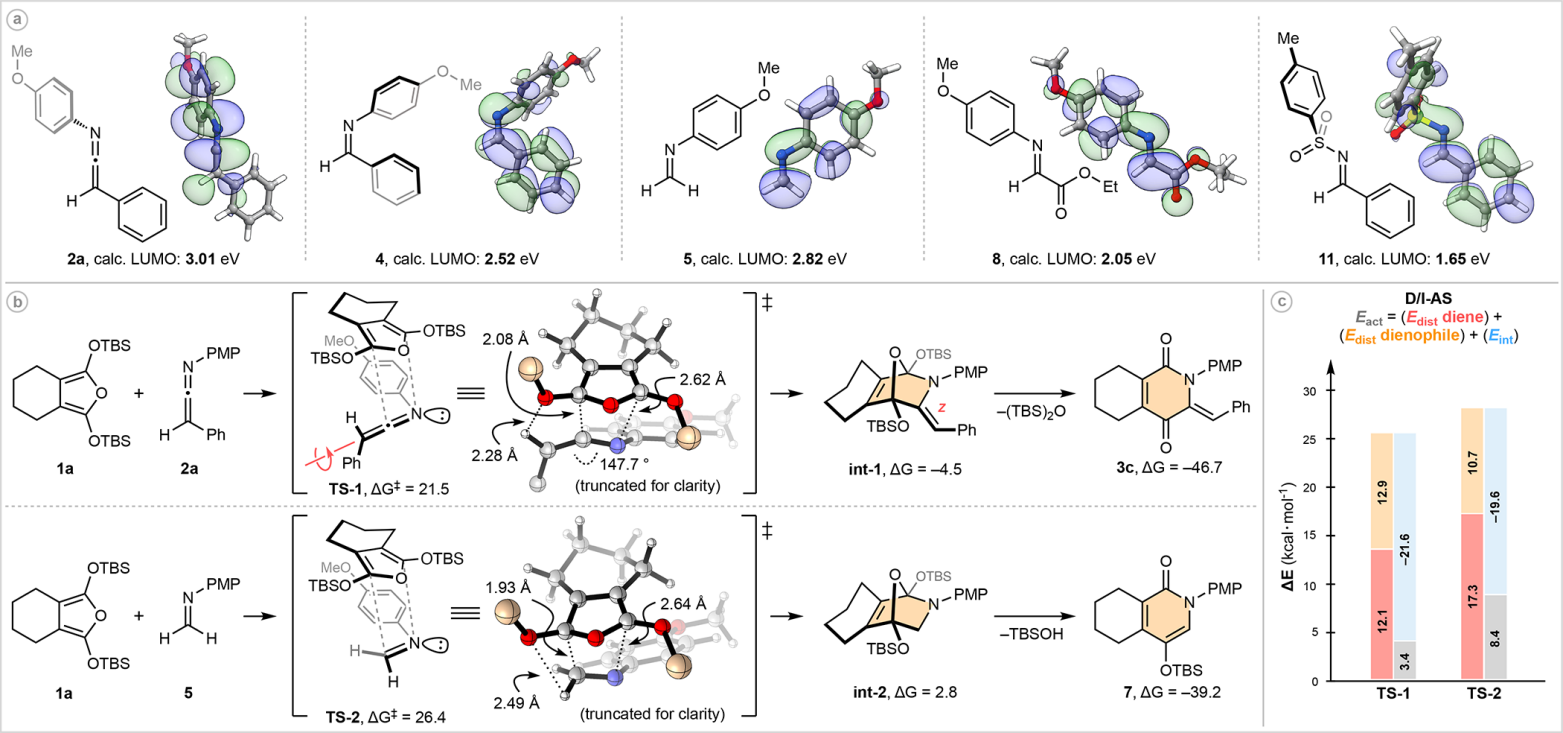
Computations: ωb97X-D/def2TZVP(CPCM,toluene)//ωb97X-D/6-31+G(d), *t* = 353.15 K. Δ*G*/Δ*G*^⧧^ values are given in kcal·mol^–1^.

To understand why *N*-aryl ketenimines are superior aza-dienophiles we began by calculating the LUMO energies for each dienophile employed within our comparative studies (Figure 4a).^21^ Despite the incorporation of sp-hybridization within ketenimine **2a**, it exhibits the highest LUMO energy at 3.01 eV (almost 1.4 eV higher than the next most reactive partner, tosyl imine **11**). This indicates that frontier molecular orbital (FMO) interactions are not the dominant controlling factor in the Diels–Alder reactivity of *N*-aryl ketenimines, which helps account for the largely inconsequential electronic nature of their *N*-aryl substituents.

We next modeled the aza-Diels–Alder reaction of ketenimine **2a**, alongside the second most chemoselective dienophile, formaldimine **5** (Figure 4b). In alignment with our previous studies concerning the Diels–Alder reactivity of simple imino dienophiles,^22^ DFT calculations indicate both reactions proceed via an asynchronous but concerted cycloaddition that preferentially occurs via the *exo* mode with respect to the *N*-lone pair (see the Supporting Information for *endo*-mode transition-state coordinates). This orientation minimizes electrostatic repulsion between the nitrogen lone pair and the furan π-electrons and may be accompanied by stabilizing C–H−π interactions between the cyclohexyl and *para*-methoxyphenyl substituents. With respect to the diastereoselectivity of our ketenimine cycloadditions, the diene preferentially approaches from the opposite face of the largest substituent (Ph). As the new C—C bond forms, the requisite bond rotation (shown by red arrow) translates into a *Z*-relationship in **int-1**. For both **TS-1** and **TS-2**, the forming C—C bond is shorter than the forming C—N bond, although the difference between the two transition states is more pronounced with respect to C—C formation (2.08 Å vs 1.93 Å). This can be rationalized through pseudo-A^1,3^ strain between the ketenimine C—H bond and the proximal OTBS group (H—O distance = 2.28 Å vs 2.49 Å). For **TS-1**, significant bending about the ketenimine C=C=N bond is observed, distorting away from its ground state (175.5°) to 147.7° as it rehybridizes. Despite these effects, **TS-1** is calculated to be 4.9 kcal·mol^–1^ lower in energy than **TS-2**.

The cycloaddition of formaldimine **5** is endergonic by 2.8 kcal·mol^–1^, rationalizing our unsuccessful attempts to experimentally observe **int-2**. Furan hetero-Diels–Alder reactions are often highly reversible;^6g^ however, the retro-aza-Diels–Alder pathway appears to be inhibited when ketenimines are employed as dienophiles (Δ*G* for **int-1** = −4.5 kcal·mol^–1^). The stabilization provided by generation of the enamine functionality is likely a key contributor. Ether-cleavage is highly energetically favorable in both reactions.

While these results help rationalize many of our experimental observations, the enhanced dienophilicty of **2a** has yet to be addressed. To elucidate the origin of this effect we next carried out Distortion/Interaction-Activation Strain (D/I-AS) analysis^23^ of both **TS-1** and **TS-2** (Figure 4c). Overall, *E*_act_ for **TS-1** is 5 kcal·mol^–1^ lower in energy than that for **TS-2**, which parallels the difference in their Δ*G*^⧧^ values and is in good agreement with our experimental results. The individual D/I-AS analysis components indicate significantly less distortion of the furan upon reaction with ketenimine **2a** relative to formaldimine **5** (12.1 vs 17.3 kcal·mol^–1^), aligning well with the characteristics of an early transition state in the case of **TS-1**. The interaction energies of both processes are also informative. The value for **TS-1** is 2.0 kcal·mol^–1^ greater in magnitude than that for **TS-2**, suggesting that ketenimine **2a** is slightly more electrophilic than **5**, and/or the former is benefiting from a more favorable CH–O interaction.

**Figure 5:**
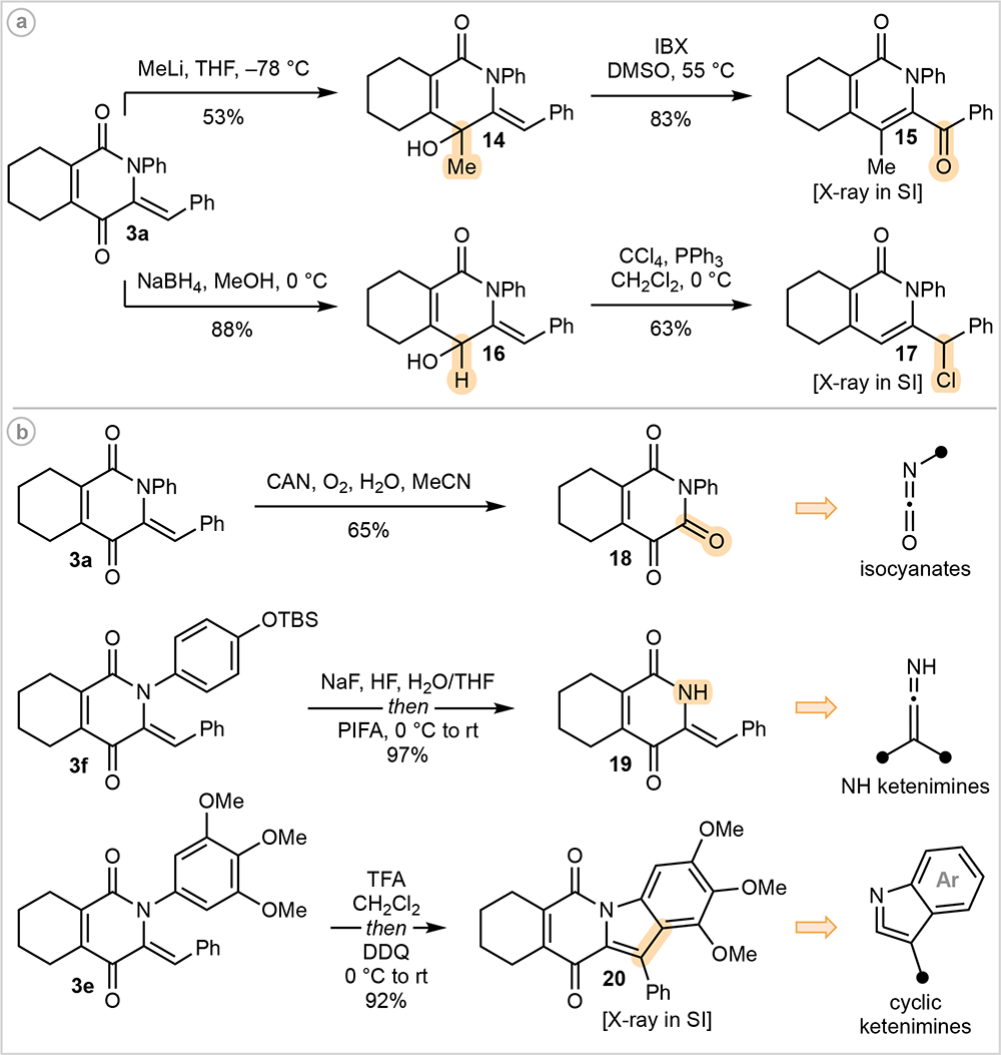
Derivatization studies.

While our furyl/ketenimine cycloaddition provides efficient access to unusual *N*-heterocyclic chemical space, an important goal in its own right,^24^ conversion into frameworks of already established importance was readily achieved by a general two-step protocol (Figure 5a). Nucleophilic addition into the ketone fragment of **3a** generated either **14** or **16**, which was followed by an allylic transposition event to generate the ubiquitous 2-pyridone motif,^25^ demonstrated under both oxidative (**15**)^26^ and redox-neutral (**17**) conditions.

Chemoselective modification of the ketenimine-derived fragment in **3** creates opportunities for the development of synthetic equivalents to otherwise inaccessible or poorly behaved aza-dienophiles. With this objective in mind, while also looking to intercept biologically relevant frameworks, we have developed three such protocols (Figure 5b). Oxidative cleavage of the semicyclic olefin in **3a** was achieved through treatment with CAN in the presence of water and oxygen to generate pyridine-2,3,6-trione **18** (cf. hermidin quinone, Figure 1a). Presumably this reaction proceeds via an oxa-Michael/retro-aldol/enolate oxidation sequence^27^ and formally represents the application of an isocyanate as aza-dienophile, a reactivity profile that has only been realized with sulfonyl isocyanates in low yield.^28^ While NH ketenimines have been implicated as intermediates in flash vacuum pyrolysis experiments,^29^ to the best of our knowledge they have not been isolated. To this end, a high-yielding one-pot desilylation/oxidative phenol cleavage^30^ of **3f** was employed to reveal deprotected derivative **19** (cf. azagrevellin analog, Figure 1a), facilitating the introduction of *N*-functionality that may not be tolerated during cycloaddition. Treatment of **3e** with TFA, followed by addition of DDQ, provided indole-fused derivative **20** in 92% yield (cf. DNA intercalator, Figure 1a). Formally, this transformation represents the employment of a cyclic ketenimine as aza-dienophile, an unrealized class of strained dienophile.

**Figure 6:**
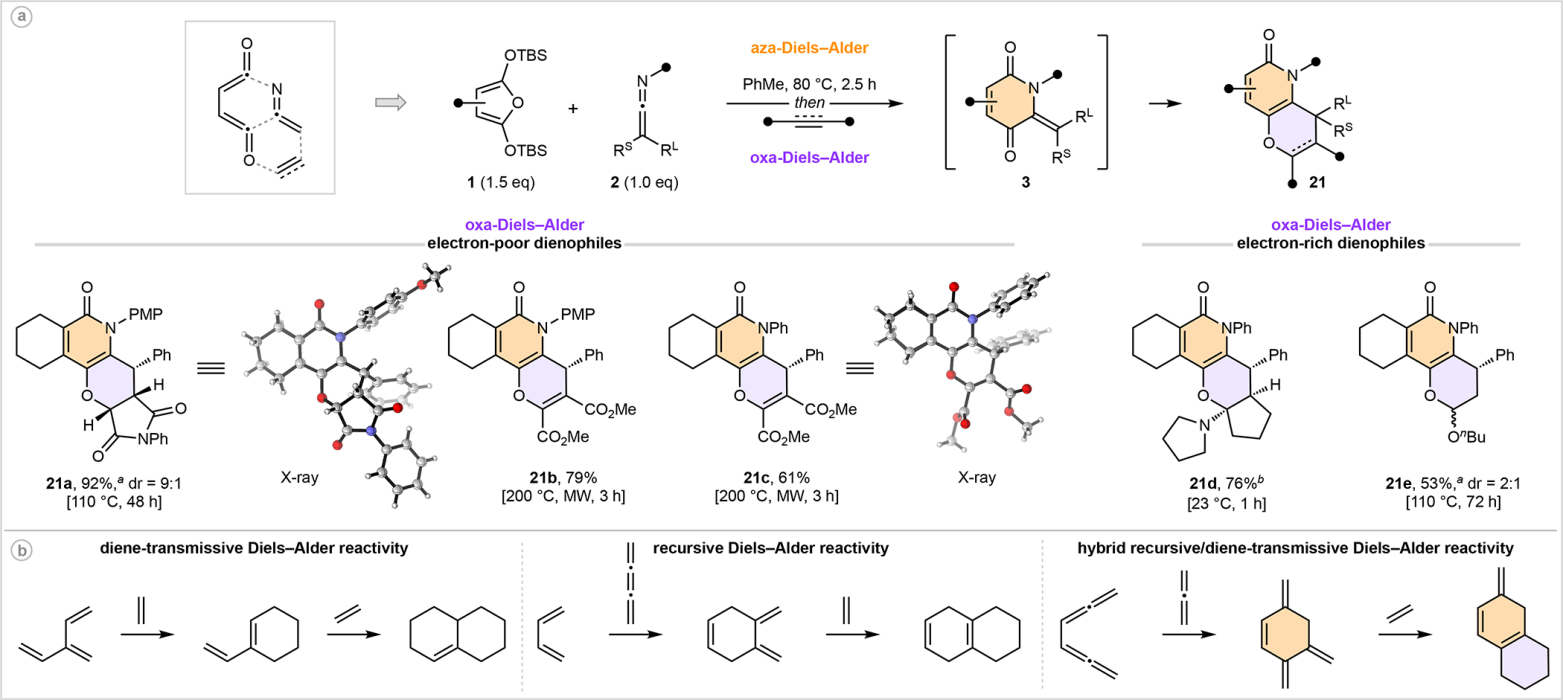
Ketenimines as linchpin reagents in one-pot, three-component aza-Diels–Alder/oxa-Diels–Alder sequences. ^*a*^Yield of both diastereoisomers. ^*b*^Isolated as an equilibrating 6:1 mixture of its ring-closed (as drawn) and ring-opened hemiaminal ether; see the Supporting Information for more details.

A significant advantage of the development of reagent free reactions is conduciveness to telescoped reactivity. For our final set of derivatization experiments, we investigated the potential of ketenimines as linchpin reagents in one-pot aza-Diels–Alder/oxa-Diels–Alder sequences for the efficient synthesis of highly functionalized pyran-fused pyridones (Figure 6a). Specifically, we envisioned trapping the oxadienes generated in the aza-Diels–Alder/ring-opening sequence with a second dienophile, ultimately resulting in the union of three discrete molecules via the formation of two new rings. Gratifyingly, the proposed sequence could be realized through addition of a carbo-dienophile following formation of intermediate **3**. The scope for the second Diels–Alder process was broad—with both electron-poor (**21a**–**21c**) and electron-rich dienophiles (**21d** and **21e**) being well tolerated, suggestive of a neutral electron-demand process.^31^ From a strategic perspective (Figure 6b), this reaction sequence can be regarded as a hybrid of diene-transmissive reactivity^32^ (in that a new diene is generated elsewhere in the molecule) and recursive reactivity^33^ (in that a dienophile is later incorporated within a new diene). To the best of our knowledge, this connection between two Diels–Alder reactions has never been realized, at least in close succession, and may hold potential as a generalizable strategy for rapid access to polycyclic systems through the combination of two or more cumulenic molecules (or equivalents thereof).

## Conclusions

We have established *N*-aryl ketenimines as versatile and highly reactive aza-dienophiles for the convergent synthesis of oxygenated pyridone derivatives under redox-neutral conditions. Peri-, regio-, chemo-, and diastereo-selectivity were all demonstrated upon reaction with 2,5-bis(silyloxy)furans, and detailed computational studies were employed to elucidate the origins of their unique reactivity. The success of ketenimines as aza-dienophiles can primarily be attributed to reduced distortion interactions during cycloaddition. This move away from FMO control eliminates the requirement of electron-withdrawing substituents bound to the aza-dienophile functionality that is not conducive to chemoselective C–O cleavage when furans are employed as dienes. Moreover, the semicyclic olefin generated from ketenimine cycloadditions appears to inhibit retrocycloaddition processes, and derivatization studies demonstrated that this functionality can be readily cleaved or modified. Overall, we anticipate that the methodology developed herein will permit the application of ketenimines in alternate settings. More generally, we hope this work will pave the way for the design of new highly reactive cumulenic heterodienophiles, an area we believe has much to offer.

## Supporting Information Available

Experimental details, characterization data, spectra, and computational studies (PDF)
