# Supplementary material for: Ketenimines as Aza-Dienophiles
Source: J Am Chem Soc. 2025 Feb 7;147(7):6087–94. doi: 10.1021/jacs.4c17174 (PMC11848816; doi:10.1021/jacs.4c17174)
Supplement: Supplementary file 1 — ja4c17174_si_001.pdf [file ja4c17174_si_001.pdf]

Ketenimines as Aza-Dienophiles

Christopher J. DeAngelis[a‡], Geeta Goyal[a‡], Marshall J. Liss[a‡], Jessica E. Budwitz[a], Mary Stuart Herlihy[a], Audrey V. Conner[a], Steven E. Wheeler[a], Pengchen Ma[b#], Miranda Li[b], K. N. Houk[b*], and Christopher G. Newton[a*]

[a] *Department of Chemistry, University of Georgia, Athens, Georgia 30602, United States*

[b] *Department of Chemistry and Biochemistry, University of California, Los Angeles, California 90095, United States*

[*] Email: houk@chem.ucla.edu

[*] Email: chris.newton@uga.edu

[‡] *C.J.D., G.G., and M.J.L. contributed equally to this work and are listed in alphabetical order*

PRIOR WORK 3

AZA-DIELS–ALDER REACTIONS OF FURANS 3
AZA-DIELS–ALDER REACTION OF KETENIMINE DIENOPHILES 5

EXPERIMENTAL PROCEDURES AND CHARACTERIZATION DATA 9

SYNTHESIS OF BIS(SILYLOXY)FURANS 9
Cyclic Anhydrides and their Precursors *9*
Bis(silyloxy)furans *19*
SYNTHESIS OF KETENIMINES 24
Ketenimine Precursors *24*
Anilines 24
Amides 25
Carbamates 37
Ketenimines *38*
KETENIMINE STABILITY STUDIES 50
BIS(SILYLOXY)FURAN + KETENIMINE AZA-DIELS–ALDER REACTIONS 53
Ring-Opened Products *53*
Direct Diels–Alder Cycloadducts (i.e., Non-Ring-Opened) *69*
Unsuccessful Diels–Alder Reactions *72*
DERIVATIZATION STUDIES 73
Allylic Transposition for the Synthesis of 2-Pyridones *73*
N-Aryl Ketenimines as Synthetic Equivalents of Other Aza-Dienophiles *77*
ONE-POT AZA-DIELS–ALDER/OXA-DIELS–ALDER SEQUENCES 80
COMPARING KETENIMINES AGAINST IMINO DIENOPHILES 87
Dienophile Synthesis *87*
Diels–Alder Reactions *89*
Rate Studies *98*

X-RAY CRYSTALLOGRAPHIC DATA 105

CALCULATIONS 114

[3,3] Sigmatropic Rearrangements *114*
LUMO energies and Diels–Alder Reactions *117*

NMR SPECTRA 133

SYNTHESIS OF BIS(SILYLOXY)FURANS 133
Cyclic Anhydrides and their Precursors *133*
Bis(silyloxy)furans *149*
SYNTHESIS OF KETENIMINES 162
Ketenimine Precursors *162*
Amides 162
Carbamates 193
Ketenimines *194*
BIS(SILYLOXY)FURAN + KETENIMINE AZA-DIELS–ALDER REACTIONS 229
Ring-Opened Products *229*
DERIVATIZATION STUDIES 282
Allylic Transposition for the Synthesis of 2-Pyridones *282*
N-Aryl Ketenimines as Synthetic Equivalents of Other Aza-Dienophiles *290*
ONE-POT AZA-DIELS–ALDER/OXA-DIELS–ALDER SEQUENCES 296
COMPARING KETENIMINES AGAINST IMINO DIENOPHILES 306
Dienophile Synthesis *306*
Diels–Alder Reactions *307*

REFERENCES 319

Prior Work

Aza-Diels–Alder Reactions of Furans

Summary

Of the 15 furyl aza-cycloadducts that have been reported, low yields, poor diastereoselectivities, and highly forcing conditions are all commonplace, with some derivatives also being described as “extremely unstable”. Moreover, several of these products were primarily characterized by elemental analysis, raising concerns regarding the reliability of these reports (more details below).

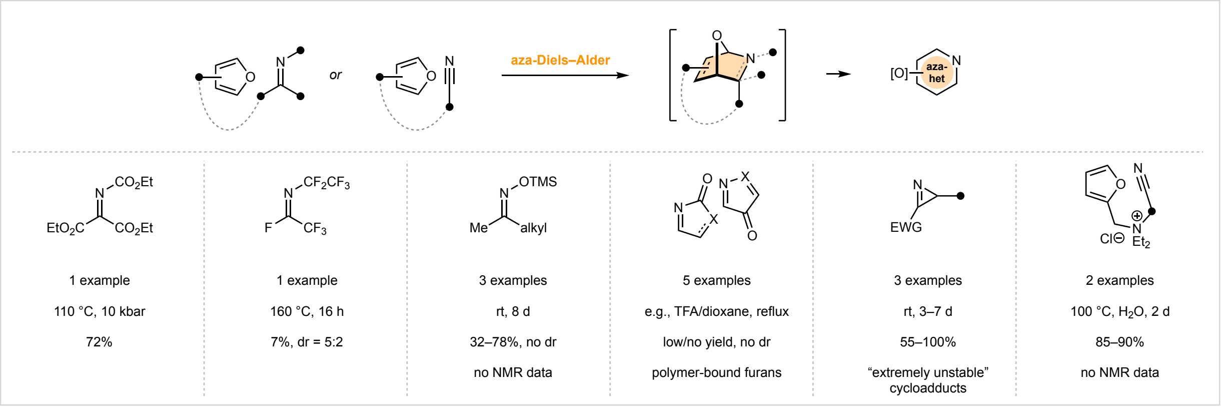

Full Details

C≡N Bonds as dienophiles

Reference: Babayan, 1974.[1]

In this report, two intramolecular Diels–Alder reactions across a C≡N bond are described, and the products were characterized by IR and elemental analysis. Mechanistic studies from the Rickborn laboratory demonstrate that isobenzofuran/benzonitrile (formal) Diels–Alder adducts undergo a retro-Diels–Alder reaction upon heating at 40 °C.[2] Given this, and a lack of NMR data, Rickborn brings into question the accuracy of Babayan’s disclosure.[3]

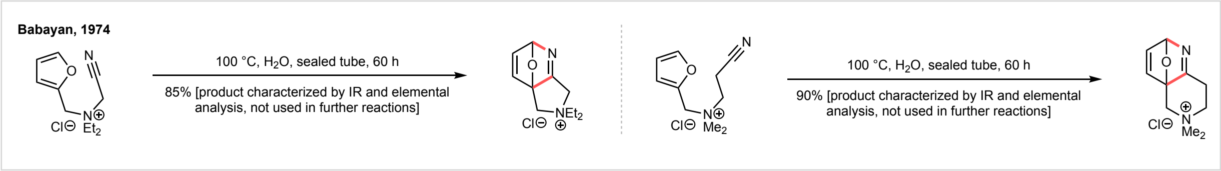

C=N Bonds as dienophiles

References: Plieninger 1972,[4] Knunynats 1988,[5] Martynov 1988,[6] Gaviña 1988,[7] Gaviña 1990,[8] Gaviña 1991,[9] Gilchrist 1998,[10] Gilchrist 2001.[11]

Of note, Martynov does not report NMR data, and to the best of our knowledge another report of *O*-silylated oximes behaving as aza-dienophiles has not been disclosed.

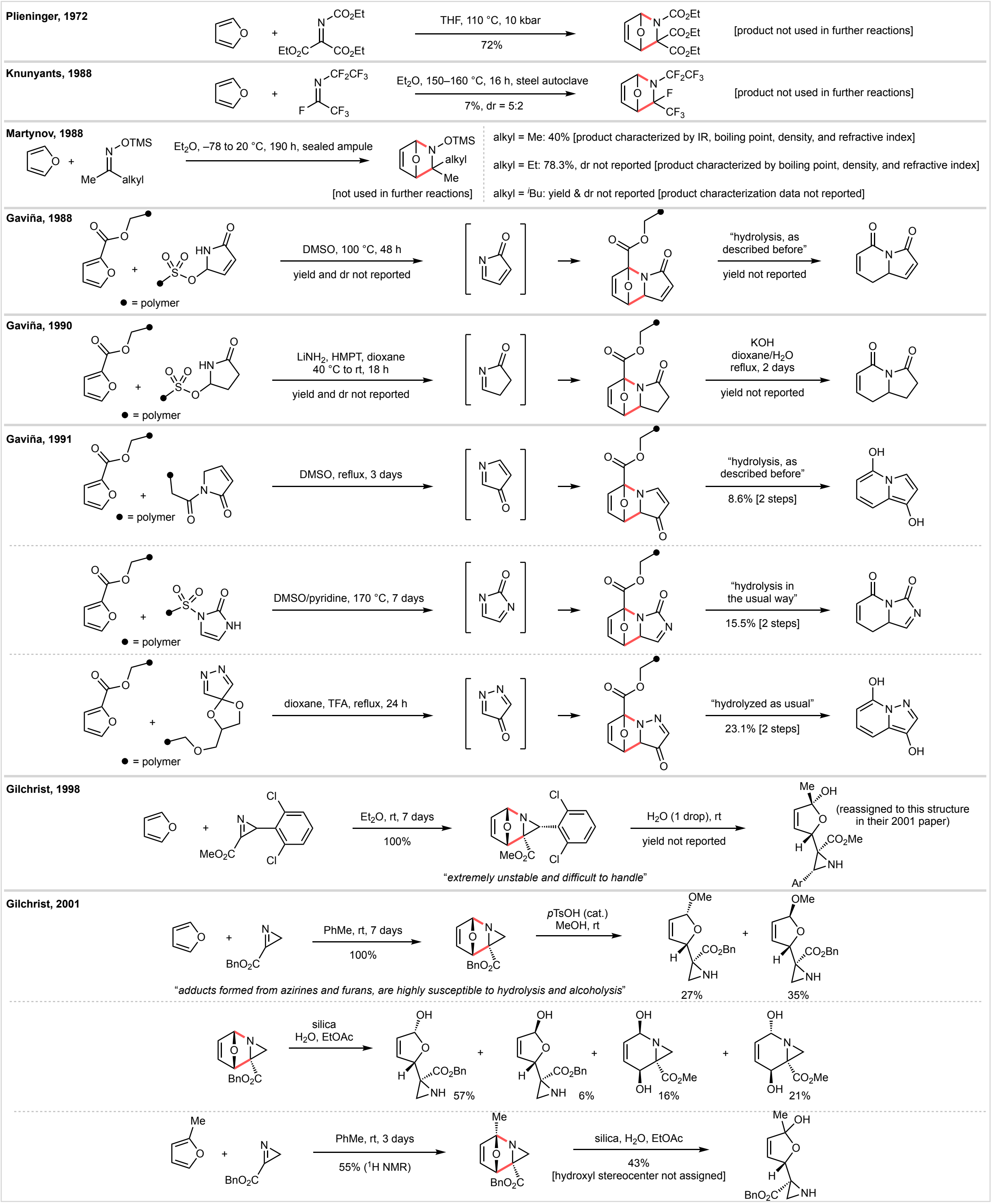

Aza-Diels–Alder Reaction of Ketenimine Dienophiles

References: Lectka 2012,[12] Sarpong 2014.[13]

Lectka explored ketenimine Diels–Alder reactivity with an excess of cyclopentadiene under copper(I)-catalyzed conditions, generating a diastereomeric mixture of aza-cycloadducts, followed by unavoidable cleavage of the newly formed *N*-heterocycle. Lectka recommended ketenimine preparation immediately prior to use, reporting *N*-aryl ketenimines are more stable when stored in solution, suggestive of an intermolecular decomposition pathway.

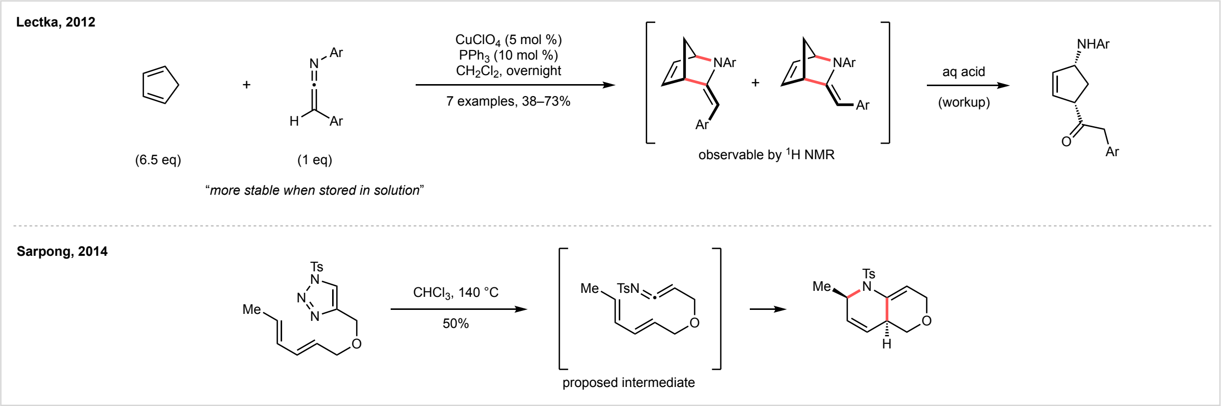

General Experimental

NMR Spectroscopy

^{1}H NMR spectra were recorded using either a Bruker 600 MHz Avance NEO console with an Oxford AS600 magnet, Bruker 600 MHz Avance NEO console with a Varian Premium Shielded 600 MHz magnet, Bruker 900 MHz Avance NEO console with an Oxford 600 MHz magnet, Avance 400 MHz III HD console with a Bruker Ascend 400 MHz magnet, or Agilent 600 MHz DD2 console with an Oxford 600 MHz magnet. ^{13}C NMR spectra were recorded using either a Bruker 600 MHz Avance NEO console with an Oxford AS600 magnet at 150 MHz, Bruker 600 MHz Avance NEO console with a Varian Premium Shielded 600 MHz magnet at 150 MHz, Bruker 900 MHz Avance NEO console with an Oxford NMR900 magnet at 225 MHz, or Avance 400MHz III HD console with a Bruker Ascend 400 MHz magnet at 100 MHz. ^{19}F NMR spectra were recorded using an Avance 400MHz III HD console on a Bruker Ascend 400 MHz magnet at 377 MHz. Residual solvent peaks were used as an internal reference for ^{1}H NMR spectra [$CDCl_3$ δ 7.26 ppm, CD_3CN δ 1.94 ppm, $(CD_3)_2CO$ δ 2.05 ppm, $(CD_3)_2SO$ δ 2.50 ppm, or CD_2Cl_2 δ 5.32 ppm] and ^{13}C NMR spectra [$CDCl_3$ δ 77.16 ppm, CD_3CN δ 118.26 ppm, $(CD_3)_2CO$ δ 29.84 ppm, $(CD_3)_2SO$ δ 39.52 ppm, or CD_2Cl_2 δ 53.80 ppm]. ^{19}F NMR spectra were reported relative to the ^{19}F resonance of C_6F_6 [$CDCl_3$ δ −161.64 ppm, or $(CD_3)_2SO$ δ −162.45 ppm]. Coupling constants (*J*) were quoted to the nearest 0.1 Hz. For ^{13}C NMR, coupling constants were included only in the case of coupling with ^{19}F nuclei. The following abbreviations (or combinations thereof) were used to describe ^{1}H NMR multiplicities: s = singlet, d = doublet, t = triplet, q = quartet, p = pentet, m = multiplet, br = broad, app = apparent.

Infrared Spectroscopy

IR spectra were recorded neat on a Thermo Nicolet iS10 spectrometer and are reported in wavenumbers (cm^{-1}).

Mass Spectrometry

High resolution mass spectrometry (HRMS) data were acquired via electrospray ionization (ESI) using either a Q-TOF mass spectrometer produced by Bruker model Impact II or Bruker Solarix XR 12 T FTICR MS.

Chromatography

Flash chromatography was performed with SiliaFlash® P60 silica, 0.040–0.063 mm grade. Analytical thin-layer chromatography was performed with commercial glass sheets coated with 0.25 mm silica gel (SiliaPlate™, silica gel 60, F254). Compounds were either visualized under UV-light at 254 nm, or by dipping the plates in an aqueous potassium permanganate solution followed by heating, unless stated otherwise. All R_f values were measured to the nearest 0.1 cm.

Melting Points

Melting points were measured on a DigiMelt melting point apparatus, model SRS MPA161, and are uncorrected.

Optical Rotations

Optical rotations were recorded on an AUTOPOL III Automatic Polarimeter with a path length of 10 dm at the specified temperature.

Preparation of Phosphate Buffered Silica (pH = 7)

Prepared according to the method of Newton.[14] To a 5.00 L Erlenmeyer flask open to air and equipped with stir bar was added 3.80 L of deionized water. Sodium phosphate dibasic (114 g, 800 mmol) was slowly added with vigorous stirring, followed by additional deionized water to reach a total volume of 4000 mL (0.20 M). Once fully dissolved (ca. 10 minutes), 400 g of silica gel was slowly added. The pH of the mixture was measured (pH paper, range 1–13) to confirm the solution is neutral. If basic, the mixture was neutralized by either: (i) dropwise addition of phosphoric acid or (ii) addition of further silica gel (10.0 g portions). If acidic, additional phosphate buffer (0.2 M, prepared as above) is added. Once neutral, the mixture was filtered through a 2.00 L, 13.5 cm diameter sintered funnel (medium grit), then air was pulled through the funnel for 30 minutes. The phosphate buffered silica was transferred to a 2.00 L shallow glass dish and placed in an oven (105 °C) for three days, stirring every day to ensure even drying. The phosphate buffered silica was allowed to cool to ambient temperature and was sifted through a sieve into a container for long term storage. **Appearance**: White, free-flowing solid that partially adheres to glass when wet with solvent. **Note**: When using phosphate buffered silica, the addition of acidified sand to ensure a level silica gel line should be avoided. Instead, we recommend using additional phosphate buffered silica as a sand replacement.

Experimental Procedures and Reagents

Commercially available chemicals were used as purchased or, where specified, purified by standard techniques. Solvent compositions are given in v/v. All reactions were carried out under an atmosphere of argon in flame-dried glassware unless otherwise indicated. All reactions underwent magnetic stirring employing an IKA plate, with heating facilitated by either an OptiTherm® heating mantle or a silicon oil bath when required. MeCN, CH_2Cl_2, THF, Et_2O, and toluene were purified by an Mbraun solvent purification system. All other solvents were used as purchased or, where specified, purified by standard techniques.

Microwave

Reaction conditions invoking microwave irradiation were carried out in a CEM Discover 2.0 microwave synthesis system.

X-Ray Crystallography

X-ray data were measured at room temperature on a Bruker D8 Quest PHOTON 100 CMOS X-ray diffractometer system with Incoatec Microfocus Source (IμS) monochromated Mo K(alpha) radiation (lambda = 0.71073 Å, sealed tube) using phi and omega-scan technique. The data were integrated with the manufacturer's SAINT software and corrected for absorption effects using the Multi-Scan method (SADABS). The structure was solved and refined using the Bruker SHELXTL Software Package.[15] Non-hydrogen atoms were located from successive difference Fourier map calculations. In the final cycles of each refinement, all the non-hydrogen atoms were refined in anisotropic displacement parameters. All the hydrogen atom positions were calculated at geometrical positions and allowed to ride on the carbon to which they are bonded. Hydrogen atom temperature factors were fixed at n (n = 1.2 for PhH, CH, and CH_2 groups, n = 1.5 for CH_3 groups) times the isotropic temperature factors of the C–atoms to which they are bonded. Graphical representations of the X-ray structures were generated using CYLview20 (Legault, C. Y., Université de Sherbrooke, 2020, http://www.cylview.org).

General Comments on Compound Characterization

All new compounds were fully characterized. For compounds that have been previously prepared in the literature, if we modified their procedure we include a description of appearance, R_f, and ^{1}H NMR data, alongside our updated procedure.

Experimental Procedures and Characterization Data

Synthesis of Bis(silyloxy)furans

Cyclic Anhydrides and their Precursors

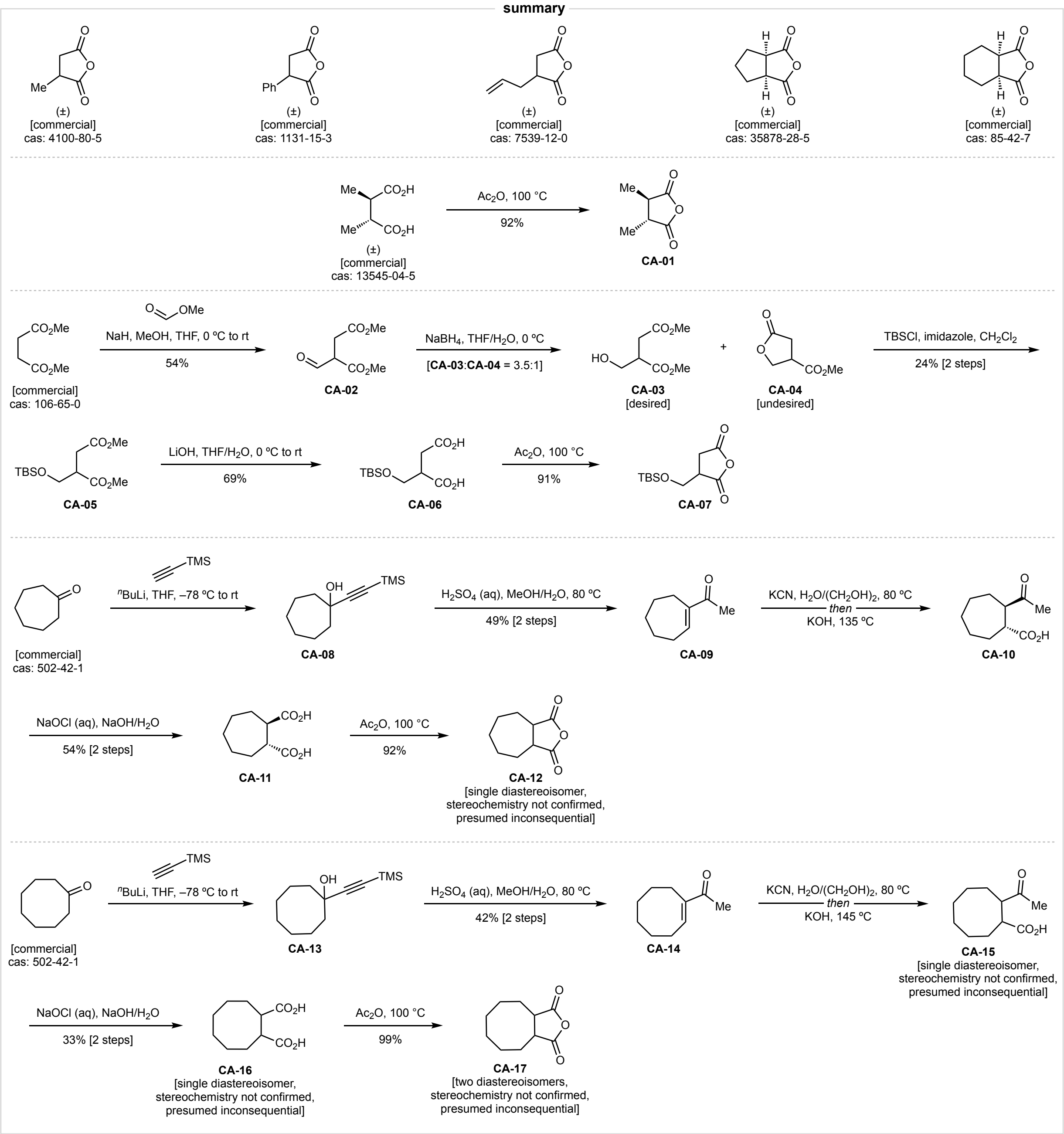

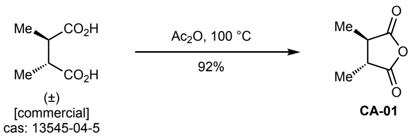

Prepared according to a modification of the procedure reported by Newton.[16] To a flame-dried 100 mL one-neck round-bottom flask under argon and equipped with a stir bar and high efficiency air condenser was added 2,3-dimethylsuccinic acid (1.20 g, 8.21 mmol, 1.00 equiv) and acetic anhydride (8.21 mL, 1.0 M). The reaction was warmed on a heating mantle at 100 °C for 4 hours, cooled to ambient temperature, and concentrated under reduced pressure. The residue was co-evaporated with toluene (×3) then filtered over phosphate buffered silica (pH = 7, ratio of buffered silica to crude mass = 10:1, eluting with Et_2O). The filtrate was concentrated under reduced pressure and again co-evaporated with toluene (×3) to remove remaining traces of acetic anhydride/acetic acid, yielding **CA-01**.

Characterization data matched those reported by Evans.[17]

Yield: 972 mg, 7.59 mmol, 92%;

Appearance: White solid;

R*f*: 0.28 (4:1 hexane:EtOAc, visualized with bromocresol green);

^{1}H NMR (600 MHz, $CDCl_3$): δ 2.80 – 2.71 (m, 2H), 1.42 (dd, J = 6.9 Hz, 6H) ppm.

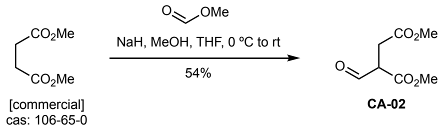

Prepared according to a modification of the procedure reported by Soós.[18] To a flame-dried 250 mL three-neck round-bottom flask under argon and equipped with a stir bar was added anhydrous THF (90.0 mL, 0.76 M) and NaH (60% dispersion in mineral oil, 4.13 g, 103 mmol, 1.51 equiv). The reaction flask was submerged in an ice/water bath and methanol (0.277 mL, 6.84 mmol, 0.100 equiv), dimethyl succinate (10.0 g, 68.4 mmol, 1.00 equiv), and methyl formate (27.0 mL, 439 mmol, 6.41 equiv) were sequentially added dropwise. The ice/water bath was removed, and the reaction was allowed to stir overnight at ambient temperature. The crude mixture was concentrated under reduced pressure, diluted with water, and carefully acidified with 2N HCl (pH = 3). The aqueous layer was extracted with CH_2Cl_2 (×3), and the combined organics were washed with brine, dried over Na_2SO_4, filtered, and concentrated under reduced pressure. The residue was purified via vacuum distillation to yield **CA-02**.

Characterization data matched those reported by Soós.[18]

Yield: 6.39 g, 36.7 mmol, 54%;

Appearance: Pale yellow oil;

R*f*: 0.43 (5:95 MeOH:CH_2Cl_2);

^{1}H NMR [600 MHz, $(CD_3)_2SO$]: δ 10.89 (s, 1H), 7.70 (s, 1H), 3.59 (s, 3H), 3.56 (s, 3H), 3.16 (s, 2H) ppm.

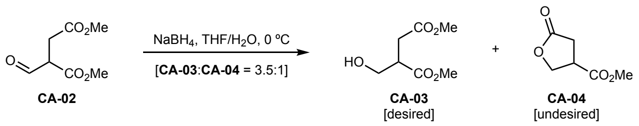

Prepared according to a modification of a procedure reported by Shenvi.[19] To a 500 mL one-neck round-bottom flask open to air and equipped with a stir bar was added **CA-02** (5.90 g, 33.9 mmol, 1.00 equiv) and THF/H_2O (24:1, 172 mL, 0.20 M). The reaction flask was submerged in an ice/water bath and $NaBH_4$ (2.56 g, 67.8 mmol, 2.00 equiv) was added portion-wise. The reaction was placed under argon and allowed to stir for one hour by which time TLC analysis indicated complete consumption of **CA-02**. The reaction was diluted with CH_2Cl_2, quenched with saturated aqueous NH_4Cl, and removed from the ice/water bath. The layers were separated, and the aqueous layer was extracted with CH_2Cl_2 (×3). The combined organics were washed with saturated aqueous $NaHCO_3$ and brine, dried over Na_2SO_4, filtered, and concentrated under reduced pressure to yield a mixture of **CA-03** and **CA-04**. This crude mixture was submitted to the next reaction without further purification. For characterization purposes, a small amount of pure **CA-03** was isolated via flash column chromatography (ratio of silica to crude mass = 50:1, eluting with a gradient of 1:9 Et_2O:CH_2Cl_2 to 1:1 Et_2O:CH_2Cl_2).

Characterization data matched those reported by Ishida.[20]

Appearance: Colorless oil;

R*f*: 0.43 (1:1 Et_2O:CH_2Cl_2);

^{1}H NMR (600 MHz, $CDCl_3$): δ 3.87 – 3.77 (m, 2H), 3.72 (s, 3H), 3.68 (s, 3H), 3.03 (p, *J* = 6.5, 6.0 Hz, 1H), 2.76 (dd, *J* = 16.9, 7.2 Hz, 1H), 2.63 (dd, *J* = 16.9, 6.5 Hz, 1H), 2.50 (br s, 1H) ppm.

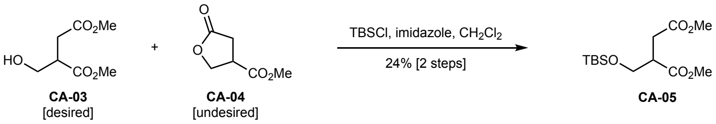

To a flame-dried 100 mL one-neck round-bottom flask under argon and equipped with a stir bar was added a crude mixture of **CA-03** and **CA-04** (2.31 g, ratio of **CA-03**:**CA-04** = 3.5:1, 10.6 mmol of **CA-03**, 1.00 equiv), anhydrous CH_2Cl_2 (11.2 mL, 0.95 M with respect to **CA-03**), imidazole (2.66 g, 39.0 mmol, 3.68 equiv with respect to **CA-03**), and TBSCl (2.94 g, 19.5 mmol, 1.84 equiv with respect to **CA-03**). The reaction was allowed to stir at ambient temperature overnight before quenching with saturated aqueous NH_4Cl. The layers were separated, and the aqueous layer was extracted with Et_2O (×3). The combined organics were washed with brine, dried over Na_2SO_4, filtered, and concentrated under reduced pressure. The crude residue was purified via flash column chromatography (ratio of silica to crude mass = 50:1, eluting with a gradient of hexane to 5:95 EtOAc:hexane) to yield **CA-05**.

Yield: 2.37 g, 8.17 mmol, 24% yield over 2 steps;

Appearance: Pale yellow oil;

R*f*: 0.20 (1:9 EtOAc:hexane);

^{1}H NMR (600 MHz, $CDCl_3$): δ 3.81 (qd, *J* = 9.9, 5.5 Hz, 2H), 3.69 (s, 3H), 3.68 (s, 3H), 3.07 – 2.99 (m, 1H), 2.80 (dd, *J* = 16.9, 8.5 Hz, 1H), 2.58 (dd, *J* = 16.9, 5.5 Hz, 1H), 0.86 (s, 9H), 0.02 (app d, *J* = 2.1 Hz, 6H) ppm;

^{13}C NMR (150 MHz, $CDCl_3$): δ 173.4, 172.8, 63.3, 52.0, 51.9, 44.3, 32.6, 25.9, 18.3, –5.5 ppm;

IR: 2954, 2930, 2858, 1743, 1439, 1704, 1731, 1463, 1439, 1254 cm^{-1};

HRMS (ESI): calculated for $[C_{13}H_{26}O_5Si+H]^+$: 291.1622, found: 291.1625.

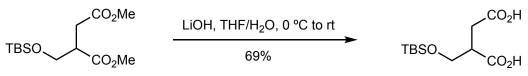

CA-05 **CA-06**

To a 250 mL one-neck round-bottom flask open to air and equipped with a stir bar was added **CA-05** (2.20 g, 7.57 mmol, 1.00 equiv) and THF/H_2O (3:1, 40.0 mL, 0.19 M). The reaction flask was submerged in an ice/water bath, and LiOH (0.544 g, 22.7 mmol, 3.00 equiv) was added in one portion. The reaction was placed under argon, the ice/water bath removed, and the reaction was allowed to stir overnight at ambient temperature. The flask was submerged in an ice/water bath, and the reaction mixture carefully acidified with 2N HCl (pH = 3). The reaction was extracted with EtOAc (×3), and the combined organics were dried over Na_2SO_4, filtered, and concentrated under reduced pressure. Trituration with hexane yielded **CA-06**.

Yield: 1.37 g, 5.23 mmol, 69%;

Appearance: White solid;

R*f*: N/A (streaks on glass-backed silica TLC plates);

^{1}H NMR (600 MHz, $CDCl_3$): δ 3.91 – 3.82 (m, 2H), 3.08 – 3.01 (m, 1H), 2.87 (dd, *J* = 17.4, 9.8 Hz, 1H), 2.62 (dd, *J* = 17.5, 4.3 Hz, 1H), 0.87 (s, 9H), 0.05 (app d, *J* = 3.2 Hz, 6H) ppm; (note: exchangeable CO_2H protons not observed)

^{13}C NMR (100 MHz, $CDCl_3$): δ 179.0, 178.7, 63.0, 44.2, 32.7, 25.9, 18.3, –5.5 ppm;

IR: 2931, 2887, 2859, 1721, 1708, 1692, 1470, 1422, 1308, 1250, 1182, cm^{-1};

HRMS (ESI): calculated for $[C_{11}H_{22}O_5Si+H]^+$: 263.1309, found: 263.1309.

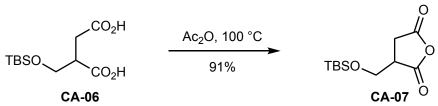

CA-06 **CA-07**

Prepared according to a modification of the procedure reported by Newton.[16] To a flame-dried 50 mL one-neck round-bottom flask under argon and equipped with a stir bar and high efficiency air condenser was added **CA-06** (1.33 g, 5.10 mmol, 1.00 equiv) and acetic anhydride (10.2 mL, 0.50 M). The reaction was warmed on a heating mantle at 100 °C for 4 hours, cooled to ambient temperature, and concentrated under reduced pressure. The residue was co-

evaporated with toluene (×3) then filtered over phosphate buffered silica (pH = 7, ratio of buffered silica to crude mass = 10:1, eluting with Et_2O) to yield **CA-07**.

Characterization data matched those reported by Coates.[21]

Yield: 1.14 g, 4.65 mmol, 91%;

Appearance: Pale yellow oil;

R*f*: N/A (streaks on glass-backed silica TLC plates);

^{1}H NMR (600 MHz, $CDCl_3$): δ 4.12 (dd, *J* = 10.0, 2.5 Hz, 1H), 3.74 (dd, *J* = 10.0, 2.8 Hz, 1H), 3.24 – 3.17 (m, 1H), 2.99 (qd, *J* = 18.5, 7.2 Hz, 2H), 0.83 (s, 9H), 0.04 (app d, *J* = 4.8 Hz, 6H) ppm.

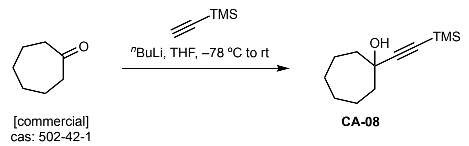

Prepared according to a modification of the procedure reported by Yamada.[22] To a flame-dried 500 mL three-neck round-bottom flask under argon and equipped with a stir bar was added ethynyltrimethylsilane (13.7 mL, 98.1 mmol, 1.10 equiv) and anhydrous THF (81.5 mL, 1.1 M). The reaction flask was submerged in an acetone/dry ice bath, and stirred for 15 minutes before freshly titrated[23] *n*-butyllithium (2.25 M in hexane, 47.6 mL, 107 mmol, 1.20 equiv) was added dropwise. The acetone/dry ice bath was removed, and the reaction was allowed to warm to ambient temperature and stir for 30 minutes. Cycloheptanone (10.0 g, 89.2 mmol, 1.00 equiv) was added dropwise as a solution in anhydrous THF (17.8 mL, 5.0 M), and the reaction was stirred for five hours. The reaction flask was submerged in an ice/water bath and carefully quenched with saturated aqueous NH_4Cl. The reaction mixture was diluted with water and extracted with Et_2O (×3). The combined organics were washed with brine, dried over $MgSO_4$, filtered, and concentrated under reduced pressure to yield crude **CA-08** as a brown oil. This was submitted to the next reaction without further purification.

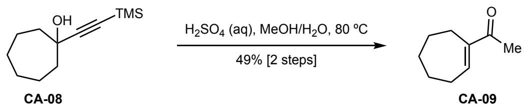

Prepared according to a modification of the procedure reported by Yamada.[22] To a 1 L one-neck round-bottom flask open to air and equipped with a stir bar was added $MeOH/H_2O$ (1:1, 308 mL) and H_2SO_4 (307 mL, 0.29 M, warning: exothermic). Once the solution reached ambient temperature, crude **CA-08** was added (small volume of MeOH employed to facilitate transfer). The reaction was warmed on a heating mantle at 80 °C for 3 hours, cooled to ambient temperature, concentrated under reduced pressure, diluted with H_2O, and extracted with Et_2O (×3). The combined organics were washed with $NaHCO_3$ and brine, dried over $MgSO_4$, filtered, and concentrated under reduced pressure.

The residue was purified via flash column chromatography (ratio of silica to crude mass = 30:1, eluting with 10:1 hexane:EtOAc) to yield **CA-09**.

Characterization data is in close agreement with those reported by Gimeno.[24]

Yield: 6.08 g, 44.0 mmol, 49% over 2 steps;

Appearance: Brown oil;

R*f*: 0.50 (1:4 EtOAc:hexane);

^{1}H NMR (600 MHz, $CDCl_3$): δ 7.08 (t, *J* = 6.7 Hz, 1H), 2.50 – 2.45 (m, 2H), 2.34 (q, *J* = 6.3 Hz, 2H), 2.29 (s, 3H), 1.77 (p, 2H), 1.53 (p, *J* = 5.8 Hz, 2H), 1.43 (p, *J* = 5.7 Hz, 2H) ppm.

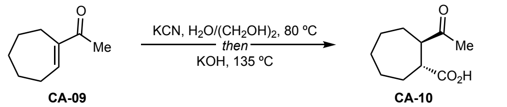

Prepared according to a modification of the procedure reported Raphael[25] and private communication with Saito.[26] To a 100 mL one-neck round-bottom flask open to air and equipped with a stir bar and high efficiency air condenser was added potassium cyanide (2.36 g, 36.2 mmol, 2.50 equiv), H_2O (3.86 mL), ethylene glycol (14.5 mL), and **CA-09** (2.00 g, 14.5 mmol, 1.00 equiv). The reaction was placed under argon and warmed in an oil bath at 80 °C for 2 hours. KOH (1.35 g, 24.0 mmol, 1.66 equiv) was added and the reaction was heated to 135 °C for 14 hours. The oil bath was removed, and the reaction was cooled to ambient temperature, diluted with water, and washed with Et_2O (×2, organics discarded). The aqueous layer was carefully acidified with 2N HCl (pH = 1) and extracted with Et_2O (×3). The combined organics were washed with brine, dried over Na_2SO_4, filtered, and concentrated under reduced pressure to yield crude **CA-10** as a brown oil. This was submitted to the next reaction without further purification.

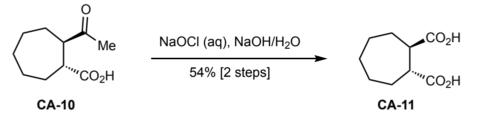

Prepared according to a modification of the procedure reported by Schreiner.[27] To a 250 mL one-neck round-bottom flask open to air and equipped with a stir bar was added crude **CA-10**, aqueous NaOCl (10–15% active chlorine, 58.0 mL, 0.25 M), and NaOH (0.872 g, 21.8 mmol, 1.50 equiv) as a solution in water (6.59 mL, 2.2 M). The reaction was placed under argon and stirred for 1 hour (white precipitate forms). The round-bottom flask was submerged in an ice/water bath, quenched with sodium metabisulfite, and carefully acidified with 2N HCl (pH = 1). The mixture was extracted with Et_2O (×3), washed with brine, dried over $MgSO_4$, filtered, and concentrated under reduced pressure. Trituration with hexane yielded **CA-11**.

Characterization data matched those reported by Schreiner.[27]

Yield: 1.46 g, 7.82 mmol, 54% over 2 steps;

Appearance: White solid;

R*f*: N/A (streaks on glass-backed silica TLC plates);

^{1}H NMR (400 MHz, $CDCl_3$): δ 11.31 (br s, 2H), 2.86 – 2.77 (m, 2H), 2.12 – 2.00 (m, 2H), 1.81 – 1.56 (m, 8H) ppm.

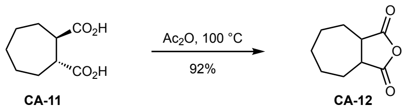

CA-11 **CA-12**
[single diastereoisomer, stereochemistry not confirmed, presumed inconsequential]

Prepared according to a modification of the procedure reported by Newton.[16] To a flame-dried 50 mL one-neck round-bottom flask under argon and equipped with a stir bar and high efficiency air condenser was added **CA-11** (1.20 g, 6.44 mmol, 1.00 equiv) and acetic anhydride (6.44 mL, 1.0 M). The reaction was warmed on a heating mantle at 100 °C for 4 hours, cooled to ambient temperature, and concentrated under reduced pressure. The residue was co-evaporated with anhydrous toluene (×3), filtered over silica (ratio of silica to crude mass = 10:1, eluting with Et_2O). The filtrate was concentrated under reduced pressure to yield **CA-12**.

Yield: 998 mg, 5.93 mmol, 92%;

Appearance: White solid;

R*f*: N/A (streaks on glass-backed silica TLC plates);

^{1}H NMR (600 MHz, $CDCl_3$): δ 2.98 – 2.91 (m, 2H), 2.40 – 2.30 (m, 2H), 1.77 – 1.62 (m, 6H), 1.59 – 1.53 (m, 2H) ppm;

^{13}C NMR (150 MHz, $CDCl_3$): δ 172.7, 46.3, 27.5, 27.1, 25.1 ppm;

IR: 2938, 2862, 1864, 1795, 1453, 1253, 1218, 1024, 932, 895cm^{-1};

HRMS (ESI): calculated for $[C_9H_{12}O_3+H]^+$: 169.0865, found: 169.0857.

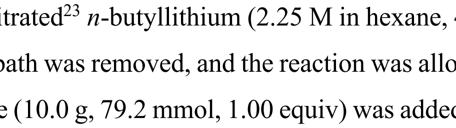

[commercial]
cas: 502-42-1
CA-13

Prepared according to a modification of the procedure reported above for **CA-08**. To a flame-dried 500 mL three-neck round-bottom flask under argon and equipped with a stir bar was added ethynyltrimethylsilane (12.2 mL, 87.2 mmol, 1.10 equiv) and anhydrous THF (72.0 mL, 1.1 M). The reaction flask was submerged in an acetone/dry ice bath and stirred for 15 minutes before freshly titrated[23] *n*-butyllithium (2.25 M in hexane, 42.3 mL, 107 mmol, 1.20 equiv) was added dropwise. The acetone/dry ice bath was removed, and the reaction was allowed to warm to ambient temperature and stir for 30 minutes. Cyclooctanone (10.0 g, 79.2 mmol, 1.00 equiv) was added dropwise as a solution in anhydrous THF (15.8 mL, 5.0 M), and the reaction was stirred for 5 hours. The reaction flask was submerged in an ice/water

bath and carefully quenched with saturated aqueous NH_4Cl. The reaction mixture was diluted with water and extracted with Et_2O (×3). The combined organics were washed with brine, dried over $MgSO_4$, filtered, and concentrated under reduced pressure to yield crude **CA-13** as a brown oil. This was submitted to the next reaction without further purification.

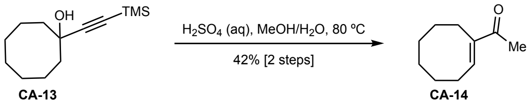

Prepared according to a modification of the procedure reported above for **CA-09**. To a 1 L one-neck round-bottom flask open to air and equipped with a stir bar was added MeOH/H_2O (1:1, 272 mL) and H_2SO_4 (273 mL, 0.29 M warning: exothermic). Once the solution reached ambient temperature, crude **CA-13** was added (small volume of MeOH employed to facilitate transfer). The reaction was warmed on a heating mantle at 80 °C for 3 hours, cooled to ambient temperature, concentrated under reduced pressure, diluted with H_2O, and extracted with Et_2O (×3). The combined organics were washed with $NaHCO_3$ and brine, dried over $MgSO_4$, filtered, and concentrated under reduced pressure. The residue was purified via flash column chromatography (ratio of silica to crude mass = 30:1, eluting with 10:1 hexane:EtOAc) to yield **CA-14**.

Characterization data are in close agreement with those reported by Gimeno.[24]

Yield: 5.09 g, 33.4 mmol, 42% over 2 steps;

Appearance: Yellow oil;

R*f*: 0.24 (5:95 EtOAc:hexane);

^{1}H NMR (600 MHz, $CDCl_3$): δ 6.85 (t, *J* = 8.3 Hz, 1H), 2.42 (t, *J* = 6.3 Hz, 2H), 2.35 – 2.29 (m, 2H), 2.28 (s, 3H), 1.64 – 1.56 (m, 2H), 1.53 – 1.47 (m, 2H), 1.47 – 1.37 (m, 4H) ppm.

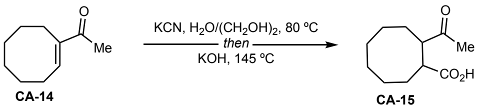

CA-15
[single diastereoisomer, stereochemistry not confirmed, presumed inconsequential]

Prepared according to a modification of the procedure reported above for **CA-10**. To a 100 mL one-neck round-bottom flask open to air and equipped with a stir bar and high efficiency air condenser was added potassium cyanide (2.14 g, 32.8 mmol, 2.5 equiv), H_2O (3.50 mL), ethylene glycol (13.1 mL), and **CA-14** (2.00 g, 13.1 mmol, 1.00 equiv). The reaction was placed under argon and warmed in an oil bath at 80 °C for 2 hours, KOH (1.22 g, 21.8 mmol, 1.66 equiv) was added, and the reaction was heated to 145 °C for 14 hours. The oil bath was removed, and the reaction was cooled to ambient temperature, diluted with water, and washed with Et_2O (×2, organics discarded). The aqueous layer was carefully acidified with 2N HCl (pH = 1) and extracted with Et_2O (×3). The combined organics were washed with

brine, dried over Na_2SO_4, filtered, and concentrated under reduced pressure to yield crude **CA-15** as a brown oil. This was submitted to the next reaction without further purification.

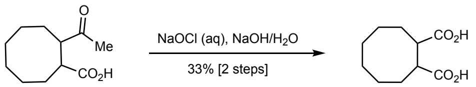

CA-15
[single diastereoisomer, stereochemistry not confirmed, presumed inconsequential]

CA-16
[single diastereoisomer, stereochemistry not confirmed, presumed inconsequential]

Prepared according to a modification of the procedure reported above for **CA-11**. To a 250 mL one-neck round-bottom flask open to air and equipped with a stir bar was added crude **CA-15**, aqueous NaOCl (10–15% active chlorine, 52.4 mL, 0.25 M) and NaOH (0.786 g, 19.7 mmol, 1.50 equiv) as a solution in water (5.95 mL, 2.2 M). The reaction was placed under argon and stirred for 1 hour (white precipitate forms). The round-bottom flask submerged in an ice/water bath, quenched with sodium metabisulfite, and carefully acidified with 2N HCl. The mixture was extracted with Et_2O (×3), washed with brine, dried over $MgSO_4$, filtered, and concentrated under reduced pressure. Trituration with hexane yielded **CA-16**.

Characterization data are in close agreement with those reported by Law and Castellano.[28]

Yield: 862 mg, 4.30 mmol, 33% over 2 steps;

Appearance: White solid;

R*f*: N/A (streaks on glass-backed silica TLC plates);

^{1}H NMR [600 MHz, $(CD_3)_2SO$]: δ 12.06 (s, 2H), 2.72 (s, 2H), 1.88 – 1.80 (m, 2H), 1.71 – 1.63 (m, 2H), 1.61 – 1.52 (m, 4H), 1.51 – 1.36 (m, 4H) ppm.

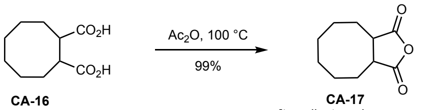

CA-16
[single diastereoisomer, stereochemistry not confirmed, presumed inconsequential]

CA-17
[two diastereoisomers, stereochemistry not confirmed, presumed inconsequential]

Prepared according to a modification of the procedure reported by Newton.[16] To a flame-dried 25 mL one-neck round-bottom flask under argon and equipped with a stir bar and high efficiency air condenser was added **CA-16** (0.750 g, 3.75 mmol, 1.00 equiv) and acetic anhydride (3.75 mL, 1.0 M). The reaction was warmed on a heating mantle at 100 °C for 4 hours, cooled to ambient temperature, and concentrated under reduced pressure. The residue was co-evaporated with anhydrous toluene (×3) and filtered over phosphate buffered silica (pH = 7, ratio of buffered silica to crude mass = 10:1, eluting with Et_2O). The filtrate was concentrated under reduced pressure to yield **CA-17**.

Yield: 0.608 g, 3.34 mmol, 89%;

Appearance: Yellow/brown oil;

R*f*: 0.14 (1:9 EtOAc:hexane);

^{1}H NMR (600 MHz, $CDCl_3$, only major diastereoisomer indicated): δ 2.96 – 2.92 (m, 2H), 2.35 – 2.32 (m, 2H), 1.78 – 1.85 (m, 4H), 1.59 – 1.56 (m, 4H), 1.36 – 1.42 (m, 2H) ppm;

^{13}C NMR (100 MHz, $CDCl_3$, only major diastereoisomer indicated): δ 173.8, 45.8, 30.2, 26.7, 24.5 ppm;

IR: 2932, 2860, 1851, 1840, 1778, 1468, 1450, 1371, 1270, 1231 cm^{-1};

HRMS (ESI): calculated for $[C_{10}H_{14}O_3+H]^+$: 183.1016, found: 183.1016.

Bis(silyloxy)furans

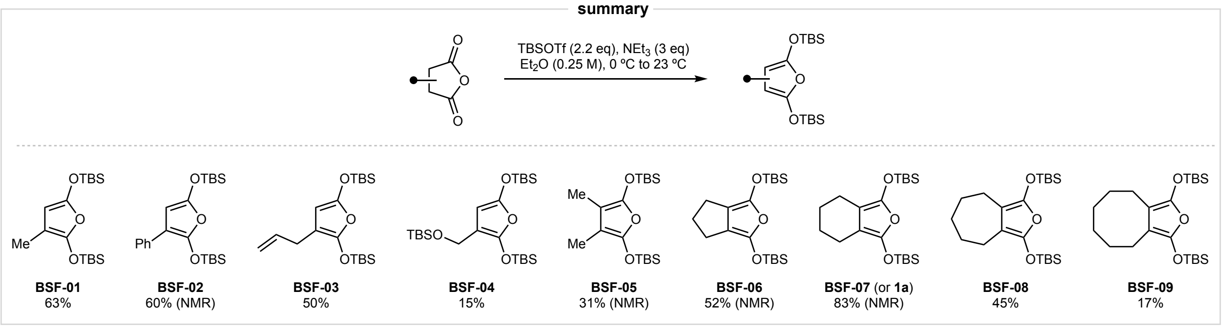

General Procedure

Prepared according to a modification of the procedure reported by Newton.[14, 16] To a flame-dried round-bottom flask equipped with a stir bar and under argon was added the appropriate cyclic anhydride (1.00 equiv). Following addition of anhydrous Et_2O (0.25 M), the reaction flask was submerged in an ice/water bath and anhydrous triethylamine (3.00 equiv) was added. After stirring for 10 minutes, *tert*-butyldimethylsilyl trifluoromethanesulfonate (2.20 equiv) was added dropwise and the reaction mixture was allowed to warm to ambient temperature. The reaction was stirred vigorously overnight to yield a biphasic mixture. The crude mixture was filtered over phosphate buffered silica (pH = 7, ratio of buffered silica to theoretical crude mass = 5:1, eluting with Et_2O) and the filtrate was concentrated under reduced pressure. Further purification details, if pursued, are provided alongside the characterization data for each furan.

Notes on Yields and Purity

Yields tend to improve on scale (e.g., crude **BSF-01** was isolated in 82% yield and in 85% purity on >20 g scale in our Organic Syntheses report[14]). The most prominent impurities present within crude bis(silyloxy)furans tend to be two TBSOTf-derived compounds (i.e., siloxane and silanol). In principle, these can be removed through vacuum distillation techniques (e.g., in the aforementioned Organic Syntheses report, further purification provided **BSF-01** in 74% yield and in 97% purity on 22.2 g scale). Within the present study we found these impurities did not impact the Diels–Alder reaction, thus attempts to remove them were not pursued. A ^{1}H NMR of these impurities is included within our Organic Syntheses report (see Figure 10), and their chemical shifts are as follows:

^{1}H NMR (400 MHz, $CDCl_3$): δ 0.91, 0.86, 0.10, 0.01 ppm.

^{13}C NMR (100 MHz, $CDCl_3$): δ 25.90, –2.77 ppm.

Notes on Stability

As discussed within our previous reports, 2,5-bis(*tert*-butyldimethylsilyloxy)furans are prone to decomposition if not stored appropriately.[14, 16] We typically recommend employing them in subsequent reactions within a few days of their

preparation, however many derivatives can be stored neat under argon in the freezer (−20 °C) in the presence of catalytic BHT for days-to-weeks with minimal decomposition.

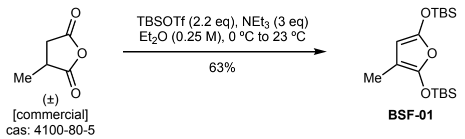

Further Purification: Flash column chromatography using phosphate buffered silica (pH = 7, ratio of buffered silica to theoretical crude mass = 10:1, eluting with hexane).

For characterization data of a further purified sample, see our previous report.[14]

Yield: 1.89 g, 5.52 mmol, 63%;

Appearance: Colorless oil;

R*f*: N/A (decomposes on glass-backed silica TLC plates);

^{1}H NMR (400 MHz, $CDCl_3$): δ 4.81 (s, 1H), 1.75 (s, 3H), 0.96 (app d, *J* = 8.2 Hz, 18H), 0.19 (app d, *J* = 4.9 Hz, 12H) ppm.

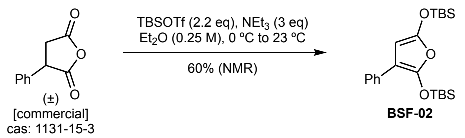

Crude furan submitted to the next reaction without further purification.

For characterization data of a further purified sample, see our previous report.[16]

Crude Yield: 1.71 mmol, 60% (NMR yield, determined using durene as internal standard);

Appearance: Yellow/orange oil;

R*f*: N/A (decomposes on glass-backed silica TLC plates);

^{1}H NMR (400 MHz, $CDCl_3$): δ 7.51 (d, *J* = 8.3 Hz, 2H), 7.30 (t, *J* = 7.8 Hz, 2H), 7.10 (t, *J* = 7.4 Hz, 1H), 5.30 (s, 1H), 0.99 (app d, *J* = 7.6 Hz, 18H), 0.25 (app s, 12H) ppm.

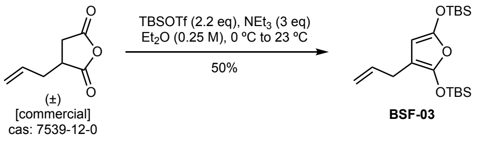

Further Purification: Flash column chromatography using phosphate buffered silica (pH = 7, ratio of buffered silica to theoretical crude mass = 10:1, eluting with hexane).

For characterization data of a further purified sample, see our previous report.[16]

Yield: 0.664 g, 1.80 mmol, 50%;

Appearance: Colorless oil;

R*f*: 0.44 (petroleum ether);

^{1}H NMR (400 MHz, $CDCl_3$): δ 5.91 – 5.79 (m, 1H), 5.04 (d, *J* = 16.3 Hz, 1H), 4.97 (d, *J* = 10.0 Hz, 1H), 4.83 (s, 1H), 2.92 (d, *J* = 6.3 Hz, 2H), 0.95 (app d, *J* = 6.4 Hz, 18H), 0.19 (app d, *J* = 5.4 Hz, 12H) ppm.

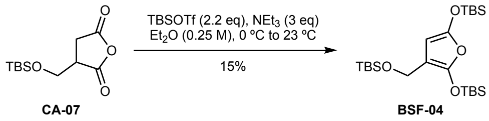

Further Purification: Flash column chromatography using phosphate buffered silica (pH = 7, ratio of buffered silica to theoretical crude mass = 10:1, eluting with hexane).

Yield: 0.152 g, 0.321 mmol, 15%;

Appearance: White solid;

R*f*: 0.29 (5:95 Hexane:EtOAc);

^{1}H NMR (600 MHz, $CDCl_3$): δ 4.99 (s, 1H), 4.32 (s, 2H), 0.96 (app d, *J* = 8.7 Hz, 18H), 0.88 (s, 9H), 0.20 (app s, 12H), 0.05 (s, 6H) ppm;

^{13}C NMR (150 MHz, $CDCl_3$): δ 147.2, 143.6, 98.3, 85.2, 56.3, 26.1, 25.7, 25.6, 18.5, 18.3, 18.1, –4.5, –4.7, –5.0 ppm;

IR: 2933, 2892, 2860, 1660, 1631, 1467, 1405, 1256, 1060, 893, 840, 785 cm^{-1};

HRMS (ESI): calculated for $[C_{23}H_{48}O_4Si_3+Na]^+$: 495.2753, found: 495.2753.

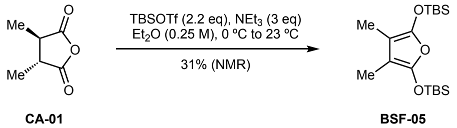

Crude furan submitted to the next reaction without further purification. For characterization purposes, a small amount was purified by flash column chromatography using phosphate buffered silica (pH = 7, ratio of buffered silica to theoretical crude mass = 10:1, eluting with hexane).

Crude Yield: 0.483 mmol, 31% (NMR yield, determined using CH_2Br_2 as internal standard);

Appearance: Colorless oil;

R*f*: N/A (decomposes on glass-backed silica TLC plates);

^{1}H NMR (400 MHz, $CDCl_3$): δ 1.70 (s, 6H), 0.96 (s, 18H), 0.18 (s, 12H) ppm;

^{13}C NMR (100 MHz, $CDCl_3$): δ 142.8, 93.7, 25.7, 18.2, 7.4, –4.5 ppm;

IR: 2932, 2867, 2851, 1722, 1713, 1658, 1468, 1408, 1385, 1363, 1308, 1255 cm^{-1};

HRMS (ESI): calculated for $[C_{13}H_{36}O_3Si_2+H]^+$: 357.2281, found: 357.2278.

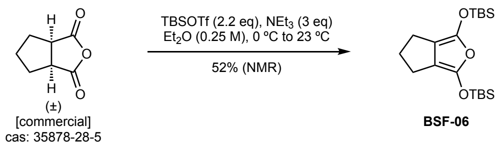

Crude furan submitted to the next reaction without further purification.

For characterization data of a further purified sample, see our previous report.[16]

Crude Yield: 1.84 mmol, 52% (NMR yield, determined using durene as internal standard);

Appearance: Pale yellow oil;

R*f*: N/A (decomposes on glass-backed silica TLC plates);

^{1}H NMR (400 MHz, $CDCl_3$): δ 2.37 (t, J = 7.0 Hz, 4H) 2.17 – 2.19 (m, 2H), 0.95 (s, 18H), 0.18 (s, 12H) ppm.

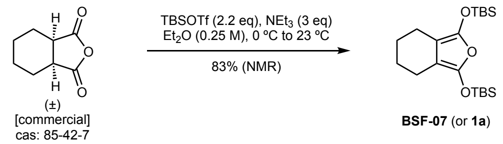

Crude furan submitted to the next reaction without further purification.

For characterization data of a further purified sample, see our previous report.[16]

Yield: 5.40 mmol, 83% (NMR yield, determined using CH_2Br_2 as internal standard);

Appearance: Pale yellow oil;

R*f*: N/A (decomposes on glass-backed silica TLC plates);

^{1}H NMR (400 MHz, $CDCl_3$): δ 2.27 (m, 4H), 1.57 (m, 4H), 0.96 (s, 18H), 0.18 (s, 12H) ppm.

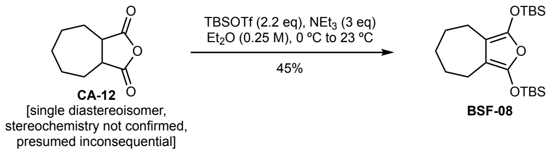

Further Purification: Flash column chromatography using phosphate buffered silica (pH = 7, ratio of buffered silica to theoretical crude mass = 10:1, eluting with hexane).

Yield: 87.3 mg, 0.220 mmol, 45%;

Appearance: Colorless oil;

R*f*: N/A (decomposes on glass-backed silica TLC plates);

^{1}H NMR (600 MHz, $CDCl_3$): δ 2.30 – 2.27 (m, 4H), 1.70 – 1.65 (m, 2H), 1.57 – 1.53 (m, 4H), 0.96 (s, 18H), 0.17 (s, 12H) ppm;

^{13}C NMR (150 MHz, $CDCl_3$): δ 141.5, 100.8, 33.1, 30.4, 25.7, 25.1, 18.2, –4.5 ppm;

IR: 2929, 2859, 1721, 1666, 1643, 1471, 1447, 1391, 1358, 1289, 1253, 1117, 1003, 942, 855, 785 cm^{-1};

HRMS (ESI): calculated for $[C_{21}H_{40}O_3Si_2+H]^+$: 397. 2594, found: 397. 2590.

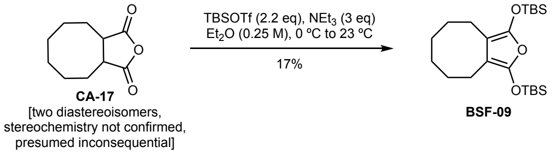

Further Purification: Filtration over phosphate buffered silica (pH = 7, ratio of buffered silica to theoretical crude mass = 5:1, eluting with hexane).

Yield: 0.267 g, 0.650 mmol, 17%;

Appearance: Colorless oil;

R*f*: 0.46 (1:9 Hexane:EtOAc);

^{1}H NMR (600 MHz, $CDCl_3$): δ 2.29 (t, 4H), 1.55 – 1.43 (m, 8H), 0.97 (s, 18H), 0.18 (s, 12H) ppm;

^{13}C NMR (100 MHz, $CDCl_3$): δ 142.0, 98.6, 30.3, 25.8, 25.7, 21.1, 18.2, –4.5 ppm;

IR: 2930, 2859, 1672, 1656, 1649, 1462, 1412, 1364, 1296, 1254 cm^{-1};

HRMS (ESI): calculated for $[C_{22}H_{42}O_3Si_2+H]^+$: 411.2745, found: 411.2741.

Synthesis of Ketenimines

Ketenimine Precursors

Anilines

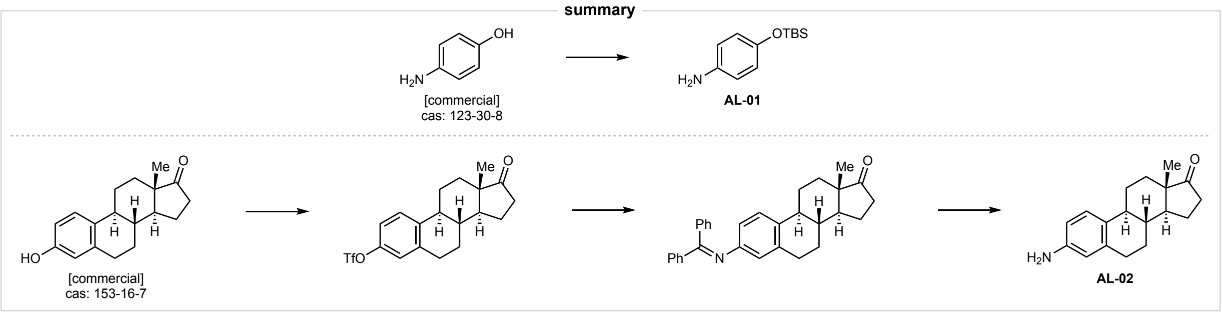

AL-01 was prepared in one step according the procedure reported by Sakakura and Ishihara.[29] Characterization data matched those reported by Crews.[30]

AL-02 was prepared in three steps according the procedure reported by Lynam and Fairlamb.[31] Characterization data matched those reported by Lynam and Fairlamb.[31]

Amides

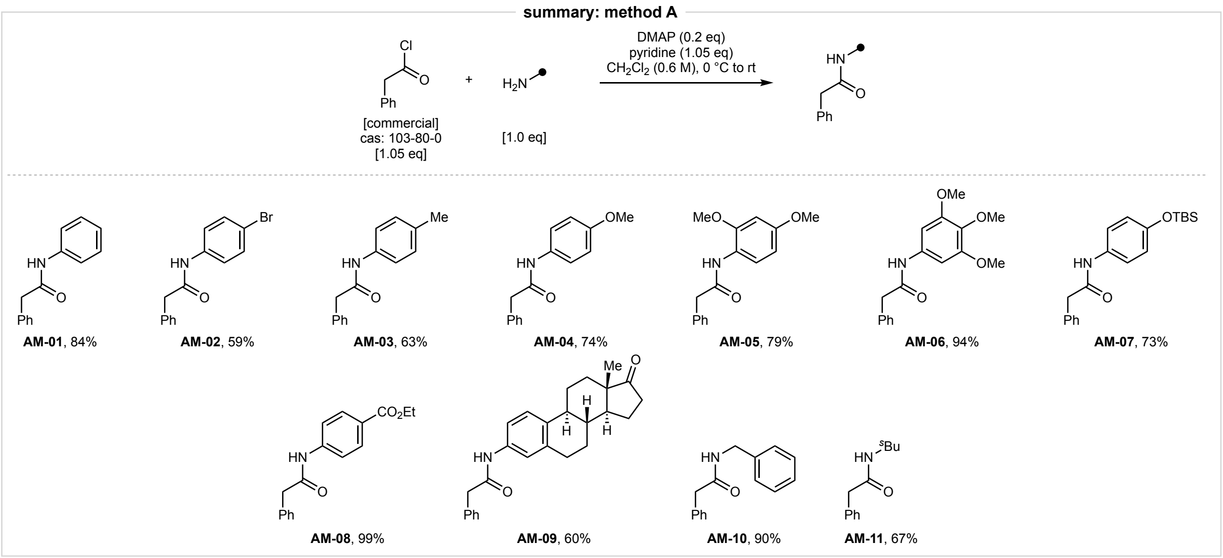

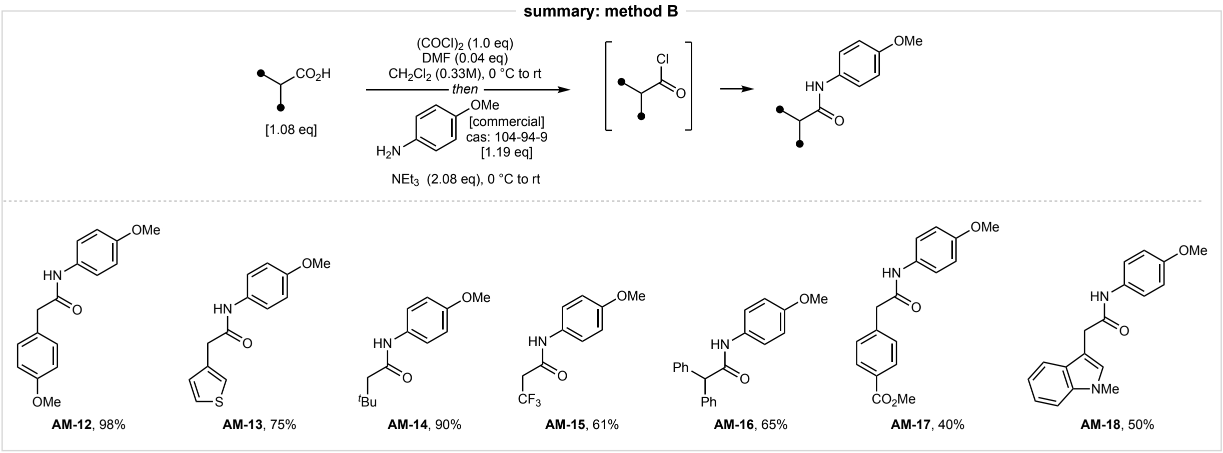

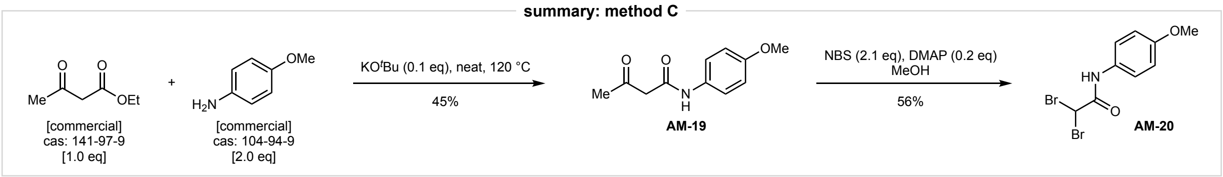

General Procedure for Method A

To a flame-dried three-neck round-bottom flask under argon and equipped with a stir bar was added the appropriate amine (1.00 equiv), anhydrous CH_2Cl_2 (0.60 M), 4-dimethylaminopyridine (0.200 equiv), and anhydrous pyridine (1.05 equiv). The reaction flask was submerged in an ice/water bath, and 2-phenylacetylchloride (1.05 equiv) was added dropwise. The reaction was allowed to warm to ambient temperature and left to stir overnight. The crude

mixture was diluted with water and CH_2Cl_2 then quenched with 2N HCl. The layers were separated, and the aqueous layer was washed with CH_2Cl_2. The combined organics were washed with saturated aqueous sodium $NaHCO_3$ and brine, dried over $MgSO_4$, filtered, and concentrated under reduced pressure. Further purification details (if employed) are provided alongside the characterization data for each amide. Yields are calculated with respect to moles of amine employed.

General Procedure for Method B

Adapted from the procedure reported by Zhang and Tu.[32] To a flame-dried three-neck round-bottom flask under argon and equipped with a stir bar was added the appropriate carboxylic acid (1.08 equiv) and anhydrous CH_2Cl_2 (0.33 M). The reaction flask was submerged in an ice/water bath, and oxalyl chloride (1.00 equiv) was added dropwise, followed by anhydrous DMF (0.0200 equiv, bubbling observed). The reaction was allowed to warm to ambient temperature and stirred for 2 hours, then additional anhydrous DMF (0.0200 equiv, bubbling observed) was added. The reaction was stirred for an additional 2 hours. The reaction flask was submerged in an ice/water bath and 4-methoxyaniline (1.19 equiv) was added, followed by triethylamine (2.08 equiv). The reaction was allowed to warm to ambient temperature and left to stir overnight. The crude mixture was diluted with water and CH_2Cl_2 then quenched with 2N HCl. The layers were separated, and the aqueous layer was washed with CH_2Cl_2. The combined organics were washed with saturated aqueous $NaHCO_3$ and brine, dried over $MgSO_4$, filtered, and concentrated under reduced pressure. Further purification details (if employed) are provided alongside the characterization data for each amide. Yields are calculated with respect to moles of oxalyl chloride employed.

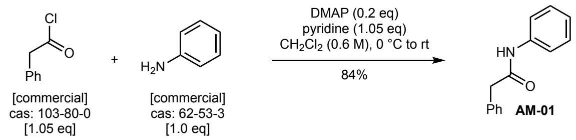

General Method A.

Further Purification: Crude reaction mixture dissolved in a minimum volume of boiling EtOH, cooled to ambient temperature, and transferred to a freezer (−20 °C) to sit overnight, allowing crystals to form. Filtration (washing with cold EtOH) yielded **AM-01**. The filtrate was concentrated under reduced pressure, and the crystallization process was repeated to obtain a second crop of **AM-01**.

Characterization data matched those reported by Nguyen.[33]

Yield: 47.8 g, 226 mmol, 84%;

Appearance: White solid;

R*f*: 0.33 (1:3 EtOAc:hexane);

^{1}H NMR (400 MHz, $CDCl_3$): δ 7.41 (t, J = 8.6 Hz, 4H), 7.34 (d, J = 7.7 Hz, 3H), 7.28 (t, J = 7.7 Hz, 2H), 7.17 (br s, 1H), 7.09 (t, J = 7.5 Hz, 1H), 3.74 (s, 2H) ppm.

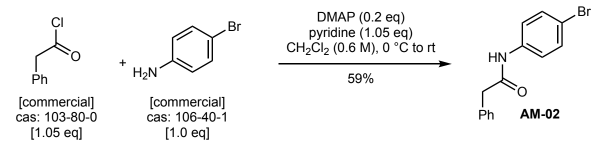

General Method A.

Further Purification: Crude reaction mixture dissolved in a minimum volume of boiling EtOH, cooled to ambient temperature, and transferred to a freezer (−20 °C) to sit overnight, allowing crystals to form. Filtration (washing with cold EtOH) yielded **AM-02**.

Characterization data matched those reported by Snape.[34]

Yield: 4.99 g, 17.2 mmol, 59%;

Appearance: Fluffy white solid;

R*f*: 0.54 (1:1 EtOAc:hexane);

^{1}H NMR (400 MHz, $CDCl_3$): δ 7.45 – 7.28 (m, 9H), 7.10 (br s, 1H), 3.73 (s, 2H) ppm.

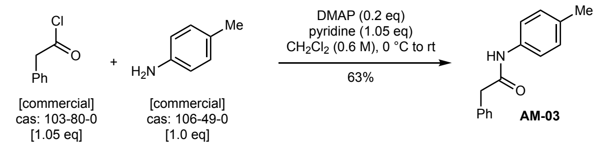

General Method A.

Further Purification: Crude reaction mixture dissolved in a minimum volume of boiling EtOH, and hexane was slowly added until a small amount of solid began to crash out of solution. The flask was cooled to ambient temperature and transferred to a freezer (−20 °C) to sit overnight, allowing further solid to crash out of solution. Filtration (washing with cold hexane) yielded **AM-03**.

Characterization data matched those reported by Ma.[35]

Yield: 1.33 g, 5.90 mmol, 63%;

Appearance: Pale yellow solid;

R*f*: 0.47 (1:1 EtOAc:hexane);

^{1}H NMR (400 MHz, $CDCl_3$): δ 7.39 (m, 2H), 7.37 – 7.27 (m, 5H), 7.08 (d, J = 7.9 Hz, 3H), 3.72 (s, 2H), 2.29 (s, 3H) ppm.

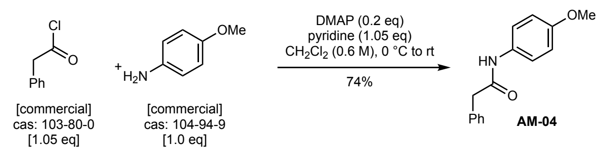

General Method A.

Further Purification: Crude reaction mixture dissolved in a minimum volume of boiling EtOH, cooled to ambient temperature, and transferred to a freezer (−20 °C) to sit overnight, allowing crystals to form. Filtration (washing with cold EtOH) yielded **AM-04**. The filtrate was concentrated under reduced pressure, and the crystallization process was repeated to obtain a second crop of **AM-04**.

Characterization data matched those reported by Joseph.[36]

Yield: 7.24 g, 30.0 mmol, 74%;

Appearance: White solid;

R*f*: 0.22 (1:3 EtOAc:hexane);

^{1}H NMR (400 MHz, $CDCl_3$): δ 7.39 (app t, *J* = 6.4 Hz, 2H), 7.36 – 7.28 (m, 5H), 7.00 (br s, 1H), 6.81 (d, *J* = 9.0 Hz, 2H), 3.77 (s, 3H), 3.72 (s, 2H) ppm.

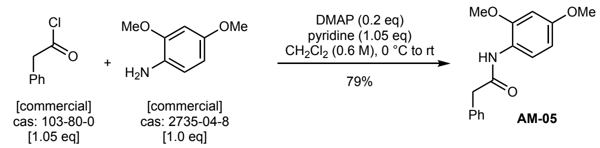

General Method A.

Crude amide submitted to the next reaction without further purification.

Yield: 0.140 g, 0.516 mmol, 79%;

Appearance: Gray solid;

R*f*: 0.40 (1:1 EtOAc:hexane);

^{1}H NMR (400 MHz, $CDCl_3$): δ 8.21 (d, *J* = 8.8 Hz, 1H), 7.56 (br s, 1H), 7.43 – 7.30 (m, 5H), 6.45 (d, *J* = 9.1 Hz, 1H), 6.40 (s, 1H), 3.77 (s, 3H), 3.74 (s, 2H), 3.70 (s, 3H) ppm;

^{13}C NMR (100 MHz, $CDCl_3$): δ 168.7, 156.5, 149.4, 134.9, 129.7, 129.1, 127.5, 121.3, 120.5, 103.8, 98.7, 55.8, 55.6, 45.1 ppm;

IR: 3389, 2944, 2836, 1678, 1665, 1603, 1547, 1530, 1494, 1462, 1453, 1440, 1414, 1300, 1209 cm^{-1};

HRMS (ESI): calculated for $[C_{16}H_{17}NO_3+H]^+$: 272.1284, found: 272.1281.

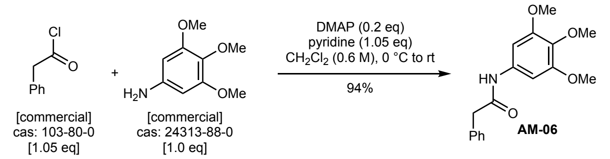

General Method A.

Crude amide submitted to the next reaction without further purification.

Yield: 7.76 g, 25.8 mmol, 94%;

Appearance: Fluffy white solid;

R*f*: 0.25 (1:1 EtOAc:hexane);

^{1}H NMR (400 MHz, $CDCl_3$): δ 7.39 (app t, *J* = 7.3 Hz, 2H), 7.37 – 7.29 (m, 3H), 7.12 (br s, 1H), 6.73 (s, 2H), 3.83 – 3.75 (m, 9H), 3.72 (s, 2H);

^{13}C NMR (100 MHz, $CDCl_3$): δ 169.2, 153.4, 134.9, 134.5, 133.9, 129.6, 129.4, 127.8, 97.7, 61.1, 56.3, 45.0 ppm;

IR: 3296, 1659, 1598, 1451, 1412, 1308, 1232, 1134, 726 cm^{-1};

HRMS (ESI): calculated for $[C_{17}H_{19}NO_4+H]^+$: 302.1391, found: 302.1387.

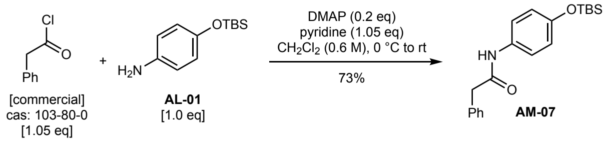

General Method A.

Further Purification:

Crude reaction mixture triturated with hexane to yield **AM-07**.

Yield: 3.39 g, 9.77 mmol, 73%;

Appearance: White solid;

R*f*: 0.43 (1:2 EtOAc:hexane);

^{1}H NMR (600 MHz, $CDCl_3$): δ 7.39 (t, *J* = 6.9 Hz, 2H), 7.33 (app d, *J* = 7.2 Hz, 3H), 7.26 – 7.23 (m, 2H), 6.94 (br d, 1H), 6.75 (d, *J* = 8.8 Hz, 2H), 3.72 (s, 2H), 0.96 (s, 9H), 0.16 (s, 6H) ppm;

^{13}C NMR (150 MHz, $CDCl_3$): δ 169.0, 152.6, 134.8, 131.5, 129.7, 129.3, 127.7, 121.7, 120.4, 44.9, 25.8, 18.3, –4.3 ppm;

IR: 3295, 3196, 3136, 3063, 2957, 2857, 1658, 1612, 1572, 1468, 1353, 1270, 1249, 1144, 917, 841 cm^{-1};

HRMS (ESI): calculated for $[C_{20}H_{27}NO_2Si+H]^+$: 342.1889, found: 342.1880.

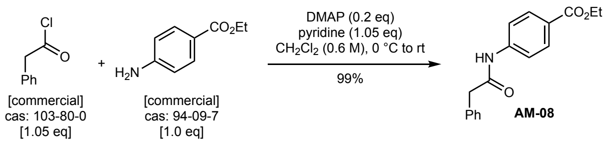

General Method A.

Crude amide submitted to the next reaction without further purification.

Yield: 3.40 g, 12.0 mmol, 99%;

Appearance: White solid;

R*f*: 0.43 (1:1 EtOAc:hexane);

^{1}H NMR (400 MHz, $CDCl_3$): δ 7.96 (d, *J* = 8.7 Hz, 2H), 7.49 (d, *J* = 8.3 Hz, 2H), 7.41 (t, *J* = 7.3 Hz, 2H), 7.35 (t, *J* = 9.1 Hz, 3H), 7.22 (br s, 1H), 4.34 (q, *J* = 7.0 Hz, 2H), 3.77 (s, 2H), 1.38 (t, *J* = 7.1 Hz, 3H) ppm;

^{13}C NMR (100 MHz, $CDCl_3$): δ 169.3, 166.2, 141.8, 134.1, 130.9, 129.7, 129.5, 128.1, 126.3, 118.9, 61.0 45.1, 14.5 ppm;

IR: 3251, 3187, 3118, 3062, 2986, 1704, 1672, 1598, 1536, 1484, 1407, 1365, 1282, 1173, 1128, 1026, 966, 862, 834, 769, 716 cm^{-1};

HRMS (ESI): calculated for $[C_{17}H_{17}NO_4+H]^+$: 284.1279, found: 284.1281.

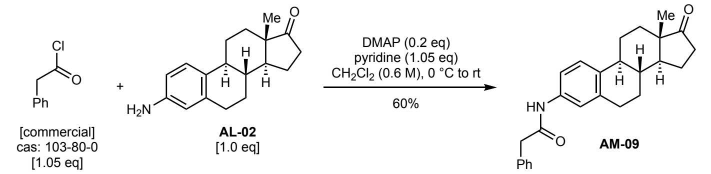

General Method A.

Further Purification: Crude reaction mixture triturated with EtOAc/hexane (1:1) to yield **AM-09**. The filtrate was concentrated under reduced pressure, and the trituration process was repeated to obtain a second crop of **AM-09**.

Yield: 294 mg, 0.759 mmol, 60%;

Appearance: White solid;

R*f*: 0.17 (1:2 EtOAc:hexane);

^{1}H NMR (900 MHz, $CDCl_3$): δ 7.40 (t, *J* = 7.4 Hz, 2H), 7.33 (t, *J* = 7.8 Hz, 1H), 7.23 (s, 1H), 7.19 (d, *J* = 8.5 Hz, 1H), 7.10 (d, *J* = 7.5 Hz, 1H), 6.97 (br s, 1H), 3.73 (s, 2H), 2.86 (dd, *J* = 9.1, 4.2 Hz, 2H), 2.50 (dd, *J* = 8.9, 1H), 2.37 (d, *J* = 11.8 Hz, 1H), 2.24 (t, *J* = 11.2 Hz, 1H), 2.13 (p, *J* = 9.1, 1H), 2.07 – 2.02 (m, 1H), 2.01 – 1.96 (m, 1H), 1.94 (d, *J* = 10.5 Hz, 1H), 1.61 (p, *J* = 10.7 Hz, 1H), 1.54 (d, *J* = 9.2 Hz, 1H), 1.52 – 1.44 (m, 3H), 1.41 (p, *J* = 10.3 Hz, 1H), 0.89 (s, 3H) ppm;

^{13}C NMR (225 MHz, $CDCl_3$): δ 221.1, 169.3, 137.5, 136.3, 135.6, 134.8, 129.7, 129.4, 127.8, 126.0, 120.5, 117.7, 50.6, 48.2, 45.0, 44.4, 38.4, 36.1, 31.8, 29.7, 26.6, 26.0, 21.8, 14.1 ppm;

IR: 3315, 3062, 3030, 2931, 2861, 2247, 1737, 1726, 1678, 1659, 1598, 1502, 1494, 1433, 1343, 1256, 1008, 911, 733 cm^{-1};

HRMS (ESI): calculated for $[C_{26}H_{29}NO_2+H]^+$: 388.2277, found: 388.2270;

$[\alpha]_D^{20}$: +112.4 (c = 0.77, CH_2Cl_2).

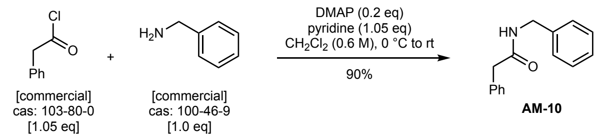

General Method A.

Further Purification: Crude reaction mixture dissolved in a minimum volume of boiling EtOH, cooled to ambient temperature, and transferred to a freezer (−20 °C) to sit overnight, allowing crystals to form. Filtration (washing with cold EtOH) yielded **AM-10**. The filtrate was concentrated under reduced pressure, and the crystallization process was repeated to obtain a second crop of **AM-10**.

Characterization data matched those reported by Adolfsson.[37]

Yield: 2.00 g, 9.00 mmol, 90%;

Appearance: White solid;

R*f*: 0.33 (1:1 EtOAc:hexane);

^{1}H NMR (600 MHz, $CDCl_3$): δ 7.36 (t, *J* = 7.3 Hz, 2H), 7.33 – 7.24 (m, 6H), 7.19 (d, *J* = 7.3 Hz, 2H), 5.71 (s, 1H), 4.43 (d, *J* = 5.9 Hz, 2H), 3.64 (s, 2H) ppm.

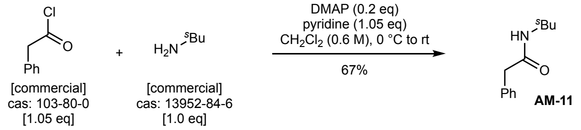

General Method A.

Further Purification: Crude reaction mixture triturated with hexane to yield **AM-11**. The filtrate was concentrated under reduced pressure, and the trituration process was repeated to obtain a second crop of **AM-011**.

Characterization data matched those reported by Huan-Feng Jiang.[38]

Yield: 3.52 g, 18.4 mmol, 67%;

Appearance: Pale orange solid;

R*f*: 0.38 (3:7 EtOAc:hexane);

^{1}H NMR (400 MHz, $CDCl_3$) δ 7.39 – 7.22 (m, 5H), 5.13 (br s, 1H), 3.93 – 3.86 (m, 1H), 3.55 (s, 2H), 1.31–1.46 (m, 2H), 1.03 (d, *J* = 6.6 Hz, 3H), 0.82 – 0.78 (m, 3H) ppm.

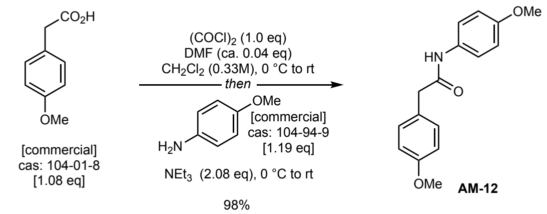

General Method B.

Further Purification: Crude reaction mixture dissolved in a minimum volume of boiling EtOH, cooled to ambient temperature, and transferred to a freezer (−20 °C) to sit overnight, allowing crystals to form. Filtration (washing with cold EtOH) yielded **AM-12**.

Characterization data matched those reported by Hwang.[39]

Yield: 2.20 g, 8.12 mmol, 98%;

Appearance: White solid;

R*f*: 0.33 (1:1 EtOAc:hexane);

^{1}H NMR (400 MHz, $CDCl_3$): δ 7.30 (d, *J* = 8.8 Hz, 2H), 7.24 (d, 2H), 6.93 (d, *J* = 8.2 Hz, 3Hfz), 6.81 (d, *J* = 8.7 Hz, 2H), 3.83 (s, 3H), 3.77 (s, 3H), 3.67 (s, 2H) ppm.

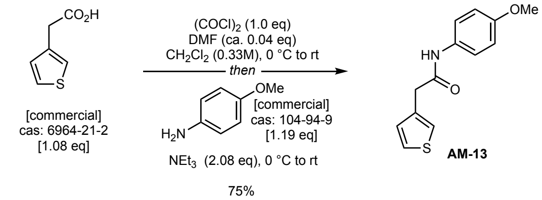

General Method B.

Further Purification: Crude reaction mixture was filtered through a silica plug, sequentially eluting with hexane, CH_2Cl_2, and EtOAc. The EtOAc wash was concentrated under reduced pressure to yield **AM-13**.

Characterization data matched those reported by Hwang.[39]

Yield: 1.46 g, 5.92 mmol, 75%;

Appearance: Brown solid;

R*f*: 0.33 (1:3 EtOAc:hexane);

^{1}H NMR (400 MHz, $CDCl_3$): δ 7.38 (br s, 1H), 7.32 (d, *J* = 8.4 Hz, 2H), 7.21 (m, 2H), 7.07 (d, *J* = 4.9 Hz, 1H), 6.82 (d, *J* = 8.4 Hz, 2H), 3.75 (app d, *J* = 12.2 Hz, 5H) ppm.

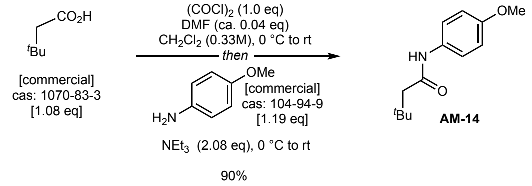

General Method B.

Crude amide submitted to the next reaction without further purification.

While characterization data matched those reported by Cheung and Ma,[40] the amide carbonyl signal was not observable in their ^{13}C NMR spectrum (resolution too low), so we include our ^{13}C NMR data here.

Yield: 3.15 g, 14.2 mmol, 90%;

Appearance: Brown solid;

R*f*: 0.47 (1:1 EtOAc:hexane);

^{1}H NMR (400 MHz, $CDCl_3$): δ 7.40 (d, *J* = 8.4 Hz, 2H), 7.01 (br s, 1H), 6.85 (d, *J* = 8.4 Hz, 2H), 3.78 (s, 3H), 2.20 (s, 2H), 1.10 (s, 9H) ppm;

^{13}C NMR (100 MHz, $CDCl_3$): δ 170.3, 156.5, 131.2, 122.2, 114.2, 55.6, 51.4, 31.4, 30.0 ppm.

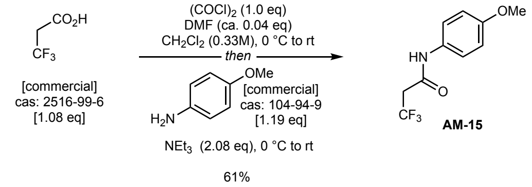

General Method B.

Further Purification: Crude reaction mixture dissolved in a minimum volume of boiling EtOH, cooled to ambient temperature, and transferred to a freezer (−20 °C) to sit overnight, allowing crystals to form. Filtration (washing with cold EtOH) yielded **AM-15**.

While ^{1}H NMR data matched those reported by Zhang and Tu,[32] neither ^{13}C NMR nor ^{19}F NMR data have been reported, so we include both here.

Yield: 3.35 g, 14.4 mmol, 61%;

Appearance: Gray solid;

R*f*: 0.40 (1:1 EtOAc:hexane);

^{1}H NMR [400 MHz, $(CD_3)_2SO$]: δ 10.15 (s, 1H), 7.47 (d, *J* = 8.6 Hz, 2H), 6.90 (d, *J* = 8.6 Hz, 2H), 3.72 (s, 3H), 3.45 (q, *J* = 11.2 Hz, 2H) ppm;

^{13}C NMR [100 MHz, $(CD_3)_2SO$]: δ 161.4 (q, *J* = 3.8 Hz), 156.1, 132.0, 125.3 (q, *J* = 276.3 Hz), 121.3, 114.5, 55.6, 40.9 (q, *J* = 27.7 Hz) ppm;

^{19}F NMR [377 MHz, $(CD_3)_2SO$]: δ −61.35 (t, *J* = 11.2 Hz) ppm;

IR: 3297, 3081, 2841, 1650, 1602, 1548, 1514, 1372, 1292, 1249, 1150, 1084, 1029, 834 cm^{-1};

HRMS (ESI): calculated for $[C_{10}H_{10}F_3NO_2+H]^+$: 234.0736, found: 234.0734.

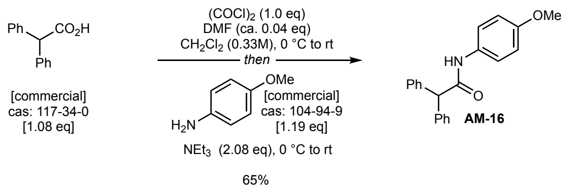

General Method B.

Further Purification: Crude reaction mixture dissolved in a minimum volume of boiling EtOH, cooled to ambient temperature, and transferred to a freezer (−20 °C) to sit overnight, allowing crystals to form. Filtration (washing with cold EtOH) yielded **AM-16**.

While ^{1}H NMR data matched those reported Liu,[41] we were unable to find additional characterization data, so we include it here.

Yield: 1.797 g, 5.66 mmol, 65%;

Appearance: White solid;

R*f*: 0.50 (1:1 EtOAc:hexane);

^{1}H NMR (400 MHz, $CDCl_3$): δ 7.40 – 7.28 (m, 12H), 7.16 (br s, 1H), 6.83 (d, *J* = 8.5 Hz, 2H), 5.07 (s, 1H), 3.78 (s, 3H) ppm;

^{13}C NMR (100 MHz, $CDCl_3$): δ 170.0, 156.7, 139.4, 130.9, 129.1, 129.0, 127.6, 121.8, 114.2, 60.1, 55.6 ppm;

IR: 3285, 2998, 2917, 2428, 1765, 1658, 1649, 1511, 1307, 1245, 1054 cm^{-1};

HRMS (ESI): calculated for $[C_{21}H_{19}NO_2+H]^+$: 318.1489, found: 318.1491.

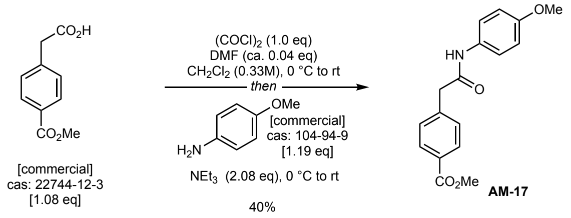

General Method B.

Further Purification: Crude reaction mixture dissolved in a minimum volume of boiling EtOH, cooled to ambient temperature, and transferred to a freezer (−20 °C) to sit overnight, allowing crystals to form. Filtration (washing with cold EtOH) yielded **AM-17**. The filtrate was concentrated under reduced pressure, and the crystallization process was repeated to obtain a second crop of **AM-17**.

Yield: 227 mg, 0.758 mmol, 40%;

Appearance: Pale purple solid;

R*f*: 0.17 (3:7 EtOAc:hexane);

^{1}H NMR (600 MHz, $CDCl_3$): δ 8.04 (d, *J* = 8.1 Hz, 2H), 7.41 (d, *J* = 8.1 Hz, 2H), 7.32 (d, *J* = 8.7 Hz, 2H), 7.11 (br s, 1H), 6.81 (d, *J* = 8.8 Hz, 2H), 3.92 (s, 3H), 3.79 – 3.74 (m, 5H) ppm;

^{13}C NMR (225 MHz, $CDCl_3$): δ 168.3, 166.9, 156.8, 134.0, 130.7, 130.4, 129.6, 129.4, 122.1, 114.2, 55.6, 52.3, 44.5 ppm;

IR: 3279, 2905, 1721, 1658, 1597, 1530, 1513, 1432, 1410, 1278, 872 cm^{-1};

HRMS (ESI): calculated for $[C_{17}H_{17}NO_4+H]^+$: 300.1236, found: 300.1230.

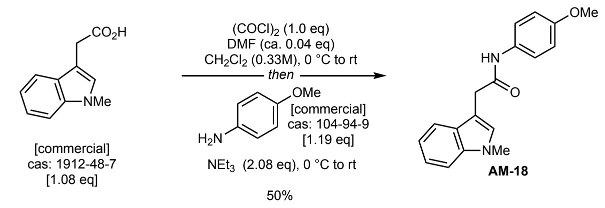

General Method B.

Further Purification: Crude reaction mixture was filtered through a silica plug, sequentially eluting with hexane/CH_2Cl_2 (1:1) and EtOAc. The EtOAc wash was concentrated under reduced pressure and triturated with EtOAc/hexane (1:5) to yield **AM-18**.

Yield: 325 mg, 1.10 mmol, 50%;

Appearance: White solid;

R*f*: 0.33 (1:1 EtOAc:hexane);

^{1}H NMR (900 MHz, $CDCl_3$): δ 7.61 (d, *J* = 7.9 Hz, 1H), 7.37 (d, *J* = 8.2 Hz, 1H), 7.35 (br s, 1H), 7.30 (t, *J* = 7.6 Hz, 1H), 7.24 (d, *J* = 8.9 Hz, 2H), 7.18 (t, *J* = 7.5 Hz, 1H), 7.07 (s, 1H), 6.78 (d, *J* = 9.0 Hz, 2H), 3.86 (s, 2H), 3.82 (s, 3H), 3.75 (s, 3H) ppm;

^{13}C NMR (225 MHz, $CDCl_3$): δ 169.8, 156.5, 137.4, 130.9, 128.7, 127.5, 122.5, 122.0, 120.0, 118.9, 114.1, 109.8, 107.2, 55.6, 34.3, 33.0 ppm;

IR: 3266, 3133, 3054, 2936, 2836, 1658, 1642, 1604, 1530, 1412, 1296, 1242, 1172 cm^{-1};

HRMS (ESI): calculated for $[C_{18}H_{18}N_2O_2+H]^+$: 295.1447, found: 295.1444.

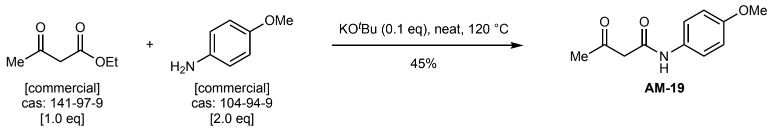

Prepared according to a modification of the procedure reported by Lu, Liu, and Luo.[42] To a flame-dried 8 mL microwave vessel under argon and equipped with a stir bar was added ethyl acetoacetate (3.00 g, 24.4 mmol, 1.00 equiv), 4-methoxyaniline (6.34 g, 48.7 mmol, 2.00 equiv, purified via crystallization from aqueous EtOH), and KO^{t}Bu (273 mg, 2.44 mmol, 0.100 equiv). The reaction was heated to 120 ºC in the microwave and stirred for 2 hours. The reaction was cooled to ambient temperature, filtered through a silica plug (eluting with a Et_2O/CH_2Cl_2, 1:9), and concentrated under reduced pressure. Trituration with cold Et_2O yielded **AM-19**.

Characterization data matched those reported by Lu, Liu, and Luo.[42]

Yield: 2.26 g, 10.9 mmol, 45%;

Appearance: White solid;

R*f*: 0.22 (1:9 Et_2O:CH_2Cl_2);

^{1}H NMR (600 MHz, $CDCl_3$): δ 9.03 (br s, 1H), 7.44 (d, *J* = 8.9 Hz, 2H), 6.86 (d, *J* = 8.9 Hz, 2H), 3.79 (s, 3H), 3.58 (s, 2H), 2.33 (s, 3H) ppm.

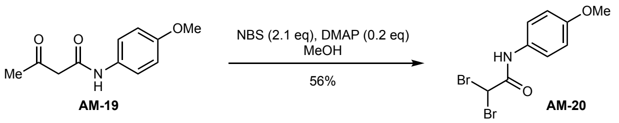

Prepared according to a modification of the procedure reported by Ke and Yeung.[43] To a 500 mL one-neck round-bottom flask open to air and equipped with a stir bar was added **AM-19** (2.46 g, 11.9 mmol, 1.00 equiv), MeOH (238 mL, 0.050 M), NBS (4.44 g, 24.9 mmol, 2.09 equiv, recrystallized from H_2O), and DMAP (0.290 g, 2.38 mmol, 0.200 equiv). The reaction was placed under argon and stirred overnight, quenched with saturated aqueous $Na_2S_2O_3$, and extracted with Et_2O (×3). The combined organics were washed with H_2O (×4) and brine, dried over $MgSO_4$, filtered, and concentrated under reduced pressure. The crude reaction mixture dissolved in a minimum volume of boiling EtOH, cooled to ambient temperature, and transferred to a freezer (−20 °C) to sit overnight, allowing crystals to form. Filtration (washing with cold EtOH) yielded **AM-20**.

Characterization data matched those reported by Ke and Yeung.[43]

Yield: 2.14 g, 6.63 mmol, 56%;

Appearance: Pale yellow solid;

R*f*: 0.22 (1:4 EtOAc:hexane);

^{1}H NMR (600 MHz, $CDCl_3$): δ 8.03 (br s, 1H), 7.45 (d, *J* = 9.0 Hz, 2H), 6.91 (d, *J* = 8.9 Hz, 2H), 5.92 (s, 1H), 3.81 (s, 3H) ppm.

Carbamates

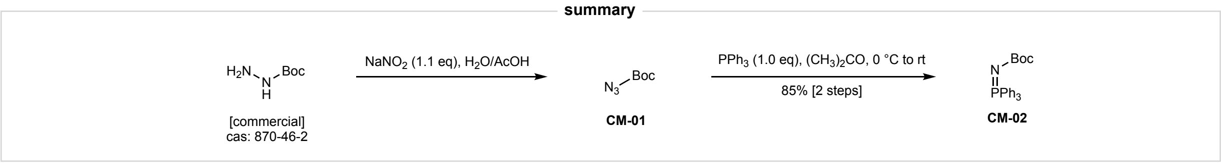

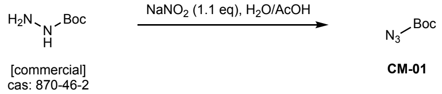

Prepared according to a modification of the procedure reported by Bach.[44] To a 50 mL one-neck round-bottom flask open to air and equipped with a stir bar was added *tert*-butyl carbazate (1.00 g, 7.57 mmol, 1.00 equiv), H_2O (2.34 mL), and AcOH (1.67 mL). The reaction flask was submerged in an ice/water bath, and sodium nitrite (574 mg, 8.32 mmol, 1.01 equiv) was added in portions. The reaction was placed under argon and stirred for 2 hours. The ice/water bath was removed, and the reaction was allowed to warm to ambient temperature and stir for 30 minutes. The mixture was diluted with water and extracted with Et_2O (×3). The combined organics were washed with saturated aqueous $NaHCO_3$, brine, dried over Na_2SO_4, filtered, and concentrated under reduced pressure to yield crude **CM-01** (775 mg) as a yellow oil. The crude mixture was submitted to the next reaction without further purification.

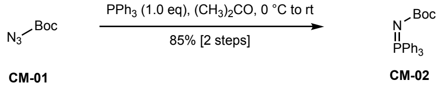

Prepared according to a modification of the procedure reported by Domingues.[45] To a 50 mL one-neck round-bottom flask open to air and equipped with a stir bar was added crude **CM-01** (775 mg, assumed to be 5.41 mmol, 1.00 equiv) and acetone (10.8 mL, 0.5 M). The reaction flask was submerged in an ice/water bath and triphenylphosphine (1.45 g, 5.52 mmol, 1.02 equiv) was added in portions over 15 minutes. The reaction was placed under argon, the ice/water bath was removed, and the reaction was allowed to warm to ambient temperature and stir for 1.5 hours. The reaction was concentrated under reduced pressure, diluted with hexane, and filtered through a plug of silica (ratio of silica to crude mass = 10:1, washing with hexane then EtOAc). The EtOAc wash was concentrated under reduced pressure to yield **CM-02**.

Spectral data matched that reported by Domingues.[45]

Yield: 1.74 g, 4.60 mmol, 85% over 2 steps;

Appearance: White solid;

R*f*: 0.26 (1:1 EtOAc:hexane);

^{1}H NMR (600 MHz, ($CDCl_3$): δ 7.75 – 7.69 (m, 6H), 7.57 – 7.52 (m, 3H), 7.45 (td, J = 7.9, 3.3 Hz, 6H), 1.37 (s, 9H) ppm.

Ketenimines

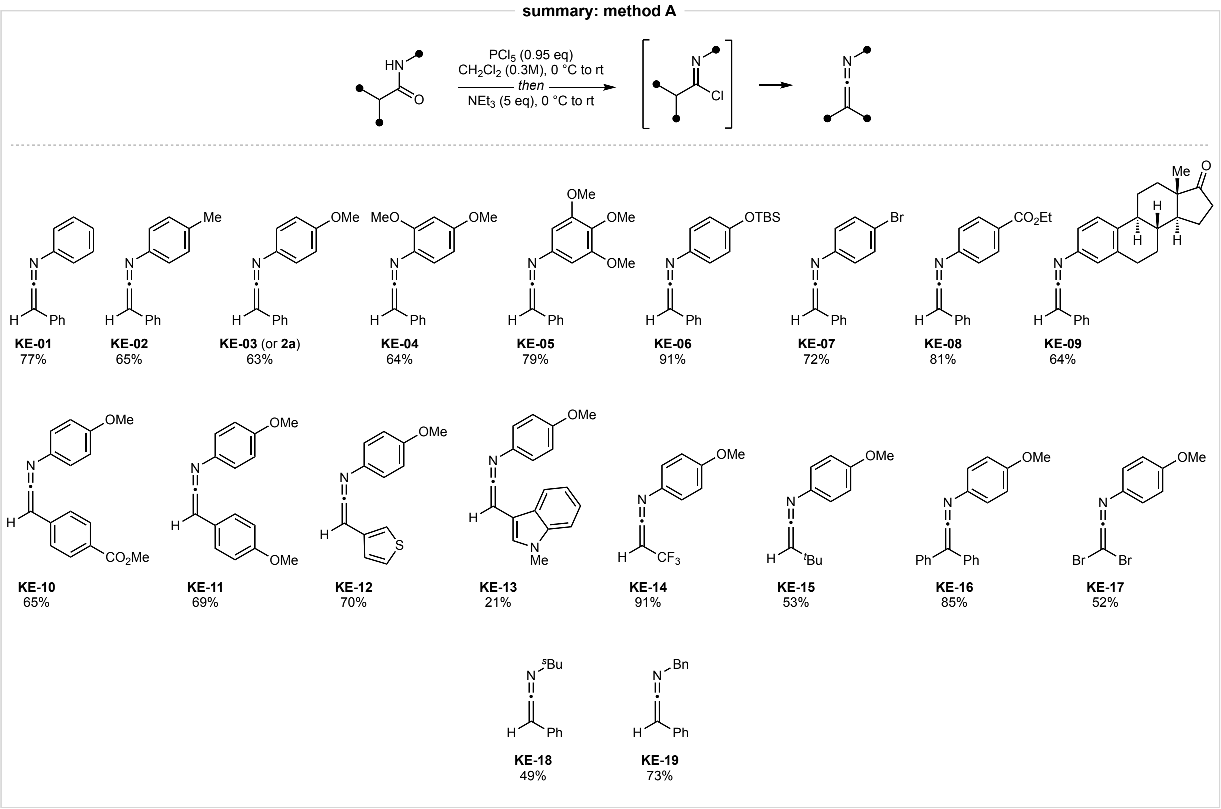

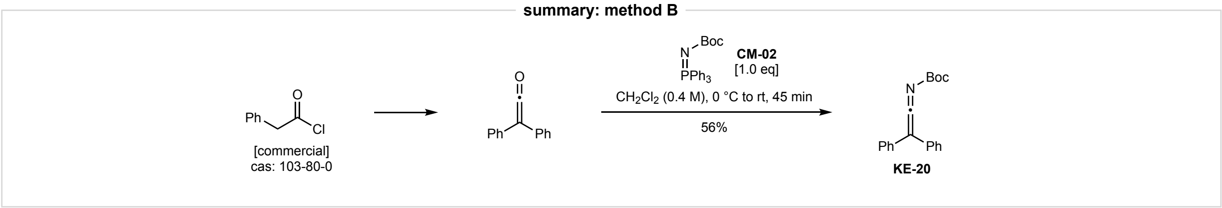

General Procedure for Method A

Adapted from a two-step procedure originally reported by Hegarty.[46] To a flame-dried three-neck round-bottom flask under argon and equipped with a stir bar was added the appropriate amide (1.00 equiv) and anhydrous CH_2Cl_2 (0.30 M). The reaction flask was submerged in an ice/water bath and phosphorous pentachloride (0.950 equiv) was added. The reaction was allowed to warm to ambient temperature and stirred for 1.5 hours. The round-bottom flask was submerged in an ice/water bath and anhydrous triethylamine (5.00 equiv) was added dropwise. The reaction was allowed to warm to ambient temperature, stirred for 30 minutes, and diluted with hexane. The crude mixture was filtered over a Celite plug (eluting with hexane), concentrated under reduced pressure, diluted again with hexane, and filtered over phosphate buffered silica (pH = 7, ratio of buffered silica to crude mass = 5:1, eluting with **solvent mixture A**). The filtrate was concentrated under reduced pressure, diluted with hexane, and filtered over a final Celite

plug (eluting with **solvent mixture B**). The filtrate was concentrated under reduced pressure to afford the corresponding ketenimine. The compositions of **solvent mixture A** and **solvent mixture B** are provided alongside the characterization data for each ketenimine. Yields are calculated with respect to moles of amide employed.

Notes on Reaction Optimization

We found that using a substoichiometric quantity of phosphorous pentachloride (0.950 equiv) aided purification, for unreacted amides were simple to remove from the corresponding ketenimines via filtration, whereas products tentatively assigned as those of over-chlorination proved more challenging to remove.

Notes on Storage

We elected to store the ketenimines neat under argon in the freezer (−80 °C). Based on our stability studies described in the subsequent section, it is assumed warmer temperatures (e.g., −20 °C freezer) would also be suitable.

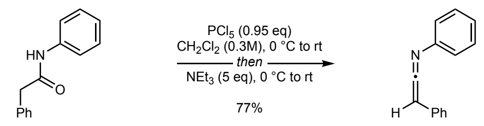

AM-01 KE-01

General Method A.

Purification: Solvent mixture A = 1:4 Et_2O:hexane, solvent mixture B = hexane.

Characterization data matched those reported by Sung.[47]

Yield: 7.0 g, 36.2 mmol, 77%;

Appearance: Yellow oil;

R*f*: N/A (decomposes on glass-backed silica TLC plates);

^{1}H NMR (400 MHz, CD_3CN): δ 7.42 (t, *J* = 7.6 Hz, 2H), 7.38 – 7.25 (m, 5H), 7.19 (d, *J* = 7.8 Hz, 2H), 7.12 (t, *J* = 7.3 Hz, 1H), 5.39 (s, 1H) ppm;

For convenience, we also report ^{1}H NMR data in $CDCl_3$:

^{1}H NMR (400 MHz, $CDCl_3$): δ 7.42 – 7.33 (m, 4H), 7.32 – 7.27 (m, 3H), 7.18 (d, *J* = 7.0 Hz, 2H), 7.11 (t, *J* = 7.3 Hz, 1H), 5.26 (s, 1H) ppm.

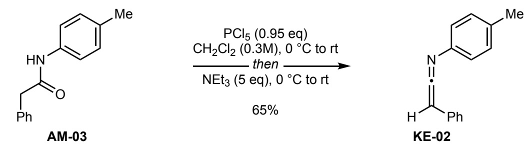

General Method A.

Purification: Solvent mixture A = 1:4 Et_2O:hexane, solvent mixture B = hexane.

Characterization data matched those reported by Sung.[48]

Yield: 299 mg, 1.44 mmol, 65%;

Appearance: Yellow oil;

R*f*: N/A (decomposes on glass-backed silica TLC plates);

^{1}H NMR (400 MHz, $CDCl_3$): δ 7.33 – 7.26 (m, 4H), 7.22 – 7.17 (m, 4H), 7.13 (t, *J* = 7.5 Hz, 1H), 5.26 (s, 1H), 2.39 (s, 3H) ppm.

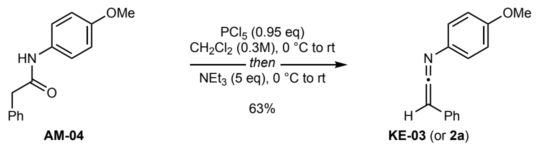

General Method A.

Purification: Solvent mixture A = 1:4 Et_2O:hexane, solvent mixture B = hexane.

Characterization data matched those reported by Sung.[49]

Yield: 589 mg, 2.64 mmol, 63%;

Appearance: Orange oil;

R*f*: N/A (decomposes on glass-backed silica TLC plates);

^{1}H NMR (400 MHz, $CDCl_3$): δ 7.30 (d, *J* = 8.9 Hz, 2H), 7.26 (d, *J* = 7.8 Hz, 2H), 7.16 (d, *J* = 7.2 Hz, 2H), 7.10 (t, *J* = 7.4 Hz, 1H), 6.89 (d, *J* = 8.9 Hz, 2H), 5.22 (s, 1H), 3.82 (s, 3H) ppm.

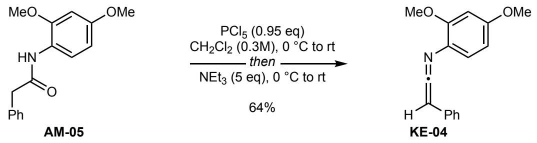

General Method A.

Purification: Solvent mixture A = 1:1 Et_2O:hexane, solvent mixture B = 1:9 Et_2O:hexane.

Yield: 595 mg, 2.35 mmol, 64%;

Appearance: Orange oil;

R*f*: N/A (decomposes on glass-backed silica TLC plates);

^{1}H NMR (900 MHz, $CDCl_3$): δ 7.26 – 7.23 (m, 3H), 7.18 (d, *J* = 7.6 Hz, 2H), 7.07 (t, *J* = 7.4 Hz, 1H), 6.51 (d, *J* = 1.5 Hz, 1H), 6.47 (m, 1H), 5.09 (s, 1H), 3.85 (s, 3H), 3.82 (s, 3H) ppm;

^{13}C NMR (225 MHz, $CDCl_3$): 189.3, 160.3, 154.9, 134.0, 128.8, 126.0, 125.5, 124.6, 121.7, 104.8, 99.6, 58.7, 56.1, 55.7 ppm;

IR: 3026, 2938, 2836, 2009, 1599, 1502, 1461, 1420, 1310, 1286, 1210, 1161, 1032, 834, 760 cm^{-1};

HRMS (ESI): calculated for $[C_{16}H_{15}NO_2+H]^+$: 254.1176, found: 254.1174.

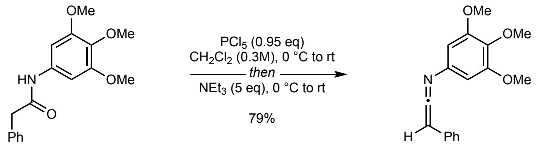

AM-06 **KE-05**

General Method A.

Purification: Solvent mixture A = 1:4 EtOAc:hexane, solvent mixture B = hexane.

Yield: 744 mg, 2.63 mmol, 79%;

Appearance: Orange oil;

R*f*: N/A (decomposes on glass-backed silica TLC plates);

^{1}H NMR (400 MHz, $CDCl_3$): δ 7.28 (t, *J* = 7.7 Hz, 2H), 7.17 (d, *J* = 7.7 Hz, 2H), 7.12 (t, *J* = 7.5 Hz, 1H), 6.61 (s, 2H), 5.27 (s, 1H), 3.85 (s, 9H) ppm;

^{13}C NMR (100 MHz, $CDCl_3$): δ 190.4, 153.8, 137.9, 136.0, 132.6, 129.0, 125.7, 125.5, 101.3, 61.4, 61.1, 56.3 ppm;

IR: 2937, 2835, 2009, 1593, 1502, 1462, 1415, 1328, 1227, 1130, 726 cm^{-1};

HRMS (ESI): calculated for $[C_{17}H_{17}NO_3+H]^+$: 284.1281, found: 284.1270.

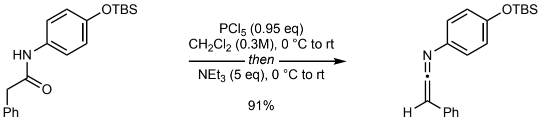

AM-07 **KE-06**

General Method A.

Purification: Solvent mixture A = 1:4 Et_2O:hexane, solvent mixture B = hexane.

Yield: 1.72 g, 5.32 mmol, 91%;

Appearance: Yellow oil;

R*f*: N/A (decomposes on glass-backed silica TLC plates);

^{1}H NMR (600 MHz, CDCl$_3$): δ 7.27 (t, *J* = 7.7 Hz, 2H), 7.24 (d, *J* = 8.7 Hz, 2H), 7.17 (d, *J* = 7.5 Hz, 2H), 7.10 (t, *J* = 7.4 Hz, 1H), 6.83 (d, *J* = 8.7 Hz, 2H), 5.21 (s, 1H), 0.99 (s, 9H), 0.21 (s, 6H) ppm;

^{13}C NMR (150 MHz, CDCl$_3$): δ 189.6, 155.6, 133.6, 133.1, 129.0, 125.6, 125.3, 125.3, 121.0, 60.9, 25.8, 18.4, –4.3 cm^{-1};

IR: 3062, 3029, 2930, 2885, 2858, 2007, 1600. 1580, 1502, 1266, 1210, 1158, 1097, 913, 842, 781, 759 cm^{-1};

HRMS (ESI): calculated for [$C_{20}H_{25}NOSi+H$]$^+$: 324.1784, found: 324.1775.

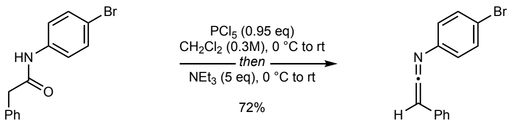

AM-02 KE-07

General Method A.

Purification: Solvent mixture A = 1:4 Et$_2$O:hexane, solvent mixture B = hexane.

Characterization data matched those reported by Sung.[49]

Yield: 1.34 g, 4.92 mmol, 72%;

Appearance: Orange solid;

R*f*: N/A (decomposes on glass-backed silica TLC plates);

^{1}H NMR (400 MHz, CDCl$_3$): δ 7.50 (d, *J* = 8.6 Hz, 2H), 7.29 (t, *J* = 7.7 Hz, 3H), 7.21 (d, *J* = 8.3 Hz, 2H), 7.18 – 7.09 (m, 3H), 5.29 (s, 1H) ppm.

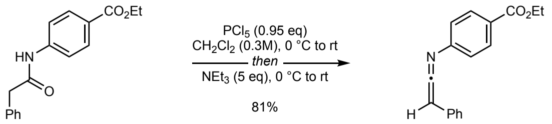

AM-08 KE-08

General Method A.

Purification: Solvent mixture A = 1:4 Et$_2$O:hexane, solvent mixture B = hexane.

Yield: 264 mg, 0.997 mmol, 81%;

Appearance: Orange oil;

R*f*: N/A (decomposes on glass-backed silica TLC plates);

^{1}H NMR (400 MHz, CDCl$_3$): δ 8.07 (d, *J* = 7.8 Hz, 2H), 7.36 (d, *J* = 8.4 Hz, 2H), 7.30 (t, *J* = 7.3 Hz, 2H), 7.23 – 7.10 (m, 3H), 5.33 (s, 1H), 4.39 (q, *J* = 7.1 Hz, 2H), 1.40 (t, *J* = 7.1 Hz, 3H) ppm;

^{13}C NMR (100 MHz, CDCl$_3$): δ 192.8, 165.83, 144.76, 131.80, 131.07, 129.59, 129.04, 125.76, 125.69, 123.46, 61.17, 60.98, 14.36 ppm;

IR: 3207, 2981, 2005, 1722, 1711, 1598, 1494, 1460, 1413, 1391, 1367, 1277, 1167, 1107, 1017, 861 cm^{-1};

HRMS (ESI): calculated for $[C17H_{15}NO_2+H]^+$: 266.1176, found: 266.1169.

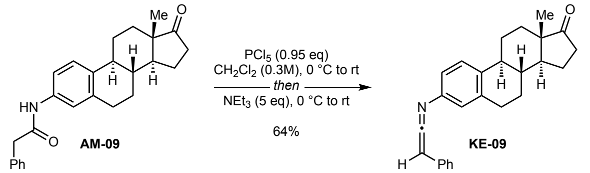

General Method A.

Purification: Solvent mixture A = 1:4 Et_2O:hexane, solvent mixture B = hexane.

Yield: 61.1 mg, 0.165 mmol, 64%;

Appearance: Brown oil;

R*f*: N/A (decomposes on glass-backed silica TLC plates);

^{1}H NMR (900 MHz, $CDCl_3$): δ 7.30 (d, *J* = 8.4 Hz, 1H), 7.27 (t, *J* = 7.7 Hz, 2H), 7.17 (d, *J* = 7.7 Hz, 2H), 7.14 (d, *J* = 8.3 Hz, 1H), 7.11 (t, *J* = 7.5 Hz, 1H), 7.08 (s, 1H), 5.23 (s, 1H), 2.97 – 2.85 (m, 2H), 2.51 (dd, *J* = 19.2, 8.9 Hz, 1H), 2.42 (d, *J* = 13.1 Hz, 1H), 2.31 (t, *J* = 8.8 Hz, 1H), 2.15 (dt, *J* = 18.7, 8.9 Hz, 1H), 2.10 – 2.01 (m, 2H), 1.98 (d, *J* = 12.5 Hz, 1H), 1.68 – 1.57 (m, 2H), 1.56 – 1.43 (m, 4H), 0.92 (s, 3H) ppm;

^{13}C NMR (225 MHz, $CDCl_3$): δ 220.8, 190.4, 139.8, 138.2, 138.1, 132.9, 129.0, 126.6, 125.6, 125.3, 124.1, 121.4, 60.8, 50.6, 48.1, 44.5, 38.2, 36.0, 31.7, 29.4, 26.5, 25.9, 21.7, 14.0 ppm;

IR: 3315, 3062, 3030, 2932, 2861, 2247, 1726, 1678, 1598, 1502, 1495, 1433, 1343, 1256, 911, 733 cm^{-1};

HRMS (ESI): calculated for $[C_{26}H_{27}NO+Na]^+$: 392.1990, found: 392.1991;

$[\alpha]_D^{20}$: +92.4 (c = 0.80, CH_2Cl_2).

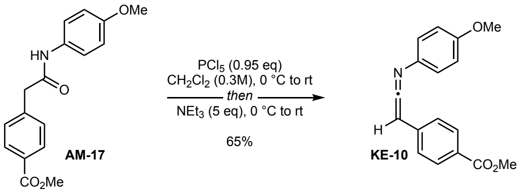

General Method A.

Purification: Solvent mixture A = 1:4 Et_2O:hexane, solvent mixture B = hexane.

Yield: 61.4 mg, 0.218 mmol, 65%;

Appearance: Yellow/orange oil;

R*f*: N/A (decomposes on glass-backed silica TLC plates);

^{1}**H NMR** (900 MHz, $CDCl_3$): δ 7.92 (d, *J* = 8.6 Hz, 2H), 7.30 (d, *J* = 9.0 Hz, 2H), 7.18 (d, *J* = 8.4 Hz, 2H), 6.90 (d, *J* = 9.0 Hz, 2H), 5.22 (s, 1H), 3.89 (s, 3H), 3.82 (s, 3H) ppm;

^{13}**C NMR** (225 MHz, $CDCl_3$): δ 186.3, 167.1, 159.7, 139.4, 131.9, 130.4, 126.6, 125.6, 125.1, 114.9, 60.6, 55.7, 52.1 ppm;

IR: 2949, 2005, 1721, 1711, 1604, 1583, 1502, 1434, 1282, 1251 cm^{-1};

HRMS (ESI): calculated for $[C_{17}H_{15}NO_3+H]^+$: 283.1130, found: 282.1124.

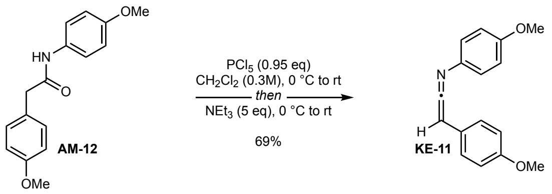

General Method A.

Purification: Solvent mixture A = 1:4 Et_2O:hexane, solvent mixture B = hexane.

Yield: 64.4 mg, 0.254 mmol, 69%;

Appearance: Yellow oil;

R*f*: N/A (decomposes on glass-backed silica TLC plates);

^{1}**H NMR** [400 MHz, $(CD_3)_2CO$]: δ 7.30 (d, *J* = 8.4 Hz, 2H), 7.10 (d, *J* = 8.3 Hz, 2H), 6.99 (d, *J* = 8.4 Hz, 2H), 6.88 (d, *J* = 8.2 Hz, 2H), 5.40 (s, 1H), 3.82 (s, 3H), 3.76 (s, 3H) ppm;

^{13}**C NMR** [100 MHz, $(CD_3)_2CO$]: δ 192.0, 160.3, 158.7, 134.4, 127.3, 125.7, 125.4, 115.6. 115.4, 60.5, 55.9, 55.6 ppm;

IR: 3001, 2932, 2835, 2003, 1605, 1582, 1502, 1462, 1294, 1247, 1180, 1105, 1033, 841 cm^{-1};

HRMS (ESI): calculated for $[C_{16}H_{15}NO_2+H]^+$: 254.1176, found: 254.1173.

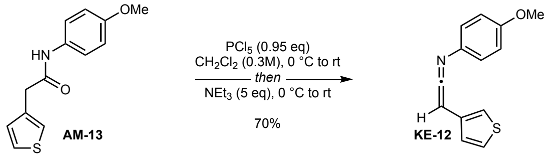

General Method A.

Purification: Solvent mixture A = 1:4 Et_2O:hexane, solvent mixture B = hexane.

Yield: 162 mg, 0.710 mmol, 70%;

Appearance: Orange oil;

R*f*: N/A (decomposes on glass-backed silica TLC plates);

^{1}H NMR (400 MHz, $CDCl_3$): δ 7.29 (d, *J* = 8.1 Hz, 3H), 6.96 – 6.92 (m, 2H), 6.89 (d, *J* = 7.9 Hz, 2H), 5.35 (s, 1H), 3.82 (s, 3H) ppm;

^{13}C NMR (100 MHz, $CDCl_3$): δ 191.2, 159.3, 133.3, 131.8, 126.2, 126.1, 125.2, 117.4, 114.7, 56.2, 55.6 ppm;

IR: 3100, 3020, 2956, 2835, 2003, 1602, 1583, 1502, 1462, 1441, 1384, 1298, 1249, 1163, 1105, 1032, 836 cm^{-1};

HRMS (ESI): calculated for $[C_{13}H_{11}NOS+H]^+$: 230.0634, found: 230.0630.

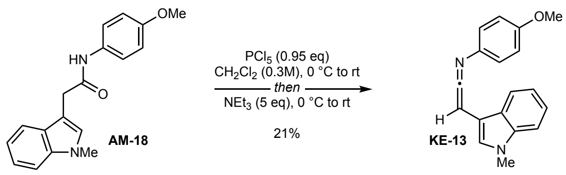

General Method A.

Purification: Solvent mixture A = 1:1 Et_2O:hexane, solvent mixture B = 1:1 Et_2O:hexane.

Yield: 48.6 mg, 0.176 mmol, 21%;

Appearance: Orange oil;

Rf: N/A (decomposes on glass-backed silica TLC plates);

^{1}H NMR (900 MHz, $CDCl_3$): δ 7.62 (d, *J* = 7.9 Hz, 1H), 7.39 (d, *J* = 8.0 Hz, 2H), 7.30 (d, *J* = 8.3 Hz, 1H), 7.24 (t, *J* = 7.6 Hz, 1H), 7.07 (t, *J* = 7.5 Hz, 1H), 6.96 (s, 1H), 6.91 (m, 2H), 5.52 (s, 1H), 3.82 (s, 3H), 3.75 (s, 3H) ppm;

^{13}C NMR (225 MHz, $CDCl_3$): δ 195.5, 159.1, 137.5, 135.0, 126.2, 125.1, 125.0, 122.2, 119.5, 119.30, 114.7, 109.5, 104.1, 55.6, 53.1, 32.9 ppm;

IR: 2912, 2000, 1602, 1502, 1462, 1442, 1326, 1298, 1248, 1027 cm^{-1};

HRMS (ESI): calculated for $[C_{18}H_{16}N_2O+Na]^+$: 299.1160, found: 299.1147.

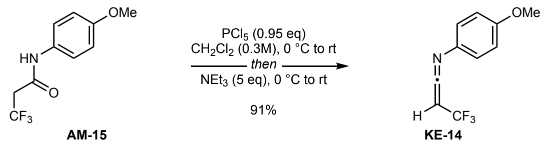

General Method A.

Purification: Solvent mixture A = 1:4 Et_2O:hexane, solvent mixture B = hexane.

Yield: 210. mg, 0.976 mmol, 91%;

Appearance: Yellow oil;

Rf: N/A (decomposes on glass-backed silica TLC plates);

^{1}H and ^{19}F NMR data matched those reported by Katagiri.[50] No ^{13}C NMR has been reported, so we include it here.

^{1}H NMR (400 MHz, $CDCl_3$): δ 7.27 (d, *J* = 8.8 Hz, 2H), 6.91 (d, *J* = 8.4 Hz, 2H), 4.45 (q, *J* = 6.5 Hz, 1H), 3.83 (s, 3H) ppm;

^{13}C NMR (225 MHz, $CDCl_3$): δ 176.7 (q, *J* = 5.3 Hz), 160.3, 129.8 (q, *J* = 2.5 Hz), 126.1, 126.0 (q, *J* = 267.5 Hz), 115.0, 55.7, 51.6 (q, *J* = 41.4 Hz) ppm.

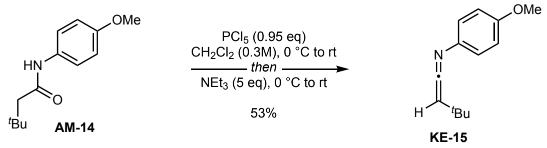

General Method A.

Purification: Solvent mixture A = 1:4 Et_2O:hexane, solvent mixture B = hexane.

While characterization data matched those reported by Moderhack,[51] no ^{13}C NMR data has been reported, so we include it here.

Yield: 121 mg, 0.594 mmol, 53%;

Appearance: Yellow oil;

R*f*: N/A (decomposes on glass-backed silica TLC plates);

^{1}H NMR (900 MHz, $CDCl_3$): δ 7.25 (d, *J* = 8.7 Hz, 2H), 6.88 (d, *J* = 8.7 Hz, 2H), 4.12 (s, 1H), 3.81 (s, 3H), 1.16 (s, 9H) ppm;

^{13}C NMR (225 MHz, $CDCl_3$): δ 191.5, 158.8, 135.6, 124.3, 114.6, 68.2, 55.7, 31.7, 31.0 ppm.

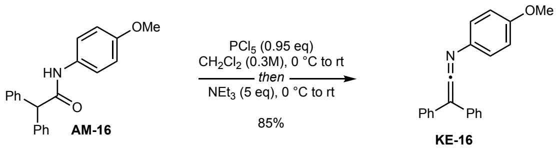

General Method A.

Purification: Solvent mixture A = 1:4 Et_2O:hexane, solvent mixture B = hexane.

Characterization data matched those reported by Lu and Wang.[52]

Yield: 0.988 g, 3.30 mmol, 85%;

Appearance: Yellow solid;

R*f*: N/A (decomposes on glass-backed silica TLC plates);

^{1}H NMR (400 MHz, $CDCl_3$): δ 7.37 – 7.34 (m, 10H), 7.24 – 7.22 (m, 2H), 6.91 (d, *J* = 8.4 Hz, 2H), 3.83 (s, 3H) ppm.

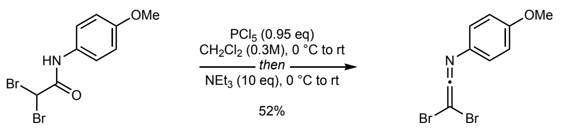

AM-20 **KE-17**

Slight modification of General Method A: Triethylamine stoichiometry increased to 10.0 equiv, and time spent stirring at ambient temperature following addition of triethylamine increased to 17 hours.

Purification: Solvent mixture A = 1:4 Et_2O:hexane, solvent mixture B = hexane.

Yield: 0.247 g, 0.810 mmol, 52%;

Appearance: Yellow oil (darkens when held neat);

R*f*: N/A (decomposes on glass-backed silica TLC plates);

^{1}H NMR (600 MHz, $CDCl_3$): δ 7.31 (d, *J* = 8.9 Hz, 2H), 6.93 (d, *J* = 8.7 Hz, 2H), 3.85 (s, 3H) ppm;

^{13}C NMR (100 MHz, $CDCl_3$): δ 186.4, 160.7, 130.9, 127.1, 115.0, 55.8, 36.4 ppm;

IR: 2963, 2931, 2836, 1998, 1691, 1599, 1582, 1502, 1301, 1247, 1181, 1127, 1033 cm^{-1};

HRMS (ESI): calculated for $[C_9H_7NOBr_2+H]^+$: 305.8947, found: 305.8948.

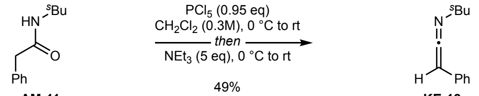

AM-11 **KE-18**

General Method A.

Purification: Solvent mixture A = 1:4 Et_2O:hexane, solvent mixture B = hexane.

Yield: 132 mg, 0.762 mmol, 49%;

Appearance: Yellow oil;

R*f*: N/A (decomposes on glass-backed silica TLC plates);

^{1}H NMR (400 MHz, $CDCl_3$): δ 7.26 (t, *J* = 7.0 Hz, 2H), 7.12 (d, *J* = 7.6 Hz, 2H), 7.06 (t, *J* = 7.6 Hz, 1H), 4.80 (s, 1H), 3.64 (q, *J* = 6.7 Hz, 1H), 1.74 – 1.61 (m, 2H), 1.33 (d, *J* = 6.5 Hz, 3H), 1.03 (t, *J* = 7.7 Hz, 3H) ppm;

^{13}C NMR (100 MHz, $CDCl_3$) δ 184.7, 134.6, 128.9, 125.0, 124.5, 60.9, 58.1, 30.7, 21.4, 10.9 ppm;

IR: 3029, 2969, 2931, 2876, 2023, 1672, 1659, 1598, 1495, 1451, 1377, 1327, 1178, 759 cm^{-1};

HRMS (ESI): calculated for $[C_{12}H_{15}N+H]^+$: 174.1277, found: 174.1280.

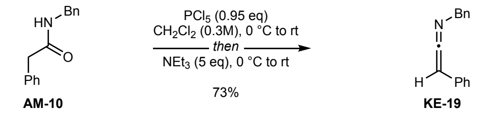

Slight modification of General Method A: Time spent stirring at ambient temperature following addition of triethylamine increased to 2 hours.

Purification: Solvent mixture A = 1:4 Et_2O:hexane, solvent mixture B = hexane.

Characterization data matched those reported by Shimizu.[53]

Yield: 0.674 g, 3.25 mmol, 73%;

Appearance: Yellow oil;

R*f*: N/A (decomposes on glass-backed silica TLC plates);

^{1}H NMR (400 MHz, $CDCl_3$): δ 7.42 – 7.29 (m, 5H), 7.24 (t, *J* = 7.0 Hz, 2H), 7.07 (t, *J* = 8.0 Hz, 3H), 4.84 (br s, 1H), 4.73 (br s, 2H) ppm.

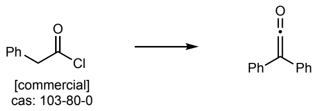

Diphenylketene was prepared in one step according to the procedure of Hawks.[54] It was routinely isolated with variable quantities of benzophenone, which was mitigated on larger scales. A representative run employing 1 g of phenylacetyl chloride provided a 4:1 mixture of diphenylketene and benzophenone (53% yield of diphenylketene, determined by ^{1}H NMR). Benzophenone did not appear to impact the subsequent reaction, and this mixture was employed without further purification. Characterization data for diphenylketene matched those reported by Fillion.[55]

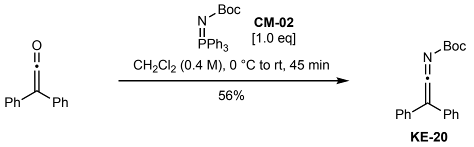

Reaction adapted from a related procedure reported by Würthwein.[56] To a flame-dried 25 mL one-neck round-bottom flask under argon and equipped with a stir bar was added diphenylketene (1.56 mmol, 1.00 equiv, contaminated with benzophenone) and anhydrous CH_2Cl_2 (2.90 mL, 0.40 M). The round-bottom flask was submerged in an ice/water bath and **CM-02** (437 mg, 1.16 mmol, 1.00 equiv) was added. The reaction was warmed to ambient temperature, stirred for 45 minutes, and concentrated under reduced pressure. The crude mixture was filtered through a Celite plug (eluting with hexane), concentrated under reduced pressure, diluted with hexane, and filtered over phosphate buffered silica (pH = 7, ratio of buffered silica to crude mass = 5:1, eluting with 1:1 hexane:Et_2O). The filtrate was concentrated under reduced pressure to afford **KE-20** contaminated with benzophenone (ca. 1:2.5 **KE-20**:benzophenone).

Benzophenone did not appear to impact the subsequent reaction, and this mixture was employed without further purification.

Yield: 190 mg, 0.648 mmol, 56%;

Appearance: Yellow oil;

R*f*: 0.47 (5:1 hexane:Et_2O);

^{1}H NMR (600 MHz, $CDCl_3$): δ 7.40 – 7.33 (m, 8H), 7.28 (t, J = 6.7 Hz, 2H), 1.57 (s, 9H) ppm;

^{13}C NMR (150 MHz, $CDCl_3$): δ 195.9, 153.0, 132.3, 129.0, 127.3, 121.8, 85.2, 77.6, 27.8 ppm;

IR: 3060, 3028, 2980, 2021, 1745, 1659, 1598, 1494, 1446, 1370, 1318, 1278, 1234, 1144, 942, 764 cm^{-1};

HRMS (ESI): calculated for $[C_{19}H_{19}NO_2+Na]^+$: 316.1313, found: 316.1310.

Ketenimine Stability Studies

Stability studies adapted from those originally disclosed by Sherburn.[57]

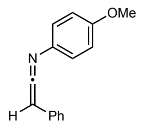

KE-03 (or **2a**)

**water** (biphasic)	≥95%	**silica** (plug)	88%
**water** (homogeneous)	≥95%	**silica** (column)	0%
**MeOH**	≥95%	**buffered silica** (plug)	≥95%
**AcOH**	0%	**buffered silica** (column)	traces
**oxygen**	≥95%	**neat** (open to air)	90%
**heat**	87%	**neat** (Ar atmosphere)	≥95%

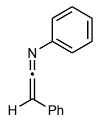

KE-01

**water** (biphasic)	≥95%
**water** (homogeneous)	≥95%

Water (Biphasic)

To a 2-dram vial equipped with a stir bar was added a 0.10 M $CDCl_3$ solution of pure ketenimine ($CDCl_3$ stored over K_2CO_3 to neutralize any trace acid present). BHT (ca. 2.00 mg/mL) was added to inhibit the autoxidative effects of any oxygen present in solution, followed by an internal standard (durene). An initial ^{1}H NMR spectrum was obtained. An equivalent volume of deionized H_2O was added, the vial was sealed under a blanket of argon, and the biphasic mixture was vigorously stirred for 1 hour. $MgSO_4$ was added to remove H_2O, and a second ^{1}H NMR spectrum was obtained to determine whether any decomposition had occurred.

Water (Homogeneous)

To a 2-dram vial equipped with a stir bar was added a 0.1 M $(CD_3)_2CO/D_2O$ (9:1) solution of pure ketenimine. BHT (ca. 2.00 mg/mL) was added to inhibit the autoxidative effects of any oxygen present in solution, followed by an internal standard (durene). An initial ^{1}H NMR spectrum was obtained. The vial was sealed under a blanket of argon, and the sample was stirred for 1 hour. A second ^{1}H NMR spectrum was obtained to determine whether any decomposition had occurred.

Methanol

To a 2-dram vial equipped with a stir bar was added a 0.1 M $CD_3OD/CDCl_3$ (4:1) solution of pure ketenimine ($CDCl_3$ stored over K_2CO_3 to neutralize any trace acid present). BHT (ca. 2.00 mg/mL) was added to inhibit the autoxidative effects of any oxygen present in solution, followed by an internal standard (durene). An initial ^{1}H NMR spectrum was obtained. The vial was sealed under a blanket of argon, and the sample was stirred for 1 hour. A second ^{1}H NMR spectrum was obtained to determine whether any decomposition had occurred.

Acetic Acid

To a 2-dram vial equipped with a stir bar was added a 0.10 M $CDCl_3$ solution of pure ketenimine. BHT (ca. 2.00 mg/mL) was added to inhibit the autoxidative effects of any oxygen present in solution, followed by an internal standard (durene). An initial ^{1}H NMR spectrum was obtained. An equivalent volume of 0.20 M AcOH in $CDCl_3$ was added, the vial was sealed under a blanket of argon, and the sample was stirred for 1 hour. A second ^{1}H NMR spectrum was obtained to determine whether any decomposition had occurred.

Oxygen

To a 2-dram vial equipped with a stir bar was added a 0.10 M $CDCl_3$ solution of pure ketenimine ($CDCl_3$ stored over K_2CO_3 to neutralize any trace acid present). An internal standard (durene) was added, and an initial ^{1}H NMR spectrum was obtained. The vial was fitted with a rubber septum, anhydrous O_2 gas (balloon) was bubbled through the solution for five minutes, and then the vial stirred under an O_2 atmosphere for 1 hour (balloon still attached). A second ^{1}H NMR spectrum was obtained to determine whether any decomposition had occurred.

Heat

To a flame-dried 10 mL microwave vessel equipped with a stir bar was added pure ketenimine, BHT (ca. 2.00 mg/mL) to inhibit the autoxidative effects of any oxygen present in solution, and an internal standard (durene). The vessel was equipped with a rubber septum and evacuated and purged with argon (×3). Sufficient C_6D_6 was added to generate a 0.10 M solution of ketenimine, and an initial ^{1}H NMR spectrum was obtained. The rubber septum was replaced with a microwave cap and the sample was heated to 120 °C for 2 hours in a microwave reactor. A second ^{1}H NMR spectrum was obtained to determine whether any decomposition had occurred.

Silica (Plug)

A monster pipette was charged with 150 mg of silica. The silica was pre-wet with hexanes, 30.0 mg of pure ketenimine was loaded onto the silica using a minimum volume of 4:1 Et_2O/hexanes (ca. 0.500 mL), and the pipette was eluted with 10.0 mL of 4:1 Et_2O/hexanes directly into a round-bottom flask. The solvent was removed under reduced pressure, two internal standards (dibromomethane and 1,2-dichloroethane) were added, and an ^{1}H NMR spectrum was obtained to determine the percent recovery of ketenimine.

Silica (Column)

A flash chromatography column was charged with 5.00 g of silica. The silica was pre-wet with hexanes, 50.0 mg of pure ketenimine was loaded onto the silica using a minimum volume of CH_2Cl_2 (ca. 0.500 mL), and the column was eluted with 50.0 mL of hexane and then 50.0 mL of EtOAc directly into a round-bottom flask. The solvent was removed under reduced pressure, two internal standards (dibromomethane and 1,2-dichloroethane) were added, and an ^{1}H NMR spectrum was obtained to determine the percent recovery of ketenimine.

Buffered Silica (Plug)

A monster pipette was charged with 150 mg of phosphate buffered silica (pH = 7). The silica was pre-wet with hexanes, 30.0 mg of pure ketenimine was loaded onto the silica using a minimum volume of 4:1 Et_2O/hexanes (ca. 0.500 mL), and the pipette was eluted with 10.0 mL of 4:1 Et_2O/hexanes directly into a round-bottom flask. The solvent was removed under reduced pressure, two internal standards (dibromomethane and 1,2-dichloroethane) were added, and an ^{1}H NMR spectrum was obtained to determine the percent recovery of ketenimine.

Buffered Silica (Column)

A flash chromatography column was charged with 5.00 g of phosphate buffered silica (pH = 7). The silica was pre-wet with hexanes, 50.0 mg of pure ketenimine was loaded onto the silica using a minimum volume of CH_2Cl_2 (ca. 0.500 mL), and the column was eluted with 50.0 mL of hexane and then 50.0 mL of EtOAc directly into a round-bottom flask. The solvent was removed under reduced pressure, two internal standards (dibromomethane and 1,2-dichloroethane) were added, and an ^{1}H NMR spectrum was obtained to determine the percent recovery of ketenimine.

Neat (Open to Air)

To a 2-dram vial open to air was added pure ketenimine. The vial was sealed, and the sample was kept for 24 hours at ambient temperature. Two internal standards (dibromomethane and 1,2-dichloroethane) were added, and an ^{1}H NMR spectrum was obtained to determine whether any decomposition had occurred.

Neat (Argon Atmosphere)

To a 2-dram vial open to air was added pure ketenimine. The vial was equipped with a rubber septum and evacuated and purged with argon (×3). The vial was sealed (still under argon) and the sample was kept for 24 hours at ambient temperature. Two internal standards (dibromomethane and 1,2-dichloroethane) were added and an ^{1}H NMR spectrum was obtained to determine whether any decomposition had occurred.

Bis(silyloxy)furan + Ketenimine Aza-Diels–Alder Reactions

Ring-Opened Products

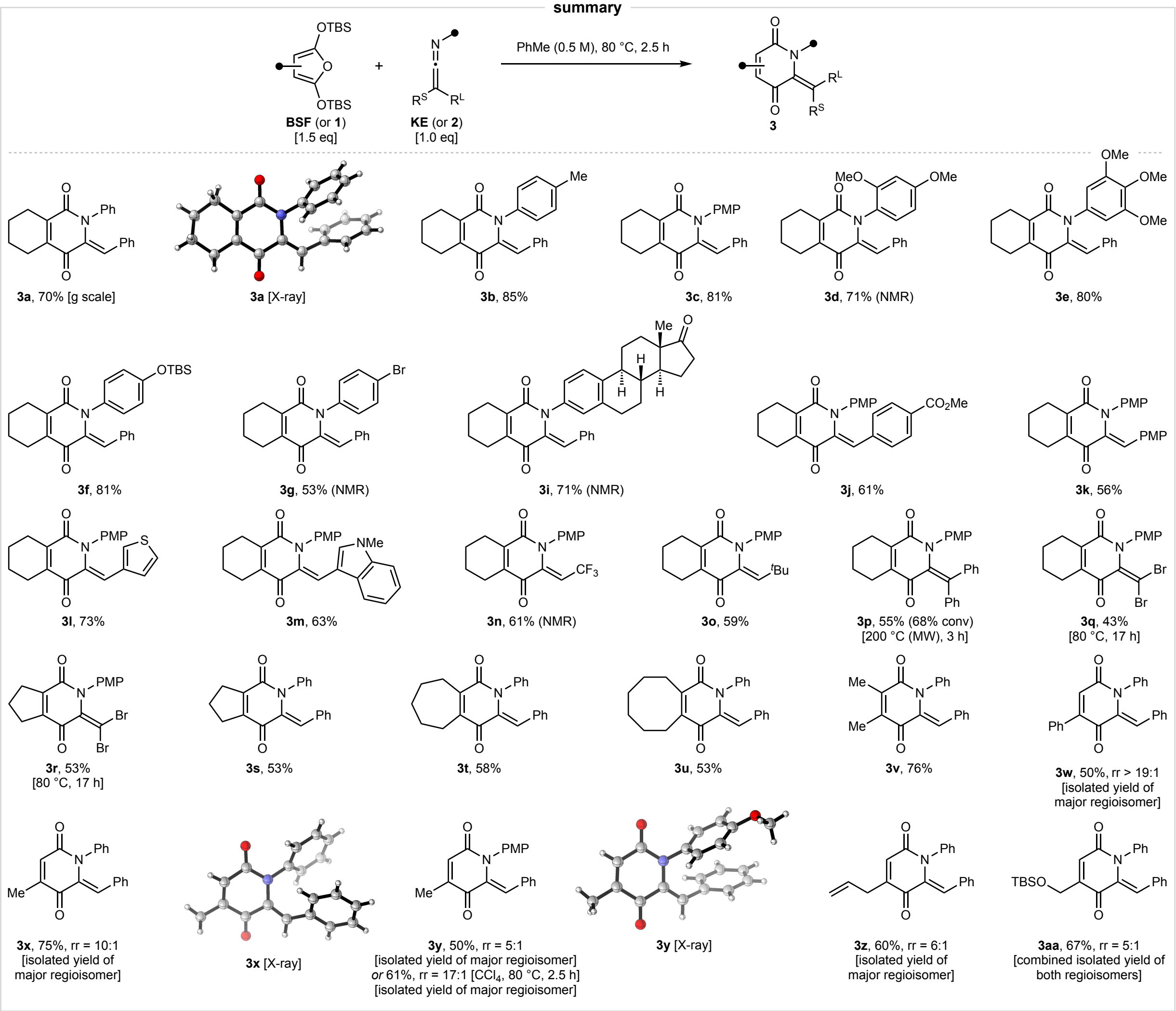

General Procedure

Given the propensity of silyloxyfurans and ketenimines to decompose if not appropriately handled (see notes concerning stability and storage in their respective preparation sections earlier in this document), directly before each Diels–Alder reaction, the purity of both was determined by ^{1}H NMR using a suitable internal standard (1,2-dibromomethane, 1,2-dichloroethane or durene). To a flame-dried one-neck round-bottom flask under argon and equipped with a stir bar was added the appropriate ketenimine (1.00 equiv). Following addition of anhydrous toluene (0.50 M), the appropriate furan (1.50 equiv) was added, and the reaction was warmed on a heating mantle at 80 °C 2.5 hours. The reaction was cooled to ambient temperature and concentrated under reduced pressure to afford the corresponding crude ring-opened Diels–Alder cycloadduct. Specific purification details for each are provided alongside their characterization data below.

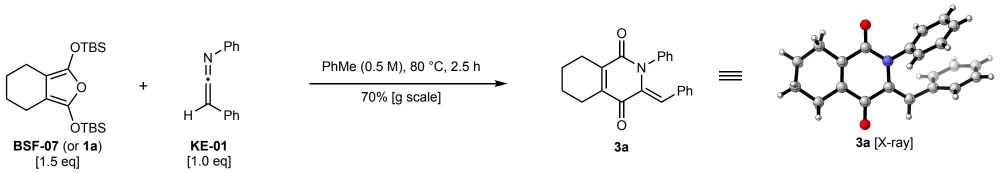

Prepared according to the general procedure.

Purification: Crude residue dissolved in hexane and transferred to a freezer (−20 °C) to sit overnight, allowing a solid to crash out. Filtration (washing with cold hexane) yielded **3a**. A small quantity of pure **3a** was crystallized via the slow evaporation of acetone to yield yellow needle-like crystals suitable for X-ray analysis.

Yield: 1.60 g, 6.99 mmol, 70%;

Appearance: Yellow solid;

R*f*: 0.31 (49:49:2 hexane:CH_2Cl_2:Et_2O);

M.p.: 125 – 130 °C;

^{1}H NMR (400 MHz, $CDCl_3$): δ 7.30 (s, 1H), 7.08 (d, *J* = 8.6 Hz, 2H), 7.01 (t, *J* = 7.8 Hz, 2H), 6.93 (m, 4H), 6.85 (d, *J* = 7.7 Hz, 2H), 2.61 (app d, *J* = 29.3 Hz, 2H), 1.78 (br s, 4H) ppm;

^{13}C NMR (100 MHz, $CDCl_3$): δ 181.9, 160. 7, 145.0, 144.0, 137.9, 134.9, 133.8, 129.1, 128.3, 128.2, 127.5, 127.3, 127.0, 123.9, 25.2, 23.0, 21.5, 21.1 ppm;

IR: 2937, 1666, 1593, 1493, 1402, 1370, 1291 cm^{-1};

HRMS (ESI): calculated for $[C_{22}H_{19}NO_2+H]^+$: 330.1489, found: 330.1476.

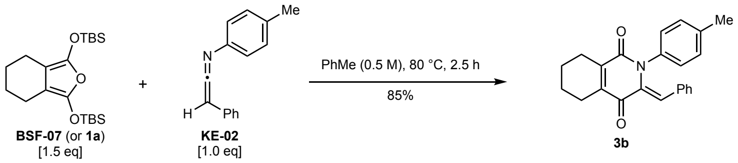

Prepared according to the general procedure.

Purification: Crude residue dissolved in hexane and transferred to a freezer (−20 °C) to sit overnight, allowing a solid to crash out. Filtration (washing with cold hexane) yielded **3b**.

Yield: 177 mg, 0.515 mmol, 85%;

Appearance: Yellow solid;

R*f*: 0.26 (1:9 EtOAc:hexane);

^{1}H NMR (400 MHz, $CDCl_3$): δ 7.28 (s, 1H), 6.98 – 6.87 (m, 5H), 6.80 (m, 4H), 2.60 (app d, *J* = 28.7 Hz, 4H), 2.11 (s, 3H), 1.76 (br s, 4H) ppm;

^{13}C NMR (100 MHz, $CDCl_3$): δ 181.8, 160.6, 144.9, 143.8, 136.7, 135.3, 135.0, 133.8, 129.0, 128.6, 128.0, 127.1, 127.1, 123.6, 25.1, 22.9, 21.4, 21.0, 20.9 ppm;

IR: 3042, 2944, 2877, 1666, 1641, 1582, 1511, 1402, 1315, 1293, 1180, 979, 749 cm^{-1};

HRMS (ESI): calculated for $[C_{23}H_{21}NO_2+H]^+$: 344.1651, found: 344.1652.

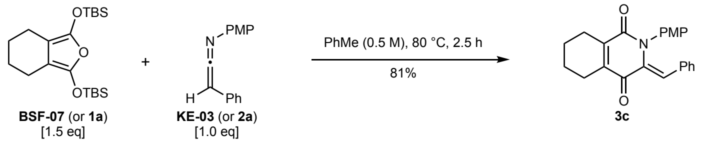

BSF-07 (or **1a**) [1.5 eq] + **KE-03** (or **2a**) [1.0 eq] → **3c**

Prepared according to the general procedure.

Purification: Crude residue dissolved in hexane and transferred to a freezer (−20 °C) to sit overnight, allowing a solid to crash out. Filtration (washing with cold hexane), followed by flash column chromatography (ratio of silica to crude mass = 100:1, eluting with 99:1 CH_2Cl_2:Et_2O) yielded **3c.**

Yield: 131 mg, 0.360 mmol, 81%;

Appearance: Dark yellow solid;

R*f*: 0.33 (CH_2Cl_2);

^{1}H NMR (400 MHz, $CDCl_3$): δ 7.29 (s, 1H), 6.99 – 6.90 (m, 5H), 6.82 (d, *J* = 6.7 Hz, 2H), 6.52 (d, *J* = 8.9 Hz, 2H), 3.64 (s, 3H), 2.60 (app d, *J* = 29.7 Hz, 4H), 1.77 (br s, 4H) ppm;

^{13}C NMR (100 MHz, $CDCl_3$): δ 181.9, 160.8, 158.3, 145.0, 144.0, 135.2, 134.0, 130.8, 129.4, 129.0, 127.3, 127.3, 123.6, 113.5, 55.5, 25.2, 23.0, 21.5, 21.1 ppm;

IR: 2940, 1666, 1640, 1584, 1511, 1453, 1250, 726 cm^{-1};

HRMS (ESI): calculated for $[C_{23}H_{21}NO_3+H]^+$: 360.1594, found: 360.1577.

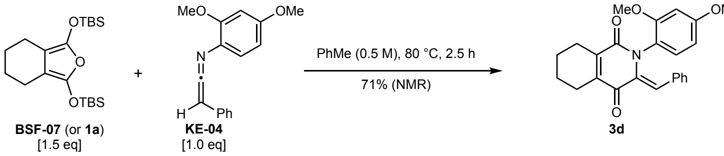

BSF-07 (or **1a**) [1.5 eq] + **KE-04** [1.0 eq] → **3d**

Prepared according to the general procedure.

Purification: Compound **3d** is prone to olefin isomerization/decomposition during purification. For characterization purposes, a small quantity of pure **3d** was isolated via trituration of the crude residue with cold hexane, followed by flash column chromatography using phosphate buffered silica (pH = 7, ratio of buffered silica to crude mass = 100:1, eluting with 98:2 CH_2Cl_2:Et_2O).

Crude Yield: 0.186 mmol, 71% (NMR yield, determined using CH_2Br_2 as internal standard);

Appearance: Dark yellow solid;

R*f*: 0.38 (3:2 hexane:EtOAc);

^{1}H NMR (400 MHz, $CDCl_3$): δ 7.27 (s, 1H), 6.94 (m, 3H), 6.77 (app dd, J = 16.2, 7.8 Hz, 3H), 6.15 (d, J = 8.5 Hz, 1H), 6.00 (s, 1H), 3.65 (s, 6H), 2.60 (app d, J = 28.7 Hz, 4H), 1.77 (br s, 4H) ppm;

^{13}C NMR (100 MHz, $CDCl_3$): δ 181.5, 160.6, 160.3, 155.4, 145.0, 143.8, 134.9, 134.5, 130.1, 128.2, 126.9, 126.6, 122.5, 120.1, 104.4, 98.9, 55.6, 55.3, 25.1, 23.1, 21.9, 21.1 ppm;

IR: 3001, 2935, 2836, 1666, 1650, 1605, 1529, 1512, 1494, 1468, 1453, 1284, 1209, 1160, 1034, 832 cm^{-1};

HRMS (ESI): calculated for $[C_{24}H_{23}NO_4+H]^+$: 390.1705, found: 390.1696.

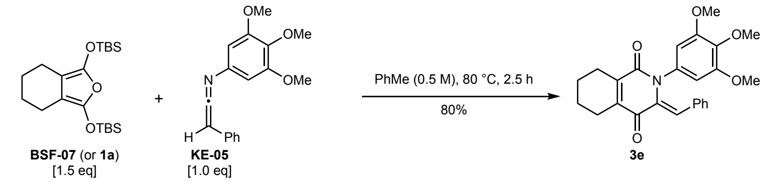

Prepared according to the general procedure.

Purification: Crude residue purified by flash column chromatography using phosphate buffered silica (pH = 7, ratio of buffered silica to crude mass = 100:1, eluting with 5:95 Et_2O:CH_2Cl_2).

Yield: 190 mg, 0.453 mmol, 80%;

Appearance: Dark yellow solid;

R_f: 0.33 (5:95 Et_2O: CH_2Cl_2);

^{1}H NMR (400 MHz, $CDCl_3$): δ 7.32 (s, 1H), 7.03 – 6.96 (m, 3H), 6.84 (d, J = 6.9 Hz, 2H), 6.28 (s, 2H), 3.67 (s, 6H), 3.62 (s, 3H), 2.60 (app d, J = 29.5 Hz, 4H), 1.81 – 1.72 (br s, 4H) ppm;

^{13}C NMR (100 MHz, $CDCl_3$): δ 181.5, 160.6, 152.6, 144.9, 144.1, 136.9, 135.0, 134.2, 133.2, 128.3, 127.5, 127.1, 123.8, 106.8, 60.7, 56.1, 25.2, 23.0, 21.4, 21.0 ppm;

IR: 2937, 1666, 1598, 1502, 1462, 1446, 1306, 1234, 1136, 981 cm^{-1};

HRMS (ESI): calculated for $[C_{25}H_{25}NO_5+H]^+$: 420.1811, found: 420.1806.

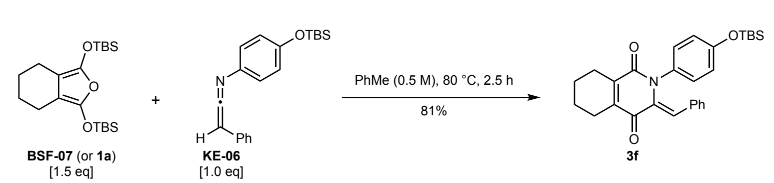

Prepared according to the general procedure.

Purification: Crude residue purified by flash column chromatography using phosphate buffered silica (pH = 7, ratio of buffered silica to crude mass = 50:1, eluting with a gradient of 1:20 EtOAc:hexane to 1:10 EtOAc:hexane). The

resulting yellow oil was triturated with cold hexane to yield **3f**. The filtrate was concentrated under reduced pressure and the trituration process was repeated to obtain a second crop of **3f**.

Yield: 442 mg, 0.962 mmol, 81%;

Appearance: Yellow solid;

R*f*: 0.36 (4:1 hexane:EtOAc);

^{1}H NMR (600 MHz, $CDCl_3$): δ 7.27 (s, 1H), 6.99 – 6.96 (m, 3H), 6.93 (d, *J* = 8.8 Hz, 2H), 6.90 – 6.87 (m, 2H), 6.46 (d, *J* = 8.8 Hz, 2H), 2.63 (m, 2H), 2.57 (m, 2H), 1.81 – 1.72 (m, 4H), 0.91 (s, 9H), 0.05 (s, 6H) ppm;

^{13}C NMR (150 MHz, $CDCl_3$): δ 182.1, 160.7, 154.2, 145.1, 143.9, 135.3, 133.8, 131.5, 129.3, 129.2, 127.7, 127.3, 123.8, 119.5, 25.8, 25.2, 23.0, 21.5, 21.1, 18.2, –4.4 ppm;

IR: 3055, 2935, 2884, 2858, 1678, 1666, 1650, 1600, 1493, 1469, 1403, 1310, 1292, 1252, 1167, 979, 919, 841 cm^{-1};

HRMS (ESI): calculated for $[C_{28}H_{33}NO_3Si+H]^+$: 460.2308, found: 460.2292.

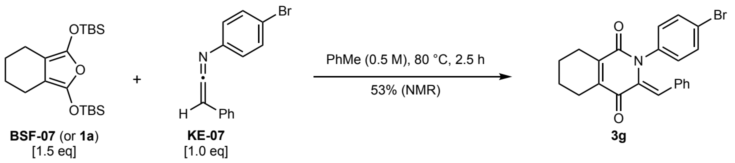

Prepared according to the general procedure.

Purification: Compound **3g** is prone to olefin isomerization/decomposition during purification. For characterization purposes a small quantity of pure **3g** was isolated via trituration with cold hexane.

Crude Yield: 0.526 mmol, 53% (NMR yield, determined using durene as internal standard);

Appearance: Yellow solid;

R*f*: 0.20 (1:9 Et_2O:hexane);

^{1}H NMR [400 MHz, $(CD_3)_2CO$]: δ 7.18 (d, *J* = 8.5 Hz, 3H), 7.13 (d, *J* = 8.5 Hz, 2H), 7.02 (m, *J* = 7.4 Hz, 3H), 6.96 (d, *J* = 7.2 Hz, 2H), 2.54 (app d, *J* = 21.4 Hz, 4H), 1.76 (br s, 4H) ppm;

^{13}C NMR [100 MHz, $(CD_3)_2CO$]: δ 181.8, 160.8, 144.9, 144.3, 138.6, 136.0, 134.8, 131.6, 131.5, 130.0, 128.2, 128.1, 122.7, 120.4, 25.6, 23.4, 22.0, 21.6 ppm;

IR: 3055, 2934, 2351, 1666, 1640, 1585, 1458, 1288 cm^{-1};

HRMS (ESI): calculated for $[C_{22}H_{18}BrNO_2+H]^+$: 408.0599, found: 408.0595.

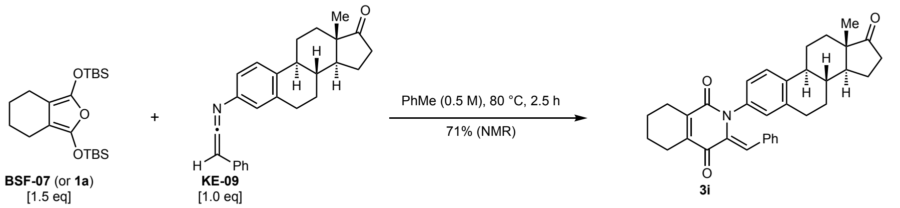

Prepared according to the general procedure.

Purification: Compound **3i** is prone to olefin isomerization/decomposition during purification. For characterization purposes a small quantity of pure **3i** was isolated via trituration with cold hexane, followed by flash column chromatography using phosphate buffered silica (pH = 7, ratio of buffered silica to crude mass = 50:1, eluting with 9:1 hexane:EtOAc).

Crude Yield: 0.0607 mmol, 71% (NMR yield, determined using durene as internal standard);

Appearance: Yellow solid;

R*f*: 0.19 (1:4 EtOAc:hexane);

^{1}H NMR (600 MHz, $CDCl_3$): δ 7.28 (s, 1H), 6.95 – 6.89 (m, 4H), 6.85 (d, *J* = 2.3 Hz, 1H), 6.78 – 6.74 (m, 2H), 6.62 (s, 1H), 2.65 (br s, 2H), 2.59 – 2.54 (m, 4H), 2.50 (dd, *J* = 10.3, 8.8 Hz, 1H), 2.25 (m, 1H), 2.17 – 2.08 (m, 1H), 2.08 – 2.00 (m, 2H), 1.92 (d, *J* = 3.2 Hz, 1H), 1.89 – 1.85 (m, 1H), 1.78 (br s, 4H), 1.63 – 1.55 (m, 1H), 1.47 – 1.40 (m, 2H), 1.38 – 1.30 (m, 2H), 1.25 – 1.20 (m, 1H), 0.90 (s, 3H) ppm;

^{13}C NMR (150 MHz, $CDCl_3$): δ 220.7, 181.6, 160.6, 145.0, 144.0, 138.7, 136.4, 135.3, 135.1, 134.6, 129.1, 128.5, 126.9, 126.7, 125.8, 125.5, 123.2, 50.6, 48.0, 44.3, 38.0, 36.0, 31.7, 29.3, 26.4, 25.8, 25.2, 23.1, 21.8, 21.6, 21.1, 13.9 ppm;

IR: 3087, 2933, 2859, 1738, 1666, 1640, 1584, 1494, 1452, 1407, 1310, 1218, 736 cm^{-1};

HRMS (ESI): calculated for $[C_{34}H_{35}NO_3+H]^+$: 506.2695, found: 506.2695;

$[\alpha]_D^{20}$: +68.1 (c = 0.24, CH_2Cl_2).

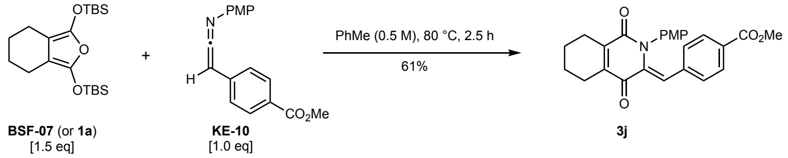

Prepared according to the general procedure.

Purification: Crude residue purified by flash column chromatography using phosphate buffered silica (pH = 7, ratio of buffered silica to crude mass = 100:1, eluting with 1:99 Et_2O:CH_2Cl_2).

Yield: 138 mg, 0.331 mmol, 61%;

Appearance: Yellow solid;

R*f*: 0.40 (2:98 Et_2O:CH_2Cl_2);

^{1}H NMR (900 MHz, $CDCl_3$): δ 7.62 (d, *J* = 8.2 Hz, 2H), 7.25 (s, 1H), 6.92 (d, *J* = 8.9 Hz, 2H), 6.87 (d, *J* = 8.6 Hz, 2H), 6.51 (d, *J* = 9.0 Hz, 2H), 3.87 (s, 3H), 3.61 (s, 3H), 2.64 (m, 2H), 2.57 (m, 2H), 1.80 – 1.74 (m, 4H) ppm;

^{13}C NMR (225 MHz, $CDCl_3$): δ 181.5, 166.7, 160.7, 158.5, 145.2, 144.0, 139.0, 136.2, 130.7, 129.5, 128.8, 128.4, 128.4, 121.5, 113.7, 55.5, 52.2, 25.2, 23.0, 21.4, 21.0 ppm;

IR: 2942, 2836, 1722, 1712, 1666, 1640, 1606, 1586, 1512, 1433, 1408, 1284, 1254, 1104 cm^{-1};

HRMS (ESI): calculated for $[C_{25}H_{23}NO_5+H]^+$: 418.1654, found: 418.1649.

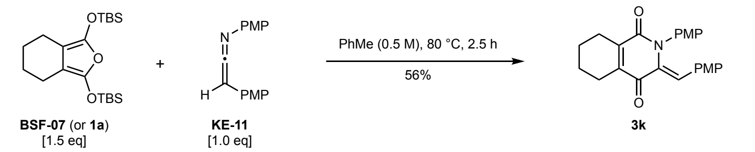

Prepared according to the general procedure.

Purification: Crude residue dissolved in hexane and transferred to a freezer (−20 °C) to sit overnight, allowing a solid to crash out. Filtration (washing with cold hexane) yielded **3k.**

Yield: 196 mg, 0.506 mmol, 56%;

Appearance: Orange solid;

R*f*: 0.33 (5:95 Et_2O: CH_2Cl_2);

^{1}H NMR (600 MHz, $CDCl_3$): δ 7.25 (s, 1H), 7.02 (d, *J* = 9.0 Hz, 2H), 6.82 (d, *J* = 8.9 Hz, 2H), 6.57 (d, *J* = 9.0 Hz, 2H), 6.49 (d, *J* = 8.7 Hz, 2H), 3.69 (s, 3H), 3.65 (s, 3H), 2.65 – 2.62 (m, 2H), 2.58 – 2.52 (m, 2H), 1.78 – 1.73 (m, 4H) ppm;

^{13}C NMR (150 MHz, $CDCl_3$): δ 182.0, 160.8, 159.2, 158.1, 144.7, 144.0, 134.0, 131.0, 130.9, 129.1, 126.1, 124.1, 113.4, 112.9, 55.5, 55.3, 25.2, 23.0, 21.5, 21.0 ppm;

IR: 2937, 2836, 1665, 1660, 1605, 1512, 1503, 1442, 1298, 1250 cm^{-1};

HRMS (ESI): calculated for $[C_{24}H_{23}NO_4+H]^+$: 390.1705, found: 390.1707.

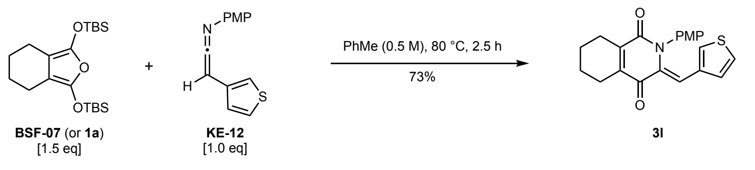

Prepared according to the general procedure.

Purification: Crude residue purified via trituration with cold hexane.

Yield: 188 mg, 0.514 mmol, 73%;

Appearance: Orange solid;

R*f*: 0.14 (1:5 EtOAc:hexane);

^{1}H NMR (400 MHz, $CDCl_3$): δ 7.22 (s, 1H), 7.07 (d, *J* = 9.3 Hz, 2H), 6.93 – 6.87 (br s, 1H), 6.71 (br s, 1H), 6.65 (d, *J* = 8.7 Hz, 2H), 6.55 (d, *J* = 4.9 Hz, 1H), 3.71 (s, 3H), 2.59 (app d, *J* = 34.0 Hz, 4H), 1.76 (s, 4H) ppm;

^{13}C NMR (100 MHz, $CDCl_3$): δ 181.9, 160.8, 158.4, 144.8, 144.0, 134.5, 134.3, 130.8, 129.0, 128.7, 126.5, 124.1, 118.2, 113.6, 55.6, 25.2, 23.0, 21.5, 21.0 ppm;

IR: 3103, 2937, 2860, 2835, 1688, 1659, 1632, 1583, 1512, 1408, 1315, 1286, 1249, 1179, 1033, 980, 831 cm^{-1};

HRMS (ESI): calculated for $[C_{21}H_{19}NO_3S+H]^+$: 366.1164, found: 366.1153.

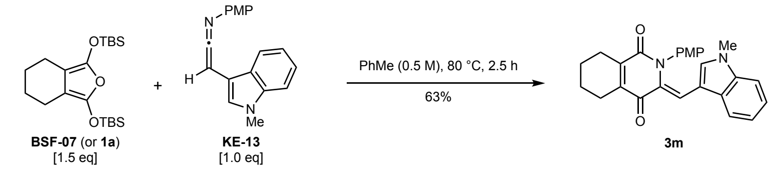

Prepared according to the general procedure.

Purification: Crude residue triturated with cold hexane followed by flash column chromatography using phosphate buffered silica (pH = 7, ratio of buffered silica to crude mass = 50:1, eluting with a gradient of 1:99 Et_2O:CH_2Cl_2 to 1:9 Et_2O:CH_2Cl_2). The resulting red oil was triturated with cold hexane.

Yield: 23.0 mg, 0.0558 mmol, 63%;

Appearance: Red solid;

R*f*: 0.29 (2:1 EtOAc:hexane);

^{1}H NMR (900 MHz, $CDCl_3$): δ 9.18 (s, 1H), 7.33 (d, *J* = 8.0 Hz, 1H), 7.26 – 7.23 (m, 3H), 7.14 – 7.10 (m, 3H), 7.08 (d, *J* = 7.8 Hz, 1H), 6.86 (s, 1H), 3.92 (s, 3H), 3.89 (s, 3H), 2.66 (br s, 4H), 1.79 (br s, 4H) ppm;

^{13}C NMR (225 MHz, $CDCl_3$): δ 178.3, 160.3, 159.5, 144.6, 142.6, 137.1, 136.4, 132.7, 131.2, 130.4, 130.1, 123.1, 121.3, 121.1, 117.9, 115.5, 110.3, 110.1, 55.8, 33.7, 25.0, 23.2, 21.6, 21.4 ppm;

IR: 3420, 2913, 1640, 1612, 1497, 1453, 1345, 1162, 1017, 969, 726 cm^{-1};

HRMS (ESI): calculated for $[C_{26}H_{24}N_2O_3+Na]^+$: 435.1685, found: 435.1680.

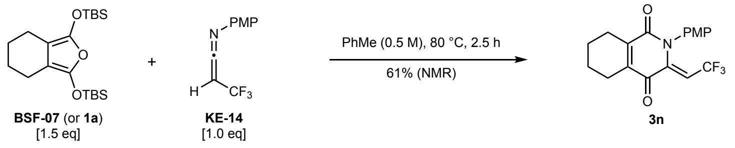

Prepared according to the general procedure.

Purification: Crude residue purified by flash column chromatography using phosphate buffered silica (pH = 7, ratio of buffered silica to crude mass = 100:1, eluting with a gradient of 1:20 hexane:EtOAc to 1:10 hexane:EtOAc). The resulting yellow oil was triturated with cold hexane.

Crude Yield: 0.136 mmol, 61% (NMR yield, determined using CH_2Br_2 as internal standard);

Appearance: Yellow solid;

R*f*: 0.28 (1:4 EtOAc:hexane);

^{1}H NMR [600 MHz, $(CD_3)_2CO$]: δ 7.27 (d, *J* = 9.0 Hz, 2H), 6.97 (d, *J* = 9.0 Hz, 2H), 5.90 (q, *J* = 10.5 Hz, 1H), 3.83 (s, 3H), 2.54 (m, 2H), 2.47 (m, 2H), 1.76 – 1.71 (m, 4H) ppm;

^{13}C NMR [150 MHz, $(CD_3)_2CO$]: δ 180.6, 161.4, 160.6, 145.5, 143.7, 142.1 (q, *J* = 5.9 Hz), 131.9, 131.3 (d, *J* = 2.1 Hz), 124.2 (q, *J* = 267.9 Hz), 114.5, 103.3 (q, *J* = 38.8 Hz), 55.8, 25.6, 23.3, 21.8, 21.4 ppm;

^{19}F NMR (377 MHz, $CDCl_3$): δ −53.91 (d, *J* = 10.2 Hz) ppm;

IR: 2944, 2862, 1689, 1680, 1613, 1512, 1427, 1230, 1257 cm^{-1};

HRMS (ESI): calculated for $[C_{18}H_{16}F_3NO_3+H]^+$: 352.1161, found: 352.1159.

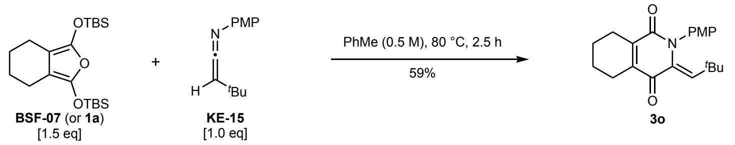

Prepared according to the general procedure.

Purification: Crude residue purified by flash column chromatography using phosphate buffered silica (pH = 7, ratio of buffered silica to crude mass = 100:1, eluting with hexane for one column volume, then eluting with 7:93 EtOAc:hexane).

Yield: 150 mg, 0.442 mmol, 59%;

Appearance: Yellow solid;

R*f*: 0.33 (1:4 EtOAc:hexane);

^{1}H NMR (900 MHz, $CDCl_3$): δ 7.29 (d, *J* = 8.5 Hz, 2H), 6.88 (d, *J* = 8.4 Hz, 2H), 6.13 (s, 1H), 3.81 (s, 3H), 2.57 (br s, 2H), 2.47 (br s, 2H), 1.74 – 1.70 (m, 4H), 0.79 (s, 9H) ppm;

^{13}C NMR (225 MHz, $CDCl_3$): δ 185.2, 161.7, 158.4, 144.6, 143.8, 139.9, 136.5, 134.7, 129.4, 113.6, 55.6, 33.0, 29.7, 25.1, 22.9, 21.5, 21.0 ppm;

IR: 2938, 2864, 1769, 1666, 1641, 1605, 1512, 1462, 1317, 1287, 1250, 1178, 1034, 832, 728 cm^{-1};

HRMS (ESI): calculated for $[C_{21}H_{25}NO_3+H]^+$: 340.1913, found: 340.1908.

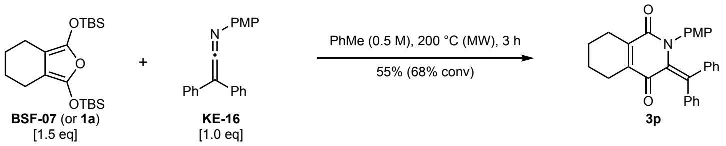

BSF-07 (or **1a**) [1.5 eq] + **KE-16** [1.0 eq] → **3p**

Slight modification of the general procedure: Reaction run in a microwave vessel in the microwave at 200 °C for 3 hours. Conversion determined by NMR analysis of the crude using dibromomethane as internal standard. Attempts to run the reaction for longer resulted in poorer yields.

Purification: Crude residue purified by flash column chromatography (ratio of silica to crude mass = 100:1, eluting with 1:99 Et_2O:CH_2Cl_2).

Yield: 80.0 mg, 0.180 mmol, 55%;

Appearance: Orange solid;

R*f*: 0.27 (CH_2Cl_2);

^{1}H NMR (400 MHz, $CDCl_3$): δ 7.34 – 7.29 (m, 3H), 7.19 (d, *J* = 6.6 Hz, 2H), 7.10 – 7.01 (m, 3H), 6.82 (d, *J* = 8.2 Hz, 2H), 6.71 (d, *J* = 8.0 Hz, 2H), 6.51 (d, *J* = 8.1 Hz, 2H), 3.68 (s, 3H), 2.62 (br s, 2H), 2.42 (br s, 2H), 1.76 – 1.58 (m, 4H) ppm;

^{13}C NMR (100 MHz, $CDCl_3$): δ 180.7, 161.2, 157.3, 144.8, 143.6, 143.1, 142.0, 139.9, 136.2, 134.1, 131.2, 129.8, 129.0, 128.8, 128.1, 128.0, 127.5, 113.3, 55.5, 24.9, 23.0, 21.5, 21.2 ppm;

IR: 3058, 2939, 1659, 1632, 1512, 1443, 1392, 1293, 1248 cm^{-1};

HRMS (ESI): calculated for $[C_{29}H_{25}NO_3+H]^+$: 436.1913, found: 436.1910.

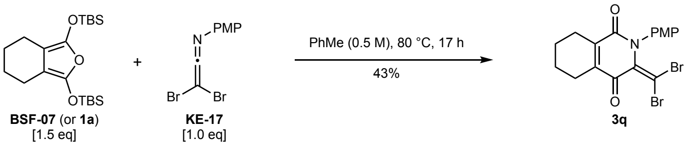

BSF-07 (or **1a**) [1.5 eq] + **KE-17** [1.0 eq] → **3q**

Slight modification of the general procedure: Reaction run for 17 hours.

Purification: Crude residue purified by flash column chromatography using phosphate buffered silica (pH = 7, ratio of silica to crude mass = 100:1, eluting with a gradient of 1:9 EtOAc:hexane to 3:17 EtOAc:hexane).

Yield: 32.0 mg, 0.0725 mmol, 43%;

Appearance: Yellow solid;

R*f*: 0.24 (3:17 EtOAc:hexane);

^{1}H NMR (600 MHz, $CDCl_3$): δ 7.20 (d, *J* = 8.9 Hz, 2H), 6.89 (d, *J* = 9.1 Hz, 2H), 3.81 (s, 3H), 2.57 (d, *J* = 5.9 Hz, 2H), 2.52 (d, *J* = 5.9 Hz, 2H), 1.77 – 1.70 (m, 4H) ppm;

^{13}C NMR (225 MHz, $CDCl_3$): δ 182.5, 160.5, 158.3, 143.6, 143.2, 138.7, 131.8, 129.0, 113.9, 93.1, 55.6, 24.8, 22.8, 21.3, 20.9 ppm;

IR: 2936, 2350, 1678, 1511, 1503, 1282, 1250 cm^{-1};

HRMS (ESI): calculated for [$C_{17}H_{15}Br_2NO_3$+H]$^+$: 439.9491, found: 439.9491.

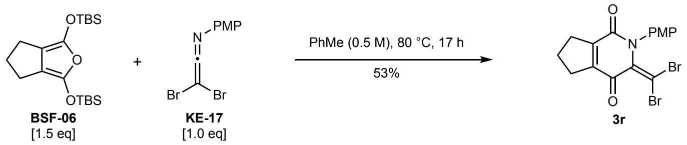

Slight modification of the general procedure: Reaction run for 17 hours.

Purification: Crude residue purified by flash column chromatography using phosphate buffered silica (pH = 7, ratio of buffered silica to crude mass = 100:1, eluting with a gradient of hexane to 3:17 EtOAc:hexane).

Yield: 34.0 mg, 0.0796 mmol, 53%;

Appearance: Yellow solid;

R*f*: 0.20 (3:17 EtOAc:hexane);

^{1}H NMR (600 MHz, $CDCl_3$): δ 7.21 (d, *J* = 8.9 Hz, 2H), 6.89 (d, *J* = 9.0 Hz, 2H), 3.81 (s, 3H), 2.91 (q, *J* = 7.6 Hz, 4H), 2.11 (p, *J* = 7.7 Hz, 2H) ppm;

^{13}C NMR (150 MHz, $CDCl_3$): δ 180.4, 159.2, 158.3, 150.4, 149.1, 140.3, 132.1, 129.0, 113.9, 94.9, 55.6, 32.6, 31.0, 21.8 ppm;

IR: 2955, 2838, 1678, 1631, 1511, 1502, 1322, 1281, 1250, 1190 cm^{-1};

HRMS (ESI): calculated for [$C_{16}H_{13}Br_2NO_3$+H]$^+$: 425.9335, found: 425.9341.

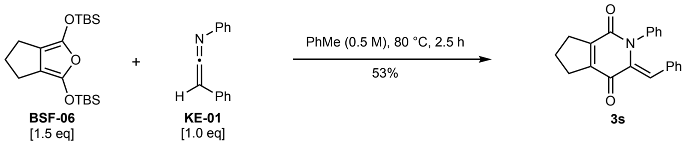

Prepared according to the general procedure.

Purification: Crude residue purified via trituration with cold hexane.

Yield: 60 mg, 0.190 mmol, 53%;

Appearance: Yellow solid;

R*f*: 0.28 (1:99 Et_2O:CH_2Cl_2);

^{1}H NMR [400 MHz, $(CH_3)_2CO$]: δ 7.18 (t, *J* = 4.3 Hz, 3H), 6.99 (m, 7H), 6.91 (t, *J* = 7.4 Hz, 1H), 2.90 (q, *J* = 8.0 Hz, 4H), 2.13 (q, *J* = 7.7 Hz, 2H) ppm;

^{13}C NMR [100 MHz, $(CH_3)_2CO$]: δ 180.4, 159.8, 151.7, 149.9, 139.3, 138.1, 134.7, 130.0, 129.5, 128.5, 128.1, 127.9, 127.2, 123.5, 33.4, 31.4, 22.2 ppm;

IR: 3052, 2951, 1666, 1631, 1592, 1584, 1572, 1492, 1330, 1288, 1161 cm^{-1};

HRMS (ESI): calculated for $[C_{21}H_{17}NO_2+H]^+$: 316.1332, found: 316.1333.

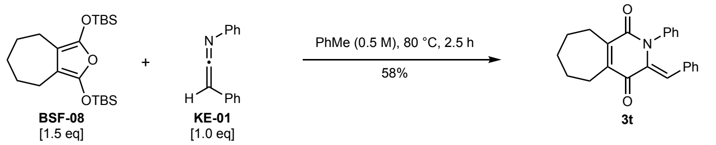

Prepared according to the general procedure.

Purification: Crude residue purified by flash column chromatography using phosphate buffered silica (pH = 7, ratio of buffered silica to crude mass = 50:1, eluting with a gradient of 1:20 Et_2O:hexane to 1:5 Et_2O:hexane). The resulting yellow oil was dissolved in hexane and transferred to a freezer (−20 °C) to sit overnight, allowing a solid to crash out. Filtration (washing with cold hexane) yielded **3t**.

Yield: 219 mg, 0.638 mmol, 58%;

Appearance: Yellow solid;

R*f*: 0.37 (5:1 hexane:EtOAc);

^{1}H NMR (600 MHz, $CDCl_3$): δ 7.25 (s, 1H), 7.09 (d, *J* = 7.8 Hz, 2H), 7.01 (t, *J* = 7.7 Hz, 2H), 6.97 – 6.91 (m, 4H), 6.87 (d, *J* = 7.3 Hz, 2H), 2.95 – 2.92 (m, 2H), 2.87 – 2.83 (m, 2H), 1.93 – 1.87 (m, 2H), 1.69 – 1.62 (m, 4H) ppm;

^{13}C NMR (150 MHz, $CDCl_3$): δ 182.0, 160.9, 149.6, 148.6, 138.1, 135.5, 133.7, 129.2, 128.2, 128.1, 127.6, 127.3, 126.9, 123.9, 32.3, 28.6, 25.7, 25.4, 25.3 ppm;

IR: 3059, 2924, 2853, 1656, 1594, 1492, 1446, 1292, 1215, 977, 760 cm^{-1};

HRMS (ESI): calculated for $[C_{23}H_{21}NO_2M+H]^+$: 344.1651, found: 344.1646.

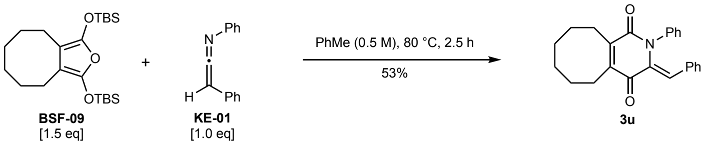

Prepared according to the general procedure.

Purification: Crude residue purified by flash column chromatography using phosphate buffered silica (pH = 7, ratio of buffered silica to crude mass = 100:1, eluting with a gradient of hexane to 5:95 EtOAc:Hexane).

Yield: 81.0 mg, 0.230 mmol, 53%;

Appearance: Yellow solid;

R*f*: 0.28 (9:1 hexane:EtOAc);

^{1}H NMR (600 MHz, CD_2Cl_2): δ 7.20 (s, 1H), 7.11 (d, *J* = 8.1 Hz, 2H), 7.02 (t, *J* = 7.8 Hz, 2H), 6.99 – 6.91 (m, 4H), 6.88 (d, *J* = 7.3 Hz, 2H), 2.78 (app dt, *J* = 43.4, 6.3 Hz, 4H), 1.76 (app d, *J* = 24.5 Hz, 4H), 1.52 (br s, 4H) ppm;

^{13}C NMR (100 MHz, CD_2Cl_2): δ 181.9, 160.6, 147.5, 146.8, 138.6, 135.9, 134.3, 129.4, 128.8, 128.2, 127.6, 127.5, 127.0, 123.2, 30.4, 30.0, 27.8, 27.1, 26.9, 25.6 ppm;

IR: 3054, 2924, 2857, 1716, 1660, 1586, 1491, 1448, 1384, 1292, 1172, 912, 746, 694 cm^{-1};

HRMS (ESI): calculated for $[C_{24}H_{23}NO_2+H]^+$: 358.1802, found: 358.1804.

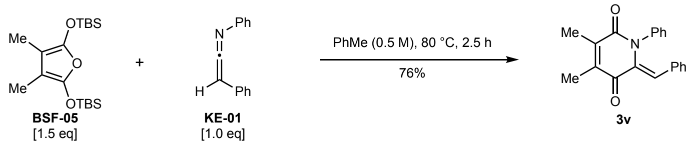

Prepared according to the general procedure.

Purification: Crude residue purified by flash column chromatography using phosphate buffered silica (pH = 7, ratio of buffered silica to crude mass = 50:1, eluting with a gradient of hexane to 1:9 EtOAc:hexane). The resulting yellow oil was filtered over a phosphate buffered silica plug (pH = 7, ratio of buffered silica to crude mass = 10:1, eluting with CH_2Cl_2 then 9:1 CH_2Cl_2:Et_2O).

Yield: 41.0 mg, 0.135 mmol, 76%;

Appearance: Yellow solid;

R*f*: 0.48 (2:1 hexane:EtOAc);

^{1}H NMR (900 MHz, $CDCl_3$): δ 7.28 (s, 1H), 7.08 (d, *J* = 7.3 Hz, 2H), 7.01 (t, *J* = 7.9 Hz, 2H), 6.96 (t, *J* = 7.2 Hz, 1H), 6.95 – 6.91 (m, 3H), 6.85 (d, *J* = 7.5 Hz, 2H), 2.26 (s, 3H), 2.17 (s, 3H) ppm;

^{13}C NMR (225 MHz, $CDCl_3$): δ 182.3, 160.9, 143.3, 142.6, 138.0, 135.0, 133.7, 129.1, 128.3, 128.2, 127.5, 127.3, 127.0, 124.0, 15.2, 12.9 ppm;

IR: 3059, 2924, 2853, 1666, 1594, 1492, 1446, 1321, 1292, 1215, 977, 760 cm^{-1};

HRMS (ESI): calculated for $[C_{20}H_{17}NO_2+H]^+$: 304.1338, found: 304.1333.

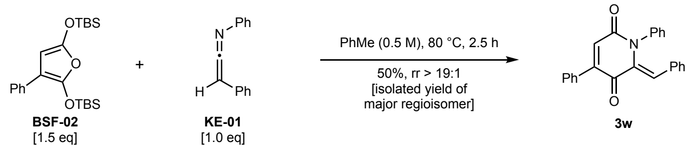

Prepared according to the general procedure.

Purification: Crude residue purified via trituration with cold hexane.

Yield: 194 mg, 0.552 mmol, 50%;

Appearance: Dark orange solid;

R*f*: 0.5 (1:99 Et_2O:CH_2Cl_2);

^{1}H NMR (400 MHz, $CDCl_3$): δ 7.66 – 7.63 (m, 2H), 7.50 (br s, 3H), 7.35 (s, 1H), 7.19 – 7.16 (m, 3H), 7.06 (t, *J* = 7.5 Hz, 2H), 7.02 – 6.88 (m, 6H) ppm;

^{13}C NMR (100 MHz, $CDCl_3$): δ 181.8, 160.1, 147.6, 137.4, 135.9, 134.4, 133.4, 132.6, 130.3, 129.2, 129.1, 128.7, 128.3, 128.1, 127.9, 127.4, 127.2, 125.7 ppm;

IR: 3728, 2345, 2267, 1666, 1597, 1490, 1452, 1361, 1235 cm^{-1};

HRMS (ESI): calculated for $[C_{24}H_{17}NO_2+H]^+$: 352.1333, found: 352.1332.

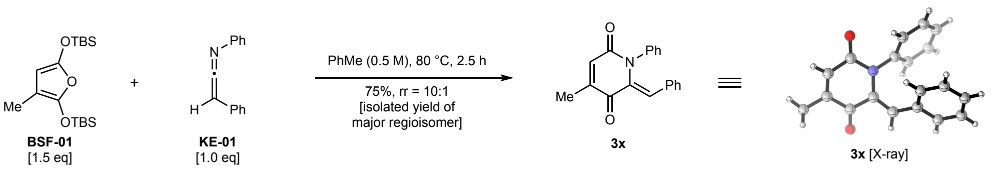

Prepared according to the general procedure.

Purification: Crude residue purified by flash column chromatography (ratio of silica to crude mass = 100:1, eluting with 1:99 Et_2O:CH_2Cl_2). A small quantity of pure **3x** was crystallized via the slow evaporation of acetone to yield yellow needle-like crystals suitable for X-ray analysis.

Yield: 94.0 mg, 0.330 mmol, 75%;

Appearance: Yellow solid;

R*f*: 0.36 (1:99 Et_2O:CH_2Cl_2);

M.p.: 118 – 122 °C;

^{1}H NMR (400 MHz, $CDCl_3$): δ 7.30 (s, 1H), 7.08 (d, *J* = 7.9 Hz, 2H), 7.02 (t, *J* = 7.7 Hz, 2H), 6.99 – 6.90 (m, 5H), 6.85 (d, *J* = 7.3 Hz, 2H), 2.18 (s, 3H) ppm;

^{13}C NMR (100 MHz, $CDCl_3$): δ 182.7, 159.9, 147.4, 137.5, 135.0, 134.9, 133.5, 129.1, 128.2, 128.2, 127.7, 127.4, 127.1, 125.1, 15.9 ppm;

IR: 1666, 1639, 1632, 1582, 1492, 1444, 1333, 1307 cm^{-1};

HRMS (ESI): calculated for $[C_{19}H_{15}O_2N+H]^+$: 290.1175, found: 290.1169.

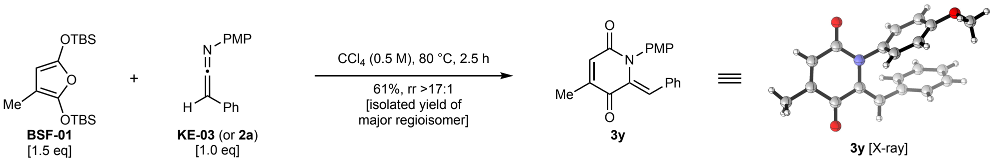

Slight modification of the general procedure: anhydrous CCl_4 rather than toluene as solvent.

Purification: Crude residue purified via trituration with cold hexane. A small quantity of pure **3y** was crystallized via the slow evaporation of EtOAc to yield yellow needle-like crystals suitable for X-ray analysis.

Yield: 35.0, 0.110 mmol, 61%;

Appearance: Yellow solid;

R*f*: 0.40 (2:3 EtOAc:hexane);

M.p.: 137 – 142 °C;

^{1}H NMR (400 MHz, $CDCl_3$): δ 7.30 (s, 1H), 7.02 – 6.92 (m, 6H), 6.83 (d, J = 6.9 Hz, 2H), 6.52 (d, J = 8.8 Hz, 2H), 3.64 (s, 3H), 2.17 (s, 3H) ppm;

^{13}C NMR (100 MHz, $CDCl_3$): δ 182.6, 160.0, 158.3, 147.3, 135.2, 134.9, 133.7, 130.4, 129.2, 129.0, 127.5, 127.3, 124.7, 113.6, 55.5, 15.9 ppm;

IR: 3056, 2922, 2836, 1678, 1632, 1511, 1330, 1299, 1250, 1032, 977 cm^{-1};

HRMS (ESI): calculated for $[C_{20}H_{17}O_3N+H]^+$: 320.1281, found: 320.1283.

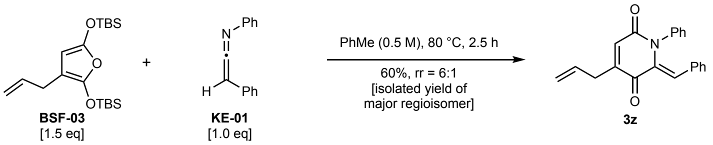

Prepared according to the general procedure.

Purification: Crude residue purified by flash column chromatography using phosphate buffered silica (pH = 7, ratio of buffered silica to crude mass = 100:1, eluting with 1:9 EtOAc:hexane).

Yield: 76.0 mg, 0.240 mmol, 60%;

Appearance: Yellow solid;

R*f*: 0.19 (1:9 EtOAc:hexane);

^{1}H NMR (600 MHz, $CDCl_3$): δ 7.31 (s, 1H), 7.09 (d, J = 7.8 Hz, 2H), 7.02 (t, J = 7.8 Hz, 2H), 6.97 (app t, J = 7.3 Hz, 1H), 6.94 (t, J = 7.5 Hz, 3H), 6.91 (s, 1H), 6.85 (d, J = 7.5 Hz, 2H), 5.97 – 5.87 (m, 1H), 5.30 – 5.24 (m, 2H), 3.32 (d, J = 7.0 Hz, 2H) ppm;

^{13}C NMR (150 MHz, $CDCl_3$): δ 182.0, 159.9, 149.2, 137.5, 135.1, 134.5, 133.5, 132.7, 129.1, 128.2, 128.2, 127.7, 127.4, 127.1, 125.1, 119.3, 33.1 ppm;

IR: 3059, 1673, 1631, 1593, 1493, 1390, 1333, 1305, 1239 cm^{-1};

HRMS (ESI): calculated for $[C_{21}H_{17}NO_2+H]^+$: 316.1332, found: 316.1330.

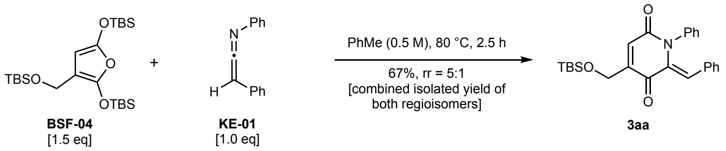

Prepared according to the general procedure.

Purification: Crude residue purified by flash column chromatography using phosphate buffered silica (pH = 7, ratio of buffered silica to crude mass = 100:1, eluting with 5:95 EtOAc:hexane).

Yield: 83.0 mg, 0.200 mmol, 67%;

Appearance: Yellow solid;

R*f*: 0.16 (5:95 EtOAc:hexane);

^{1}H NMR (600 MHz, $CDCl_3$, only major isomer indicated): δ 7.29 (s, 1H), 7.16 (t, *J* = 2.2 Hz, 1H), 7.08 (t, *J* = 7.1 Hz, 2H), 7.03 (t, *J* = 7.8 Hz, 2H), 7.00 – 6.96 (m, 1H), 6.94 (t, *J* = 7.5 Hz, 3H), 6.84 (d, *J* = 7.9 Hz, 2H), 4.67 (d, *J* = 2.3 Hz, 2H), 0.96 (s, 9H), 0.15 (s, 6H) ppm;

^{13}C NMR (100 MHz, $CDCl_3$, only major isomer indicated): δ 181.7, 160.2, 149.8, 137.6, 135.1, 133.5, 132.6, 129.1, 129.1, 128.3, 127.8, 127.4, 127.2, 124.7, 59.5, 26.1, 18.5, –5.3 ppm;

IR: 3061, 2954, 2929, 2885, 2856, 1687, 1672, 1640, 1585, 1493, 1445, 1325, 1301, 1255, 1201, 1127 cm^{-1};

HRMS (ESI): calculated for $[C_{25}H_{29}NO_3Si+H]^+$: 420.1989, found: 420.1994.

Direct Diels–Alder Cycloadducts (i.e., Non-Ring-Opened)

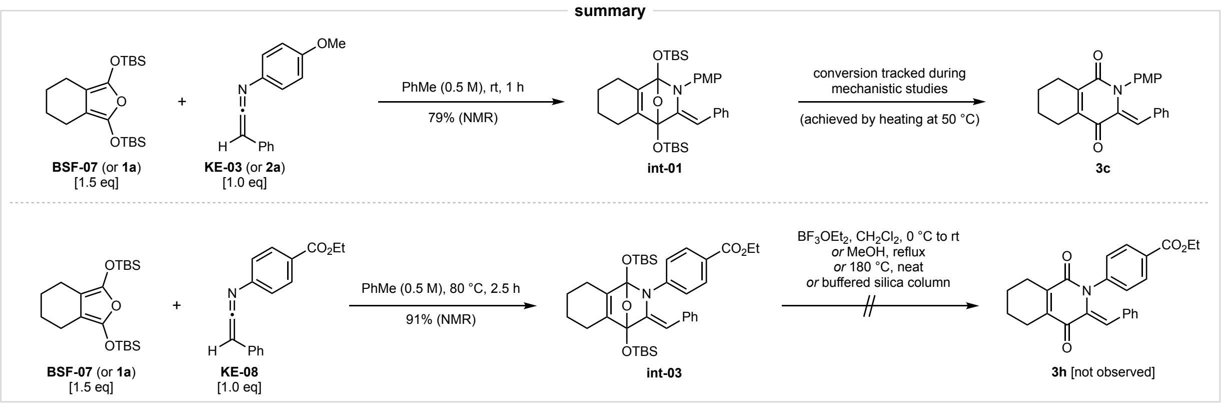

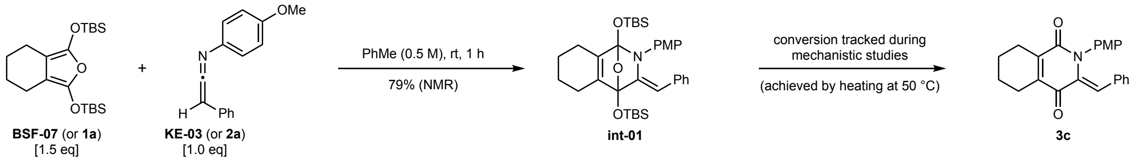

Given the propensity of silyloxyfurans and ketenimines to decompose if not appropriately handled (see notes concerning stability and storage in their respective preparation sections earlier in this document), directly before the Diels–Alder reaction the purity of both was determined by ^{1}H NMR using 1,2-dibromomethane as internal standard. To minimize confusion, the masses of silyloxyfurans and ketenimines are omitted from this procedure. To a flame-dried one-neck round-bottom flask under argon and equipped with a stir bar was added **KE-03** (0.310 mmol, 1.00 equiv), anhydrous toluene (0.621 mL, 0.50 M), and **BSF-07** (0.466 mmol, 1.50 equiv). The reaction was stirred at ambient temperature for 1 hour and concentrated under reduced pressure to afford crude **int-01**. Attempts to further purify were unsuccessful (product prone to facile ring-opening and/or competing decomposition pathways).

Crude Yield: 0.245 mmol, 79% (NMR yield, determined using dimethyl terephthalate as internal standard);

Appearance: Yellow oil;

R*f*: N/A (decomposes on glass-backed silica TLC plates);

HRMS (ESI): calculated for $[C_{35}H_{51}NO_4Si_2+H]^+$: 606.3435, found: 606.3443;

^{1}H NMR (600 MHz, $CDCl_3$, 3–8 ppm region only): δ 7.06 – 6.76 (m, 7H), 6.57 (app d, *J* = 8.4 Hz, 2H), 5.90 (s, 1H), 3.69 (s, 3H) ppm. While the crude ^{1}H NMR spectrum of **int-01** is highly complex in the aliphatic region because of unreacted furan and/or various decomposition products, the region from 3–8 ppm is relatively clean (see below). During our mechanistic studies (described later in this document), we observed clean conversion of **int-01** into **3c** upon heating in CCl_4 at 50 °C. Attempts to obtain additional characterization data for **int-01** were unsuccessful.

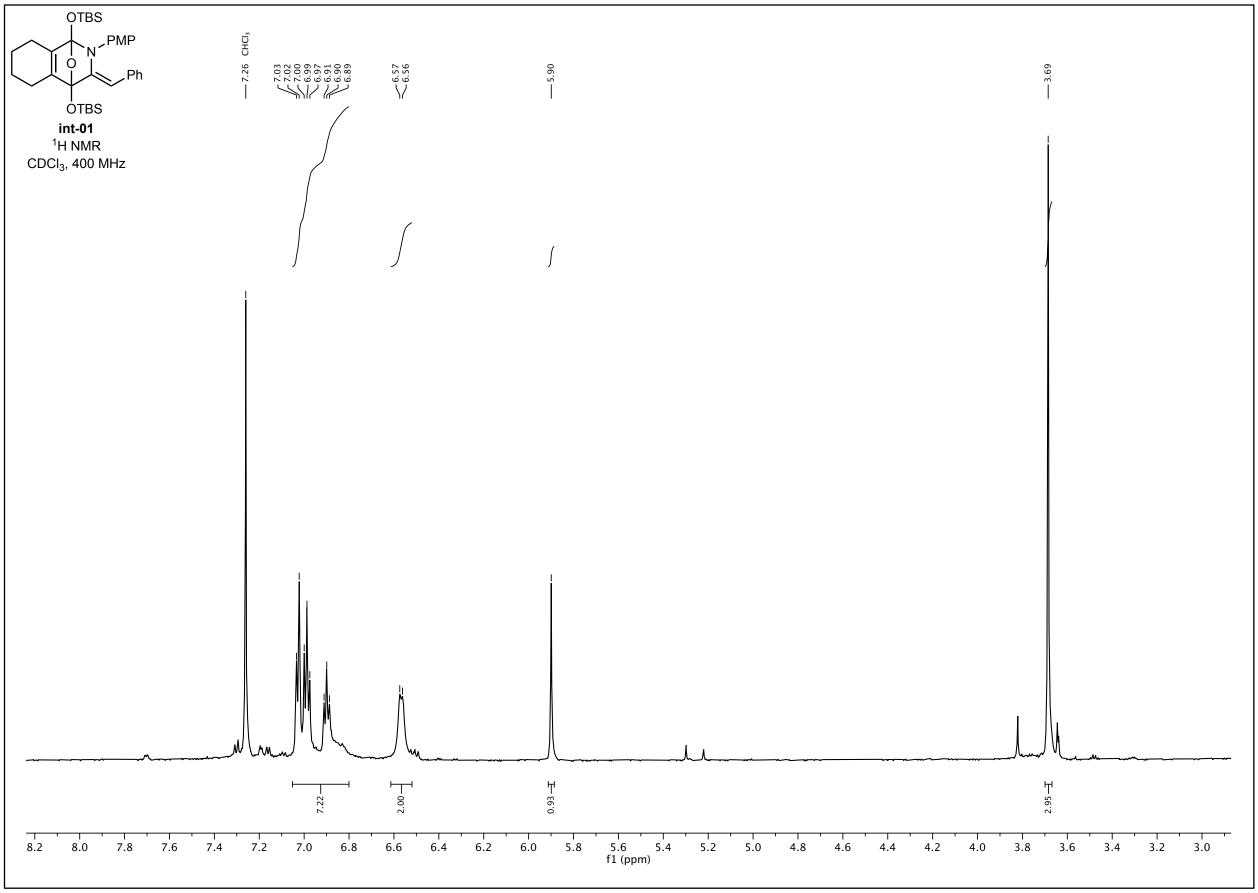

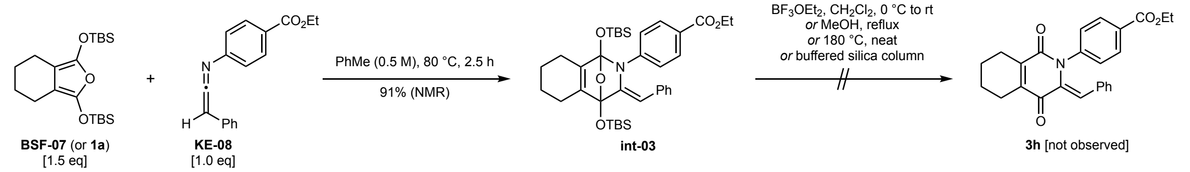

Given the propensity of silyloxyfurans and ketenimines to decompose if not appropriately handled (see notes concerning stability and storage in their respective preparation sections earlier in this document), directly before the Diels–Alder reaction the purity of both was determined by ^{1}H NMR using 1,2-dibromomethane as internal standard. To minimize confusion, the masses of silyloxyfurans and ketenimines are omitted from this procedure. To a flame-dried one-neck round-bottom flask under argon and equipped with a stir bar was added **KE-08** (0.550 mmol, 1.00 equiv), anhydrous toluene (1.10 mL, 0.50 M), and **BSF-07** (1.37 mmol, 1.50 equiv). The reaction was warmed on a heating mantle at 80 °C for 2.5 hours, cooled to ambient temperature, and concentrated under reduced pressure. Kugelrohr distillation (180 °C at 10 torr) was employed to remove volatile impurities, affording crude **int-03**. All attempts to purify further, or ring-open, were unsuccessful (product prone to decomposition). **Int-03** appears significantly more thermally stable than **int-01**.

Crude Yield: 0.483 mmol, 91% (NMR yield, determined using 1,2-dichloroethane as internal standard);

Appearance: Yellow oil;

R*f*: N/A (decomposes on glass-backed silica TLC plates);

HRMS (ESI): calculated for $[C_{37}H_{53}NO_5Si_2+H]^+$: 648.3541, found: 648.3537;

^{1}H NMR (600 MHz, $CDCl_3$, 3–8 ppm region only): δ 7.65 (d, J = 8.2 Hz, 2H), 6.92 – 6.87 (m, 4H), 6.86 – 6.80 (m, 3H), 5.74 (s, 1H), 4.27 (q, J = 6.7 Hz, 2H) ppm. While the crude ^{1}H NMR spectrum of **int-03** is highly complex in the aliphatic region because of unreacted furan and/or various decomposition products, the region from 3–8 ppm is relatively clean (insert below). Attempts to obtain additional characterization data for **int-03** were unsuccessful.

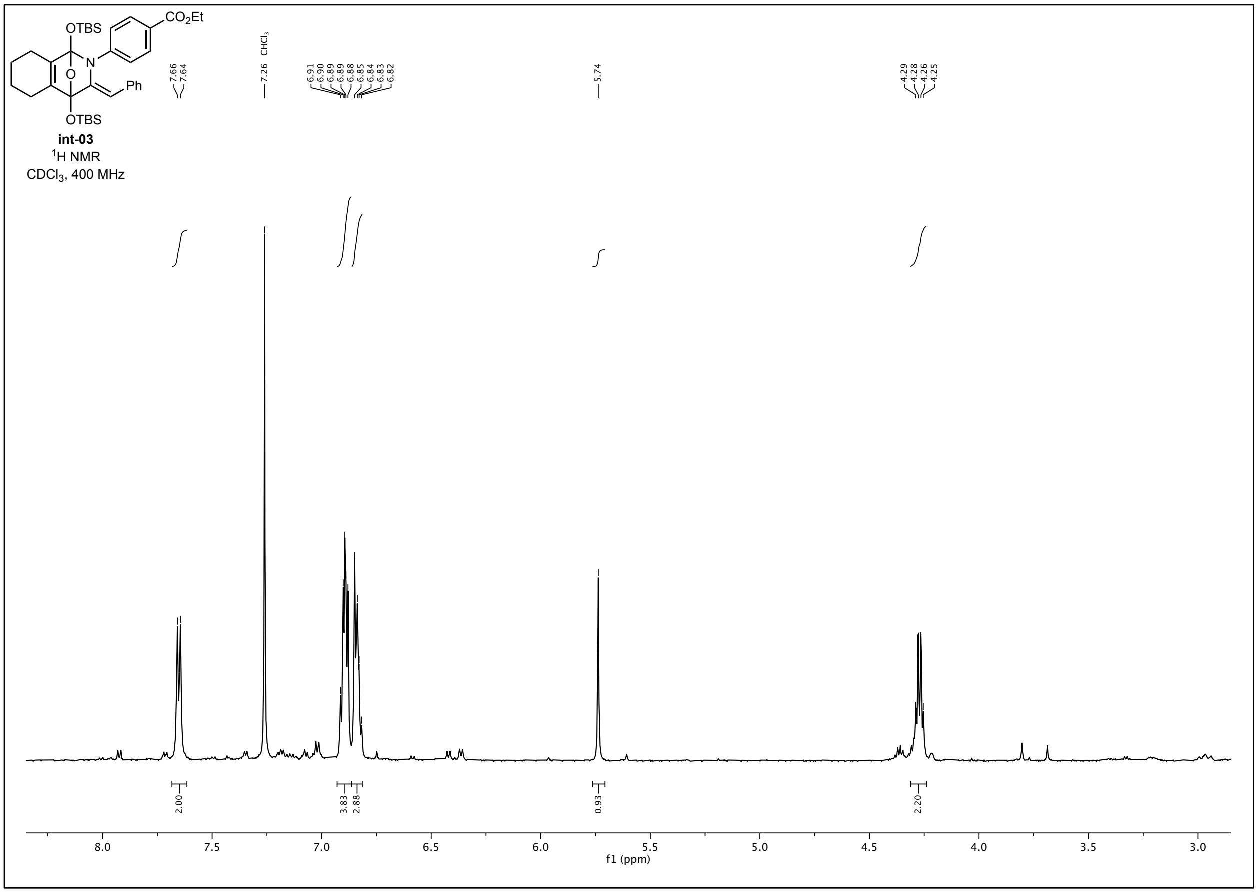

Unsuccessful Diels–Alder Reactions

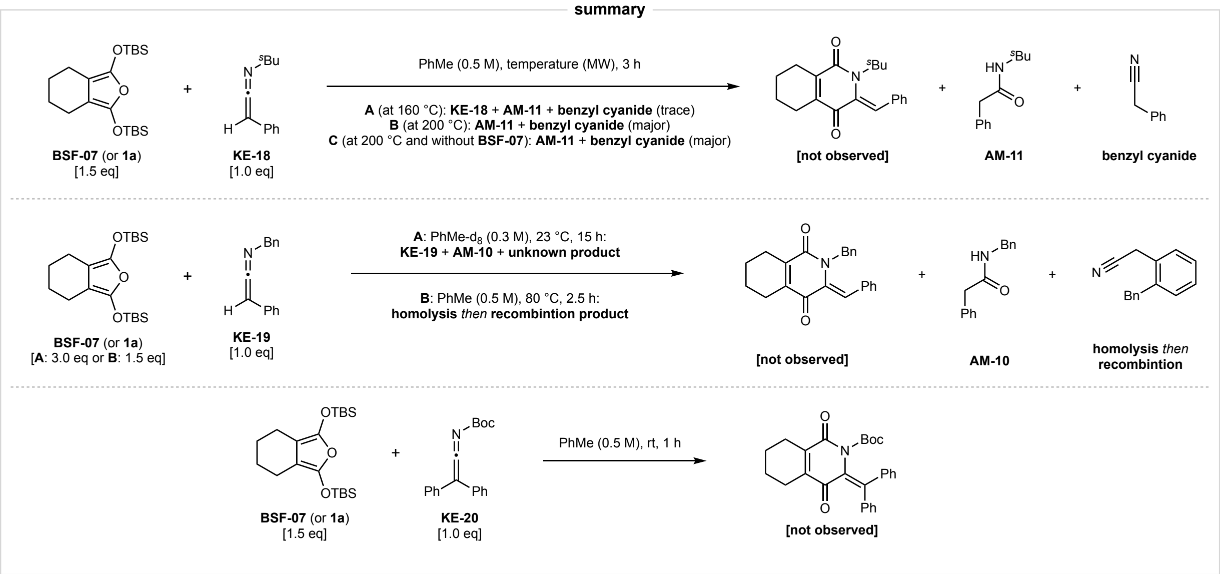

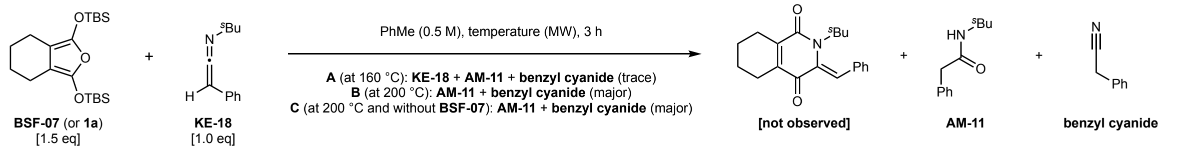

No evidence for Diels–Alder reactivity was observed when **KE-18** was reacted with furan **BSF-07** across a range of temperatures. Rather, complex mixtures were observed. Aside from unreacted starting material, compounds that could be identified in crude mixtures included **AM-11** and what appears to be benzyl cyanide.

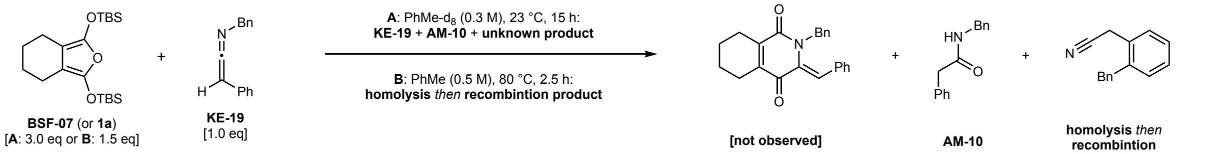

No evidence of Diels–Alder reactivity was observed when **KE-19** was reacted with furan **BSF-07** across a range of temperatures. Rather, **KE-19** undergoes a homolysis/recombination process (slow at room temperature, fast at 80 °C).

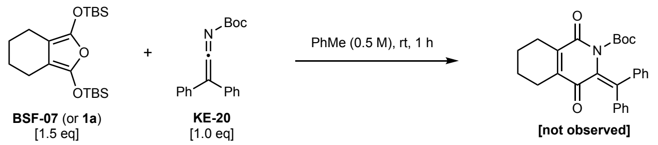

No evidence of Diels–Alder reactivity was observed when **KE-20** was reacted with furan **BSF-07** at ambient temperature (complete consumption of ketenimine was observed, resulting in a complex crude mixture).

Derivatization Studies

Allylic Transposition for the Synthesis of 2-Pyridones

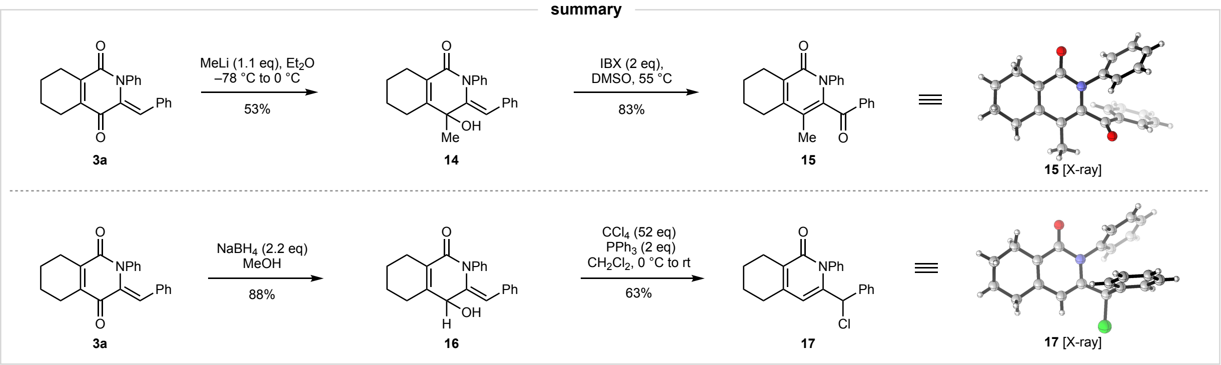

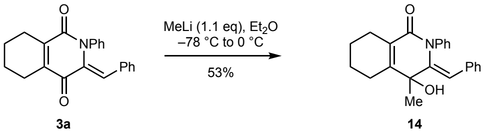

To a flame-dried 25 mL one-neck round-bottom flask under argon and equipped with a stir bar was added **3a** (52.0 mg, 0.158 mmol, 1.00 equiv) and anhydrous Et_2O (5.64 mL, 0.028 M). The round-bottom flask was submerged in a dry ice/acetone bath and a solution of freshly titrated[58] methyl lithium (0.88 M in Et_2O, 0.200 mL, 0.176 mmol, 1.10 equiv) was added dropwise via syringe. The dry ice/acetone bath was replaced with an ice/water bath, and the reaction was allowed to stir for two hours before being quenched by the addition of saturated aqueous ammonium chloride. The ice/water bath was removed, and the reaction was allowed to warm to ambient temperature. The crude mixture was diluted with EtOAc, the layers separated, and the aqueous layer was extracted with EtOAc (×3). The combined organic layers were washed with brine, dried over Na_2SO_4, filtered, and concentrated under reduced pressure. Purification via flash column chromatography using phosphate buffered silica (pH = 7, ratio of buffered silica to crude mass = 50:1, eluting with 1:99 Et_2O:CH_2Cl_2) yielded **14**.

Yield: 29.0 mg, 0.0839 mmol, 53%;

Appearance: Pale orange solid;

R*f*: 0.40 (1:99 Et_2O:CH_2Cl_2);

^{1}H NMR (600 MHz, $CDCl_3$): δ 7.16 (d, J = 8.4 Hz, 2H), 7.02 – 6.88 (m, 7H), 6.85 (t, J = 7.3 Hz, 1H), 6.45 (s, 1H), 2.56 – 2.47 (m, 1H), 2.47 – 2.25 (m, 3H), 2.04 (s, 1H), 1.87 – 1.76 (m, 2H), 1.69 (s, 3H), 1.65 – 1.58 (m, 2H) ppm;

^{13}C NMR (150 MHz, $CDCl_3$): δ 163.0, 151.8, 144.6, 139.2, 135.0, 129.1, 127.6, 127.4, 126.6, 126.4, 126.3, 125.5, 113.2, 73.4, 26.7, 23.9, 23.5, 22.1, 22.0 ppm;

IR: 3402, 2937, 2429, 2230, 1940, 1834, 1725, 1631, 1598, 1494, 1383, 1302 cm^{-1};

HRMS (ESI): calculated for $[C_{23}H_{23}NO_2+H]^+$: 346.1801, found: 346.1790.

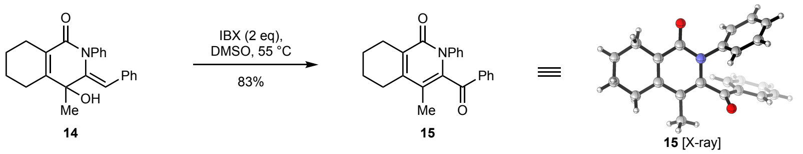

Prepared according to a modification of the procedure reported by Iwabuchi.[59] To a flame-dried 10 mL three-neck round-bottom flask under argon and equipped with a stir bar was added **14** (23.0 mg, 0.0666 mmol, 1.00 equiv), anhydrous DMSO (0.705 mL, 0.95 M), and IBX (37.0 mg, 0.132 mmol, 2.00 equiv). The reaction flask was warmed on a heating mantle at 55 °C for 4 hours by which time TLC indicated the complete consumption of starting material. The flask was removed from the heating mantle and allowed to cool to ambient temperature. The reaction was diluted with water, extracted with Et_2O (×3), and the combined organics were washed with brine, dried over $MgSO_4$, filtered, and concentrated under reduced pressure. Purification via flash column chromatography (ratio of silica to crude mass = 50:1, eluting with 5:95 Et_2O:CH_2Cl_2) yielded **15**. A small quantity of pure **15** was crystallized via the slow evaporation of EtOAc:hexane to yield white crystals suitable for X-ray analysis.

Yield: 19.0 mg, 0.0553 mmol, 83%;

Appearance: Off-white solid;

R*f*: 0.20 (5:95 Et_2O:CH_2Cl_2);

M.p.: 151 – 154 °C

^{1}H NMR (600 MHz, $CDCl_3$): δ 7.55 (d, *J* = 8.4 Hz, 2H), 7.50 (t, *J* = 7.5 Hz, 1H), 7.33 (t, *J* = 7.9 Hz, 3H), 7.15 (t, *J* = 7.5 Hz, 2H), 6.98 (br s, 1H), 6.85 (br s, 1H), 2.65 (t, *J* = 6.2 Hz, 2H), 2.59 (t, *J* = 6.2 Hz, 2H), 1.91 (s, 3H), 1.86 – 1.80 (m, 4H) ppm; Note: significant peak broadening in the aromatic region was observed, presumably as a consequence of restricted rotation on the NMR timescale.

^{13}C NMR (100 MHz, $CDCl_3$): δ 191.8, 161.4, 147.7, 138.8, 137.8, 136.8, 134.1, 129.6, 129.4, 129.1, 128.8, 128.4, 112.9, 27.2, 24.4, 22.2, 21.8, 13.8 ppm; Note: fewer carbon environments than expected were observed, and quantitative NMR experiments did not elucidate which peak(s) may be coincident. We speculate this may be a consequence of restricted rotation on the NMR timescale causing significant peak broadening, as observed by ^{1}H NMR, rather than peaks simply overlapping. VT NMR experiments were not pursued given the structure was proven by X-ray analysis.

IR: 2928, 2430, 1701, 1658, 1649, 1642, 1546, 1494, 1452, 1308, 1283, 1244, 1089 cm^{-1};

HRMS (ESI): calculated for $[C_{23}H_{21}NO_2+H]^+$: 344.1645 , found: 344.1628.

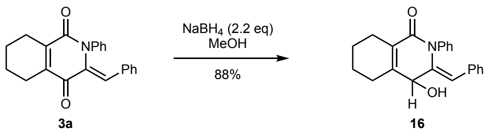

To a 25 mL one-neck round-bottom flask under argon and equipped with a stir bar was added **3a** (70.0 mg, 0.212 mmol, 1.00 equiv), MeOH (3.50 mL, 0.060 M), and $NaBH_4$ (18.0 mg, 0.476 mmol, 2.24 equiv). The reaction was allowed to stir for 3 hours by which time TLC indicated the complete consumption of starting material. The reaction was concentrated under reduced pressure, diluted with CH_2Cl_2 and saturated aqueous NH_4Cl, and extracted with CH_2Cl_2 (×3). The combined organics were washed with brine, dried over Na_2SO_4, filtered, and concentrated under reduced pressure. Purification via flash column chromatography using phosphate buffered silica (pH = 7, ratio of buffered silica to crude mass = 50:1, eluting with 1:9 Et_2O:CH_2Cl_2) yielded **16**.

Yield: 62.0 mg, 0.187 mmol, 88%;

Appearance: Off-white solid;

R*f*: 0.35 (1:9 Et_2O:CH_2Cl_2);

^{1}H NMR [600 MHz, $(CD_3)_2SO$]: δ 7.28 (d, *J* = 8.0 Hz, 2H), 7.04 (t, *J* = 7.9 Hz, 2H), 7.01 – 6.99 (m, 4H), 6.96 – 6.93 (m, 1H), 6.89 – 6.86 (t, *J* = 7.4 Hz, 1H), 6.18 (s, 1H), 5.92 (br s, 1H), 4.46 (s, 1H), 2.52 – 2.48 (m, 1H), 2.33 (q, *J* = 9.2 Hz, 2H), 2.18 (d, *J* = 17.0 Hz, 1H), 1.75 – 1.69 (m, 2H), 1.62 – 1.52 (m, 2H) ppm;

^{13}C NMR [150 MHz, $(CD_3)_2SO$]: δ 162.7, 147.4, 139.7, 139.5, 134.0, 128.8 (2 coincident peaks), 127.3 (3 coincident peaks), 127.1 (2 coincident peaks), 126.6 (2 coincident peaks), 126.5, 124.9, 117.6, 69.8, 26.9, 23.3, 21.6, 21.5 ppm; Note: quantitative ^{13}C NMR was employed to assign those peaks which correspond to multiple carbon signals.

IR: 3379, 2919, 2465, 1743, 1667, 1633, 1597, 1489, 1371, 1185, 1017, 872 cm^{-1};

HRMS (ESI): calculated for $[C_{22}H_{21}NO_2+H]^+$: 332.1645, found: 332.1631.

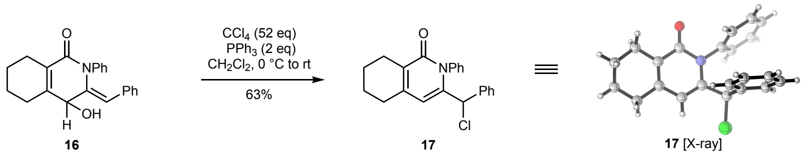

To a flame-dried 10 mL one-neck round-bottom flask under argon and equipped with a stir bar was added **16** (30.0 mg, 0.0905 mmol, 1.00 equiv), anhydrous CH_2Cl_2 (0.455 mL, 0.20 M), and PPh_3 (47.0 mg, 0.179 mmol, 1.98 equiv). The round-bottom flask was submerged in an ice/water bath and anhydrous CCl_4 (0.450 mL, 4.70 mmol, 52.0 equiv) was added dropwise. The ice/water bath was removed, and the reaction was stirred for 16 hours by which time TLC analysis indicated the complete consumption of starting material. The reaction was concentrated under reduced pressure and purified via flash column chromatography (ratio of silica to crude mass = 50:1, eluting with 5:95 Et_2O:CH_2Cl_2) to yield **17**. A small quantity of pure **17** was crystallized via the slow evaporation of CH_2Cl_2 to yield white crystals suitable for X-ray analysis.

Yield: 20.0 mg, 0.0572 mmol, 63%;

Appearance: White crystalline solid;

R*f*: 0.27 (5:95 Et_2O:CH_2Cl_2);

M.p.: 176 – 178 °C;

^{1}H NMR (400 MHz, $CDCl_3$): δ 7.53 (t, *J* = 6.9 Hz, 1H), 7.41 (t, *J* = 7.4 Hz, 1H), 7.36 (d, *J* = 8.3 Hz, 1H), 7.32 – 7.27 (m, 4H), 7.16 – 7.08 (m, 2H), 6.74 (d, *J* = 7.8 Hz, 1H), 6.29 (s, 1H), 5.59 (s, 1H), 2.63 – 2.49 (m, 4H), 1.77 (br s, 4H) ppm;

^{13}C NMR (100 MHz, $CDCl_3$): δ 163.2, 146.9, 142.9, 138.1, 137.6, 129.7, 129.4, 129.3, 129.0, 129.0, 128.8, 128.8 (2 coincident peaks), 127.9, 127.8 (2 coincident peaks), 109.5, 58.8, 29.6, 23.8, 22.2, 22.1 ppm; Note: the carbon atoms on one of the phenyl substituents are no longer equivalent, presumably as a result of restricted rotation on the NMR timescale. Quantitative ^{13}C NMR was employed to assign those peaks which correspond to multiple carbon signals.

IR: 3727, 3061, 2932, 2859, 2351, 1658, 1592, 1563, 1555, 1452, 1389, 1261, 1051 cm^{-1};

HRMS (ESI): calculated for $[C_{22}H_{20}ClNO+H]^+$: 350.1306, found: 350.1310.

N-Aryl Ketenimines as Synthetic Equivalents of Other Aza-Dienophiles

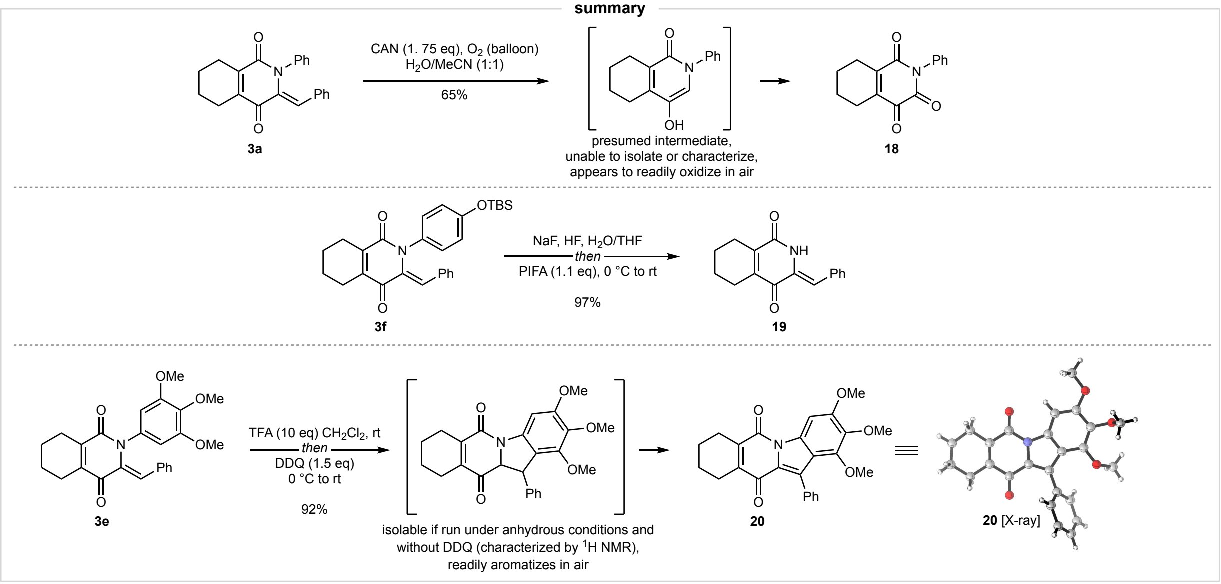

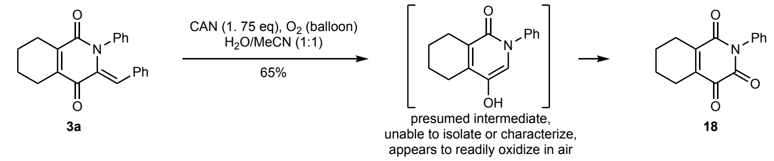

A conceptually related air-promoted oxidation of our presumed intermediate was reported by Massa.[60] To a 5 mL one-neck round-bottom flask open to air and equipped with a stir bar was added **3a** (30.0 mg, 0.0911 mmol, 1.00 equiv), acetonitrile (0.455 mL, 0.20 M), and a solution of ceric ammonium nitrate (87.4 mg, 0.159 mmol, 1.75 equiv) in H_2O (0.455 mL, 0.20 M). An O_2 balloon was attached to the round-bottom flask and the reaction was stirred under an O_2 atmosphere for 24 hours before being diluted with CH_2Cl_2 and saturated aqueous sodium bicarbonate. The layers were separated, and the aqueous layer was washed with CH_2Cl_2. The combined organics were washed with brine, dried over $MgSO_4$, filtered, and concentrated under reduced pressure. Purification via flash column chromatography (ratio of silica to crude mass = 100:1, eluting with a gradient of 1:19 to 3:7 EtOAc:hexane) yielded **18**.

Yield: 15.0 mg, 0.059 mmol, 65%;

Appearance: Pale yellow solid;

R*f*: 0.37 (3:7 EtOAc:hexane);

^{1}H NMR (400 MHz, $CDCl_3$): δ 7.54 – 7.44 (m, 3H), 7.17 (d, *J* = 7.1 Hz, 2H), 2.62 (d, 4H), 1.80 (s, 4H) ppm;

^{13}C NMR (100 MHz, $CDCl_3$): δ 175.7, 163.1, 155.8, 145.1, 144.6, 133.4, 129.6, 129.4, 128.2, 25.2, 22.7, 21.1, 20.7 ppm;

IR: 3063, 2936, 2867, 1727, 1692, 1679, 1641, 1493, 1453, 1402, 1351, 1281, 1017 cm^{-1};

HRMS (ESI): calculated for $[C_{15}H_{13}NO_3+H]^+$: 256.0974, found: 256.0974.

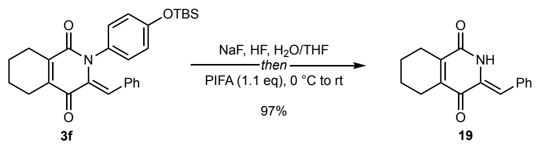

Note: Hydrofluoric acid (HF) is especially hazardous, and all appropriate cautions should be taken. Oxidative deprotection adapted from a procedure originally reported by Nakazaki.[61] To a 10 mL plastic scintillation vial open to air and equipped with a stir bar was added **3f** (30.0 mg, 0.0653 mmol, 1.00 equiv), THF (0.473 mL, 0.20 M), and a fluoride buffer[62] (pH = 5, 1.18 mL). The reaction was stirred for 20 hours by which time TLC analysis indicated the complete consumption of starting material. The vial was submerged in an ice/water bath and [bis(trifluoroacetoxy)iodo]benzene (30.9 mg, 0.0718 mmol, 1.10 equiv) was added portionwise. The ice/water bath was removed, and the reaction was allowed to warm to ambient temperature and stirred for 1 hour. The mixture was diluted with CH_2Cl_2 and saturated aqueous sodium bicarbonate. The layers were separated, and the aqueous layer was washed with CH_2Cl_2. The combined organics were washed with brine, dried over $MgSO_4$, filtered, and concentrated under reduced pressure. The crude residue was triturated with cold hexane, and the resulting yellow solid was further purified via flash column chromatography (ratio of silica to crude mass = 50:1, eluting with 98:2 CH_2Cl_2:Et_2O).

Yield: 16.0 mg, 0.0632 mmol, 97%;

Appearance: Yellow solid;

R*f*: 0.20 (1:4 EtOAc:hexane);

^{1}H NMR (400 MHz, $CDCl_3$): δ 8.14 (br s, 1H), 7.49 – 7.34 (m, 5H), 7.06 (s, 1H), 2.55 (app d, J = 27.8 Hz, 4H), 1.77 – 1.71 (m, 4H) ppm;

^{13}C NMR (150 MHz, $CDCl_3$): δ 179.4, 160.4, 145.4, 143.8, 133.4, 131.1, 129.7, 129.3, 129.1, 117.9, 24.6, 23.2, 21.3, 21.1 ppm;

IR: 3314, 2941, 1665, 1634, 1583, 1456, 1419, 1345, 941, 758 cm^{-1};

HRMS (ESI): calculated for $[C_{16}H_{15}NO_2+H]^+$: 254.1181, found: 254.1169.

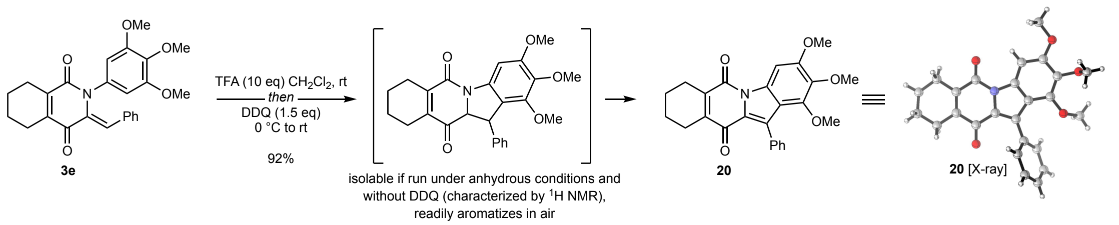

To a 5 mL one-neck round-bottom flask open to air and equipped with a stir bar was added **3e** (50.0 mg, 0.119 mmol, 1.00 equiv), CH_2Cl_2 (1.19 mL, 0.10 M), and trifluoroacetic acid (0.0918 mL, 1.19 mmol, 10.0 equiv). The reaction flask was loosely stoppered and allowed to stir for 1.5 hours by which time TLC analysis indicated the complete consumption of starting material. The round-bottom flask was submerged in an ice/water bath, and DDQ (40.6 mg, 0.179 mmol, 1.50 equiv) was added. The ice/water bath was removed, and the reaction was stirred at ambient

temperature for 16 hours. The mixture was diluted with CH_2Cl_2 and saturated aqueous sodium bicarbonate. The layers were separated, and the aqueous layer was washed with CH_2Cl_2. The combined organics were washed with brine, dried over $MgSO_4$, filtered, and concentrated under reduced pressure. Purification via flash column chromatography (ratio of silica to crude mass = 100:1, eluting with 99:1 CH_2Cl_2:Et_2O) yielded **20**. A small quantity of pure **20** was crystallized via slow evaporation of CH_2Cl_2 to yield orange brick-like crystals suitable for X-ray analysis.

Yield: 46.0 mg, 0.119 mmol, 92%;

Appearance: Orange solid;

$\mathbf{R}_f$: 0.45 (1:4 EtOAc:hexane);

M.p.: 203.8 – 206.7 °C;

^{1}H NMR (900 MHz, $CDCl_3$): δ 7.95 (s, 1H), 7.50 (d, J = 7.2 Hz, 2H), 7.44 (t, J = 7.3 Hz, 2H), 7.43 – 7.41 (m, 1H), 4.00 (s, 3H), 3.85 (s, 3H), 3.44 (s, 3H), 2.66 – 2.63 (m, 2H), 2.48 – 2.45 (m, 2H), 1.78 – 1.75 (m, 2H), 1.72 – 1.69 (m, 2H) ppm;

^{13}C NMR (225 MHz, $CDCl_3$): δ 176.0, 160.7, 157.5, 149.6, 147.2, 141.2, 140.3, 133.7, 133.3, 132.6, 129.6, 128.4, 127.59, 126.3, 116.6, 95.5, 61.5, 61.3, 56.6, 24.1, 22.8, 21.4, 21.0 ppm;

IR: 2938, 2836, 2363, 1681, 1642, 1601, 1537, 1494, 1379, 1369, 1302, 1227, 1016, 941, 872, 726 cm^{-1};

HRMS (ESI): calculated for $[C_{25}H_{23}NO_5+H]^+$: 418.1654, found: 418.1649.

One-Pot Aza-Diels–Alder/Oxa-Diels–Alder Sequences

summary

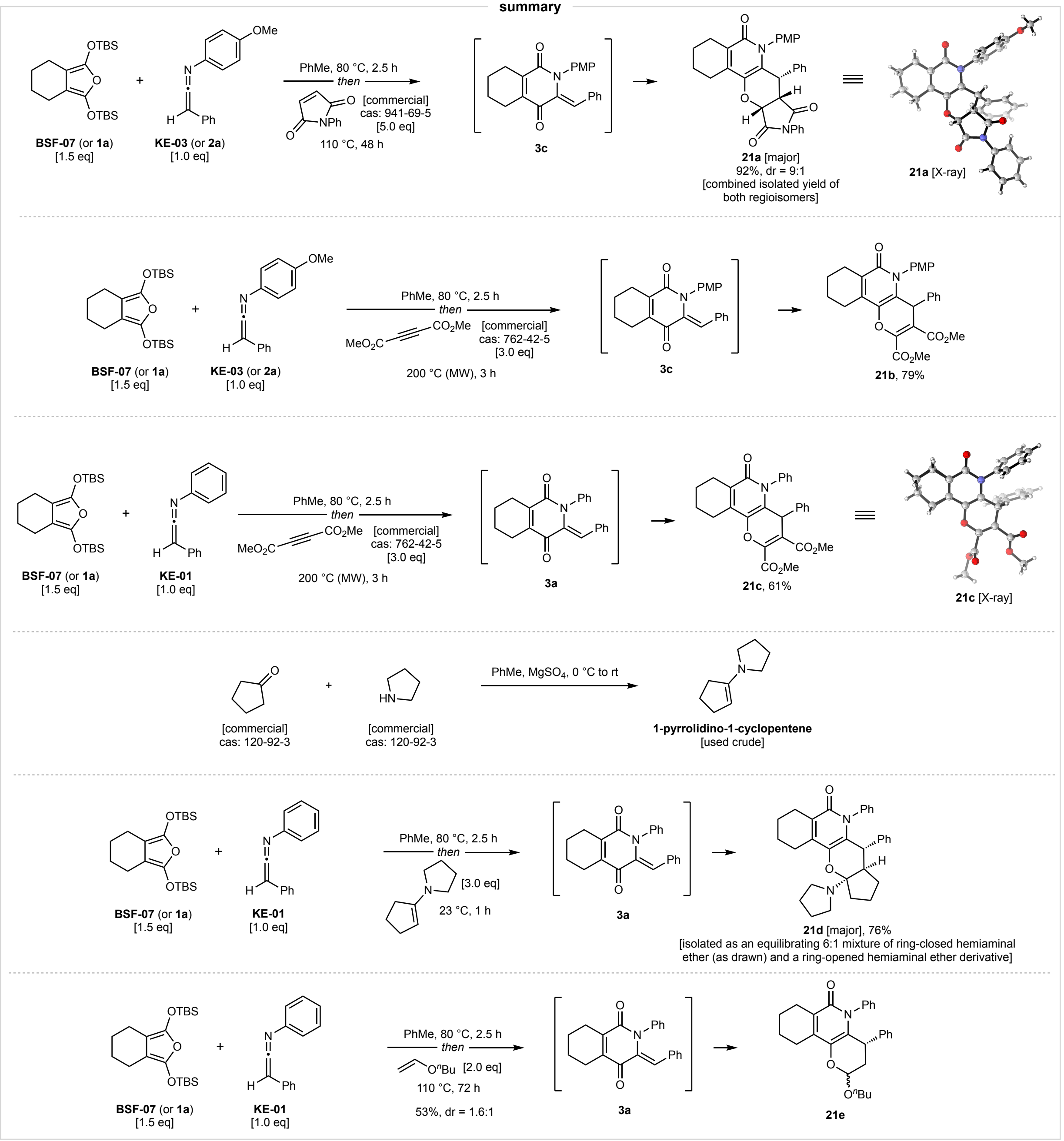

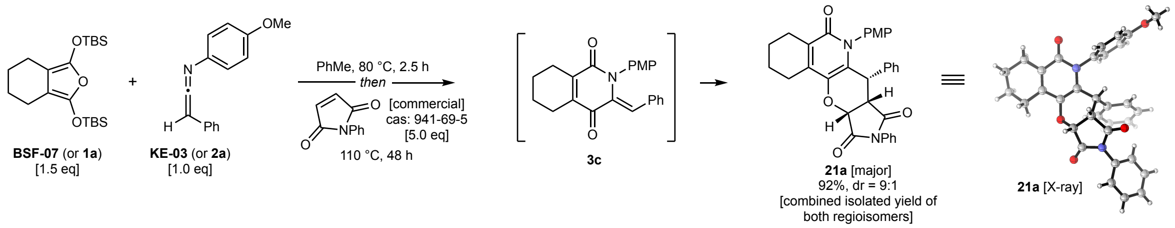

Given the propensity of silyloxyfurans and ketenimines to decompose if not appropriately handled (see notes concerning stability and storage in their respective preparation sections earlier in this document), directly before the Diels–Alder reaction the purity of both was determined by ^{1}H NMR using 1,2-dibromomethane as internal standard. To minimize confusion, the masses of silyloxyfurans and ketenimines are omitted from this procedure. To a flame-dried 25 mL one-neck round-bottom flask under argon and equipped with a stir bar was added **2a** (0.824 mmol, 1.00 equiv), anhydrous toluene (1.65 mL, 0.50 M), and **1a** (1.24 mmol, 1.50 equiv). The reaction was warmed on a heating mantle at 80 °C for 2.5 hours, followed by addition of *N*-phenylmaleimide (0.714 g, 5.00 equiv). The reaction was further warmed to 110 °C for 48 hours, cooled to ambient temperature, and concentrated under reduced pressure. Purification via flash column chromatography (ratio of silica to crude mass = 50:1, eluting with a gradient of CH_2Cl_2 to 1:4 Et_2O:CH_2Cl_2) yielded **21a** as a diastereomeric mixture. A small quantity of the major diastereoisomer was isolated in pure form via the slow evaporation of mixture of 1,2-dichloroethane and EtOAc. The resulting white crystals were suitable for X-ray analysis.

Yield: 0.405 g, 0.760 mmol, 92%;

Appearance: Pale brown solid;

R*f*: 0.50 (5:95 MeOH:CH_2Cl_2);

M.p.: >260 °C;

^{1}H NMR (600 MHz, $CDCl_3$, major diastereoisomer): δ 7.31 – 7.20 (m, 6H), 7.17 (dd, *J* = 8.7, 2.6 Hz, 1H), 7.04 (dd, *J* = 8.7, 2.9 Hz, 1H), 6.92 (d, *J* = 7.6 Hz, 2H), 6.64 (dd, *J* = 8.8, 2.9 Hz, 1H), 6.38 (d, *J* = 9.0 Hz, 2H), 6.23 (dd, *J* = 8.7, 2.5 Hz, 1H), 5.26 (d, *J* = 8.9 Hz, 1H), 4.17 (d, *J* = 7.3 Hz, 1H), 3.83 (s, 3H), 3.64 (t, *J* = 8.1 Hz, 1H), 2.98 – 2.87 (m, 1H), 2.82 – 2.72 (m, 1H), 2.58 (br s, 2H), 1.91 – 1.76 (m, 4H) ppm;

^{13}C NMR (150 MHz, $CDCl_3$, major diastereoisomer): δ 172.6, 159.8, 141.5, 135.9, 134.6, 130.4, 129.9, 129.7, 129.6, 129.5, 129.2, 129.2, 129.1, 129.0, 128.7, 126.7, 126.3, 114.9, 114.7, 72.7, 55.7, 45.9, 40.5, 24.3, 24.0, 21.9, 21.5 ppm;

IR: 3065, 2936, 2838, 1726, 1659, 1592, 1511, 1494, 1390, 1250, 1198, 913, 733 cm^{-1};

HRMS (ESI): calculated for $[C_{33}H_{28}O_5N_2+H]^+$: 533.2071, found: 533.2074.

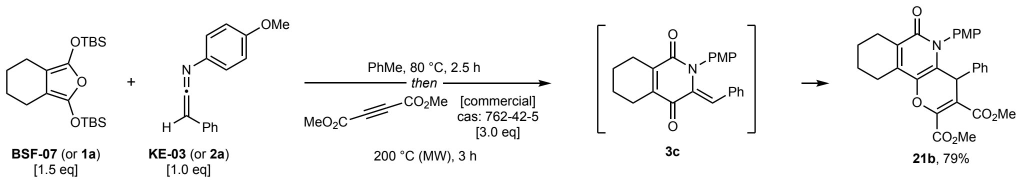

Given the propensity of silyloxyfurans and ketenimines to decompose if not appropriately handled (see notes concerning stability and storage in their respective preparation sections earlier in this document), directly before the Diels–Alder reaction the purity of both was determined by ^{1}H NMR using 1,2-dibromomethane as internal standard. To minimize confusion, the masses of silyloxyfurans and ketenimines are omitted from this procedure. To a flame-dried 8 mL microwave vessel under argon and equipped with a stir bar was added **2a** (0.824 mmol, 1.00 equiv), anhydrous toluene (1.65 mL, 0.50 M), and **1a** (1.24 mmol, 1.50 equiv). The reaction was heated to 80 °C for 2.5 hours in a microwave reactor, then cooled to ambient temperature before dimethyl acetylenedicarboxylate (0.351 g, 2.47 mmol, 3.00 equiv) was added. The reaction was heated to 200 °C for 3 hours in a microwave reactor, allowed to cool to ambient temperature, and concentrated under reduced pressure. Purification via flash column chromatography (ratio of silica to crude mass = 50:1, eluting with CH_2Cl_2 to 1:9 Et_2O:CH_2Cl_2) yielded **21b**.

Yield: 327 mg, 0.652 mmol, 79%;

Appearance: Yellow solid;

R*f*: 0.24 (1:9 Et_2O:CH_2Cl_2);

^{1}H NMR (400 MHz, $CDCl_3$): δ 7.14 – 7.07 (m, 4H), 7.00 (dd, *J* = 8.6, 2.9 Hz, 1H), 6.67 (d, *J* = 6.2 Hz, 2H), 6.55 (dd, *J* = 8.7, 2.9 Hz, 1H), 6.10 (dd, *J* = 8.8, 2.6 Hz, 1H), 4.63 (s, 1H), 3.88 (s, 3H), 3.82 (s, 3H), 3.58 (s, 3H), 2.75 – 2.70 (m, 2H), 2.55 – 2.52 (m, 2H), 1.84 – 1.77 (m, 4H) ppm;

^{13}C NMR (100 MHz, $CDCl_3$): δ 165.3, 162.8, 162.2, 159.7, 147.2, 140.9, 139.5, 133.7, 131.0, 129.3, 129.1, 128.7 (2 coincident peaks), 128.5, 128.4 (2 coincident peaks), 127.7, 124.3, 114.4, 114.4, 109.8, 55.7, 53.2, 52.3, 40.0, 24.1, 23.6, 21.8, 21.3 ppm; Note: the carbon atoms on one of the aryl substituents are no longer equivalent, presumably as a result of restricted rotation on the NMR timescale. Quantitative ^{13}C NMR was employed to assign those peaks which correspond to multiple carbon signals.

IR: 2937, 2862, 1754, 1725, 1666, 1600, 1511, 1461, 1440, 1348, 1300, 1250 cm^{-1};

HRMS (ESI): calculated for $[C_{29}H_{27}O_7N+H]^+$: 502.1860, found: 502.1860.

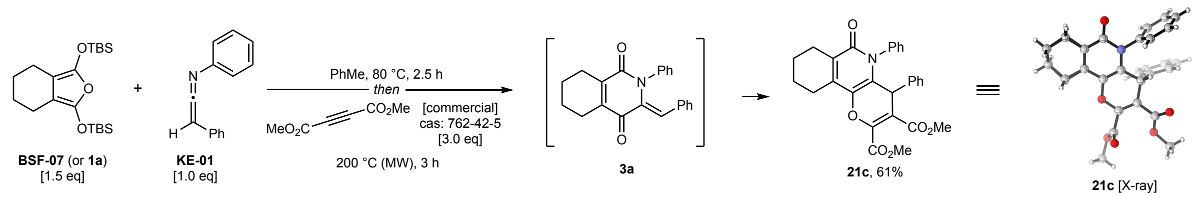

Given the propensity of silyloxyfurans and ketenimines to decompose if not appropriately handled (see notes concerning stability and storage in their respective preparation sections earlier in this document), directly before the Diels–Alder reaction the purity of both was determined by ^{1}H NMR using 1,2-dibromomethane as internal standard. To minimize confusion, the masses of silyloxyfurans and ketenimines are omitted from this procedure. To a flame-dried 8 mL microwave vessel under argon and equipped with a stir bar was added **KE-01** (0.494 mmol, 1.00 equiv), anhydrous toluene (0.989 mL, 0.50 M), and **1a** (0.742 mmol, 1.50 equiv). The reaction was heated to 80 °C for 2.5 hours in a microwave reactor, then cooled to ambient temperature before dimethyl acetylenedicarboxylate (211 mg, 0.183 mL, 1.48 mmol, 3.00 equiv) was added. The reaction was heated to 200 °C for 3 hours in a microwave reactor, allowed to cool to ambient temperature, and concentrated under reduced pressure. Purification via flash column chromatography (ratio of silica to crude mass = 50:1, eluting with 1:9 Et_2O:CH_2Cl_2) yielded **21c.**

Yield: 143 mg, 0.302 mmol, 61%;

Appearance: Yellow solid;

R*f*: 0.36 (1:9 Et_2O:CH_2Cl_2);

M.p.: 192 – 197 °C;

^{1}H NMR (600 MHz, $CDCl_3$): δ 7.51 (m, 1H), 7.35 (tt, J = 7.5, 1.2 Hz, 1H), 7.19 (dt, J = 8.1, 1.4 Hz, 1H), 7.15 – 7.11 (m, 1H), 7.11 – 7.07 (m, 2H), 7.03 (td, J = 7.7, 1.6 Hz, 1H), 6.62 – 6.60 (m, 2H), 6.22 (dt, J = 7.8, 1.8 Hz, 1H), 4.62 (s, 1H), 3.88 (s, 3H), 3.57 (s, 3H), 2.78 – 2.69 (m, 2H), 2.54 (app s, 2H), 1.85 – 1.76 (m, 4H) ppm;

^{13}C NMR (150 MHz, $CDCl_3$): δ 165.2, 162.8, 161.9, 147.2, 140.8, 139.6, 136.6, 133.7, 130.1, 129.2, 129.0, 128.8, 128.6 (3 coincident peaks), 128.4 (2 coincident peaks), 128.1, 127.7, 123.8, 109.8, 53.2, 52.3, 40.0, 24.0, 23.6, 21.7, 21.3 ppm; Note: the carbon atoms on one of the phenyl substituents are no longer equivalent, presumably as a result of restricted rotation on the NMR timescale. Quantitative ^{13}C NMR was employed to assign those peaks which correspond to multiple carbon signals.

IR: 2935, 1725, 1662, 1606, 1439, 1292, 1203, 1090 cm^{-1};

HRMS (ESI): calculated for $[C_{28}H_{25}O_6N+H]^+$: 472.1755, found: 472.1750.

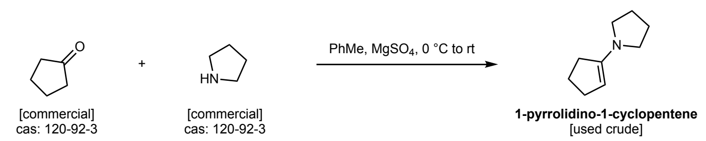

Prepared according to a modification of the procedure reported by Zhang.[63] To a flame-dried 25 mL one-neck round-bottom flask under argon and equipped with a stir bar was added cyclopentanone (225 mg, 0.240 mL, 2.67 mmol, 1.00 equiv), anhydrous toluene (4.12 mL, 0.65 M), and magnesium sulfate (1.61 g, 13.4 mmol, 5.00 equiv). The reaction flask was submerged in an ice/water bath, and pyrrolidine (0.951 g, 1.10 mL, 13.4 mmol, 5.00 equiv) was added dropwise. The reaction was allowed to stir for 30 mins, warmed to ambient temperature, and stirred overnight. The reaction mixture was filtered over a sintered funnel (washing with CH_2Cl_2), and the filtrate was concentrated under reduced pressure to afford crude 1-pyrrolidino-1-cyclopentene. The crude mixture was submitted to the next reaction without further purification.

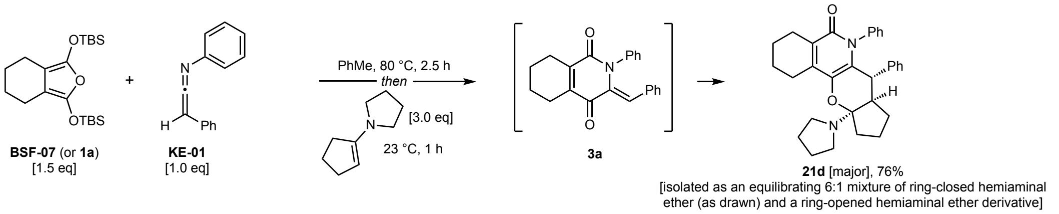

Given the propensity of silyloxyfurans and ketenimines to decompose if not appropriately handled (see notes concerning stability and storage in their respective preparation sections earlier in this document), directly before the Diels–Alder reaction the purity of both was determined by ^{1}H NMR using 1,2-dibromomethane as internal standard. To minimize confusion, the masses of silyloxyfurans and ketenimines are omitted from this procedure. To a flame-dried 25 mL one-neck round-bottom flask under argon and equipped with a stir bar was added **KE-01** (0.880 mmol, 1.00 equiv), anhydrous toluene (1.76 mL, 0.50 M), and **1a** (1.32 mmol, 1.50 equiv). The reaction was warmed on a heating mantle at 80 °C for 2.5 hours, cooled to ambient temperature, followed by addition of crude 1-pyrrolidino-1-cyclopentene (0.362 g, 2.64 mmol, 3.00 equiv) as a solution in anhydrous toluene (0.210 mL). The reaction was allowed to stir for one hour at ambient temperature and then concentrated under reduced pressure. Purification via flash column chromatography using phosphate buffered silica (ratio of buffered silica to crude mass = 50:1, eluting with a gradient of 1:99 to 1:4 Et_2O:CH_2Cl_2) yielded **21d** as an equilibrating 6:1 mixture of its ring-closed hemiaminal ether form (as drawn) and a ring-opened hemiaminal ether derivative. Although the structure of the ring-opened form could not be unequivocally assigned, evidence for its identity includes:

i. To a flame-dried 10 mL one-neck round-bottom flask under argon and equipped with a stir bar was added **21d** (6:1 mixture favoring its ring-opened hemiaminal ether form, 30.0 mg, 0.064 mmol, 1.00 equiv), toluene (0.210 mL, 0.30 M), and trifluoroacetic acid (0.250 mL, 3.20 mmol, 50.0 equiv). The reaction was warmed on a heating mantle at 110 °C for 3 hours, cooled to ambient temperature, and concentrated under reduced pressure. The crude mixture was diluted with CH_2Cl_2 and quenched with saturated aqueous sodium $NaHCO_3$. The layers were separated, and the aqueous layer was washed with CH_2Cl_2. The combined organics were

washed brine, dried over $MgSO_4$, filtered, and concentrated under reduced pressure yielding **21d** now as a 1:4 mixture favoring its ring-opened hemiaminal ether form.

ii. After leaving the above mixture of in $CDCl_3$ overnight it converted back to the original 6:1 mixture favoring its ring-closed hemiaminal ether form.

Yield: 311 mg, 0.666 mmol, 76%;

Appearance: Pale yellow solid;

R*f*: 0.35 (1:4 Et_2O:CH_2Cl_2);

^{1}H NMR (400 MHz, $CDCl_3$, only major isomer indicated): δ 7.39 (td, *J* = 7.6, 1.5 Hz, 1H), 7.23 – 7.17 (m, 1H), 7.12 – 7.05 (m, 4H), 6.94 (td, *J* = 7.8, 1.6 Hz, 1H), 6.84 (m, 2H), 6.42 (d, *J* = 7.9 Hz, 1H), 3.44 (s, 1H), 2.71 – 2.63 (m, 2H), 2.59 (m, 2H), 2.50 (m, 3H), 2.30 – 2.20 (m, 2H), 2.07 – 1.95 (m, 2H), 1.79 (m, *J* = 7.5 Hz, 6H), 1.67 (m, 1H), 1.53 (m, 5H) ppm;

^{13}C NMR (150 MHz, $CDCl_3$, only major isomer indicated): δ 161.5, 142.7, 142.0, 138.2, 135.7, 129.7, 128.9, 128.6, 128.4, 127.9, 127.9, 127.3, 126.4, 101.5, 49.2, 46.5, 45.1, 33.1, 32.6, 24.4, 24.1, 23.8, 22.1, 21.8, 21.6 ppm;

IR: 2939, 2437, 1743, 1660, 1606, 1598, 1562, 1553, 1493, 1453, 1430, 1162 cm^{-1};

HRMS (ESI): calculated for $[C_{31}H_{34}O_2N_2+H]^+$: 467.2693, found: 467.2693.

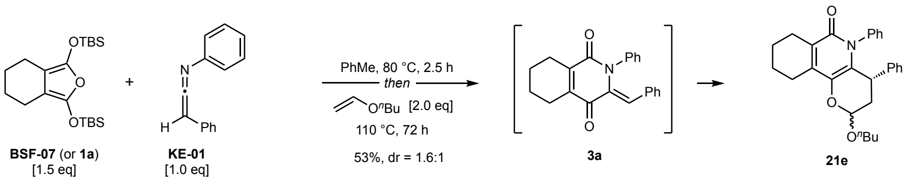

To a flame-dried 25 mL one-neck round-bottom flask under argon and equipped with a stir bar was added **KE-01** (90.0 mg, 0.466 mmol, 1.00 equiv), anhydrous toluene (0.931 mL, 0.50 M), and **1a** (0.267 g, 0.699 mmol, 1.50 equiv). The reaction was warmed on a heating mantle at 80 °C for 2.5 hours, followed by addition of addition of *n*-butyl vinyl ether (93.3 mg, 2.00 equiv). The reaction was further warmed to 110 °C for 72 hours, cooled to ambient temperature, and concentrated under reduced pressure. Purification via flash column chromatography (ratio of silica to crude mass = 50:1, eluting with a gradient of 1:99 to 1:4 Et_2O:CH_2Cl_2) yielded **21e** as a 1.6:1 diastereomeric mixture.

Yield: 106 mg, 0.247 mmol, 53%;

Appearance: Off-white solid;

R*f*: 0.54 (1:4 Et_2O:CH_2Cl_2);

^{1}H NMR (400 MHz, $CDCl_3$, both diastereoisomers indicated): δ 7.37 – 7.30 (m, 2.5H), 7.17 – 6.96 (m, 12.8H), 6.84 – 6.77 (m, 2.6H), 6.68 – 6.65 (m, 1.6H), 6.65 – 6.60 (m, 3H), 6.32 – 6.28 (m, 2H), 5.07 (dd, *J* = 6.1, 2.4 Hz, 2.4H), 3.94 – 3.80 (m, 4.8H), 3.58 – 3.46 (m, 2.7H), 2.67 (m, 4.8H), 2.57 (m, 4.8H), 2.34 (ddd, *J* = 13.9, 8.1, 2.4 Hz, 1.7H), 2.21 (td, *J* = 12.9, 6.3 Hz, 1.1H), 2.04 (dt, *J* = 13.1, 6.2 Hz, 1.7H), 1.96 (ddd, *J* = 13.6, 6.7, 2.4 Hz, 1.0H), 1.88 – 1.70 (m, 10.3H), 1.59 – 1.46 (m, 5.1H), 1.40 – 1.26 (m, 5.7H), 0.90 (m, 7.4H) ppm;

^{13}C NMR (150 MHz, $CDCl_3$, both diastereoisomers indicated): δ 161.5, 161.5, 143.2, 142.6, 141.5, 141.4, 138.1, 136.1, 130.7, 130.3, 128.7, 128.5, 128.5, 128.2, 128.1, 128.0, 127.9, 127.7, 127.6, 127.9, 127.4, 127.1, 126.6, 126.3, 124.3, 123.9, 98.2, 96.1, 68.9, 68.6, 39.3, 38.5, 37.6, 37.4, 31.8, 31.7, 24.1, 24.1, 23.7, 23.7, 22.0, 22.0, 21.7, 21.7, 19.4, 19.3, 13.9, 13.9 ppm;

IR: 3026, 2931, 2872, 1658, 1649, 1643, 1605, 1591, 1493, 1440, 1380, 1307, 1269 cm^{-1};

HRMS (ESI): calculated for $[C_{28}H_{31}O_3N+H]^+$: 430.2377, found: 430.2377.

Comparing Ketenimines Against Imino Dienophiles

Dienophile Synthesis

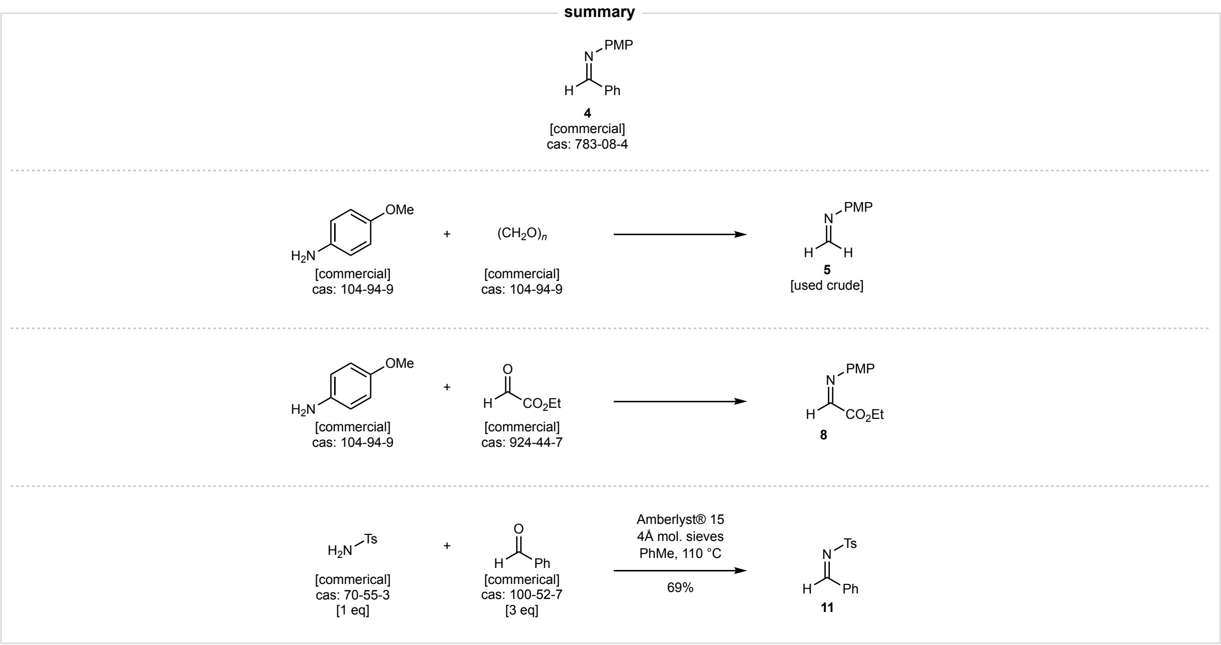

Imine **5** was prepared in one step according the procedure reported by Jiang.[64] Characterization data matched those reported by Jiang.[64] For a discussion concerning its propensity to polymerize, see the rate study section later in this document.

Imine **8** was prepared in one step according the procedure reported by Bräse.[65] Characterization data matched those reported by Bräse.[65]

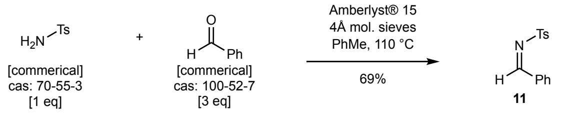

Prepared according to a modification of the procedure reported by Shimizu and Kanai.[66] To a flame-dried 250 mL one-neck round-bottom flask under argon and equipped with a stir bar and high efficiency air condenser was added *p*-toluenesulfonamide (2.25 g, 13.1 mmol, 1.00 equiv), activated powdered 4Å molecular sieves (0.400 g/mmol of sulfonamide, 5.24 g), Amberlyst 15® (5.00 mg/mmol of sulfonamide, 65.5 mg), anhydrous toluene (52.6 mL, 0.25 M), and benzaldehyde (4.18 g, 39.4 mmol, 3.00 equiv). The reaction flask was warmed on a heating mantle at 110 °C overnight and then allowed to cool to ambient temperature. The reaction was diluted with CH_2Cl_2, filtered over a Celite plug (eluting with CH_2Cl_2), and concentrated under reduced pressure. The crude reaction mixture was dissolved in a minimum volume of EtOAc, diluted with hot hexane, cooled to ambient temperature, and transferred to a freezer (−20 °C), allowing crystals to form. Filtration (washing with hexane) yielded imine **11**.

Characterization data matched those reported by Morrill.[67]

Yield: 2.36 g, 9.10 mmol, 69%;

Appearance: Flakey white solid;

R*f*: 0.42 (1:4 EtOAc:hexane);

^{1}H NMR (400 MHz, $CDCl_3$): δ 9.03 (s, 1H), 7.93 – 7.88 (m, 4H), 7.61 (t, J = 7.5 Hz, 1H), 7.48 (t, J = 7.8 Hz, 2H), 7.34 (d, J = 8.2 Hz, 2H), 2.44 (s, 3H) ppm.

Diels–Alder Reactions

summary

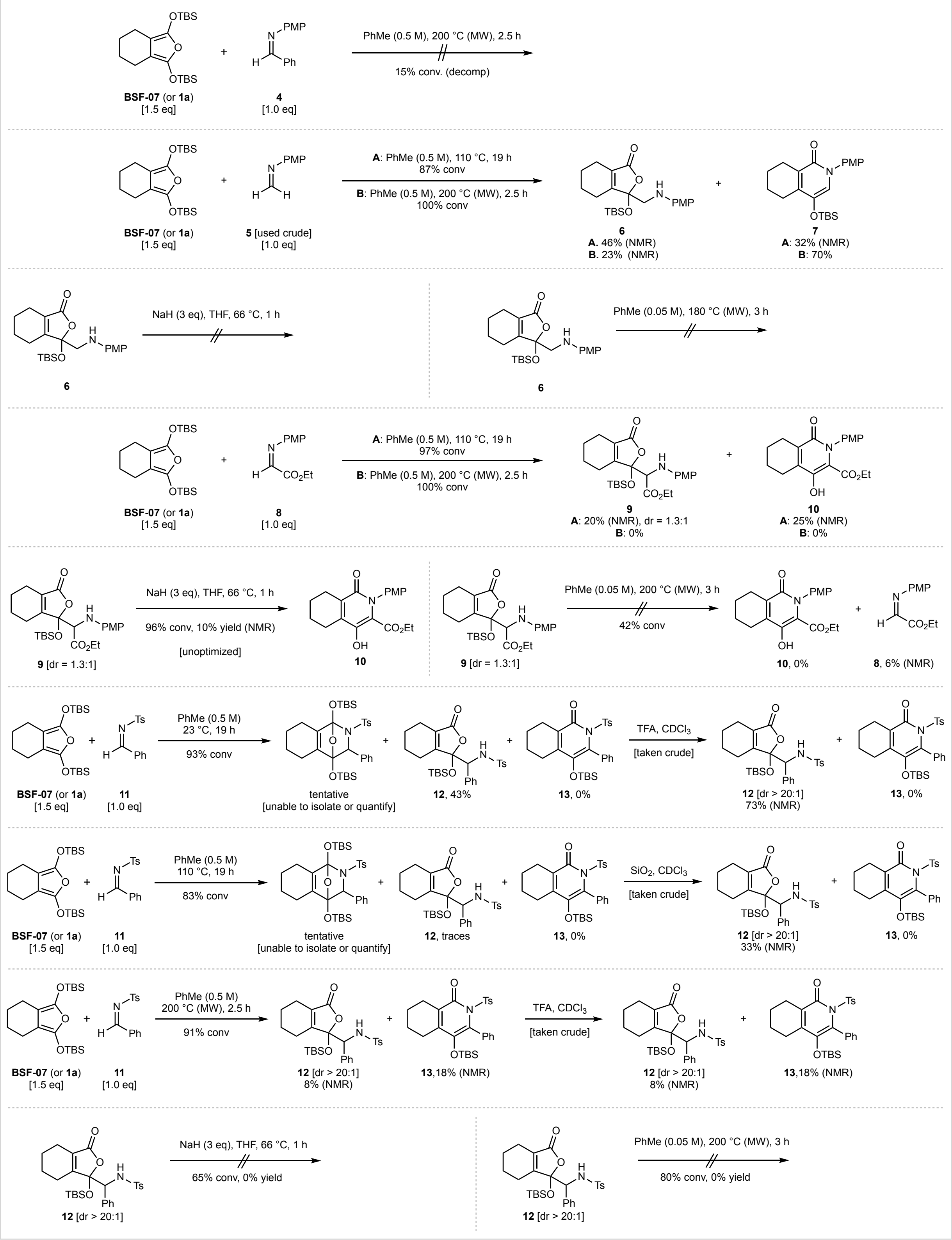

Given the propensity of **BSF-07** to decompose if not appropriately handled (see notes concerning stability and storage earlier in this document), directly before the Diels–Alder reaction, its purity was determined by ^{1}H NMR using 1,2-dibromomethane as internal standard. To minimize confusion, its mass was omitted from the following procedures.

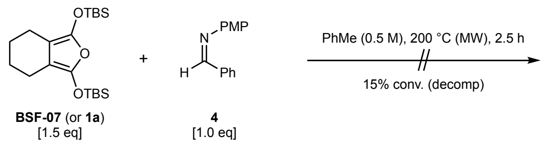

To a flame-dried 8 mL microwave vessel under argon and equipped with a stir bar was added imine **4** (500 mg, 0.237 mmol, 1.00 equiv), anhydrous toluene (0.470 mL, 0.50 M), and **BSF-07** (0.355 mmol, 1.50 equiv). The reaction was heated to 200 °C for 2.5 hours in a microwave reactor, allowed to cool to ambient temperature, and concentrated under reduced pressure. Conversion was determined using 1,2-dichloroethane as internal standard.

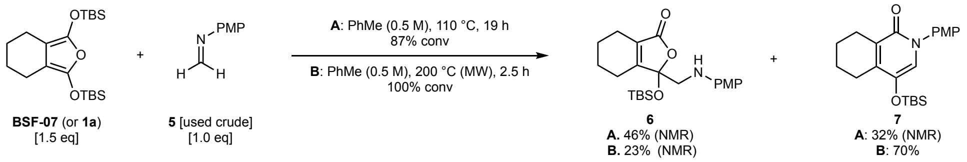

For a discussion concerning the propensity of **5** to polymerize, see the rate study section later in this document.

Conditions A: To a flame-dried 5 mL one-neck round-bottom flask equipped with a stir bar under argon was added freshly prepared imine **5** (0.052 g, 0.307 mmol, 1.00 equiv), anhydrous toluene (0.590 mL, 0.50 M), and **BSF-07** (0.464 mmol, 1.50 equiv). The reaction was warmed on a heating mantle at 110 °C for 19 hours, cooled to ambient temperature, and concentrated under reduced pressure. NMR yields were determined by addition of an internal standard (dimethyl terephthalate).

Conditions B: To a flame-dried 8 mL microwave vessel under argon and equipped with a stir bar was added freshly prepared imine **5** (0.105 g, 0.777 mmol, 1.00 equiv), anhydrous toluene (1.60 mL, 0.50 M), and **BSF-07** (1.16 mmol, 1.50 equiv). The reaction was heated to 200 °C for 2.5 hours in a microwave reactor, allowed to cool to ambient temperature, and concentrated under reduced pressure. NMR yields were determined by addition of an internal standard (dimethyl terephthalate).

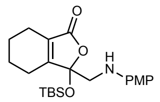

6

Conditions A.

For characterization purposes, a small amount of the crude reaction mixture was purified via flash column chromatography using phosphate buffered silica (pH = 7, ratio of buffered silica to crude mass = 50:1, eluting with a gradient of hexane to 1:6 EtOAc:hexane), yielding **6**.

Appearance: Brown oil;

R*f*: 0.53 (1:4 EtOAc:hexane);

^{1}H NMR (400 MHz, $CDCl_3$): δ 6.73 (d, *J* = 7.6 Hz, 2H), 6.55 (d, *J* = 7.7 Hz, 2H), 3.72 (s, 3H), 3.49 (d, *J* = 13.4 Hz, 1H), 3.38 (d, *J* = 13.3 Hz, 1H), 2.31 – 2.22 (app d, *J* = 19.9 Hz, 1H), 2.18 – 2.08 (app. t, 3H), 1.70 – 1.40 (m, 4H), 0.88 (s, 9H), 0.15 (s, 3H), 0.01 (s, 3H);

^{13}C NMR (100 MHz, $CDCl_3$): δ 170.8, 163.1, 152.8, 141.8, 128.7, 115.3, 115.0, 106.7, 55.9, 51.5, 25.7, 22.3, 21.5, 21.4, 19.9, 18.0, –3.14, –3.54 ppm;

IR: 3393, 2935, 2858, 1777, 1765, 1513, 1462, 1300, 1252, 1175 cm^{-1};

HRMS (ESI): calculated for $[C_{22}H_{33}NO_4Si+H]^+$: 404.2252, found: 404.2254.

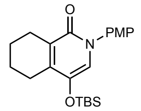

7

Conditions B.

Purification via flash column chromatography (ratio of silica to crude mass = 100:1, eluting with hexane to 1:1 EtOAc:Hexane) yielded **7**.

Yield: 210 mg, 0.545 mmol, 70%;

Appearance: Yellow solid;

R*f*: 0.32 (1:1 EtOAc:hexane);

^{1}H NMR (600 MHz, $CDCl_3$): δ 7.28 (d, *J* = 8.8 Hz, 2H), 6.96 (d, *J* = 8.8 Hz, 2H), 6.70 (s, 1H), 3.84 (s, 3H), 2.55 (app d, *J* = 20.1 Hz, 4H), 1.77 – 1.71 (m, 4H), 0.99 (s, 9H), 0.18 (s, 6H) ppm;

^{13}C NMR (150 MHz, $CDCl_3$): δ 160.3, 159.1, 144.3, 137.5, 134.7, 129.1, 127.8, 120.5, 114.5, 55.7, 25.8, 24.7, 24.4, 22.0, 21.6, 18.3, –4.3 ppm;

IR: 3047, 2933, 2858, 2360, 1769, 1691, 1665, 1600, 1573, 1512, 1443, 1250 cm^{-1};

HRMS (ESI): calculated for $[C_{22}H_{31}NO_3Si+H]^+$: 386.2146, found: 386.2151.

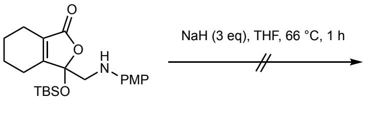

6

To a flame-dried 2-dram vial under argon and equipped with a stir bar was added **6** (29.0 mg, 71.9 µmol, 1.00 equiv) and anhydrous THF (0.378 mL, 0.19 M). The reaction vial was submerged in an ice/water bath, and NaH (60% dispersion in mineral oil, 8.62 mg, 0.216 mmol, 3.00 equiv) was added in one portion. The reaction vial was removed from the ice/water bath, sealed, and warmed on a heating mantle at 66 °C for 1 hour. The reaction was cooled to ambient temperature, quenched with saturated aqueous NH_4Cl, and extracted with EtOAc (×3). The combined organics were washed with water and brine, dried over $MgSO_4$, filtered, and concentrated under reduced pressure. No cyclized product was observed, and the crude contained predominantly starting material.

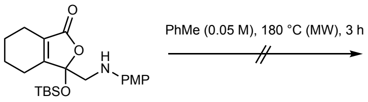

6

To a flame-dried 8 mL microwave vessel under argon and equipped with a stir bar was added **6** (73.0 mg, 0.181 mmol, 1.00 equiv) and anhydrous toluene (3.62 mL, 0.05 M). The reaction was heated to 180 °C for 3 hours in a microwave reactor, allowed to cool to ambient temperature, and concentrated under reduced pressure. NMR yields were determined by addition of dimethyl terephthalate as internal standard. No cyclized product was observed, and the crude contained predominantly starting material.

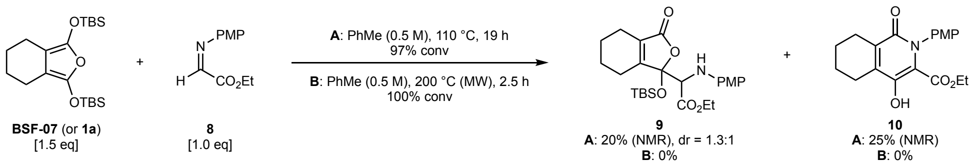

Conditions A: To a flame-dried 2-dram vial under argon and equipped with a stir bar added **8** (104 mg, 0.502 mmol, 1.00 equiv), anhydrous toluene (1.00 mL, 0.50 M), and **BSF-07** (0.753 mmol, 1.50 equiv). The reaction vial was sealed, warmed on a heating mantle at 110 °C for 19 hours, cooled to ambient temperature, and concentrated under reduced pressure. NMR yields were determined by addition of dimethyl terephthalate as internal standard.

Conditions B: To a flame-dried 8 mL microwave vessel under argon and equipped with a stir bar was added **8** (200 mg, 0.965 mmol, 1.00 equiv), anhydrous toluene (1.93 mL, 0.50 M), and **BSF-07** (1.45 mmol, 1.50 equiv). The reaction was heated to 200 °C for 2.5 hours in a microwave reactor, allowed to cool to ambient temperature, and concentrated under reduced pressure.

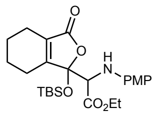

9 [dr = 1.3:1]

Conditions A.

For characterization purposes, a small amount of the crude reaction mixture was purified via flash column chromatography using phosphate buffered silica (pH = 7, ratio of buffered silica to crude mass = 50:1, eluting with a gradient of 1:99 EtOAc:hexane to 1:5 EtOAc:hexane), yielding **9** as a mixture of diastereoisomers.

Appearance: Yellow/brown oil;

R*f*: 0.36 (1:4 EtOAc:hexane);

^{1}H NMR (400 MHz, $CDCl_3$, both diastereoisomers indicated): δ 6.74 (app t, *J* = 9.7 Hz, 2H), 6.60 (app t, *J* = 9.7 Hz, 2H), 4.30 (d, *J* = 11.5 Hz, 1H), 4.21 – 4.08 (m, 2H), 3.89 (d, *J* = 11.6 Hz 1H), 3.72 (s, 3H), 2.52 – 2.08 (m, 4H), 1.85 – 1.48 (m, 4H), 1.28 – 1.16 (m, 3H), 0.88 (s, 9H), 0.13 (s, 1.3H), 0.11 (s, 1.6H), 0.00 (s, 1.2H), –0.04 (s, 1.6H) ppm;

^{13}C NMR (100 MHz, $CDCl_3$, both diastereoisomers indicated): δ 170.7, 170.0, 169.9, 169.8, 162.9, 162.0, 153.6, 153.3, 140.5, 139.9, 129.7, 129.1, 116.5, 115.9, 115.0, 114.9, 106.0, 105.9, 64.9, 63.0, 61.7, 61.5, 55.8, 55.8, 25.9, 25.5, 23.0, 22.1, 21.8, 21.5, 21.5, 21.3, 20.0, 19.9, 18.1, 18.0, 14.2, 14.2, –3.2, –3.4, –3.7, –3.9 ppm;

IR: 2931, 2859, 1777, 1743, 1513, 1468, 1451, 1251, 1195 cm^{-1};

HRMS (ESI): calculated for $[C_{25}H_{37}NO_6Si+H]^+$: 476.2463, found: 476.2462.

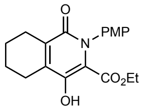

10

Conditions A.

For characterization purposes, a small amount of the crude reaction mixture was purified via trituration with hexane.

Appearance: Pale brown solid;

R*f*: 0.20 (5:95 Et_2O:CH_2Cl_2);

^{1}H NMR (600 MHz, $CDCl_3$): δ 10.81 (s, 1H), 7.09 (d, *J* = 8.8 Hz, 2H), 6.91 (d, *J* = 8.8 Hz, 2H), 3.93 (q, *J* = 7.2 Hz, 2H), 3.82 (s, 3H), 2.62 (app d, *J* = 31.5 Hz, 4H), 1.77 (br s, 4H), 0.78 (t, *J* = 7.2 Hz, 3H) ppm;

^{13}C NMR (100 MHz, $CDCl_3$): δ 166.4, 160.2, 158.9, 150.5, 141.0, 137.6, 134.3, 128.8, 113.9, 112.7, 61.8, 55.7, 24.9, 23.4, 21.6, 21.3, 13.4 ppm;

IR: 2937, 2861, 1729, 1632, 1620, 1512, 1401, 1381, 1298, 1250, 1215, 2083, 1033 cm^{-1};

HRMS (ESI): calculated for $[C_{19}H_{21}NO_5+H]^+$: 344.1492, found: 344.1494.

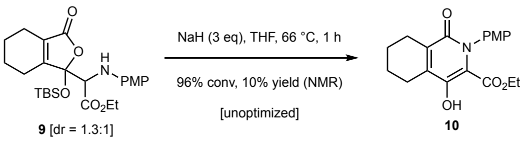

9 [dr = 1.3:1] **10**

To a flame-dried 2-dram vial under argon and equipped with a stir bar was added **9** (31.0 mg, 65.0 µmol, 1.00 equiv) and anhydrous THF (0.342 mL, 0.19 M). The reaction vial was submerged in an ice/water bath and NaH (60% dispersion in mineral oil, 7.80 mg, 0.195 mmol, 3.00 equiv) was added in one portion. The reaction vial was removed from the ice/water bath, sealed, and warmed on a heating mantle at 66 °C for 1 hour. The reaction was cooled to ambient temperature, quenched with saturated aqueous NH_4Cl, and extracted with EtOAc (×3). The combined organics were dried over $MgSO_4$, filtered, and concentrated under reduced pressure. NMR yields were determined by addition of dibromomethane as internal standard.

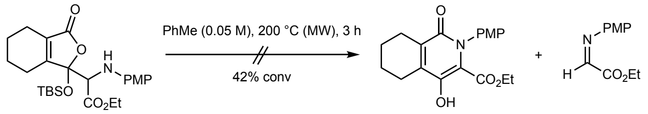

9 [dr = 1.3:1] **10**, 0% **8**, 6% (NMR)

To a flame-dried 8 mL microwave vessel under argon and equipped with a stir bar was added **9** (43.0 mg, 90.0 µmol, 1.00 equiv) and anhydrous toluene (1.80 mL, 0.05 M). The reaction was heated to 200 °C for 3 hours in a microwave reactor, allowed to cool to ambient temperature, and concentrated under reduced pressure. NMR yields were determined by addition of dibromomethane as internal standard.

Note: For the following three reactions, TFA or SiO_2 gave identical results, the only apparent difference being the reaction with TFA is faster.

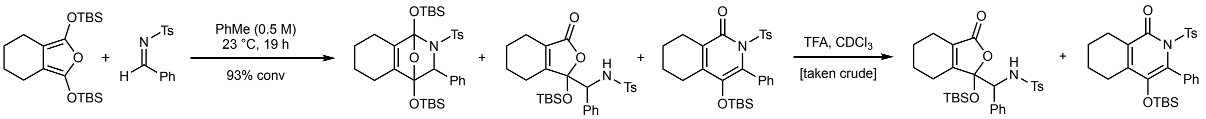

BSF-07 (or **1a**) [1.5 eq] **11** [1.0 eq] tentative [unable to isolate or quantify] **12**, 43% **13**, 0% **12** [dr > 20:1] 73% (NMR) **13**, 0%

To a flame-dried 5 mL one-neck round-bottom flask under argon and equipped with a stir bar was added **11** (50.0 mg, 0.308 mmol, 1.00 equiv), anhydrous toluene (0.386 mL, 0.50 M), and **BSF-07** (0.289 mmol, 1.50 equiv). The reaction was stirred at ambient temperature for 19 hours, then concentrated under reduced pressure (at ambient temperature). NMR yields were determined by addition of dimethyl terephthalate as internal standard. Several drops of dilute TFA in $CDCl_3$ were added to an ^{1}H NMR sample and shaken for ca. 30 seconds. Alternatively, the crude was diluted in ca. 1 mL $CDCl_3$ and treated with TFA (45.0 µL, 0.578 mmol, 3.00 equiv). The solution was stirred at ambient temperature

for 20 minutes, then an ^{1}H NMR sample was taken. ^{1}H NMR yields were determined using dimethyl terephthalate as internal standard. Both methods gave equivalent results.

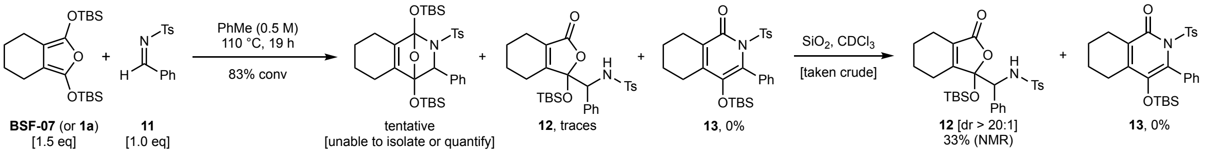

To a flame-dried 2-dram vial under argon and equipped with a stir bar added **11** (80.0 mg, 0.308 mmol, 1.00 equiv), anhydrous toluene (0.617 mL, 0.50 M), and **BSF-07** (0.462 mmol, 1.50 equiv). The reaction vial was sealed, warmed on a heating mantle at 110 °C for 19 hours, cooled to ambient temperature, and concentrated under reduced pressure. NMR yields were determined by addition of dimethyl terephthalate as internal standard. To the crude mixture was added $CDCl_3$ (1.00 mL, 0.31 M) and silica gel (1.62 g/mmol of **11**, 0.500 g). The reaction was stirred at ambient temperature for 2 hours, filtered (washing with CH_2Cl_2), and concentrated under reduced pressure. NMR yields were determined by addition of dibromomethane as internal standard.

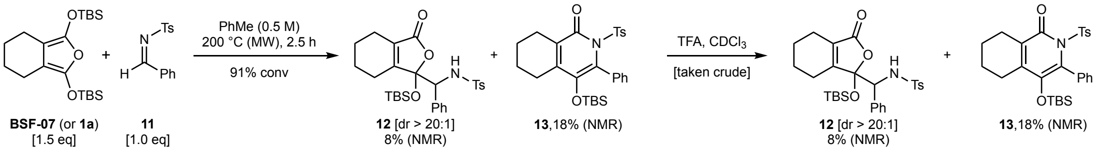

To a flame-dried 8 mL microwave vessel under argon and equipped with a stir bar was added **11** (80.0 mg, 0.308 mmol, 1.00 equiv), anhydrous toluene (0.617 mL, 0.50 M), and **BSF-07** (0.463 mmol, 1.50 equiv). The reaction was heated to 200 °C for 2.5 hours in a microwave reactor, allowed to cool to ambient temperature, and concentrated under reduced pressure. NMR yields were determined by addition of dimethyl terephthalate as internal standard. Several drops of dilute TFA in $CDCl_3$ were added to an ^{1}H NMR sample and shaken for ca. 30 seconds. Alternatively, to the crude mixture was added ca. 1 mL $CDCl_3$ and was treated with TFA (45.0 µL, 0.578 mmol, 3.00 equiv). The solution was stirred at ambient temperature for 20 minutes, then an ^{1}H NMR sample was taken. ^{1}H NMR yields were determined using dimethyl terephthalate as internal standard. Both methods gave equivalent results.

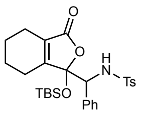

12 [dr > 20:1]

For characterization purposes, a small amount of the crude reaction mixture from the reaction run at 110 °C was purified via flash column chromatography using phosphate buffered silica (pH = 7, ratio of buffered silica to crude mass = 50:1, eluting with hexane 1:4 Et_2O:hexane).

Appearance: White solid;

R*f*: 0.40 (1:1 Et_2O:hexane);

^{1}H NMR (400 MHz, $CDCl_3$): δ 7.36 (d, *J* = 8.4 Hz, 2H), 7.15 – 7.08 (m, 1H), 7.05 (app. d, *J* = 4.4 Hz, 4H), 6.95 (d, *J* = 8.1 Hz, 2H), 5.11 (d, *J* = 10.3 Hz, 1H), 4.47 (d, *J* = 10.3 Hz, 1H), 2.60 – 2.47 (m, 1H), 2.37 – 2.28 (m, 2H), 2.26 (s, 3H), 2.22 – 2.11 (m, 1H), 1.96 – 1.86 (m, 1H), 1.79 – 1.61 (m, 3H), 0.62 (s, 9H), –0.19 (s, 3H), –0.47 (s, 3H) ppm;

^{13}C NMR (100 MHz, $CDCl_3$): δ 171.0, 163.4, 143.2, 137.1, 135.3, 129.5, 129.3, 129.1, 127.9, 127.9, 127.1, 107.0, 61.4, 25.5, 21.9, 21.7, 21.5, 21.5, 20.0, 17.8, –4.1, –4.2 ppm;

IR: 3268, 3065, 3034, 2930, 2886, 2859, 2256, 1777, 1765, 1759, 1691, 1600, 1469, 1441, 1332, 1276, 1249, 1165, 1072, 1052, 1026, 908, 832, 781 cm^{-1};

HRMS (ESI): calculated for $[C_{28}H_{37}NO_5SSi+H]^+$: 528.2234, found: 528.2239.

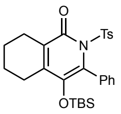

13

For characterization purposes, a small amount of the crude reaction mixture from the reaction run at 200 °C was purified via flash column chromatography using phosphate buffered silica (pH = 7, ratio of buffered silica to crude mass = 50:1, eluting with hexane to 5:95 EtOAc:hexane), followed by trituration with hexane.

Appearance: White solid;

R*f*: 0.26 (5:95 EtOAc:hexane);

^{1}H NMR (400 MHz, $CDCl_3$): δ 7.94 (d, *J* = 8.4 Hz, 2H), 7.53 – 7.44 (m, 2H), 7.32 – 7.26 (m, 5H), 2.83 (t, *J* = 5.8 Hz, 2H), 2.68 (t, *J* = 5.7 Hz, 2H), 2.44 (s, 3H), 1.83 – 1.74 (m, 4H), 0.92 (s, 9H), –0.35 (s, 6H) ppm;

^{13}C NMR (100 MHz, $CDCl_3$): δ 149.4, 146.6, 144.6, 144.0, 143.6, 138.0, 135.4, 129.8, 129.4, 129.12, 128.2, 127.8, 124.4, 26.0, 25.4, 23.6, 21.9, 21.8, 21.7, 18.5, –4.0 ppm;

IR: 3061, 2931, 2886, 2859, 1598, 1550, 1494, 1402, 1378, 1322, 1268, 1191, 1180 cm^{-1};

HRMS (ESI): calculated for $[C_{28}H_{35}NO_4SSi+H]^+$: 510.2129, found: 510.2125.

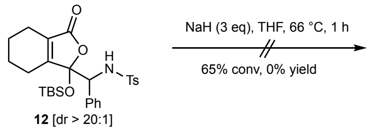

To a flame-dried 2-dram vial under argon and equipped with a stir bar was added **12** (32.0 mg, 61.0 µmol, 1.00 equiv) and anhydrous THF (0.320 mL, 0.19 M). The reaction vial was submerged in an ice/water bath, and NaH (60% dispersion in mineral oil, 7.30 mg, 0.183 mmol, 3.00 equiv) was added in one portion. The reaction vial was removed from the ice/water bath, sealed, and warmed on a heating mantle at 66 °C for 1 hour. The reaction was cooled to ambient temperature, quenched with saturated aqueous NH_4Cl, and extracted with EtOAc (×3). The combined organics were dried over $MgSO_4$, filtered, and concentrated under reduced pressure. NMR yields were determined by addition of dibromomethane as internal standard.

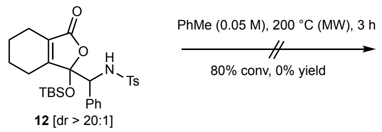

To a flame-dried 8 mL microwave vessel under argon and equipped with a stir bar was added **12** (98.0 mg, 0.190 mmol, 1.00 equiv) and anhydrous toluene (3.70 mL, 0.050 M). The reaction was heated to 200 °C for 3 hours in a microwave reactor, allowed to cool to ambient temperature, and concentrated under reduced pressure. NMR yields were determined by addition of dibromomethane as internal standard.

Rate Studies

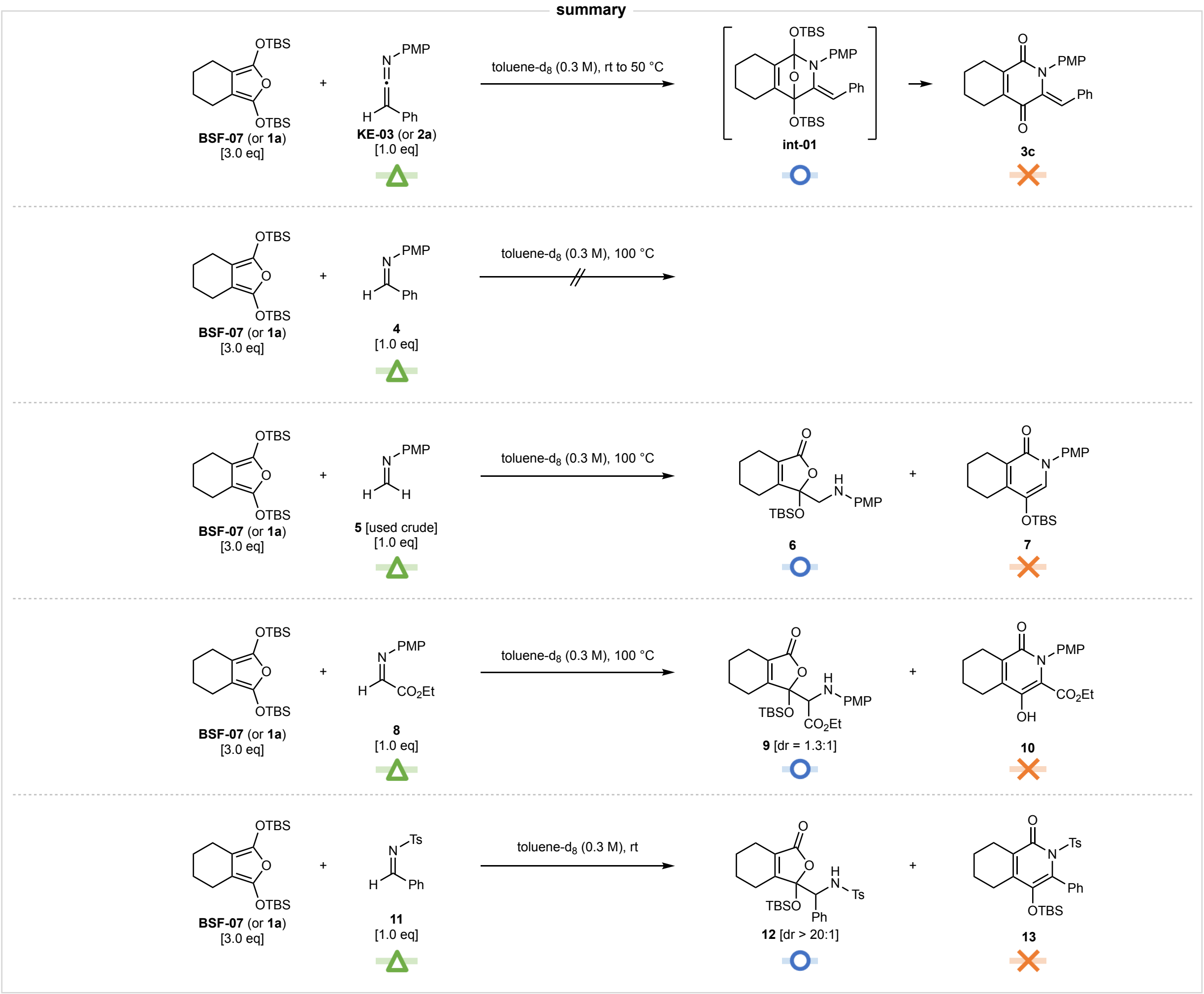

Given the propensity of **BSF-07** to decompose if not appropriately handled (see notes concerning stability and storage earlier in this document), directly before the Diels–Alder reaction its purity was determined by ^{1}H NMR using 1,2-dibromomethane as internal standard. To minimize confusion, its mass was omitted from the following procedures.

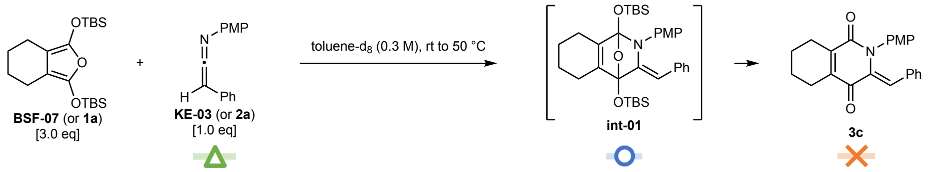

To a flame-dried 5 mL one-neck round-bottom flask under argon and equipped with a stir bar was added freshly prepared ketenimine **KE-03** (1.00 equiv), anhydrous deuterated toluene (0.30 M), and internal standard (dimethyl terephthalate). An initial ^{1}H NMR aliquot was taken, then **BSF-07** (3.00 equiv) was added. The reaction was stirred at ambient temperature for 60 minutes, and ^{1}H NMR aliquots were taken at the times specified below. The reaction was then warmed in an oil bath at 50 °C for a further 120 minutes, and ^{1}H NMR aliquots were taken at the times specified below. In all cases, ^{1}H NMR aliquots were taken using $CDCl_3$ stored over K_2CO_3 to neutralize any trace acid present.

**Temperature** (°C)	**Time** (minutes)	**KE-03** (% by NMR)	**int-01** (% by NMR)	**3c** (% by NMR)
23	– (no **BSF-07**)	100	0	0
23	1	82	12	0
23	5	38	53	0
23	10	15	76	0
23	15	8	83	0
23	20	3	86	0
23	30	0	88	0
23	45	0	88	0
23	60	0	88	0
50	75	0	55	31
50	90	0	28	58
50	105	0	15	71
50	120	0	5	81
50	150	0	0	85
50	180	0	0	85

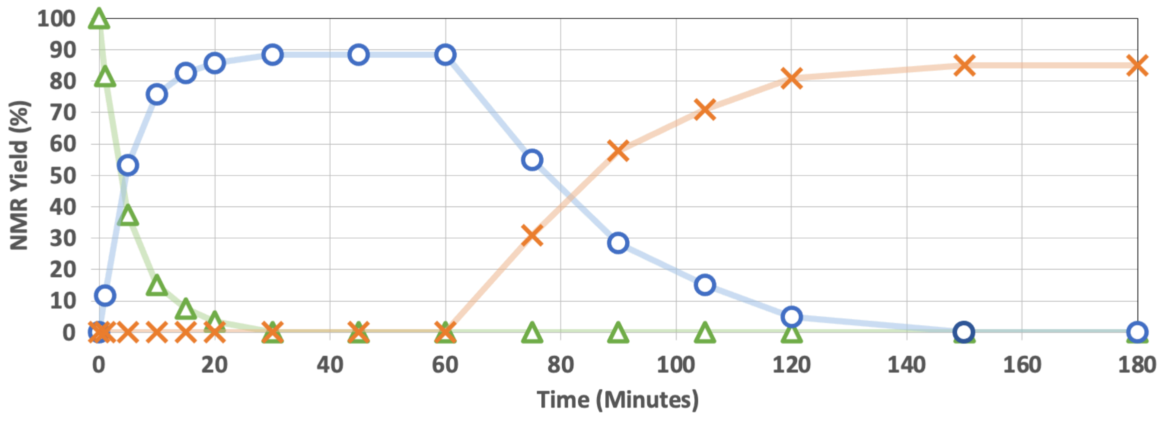

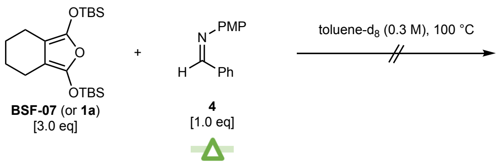

To a flame-dried 5 mL one-neck round-bottom flask under argon and equipped with a stir bar was added imine **4** (1.00 equiv), anhydrous deuterated toluene (0.30 M), and internal standard (dimethyl terephthalate). An initial ^{1}H NMR aliquot was taken, then **BSF-07** (3.00 equiv) was added, and another ^{1}H NMR aliquot immediately taken (t = 0). The reaction was stirred at ambient temperature for 1 hour, and ^{1}H NMR aliquots were taken at the times specified below. The reaction was then warmed in an oil bath at 100 °C for 300 minutes, and ^{1}H NMR aliquots were taken at the times specified below. In all cases, ^{1}H NMR aliquots were taken using $CDCl_3$ stored over K_2CO_3 to neutralize any trace acid present.

**Temperature** (°C)	**Time** (minutes)	**4** (% by NMR)
23	– (no **BSF-07**)	100
23	0	100
23	60	100
100	120	100
100	180	100
100	240	100
100	300	100

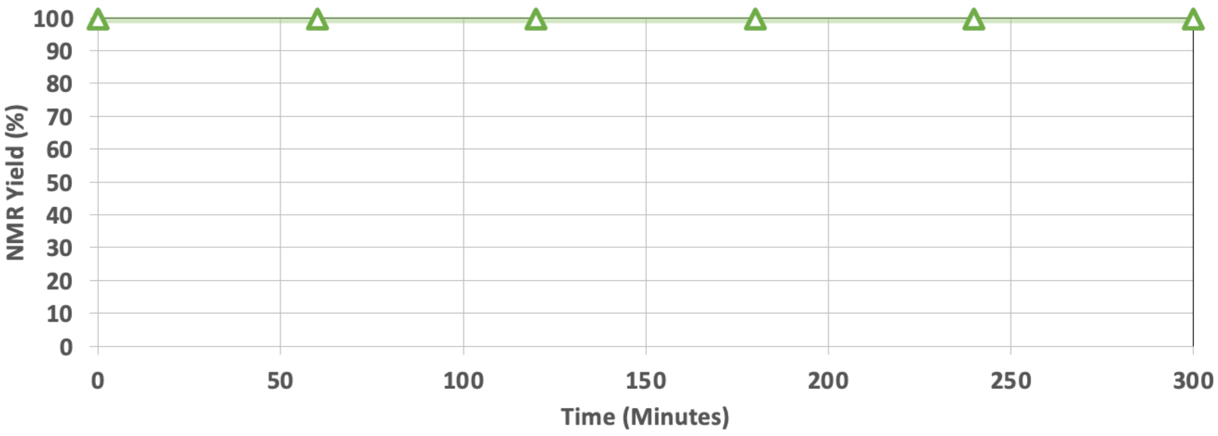

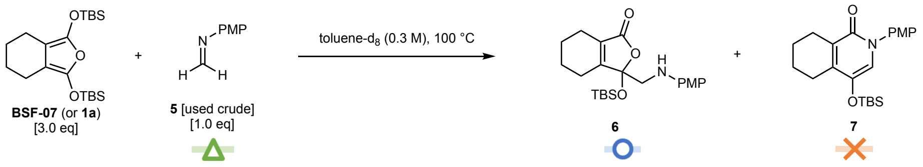

To a flame-dried 5 mL one-neck round-bottom flask under argon and equipped with a stir bar was added imine **5** (1.00 equiv), anhydrous deuterated toluene (0.30 M), and internal standard (dimethyl terephthalate). An initial ^{1}H NMR aliquot was taken, then **BSF-07** (3.00 equiv) was added, and another ^{1}H NMR aliquot immediately taken (t = 0). The reaction was stirred at ambient temperature for 1 hour, and ^{1}H NMR aliquots were taken at the times specified below. The reaction was then warmed in an oil bath at 100 °C for 300 minutes, and ^{1}H NMR aliquots were taken at the times specified below. In all cases, ^{1}H NMR aliquots were taken using $CDCl_3$ stored over K_2CO_3 to neutralize any trace acid present.

**Temperature** (°C)	**Time** (minutes)	**5** (% by NMR)	**6** (% by NMR)	**7** (% by NMR)
23	– (no **BSF-07**)	100	0	0
23	0	100	0	0
23	60	89	7	7
100	120	76	11	13
100	180	64	13	18
100	240	54	16	22
100	300	45	18	24

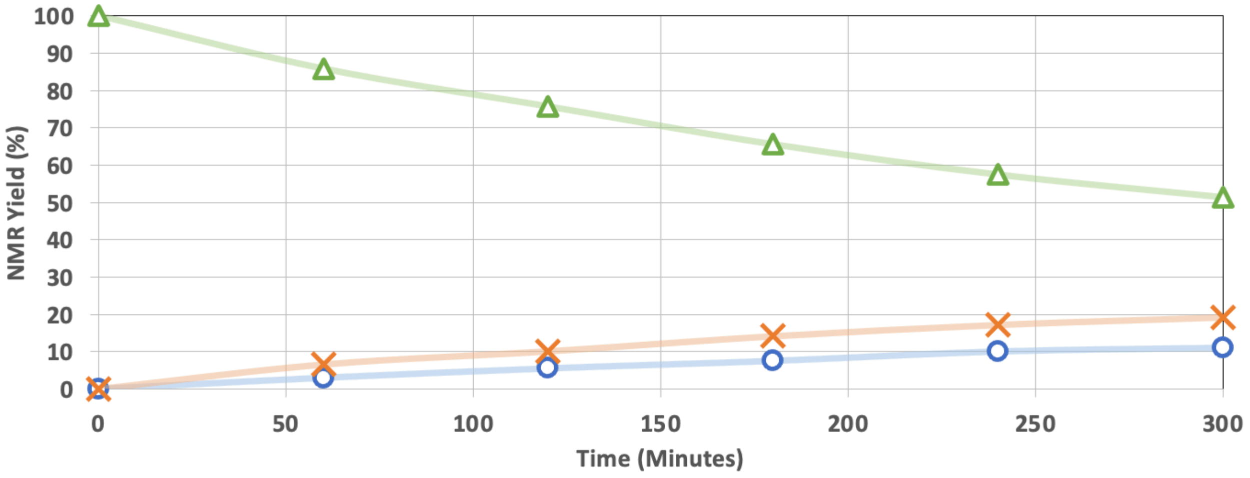

Imine **5** polymerizes when held neat/high concentration. Depolymerization readily occurs upon heating (see inserts below). The rate of depolymerization is faster than the Diels–Alder reaction at 100 °C. This was established by heating a 0.1 M solution of imine **5** in deuterated toluene at 120 °C for 2 hours in the presence of an internal standard (dimethyl terephthalate). Depolymerization conversion = 100%, and NMR yield = 95%. In the rate study above, the sum of the monomer and polymer peaks were combined to measure the quantity of imine **5**.

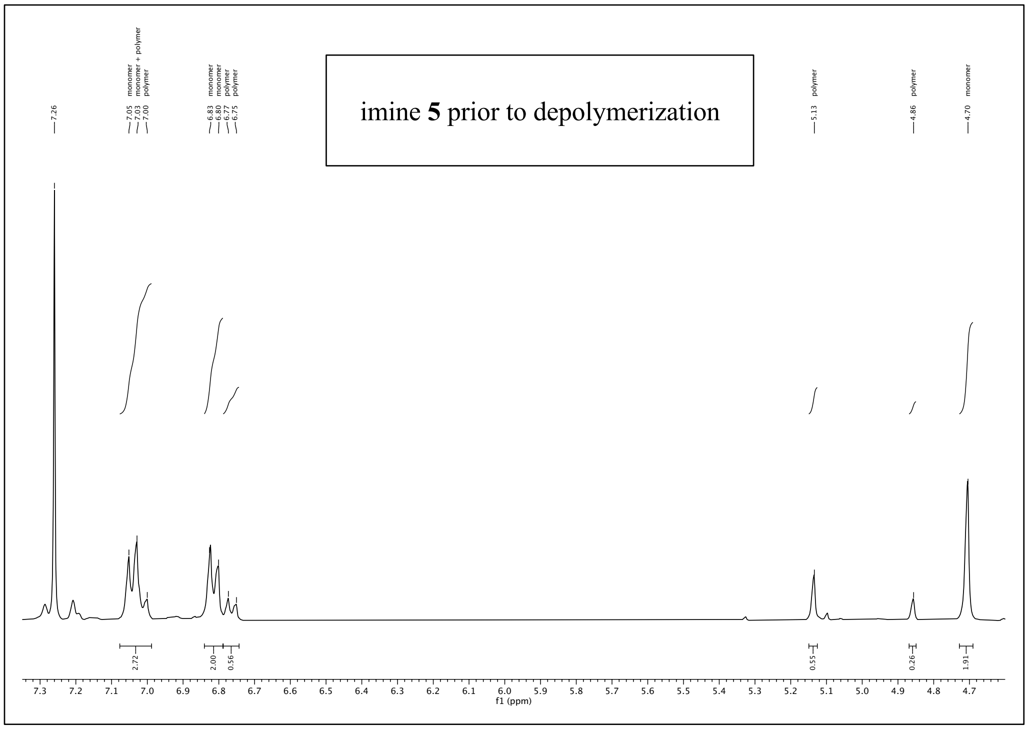

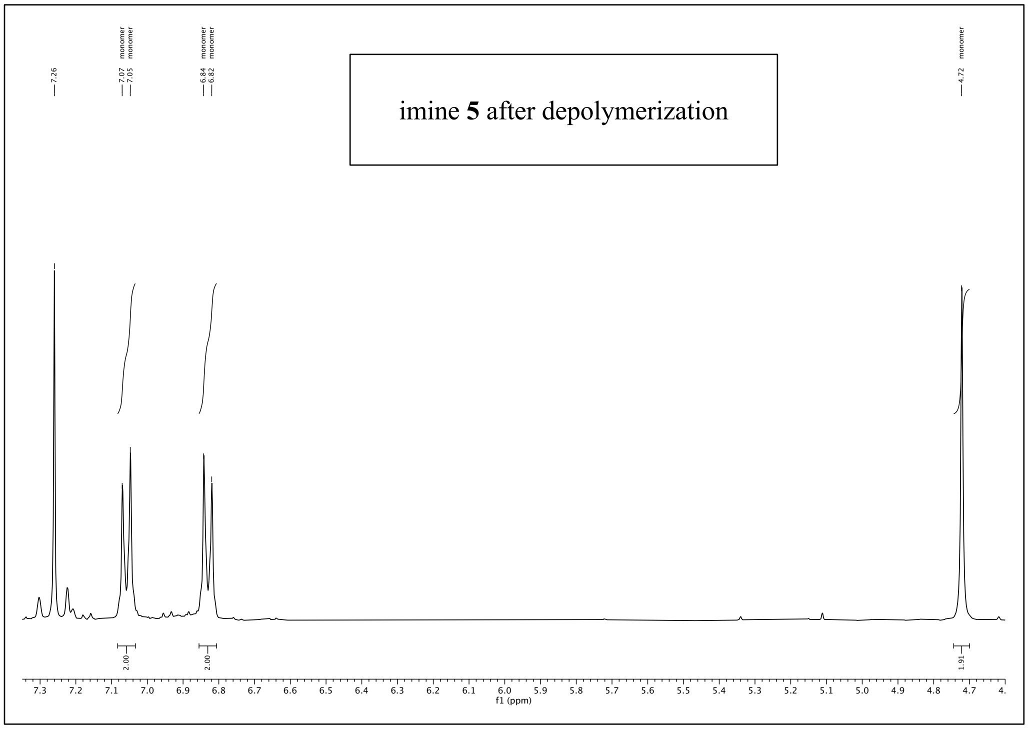

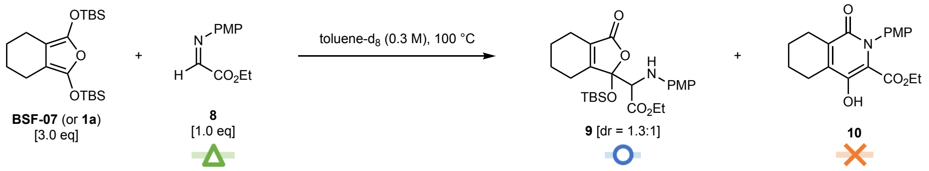

To a flame-dried 5 mL one-neck round-bottom flask under argon and equipped with a stir bar was added imine **8** (1.00 equiv), anhydrous deuterated toluene (0.30 M), and internal standard (dimethyl terephthalate). An initial ^{1}H NMR aliquot was taken, then **BSF-07** (3.00 equiv) was added, and another ^{1}H NMR aliquot immediately taken (t = 0). The reaction was stirred at ambient temperature for 1 hour, and ^{1}H NMR aliquots were taken at the times specified below. The reaction was then warmed in an oil bath at 100 °C for 300 minutes, and ^{1}H NMR aliquots were taken at the times specified below. In all cases, ^{1}H NMR aliquots were taken using $CDCl_3$ stored over K_2CO_3 to neutralize any trace acid present.

**Temperature** (°C)	**Time** (minutes)	**8** (% by NMR)	**9** (% by NMR)	**10** (% by NMR)
23	– (no **BSF-07**)	100	0	0
23	0	100	0	0
23	60	86	3	7
100	120	76	6	10
100	180	66	8	14
100	240	58	10	17
100	300	52	11	19

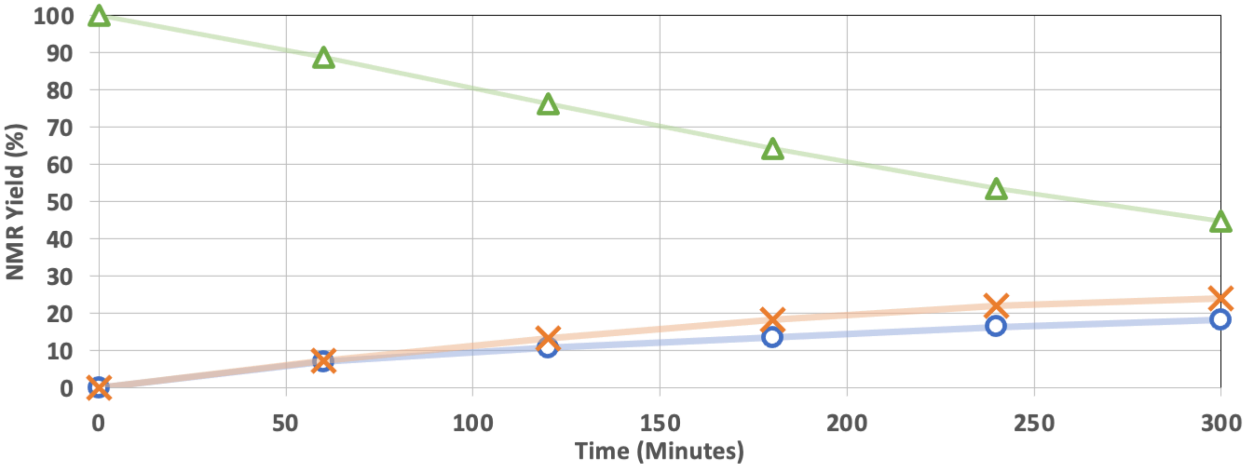

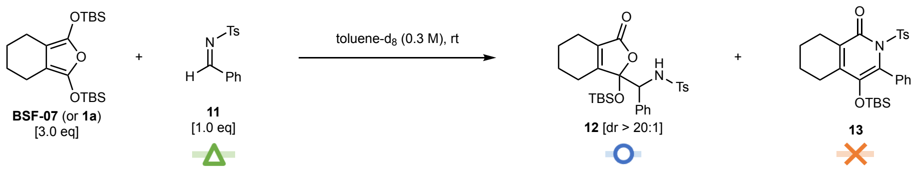

To a flame-dried 5 mL one-neck round-bottom flask under argon and equipped with a stir bar was added imine **11** (1.00 equiv), anhydrous deuterated toluene (0.30 M), and internal standard (dimethyl terephthalate). An initial ^{1}H NMR aliquot was taken, then **BSF-07** (3.00 equiv) was added, and another ^{1}H NMR aliquot immediately taken (t = 0). The reaction was stirred at ambient temperature for 210 minutes, and ^{1}H NMR aliquots were taken at the times specified below. In all cases, ^{1}H NMR aliquots were taken using $CDCl_3$ stored over K_2CO_3 to neutralize any trace acid present. These NMR aliquots were used to measure the consumption of imine **11**. To measure the formation of **12**, ^{1}H NMR aliquots were then acidified with dilute TFA in $CDCl_3$. Compound **13** was not observed before or after acidification.

**Temperature** (°C)	**Time** (minutes)	**11** (% by NMR)	**12** (% by NMR)	**13** (% by NMR)
23	– (no **BSF-07**)	100	0	0
23	1	74	26	0
23	15	57	43	0
23	30	46	52	0
23	45	37	58	0
23	60	31	64	0
23	90	20	74	0
23	120	13	80	0
23	150	8	84	0
23	180	5	87	0
23	210	3	89	0

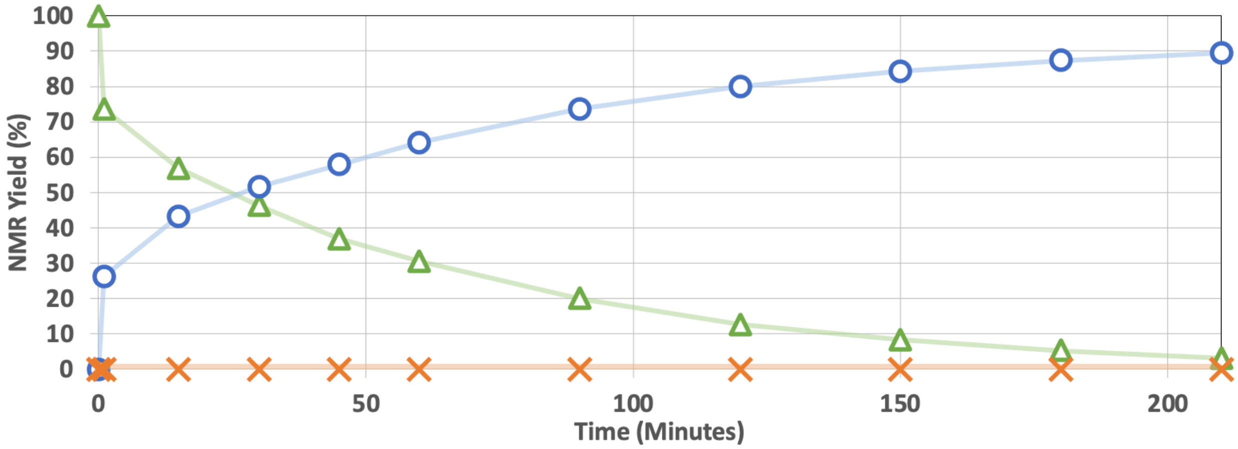

X-Ray Crystallographic Data

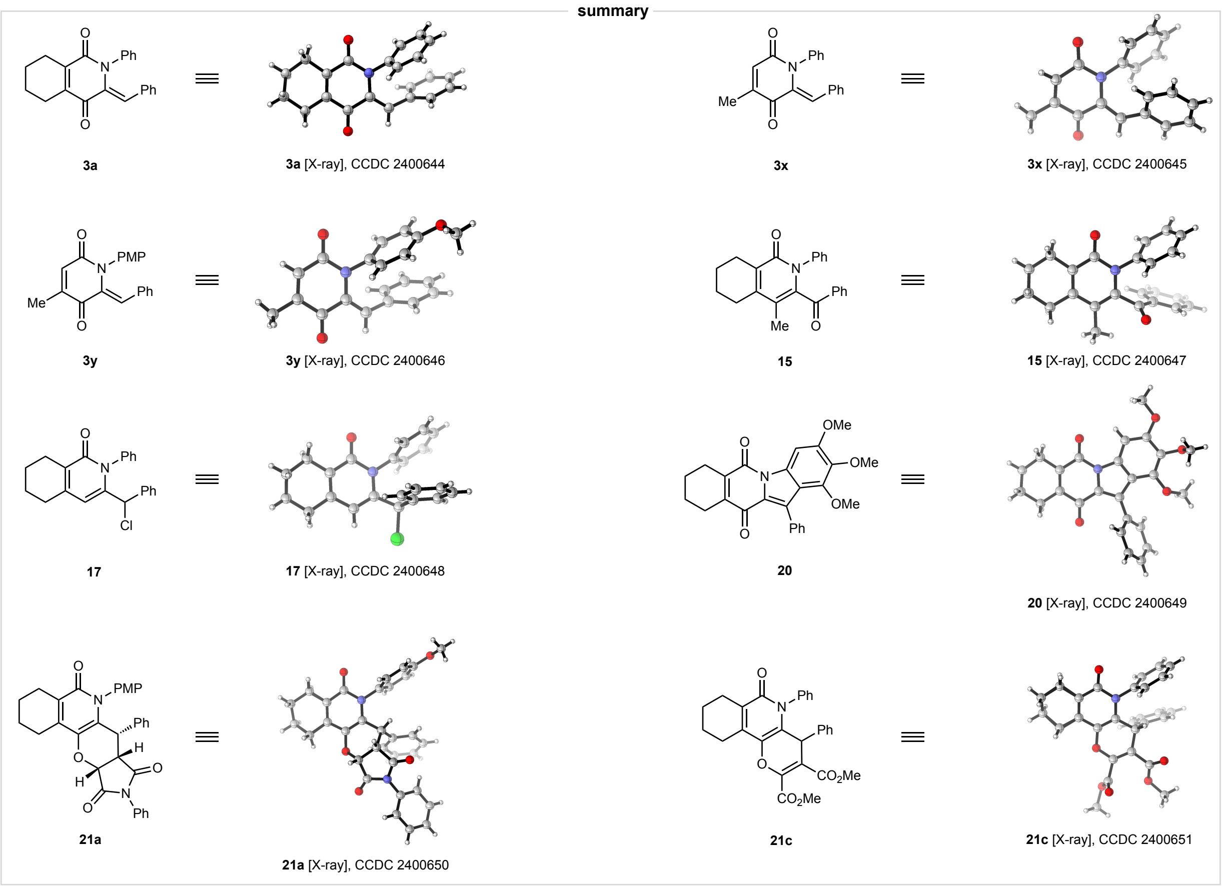

Crystallographic data for **3a**, **3x**, **3y**, **15**, 17, **20**, **21a**, and **21c** can be obtained free of charge from The Cambridge Crystallographic Data Centre (CCDC) via www.ccdc.cam.ac.uk/data_request/cif under CCDC deposition numbers CCDC 2400644−2400651.

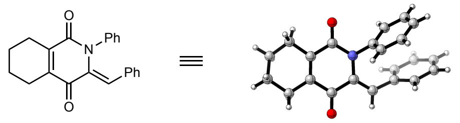

3a **3a** [X-ray], CCDC 2400644

Crystal data and structure refinement for **3a**.

Identification code	j1_a
Empirical formula	$C_{22}H_{19}NO_2$
Formula weight	329.38
Temperature	298(2) K
Wavelength	0.71073 A
Crystal system, space group	Orthorhombic, Fdd2
Unit cell dimensions	
a	23.8406(16) A
b	48.112(3) A
c	5.8363(4) A
α	90 °
β	90 °
γ	90 °
Volume	6694.3(8) A^3
Z, Calculated density	16, 1.307 Mg/m^3
Absorption coefficient	0.084 mm^{-1}
F(000)	2784
Crystal size	0.400 × 0.350 × 0.200 mm
Θ range for data collection	2.406 to 34.350 °
Limiting indices	$-36 \le h \le 37$, $-76 \le k \le 76$, $-9 \le l \le 9$
Reflections collected / unique	54449 / 7007 [R_{int} = 0.0552]
Completeness to θ	25.242 99.5%
Absorption correction	Semi-empirical from equivalents
Max. and min. transmission	0.7470 and 0.6105
Refinement method	Full-matrix least-squares on F^2
Data / restraints / parameters	7007 / 114 / 263
Goodness-of-fit on F^2	1.032
Final R indices [I>2σ(I)]	R_1 = 0.0536, wR_2 = 0.1352
R indices (all data)	R_1 = 0.0728, wR_2 = 0.1476
Absolute structure parameter	−0.1(5)
Extinction coefficient	N/A
Largest diff. peak and hole	0.248 and −0.208 e.$Å^{-3}$

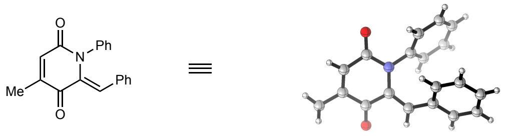

3x | **3x** [X-ray], CCDC 2400645

Crystal data and structure refinement for **3x**.

Identification code	j1_a
Empirical formula	$C_{19}H_{15}NO_2$
Formula weight	289.32
Temperature	297(2) K
Wavelength	0.71073 A
Crystal system, space group	Triclinic, P-1
Unit cell dimensions<br>a<br>b<br>c<br>α<br>β<br>γ	 6.5163(5) A<br>10.6283(9) A<br>5.8363(4) A<br>83.205(3) °<br>77.192(3) °<br>79.904(3) °
Volume	751.44(11) A^3
Z, Calculated density	2, 1.279 Mg/m^3
Absorption coefficient	0.083 mm^{-1}
F(000)	304
Crystal size	0.280 × 0.200 × 0.080 mm
Θ range for data collection	1.848 to 29.496 °
Limiting indices	$-9 \le h \le 9, -14 \le k \le 14, -13 \le l \le 15$
Reflections collected / unique	4160 / 4160 [R_{int} = ?]
Completeness to θ	25.242 99.3%
Absorption correction	Semi-empirical from equivalents
Max. and min. transmission	0.7461 and 0.6419
Refinement method	Full-matrix least-squares on F^2
Data / restraints / parameters	4160 / 0 / 201
Goodness-of-fit on F^2	1.037
Final R indices [I>2σ(I)]	R_1 = 0.0618, wR_2 = 0.1556
R indices (all data)	R_1 = 0.0872, wR_2 = 0.1690
Extinction coefficient	N/A
Largest diff. peak and hole	0.243 and −0.167 e.Å^{-3}

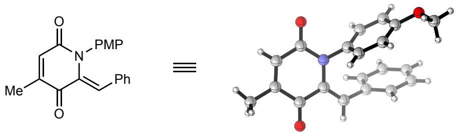

3y **3y** [X-ray], CCDC 2400646

Crystal data and structure refinement for **3y**.

Identification code	j1_a
Empirical formula	$C_{20}H_{17}NO_3$
Formula weight	319.35
Temperature	299(2) K
Wavelength	0.71073 A
Crystal system, space group	Monoclinic, C2/c
Unit cell dimensions<br>a<br>b<br>c<br>α<br>β<br>γ	 31.0863(12) A<br>5.5741(2) A<br>22.4353(9) A<br>90 °<br>124.9320(10) °<br>90 °
Volume	3187.1(2) A^3
Z, Calculated density	8, 1.331 Mg/m^3
Absorption coefficient	0.090 mm^{-1}
F(000)	1344
Crystal size	0.350 × 0.140 × 0.120 mm
Θ range for data collection	1.848 to 29.496 °
Limiting indices	$-44 \le h \le 44$, $-7 \le k \le 7$, $-32 \le l \le 32$
Reflections collected / unique	51816 / 4840 [R_{int} = 0.0511]
Completeness to θ	25.242 99.9%
Absorption correction	Semi-empirical from equivalents
Max. and min. transmission	0.7462 and 0.6508
Refinement method	Full-matrix least-squares on F^2
Data / restraints / parameters	4840 / 0 / 218
Goodness-of-fit on F^2	1.041
Final R indices [I>2σ(I)]	R_1 = 0.0515, wR_2 = 0.1294
R indices (all data)	R_1 = 0.0751, wR_2 = 0.1429
Extinction coefficient	N/A
Largest diff. peak and hole	0.256 and −0.227 e.$Å^{-3}$

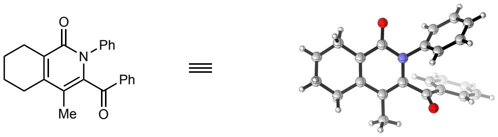

15 — **15** [X-ray], CCDC 2400647

Crystal data and structure refinement for **15**.

Identification code	j1_a
Empirical formula	$C_{23}H_{21}NO_2$
Formula weight	343.41
Temperature	299(2) K
Wavelength	0.71073 A
Crystal system, space group	Monoclinic, P2(1)
Unit cell dimensions	
a	12.0753(5) A
b	5.9738(2) A
c	12.4029(5) A
α	90 °
β	96.8130(10) °
γ	90 °
Volume	888.37(6) A^3
Z, Calculated density	2, 1.284 Mg/m^3
Absorption coefficient	0.082 mm^{-1}
F(000)	364
Crystal size	0.380 × 0.240 × 0.120 mm
Θ range for data collection	1.698 to 37.783 °
Limiting indices	$-20 \le h \le 20, -10 \le k \le 10, -21 \le l \le 21$
Reflections collected / unique	52931 / 9517 [R_{int} = 0.0815]
Completeness to θ	25.242 99.8%
Absorption correction	Semi-empirical from equivalents
Max. and min. transmission	0.7476 and 0.5079
Refinement method	Full-matrix least-squares on F^2
Data / restraints / parameters	9517 / 1 / 236
Goodness-of-fit on F^2	1.027
Final R indices [I>2σ(I)]	R_1 = 0.0708, wR_2 = 0.1674
R indices (all data)	R_1 = 0.1400, wR_2 = 0.1994
Absolute structure parameter	–0.1(8)
Extinction coefficient	N/A
Largest diff. peak and hole	0.353 and −0.190 e.$Å^{-3}$

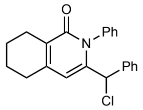

17

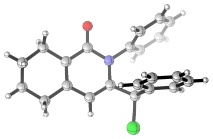

17 [X-ray], CCDC 2400648

Crystal data and structure refinement for **17**.

Identification code	j1_a
Empirical formula	$C_{22}H_{20}ClNO$
Formula weight	349.84
Temperature	297(2) K
Wavelength	0.71073 A
Crystal system, space group	Monoclinic, P2(1)
Unit cell dimensions<br>a<br>b<br>c<br>α<br>β<br>γ	 11.9669(5) A<br>6.0991(3) A<br>12.0584(6) A<br>90 °<br>97.004(2) °<br>90 °
Volume	873.54(7) A^3
Z, Calculated density	2, 1.330 Mg/m^3
Absorption coefficient	0.228 mm^{-1}
F(000)	368
Crystal size	0.380 × 0.250 × 0.060 mm
Θ range for data collection	1.701 to 33.724 °
Limiting indices	$-18 \leq h \leq 18, -9 \leq k \leq 9, -18 \leq l \leq 18$
Reflections collected / unique	37622 / 6967 [R_{int} = 0.0866]
Completeness to θ	25.242 99.8%
Absorption correction	Semi-empirical from equivalents
Max. and min. transmission	0.7469 and 0.5825
Refinement method	Full-matrix least-squares on F^2
Data / restraints / parameters	6967 / 1 / 226
Goodness-of-fit on F^2	1.015
Final R indices [I>2σ(I)]	R_1 = 0.0594, wR_2 = 0.1309
R indices (all data)	R_1 = 0.1069, wR_2 = 0.1514
Absolute structure parameter	0.01(4)
Extinction coefficient	N/A
Largest diff. peak and hole	0.330 and −0.266 e.$Å^{-3}$

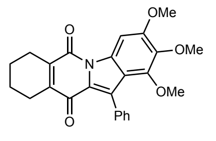

20

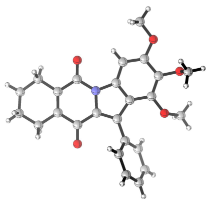

20 [X-ray], CCDC 2400649

Crystal data and structure refinement for **20**.

Identification code	j1_a
Empirical formula	$C_{25}H_{23}NO_5$
Formula weight	417.44
Temperature	298(2) K
Wavelength	0.71073 A
Crystal system, space group	Triclinic, P-1
Unit cell dimensions	
a	8.3839(5) A
b	12.2449(7) A
c	42.242(3) A
α	96.764(2) °
β	92.275(2) °
γ	104.948(2) °
Volume	4149.5(4) A^3
Z, Calculated density	8, 1.336 Mg/m^3
Absorption coefficient	0.093 mm^{-1}
F(000)	1760
Crystal size	0.450 × 0.400 × 0.250 mm
Θ range for data collection	1.737 to 25.250 °
Limiting indices	$-10 \le h \le 10$, $-14 \le k \le 14$, $-50 \le l \le 50$
Reflections collected / unique	90724 / 14938 [R_{int} = 0.0962]
Completeness to θ	25.242 99.4%
Absorption correction	Semi-empirical from equivalents
Max. and min. transmission	0.7461 and 0.3645
Refinement method	Full-matrix least-squares on F^2
Data / restraints / parameters	14938 / 351 / 1228
Goodness-of-fit on F^2	1.072
Final R indices [I>2σ(I)]	R_1 = 0.0802, wR_2 = 0.2172
R indices (all data)	R_1 = 0.1233, wR_2 = 0.2478
Extinction coefficient	N/A
Largest diff. peak and hole	0.717 and −0.437 e.$Å^{-3}$

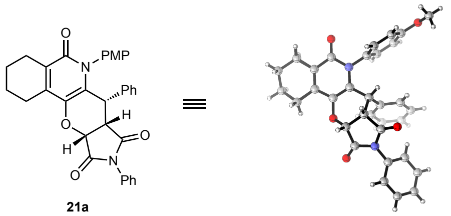

21a

21a [X-ray], CCDC 2400650

Crystal data and structure refinement for **21a**.

Identification code	j1_a
Empirical formula	$C_{33}H_{28}N_2O_5$
Formula weight	532.57
Temperature	298(2) K
Wavelength	0.71073 A
Crystal system, space group	Monoclinic, P2(1)/c
Unit cell dimensions	
a	12.8418(9) A
b	16.3956(11) A
c	12.7462(9) A
α	90 °
β	102.586(3) °
γ	90 °
Volume	2619.2(3) A^3
Z, Calculated density	4, 1.351 Mg/m^3
Absorption coefficient	0.091 mm^{-1}
F(000)	1120
Crystal size	0.450 × 0.340 × 0.200 mm
Θ range for data collection	2.045 to 34.337 °
Limiting indices	$-20 \leq h \leq 20$, $-26 \leq k \leq 26$, $-20 \leq l \leq 20$
Reflections collected / unique	115855 / 10959 [R_{int} = 0.0997]
Completeness to θ	25.242 100.0%
Absorption correction	Semi-empirical from equivalents
Max. and min. transmission	0.7470 and 0.5641
Refinement method	Full-matrix least-squares on F^2
Data / restraints / parameters	10959 / 119 / 398
Goodness-of-fit on F^2	1.004
Final R indices [I>2σ(I)]	R_1 = 0.0675, wR_2 = 0.1559
R indices (all data)	R_1 = 0.1352, wR_2 = 0.1937
Extinction coefficient	N/A
Largest diff. peak and hole	0.334 and −0.254 e.$Å^{-3}$

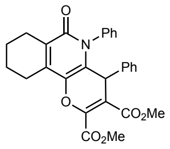

21c

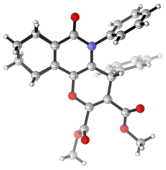

21c [X-ray], CCDC 2400651

Crystal data and structure refinement for **21c**.

Identification code	j1_a
Empirical formula	$C_{28}H_{25}NO_6$
Formula weight	471.49
Temperature	298(2) K
Wavelength	0.71073 A
Crystal system, space group	Triclinic, P-1
Unit cell dimensions<br>a<br>b<br>c<br>α<br>β<br>γ	 9.4940(5) A<br>9.9963(5) A<br>13.5513(8) A<br>74.994(2) °<br>82.333(2) °<br>78.536(2) °
Volume	1212.87(11) A^3
Z, Calculated density	2, 1.291 Mg/m^3
Absorption coefficient	0.091 mm^{-1}
F(000)	496
Crystal size	0.450 × 0.340 × 0.120 mm
Θ range for data collection	2.197 to 33.140 °
Limiting indices	−14 ≤ h ≤ 14, −15 ≤ k ≤ 15, −20 ≤ l ≤ 20
Reflections collected / unique	50982 / 9254 [R_{int} = 0.0925]
Completeness to θ	25.242 99.8%
Absorption correction	Semi-empirical from equivalents
Max. and min. transmission	0.7468 and 0.4078
Refinement method	Full-matrix least-squares on F^2
Data / restraints / parameters	9254 / 114 / 353
Goodness-of-fit on F^2	1.062
Final R indices [I>2σ(I)]	R_1 = 0.0804, wR_2 = 0.2068
R indices (all data)	R_1 = 0.1412, wR_2 = 0.2453
Extinction coefficient	N/A
Largest diff. peak and hole	0.446 and −0.355 e.$Å^{-3}$

Calculations

[3,3] Sigmatropic Rearrangements

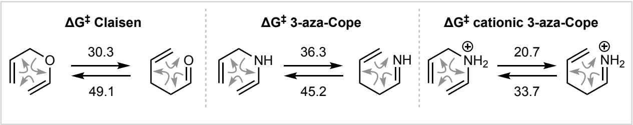

CCSD(T)/cc-pVTZ//M06-2X/6-311+G(d,p)

All calculations were carried out using Gaussian 16 Rev. A.03.[68] Optimized geometries and frequencies were computed at the M06-2X/6-311+G(d,p) level of theory followed by CCSD(T)/cc-pVTZ single point energies. All stationary points were confirmed to be energy minima (zero imaginary frequencies) or first-order saddle points (one imaginary frequency). The forward and reverse free energy barriers, $\Delta G^{\ddagger}$, were calculated under the quasi-RRHO approximation of Grimme and co-workers[69] using AaronTools[70] by combining the CCSD(T)/cc-pVTZ electronic energies with thermal and entropic corrections from the M06-2X/6-311+G(d,p) frequencies.

Cartesian Coordinates

3-(Vinyloxy)prop-1-ene

0 1

C 1.326598 -0.316264 -0.249395
C 2.562873 -0.610835 0.130625
O 0.716001 0.843548 0.108317
C -0.648186 0.955109 -0.277801
C -1.528473 0.008384 0.484204
C -2.381597 -0.829168 -0.091862
H 0.734795 -0.980430 -0.875599
H 3.016810 -1.535013 -0.195597
H 3.128380 0.062340 0.761589
H -0.916723 1.989882 -0.059318
H -0.751992 0.792505 -1.357448
H -1.432291 0.044603 1.566319
H -3.016700 -1.484998 0.491248
H -2.477574 -0.880636 -1.172356

Claisen Rearrangement Transition State

0 1

C 1.215804 -0.579377 0.252971
C 1.471876 0.661540 -0.294775
O 0.357748 -1.382391 -0.255275
C -1.330655 -0.780522 0.183694
C -1.306470 0.533972 -0.295753
C -0.474912 1.444998 0.328289
H 1.582783 -0.790571 1.266589
H 2.211156 1.306180 0.168621
H 1.252451 0.834759 -1.339760
H -1.897208 -1.537806 -0.342408
H -1.234077 -0.950402 1.250682
H -1.612477 0.715924 -1.320269
H -0.338659 2.440896 -0.078703
H -0.279813 1.356482 1.390890

Pent-4-enal

0 1

C 1.404395 -0.295900 0.370448
C 0.798972 0.995532 -0.123356
O 2.208766 -0.947340 -0.236129
C -2.162103 -0.952063 0.154699
C -1.476948 -0.003489 -0.473043

C	-0.696629	1.081278	0.212783
H	1.061203	-0.622295	1.375456
H	1.336756	1.812772	0.371907
H	0.983374	1.078001	-1.196563
H	-2.692978	-1.723247	-0.390025
H	-2.215223	-0.985719	1.238697
H	-1.439381	-0.003983	-1.560913
H	-1.070509	2.062136	-0.094346
H	-0.839492	1.008911	1.295636

***N*-vinylprop-2-en-1-amine**

0 1

C	1.753938	0.092423	-0.173954
C	1.435032	1.358206	0.116736
C	-0.396845	-0.903804	0.475889
C	-1.405760	-0.225117	-0.414602
C	-2.280208	0.679256	0.006033
H	2.760568	-0.150737	-0.500144
H	2.187260	2.127589	0.018349
H	0.440429	1.656995	0.417644
H	-0.740459	-1.922575	0.675896
H	-0.339849	-0.377059	1.439429
H	-1.394243	-0.542058	-1.454397
H	-3.012533	1.114587	-0.662670
H	-2.288905	1.017974	1.038066
N	0.910988	-1.005387	-0.142365
H	1.373881	-1.892786	-0.036223

Aza-Cope Rearrangement Transition State

0 1

C	1.381566	-0.209679	0.217312
C	1.147983	1.049789	-0.317152
C	-1.090055	-1.083480	0.152543
C	-1.418991	0.212123	-0.267525
C	-0.811724	1.293019	0.356058
H	1.889694	-0.273542	1.182573
H	1.670639	1.900476	0.105587
H	0.881357	1.131849	-1.361962
H	-1.499674	-1.926317	-0.391628
H	-0.988788	-1.254376	1.220355
H	-1.791044	0.342261	-1.278759
H	-0.989066	2.299670	-0.007742
H	-0.584269	1.236587	1.414697
N	0.741998	-1.274698	-0.284796
H	0.964491	-2.104350	0.263037

Pent-4-en-1-imine

0 1

C	1.430672	-0.194649	0.411782
C	0.789462	1.050261	-0.125951
C	-2.175650	-0.958828	0.180762
C	-1.440631	-0.067847	-0.474155
C	-0.720287	1.083100	0.166023
H	1.224644	-0.412991	1.469206
H	1.263112	1.920027	0.342436
H	0.984356	1.099423	-1.199588
H	-2.666750	-1.776593	-0.332710
H	-2.311987	-0.896157	1.256210
H	-1.317244	-0.167171	-1.551164
H	-1.125217	2.027452	-0.211058
H	-0.894134	1.069613	1.246874
N	2.149390	-0.942427	-0.313348
H	2.496103	-1.738839	0.222468

_N_-vinylprop-2-en-1-aminium

1 1

C	-1.492074	-0.068583	0.436178
C	-1.857542	-1.135398	-0.244808
C	0.733897	1.091288	0.212106
C	1.497449	-0.019141	-0.430628
C	1.959846	-1.058686	0.252983
H	-1.666642	0.097489	1.491172
H	-2.393654	-1.931989	0.254611
H	-1.644984	-1.258085	-1.301658
H	1.086808	2.075116	-0.100966
H	0.744297	1.029807	1.300390
H	1.697584	0.076447	-1.494836
H	2.535832	-1.837458	-0.231591
H	1.796019	-1.157708	1.321506
N	-0.744811	1.028601	-0.200119
H	-1.194238	1.921590	0.026031
H	-0.796800	0.927701	-1.218821

Cationic Aza-Cope Rearrangement Transition State

1 1

C	1.409309	-0.119468	0.318749
C	1.295490	1.077575	-0.305735
C	-1.236647	-1.058327	0.206312
C	-1.451007	0.208024	-0.339308
C	-0.976908	1.311845	0.326358
H	1.784779	-0.206097	1.331647
H	1.739119	1.956525	0.144948
H	1.014823	1.147510	-1.349662
H	-1.591835	-1.948530	-0.302564
H	-1.082900	-1.158342	1.275575
H	-1.795768	0.298589	-1.363991
H	-1.056953	2.305766	-0.100840
H	-0.749647	1.263525	1.385099
N	0.808452	-1.247199	-0.185664
H	1.075807	-2.130832	0.239306
H	0.761990	-1.315610	-1.198131

Pent-4-en-1-iminium

1 1

C	1.560180	-0.125195	0.281363
C	0.931264	1.093871	-0.253737
C	-1.977779	-0.870841	0.236979
C	-1.432797	0.177945	-0.374028
C	-0.537483	1.173276	0.298493
H	2.206350	-0.079226	1.155688
H	1.501544	1.965062	0.064415
H	0.898012	1.065815	-1.345657
H	-2.643510	-1.546770	-0.286521
H	-1.812206	-1.061828	1.293573
H	-1.649122	0.361397	-1.425104
H	-0.873604	2.191851	0.094543
H	-0.543647	1.039780	1.382894
N	1.322253	-1.295953	-0.189297
H	1.714713	-2.133957	0.230796
H	0.685386	-1.424793	-0.973970

LUMO energies and Diels–Alder Reactions

All calculations were carried out by using Gaussian 16 Rev. A.03.[68] Given the benchmark study done by Martin Head-Gordon and co-workers recently,[71] geometry optimizations and frequency calculations were performed at the ωB97X-D/6-31+G(d) level of theory.[72] Frequency calculations were conducted at the same level of theory to confirm the presence of local minima (no imaginary frequencies) and transition states (one imaginary frequency) on the PES. Subsequent single point energies were computed at the ωB97X-D/def2TZVP level. Solvent effect was modelled by employing the CPCM model.[73] Conformational searches were conducted using the CREST conformer-rotamer ensemble sampling tool version 2.10.2 with xtb version 6.3.3 to ensure the substrate showed are lowest energy conformers.[74] Intrinsic reaction coordinate (IRC) calculations were performed to verify that the saddle points found were true TSs connecting the reactants and the products.[75] All thermodynamic quantities (1 mol/L, 298.15 K) were computed in the GoodVibes code[76] with quasiharmonic corrections.[69, 77] 3D renderings of stationary points and X-Ray structures were generated using CYLview20 (http://www.cylview.org). Frontier Molecular Orbitals (FMOs) were plotted using the SEQCROW bundle[78] for UCSF ChimeraX.[79]

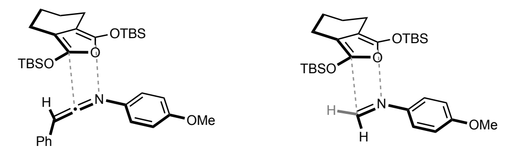

TS-SI-1, *endo*-lone pair
ΔG‡ = 23.4 kcal·mol^{-1}

TS-SI-2, *endo*-lone pair
ΔG‡ = 28.1 kcal·mol^{-1}

Ketenimine 2a

N	0.73637	-0.95426	1.10242
C	4.03431	1.14883	-0.46234
C	1.82414	-0.24006	0.53493
C	2.84730	1.08237	-1.20576
C	4.10891	0.52063	0.78198
C	1.75363	0.39469	-0.71262
C	2.99901	-0.16729	1.27256
C	-2.67588	-0.57768	0.11286
C	-3.87841	-0.96018	-0.49474
C	-2.63098	0.64944	0.79087
C	-5.00341	-0.14258	-0.42762
C	-3.75536	1.46409	0.85707
C	-1.50477	-1.46571	0.02117
C	-4.94839	1.07411	0.24807
C	-0.32310	-1.18774	0.53709
H	2.80803	1.58143	-2.16876
H	5.01282	0.55746	1.37910
H	0.83469	0.34776	-1.29162
H	3.04223	-0.65748	2.24027
H	-3.93160	-1.90865	-1.02406
H	-1.70860	0.96618	1.27246
H	-5.92613	-0.45961	-0.90584
H	-3.69977	2.41040	1.38811
H	-1.60796	-2.40777	-0.51215
H	-5.82521	1.71282	0.30078

O	5.04736	1.84595	-1.03483
C	6.26895	1.95160	-0.33402
H	6.71747	0.96447	-0.16480
H	6.92728	2.54368	-0.97024
H	6.13366	2.46424	0.62678

4-Methoxy-*N*-benzylideneaniline (4)

O	-4.75804	0.47930	-0.10541
N	0.71749	1.52272	0.01010
C	1.62417	0.62774	-0.00990
C	1.53518	-0.85163	-0.13681
C	2.57467	-1.60554	0.42109
C	0.51268	-1.51717	-0.82710
C	-0.65804	1.20413	0.00679
C	2.57836	-2.99450	0.33143
C	0.52680	-2.90379	-0.93267
C	-1.25148	0.49887	1.05185
C	-1.46950	1.68748	-1.02693
C	1.55148	-3.64685	-0.34681
C	-2.62228	0.23859	1.05438
C	-2.82844	1.41482	-1.04294
C	-3.41560	0.68652	-0.00251
C	-5.39986	-0.23991	0.92356
H	3.38614	-1.09613	0.93648
H	-0.29091	-0.95353	-1.28786
H	3.38587	-3.56460	0.78158
H	-0.26786	-3.40697	-1.47586
H	-0.63622	0.14014	1.87209
H	-1.01759	2.26802	-1.82590
H	1.55333	-4.73017	-0.42734
H	-3.04852	-0.31305	1.88498
H	-3.46013	1.77200	-1.85031
H	-6.45270	-0.28931	0.64323
H	-5.30541	0.27310	1.88965
H	-4.99912	-1.25854	1.01029
H	2.64831	1.00294	0.07893

formaldimine 5

O	-2.68820	0.59830	0.00040
N	2.73230	-0.59060	0.00030
C	-1.35860	0.30690	0.00000
C	1.36650	-0.29060	-0.00110
C	-0.41850	1.33730	-0.00030
C	-0.93600	-1.02260	0.00000
C	0.94400	1.03860	-0.00100
C	0.42650	-1.32130	-0.00060
C	-3.60360	-0.47890	0.00080
C	3.53560	0.42310	0.00160
H	-0.74400	2.37420	-0.00030
H	-1.61520	-1.86850	0.00030
H	1.57760	1.91410	-0.00160
H	0.74760	-2.35970	-0.00030
H	-4.61270	-0.05360	0.00110
H	-3.51420	-1.08640	0.90790
H	-3.51480	-1.08660	-0.90630
H	4.61160	0.17680	0.00260
H	3.34910	1.50400	0.00230

ester-imine 8

O	4.83063	0.33404	-0.14534
O	-3.33754	-0.65420	0.77504
O	-3.66986	1.55705	0.46055
N	-0.67075	-0.26040	0.44586
C	0.71885	-0.07304	0.30973

C	3.49499	0.13321	-0.02312
C	1.39959	1.09107	0.70243
C	1.45477	-1.13603	-0.21188
C	2.77038	1.19097	0.54149
C	2.83128	-1.03686	-0.40014
C	-4.75187	-0.86357	0.94038
C	-5.44805	-1.00880	-0.40077
C	-1.45838	0.73130	0.35848
C	-2.93826	0.59037	0.53423
C	5.61902	-0.70467	-0.68816
H	0.85988	1.91012	1.16946
H	0.92952	-2.04638	-0.48413
H	3.30803	2.07954	0.85669
H	3.36812	-1.87731	-0.82511
H	-4.81522	-1.78351	1.52453
H	-5.16890	-0.03503	1.51871
H	-6.50542	-1.24593	-0.24033
H	-5.38869	-0.07924	-0.97333
H	-4.99726	-1.81765	-0.98396
H	-1.14759	1.75867	0.13698
H	5.31387	-0.94089	-1.71538
H	5.56497	-1.60985	-0.07026
H	6.64299	-0.33027	-0.69234

tosyl-imine 11

S	0.78507	-1.72924	-0.35796
O	0.63594	-2.01287	-1.78480
O	1.02178	-2.81055	0.58446
N	-0.56569	-0.88002	0.23562
C	2.04746	-0.49584	-0.15924
C	4.00163	1.46237	0.17428
C	2.62310	-0.31235	1.09605
C	2.43105	0.27698	-1.24944
C	3.59483	0.66730	1.25301
C	3.40770	1.25289	-1.07324
C	5.07690	2.50259	0.35856
C	-2.77523	-0.05015	-0.21135
C	-1.53449	-0.73341	-0.58780
C	-3.78158	0.09616	-1.17191
C	-2.96898	0.45534	1.08114
C	-4.97056	0.74387	-0.84912
C	-4.15561	1.10100	1.40053
C	-5.15654	1.24617	0.43677
H	2.31598	-0.93563	1.92977
H	1.97884	0.10711	-2.22136
H	4.04984	0.81570	2.22927
H	3.71503	1.85739	-1.92273
H	4.91763	3.07820	1.27624
H	6.06289	2.02919	0.43439
H	5.10632	3.20214	-0.48187
H	-1.46505	-1.11528	-1.61383
H	-3.63155	-0.29961	-2.17367
H	-2.18255	0.33121	1.81905
H	-5.74917	0.85511	-1.59762
H	-4.30619	1.49194	2.40238
H	-6.08388	1.75120	0.69173

cyclohexyl-furyl derivative 1a

C	-0.95923	2.23341	0.47569
C	0.48197	2.33868	0.41850
C	1.19627	3.62628	0.13471
C	0.22726	4.62845	-0.51365
C	-1.10162	4.70478	0.25144
C	-1.87697	3.37957	0.17248

C	-1.22715	0.94457	0.78745
C	0.95689	1.10377	0.70201
H	1.57978	4.05499	1.07156
H	0.69608	5.61824	-0.55548
H	-0.89538	4.94494	1.30384
H	-2.28996	3.27291	-0.84217
O	-0.06981	0.22977	0.93400
O	2.19044	0.60385	0.80764
O	-2.36764	0.27435	0.96822
Si	-3.14452	-0.62780	-0.25173
Si	2.97330	-0.23575	-0.45338
C	-4.96587	-0.25064	-0.04052
H	-5.14072	0.82844	-0.11757
H	-5.56872	-0.74644	-0.81019
H	-5.33150	-0.57842	0.93890
C	-2.49924	-0.03235	-1.90931
H	-2.91352	-0.63323	-2.72802
H	-2.77867	1.01268	-2.08536
H	-1.40674	-0.09701	-1.96345
C	4.39716	0.84574	-1.01588
H	4.99848	0.35080	-1.78765
H	4.02032	1.78479	-1.43752
H	5.05875	1.09788	-0.17985
C	1.73731	-0.49303	-1.83984
H	2.20794	-0.99105	-2.69592
H	0.89272	-1.10750	-1.51051
H	1.33791	0.46722	-2.18712
C	3.56936	-1.86606	0.29971
C	4.46466	-1.57690	1.51740
H	3.92903	-1.00806	2.28570
H	4.79976	-2.52049	1.97158
H	5.36288	-1.01107	1.24065
C	4.37106	-2.65979	-0.74684
H	4.70767	-3.61500	-0.31945
H	3.76975	-2.89530	-1.63418
H	5.26608	-2.11827	-1.07752
C	2.35597	-2.69775	0.74975
H	2.69274	-3.62493	1.23542
H	1.73222	-2.15225	1.46715
H	1.72150	-2.98512	-0.09729
C	-2.77084	-2.46481	0.03289
C	-3.03473	-2.82397	1.50547
H	-2.82804	-3.89027	1.67640
H	-2.39546	-2.24548	2.18114
H	-4.07849	-2.64379	1.79162
C	-1.29873	-2.76104	-0.29963
H	-1.06707	-3.81547	-0.08998
H	-1.07799	-2.58292	-1.35998
H	-0.61724	-2.14653	0.29822
C	-3.67816	-3.31853	-0.87111
H	-3.53712	-3.08795	-1.93512
H	-3.44735	-4.38497	-0.73715
H	-4.74017	-3.18236	-0.63323
H	-2.73211	3.38969	0.85828
H	-1.72045	5.51718	-0.14641
H	0.02597	4.32536	-1.55120
H	2.06826	3.46239	-0.50990

TS-1 (ketenimine *exo*-lone pair)

C	1.34530	0.06066	1.89068
C	-0.01900	-0.11710	2.12005
C	-0.76631	0.56671	3.21866
C	0.23073	1.09450	4.26188
C	1.38141	1.86378	3.59793

C	2.23461	0.96117	2.69113
C	1.63562	-0.69821	0.76567
C	-0.51697	-0.88552	1.05488
H	-1.34173	1.40133	2.79651
H	-0.29384	1.74131	4.97352
H	0.96607	2.68123	2.99633
H	2.92083	0.35616	3.30026
O	0.57739	-1.41170	0.36284
O	-1.62469	-1.63459	1.02603
O	2.78338	-0.86179	0.15794
Si	3.08723	-1.64592	-1.34658
Si	-1.80461	-3.14065	1.79993
C	2.62842	-3.45242	-1.15274
H	1.54005	-3.57196	-1.16410
H	3.03983	-4.04978	-1.97551
H	3.00916	-3.86691	-0.21265
C	2.09838	-0.84320	-2.70958
H	2.21419	-1.41695	-3.63797
H	1.03652	-0.80929	-2.44855
H	2.41207	0.18769	-2.90197
C	-0.67349	-3.19882	3.29588
H	-0.73076	-4.18417	3.77446
H	0.36998	-3.03273	3.00358
H	-0.93201	-2.44492	4.04710
C	-1.32754	-4.47614	0.57715
H	-1.54541	-5.47747	0.96761
H	-1.86328	-4.35504	-0.37049
H	-0.25364	-4.42678	0.36383
C	-3.63958	-3.18253	2.25523
C	-3.97504	-1.97767	3.15142
H	-3.76302	-1.02794	2.64692
H	-5.04283	-1.98581	3.41246

H	-3.41023	-1.99585	4.09213
C	-3.95840	-4.48498	3.00996
H	-5.02568	-4.52006	3.27028
H	-3.74257	-5.37449	2.40492
H	-3.39189	-4.56645	3.94618
C	-4.49003	-3.11025	0.97435
H	-5.55900	-3.09774	1.23088
H	-4.27490	-2.20500	0.39480
H	-4.32054	-3.97560	0.32228
C	4.95249	-1.38888	-1.53421
C	5.70441	-2.13070	-0.41583
H	6.78723	-1.96867	-0.51568
H	5.40541	-1.77513	0.57704
H	5.53091	-3.21305	-0.45534
C	5.28832	0.11120	-1.46093
H	6.37064	0.26083	-1.58315
H	4.78941	0.68222	-2.25388
H	4.99785	0.54353	-0.49675
C	5.39665	-1.94038	-2.90138
H	4.90774	-1.41779	-3.73265
H	6.48097	-1.81127	-3.02587
H	5.18433	-3.01219	-3.00360
C	-0.79921	0.71037	-0.25500
C	-2.12601	0.80422	-0.38458
H	-2.76121	0.17471	0.22921
C	-2.79269	1.68010	-1.35893
C	-2.09475	2.48330	-2.27504
C	-4.19575	1.72659	-1.37638
C	-2.77959	3.30013	-3.16923
H	-1.00937	2.46227	-2.28976
C	-4.87861	2.54532	-2.26994
H	-4.75461	1.10866	-0.67639

C	-4.17332	3.33954	-3.17285
H	-2.21712	3.91335	-3.86848
H	-5.96541	2.56252	-2.26141
H	-4.70333	3.98117	-3.87117
N	0.34758	1.09403	-0.64343
C	0.90857	2.32051	-0.25115
C	2.25705	2.55217	-0.52641
C	0.17600	3.34082	0.37711
C	2.87927	3.74652	-0.16596
H	2.82987	1.76909	-1.01309
C	0.78344	4.53506	0.73182
H	-0.88114	3.19049	0.58060
C	2.14135	4.74535	0.47221
H	3.93226	3.87856	-0.39095
H	0.21664	5.32648	1.21328
H	2.85452	1.57213	2.02524
H	2.01885	2.32149	4.36231
H	0.63765	0.25004	4.83625
H	-1.49715	-0.11256	3.67496
C	4.00655	6.21224	0.61462
H	4.19712	7.21342	1.00361
H	4.65793	5.49332	1.12968
H	4.22404	6.19405	-0.46160
O	2.64615	5.94898	0.86932

TS-SI-1 (ketenimine *endo*-lone pair)

C	-0.75847	0.27623	2.39488
C	0.55891	0.71536	2.27186
C	1.55145	0.69249	3.38634
C	0.83325	0.43748	4.72088
C	-0.19458	-0.69579	4.59666
C	-1.33731	-0.32041	3.63995
C	-1.31305	0.39295	1.13100
C	0.78702	0.94643	0.90375
H	2.27741	-0.10886	3.18953
H	1.57250	0.19626	5.49214
H	0.30777	-1.59480	4.21606
H	-2.01424	0.39495	4.12763
O	-0.45207	0.94266	0.26151
O	1.71538	1.73400	0.34497
O	-2.52573	0.11570	0.72279
Si	-3.35384	0.86947	-0.58845
Si	1.58352	3.42669	0.22203
C	-3.22124	2.71888	-0.29734
H	-2.18715	3.05932	-0.41786
H	-3.83645	3.27260	-1.01661
H	-3.55187	2.99343	0.71066
C	-2.58099	0.37872	-2.21123
H	-3.08269	0.88586	-3.04486
H	-1.52124	0.65178	-2.23402
H	-2.64194	-0.70125	-2.37904
C	0.46575	4.03714	1.60015
H	0.36002	5.12783	1.55393
H	-0.53726	3.60272	1.51584
H	0.85546	3.77730	2.59062
C	0.84604	3.81987	-1.45436
H	0.71533	4.89920	-1.59696
H	1.48030	3.44324	-2.26413
H	-0.13571	3.34412	-1.55879
C	3.36660	4.03365	0.39387
C	3.93726	3.57739	1.74783
H	3.94083	2.48470	1.83471
H	4.97479	3.92360	1.85742
H	3.36732	3.98422	2.59271

C	3.39940	5.57004	0.31441
H	4.43377	5.93166	0.40010
H	3.00364	5.94054	-0.63960
H	2.82370	6.03602	1.12402
C	4.22753	3.44371	-0.73746
H	5.27388	3.75929	-0.61862
H	4.20727	2.34773	-0.73376
H	3.89462	3.78383	-1.72534
C	-5.12506	0.24094	-0.37993
C	-5.68682	0.67935	0.98335
H	-6.71090	0.29984	1.10867
H	-5.08425	0.29115	1.81246
H	-5.73015	1.77130	1.07692
C	-5.15703	-1.29380	-0.47209
H	-6.18947	-1.65527	-0.35954
H	-4.78149	-1.65137	-1.43772
H	-4.54930	-1.75860	0.31195
C	-5.99552	0.83201	-1.50458
H	-5.64720	0.52407	-2.49813
H	-7.03241	0.48315	-1.39960
H	-6.01839	1.92875	-1.47752
C	1.29316	-1.00610	0.46622
C	2.61959	-0.99120	0.29056
H	3.14598	-0.04383	0.33443
C	3.41991	-2.18818	-0.00023
C	2.87751	-3.48221	-0.06262
C	4.79892	-2.04122	-0.22290
C	3.68821	-4.58034	-0.33349
H	1.81550	-3.62900	0.10708
C	5.60679	-3.13993	-0.49588
H	5.23888	-1.04687	-0.17878
C	5.05533	-4.41922	-0.55369
H	3.24426	-5.57154	-0.37674
H	6.67108	-2.99657	-0.66429
N	0.24323	-1.71794	0.55066
C	-0.44731	-2.20061	-0.56725
C	0.06584	-2.15178	-1.87630
C	-1.68441	-2.81539	-0.36716
C	-0.63595	-2.70502	-2.93375
H	1.03259	-1.68701	-2.05144
C	-2.39641	-3.37773	-1.42648
H	-2.08518	-2.85595	0.64133
C	-1.87358	-3.32221	-2.71846
H	-0.23759	-2.67445	-3.94342
H	-3.35176	-3.84957	-1.22437
H	-1.93561	-1.20382	3.39057
H	-0.60355	-0.94491	5.58196
H	0.32200	1.35517	5.04439
H	2.12407	1.62832	3.41792
C	-3.72899	-4.47637	-3.65359
H	-4.03664	-4.80748	-4.64645
H	-3.63978	-5.34789	-2.99164
H	-4.48655	-3.79050	-3.25001
O	-2.48850	-3.83138	-3.82577
H	5.68291	-5.27938	-0.76942

int-1

C	-0.64660	0.51539	2.53690
C	0.52486	1.14881	2.53523
C	1.35266	1.43599	3.74075
C	0.49544	1.21271	4.99898
C	-0.33193	-0.07933	4.91010
C	-1.32800	-0.04861	3.73753
C	-0.97340	0.27267	1.07253

C	0.90226	1.26807	1.06454
H	2.22940	0.77180	3.74707
H	1.13817	1.18545	5.88593
H	0.35075	-0.92832	4.76972
H	-2.21131	0.55422	3.99101
O	-0.39497	1.41878	0.44709
O	1.79004	2.24083	0.74360
O	-2.26143	0.04650	0.75860
Si	-3.24299	0.88685	-0.34884
Si	1.95444	3.04417	-0.74227
C	-3.31396	2.69296	0.14969
H	-2.32728	3.15632	0.05722
H	-4.01654	3.24976	-0.48240
H	-3.63448	2.80409	1.19169
C	-2.57241	0.68898	-2.08426
H	-3.20045	1.22172	-2.80900
H	-1.56012	1.10251	-2.14712
H	-2.51756	-0.36402	-2.38175
C	0.59893	4.33074	-0.88327
H	0.78654	5.02057	-1.71518
H	-0.36423	3.84091	-1.06290
H	0.50997	4.92019	0.03570
C	1.83647	1.85223	-2.18428
H	1.95170	2.39242	-3.13218
H	2.59507	1.06350	-2.14294
H	0.85180	1.37284	-2.19337
C	3.67515	3.82520	-0.60284
C	3.69123	4.85421	0.54086
H	3.42596	4.39489	1.50010
H	4.69515	5.29077	0.64503
H	2.99282	5.67964	0.35610
C	4.02850	4.52905	-1.92501
H	5.01459	5.00857	-1.84651
H	4.07446	3.82499	-2.76495
H	3.30567	5.31406	-2.18094
C	4.72147	2.73489	-0.31439
H	5.72665	3.17755	-0.26198
H	4.52721	2.23610	0.64167
H	4.74304	1.96831	-1.09960
C	-4.93419	0.05527	-0.13589
C	-5.45144	0.27458	1.29636
H	-6.41892	-0.23048	1.43156
H	-4.75670	-0.13019	2.04168
H	-5.60446	1.33766	1.51797
C	-4.83604	-1.45482	-0.40611
H	-5.83170	-1.91643	-0.33008
H	-4.44351	-1.67181	-1.40678
H	-4.18061	-1.94773	0.31900
C	-5.92482	0.68096	-1.13493
H	-5.61904	0.51197	-2.17478
H	-6.92052	0.23189	-1.01033
H	-6.03585	1.76237	-0.98596
C	1.22300	-0.17329	0.61430
C	2.46194	-0.60943	0.34938
H	3.23528	0.15558	0.38053
C	2.90222	-1.96006	-0.02145
C	2.22487	-3.11760	0.38838
C	4.05698	-2.10910	-0.80289
C	2.67508	-4.37685	0.00634
H	1.34321	-3.02666	1.01465
C	4.50658	-3.36835	-1.18787
H	4.60320	-1.22129	-1.11581
C	3.81276	-4.50935	-0.78839
H	2.13221	-5.26053	0.33072

H	5.39937	-3.45896	-1.80075
N	-0.02319	-0.82571	0.61775
C	-0.43257	-1.66234	-0.45769
C	0.12523	-1.58047	-1.73782
C	-1.42613	-2.60690	-0.22494
C	-0.31594	-2.40921	-2.75579
H	0.92088	-0.87091	-1.93451
C	-1.89712	-3.42927	-1.24705
H	-1.84822	-2.69077	0.77160
C	-1.34052	-3.33149	-2.52267
H	0.12136	-2.35237	-3.74760
H	-2.68810	-4.13774	-1.02745
H	-1.69926	-1.05788	3.51747
H	-0.86502	-0.24908	5.85233
H	-0.18480	2.06623	5.12350
H	1.74160	2.46076	3.70957
C	-2.74631	-5.03376	-3.40259
H	-2.89126	-5.51520	-4.37049
H	-2.46236	-5.79059	-2.65969
H	-3.68323	-4.55182	-3.09289
O	-1.71851	-4.08697	-3.59053
H	4.15884	-5.49410	-1.08963

Heterocycle 3c

C	0.73086	0.17514	2.96694
C	-0.55240	-0.20764	3.10770
C	-1.00140	-1.24571	4.10154
C	0.16691	-2.07041	4.64078
C	1.33281	-1.15908	5.02179
C	1.84693	-0.39499	3.80148
C	1.13458	1.24980	2.01351
C	-1.59790	0.41535	2.27109
H	-1.52238	-0.73455	4.92281
H	-0.16376	-2.66124	5.50178
H	0.99746	-0.44387	5.78526
H	2.44800	-1.05613	3.16048
O	-2.78020	0.18163	2.47645
O	2.27701	1.68266	2.02442
C	-1.15299	1.26819	1.12027
C	-2.07656	1.47390	0.15637
H	-3.05936	1.09529	0.42886
C	-1.99349	2.11875	-1.16168
C	-0.94907	1.86977	-2.05879
C	-3.02958	2.97599	-1.55680
C	-0.91738	2.49777	-3.29929
H	-0.16176	1.17586	-1.78220
C	-2.99251	3.61343	-2.79209
H	-3.85882	3.15729	-0.87717
C	-1.93130	3.38036	-3.66542
H	-0.09517	2.29826	-3.98070
H	-3.79331	4.29082	-3.07462
H	-1.90050	3.87675	-4.63118
N	0.17564	1.75335	1.14373
C	0.51752	2.93711	0.40187
C	1.59122	2.94235	-0.47583
C	-0.24910	4.09162	0.55909
C	1.89355	4.08068	-1.21951
H	2.20104	2.05271	-0.58700
C	0.03068	5.22286	-0.18783
H	-1.08708	4.09359	1.24992
C	1.10131	5.22232	-1.08811
H	2.73914	4.05491	-1.89713
H	-0.57046	6.12080	-0.08991
H	2.51998	0.41708	4.09112

H	2.15133	-1.73648	5.46488
H	0.49892	-2.78270	3.87294
H	-1.75772	-1.88265	3.63093
C	2.34326	6.42420	-2.71985
H	2.30412	7.42108	-3.16017
H	3.31800	6.27862	-2.23703
H	2.20775	5.67241	-3.50821
O	1.29170	6.37516	-1.77996

TS-2 (formaldimine 5 *exo*-lone pair)

C	0.40452	0.85572	1.12801
C	-0.87426	1.15976	0.68987
C	-1.62182	2.38264	1.10675
C	-0.96681	2.99219	2.35688
C	0.56048	3.07359	2.21873
C	1.19699	1.68154	2.09132
C	0.77361	-0.30216	0.44310
C	-1.17549	0.25924	-0.36317
H	-1.60129	3.11149	0.28369
H	-1.38300	3.98935	2.53654
H	0.81401	3.66338	1.32951
H	1.22107	1.18333	3.07103
O	-0.24306	-0.79655	-0.26772
O	-2.40600	-0.11651	-0.76630
O	1.87741	-0.99106	0.56030
Si	2.51611	-2.21311	-0.47983
Si	-3.38870	-1.20643	0.09873
C	1.21307	-3.54194	-0.70025
H	0.42815	-3.20302	-1.38385
H	1.65911	-4.45209	-1.12004
H	0.74260	-3.80657	0.25330
C	3.00025	-1.43458	-2.10262
H	3.97428	-0.93587	-2.04203
H	3.05576	-2.19146	-2.89448
H	2.25898	-0.67189	-2.37763
C	-2.98396	-1.08676	1.92746
H	-3.60402	-1.78896	2.49825
H	-1.93474	-1.34316	2.11309
H	-3.15635	-0.08240	2.32928
C	-3.02494	-2.93343	-0.52997
H	-3.67525	-3.67998	-0.05844
H	-3.16147	-3.00012	-1.61487
H	-1.98616	-3.20270	-0.30731
C	-5.15292	-0.64491	-0.29337
C	-5.34277	0.81271	0.16087
H	-4.64499	1.48665	-0.34919
H	-6.36306	1.15162	-0.06891
H	-5.19782	0.92727	1.24256
C	-6.15766	-1.54514	0.44713
H	-7.18612	-1.23131	0.21905
H	-6.06530	-2.59688	0.14851
H	-6.03465	-1.49008	1.53604
C	-5.40554	-0.73766	-1.80850
H	-6.42010	-0.38577	-2.04465
H	-4.69821	-0.12136	-2.37497
H	-5.32457	-1.76876	-2.17351
C	3.99572	-2.82287	0.53023
C	3.51714	-3.50511	1.82283
H	4.37946	-3.84570	2.41380
H	2.93544	-2.81936	2.45002
H	2.89559	-4.38463	1.61469
C	4.90971	-1.63854	0.89118
H	5.77887	-1.99456	1.46273
H	5.29317	-1.12854	-0.00099

H	4.38636	-0.89690	1.50466
C	4.78790	-3.83359	-0.31918
H	5.16336	-3.38388	-1.24671
H	5.65812	-4.19993	0.24352
H	4.18424	-4.70961	-0.58857
C	-0.37964	1.08989	-1.91117
N	0.93988	1.16002	-1.75253
C	1.52420	2.37228	-1.37156
C	2.81976	2.33100	-0.83598
C	0.94725	3.64830	-1.52929
C	3.49515	3.47857	-0.42889
H	3.29174	1.35877	-0.71854
C	1.61383	4.80078	-1.13628
H	-0.03143	3.75592	-1.98743
C	2.88756	4.72822	-0.56971
H	4.49041	3.38005	-0.00793
H	1.15817	5.77798	-1.26901
H	2.23457	1.76805	1.75214
H	0.98649	3.59532	3.08282
H	-1.22032	2.37794	3.23265
H	-2.67961	2.15212	1.28722
H	-1.01321	1.98525	-1.95009
H	-0.73906	0.29344	-2.56590
C	4.74634	5.88561	0.35471
H	5.47835	5.46899	-0.35028
H	5.00649	6.92264	0.57223
H	4.76872	5.30383	1.28642
O	3.45388	5.91702	-0.20260

TS-SI-2 (formaldimine 5 *endo*-lone pair)

C	-0.23391	2.47717	-0.10682
C	1.12382	2.47508	0.15768
C	1.91542	3.70681	0.43394
C	1.11573	4.95106	0.01641
C	-0.33994	4.86988	0.49698
C	-1.08993	3.70321	-0.16395
C	-0.60572	1.13673	-0.19247
C	1.48617	1.12887	0.43413
H	2.13388	3.75126	1.51302
H	1.59867	5.84833	0.41822
H	-0.35134	4.72537	1.58524
H	-1.33021	3.95204	-1.20717
O	0.45071	0.32417	-0.08203
O	2.72137	0.59950	0.31546
O	-1.77135	0.62539	-0.48288
Si	-2.05414	-0.86646	-1.30409
Si	3.42327	0.16424	-1.17290
C	-0.97605	-0.82621	-2.84073
H	0.08267	-0.93134	-2.58170
H	-1.23345	-1.65037	-3.51688
H	-1.09774	0.11207	-3.39375
C	-1.62235	-2.30462	-0.20394
H	-1.75486	-3.25283	-0.73975
H	-0.58009	-2.23941	0.12436
H	-2.24382	-2.32615	0.69686
C	2.69409	1.23561	-2.53079
H	3.13932	0.97803	-3.49963
H	1.61137	1.08776	-2.61247
H	2.86881	2.30225	-2.35174
C	3.03480	-1.64347	-1.48017
H	3.40570	-1.97717	-2.45659
H	3.48016	-2.28328	-0.71049
H	1.95162	-1.80755	-1.45547
C	5.27431	0.47115	-0.92540

C	5.51264	1.95610	-0.60157
H	4.99377	2.25419	0.31674
H	6.58566	2.14457	-0.45432
H	5.17508	2.61314	-1.41297
C	6.03274	0.09961	-2.21196
H	7.11093	0.26529	-2.07683
H	5.89697	-0.95613	-2.47836
H	5.71490	0.70857	-3.06760
C	5.79709	-0.38639	0.24059
H	6.86516	-0.18563	0.40714
H	5.26652	-0.16668	1.17395
H	5.69538	-1.45923	0.03739
C	-3.89266	-0.76588	-1.73415
C	-4.15540	0.43436	-2.65977
H	-5.22700	0.50053	-2.89599
H	-3.85983	1.37935	-2.18954
H	-3.61657	0.34463	-3.61092
C	-4.73033	-0.61252	-0.45412
H	-5.79931	-0.56653	-0.70820
H	-4.58504	-1.45646	0.22983
H	-4.47178	0.30172	0.09092
C	-4.30334	-2.06465	-2.45285
H	-4.15904	-2.94751	-1.81811
H	-5.36872	-2.02499	-2.71984
H	-3.74198	-2.21952	-3.38294
C	1.04863	0.96894	2.28769
N	-0.26097	1.14518	2.46926
C	-1.08936	0.04706	2.69138
C	-0.67242	-1.21529	3.16760
C	-2.47156	0.22778	2.52514
C	-1.58321	-2.22235	3.45078
H	0.37863	-1.40687	3.36181
C	-3.39496	-0.77445	2.81363
H	-2.81818	1.19549	2.17395
C	-2.95306	-2.01445	3.27489
H	-1.24682	-3.18371	3.82857
H	-4.45095	-0.56938	2.67328
H	-2.04141	3.51900	0.34630
H	-0.85934	5.81158	0.28814
H	1.13134	5.04345	-1.07862
H	2.88840	3.67260	-0.07262
H	1.50938	-0.02311	2.38190
H	1.70115	1.76629	2.65485
C	-5.15927	-2.87739	3.46640
H	-5.62100	-3.81920	3.76720
H	-5.50959	-2.07374	4.12812
H	-5.45229	-2.64628	2.43262
O	-3.76985	-3.07018	3.58031

int-2

C	0.15490	2.06094	-0.52289
C	1.45940	1.95715	-0.27506
C	2.46133	3.04201	-0.48589
C	1.83570	4.13547	-1.36825
C	0.40792	4.48534	-0.92036
C	-0.54154	3.27978	-1.02755
C	-0.46580	0.79685	0.05200
C	1.61034	0.63649	0.46994
H	2.76613	3.45955	0.48582
H	2.46528	5.03216	-1.35299
H	0.43674	4.82004	0.12555
H	-0.85863	3.12887	-2.06918
O	0.58643	-0.16220	-0.15409
O	2.83292	0.03254	0.49260

O	-1.66876	0.40483	-0.41260
Si	-2.02659	-0.53825	-1.76727
Si	3.46734	-0.91417	-0.76366
C	-1.46030	0.37347	-3.30807
H	-0.40235	0.64933	-3.21748
H	-1.56609	-0.25392	-4.20131
H	-2.03347	1.29319	-3.47071
C	-1.18399	-2.20838	-1.65365
H	-1.64880	-2.92976	-2.33721
H	-0.12393	-2.12671	-1.91362
H	-1.25232	-2.60878	-0.63627
C	2.94799	-0.22844	-2.43316
H	3.39437	-0.81557	-3.24520
H	1.85912	-0.27760	-2.53625
H	3.24992	0.81603	-2.56831
C	2.83497	-2.66715	-0.56072
H	3.17984	-3.31890	-1.37250
H	3.16382	-3.10440	0.38865
H	1.73946	-2.66757	-0.56651
C	5.34142	-0.79823	-0.50426
C	5.79430	0.66579	-0.63799
H	5.30686	1.30562	0.10666
H	6.88057	0.74342	-0.48540
H	5.57531	1.07342	-1.63300
C	6.05978	-1.65249	-1.56358
H	7.14843	-1.59874	-1.41981
H	5.77491	-2.71018	-1.49967
H	5.84897	-1.30651	-2.58330
C	5.71268	-1.31099	0.89801
H	6.79692	-1.22176	1.05898
H	5.20914	-0.73689	1.68388
H	5.44970	-2.36756	1.02975
C	-3.90859	-0.68972	-1.64599
C	-4.54177	0.70849	-1.54421
H	-5.63559	0.62270	-1.46824
H	-4.18386	1.24510	-0.65890
H	-4.32343	1.32423	-2.42608
C	-4.26581	-1.49584	-0.38401
H	-5.35838	-1.57002	-0.27954
H	-3.87087	-2.51832	-0.42507
H	-3.87295	-1.02207	0.52287
C	-4.45594	-1.40738	-2.89151
H	-4.04202	-2.41776	-3.00123
H	-5.54817	-1.51059	-2.82020
H	-4.23989	-0.85184	-3.81307
C	0.96386	0.83273	1.87216
N	-0.47072	0.96693	1.53636
C	-1.35962	0.05415	2.20052
C	-1.15288	-1.33253	2.22945
C	-2.50912	0.56483	2.78666
C	-2.07462	-2.17417	2.82927
H	-0.27106	-1.75264	1.75375
C	-3.44995	-0.27049	3.39462
H	-2.67331	1.63775	2.75372
C	-3.23300	-1.64774	3.41341
H	-1.92482	-3.24946	2.84748
H	-4.33737	0.16767	3.83767
H	-1.45784	3.45083	-0.44908
H	0.02308	5.32262	-1.51339
H	1.80893	3.78094	-2.40799
H	3.37689	2.64917	-0.94558
H	1.16064	-0.05090	2.48575
H	1.34484	1.72123	2.38477
C	-5.28060	-2.08375	4.53923

H	-5.80761	-2.96662	4.90320
H	-5.08334	-1.40662	5.38054
H	-5.90256	-1.57012	3.79445
O	-4.08132	-2.55664	3.96743

Pyridone 7

C	-0.37468	2.79372	-0.11791
C	-1.42309	1.93562	-0.30567
C	-2.84535	2.42290	-0.44551
C	-3.01840	3.87828	-0.01197
C	-1.90333	4.74530	-0.59359
C	-0.54307	4.29087	-0.06634
C	0.99263	2.31308	0.04818
C	-1.17034	0.52471	-0.37684
H	-3.14974	2.29966	-1.49449
H	-4.00379	4.24085	-0.32571
H	-1.91946	4.66612	-1.68964
H	-0.40594	4.62662	0.97161
C	0.09835	0.06667	-0.25080
N	1.15602	0.92473	-0.04340
C	2.47312	0.36468	0.04285
C	2.96658	-0.39184	-1.00965
C	3.25118	0.55321	1.18712
C	4.22772	-0.98596	-0.92985
H	2.36945	-0.51719	-1.90882
C	4.50559	-0.02405	1.27179
H	2.87340	1.16503	1.99853
C	5.00116	-0.80014	0.21586
H	4.58727	-1.57263	-1.76736
H	5.12628	0.11539	2.15089
H	0.27491	4.75424	-0.62802
H	-2.06340	5.80104	-0.34808
H	-2.98839	3.94050	1.08475
H	-3.50892	1.76645	0.12783
H	0.34215	-0.98795	-0.28695
C	6.80372	-2.09360	-0.63917
H	6.91608	-1.50132	-1.55625
H	7.78810	-2.40031	-0.28429
H	6.19929	-2.98548	-0.84926
O	6.23987	-1.32475	0.40153
O	-2.21533	-0.32542	-0.59298
O	1.95643	3.05173	0.24946
Si	-2.86757	-1.44732	0.49361
C	-4.66446	-0.96973	0.74242
C	-1.93189	-1.36083	2.11768
C	-2.71870	-3.15137	-0.32465
H	-5.22747	-1.76690	1.24255
H	-4.74928	-0.06788	1.35987
H	-5.15052	-0.76564	-0.21812
H	-2.45409	-1.94908	2.88192
H	-0.90885	-1.74439	2.04060
H	-1.86983	-0.32717	2.47779
C	-3.62510	-3.21179	-1.56657
C	-3.14743	-4.24019	0.67506
C	-1.26390	-3.40380	-0.75440
H	-3.36935	-2.43080	-2.29251
H	-3.51567	-4.18313	-2.07009
H	-4.68385	-3.09698	-1.30455
H	-3.09661	-5.23082	0.20133
H	-2.49563	-4.26604	1.55702
H	-4.17918	-4.10012	1.02168
H	-1.16785	-4.40379	-1.20085
H	-0.93594	-2.67488	-1.50458
H	-0.56885	-3.36092	0.09435

(TBS)$_2$O

O	-0.00005	0.00057	-0.38569
Si	-1.39579	-0.77003	0.06844
Si	1.39608	0.77054	0.06832
C	-1.59159	-2.27214	-1.04007
H	-0.78127	-2.98904	-0.86117
H	-2.54037	-2.79132	-0.85841
H	-1.55683	-1.98930	-2.09807
C	-1.26822	-1.31736	1.86454
H	-2.13141	-1.92930	2.15306
H	-0.36708	-1.92304	2.01925
H	-1.21800	-0.46408	2.55056
C	1.26873	1.31818	1.86434
H	2.13156	1.93069	2.15266
H	0.36722	1.92327	2.01919
H	1.21915	0.46490	2.55042
C	1.59265	2.27226	-1.04057
H	2.54085	2.79209	-0.85778
H	1.55958	1.98885	-2.09847
H	0.78162	2.98873	-0.86323
C	2.82920	-0.45569	-0.16223
C	2.54065	-1.73924	0.63456
H	1.62569	-2.23220	0.28464
H	3.36648	-2.45598	0.51738
H	2.42967	-1.54221	1.70833
C	4.14500	0.16512	0.33653
H	4.97980	-0.53399	0.18286
H	4.39263	1.08933	-0.20082
H	4.10745	0.39785	1.40799
C	2.96595	-0.81481	-1.65168
H	3.75905	-1.56362	-1.79332
H	2.03550	-1.23515	-2.05203
H	3.22987	0.05949	-2.25919
C	-2.82960	0.45531	-0.16230
C	-2.96718	0.81323	-1.65198
H	-3.76071	1.56155	-1.79384
H	-2.03712	1.23375	-2.05307
H	-3.23092	-0.06168	-2.25868
C	-2.54125	1.73955	0.63344
H	-3.36750	2.45581	0.51624
H	-2.42958	1.54329	1.70729
H	-1.62672	2.23271	0.28268
C	-4.14488	-0.16574	0.33752
H	-4.10683	-0.39737	1.40918
H	-4.98009	0.53278	0.18343
H	-4.39223	-1.09060	-0.19883

HOTBS

O	1.12366	0.00005	1.71978
Si	0.80362	0.00006	0.06734
C	1.55284	1.53033	-0.73148
H	2.64555	1.53172	-0.63230
H	1.32452	1.57180	-1.80346
H	1.17594	2.44966	-0.26959
C	1.55305	-1.53009	-0.73166
H	1.32638	-1.57030	-1.80404
H	2.64562	-1.53224	-0.63082
H	1.17492	-2.44968	-0.27133
C	-1.08720	-0.00004	-0.04880
C	-1.65041	1.25327	0.64416
H	-2.74928	1.24736	0.60065
H	-1.35630	1.29649	1.69866
H	-1.31025	2.17648	0.15882
C	-1.65025	-1.25291	0.64499

H	-2.74913	-1.24706	0.60175
H	-1.31020	-2.17635	0.16005
H	-1.35588	-1.29560	1.69945
C	-1.51977	-0.00057	-1.52549
H	-1.15906	-0.88834	-2.06002
H	-2.61682	-0.00073	-1.59755
H	-1.15928	0.88688	-2.06068
H	2.04385	-0.00120	1.99909

NMR Spectra

Synthesis of Bis(silyloxy)furans

Cyclic Anhydrides and their Precursors

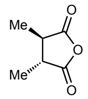

CA-01
^{1}H NMR
$CDCl_3$, 600 MHz

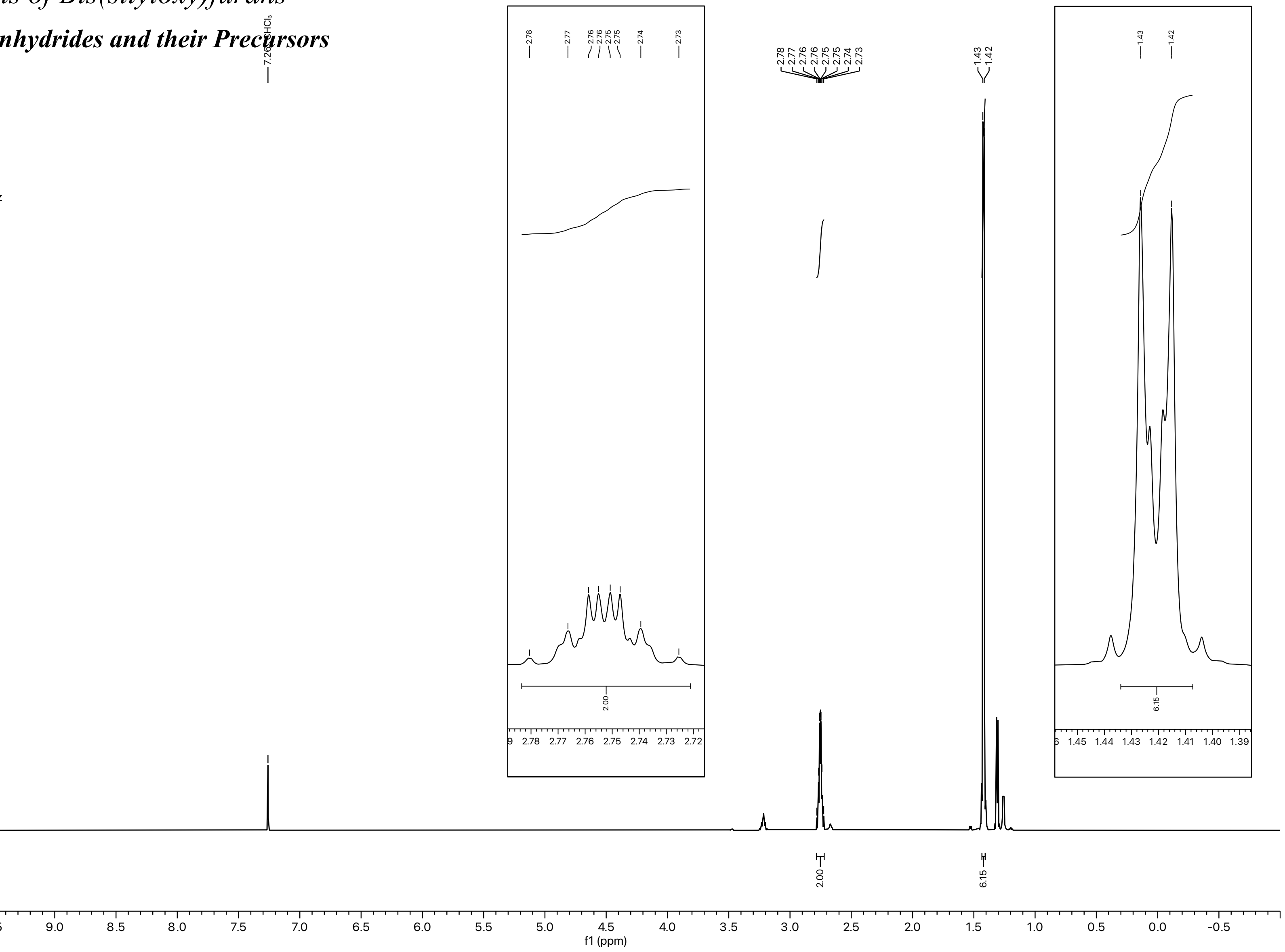

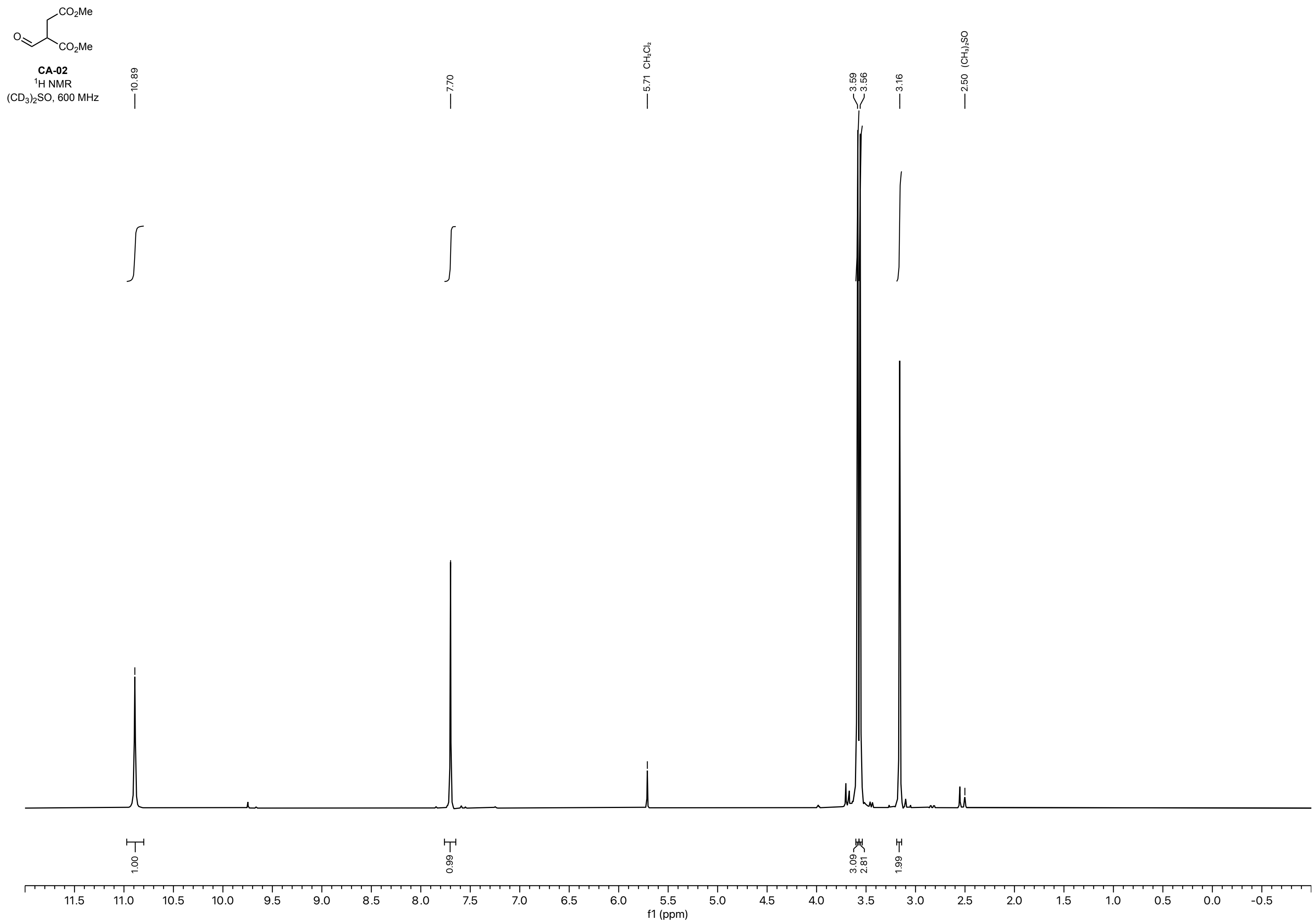

CO2Me
O
CO2Me
CA-02
1H NMR
(CD3)2SO, 600 MHz
10.89
7.70
5.71 CH2Cl2
3.59
3.56
3.16
2.50 (CH3)2SO
1.00
0.99
3.09
2.81
1.99
11.5 11.0 10.5 10.0 9.5 9.0 8.5 8.0 7.5 7.0 6.5 6.0 5.5 5.0 4.5 4.0 3.5 3.0 2.5 2.0 1.5 1.0 0.5 0.0 -0.5
f1 (ppm)

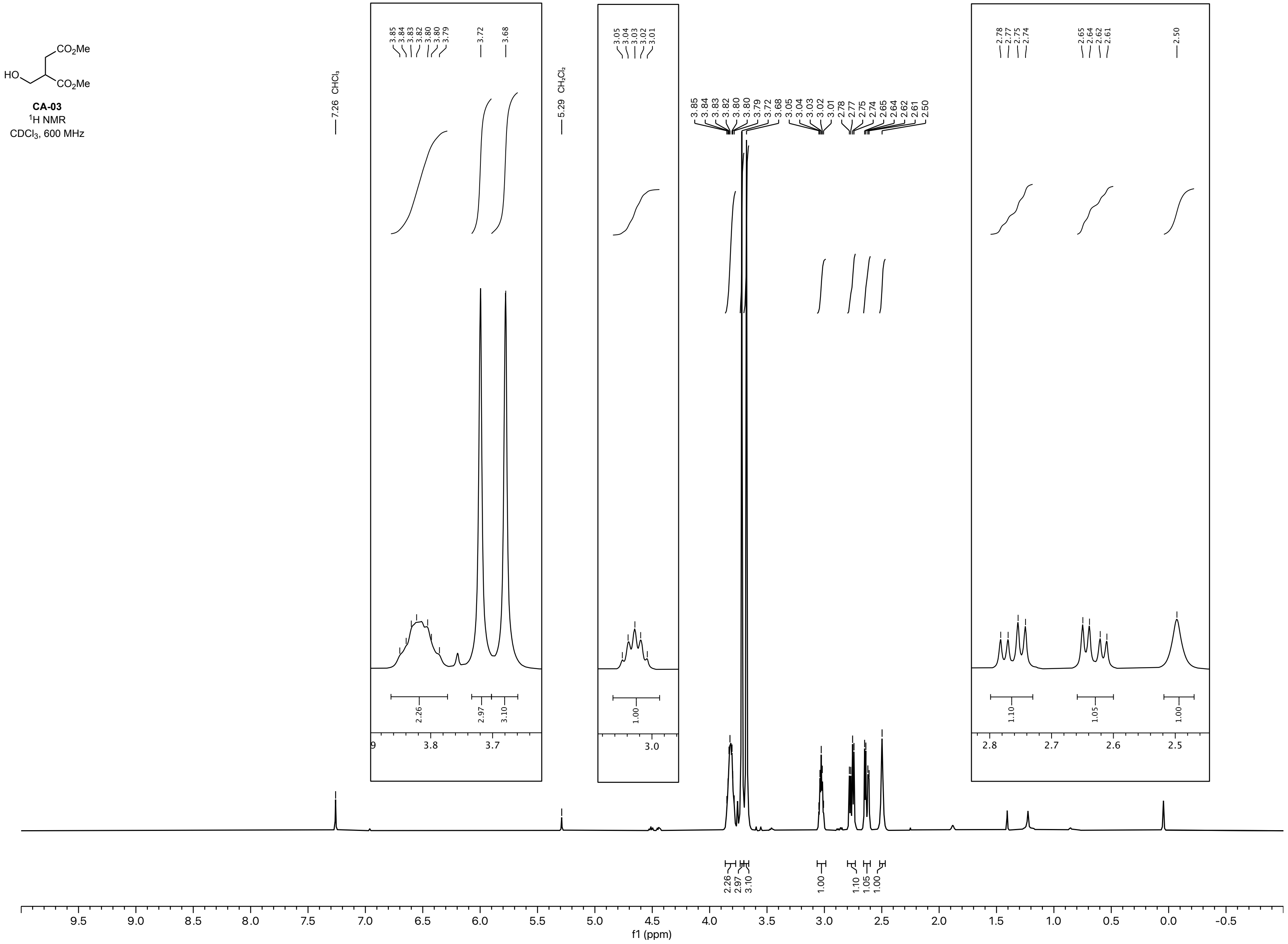

CA-03
^{1}H NMR
$CDCl_3$, 600 MHz

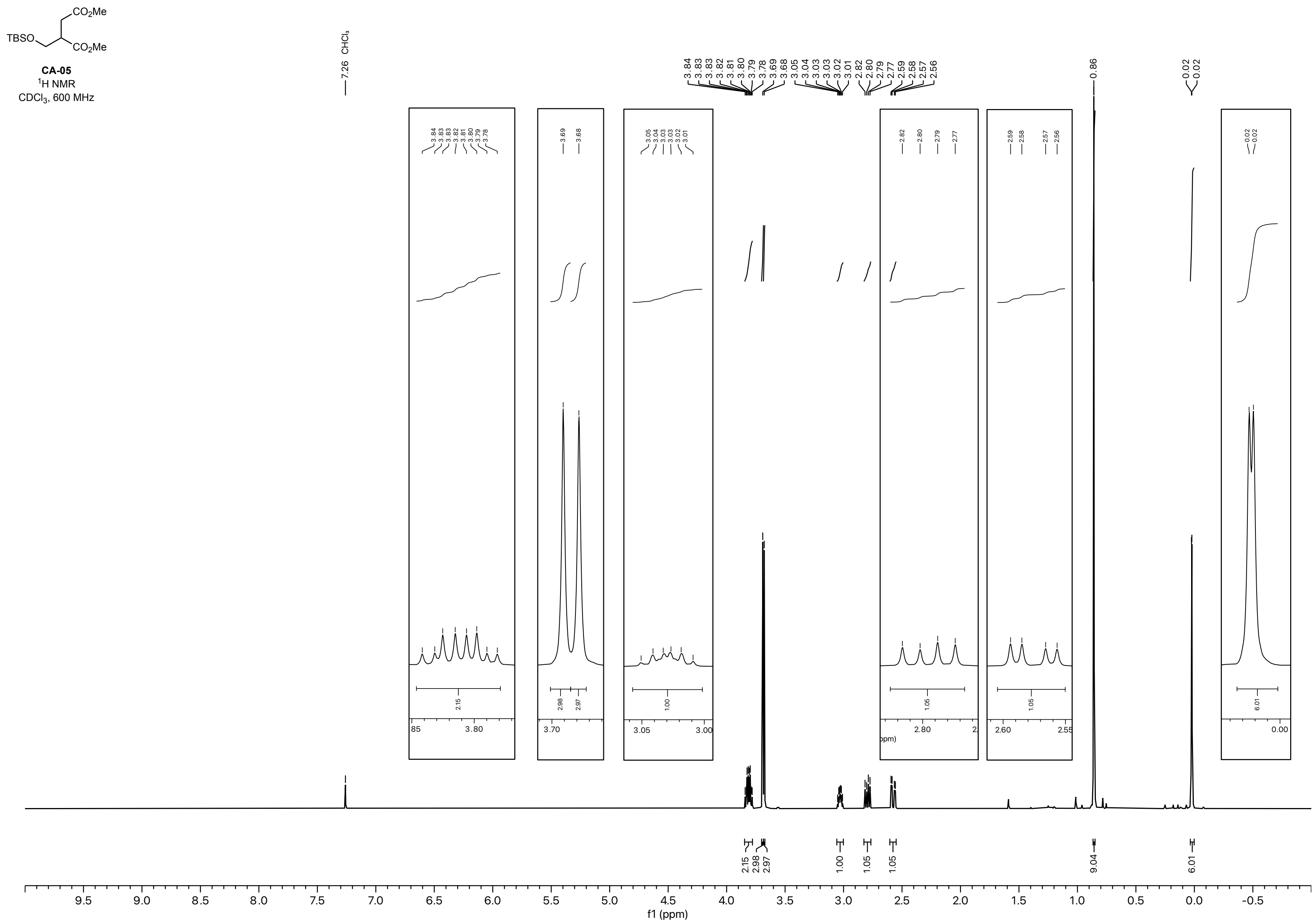
TBSO
CO_2Me
CO_2Me
CA-05
^{1}H NMR
$CDCl_3$, 600 MHz
7.26 $CHCl_3$
f1 (ppm)

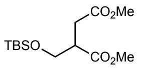

CA-05
^{13}C NMR
$CDCl_3$, 150 MHz

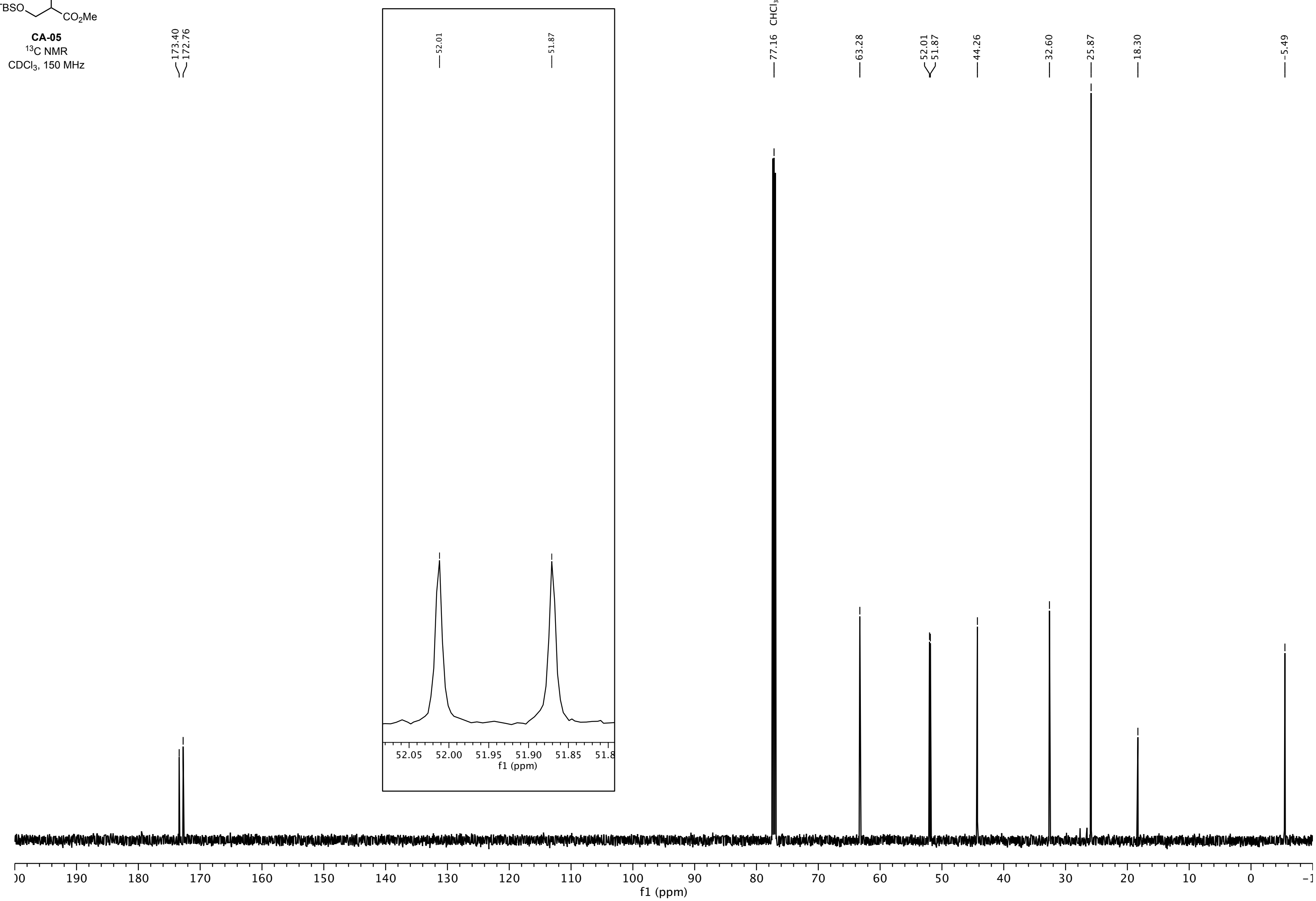

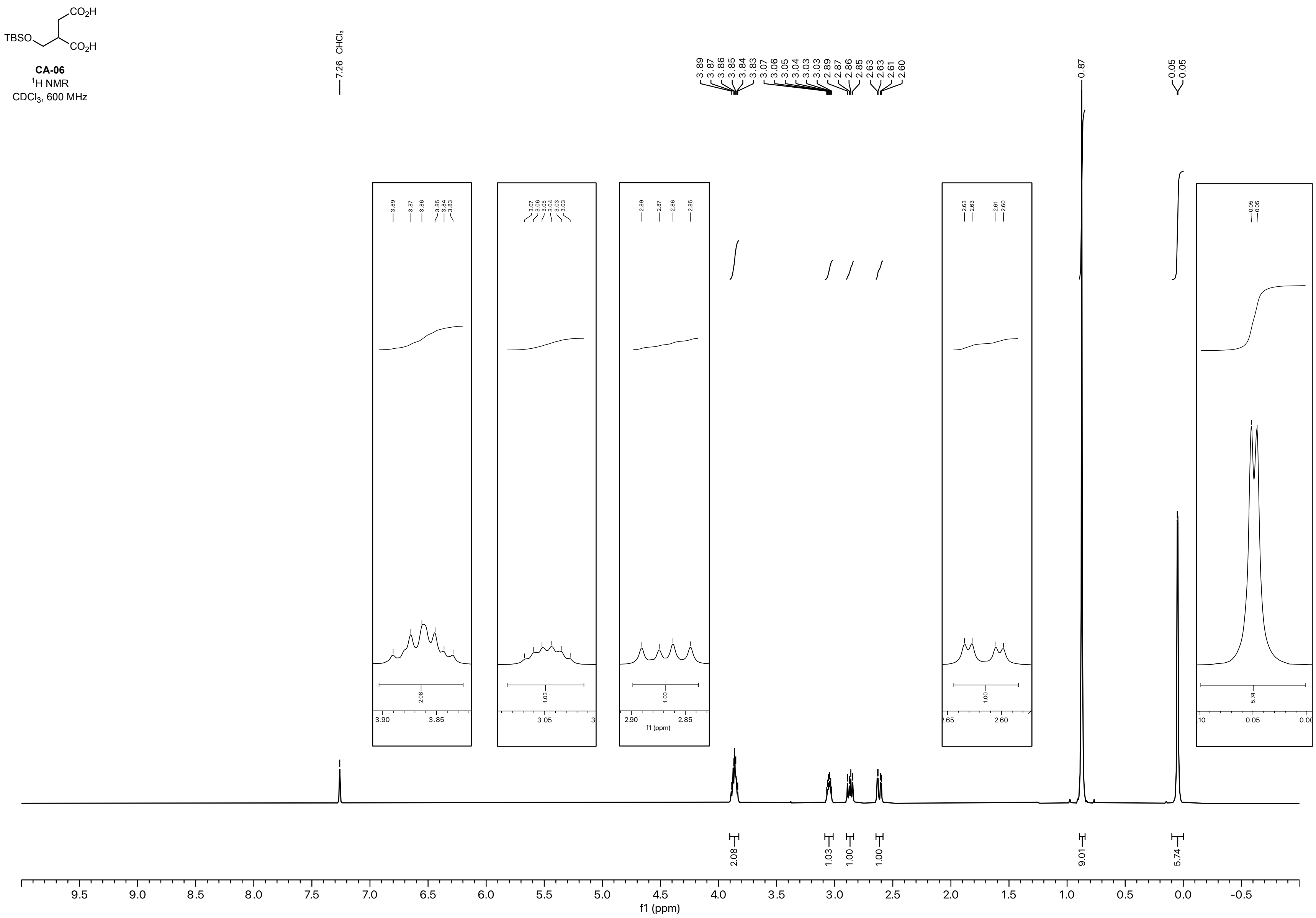

TBSO
CO2H
CO2H
CA-06
1H NMR
CDCl3, 600 MHz
7.26 CHCl3
3.89
3.87
3.86
3.85
3.84
3.83
3.07
3.06
3.05
3.04
3.03
3.03
2.89
2.87
2.86
2.85
2.63
2.63
2.61
2.60
0.87
0.05
0.05
2.08
1.03
1.00
1.00
9.01
5.74
f1 (ppm)

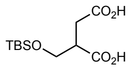

CA-06
^{13}C NMR
$CDCl_3$, 100 MHz

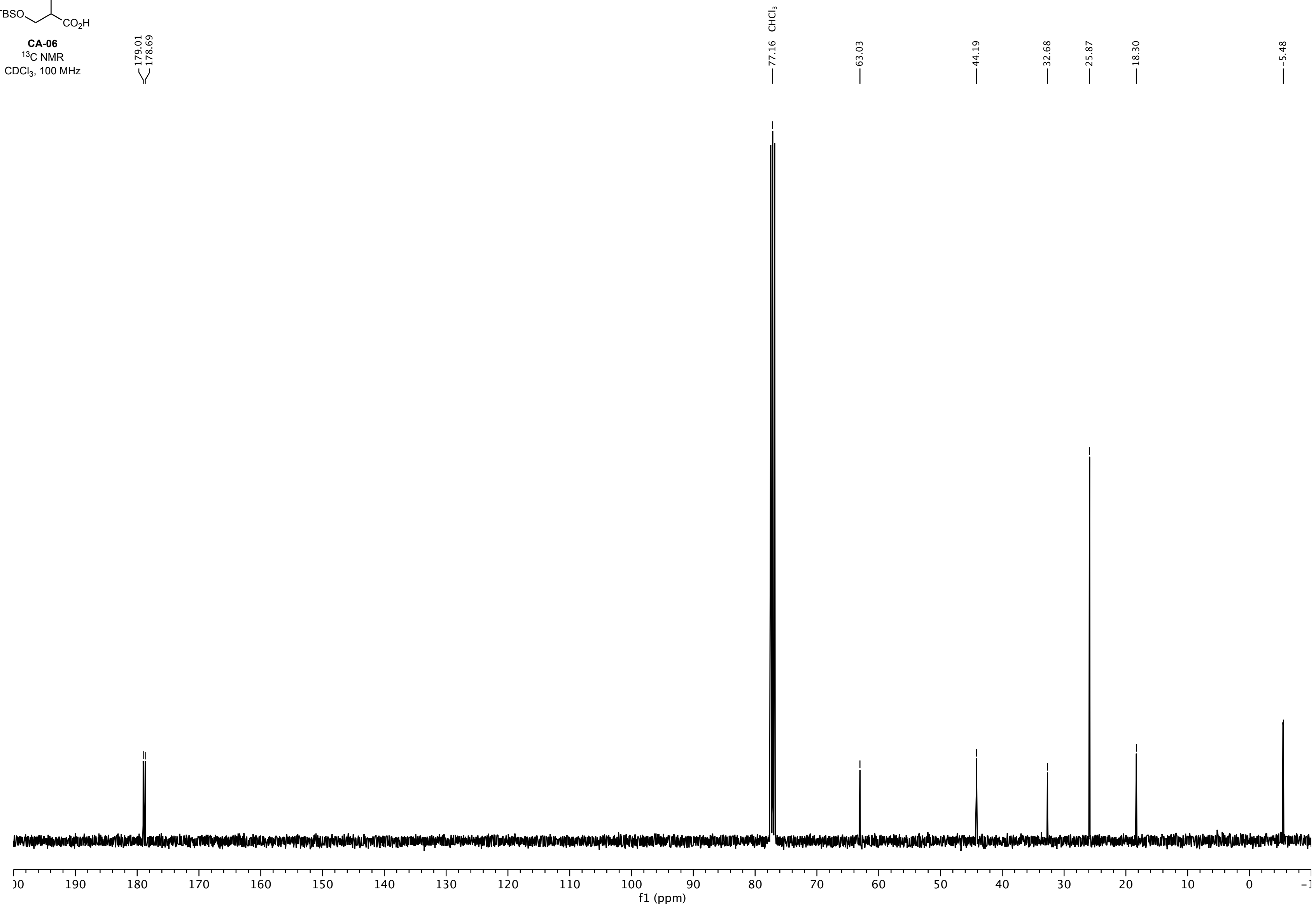

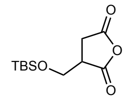

CA-07

^{1}H NMR

$CDCl_3$, 600 MHz

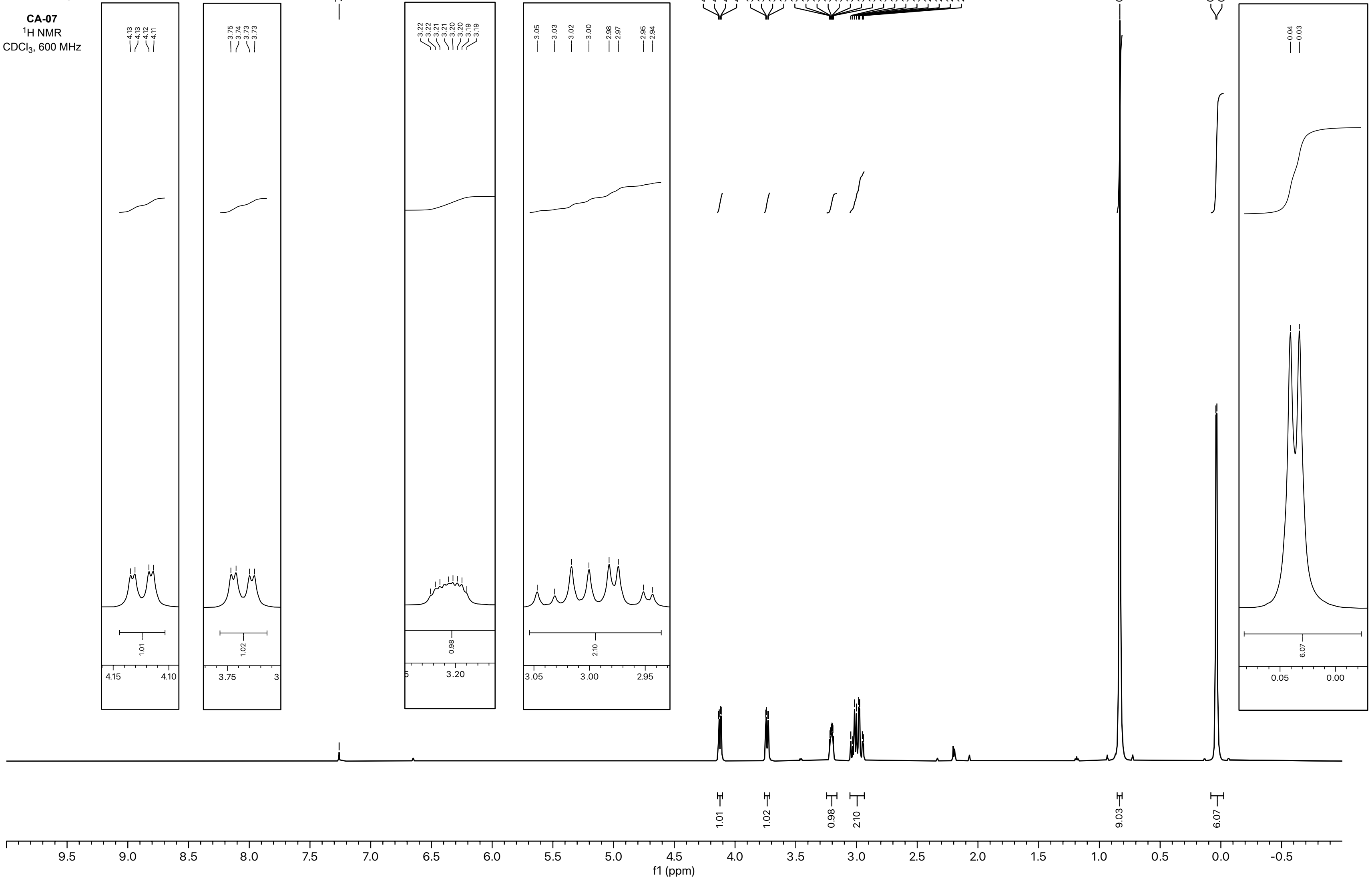

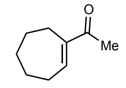

CA-09
^{1}H NMR
$CDCl_3$, 600 MHz

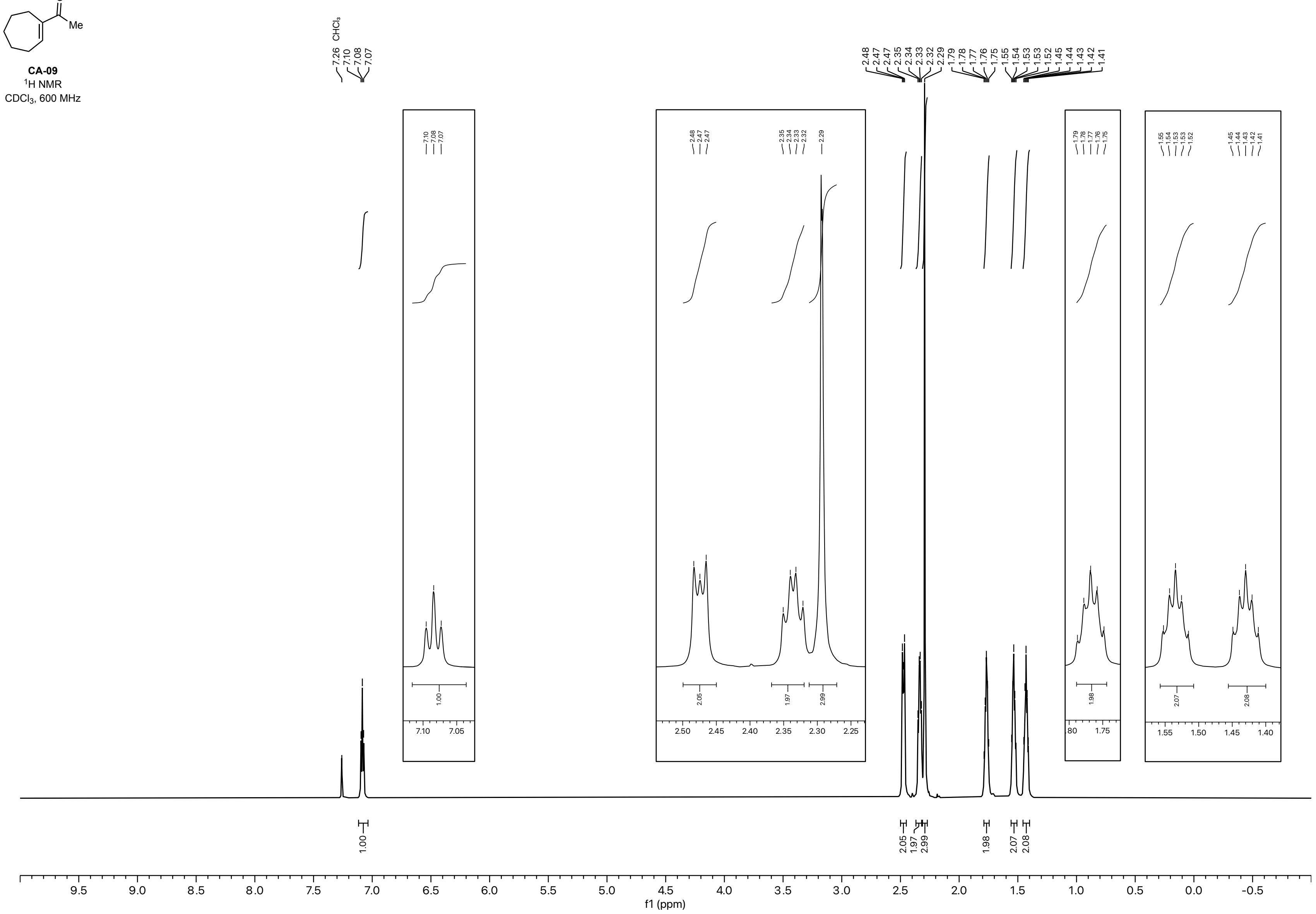

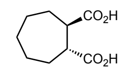

CA-11
^{1}H NMR
$CDCl_3$, 400 MHz

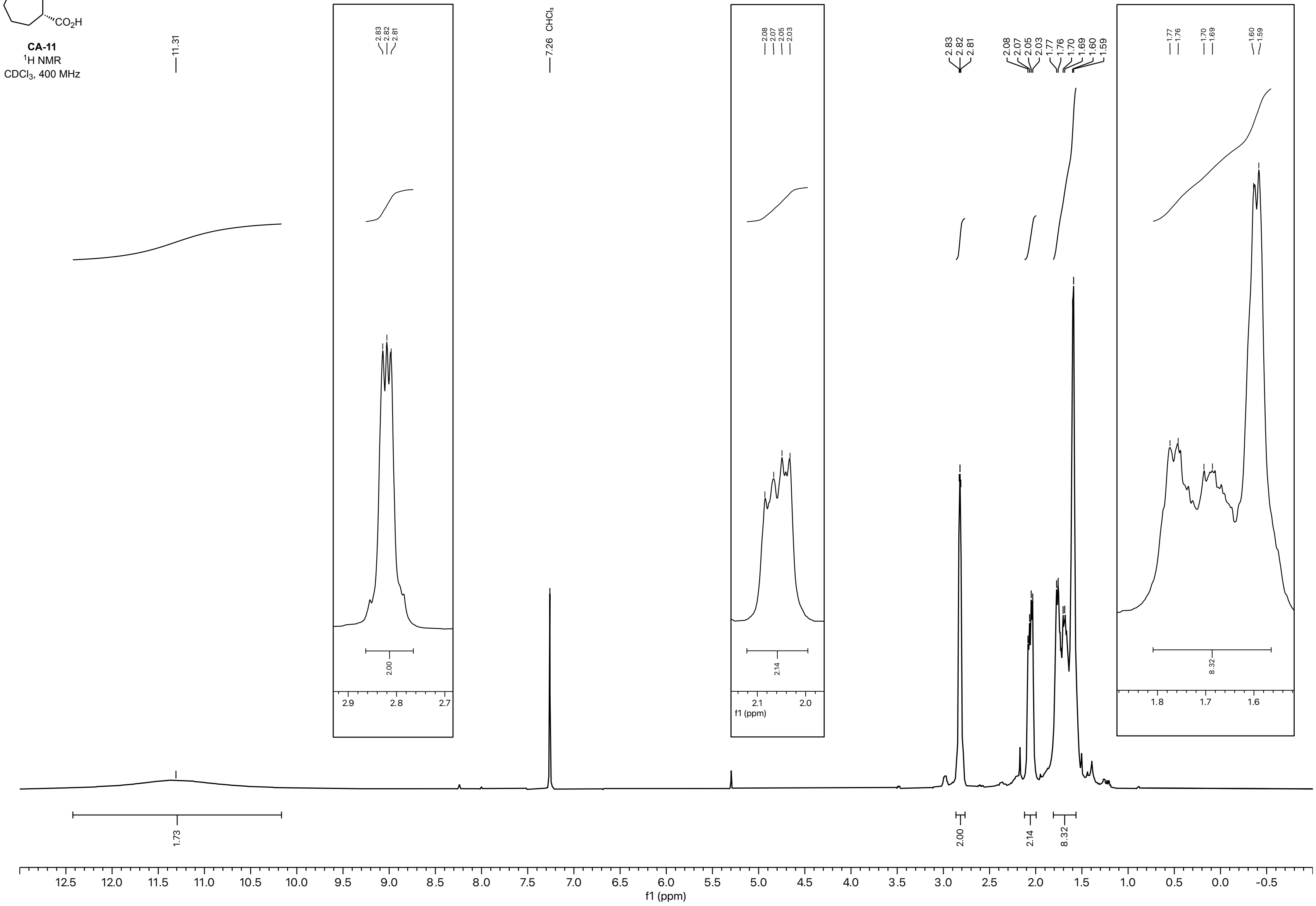

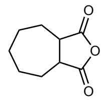

CA-12
[single diastereoisomer,
stereochemistry not confirmed,
presumed inconsequential]

^{1}H NMR
$CDCl_3$, 600 MHz

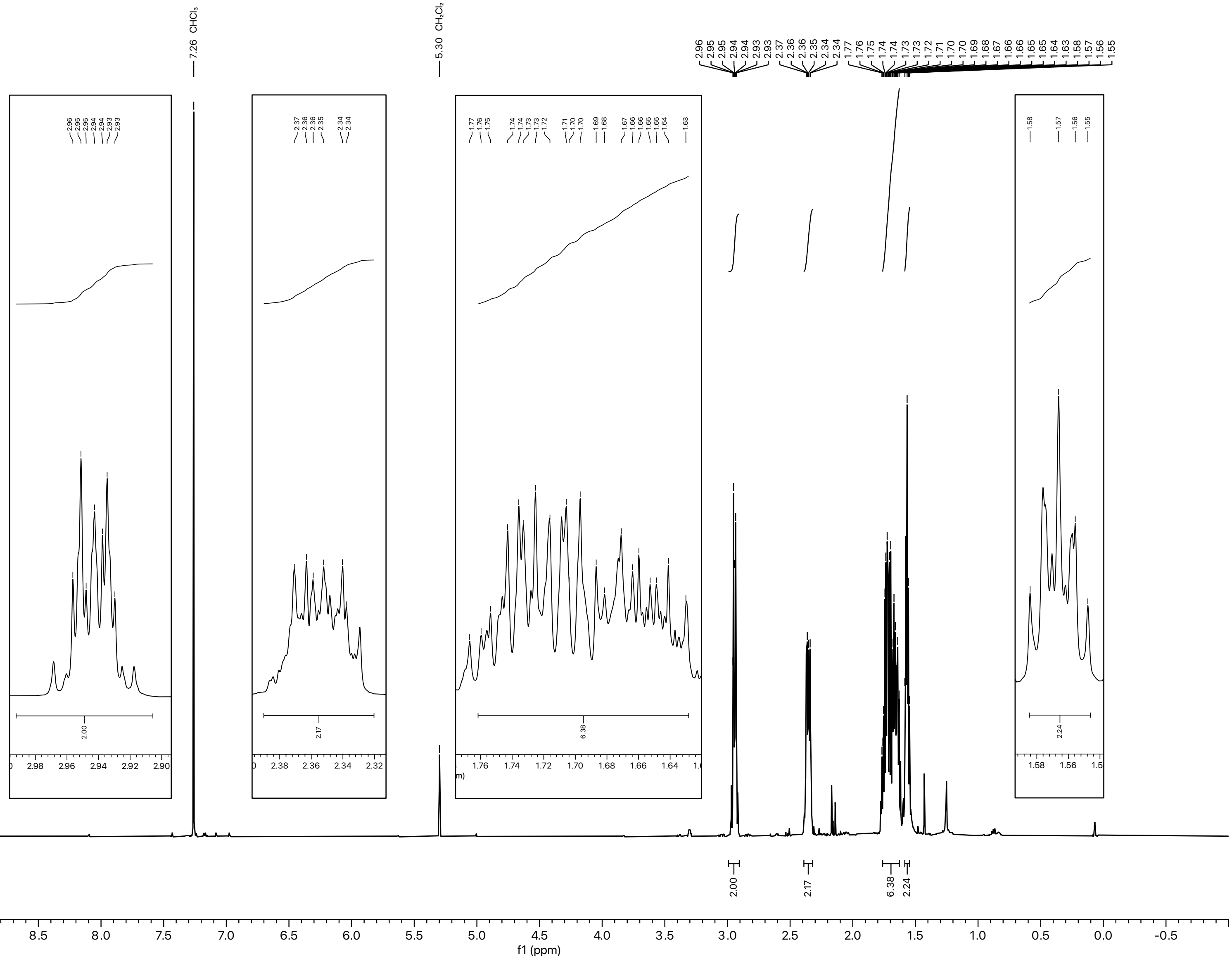

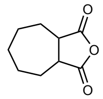

CA-12
[single diastereoisomer,
stereochemistry not confirmed,
presumed inconsequential]

^{13}C NMR
$CDCl_3$, 150 MHz

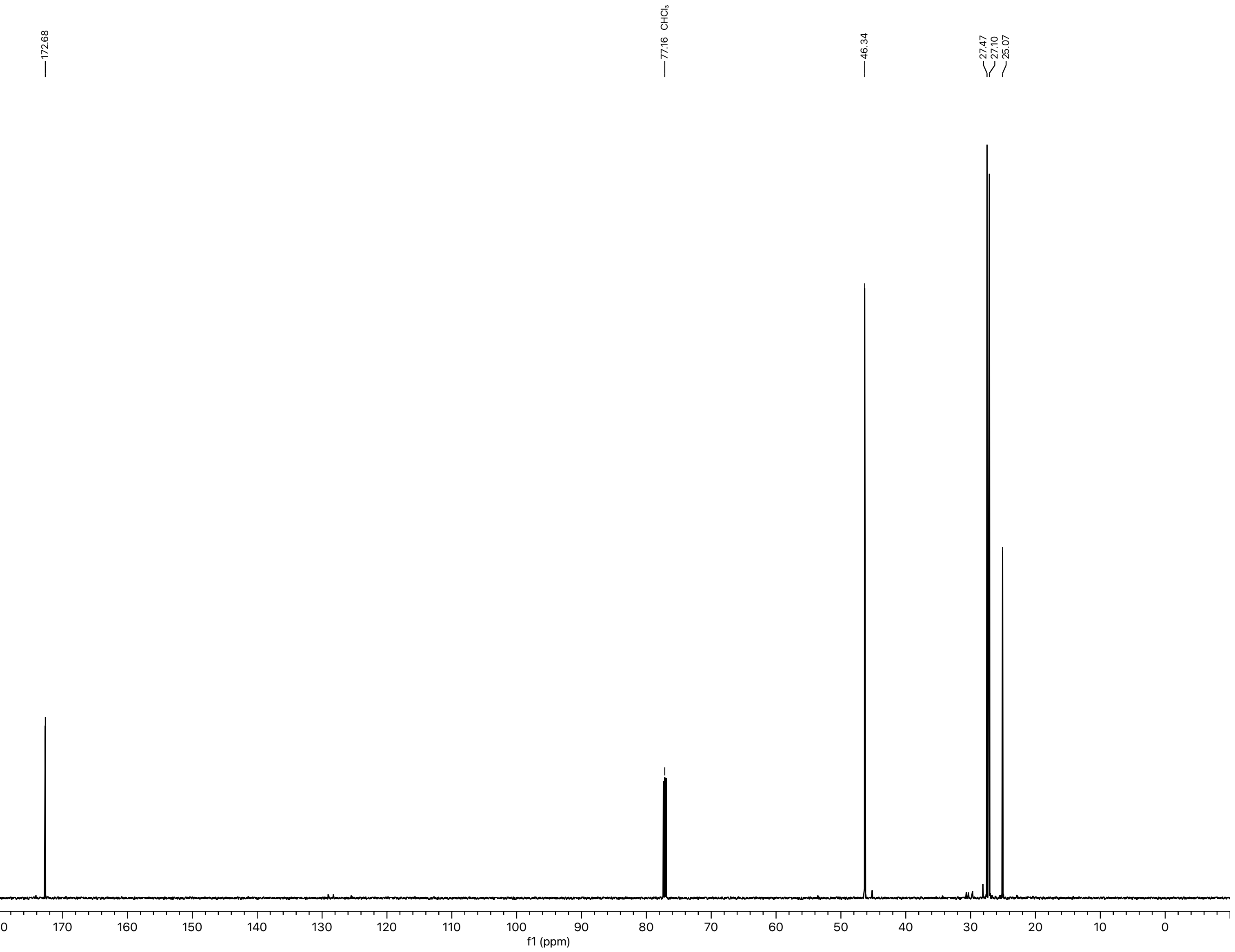

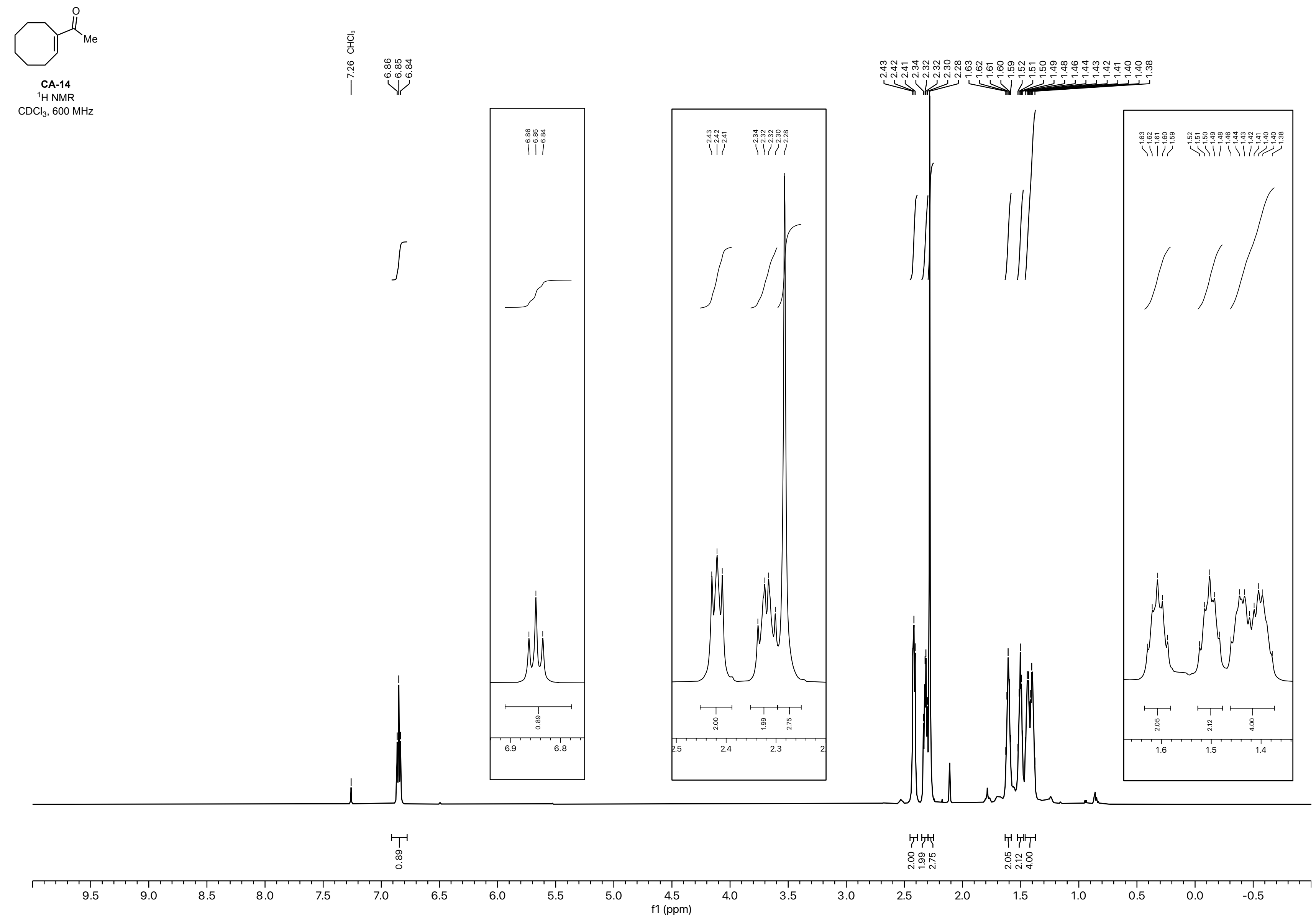

Me
O
CA-14
1H NMR
CDCl3, 600 MHz
7.26 CHCl3
6.86
6.85
6.84
2.43
2.42
2.41
2.34
2.32
2.32
2.30
2.28
1.63
1.62
1.61
1.60
1.59
1.52
1.51
1.50
1.49
1.48
1.46
1.44
1.43
1.42
1.41
1.40
1.40
1.38
0.89
2.00
1.99
2.75
2.05
2.12
4.00
f1 (ppm)

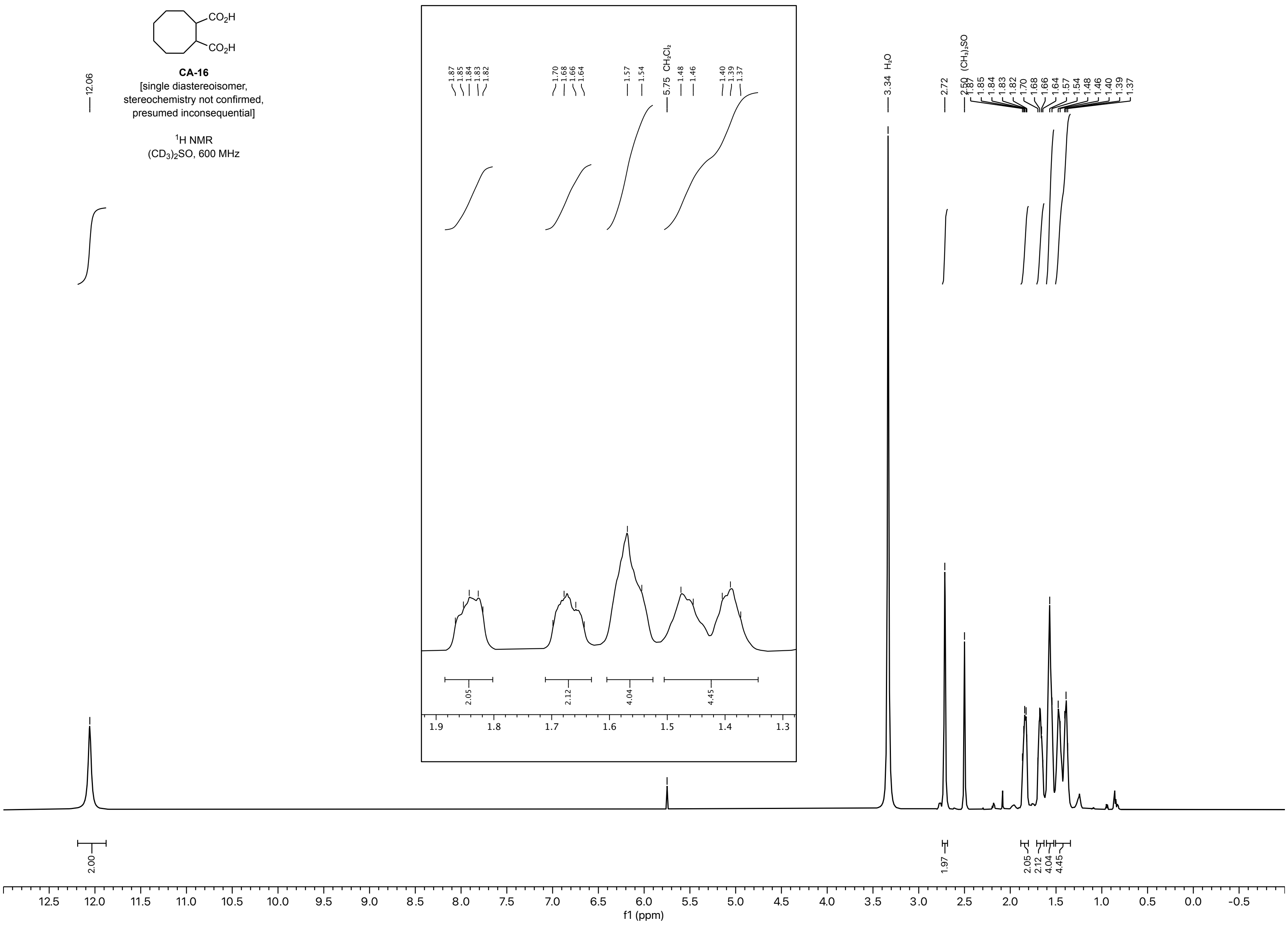

CO_2H
CO_2H
CA-16
[single diastereoisomer, stereochemistry not confirmed, presumed inconsequential]
^1H NMR
$(CD_3)_2SO$, 600 MHz
12.06
5.75 CH_2Cl_2
3.34 H_2O
2.72
2.50 $(CH_3)_2SO$
1.87 1.85 1.84 1.83 1.82 1.70 1.68 1.66 1.64 1.57 1.54 1.48 1.46 1.40 1.39 1.37
2.00
1.97
2.05 2.12 4.04 4.45
f1 (ppm)

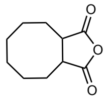

CA-17
[two diastereoisomers,
stereochemistry not confirmed,
presumed inconsequential]

only major diastereoisomer indicated

^{1}H NMR
$CDCl_3$, 600 MHz

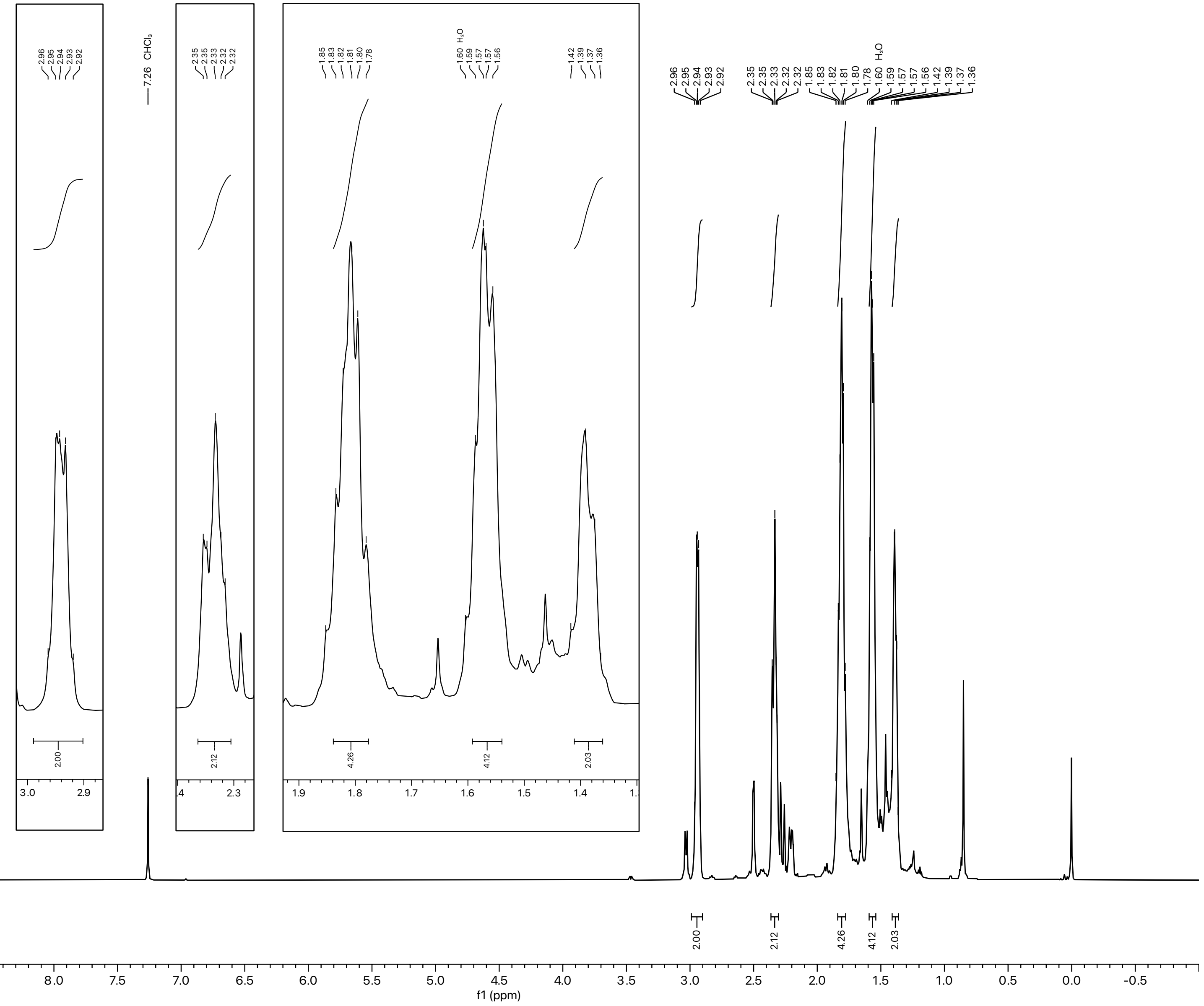

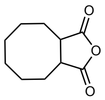

CA-17
[two diastereoisomers,
stereochemistry not confirmed,
presumed inconsequential]

only major diastereoisomer indicated

^{13}C NMR
$CDCl_3$, 100 MHz

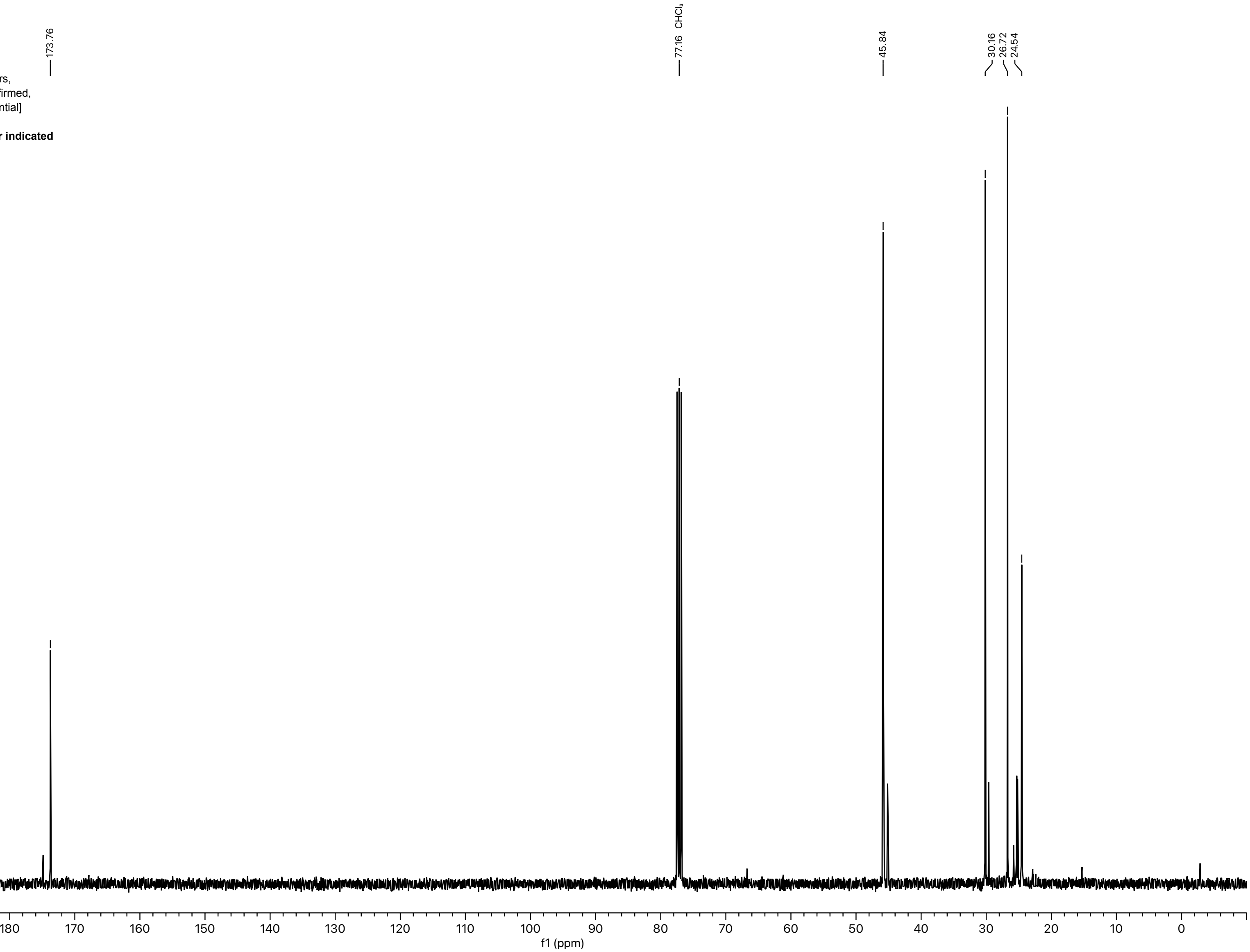

Bis(silyloxy)furans

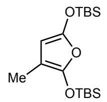

BSF-01
^1H NMR
$CDCl_3$, 400 MHz

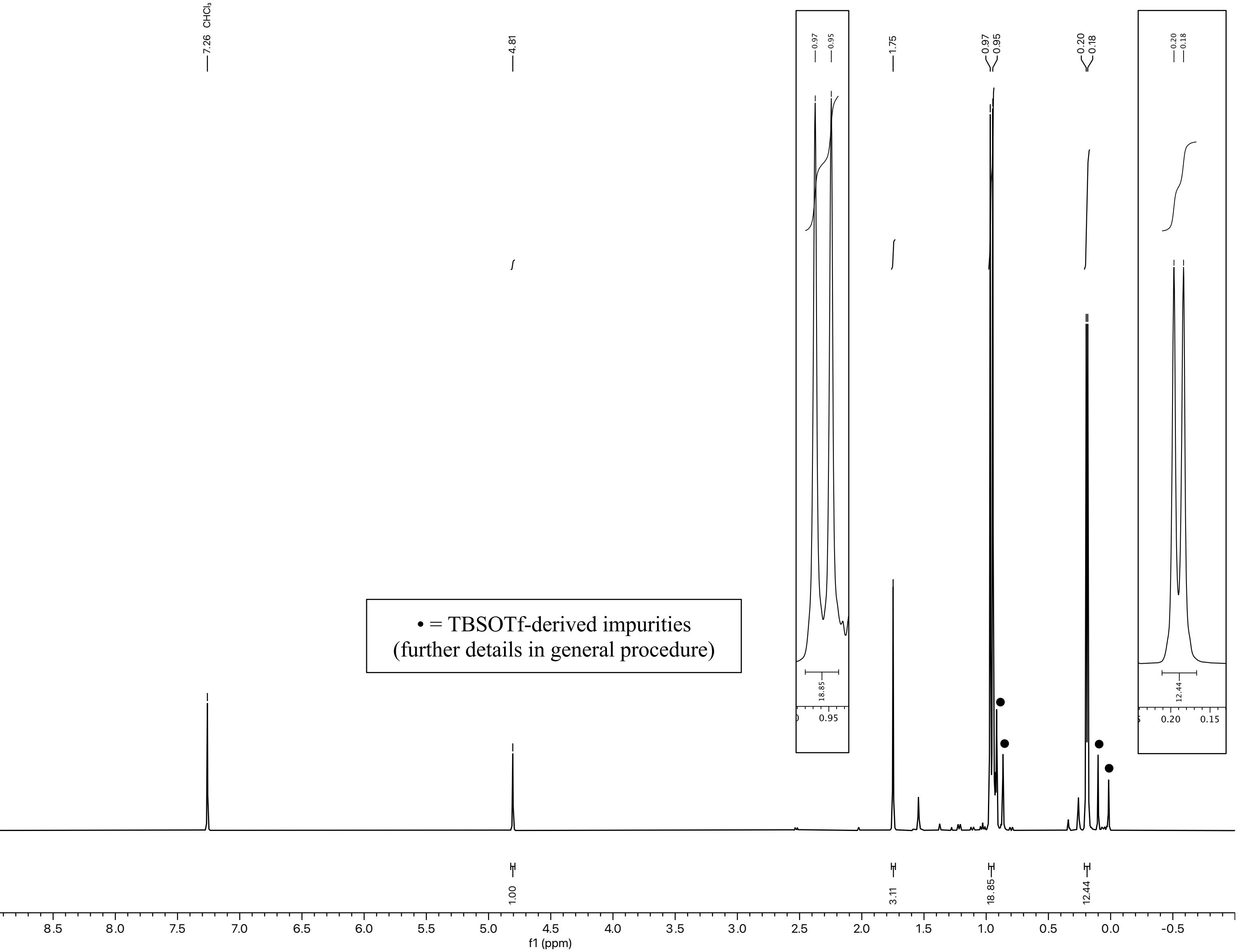

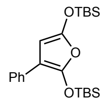

BSF-02
^{1}H NMR (crude)
$CDCl_3$, 400 MHz

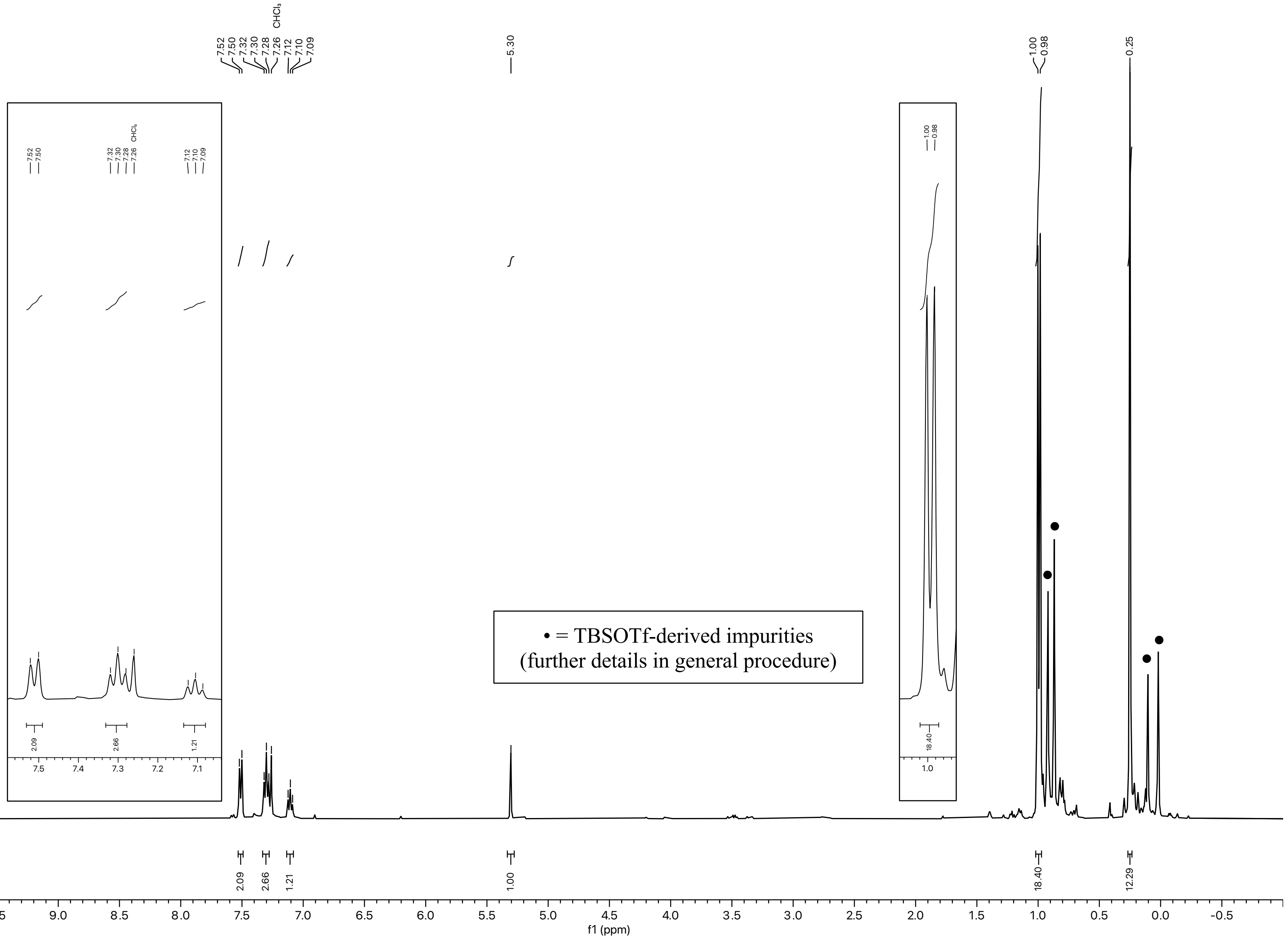

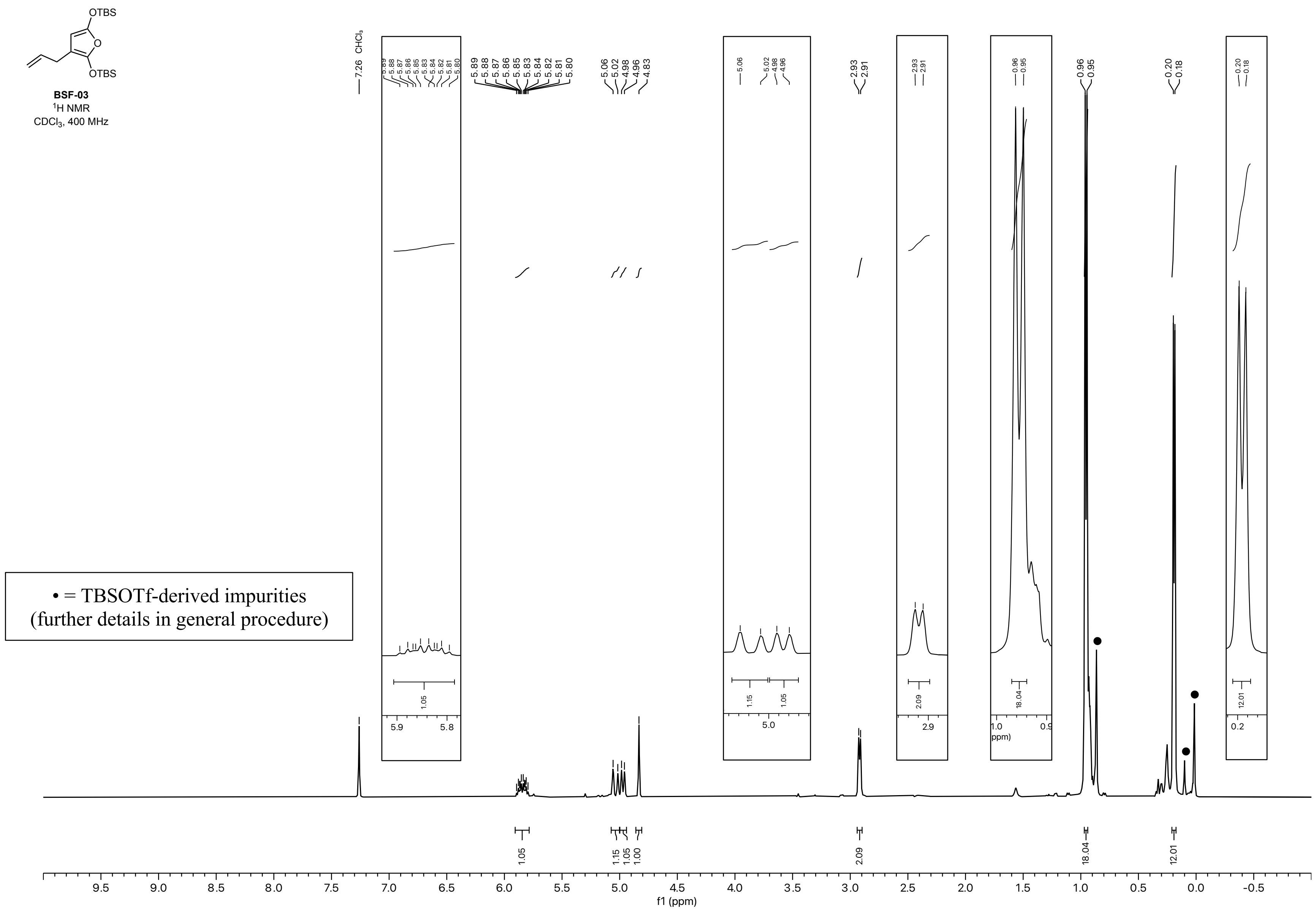
OTBS
O
OTBS
BSF-03
1H NMR
CDCl3, 400 MHz
• = TBSOTf-derived impurities
(further details in general procedure)
7.26 CHCl3
f1 (ppm)

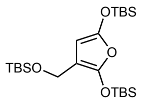

BSF-04
^1H NMR
$CDCl_3$, 600 MHz

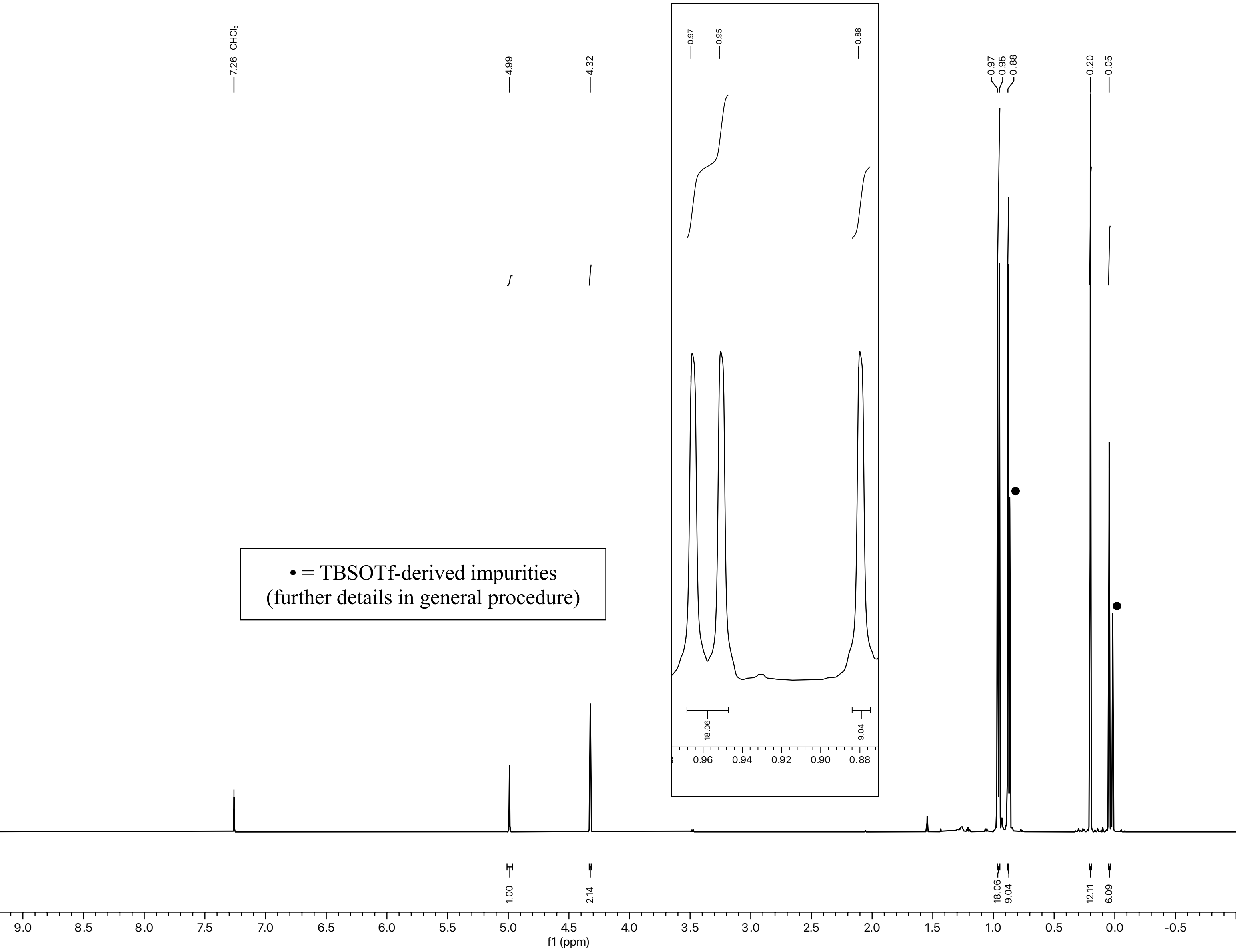

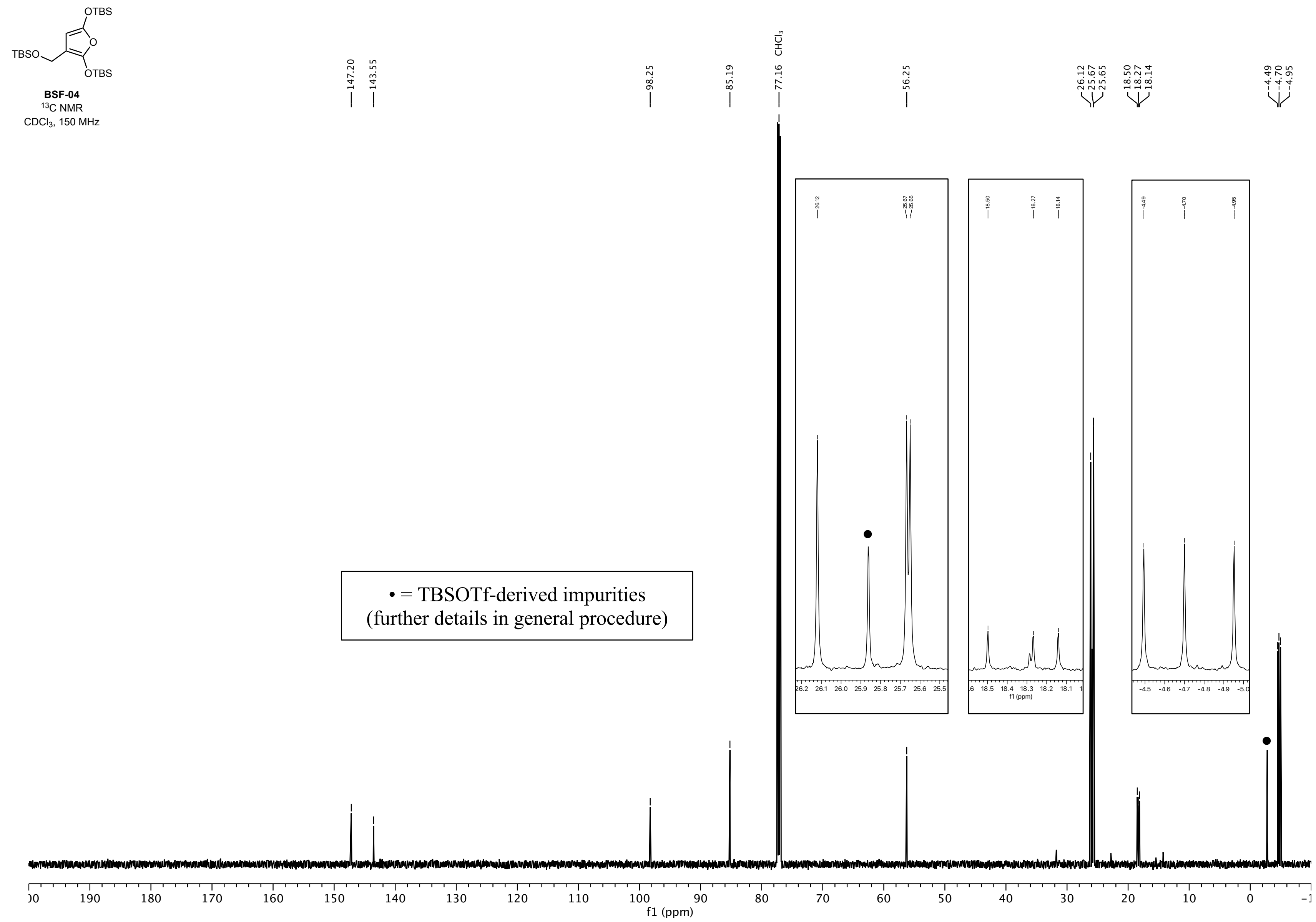
OTBS
TBSO
O
OTBS
BSF-04
^{13}C NMR
$CDCl_3$, 150 MHz
147.20
143.55
98.25
85.19
77.16 $CHCl_3$
56.25
26.12
25.67
25.65
18.50
18.27
18.14
-4.49
-4.70
-4.95
• = TBSOTf-derived impurities
(further details in general procedure)
f1 (ppm)

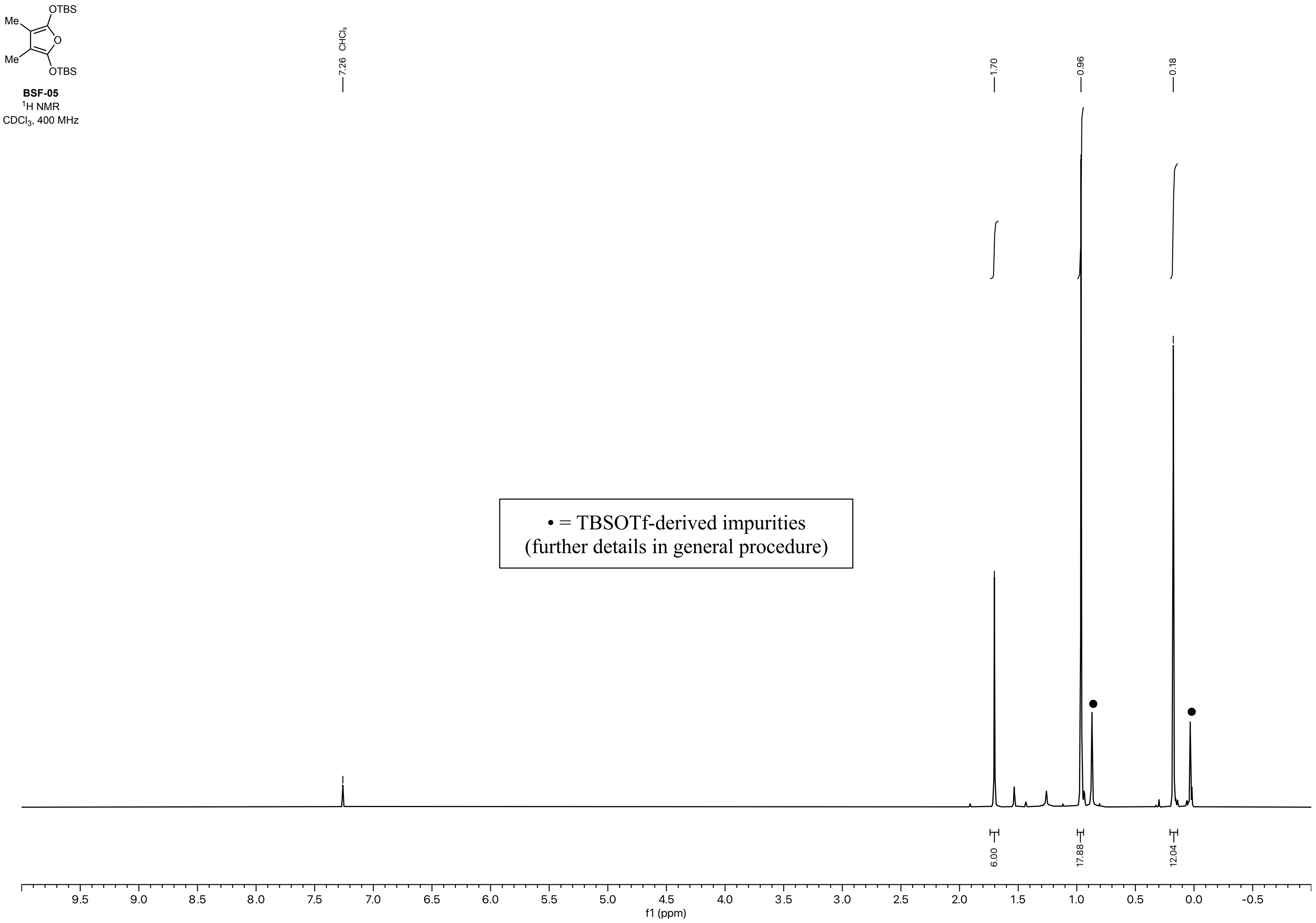

• = TBSOTf-derived impurities
(further details in general procedure)

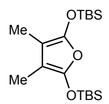

BSF-05
^{13}C NMR
$CDCl_3$, 100 MHz

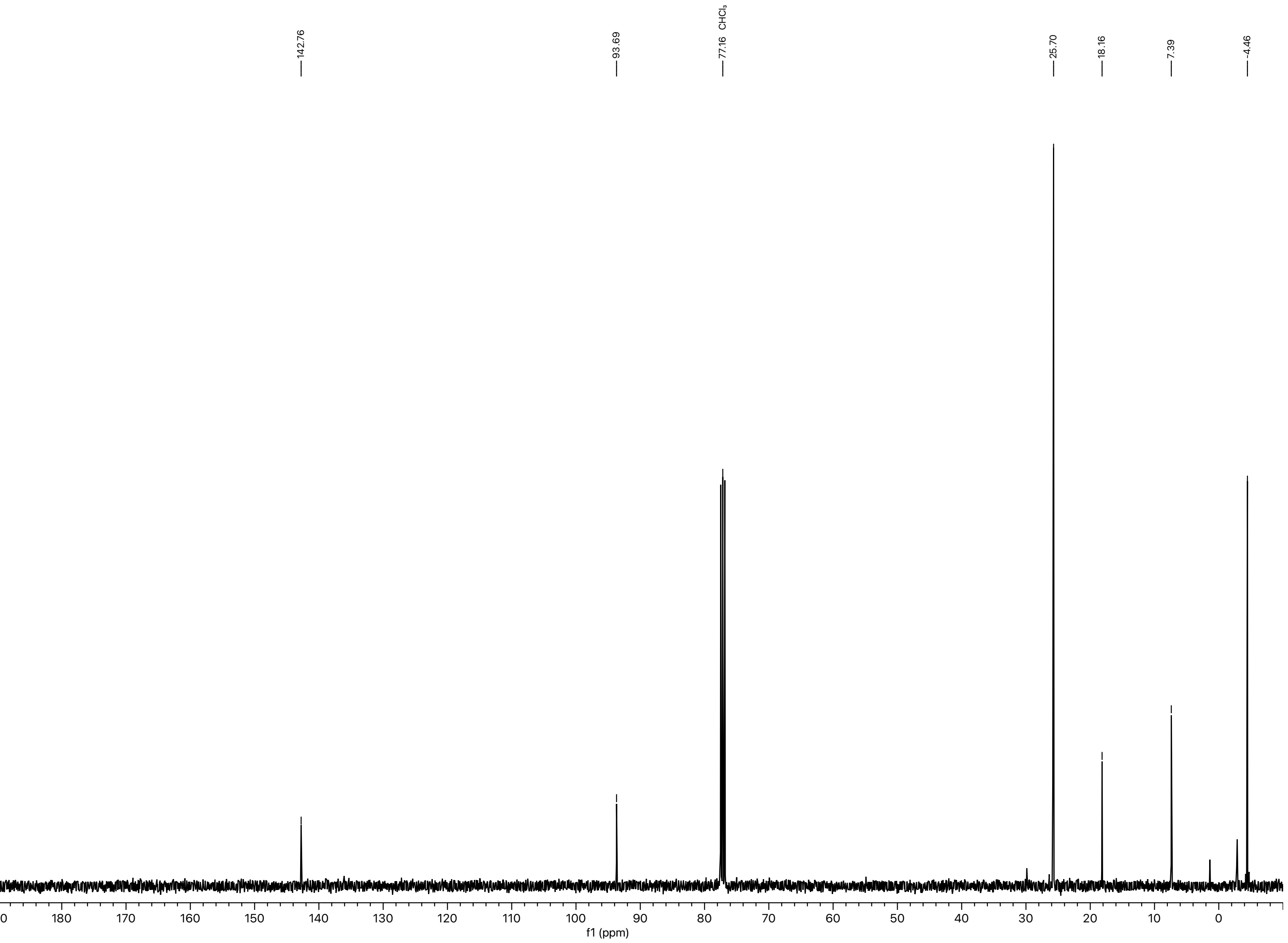

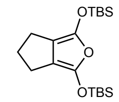

BSF-06
^{1}H NMR (crude)
$CDCl_3$, 400 MHz

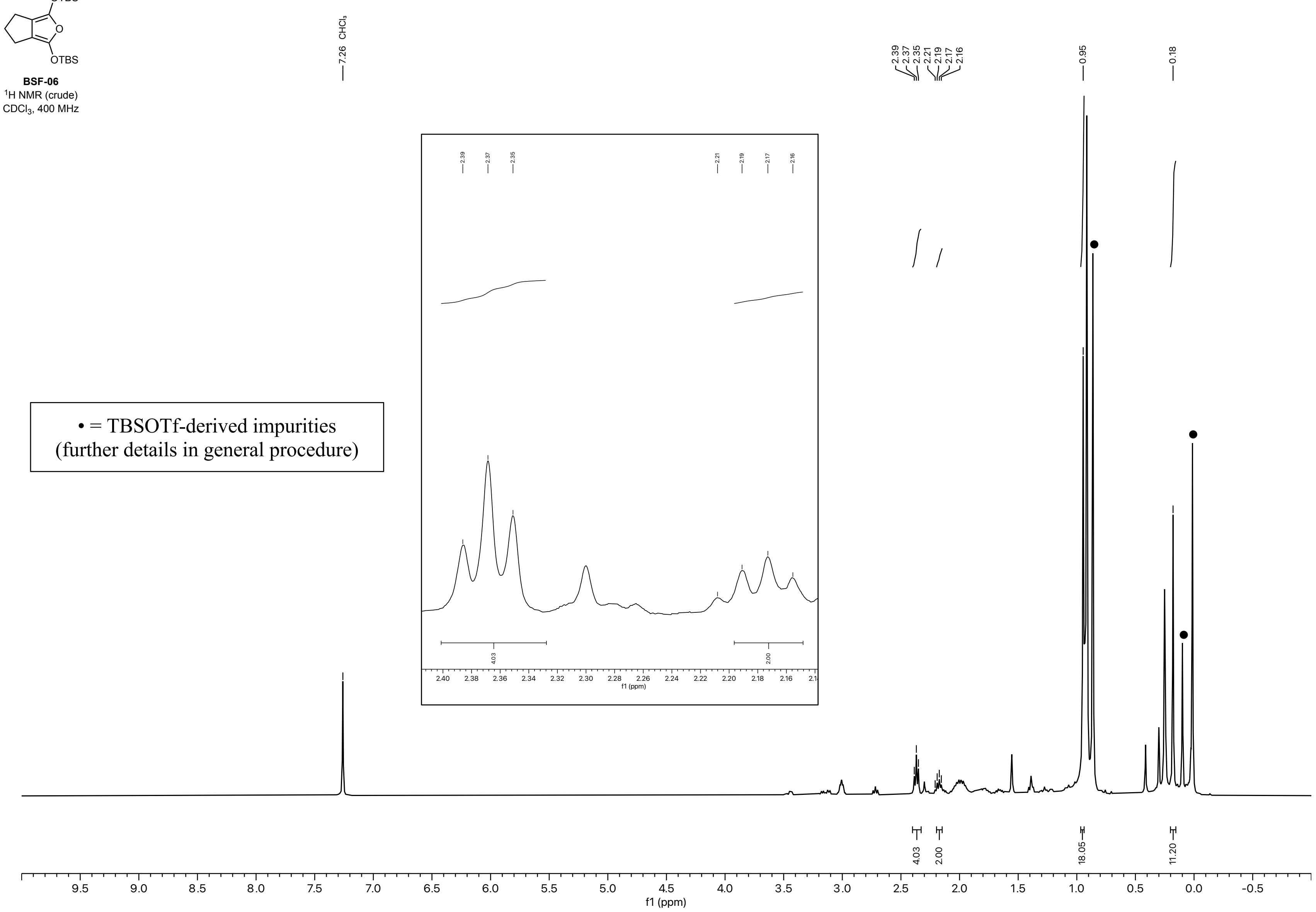

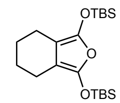

BSF-07 (or **1a**)
^{1}H NMR (crude)
$CDCl_3$, 400 MHz

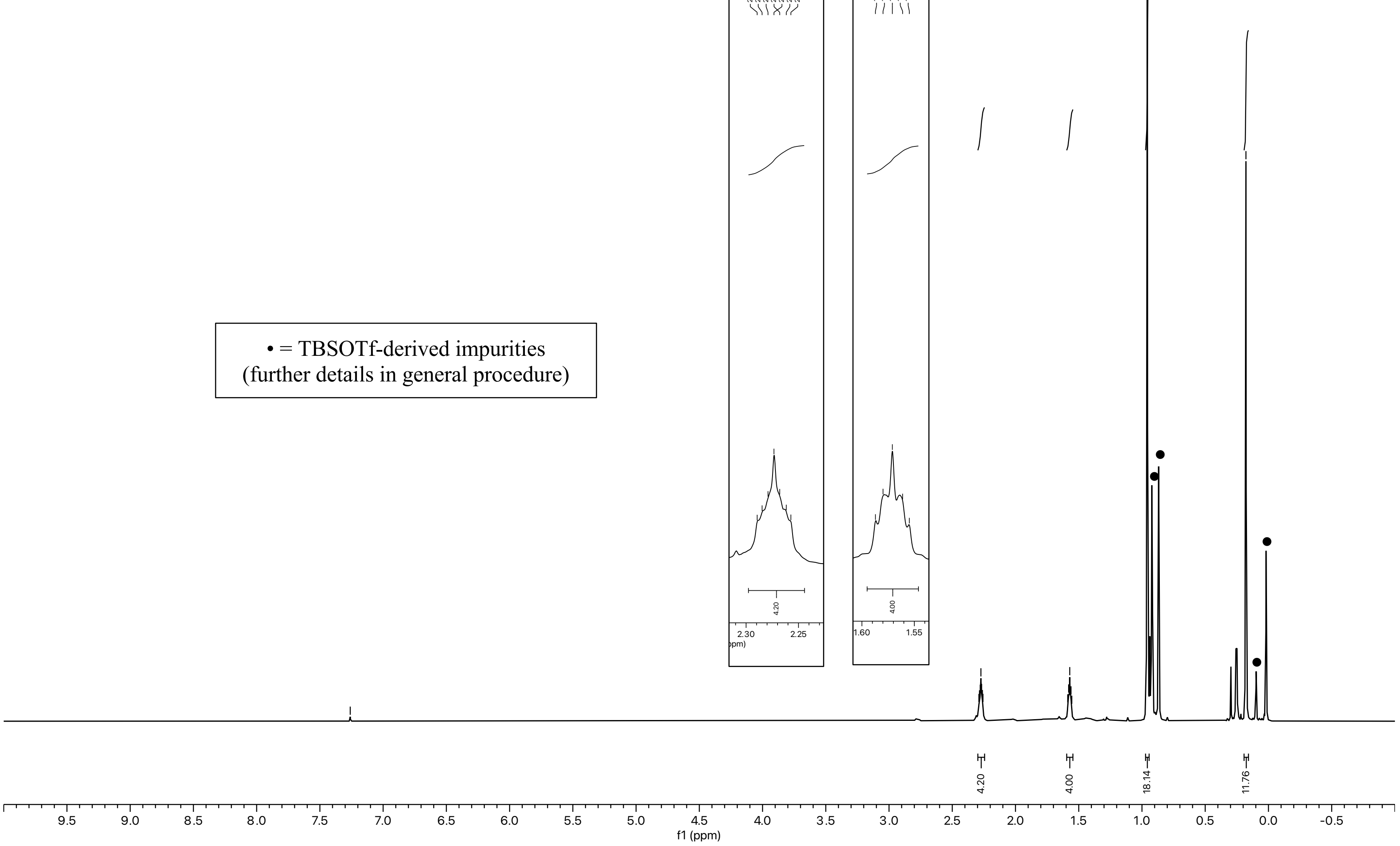

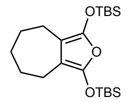

BSF-08
^{1}H NMR
$CDCl_3$, 600 MHz

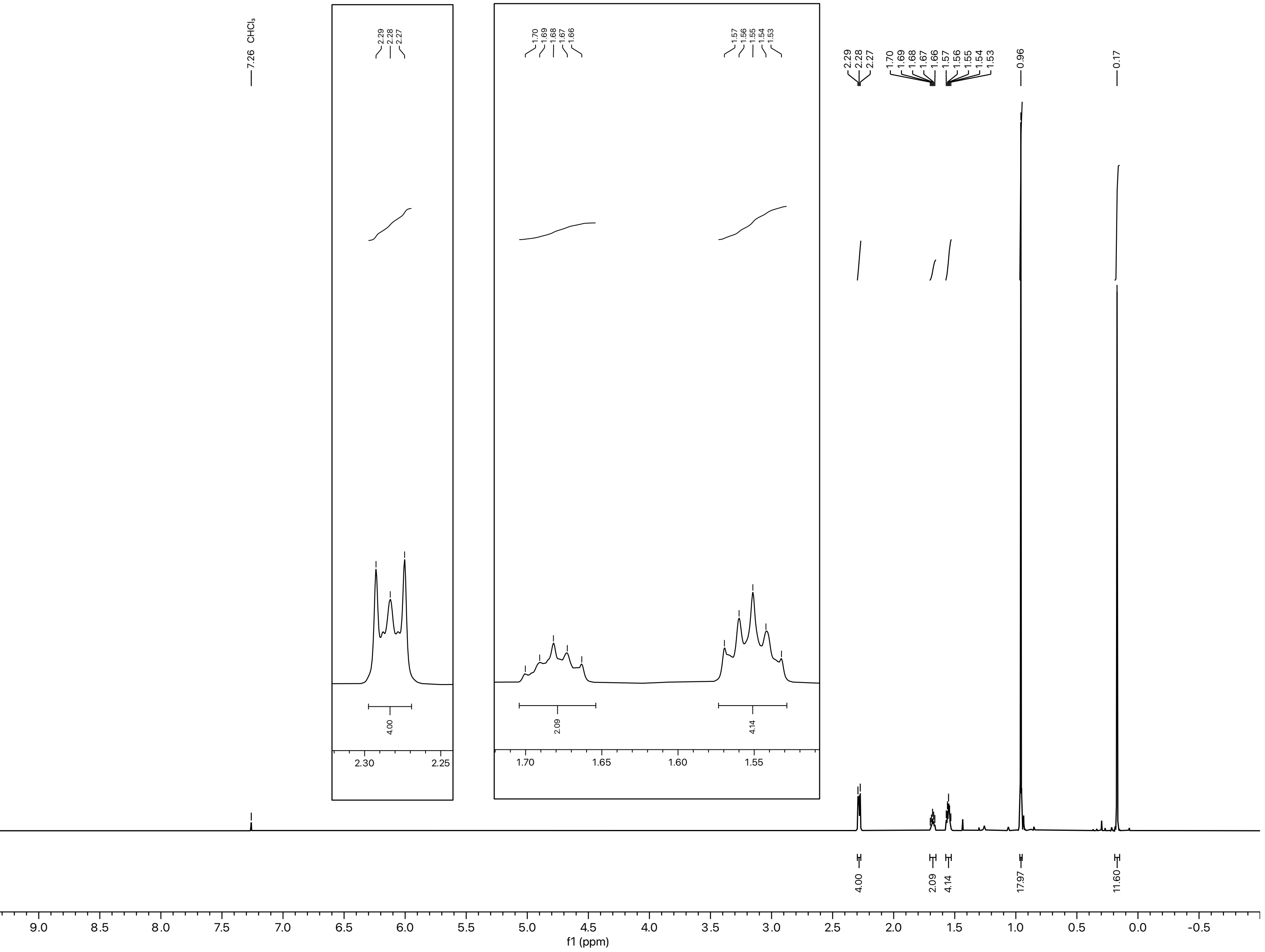

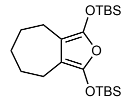

BSF-08
^{13}C NMR
$CDCl_3$, 150 MHz

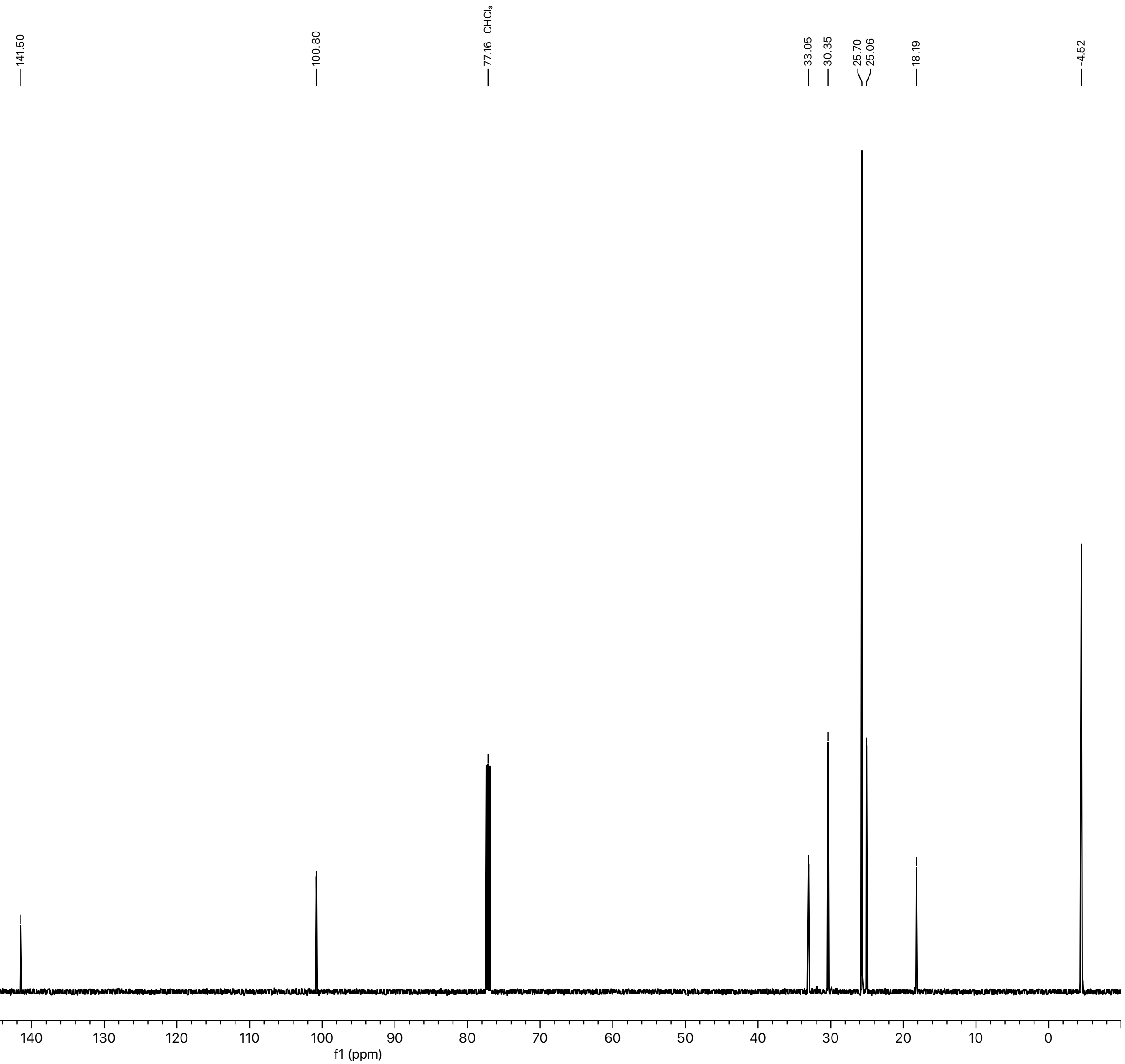

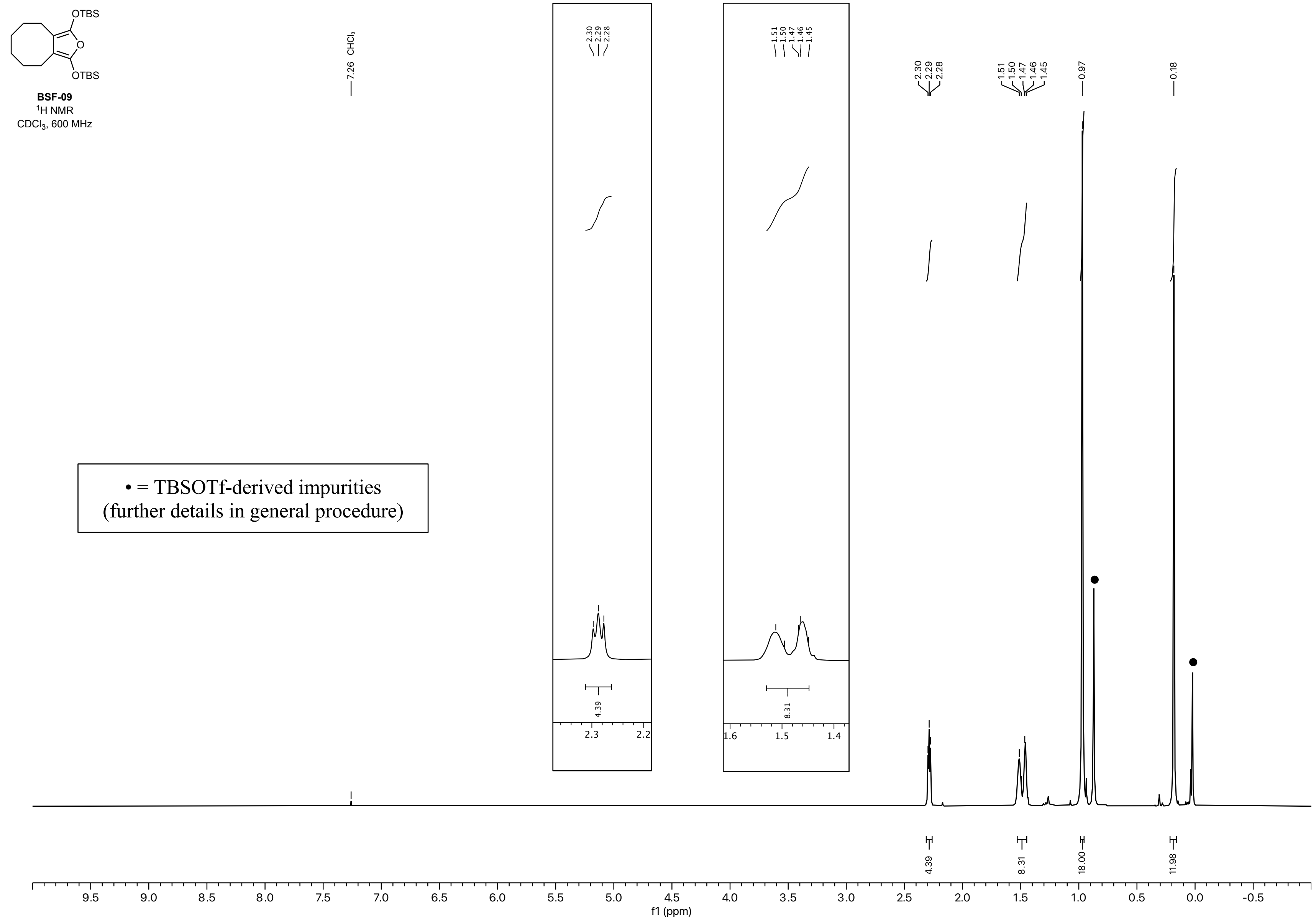
OTBS
O
OTBS
BSF-09
1H NMR
CDCl3, 600 MHz
7.26 CHCl3
• = TBSOTf-derived impurities
(further details in general procedure)
2.30
2.29
2.28
1.51
1.50
1.47
1.46
1.45
0.97
0.18
4.39
8.31
18.00
11.98
f1 (ppm)

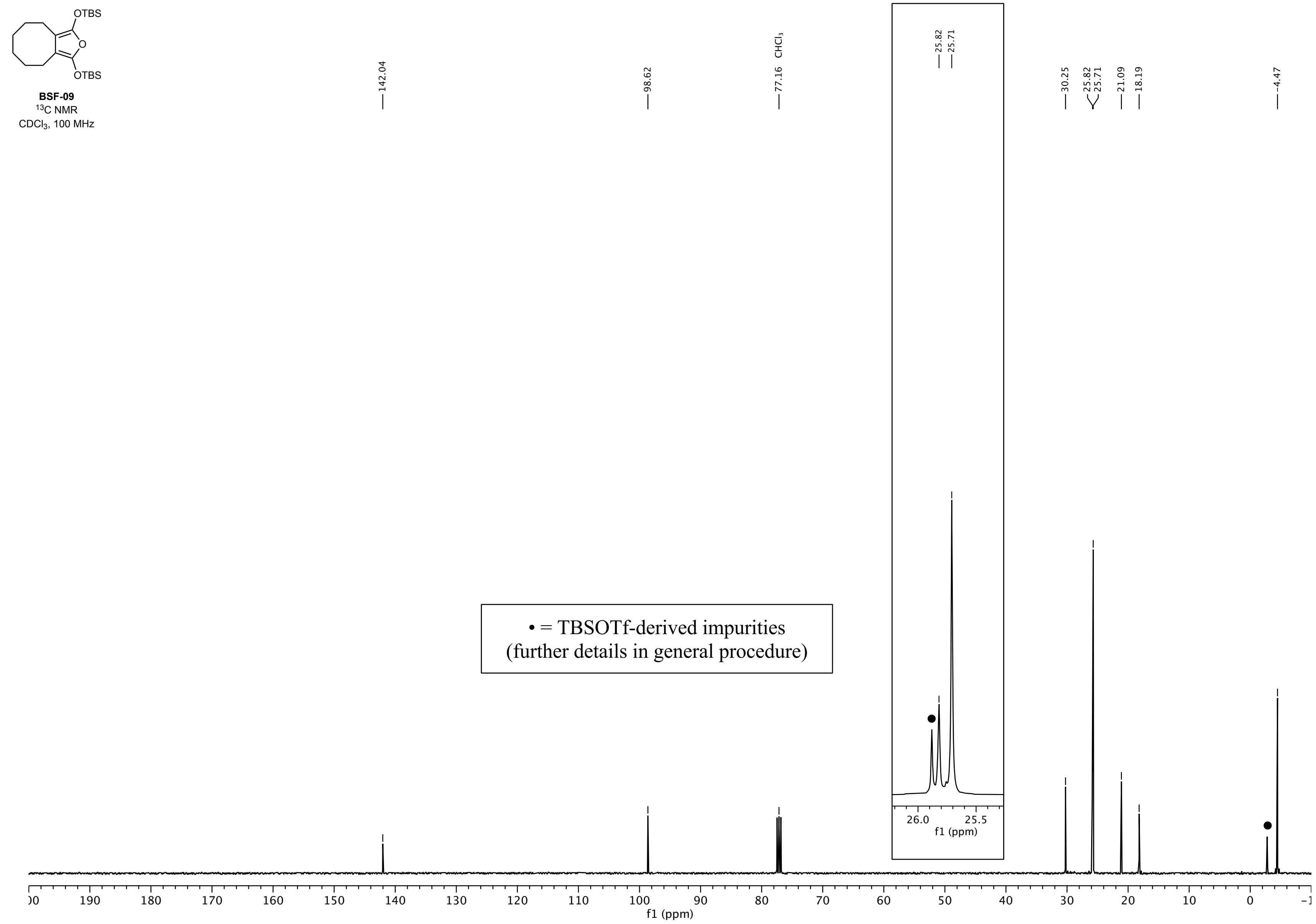

BSF-09
^{13}C NMR
$CDCl_3$, 100 MHz

Synthesis of Ketenimines

Ketenimine Precursors

Amides

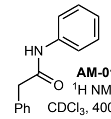

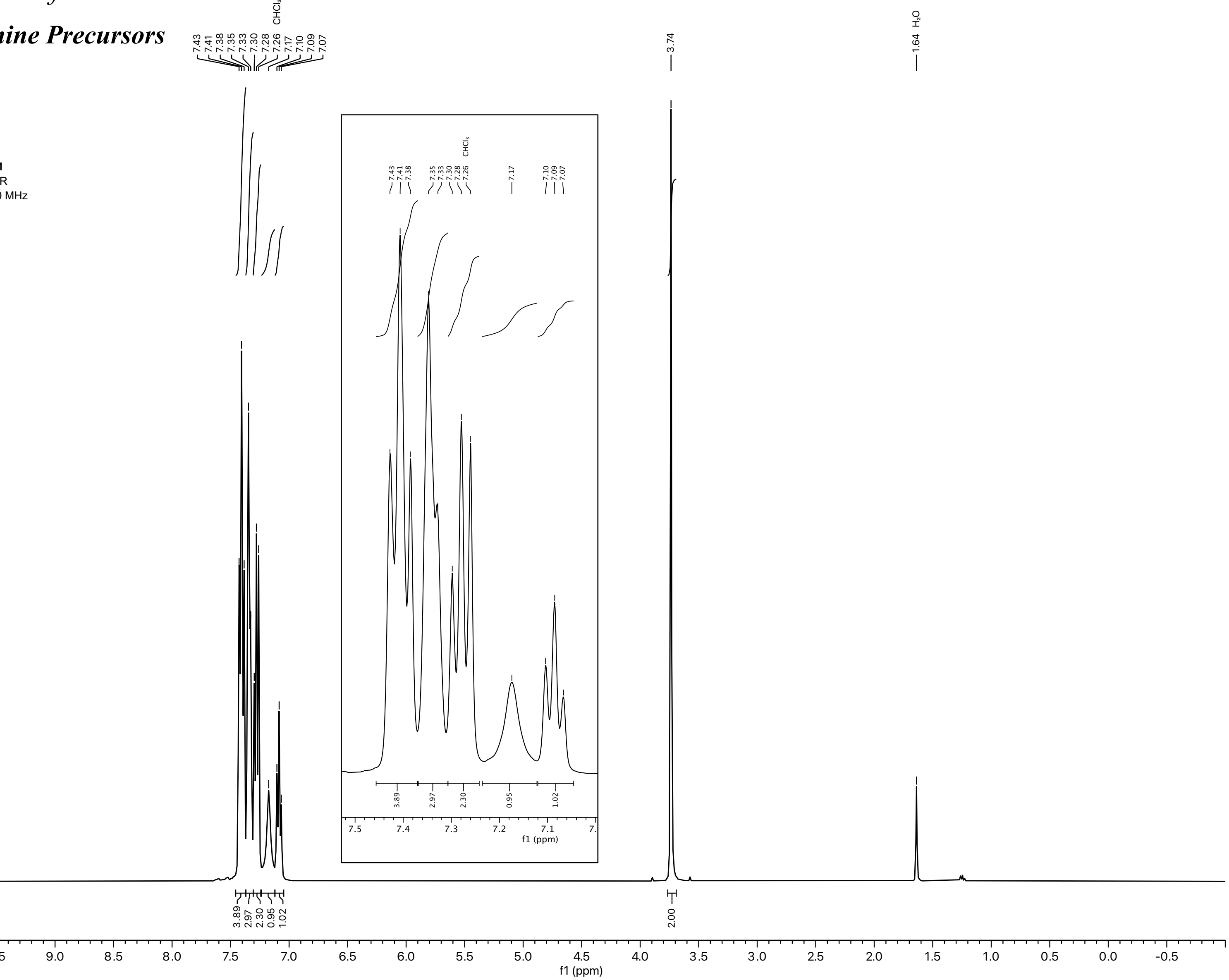

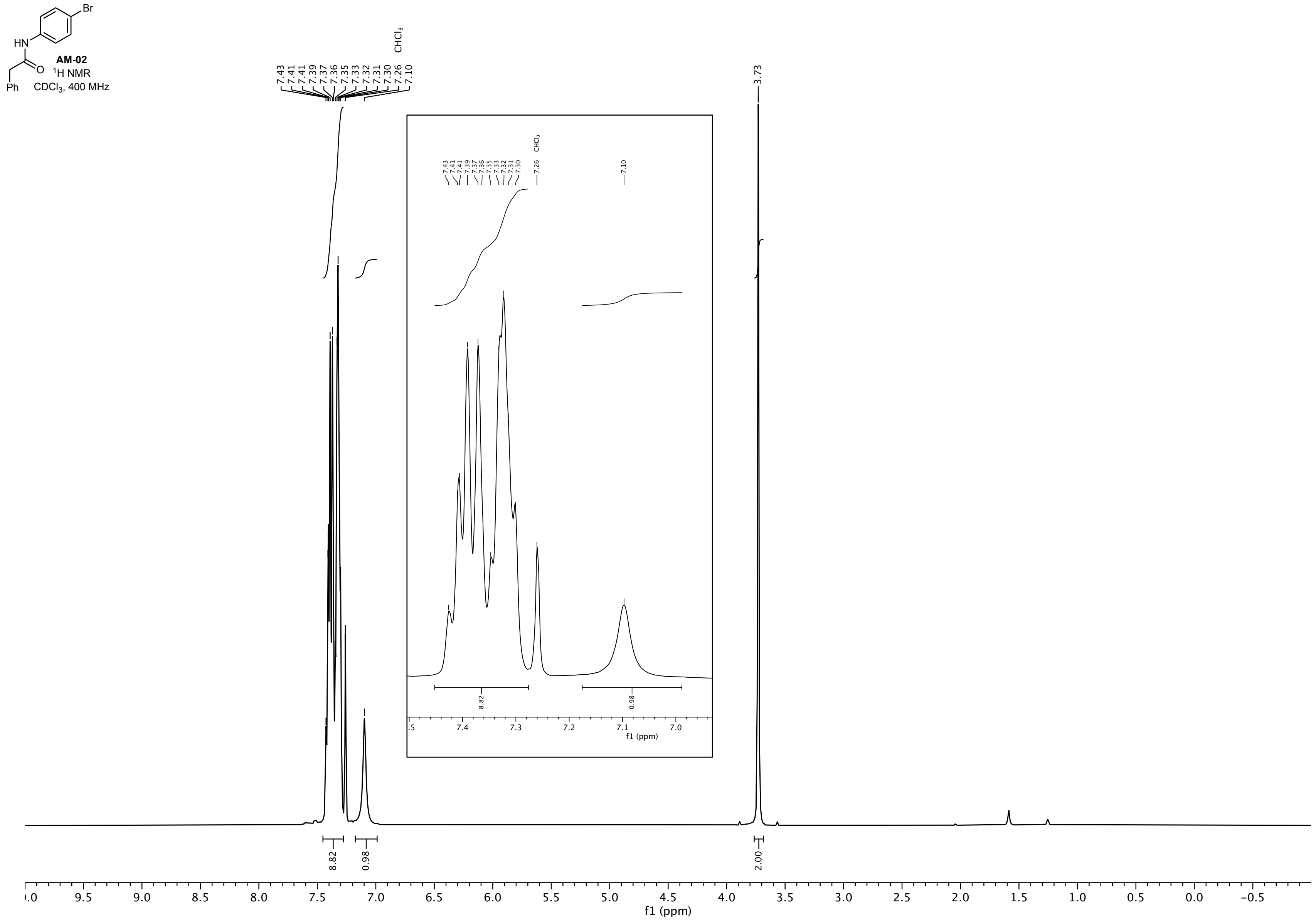
Br
HN
O
Ph
AM-02
1H NMR
CDCl3, 400 MHz
7.43
7.41
7.41
7.39
7.37
7.36
7.35
7.33
7.32
7.31
7.30
7.26
7.10
CHCl3
3.73
8.82
0.98
2.00
f1 (ppm)

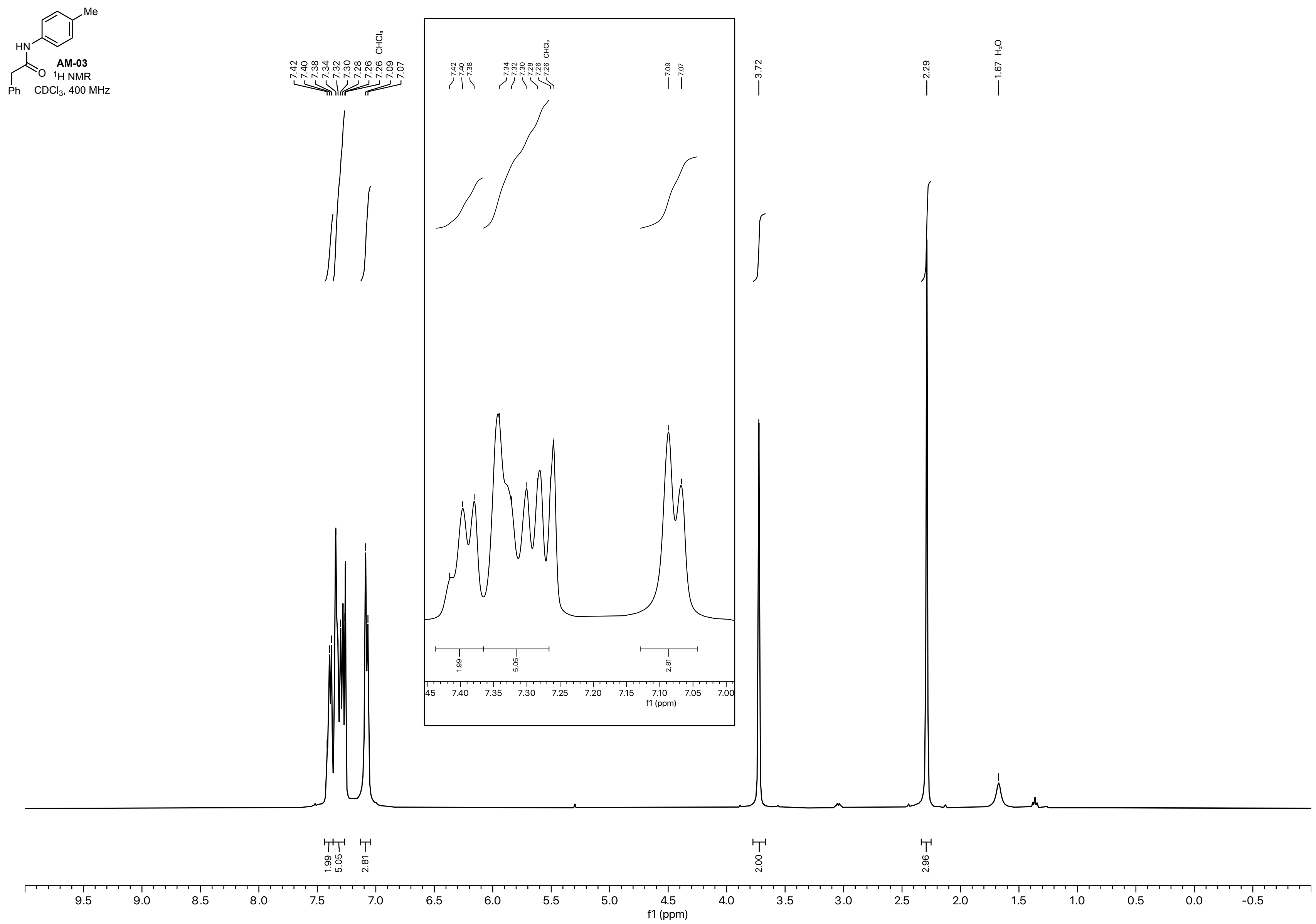

Me
HN
O
Ph
AM-03
^{1}H NMR
$CDCl_3$, 400 MHz
7.42
7.40
7.38
7.34
7.32
7.30
7.28
7.26
7.26
7.09
7.07
$CHCl_3$
3.72
2.29
1.67 H_2O
1.99
5.05
2.81
2.00
2.96
9.5 9.0 8.5 8.0 7.5 7.0 6.5 6.0 5.5 5.0 4.5 4.0 3.5 3.0 2.5 2.0 1.5 1.0 0.5 0.0 -0.5
f1 (ppm)
7.40 7.35 7.30 7.25 7.20 7.15 7.10 7.05 7.00
f1 (ppm)

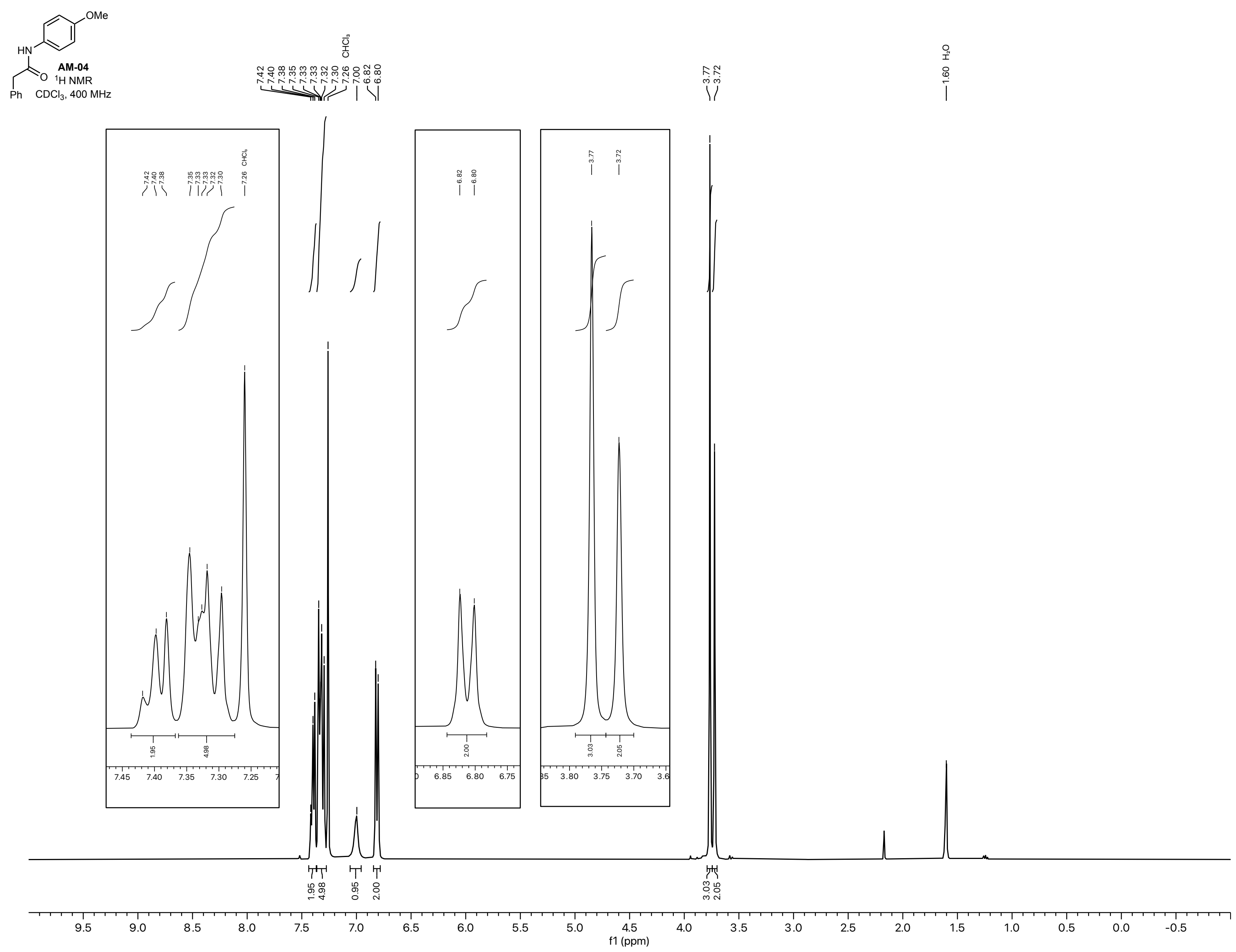

OMe
HN
O
Ph
AM-04
^{1}H NMR
$CDCl_3$, 400 MHz
7.42
7.40
7.38
7.35
7.33
7.33
7.32
7.30
7.26 $CHCl_3$
7.00
6.82
6.80
3.77
3.72
1.60 H_2O
1.95
4.98
0.95
2.00
3.03
2.05
f1 (ppm)

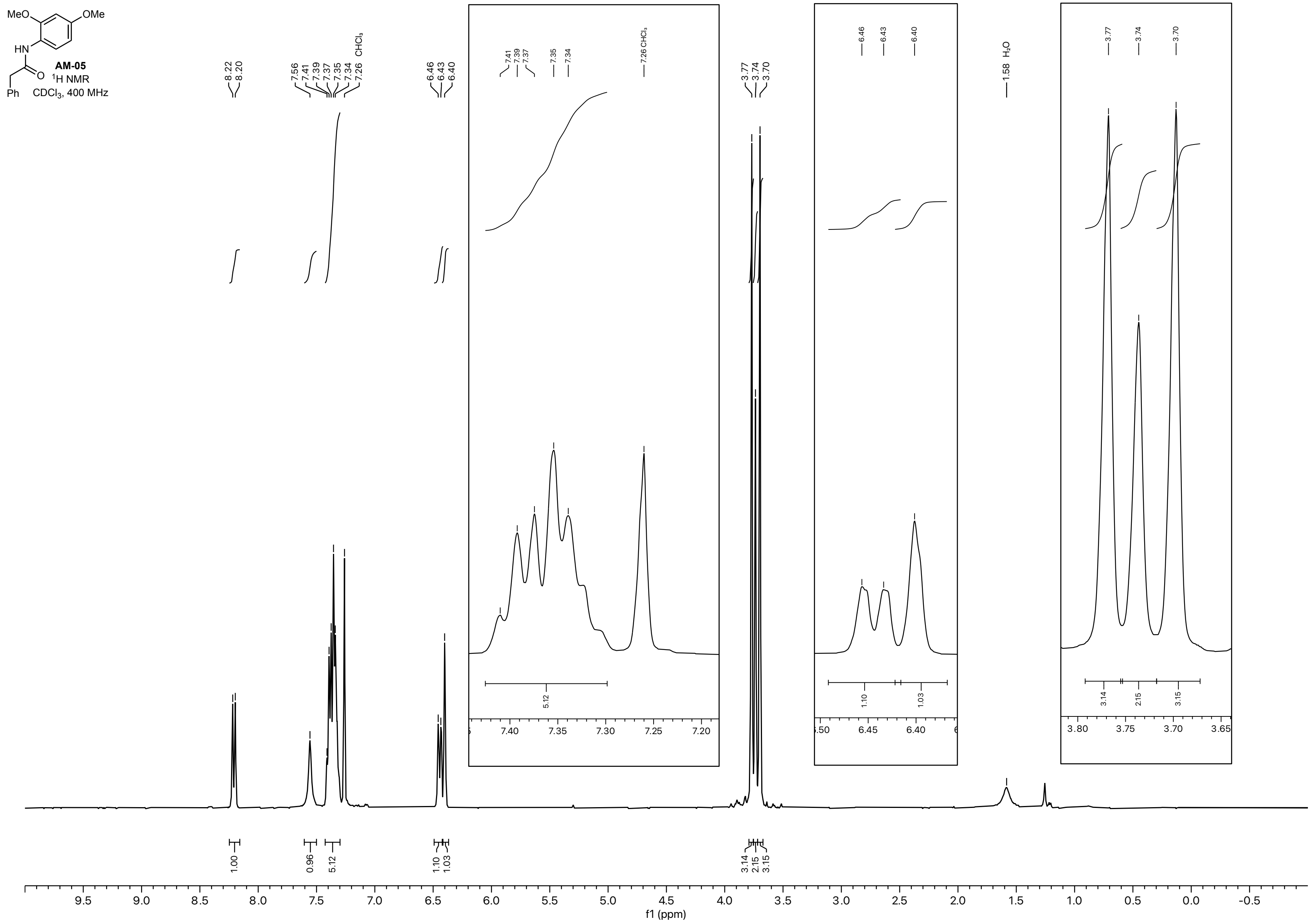
MeO
OMe
HN
O
Ph
AM-05
^{1}H NMR
CDCl$_3$, 400 MHz
8.22
8.20
7.56
7.41
7.39
7.37
7.35
7.34
7.26
CHCl$_3$
6.46
6.43
6.40
3.77
3.74
3.70
1.58 H$_2$O
7.26 CHCl$_3$
1.00
0.96
5.12
1.10
1.03
3.14
2.15
3.15
f1 (ppm)

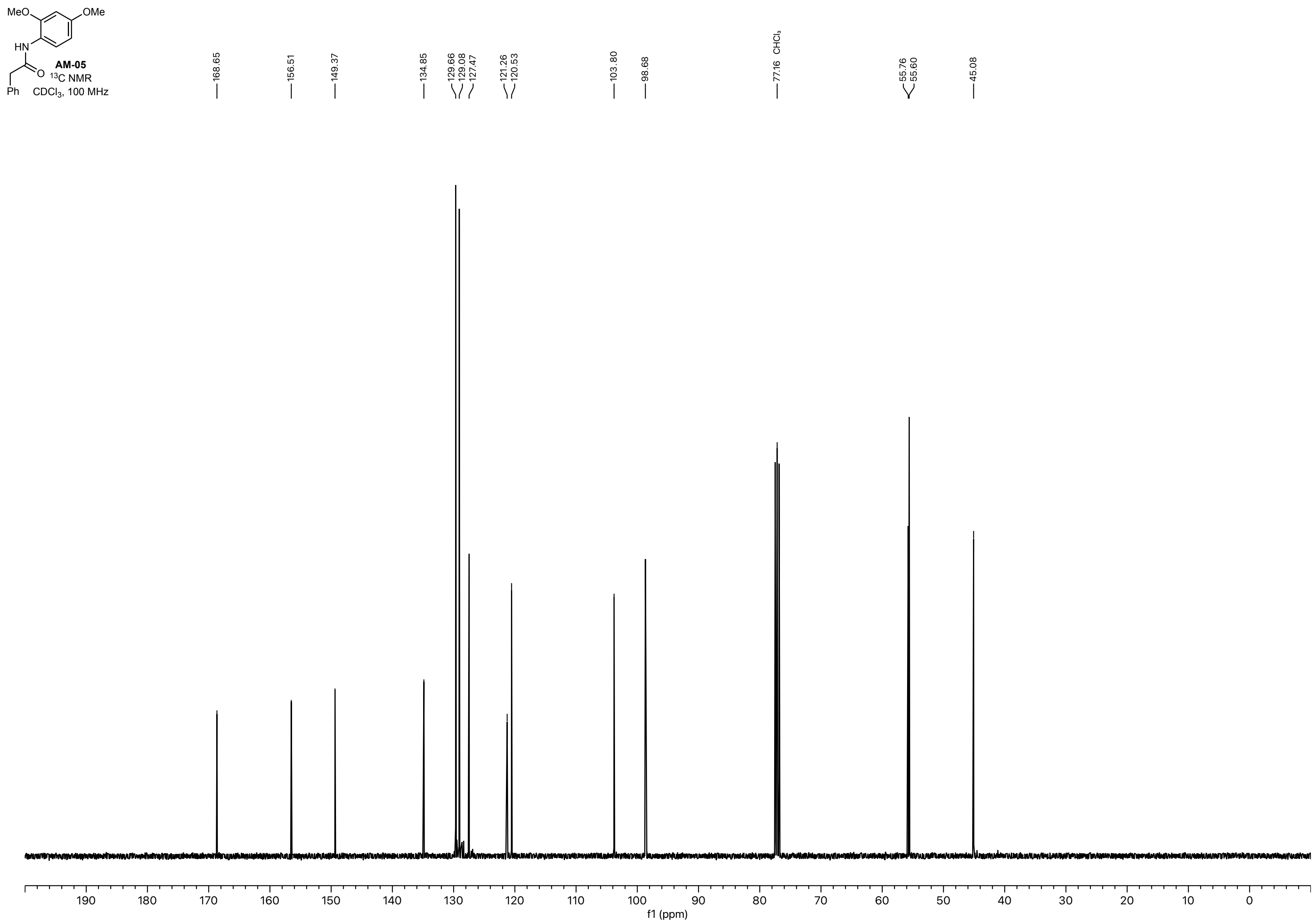

MeO
OMe
HN
O
Ph
AM-05
13C NMR
CDCl3, 100 MHz
168.65
156.51
149.37
134.85
129.66
129.08
127.47
121.26
120.53
103.80
98.68
77.16 CHCl3
55.76
55.60
45.08
190
180
170
160
150
140
130
120
110
100
90
80
70
60
50
40
30
20
10
0
f1 (ppm)

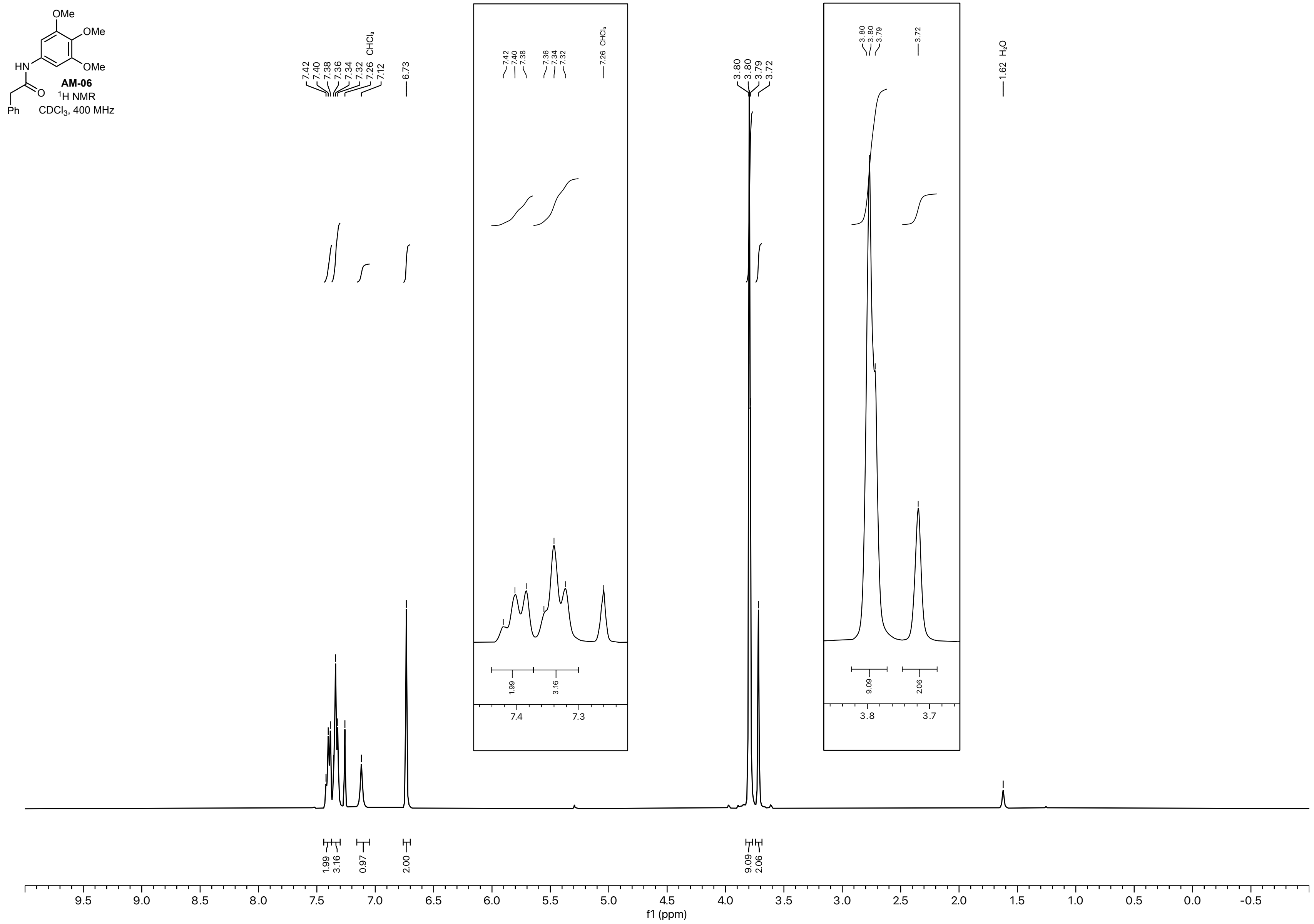

AM-06
^{1}H NMR
$CDCl_3$, 400 MHz
7.42 7.40 7.38 7.36 7.34 7.32 7.26 7.12 CHCl3
6.73
3.80 3.80 3.79 3.72
1.62 H2O
1.99 3.16 0.97 2.00 9.09 2.06
f1 (ppm)

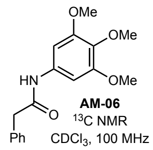

AM-06
^{13}C NMR
$CDCl_3$, 100 MHz

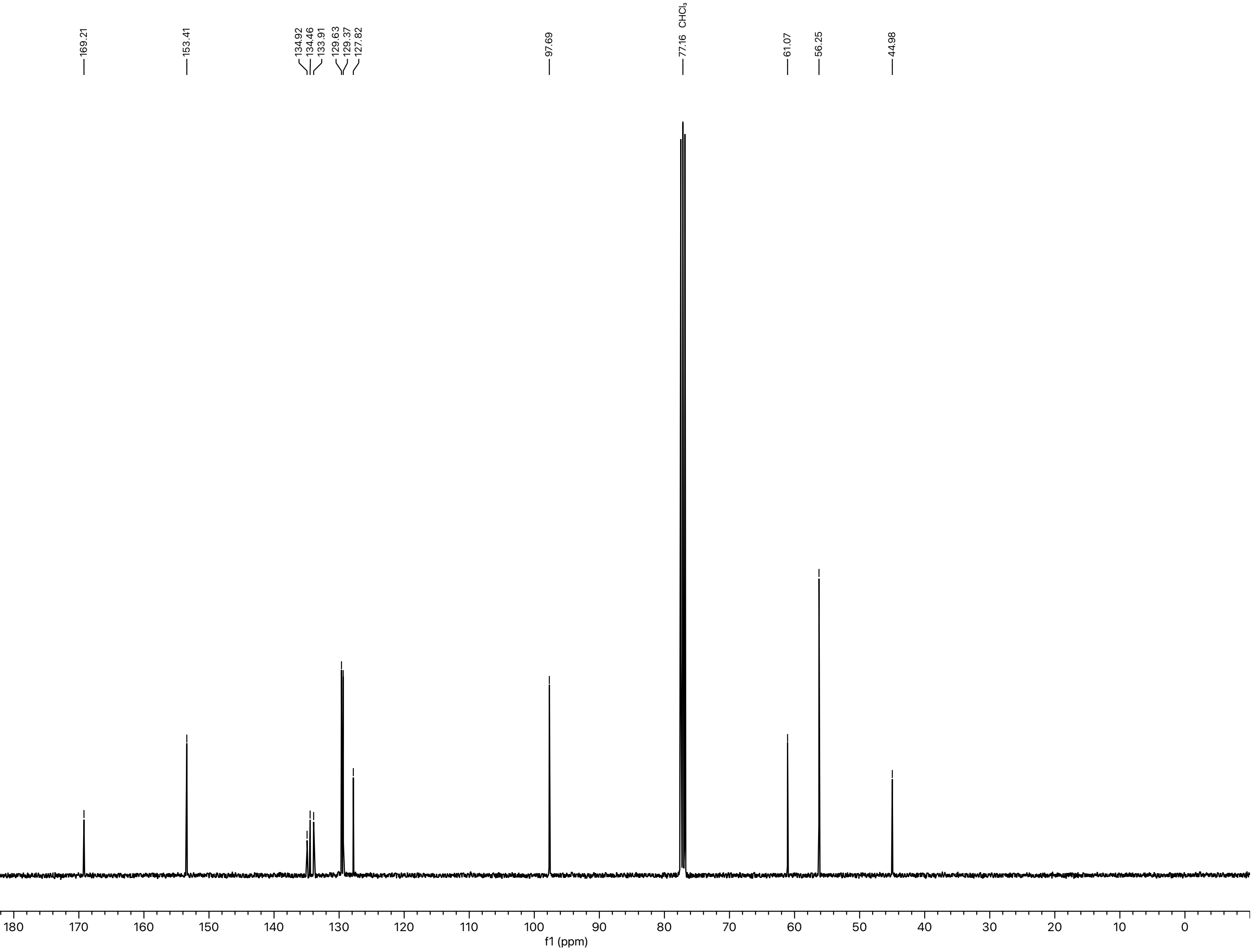

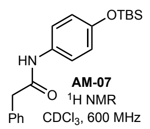

AM-07
^{1}H NMR
$CDCl_3$, 600 MHz

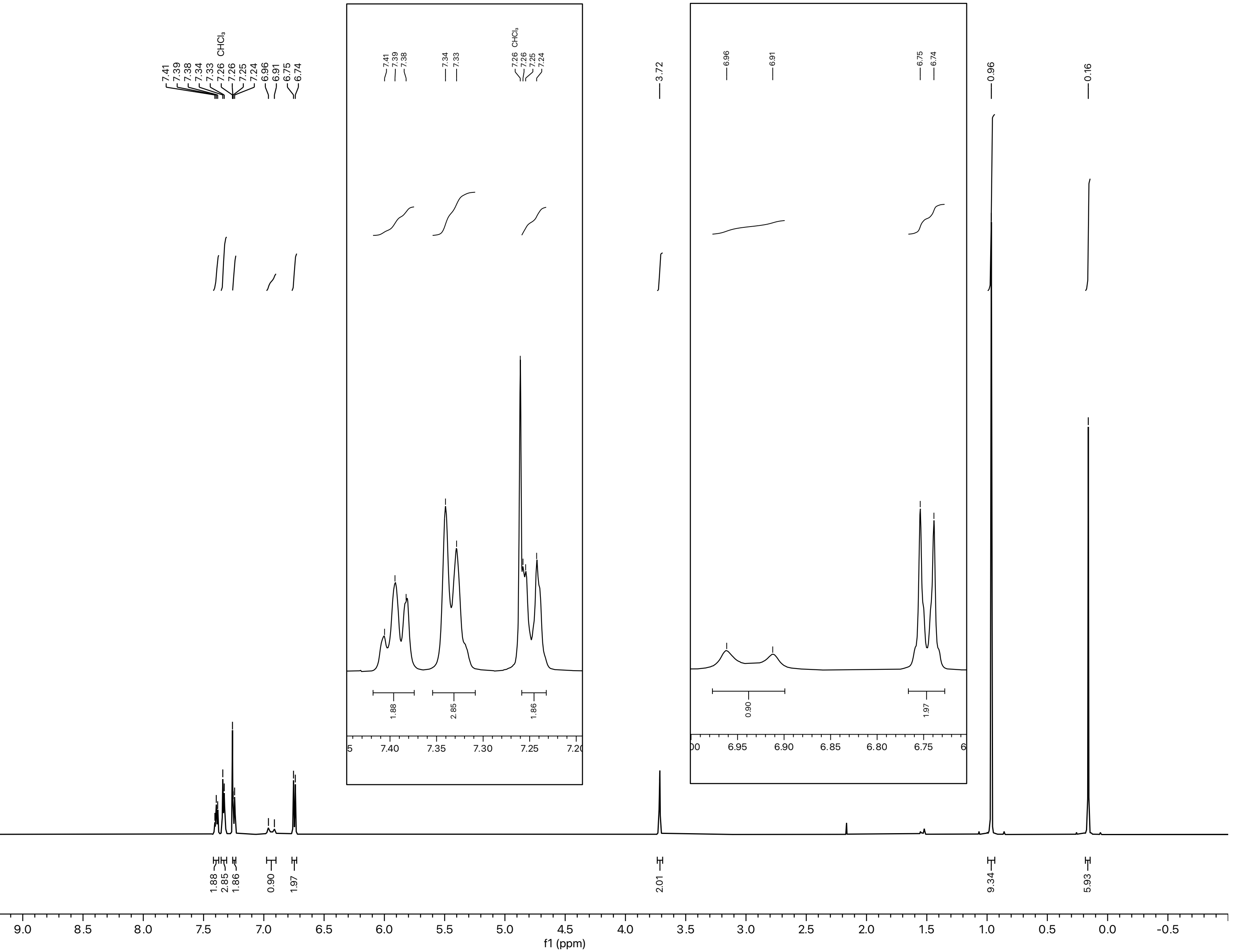

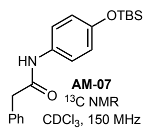
AM-07
^{13}C NMR
$CDCl_3$, 150 MHz

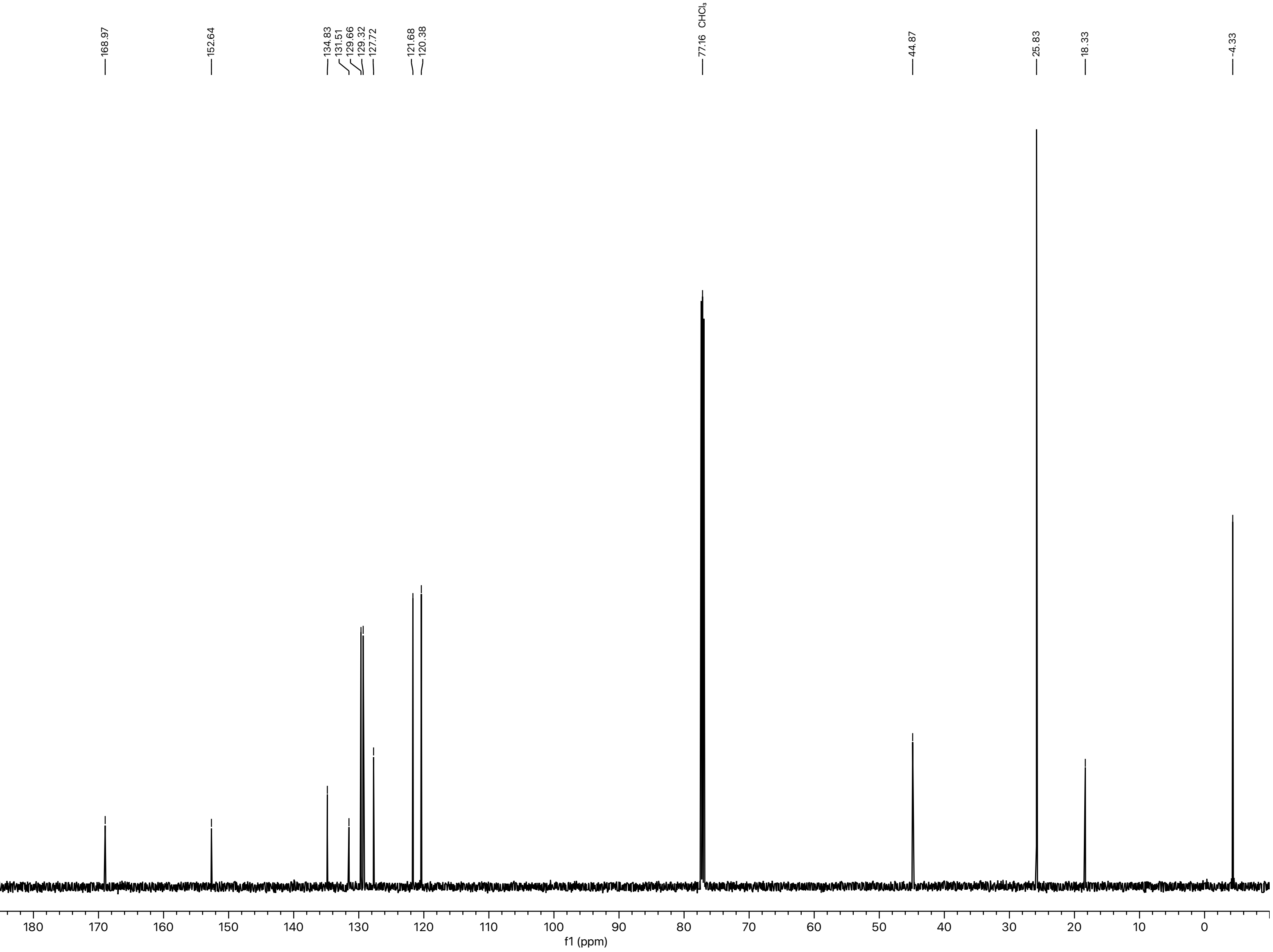

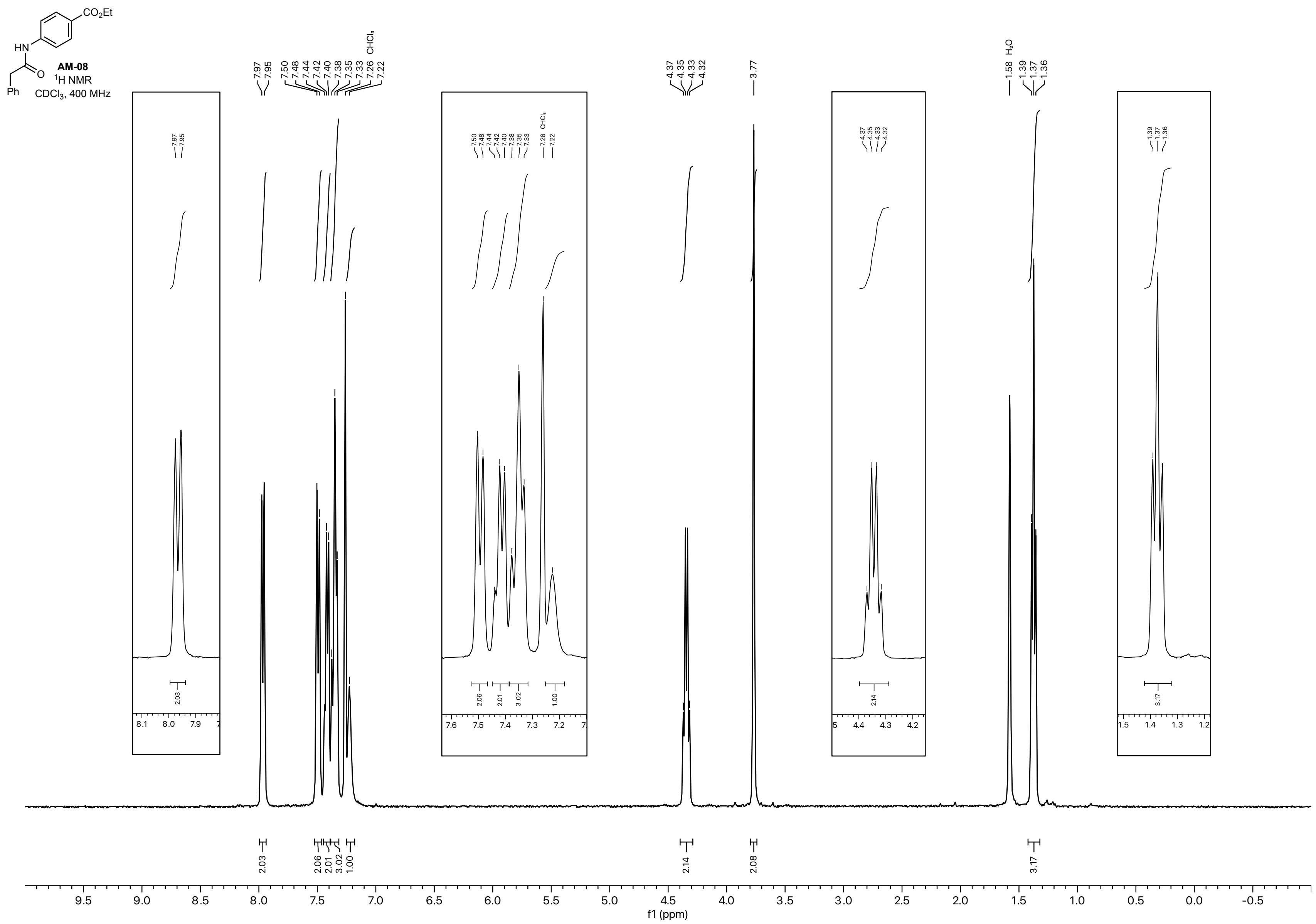
CO2Et
HN
O
Ph
AM-08
1H NMR
CDCl3, 400 MHz
7.97
7.95
7.50
7.48
7.44
7.42
7.40
7.38
7.35
7.33
7.26 CHCl3
7.22
4.37
4.35
4.33
4.32
3.77
1.58 H2O
1.39
1.37
1.36
2.03
2.06
2.01
3.02
1.00
2.14
2.08
3.17
f1 (ppm)

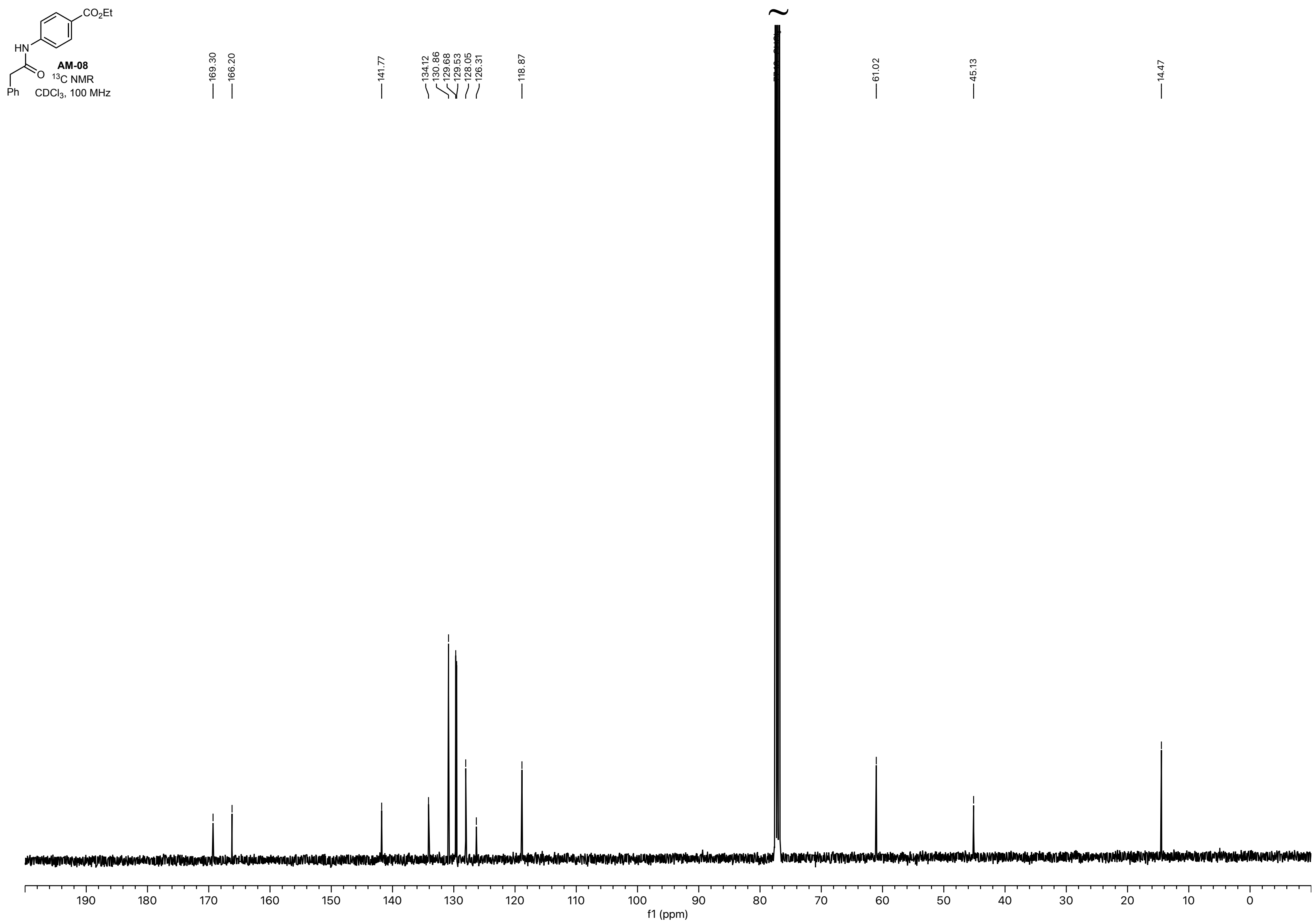
CO2Et
HN
O
Ph
AM-08
13C NMR
CDCl3, 100 MHz
169.30
166.20
141.77
134.12
130.86
129.68
129.53
128.05
126.31
118.87
61.02
45.13
14.47
190
180
170
160
150
140
130
120
110
100
90
80
70
60
50
40
30
20
10
0
f1 (ppm)

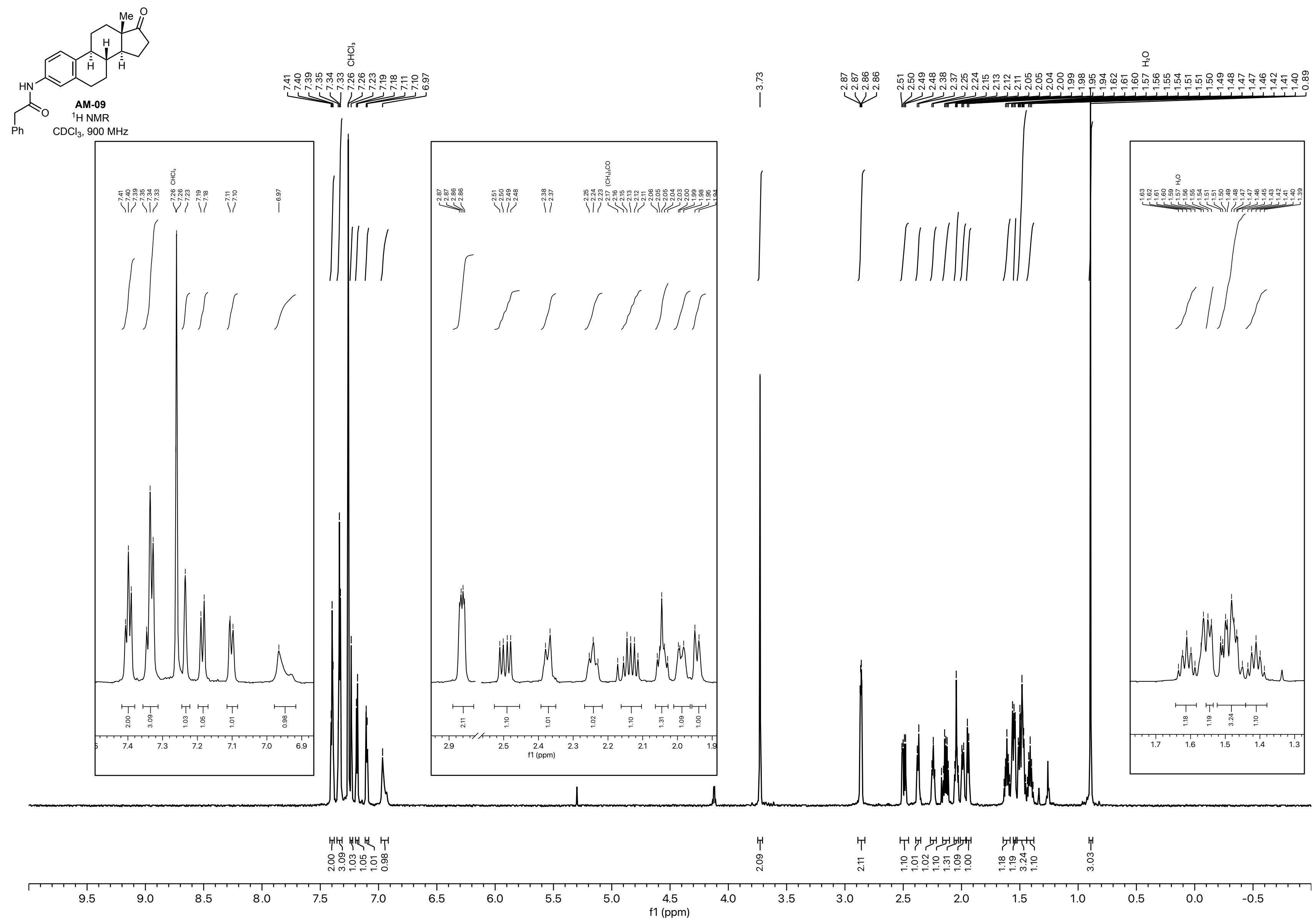
Me
O
H
H
H
HN
O
Ph
AM-09
1H NMR
CDCl3, 900 MHz
CHCl3
H2O
(CH3)2CO
f1 (ppm)

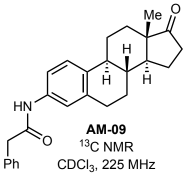

AM-09
^{13}C NMR
$CDCl_3$, 225 MHz

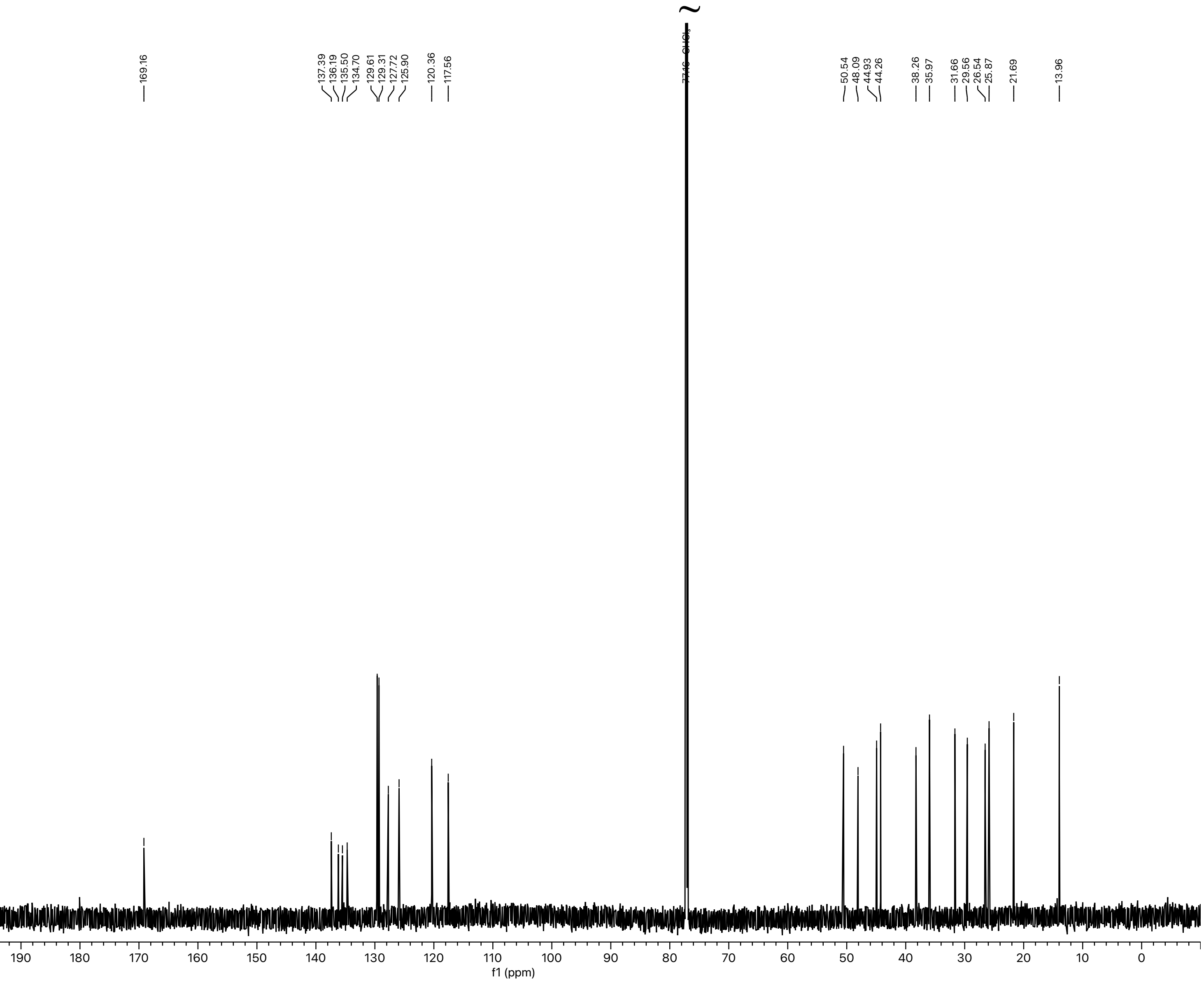

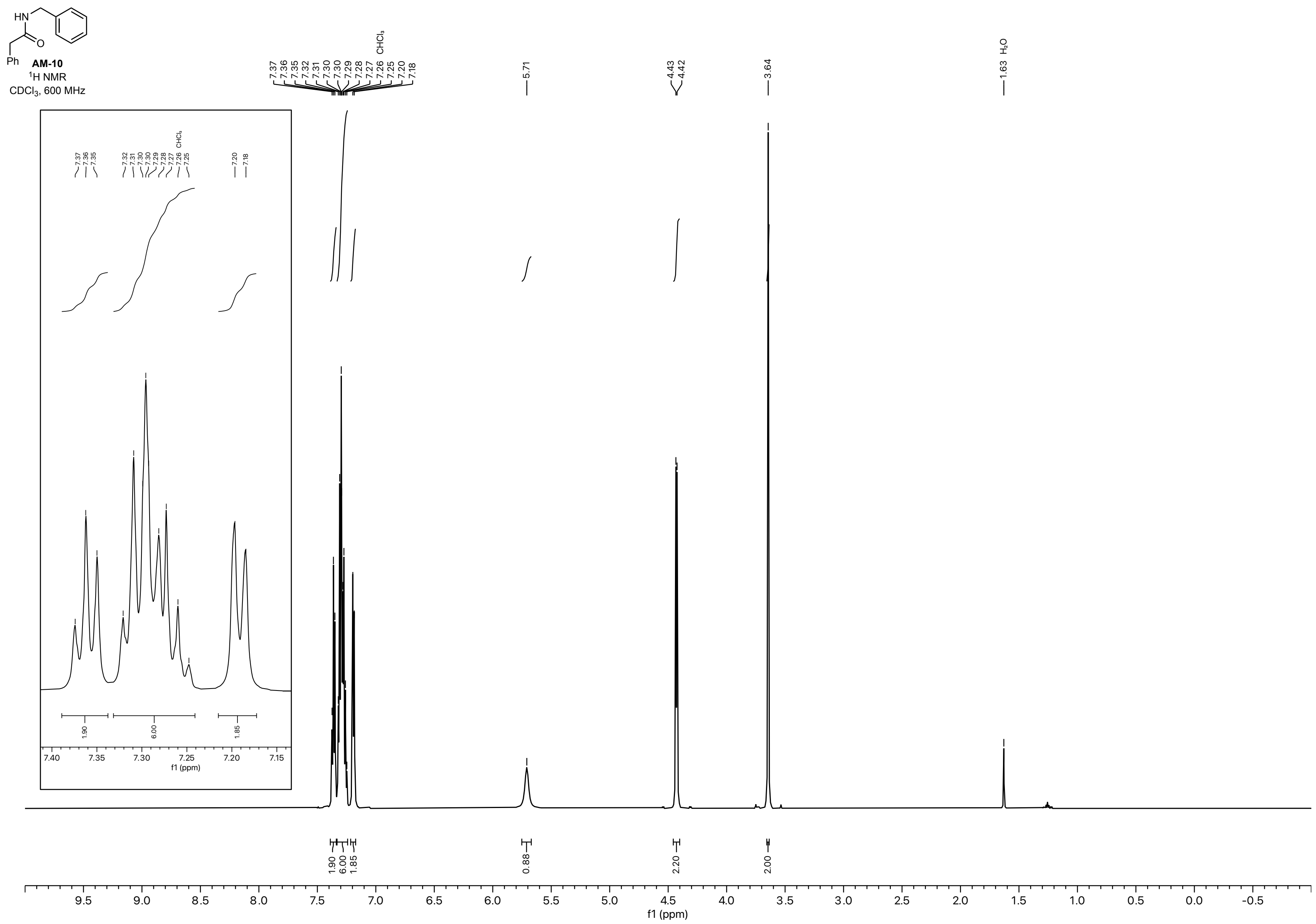

HN
O
Ph
AM-10
1H NMR
CDCl3, 600 MHz
7.37
7.36
7.35
7.32
7.31
7.30
7.30
7.29
7.28
7.27
7.26 CHCl3
7.25
7.20
7.18
5.71
4.43
4.42
3.64
1.63 H2O
1.90
6.00
1.85
0.88
2.20
2.00
f1 (ppm)

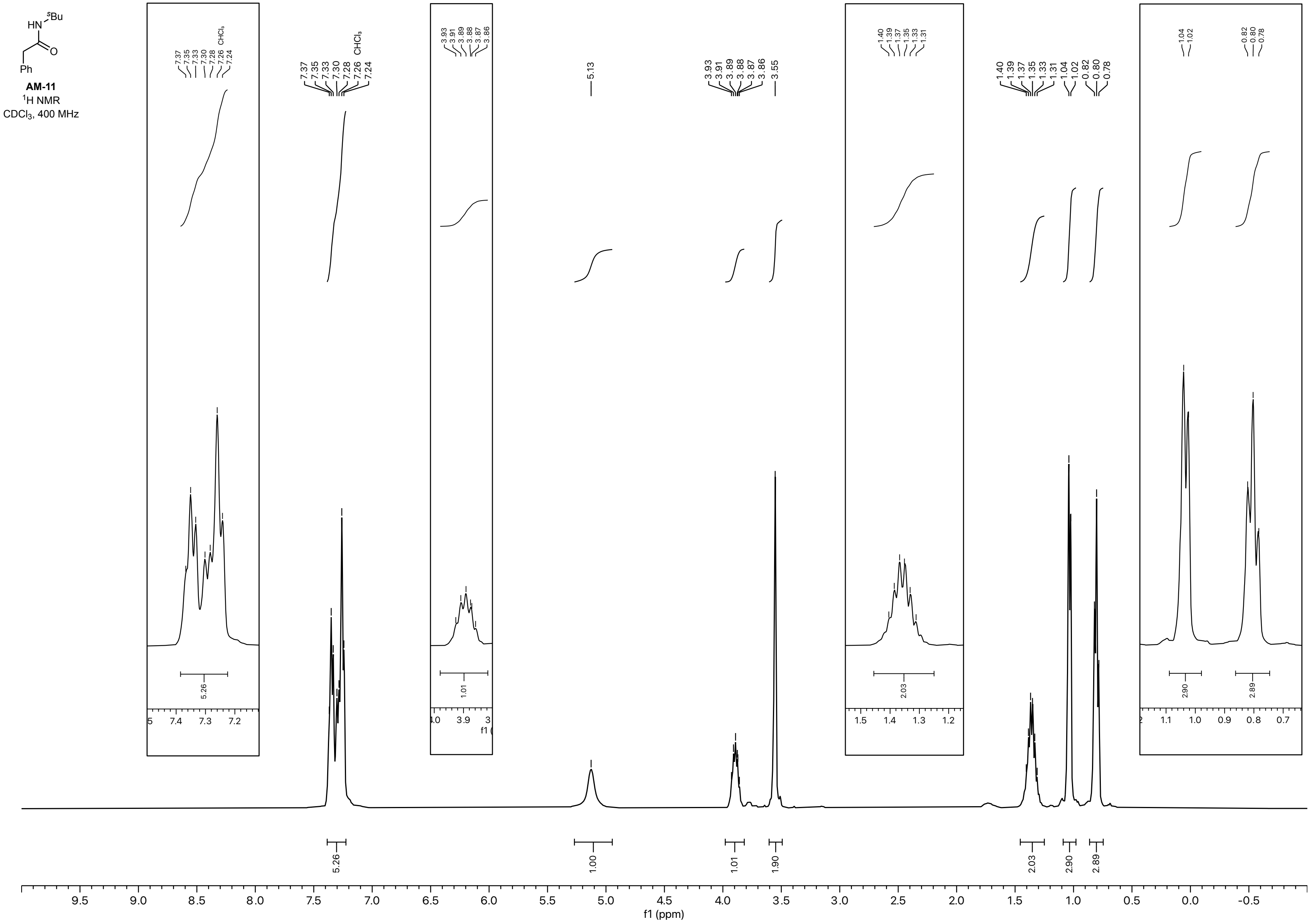
HN ^{s}Bu
O
Ph
AM-11
^{1}H NMR
CDCl$_3$, 400 MHz
7.37
7.35
7.33
7.30
7.28
7.26 CHCl$_3$
7.24
5.13
3.93
3.91
3.89
3.88
3.87
3.86
3.55
1.40
1.39
1.37
1.35
1.33
1.31
1.04
1.02
0.82
0.80
0.78
5.26
1.00
1.01
1.90
2.03
2.90
2.89
9.5 9.0 8.5 8.0 7.5 7.0 6.5 6.0 5.5 5.0 4.5 4.0 3.5 3.0 2.5 2.0 1.5 1.0 0.5 0.0 -0.5
f1 (ppm)

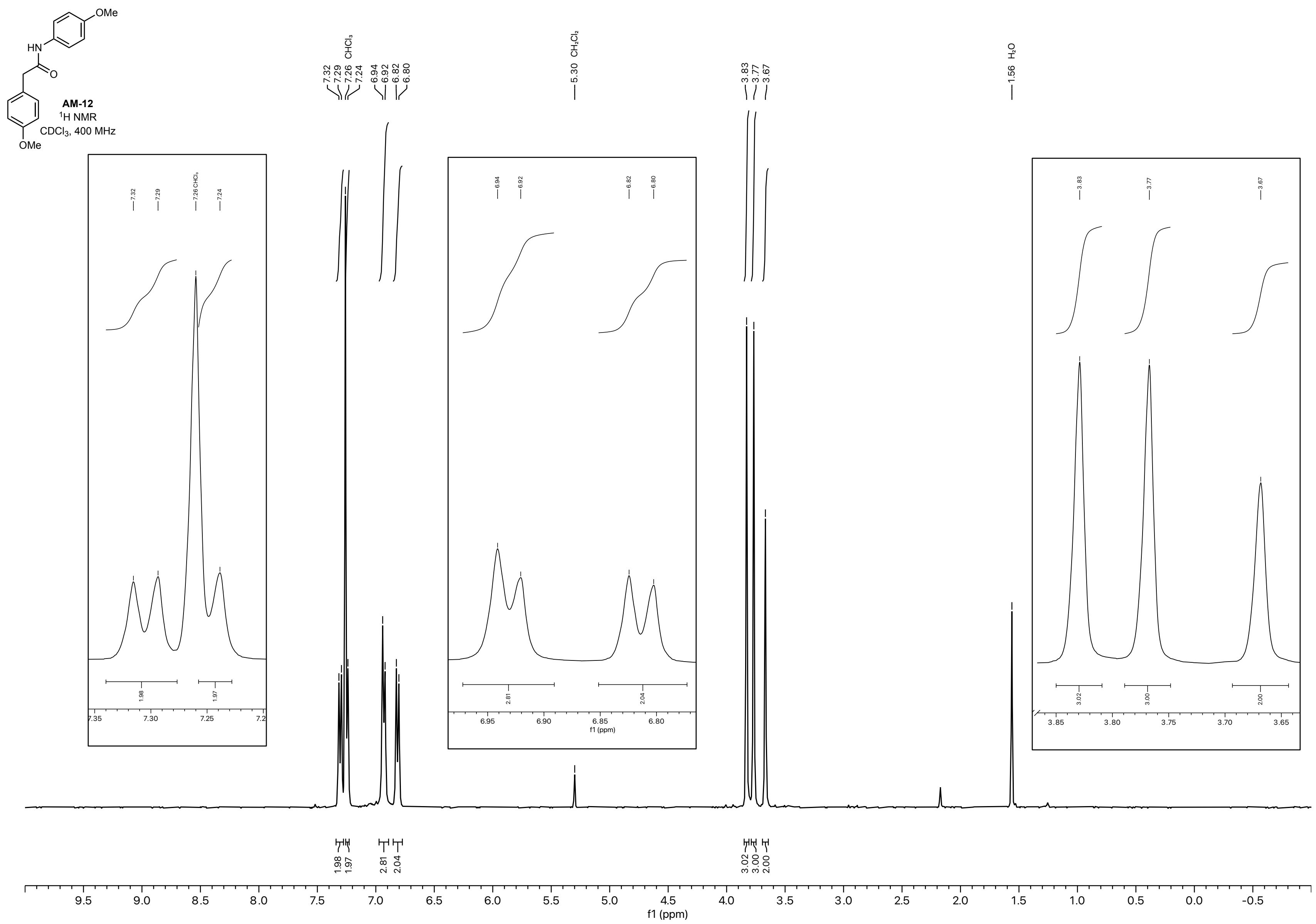
AM-12
1H NMR
CDCl3, 400 MHz
7.32
7.29
7.26 CHCl3
7.24
6.94
6.92
6.82
6.80
5.30 CH2Cl2
3.83
3.77
3.67
1.56 H2O
1.98
1.97
2.81
2.04
3.02
3.00
2.00
f1 (ppm)

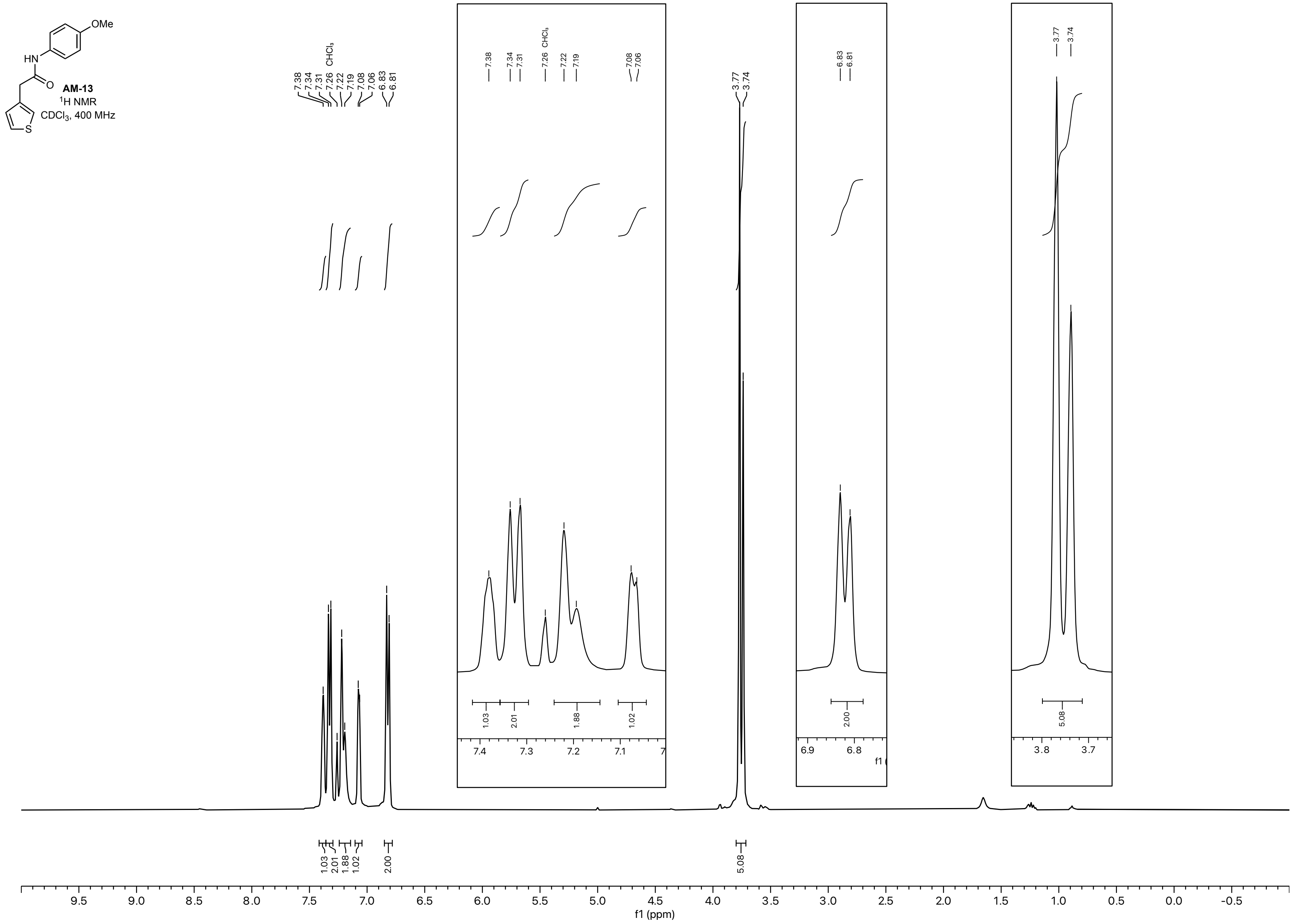
OMe
HN
O
S
AM-13
^{1}H NMR
$CDCl_3$, 400 MHz
7.38 7.34 7.31 7.26 $CHCl_3$ 7.22 7.19 7.08 7.06 6.83 6.81
3.77 3.74
1.03 2.01 1.88 1.02 2.00 5.08
f1 (ppm)

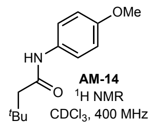

AM-14
^1H NMR
$CDCl_3$, 400 MHz

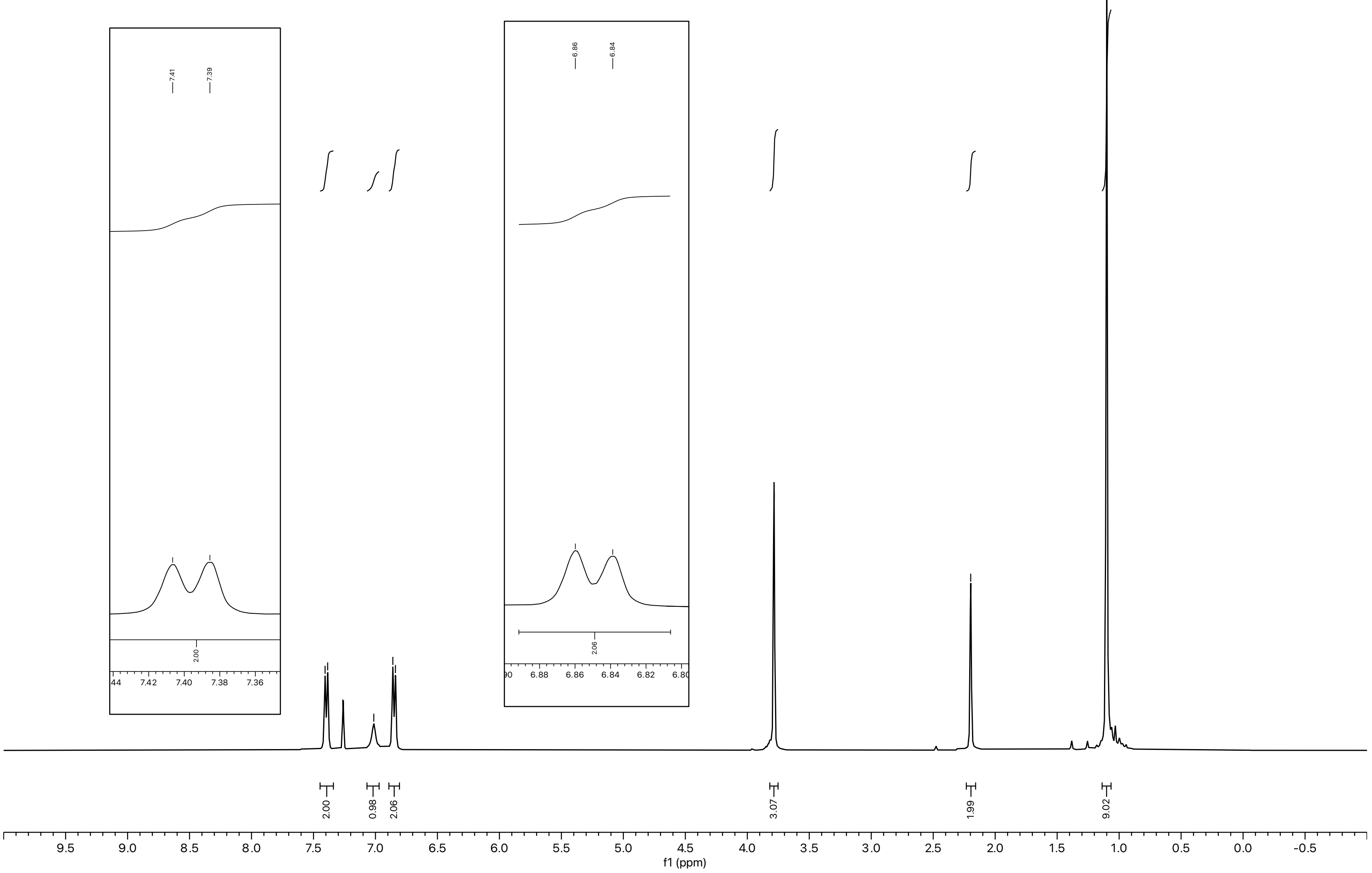

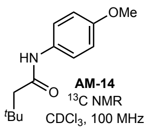

AM-14
^{13}C NMR
$CDCl_3$, 100 MHz

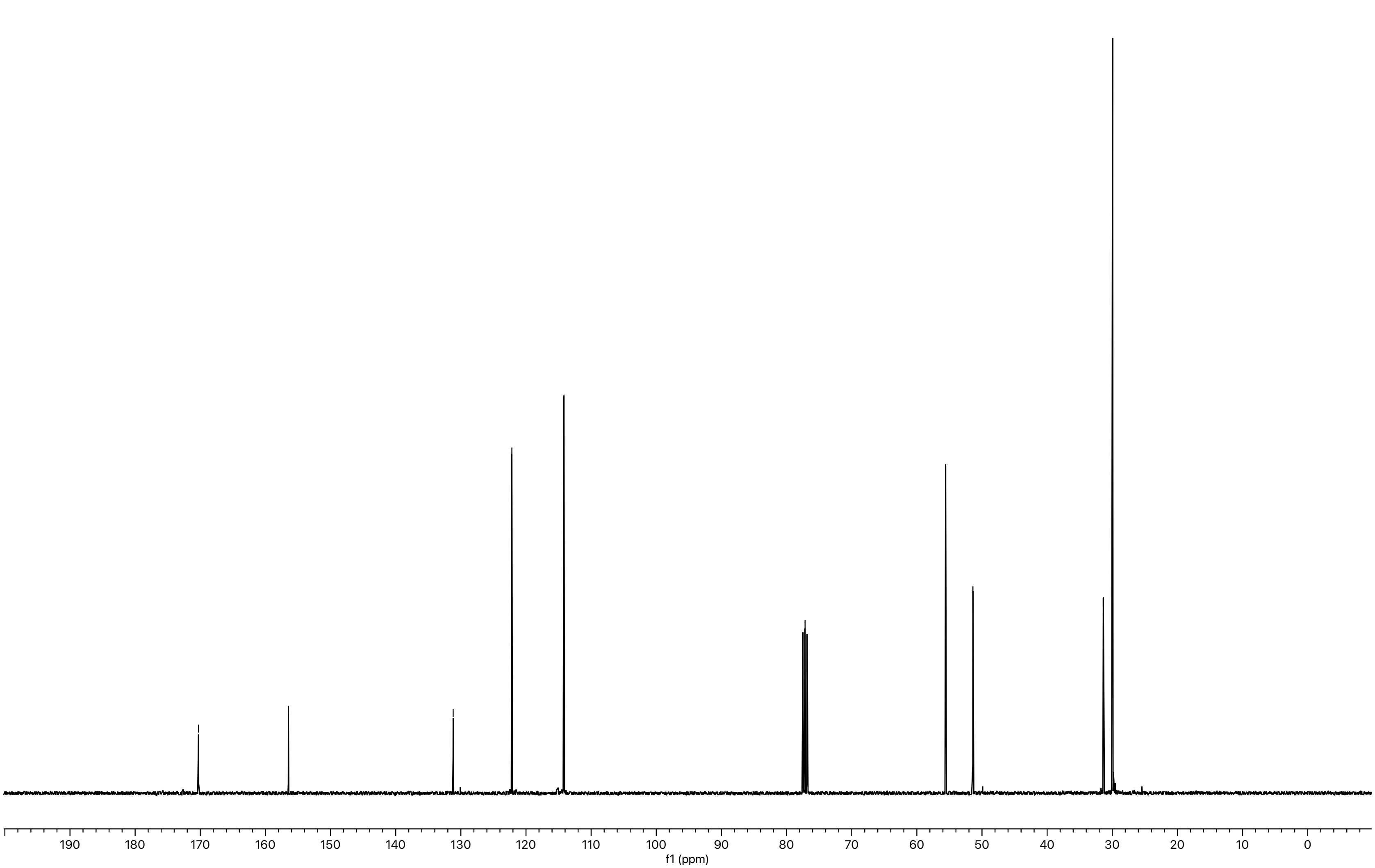

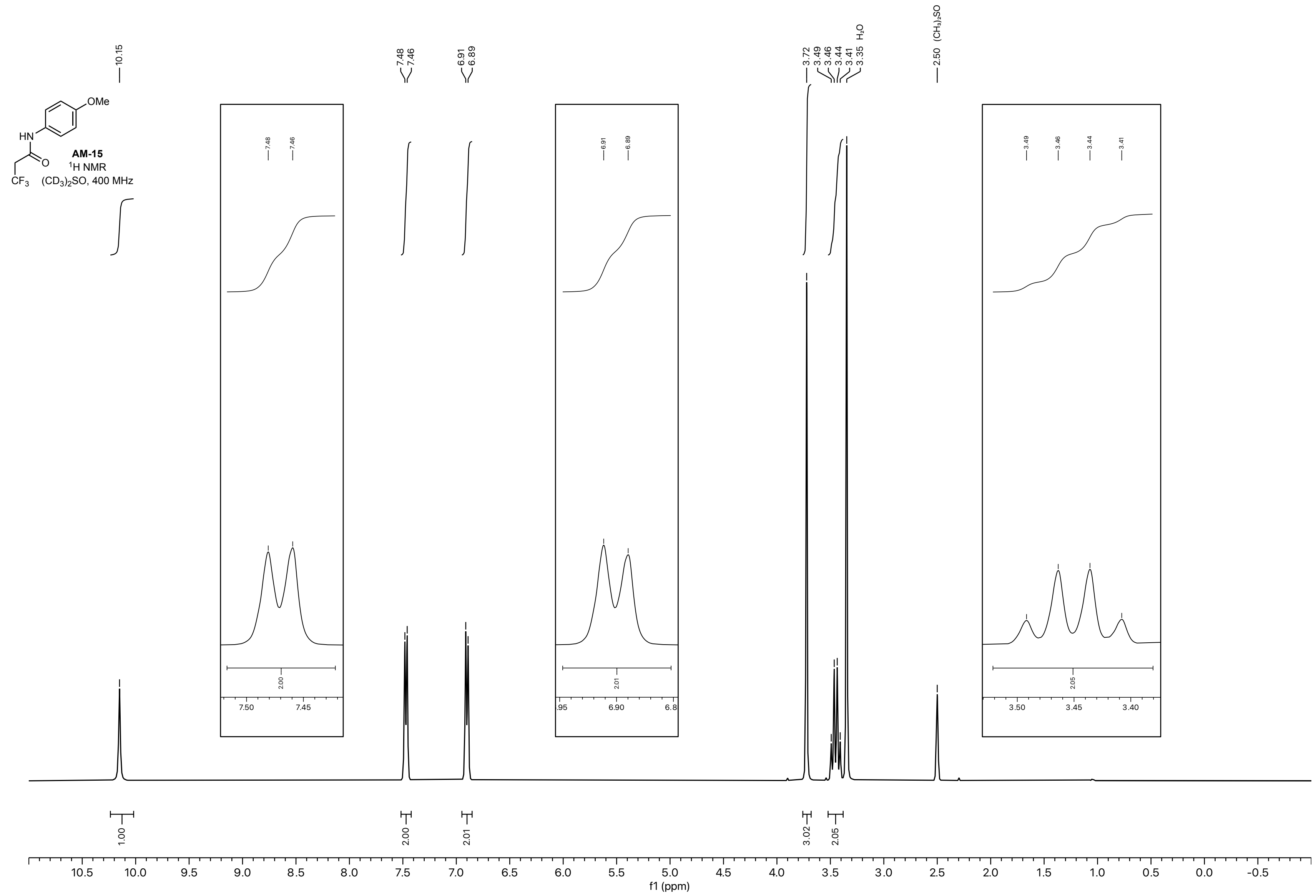
OMe
HN
O
CF3
AM-15
1H NMR
(CD3)2SO, 400 MHz
10.15
7.48
7.46
6.91
6.89
3.72
3.49
3.46
3.44
3.41
3.35 H2O
2.50 (CH3)2SO
2.00
2.01
2.05
1.00
3.02
f1 (ppm)

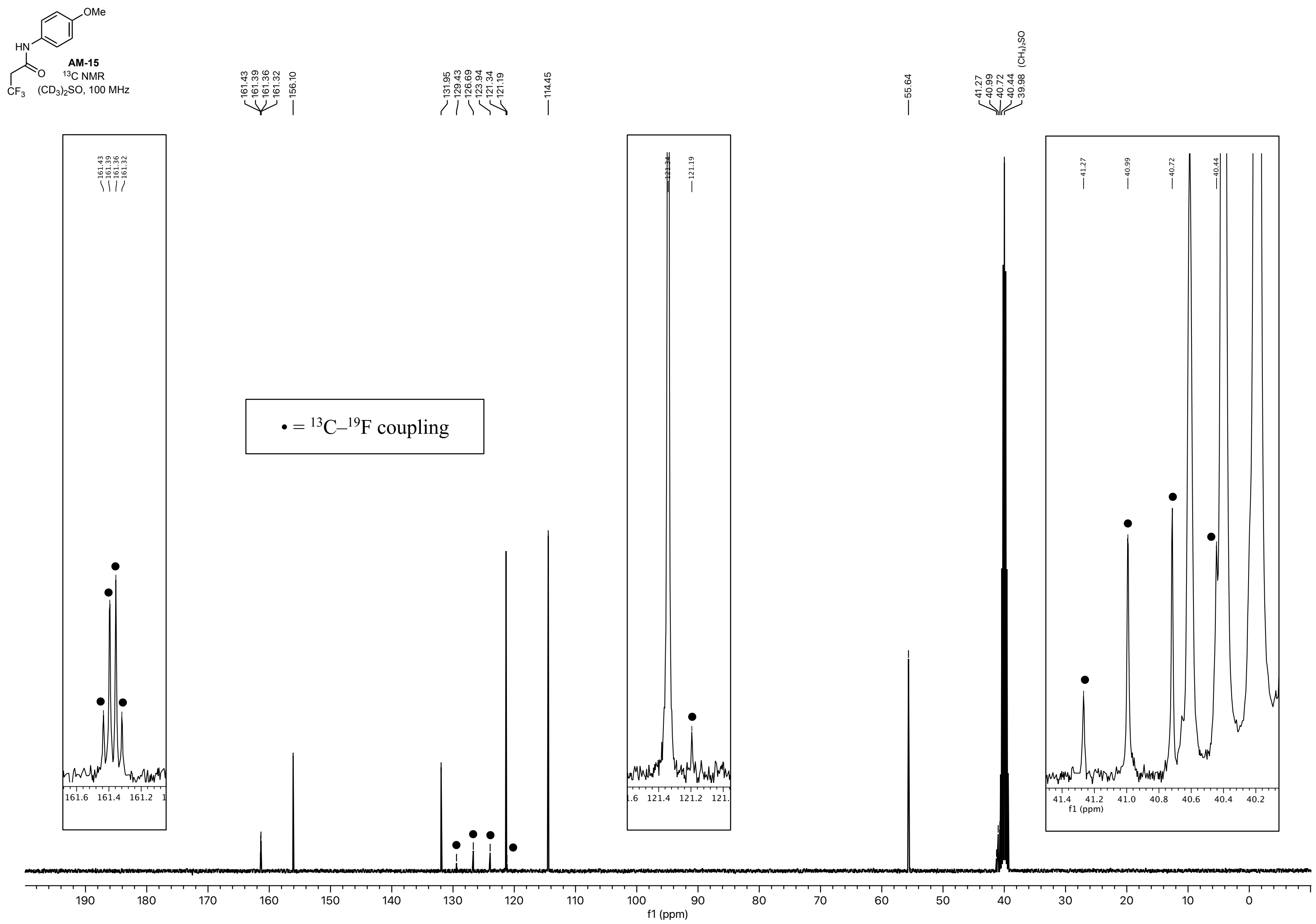

OMe
HN
O
CF3
AM-15
13C NMR
(CD3)2SO, 100 MHz
161.43
161.39
161.36
161.32
156.10
131.95
129.43
126.69
123.94
121.34
121.19
114.45
55.64
41.27
40.99
40.72
40.44
39.98
(CH3)2SO
• = 13C–19F coupling
f1 (ppm)

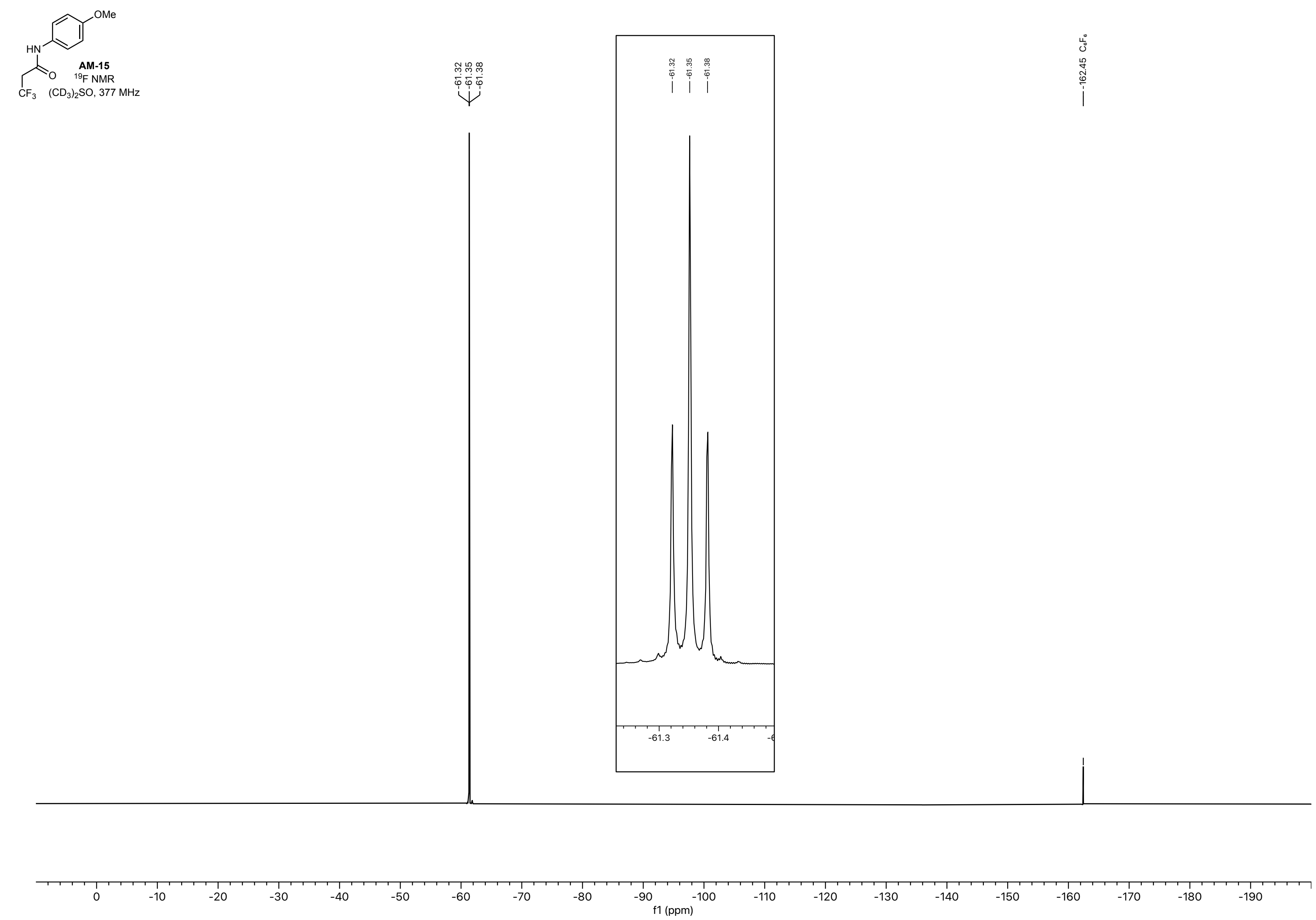

OMe
HN
O
CF3
AM-15
19F NMR
(CD3)2SO, 377 MHz
-61.32
-61.35
-61.38
-162.45 C6F6
-61.3
-61.4
0
-10
-20
-30
-40
-50
-60
-70
-80
-90
-100
-110
-120
-130
-140
-150
-160
-170
-180
-190
f1 (ppm)

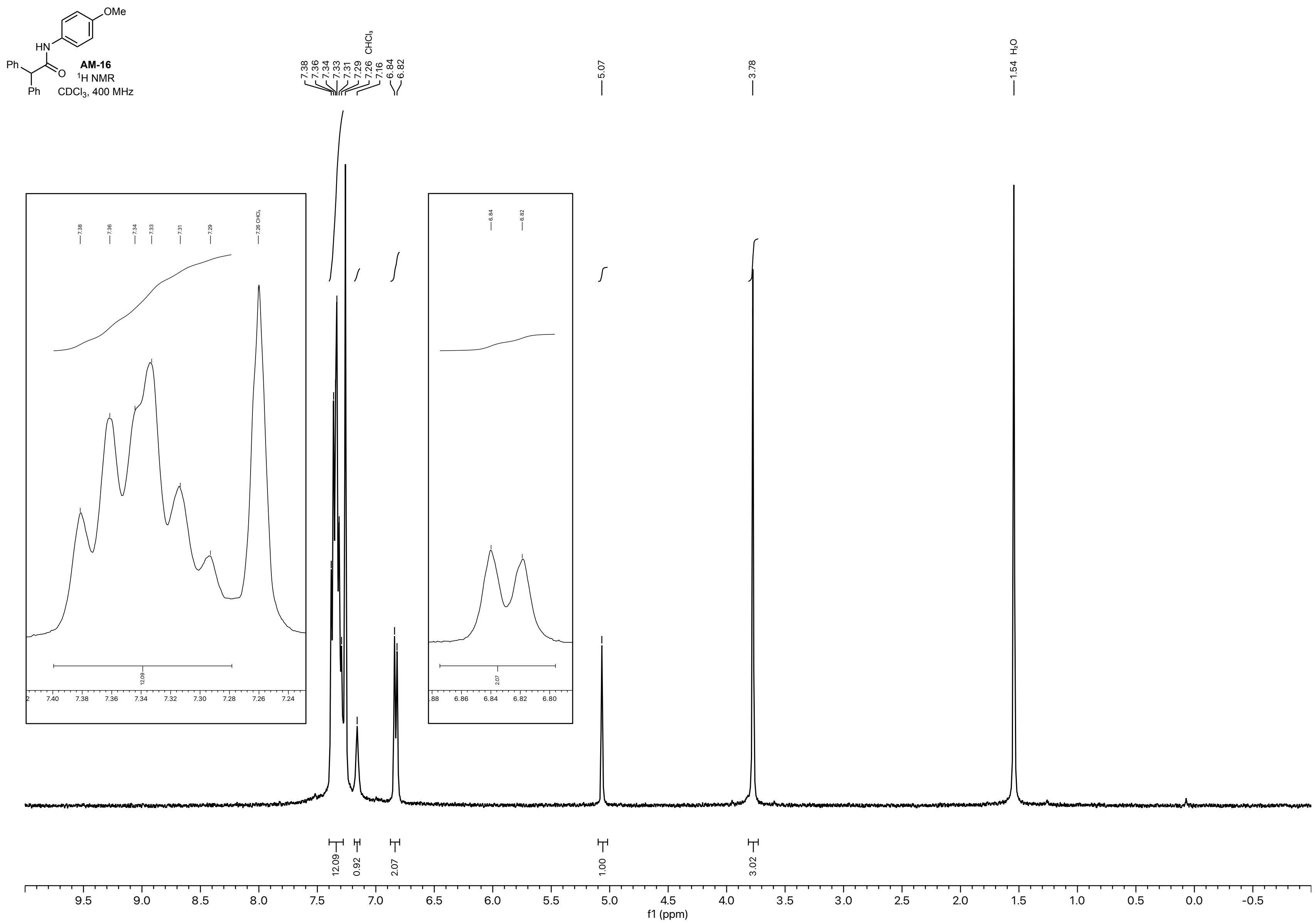
OMe
HN
Ph
O
Ph
AM-16
^{1}H NMR
$CDCl_3$, 400 MHz
7.38
7.36
7.34
7.33
7.31
7.29
7.26 $CHCl_3$
7.16
6.84
6.82
5.07
3.78
1.54 H_2O
12.09
0.92
2.07
1.00
3.02
f1 (ppm)

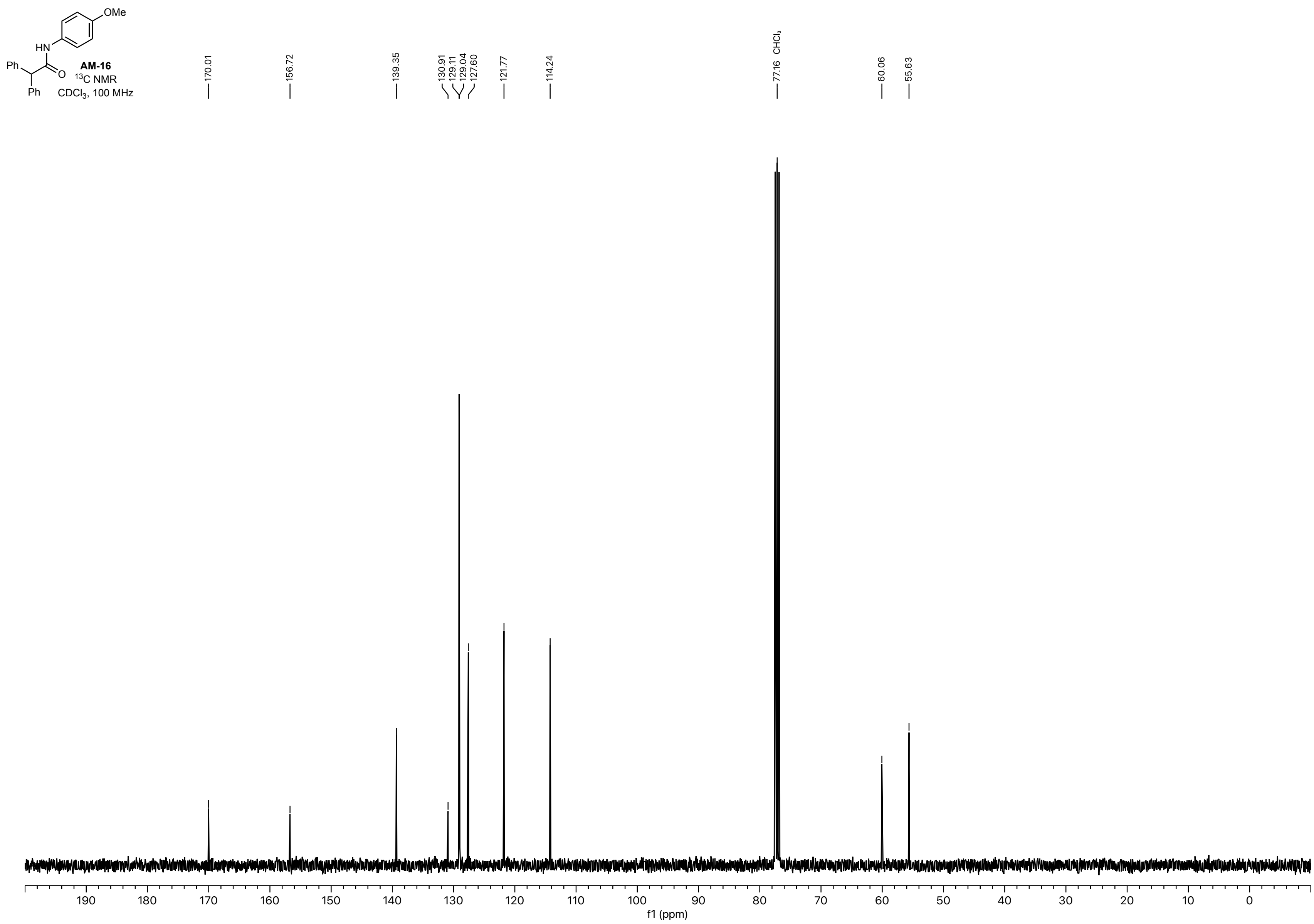

OMe
HN
Ph
O
Ph
AM-16
13C NMR
CDCl3, 100 MHz
170.01
156.72
139.35
130.91
129.11
129.04
127.60
121.77
114.24
77.16 CHCl3
60.06
55.63
190
180
170
160
150
140
130
120
110
100
90
80
70
60
50
40
30
20
10
0
f1 (ppm)

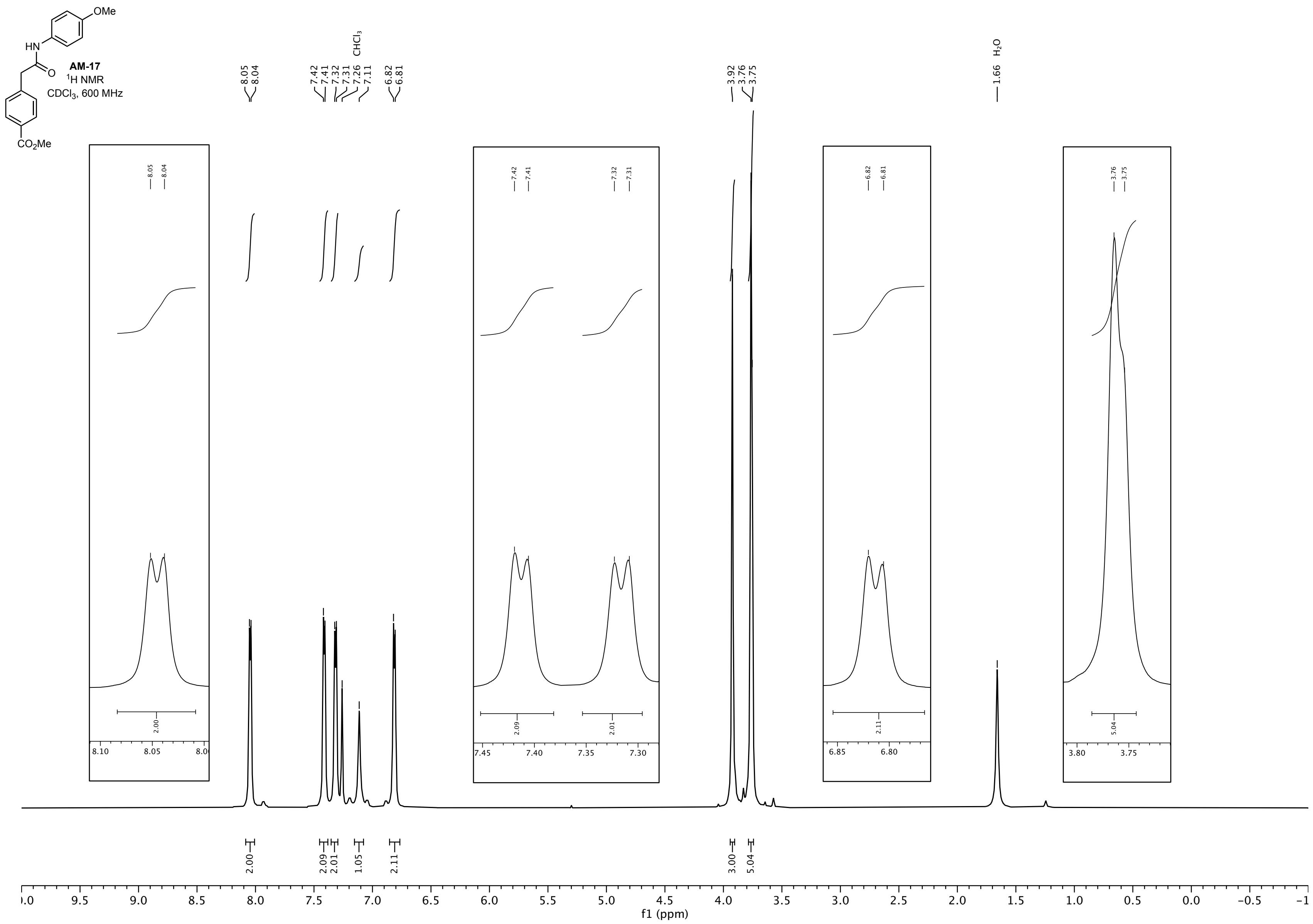
OMe
HN
O
CO2Me
AM-17
1H NMR
CDCl3, 600 MHz
8.05
8.04
7.42
7.41
7.32
7.31
7.26
7.11
CHCl3
6.82
6.81
3.92
3.76
3.75
1.66
H2O
2.00
2.09
2.01
1.05
2.11
3.00
5.04
f1 (ppm)

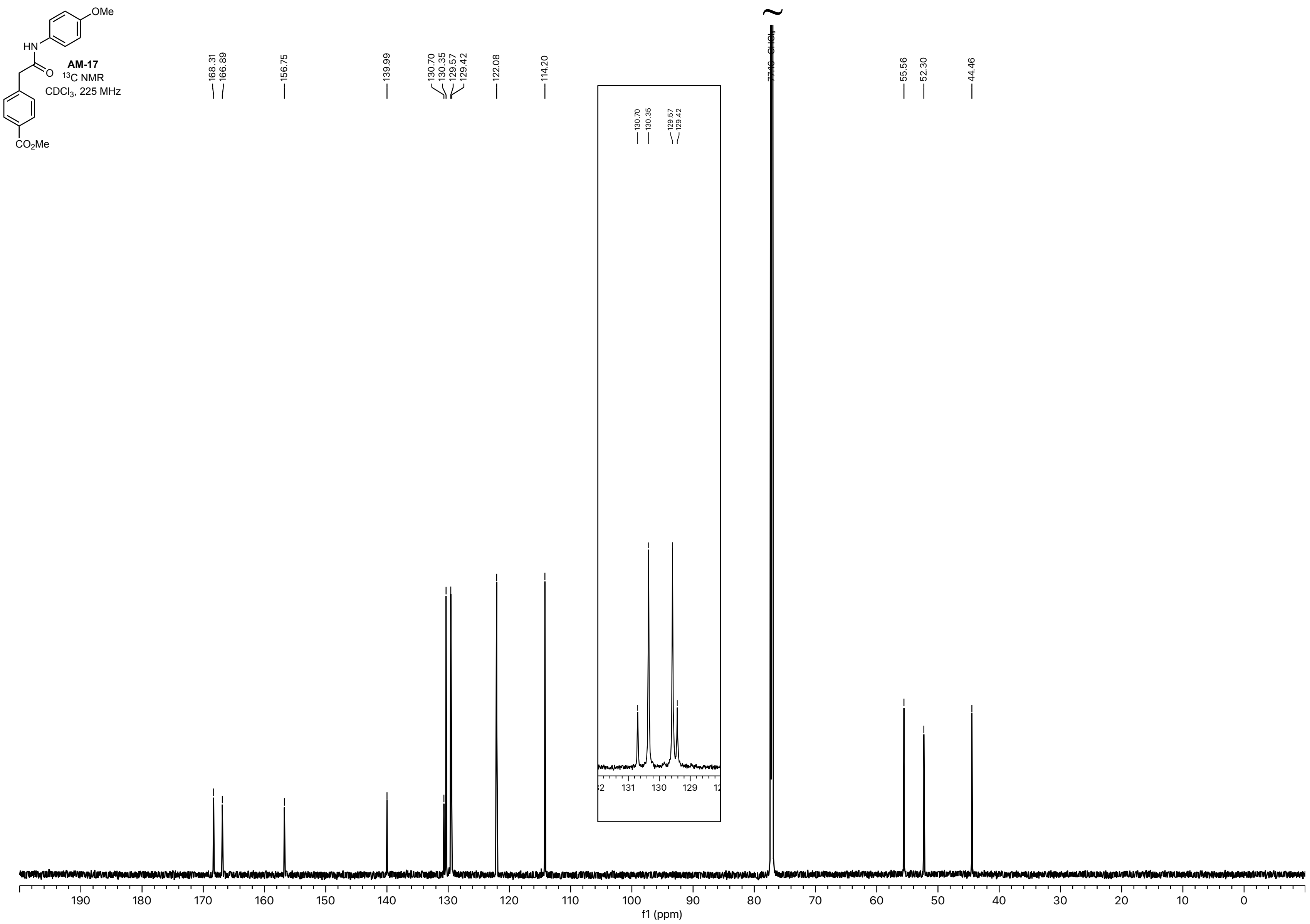

OMe
HN
O
CO$_2$Me
AM-17
^{13}C NMR
CDCl$_3$, 225 MHz
168.31
166.89
156.75
139.99
130.70
130.35
129.57
129.42
122.08
114.20
CHCl$_3$
55.56
52.30
44.46
131
130
129
190 180 170 160 150 140 130 120 110 100 90 80 70 60 50 40 30 20 10 0
f1 (ppm)

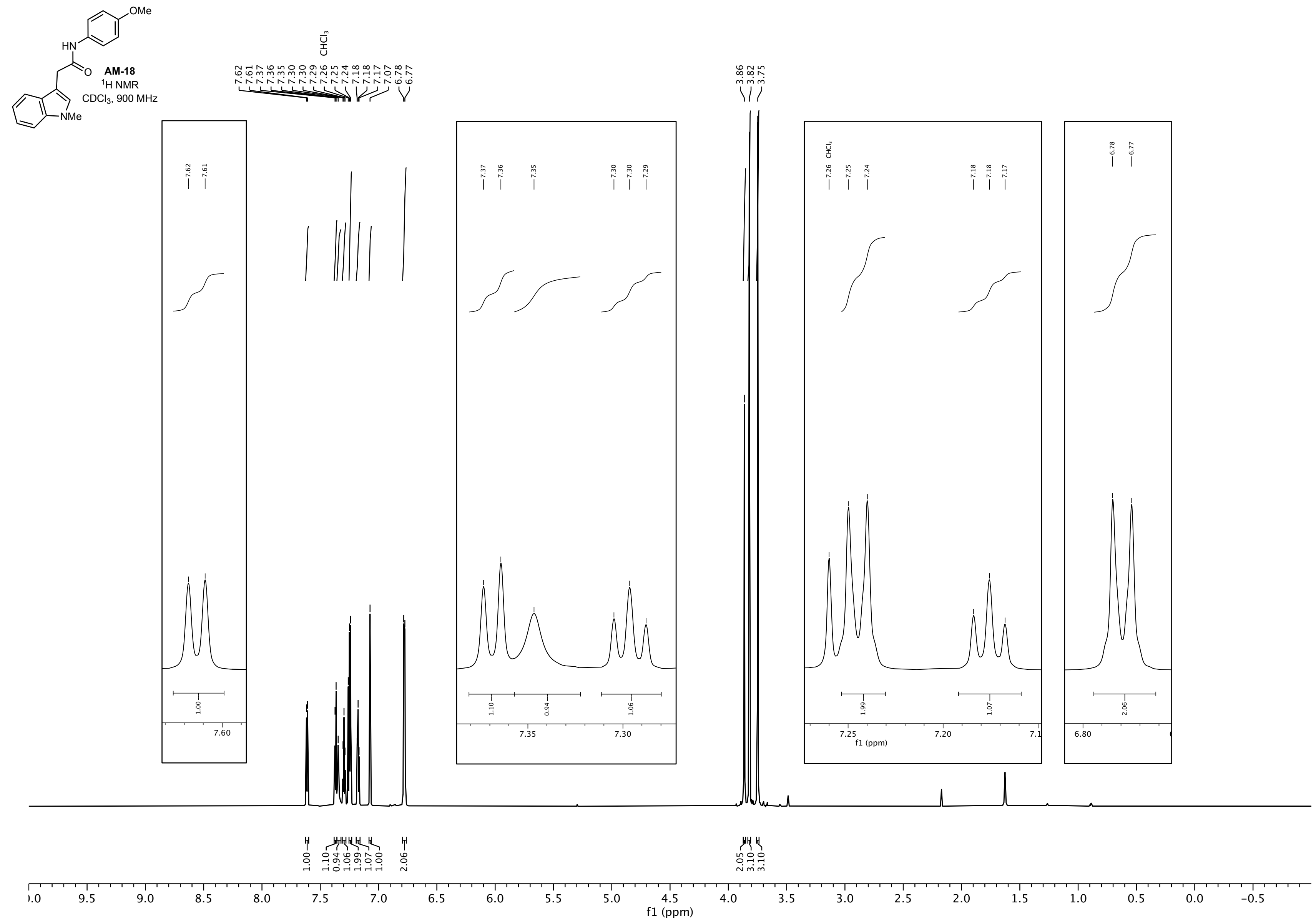

OMe
HN
O
NMe
AM-18
^{1}H NMR
$CDCl_3$, 900 MHz
$CHCl_3$
7.62
7.61
7.37
7.36
7.35
7.30
7.30
7.29
7.26
7.25
7.24
7.18
7.18
7.17
7.07
6.78
6.77
3.86
3.82
3.75
1.00
1.10
0.94
1.06
1.99
1.07
1.00
2.06
2.05
3.10
3.10
f1 (ppm)

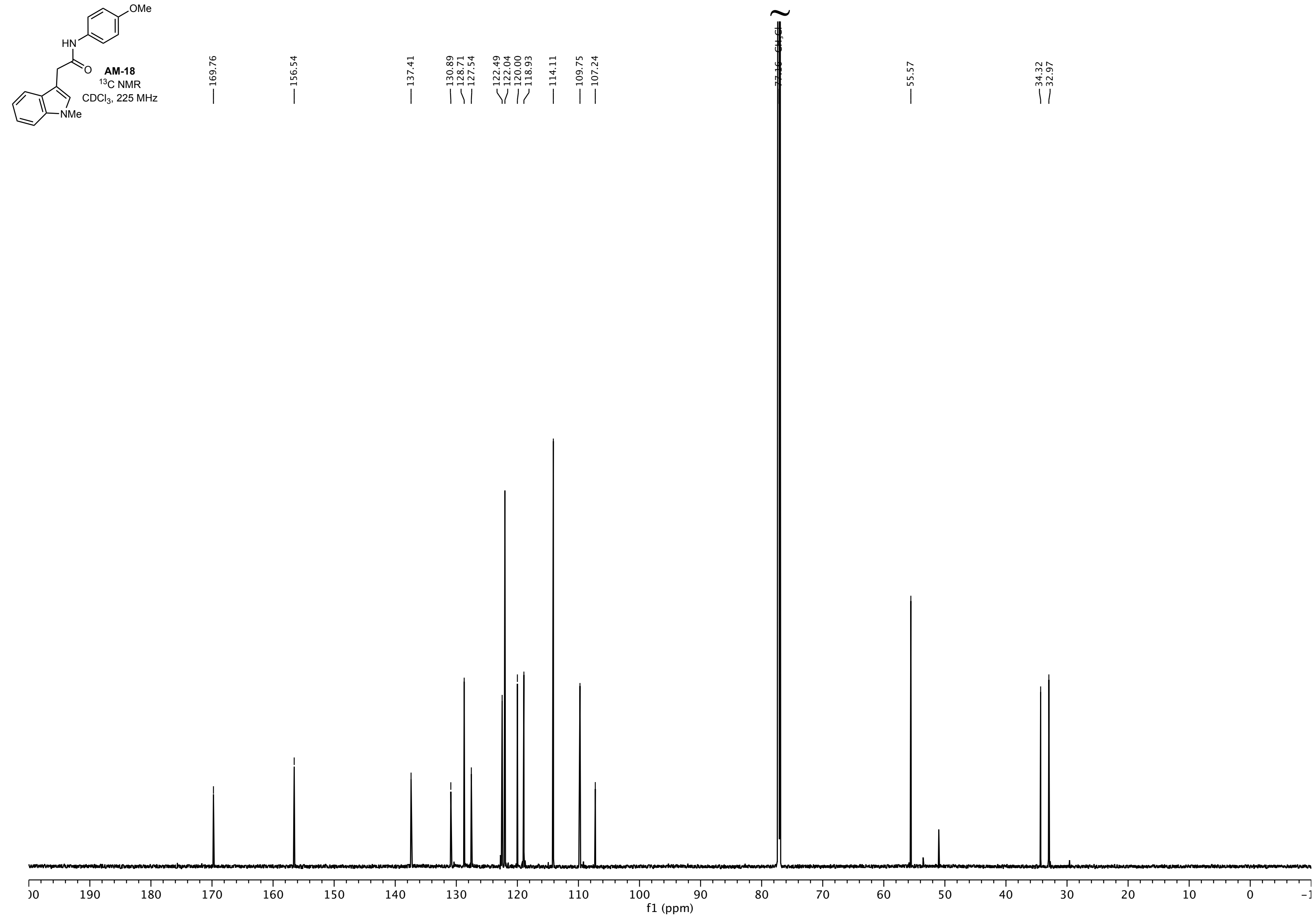
OMe
HN
O
NMe
AM-18
13C NMR
CDCl3, 225 MHz
169.76
156.54
137.41
130.89
128.71
127.54
122.49
122.04
120.00
118.93
114.11
109.75
107.24
55.57
34.32
32.97
f1 (ppm)

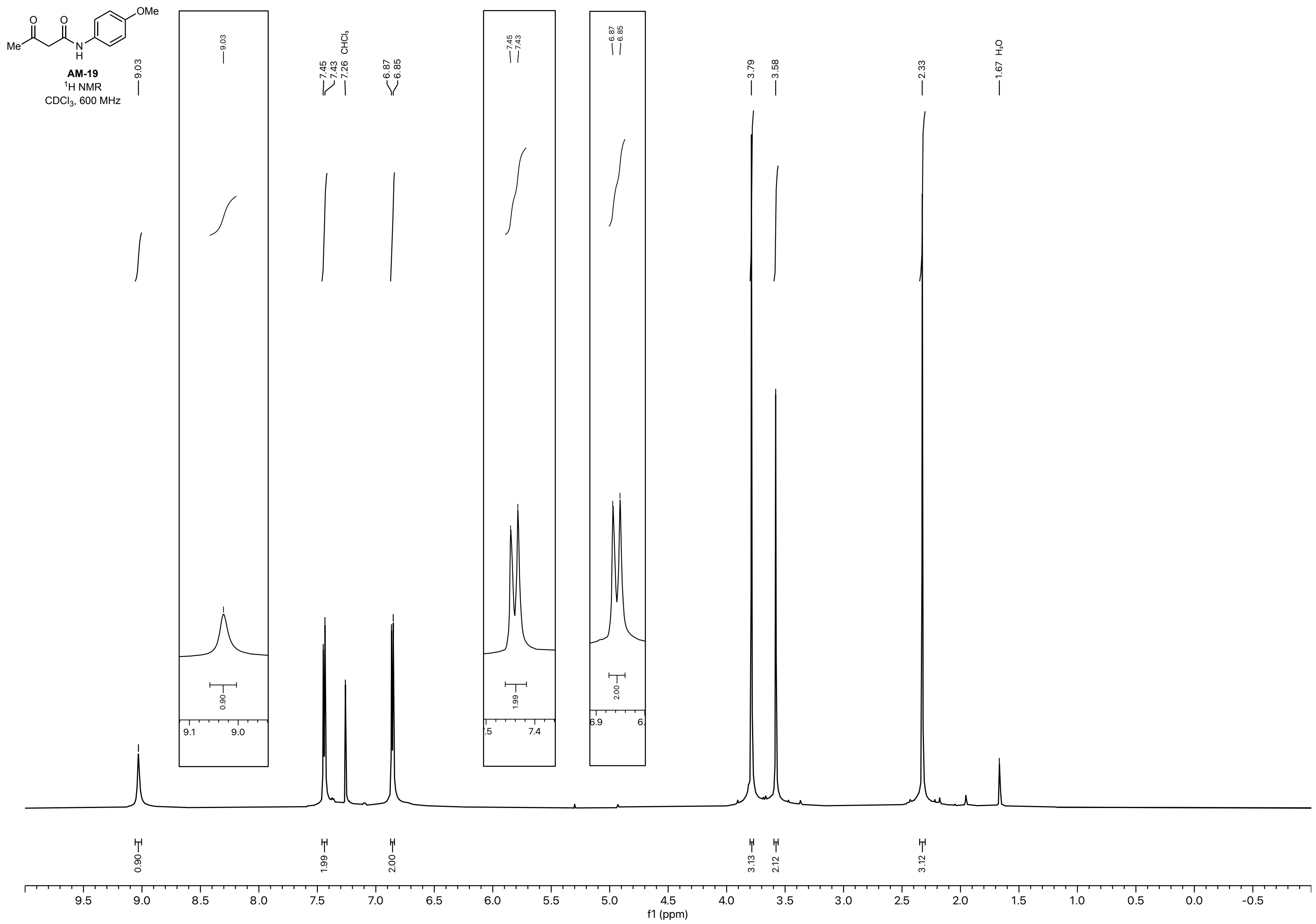
OMe
Me
O
O
N
H
AM-19
^{1}H NMR
$CDCl_3$, 600 MHz
9.03
7.45
7.43
7.26 $CHCl_3$
6.87
6.85
3.79
3.58
2.33
1.67 H_2O
0.90
1.99
2.00
3.13
2.12
3.12
f1 (ppm)

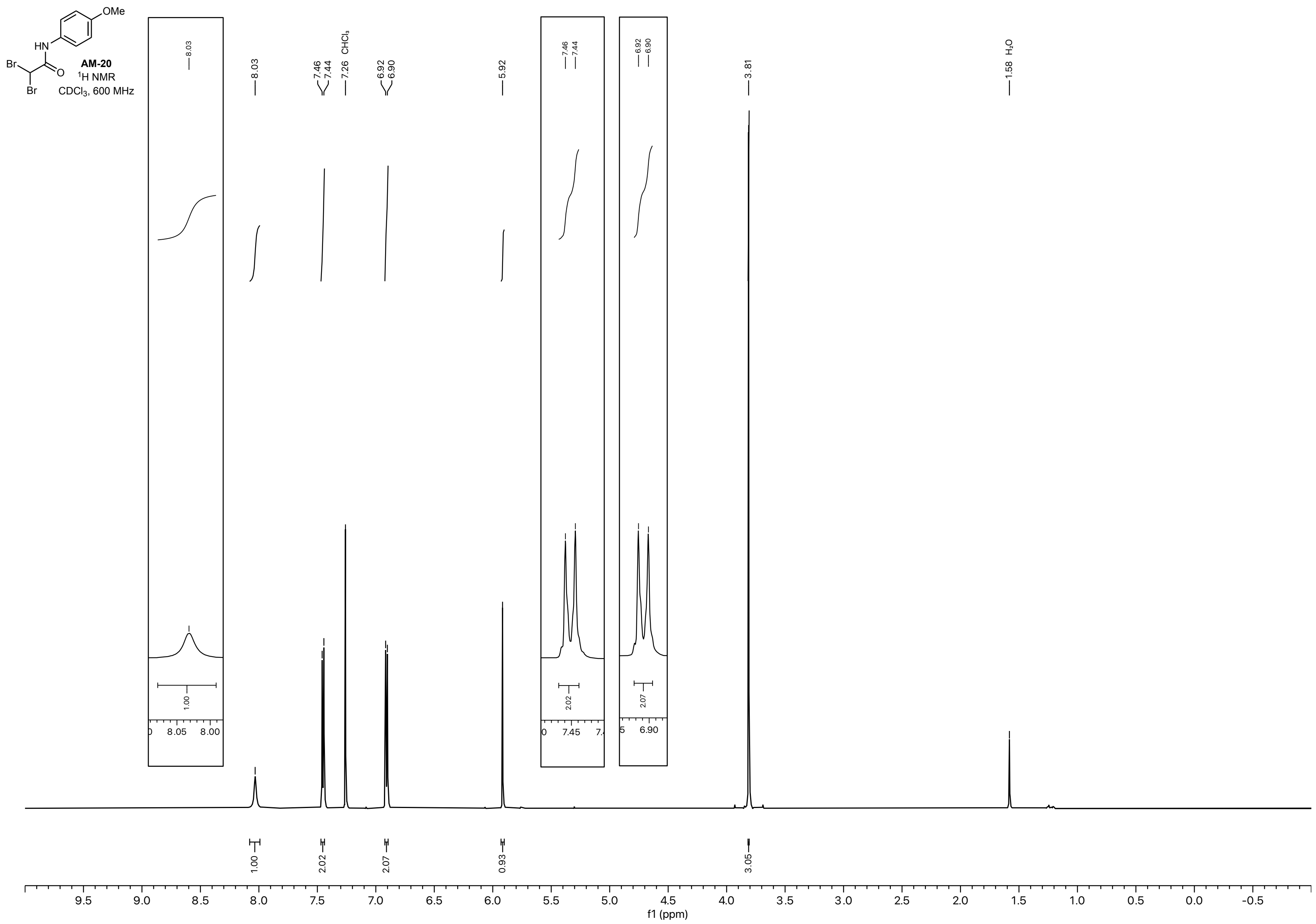

AM-20
^{1}H NMR
$CDCl_3$, 600 MHz
8.03
7.46
7.44
7.26 $CHCl_3$
6.92
6.90
5.92
3.81
1.58 H_2O
1.00
2.02
2.07
0.93
3.05
f1 (ppm)

Carbamates

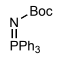

CM-02
^1H NMR
$CDCl_3$, 600 MHz

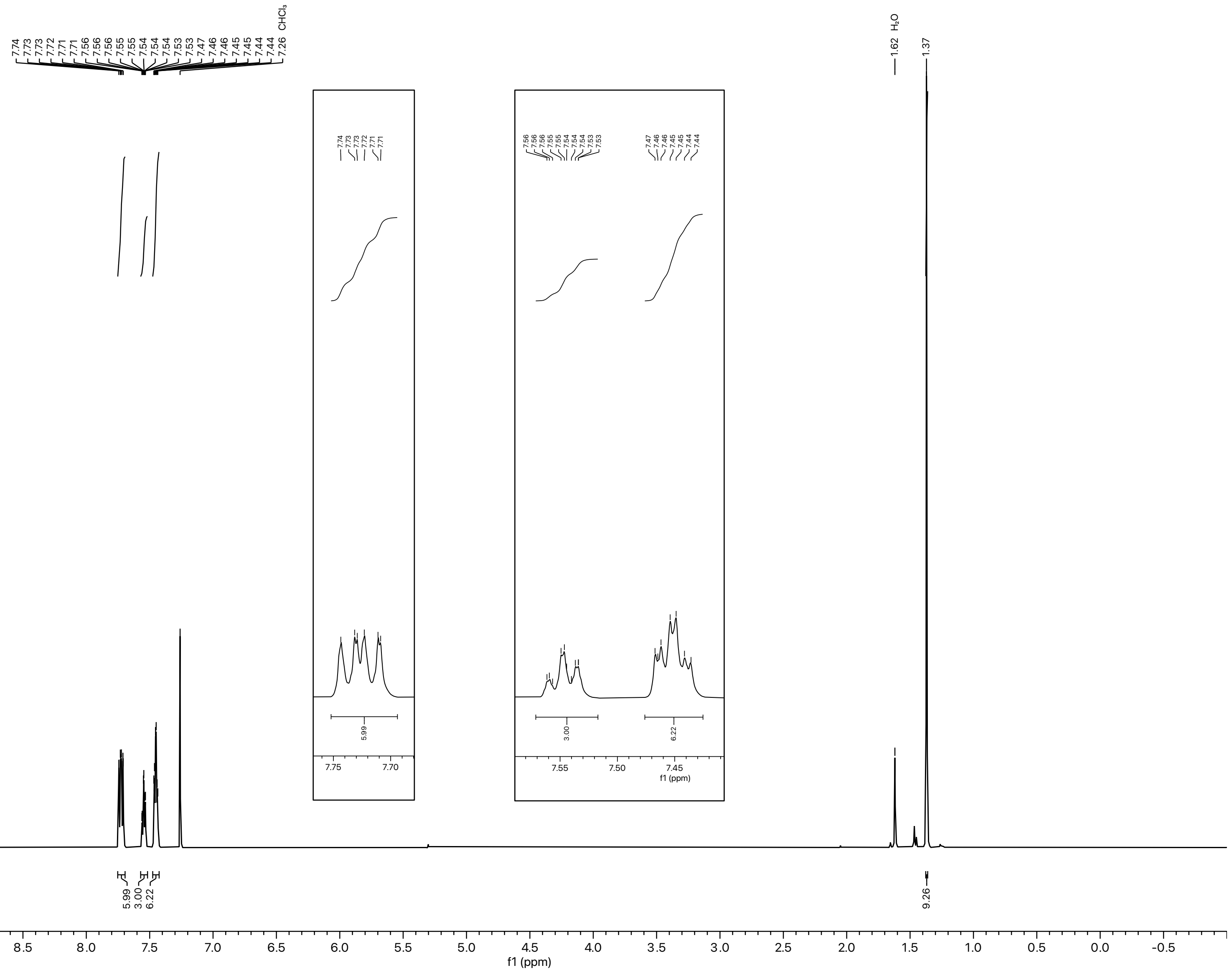

Ketenimines

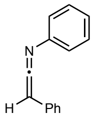

KE-01
^{1}H NMR
CD_3CN, 400 MHz

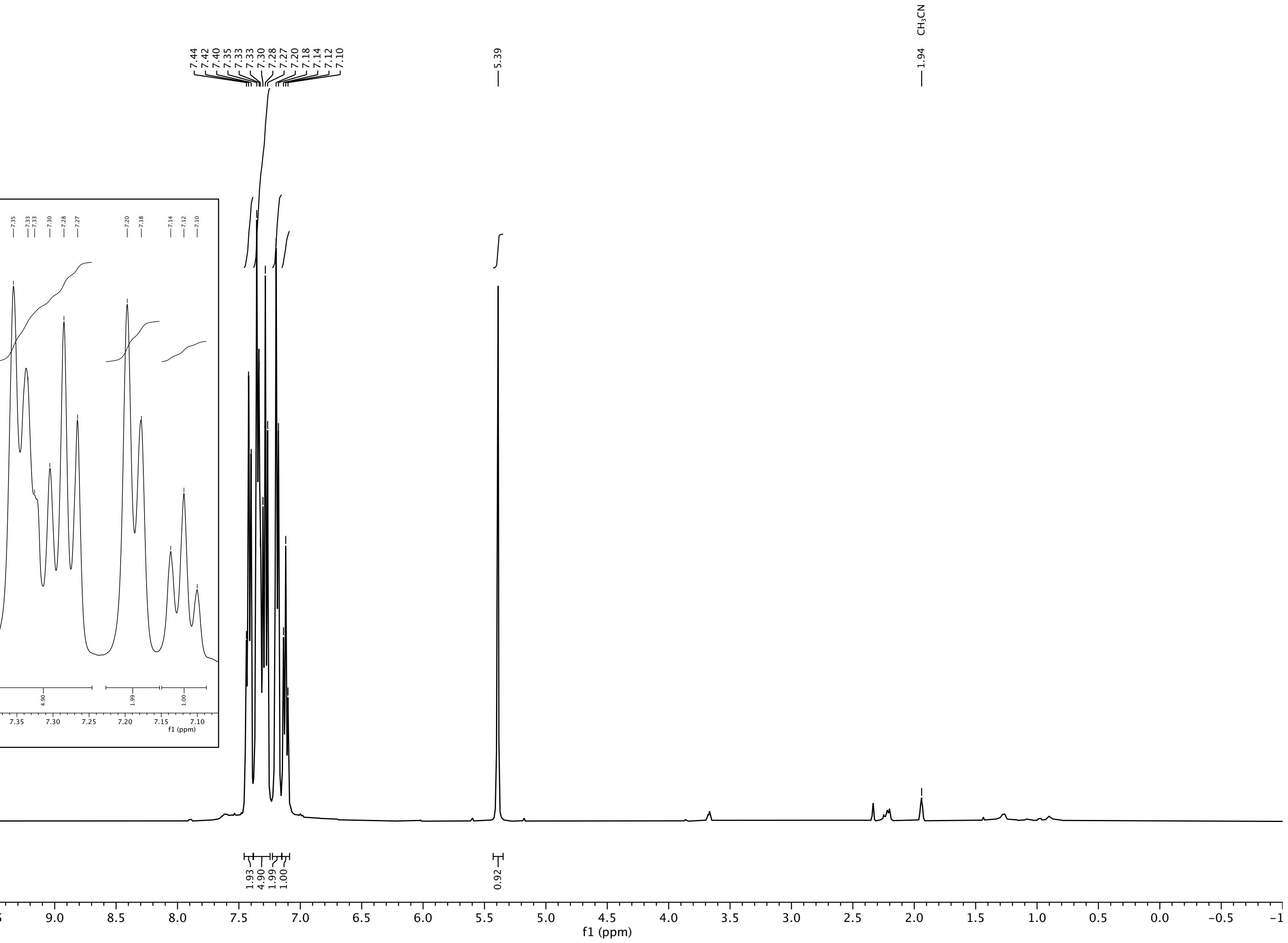

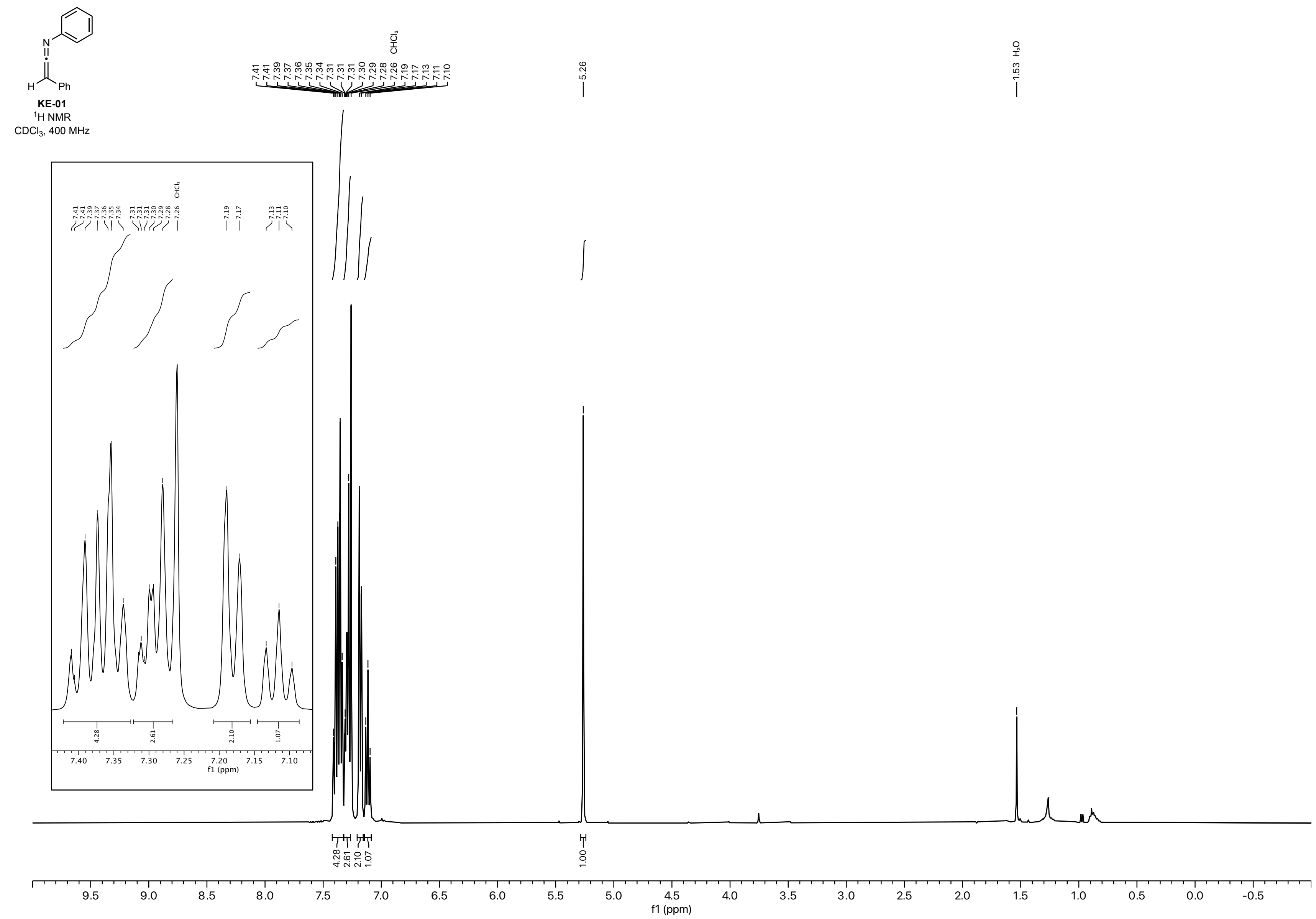
KE-01
^{1}H NMR
$CDCl_3$, 400 MHz
7.41 7.41 7.39 7.37 7.36 7.35 7.34 7.31 7.31 7.31 7.30 7.29 7.28 7.26 7.19 7.17 7.13 7.11 7.10
$CHCl_3$
5.26
1.53 H_2O
4.28 2.61 2.10 1.07
1.00
f1 (ppm)

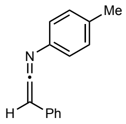

KE-02
^1H NMR
$CDCl_3$, 400 MHz

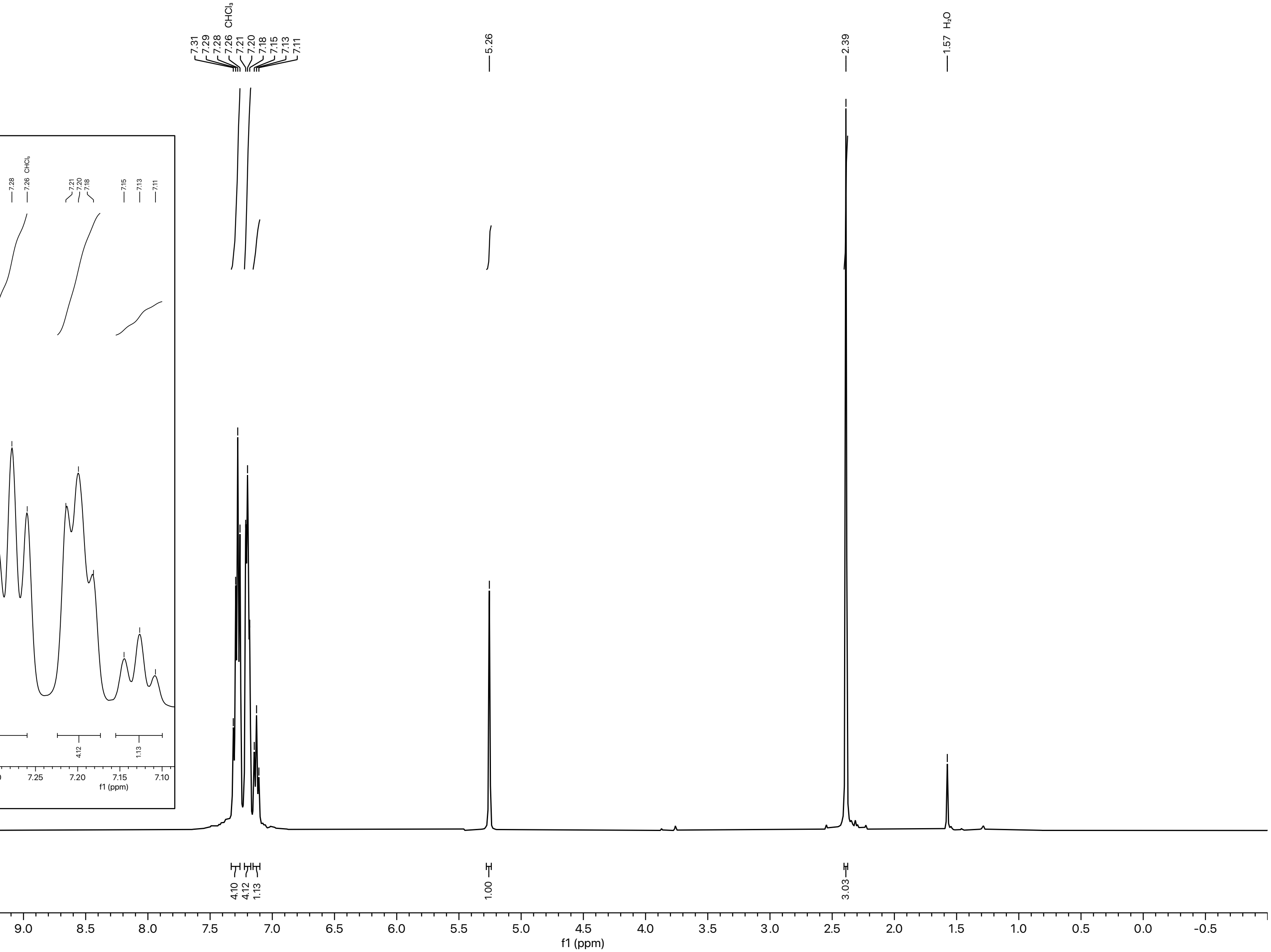

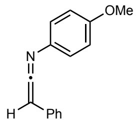

KE-03 (or **2a**)
^{1}H NMR
$CDCl_3$, 400 MHz

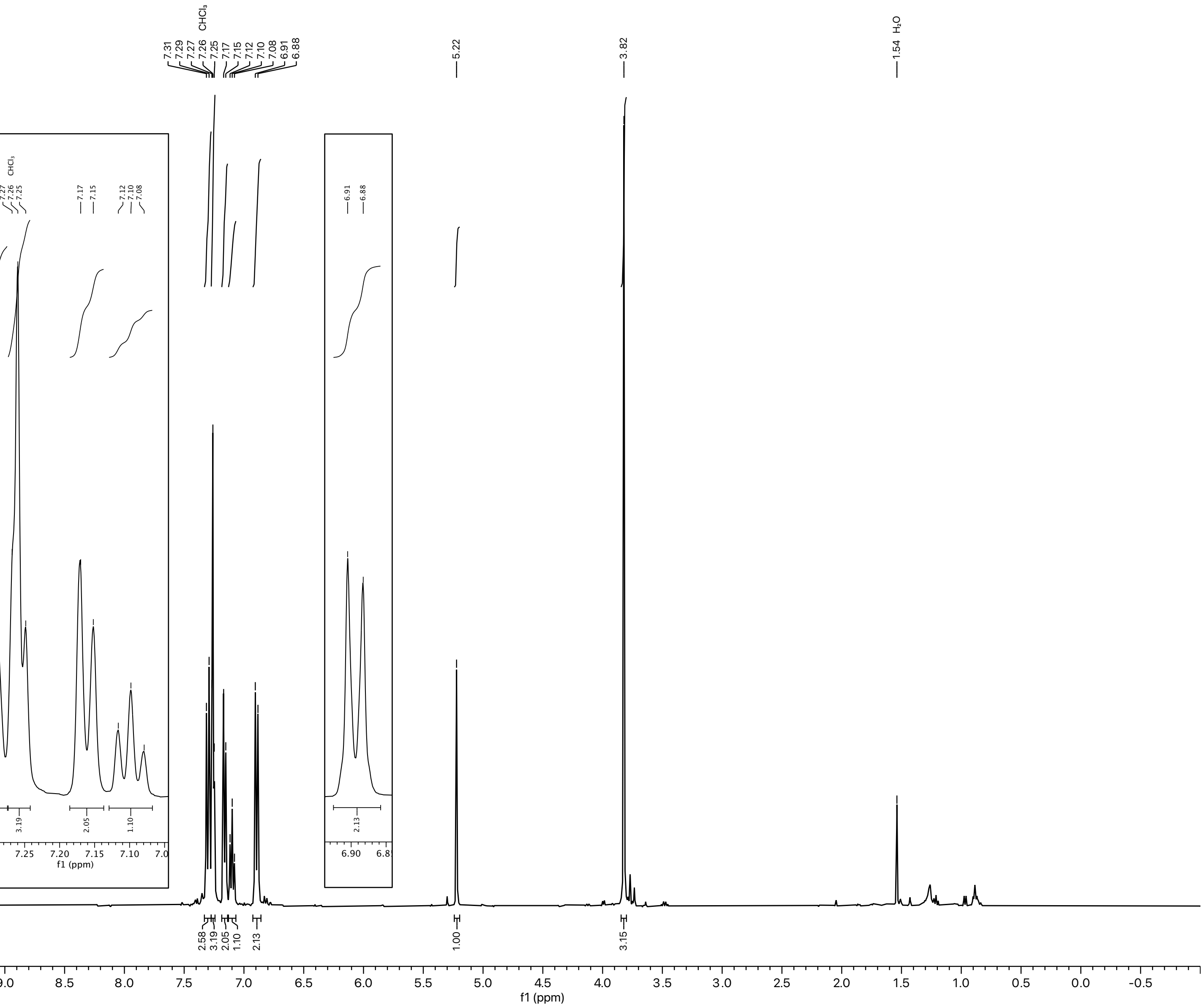

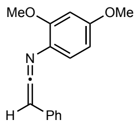

KE-04
^{1}H NMR
$CDCl_3$, 900 MHz

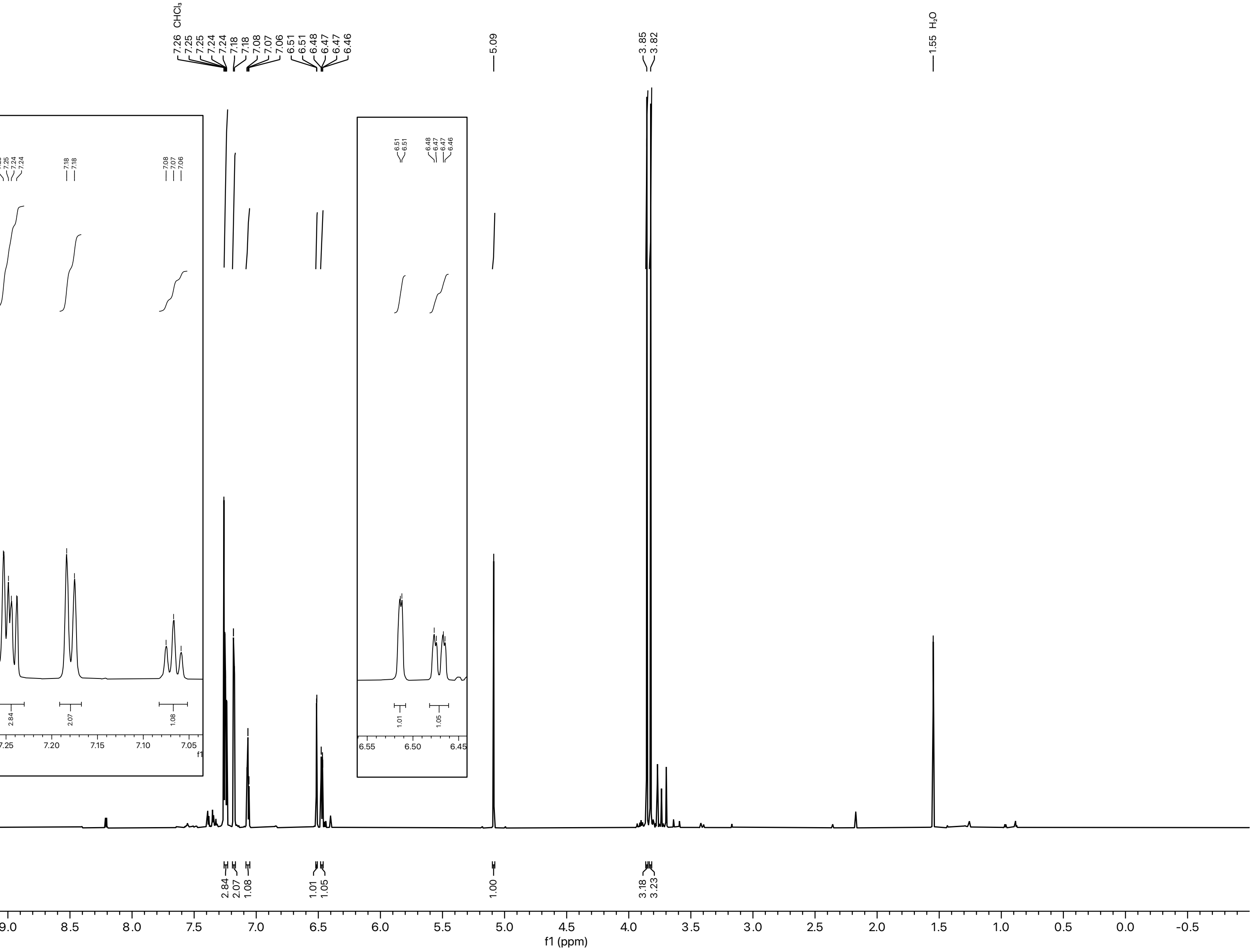

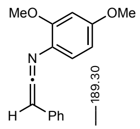

KE-04
^{13}C NMR
$CDCl_3$, 225 MHz

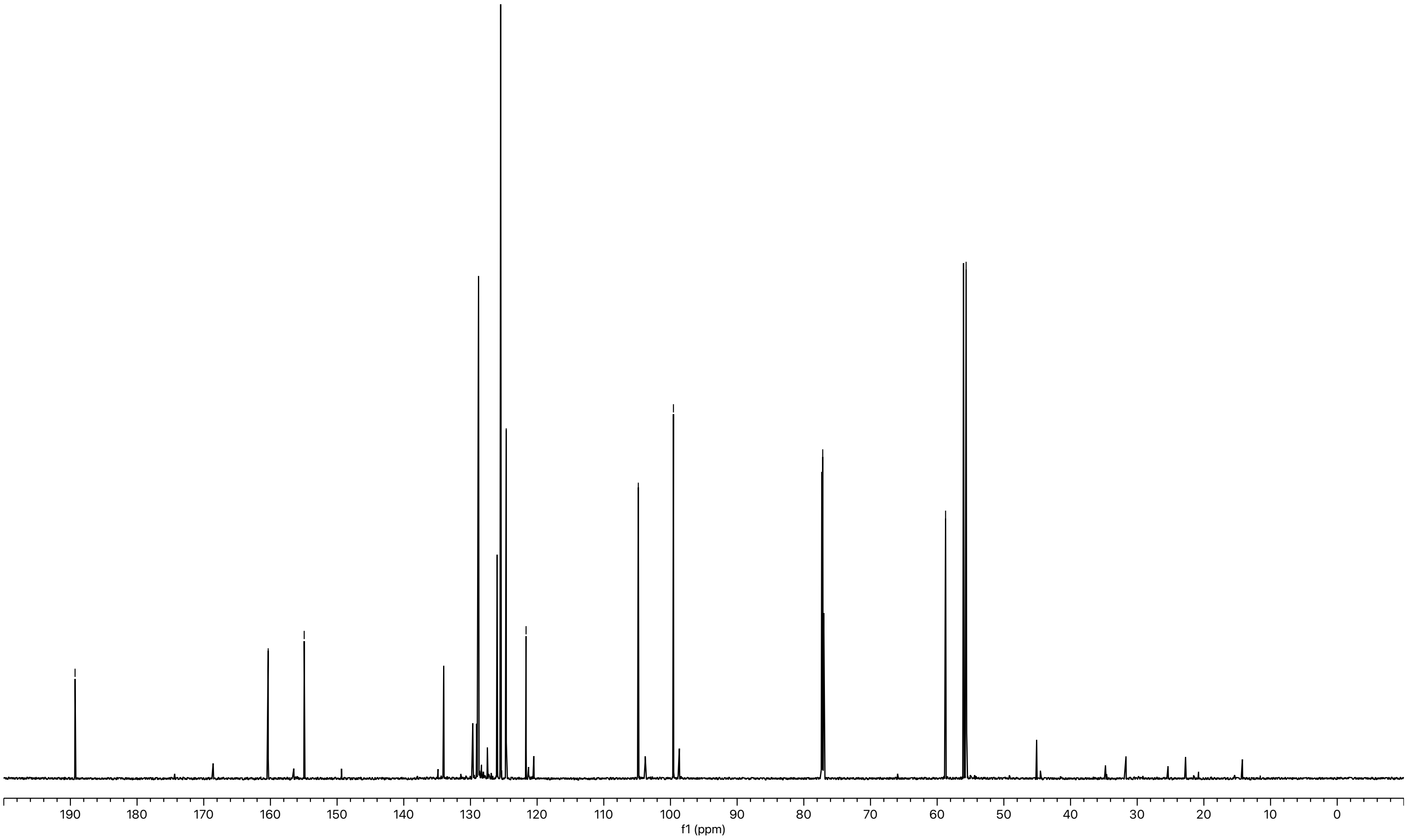

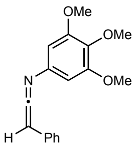

KE-05
^{1}H NMR
$CDCl_3$, 400 MHz

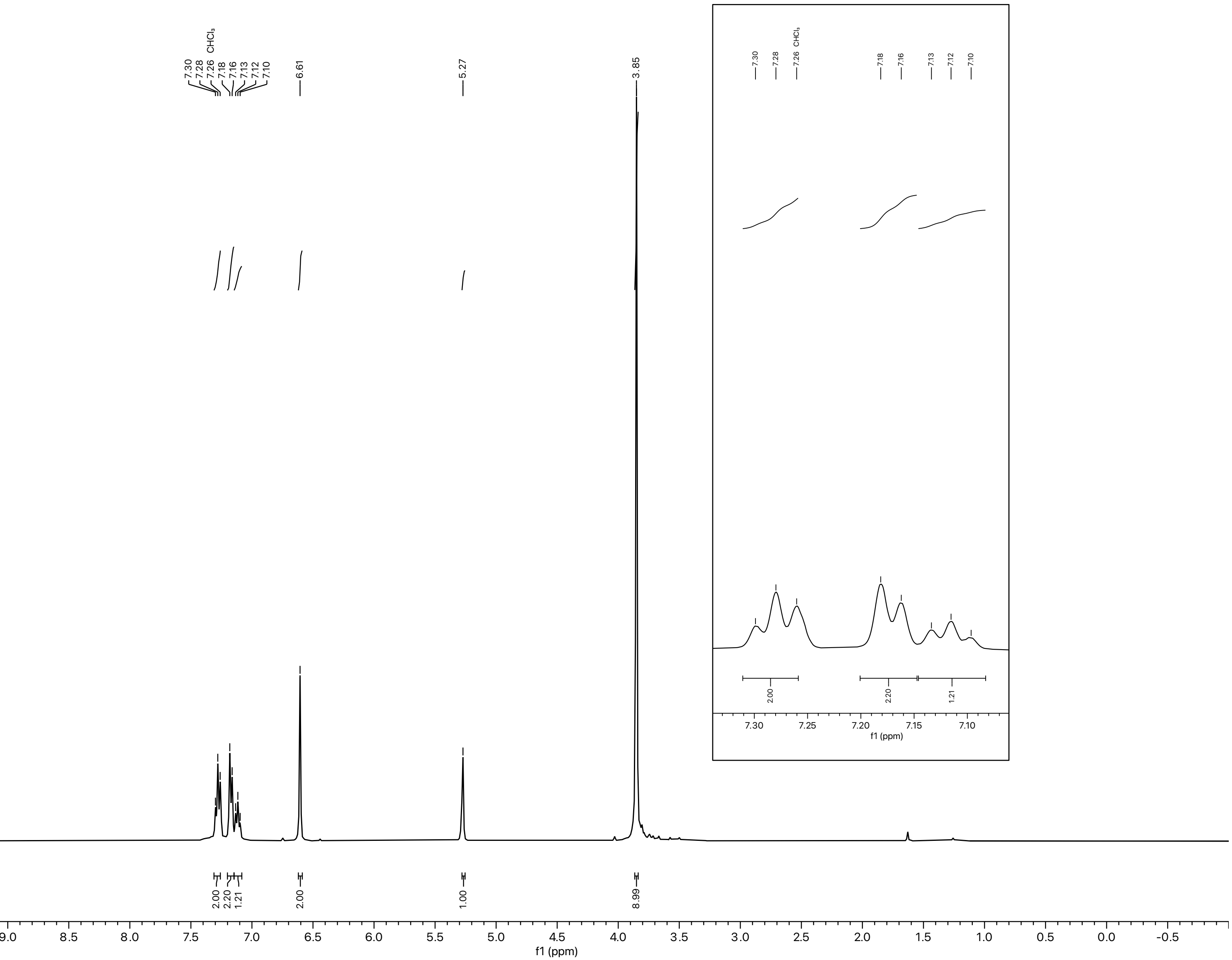

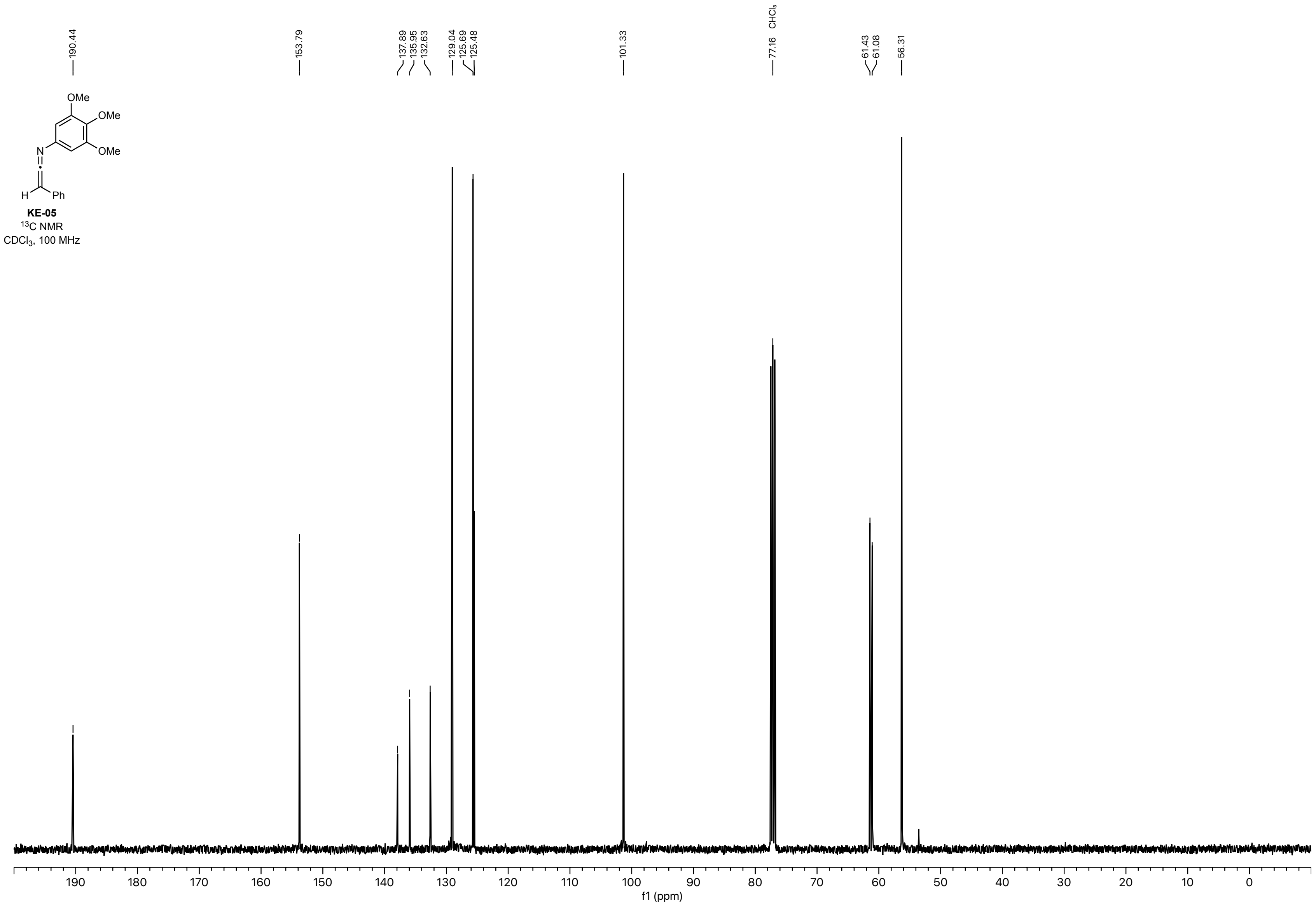

KE-05
^{13}C NMR
$CDCl_3$, 100 MHz

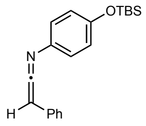

KE-06
^{1}H NMR
$CDCl_3$, 600 MHz

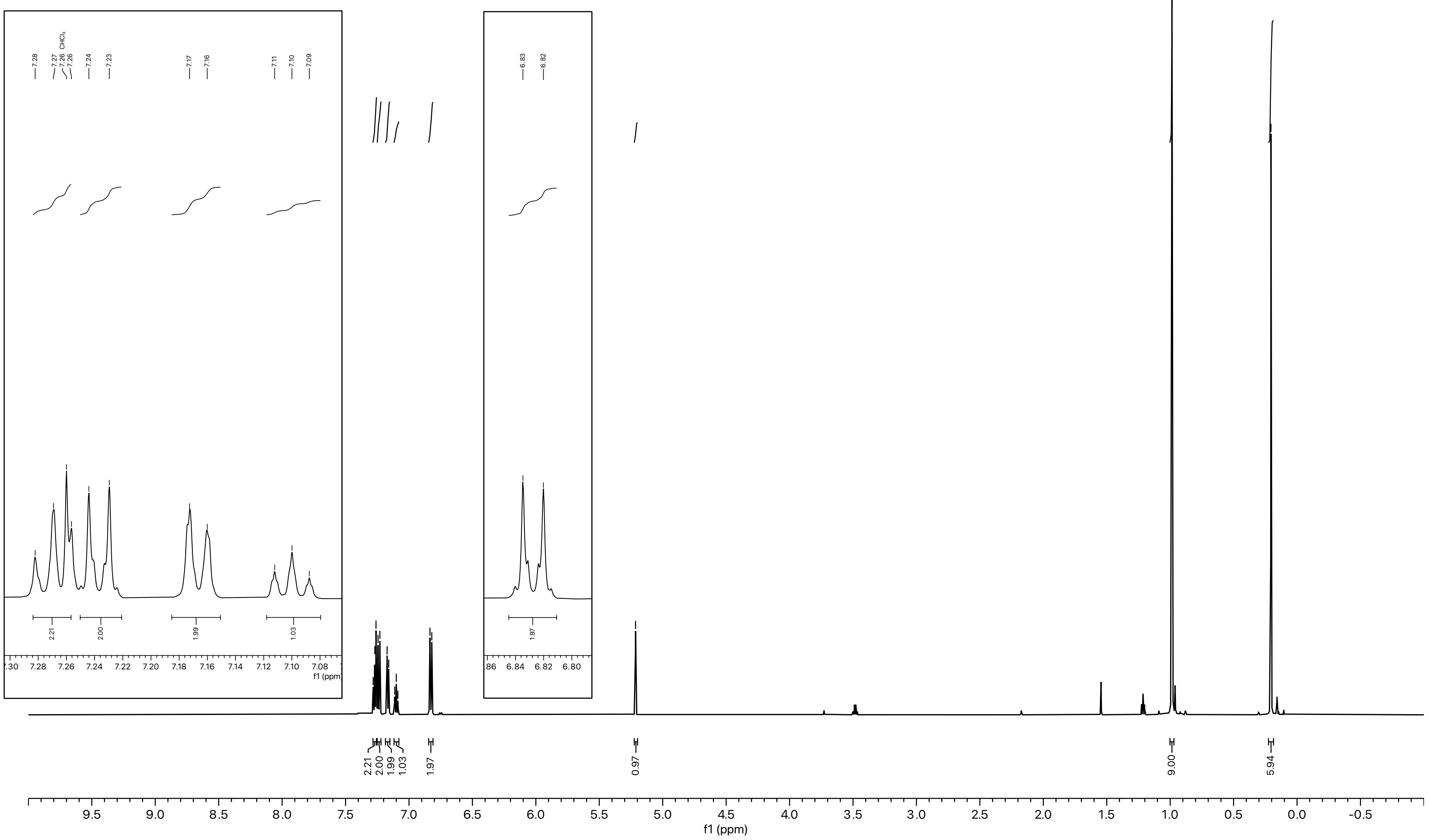

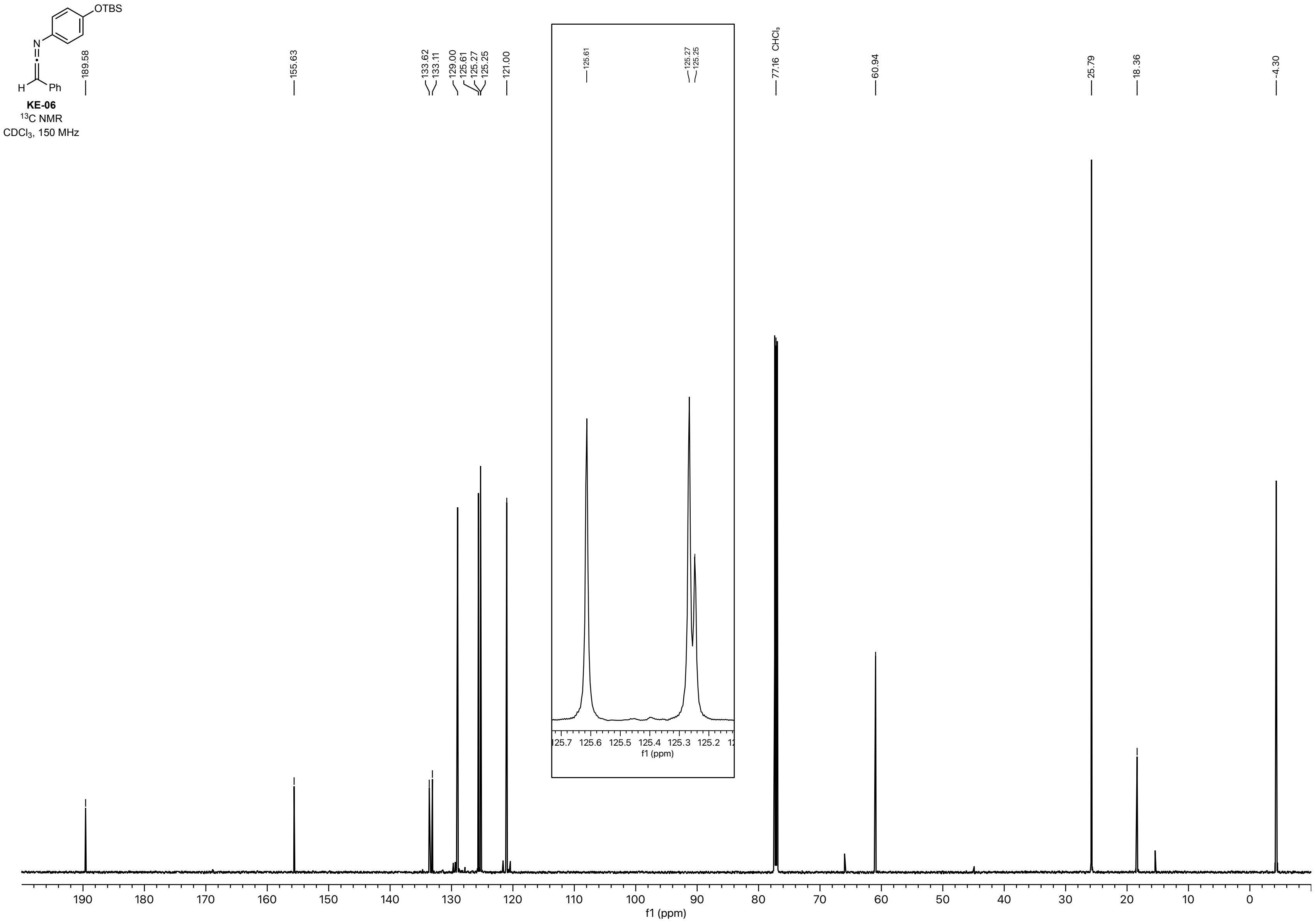
OTBS
N
H
Ph
KE-06
13C NMR
CDCl3, 150 MHz
189.58
155.63
133.62
133.11
129.00
125.61
125.27
125.25
121.00
77.16 CHCl3
60.94
25.79
18.36
-4.30
125.7 125.6 125.5 125.4 125.3 125.2
f1 (ppm)
190 180 170 160 150 140 130 120 110 100 90 80 70 60 50 40 30 20 10 0
f1 (ppm)

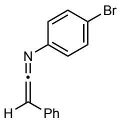

KE-07
^{1}H NMR
$CDCl_3$, 400 MHz

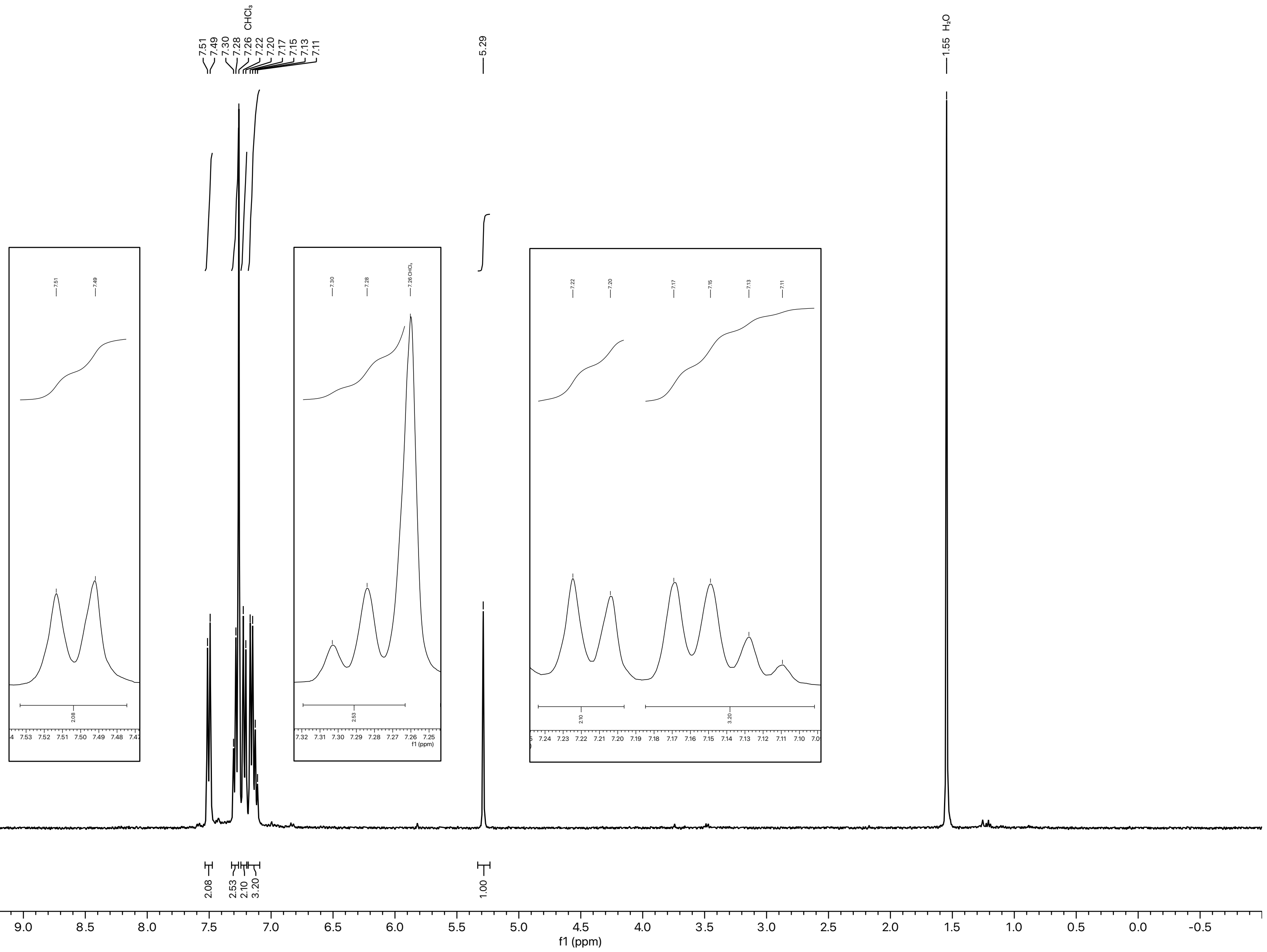

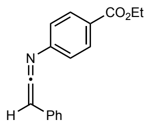

KE-08
^{1}H NMR
$CDCl_3$, 400 MHz

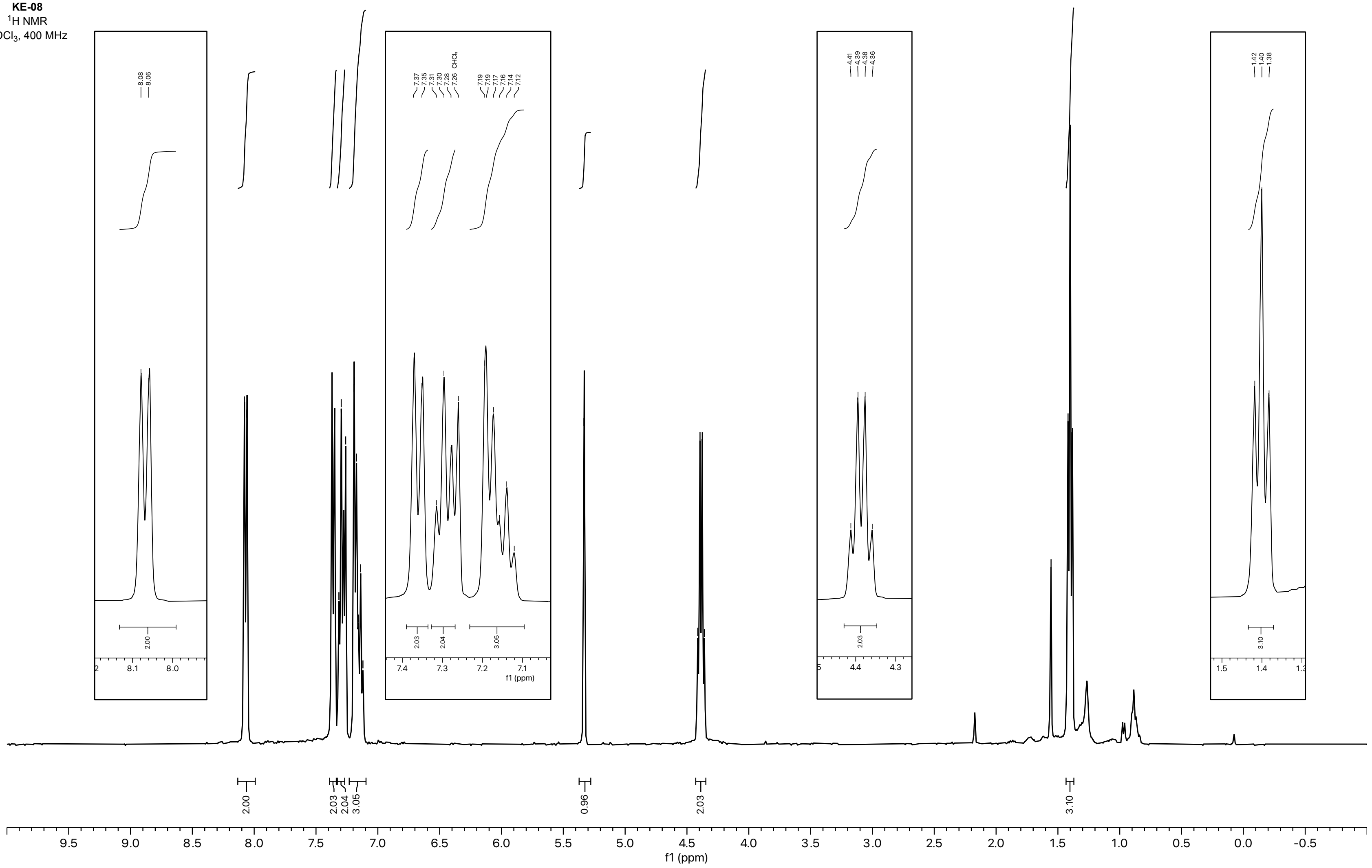

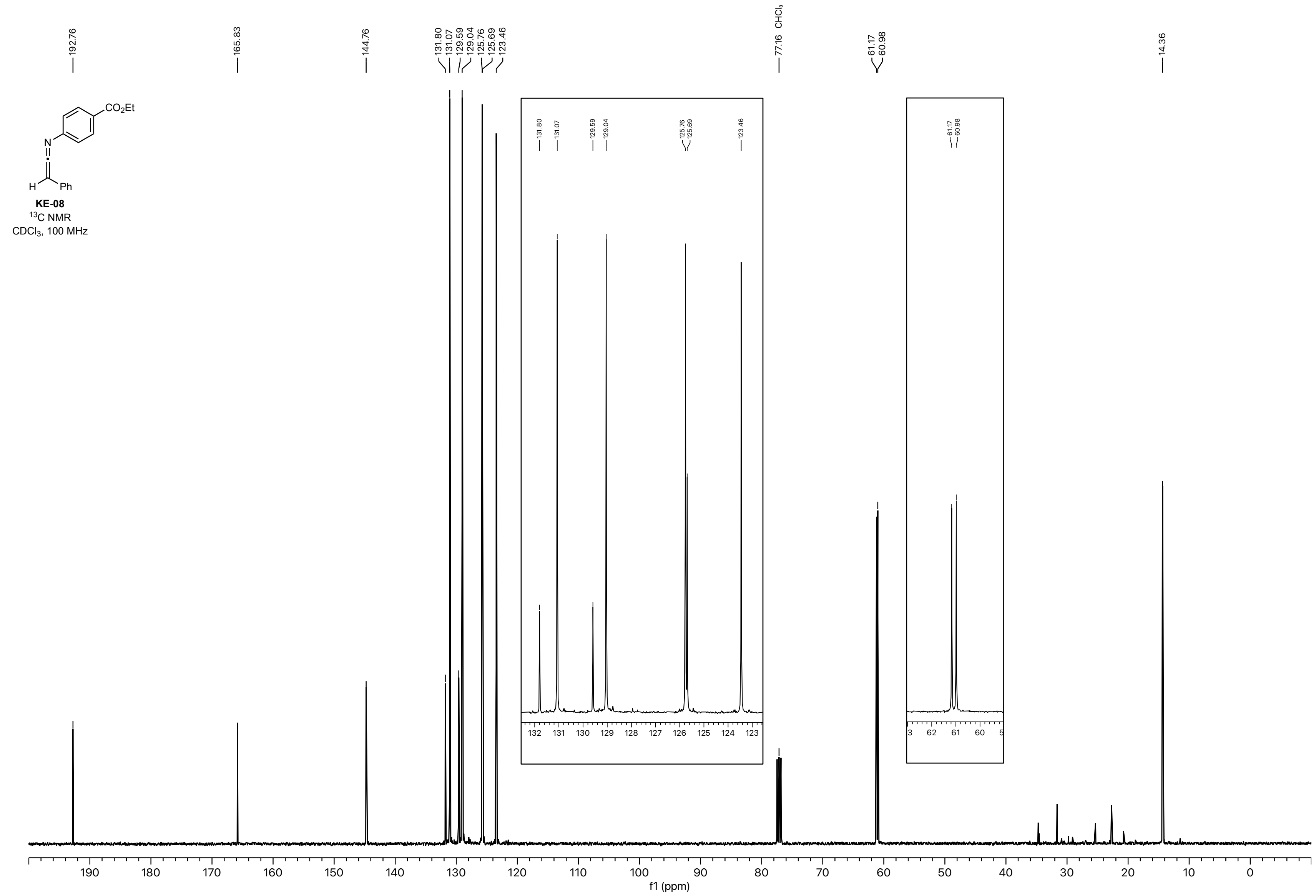

192.76
165.83
144.76
131.80
131.07
129.59
129.04
125.76
125.69
123.46
77.16 CHCl3
61.17
60.98
14.36
CO2Et
N
H
Ph
KE-08
13C NMR
CDCl3, 100 MHz
f1 (ppm)

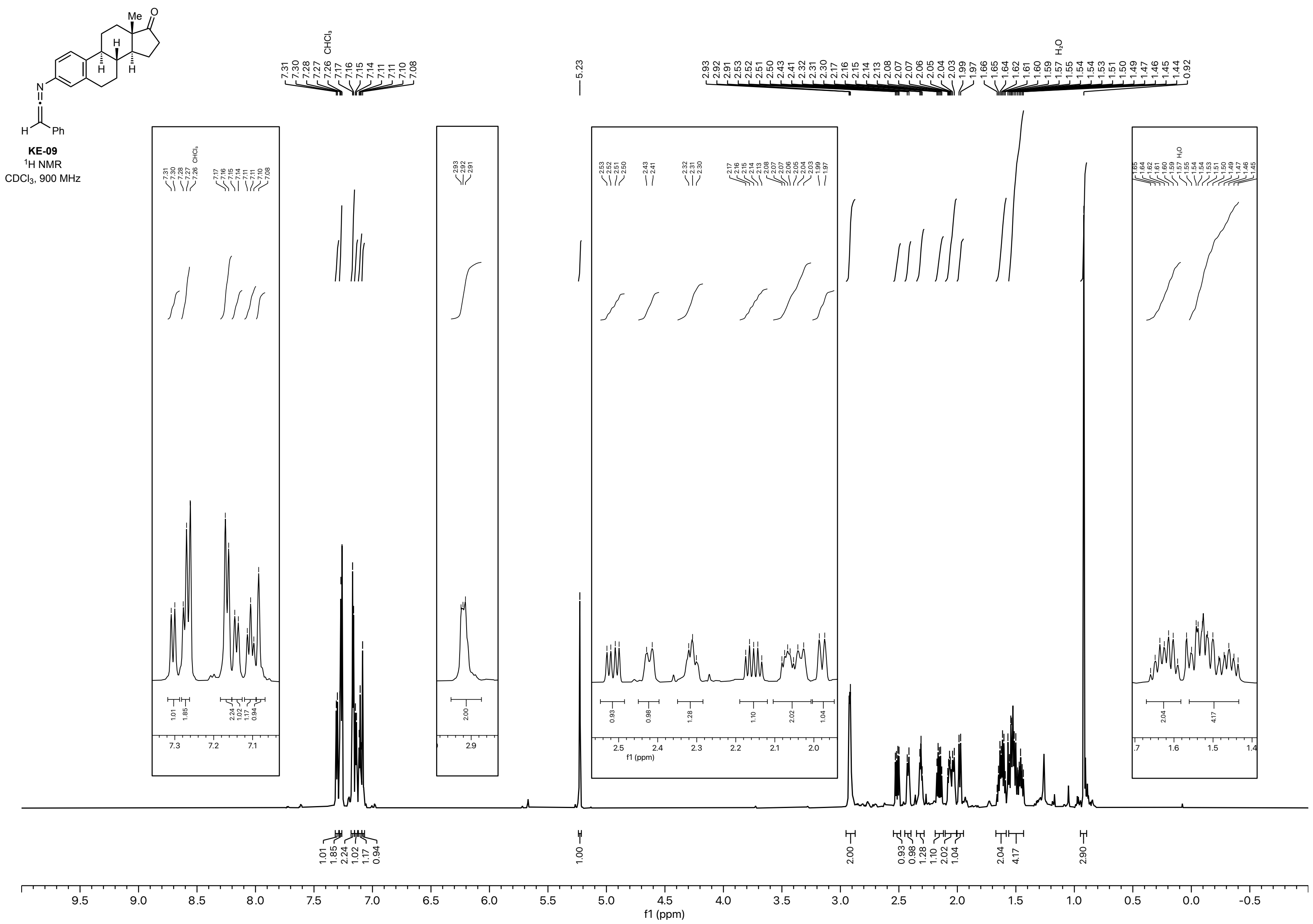
Me
H
N
Ph
KE-09
¹H NMR
CDCl₃, 900 MHz
f1 (ppm)

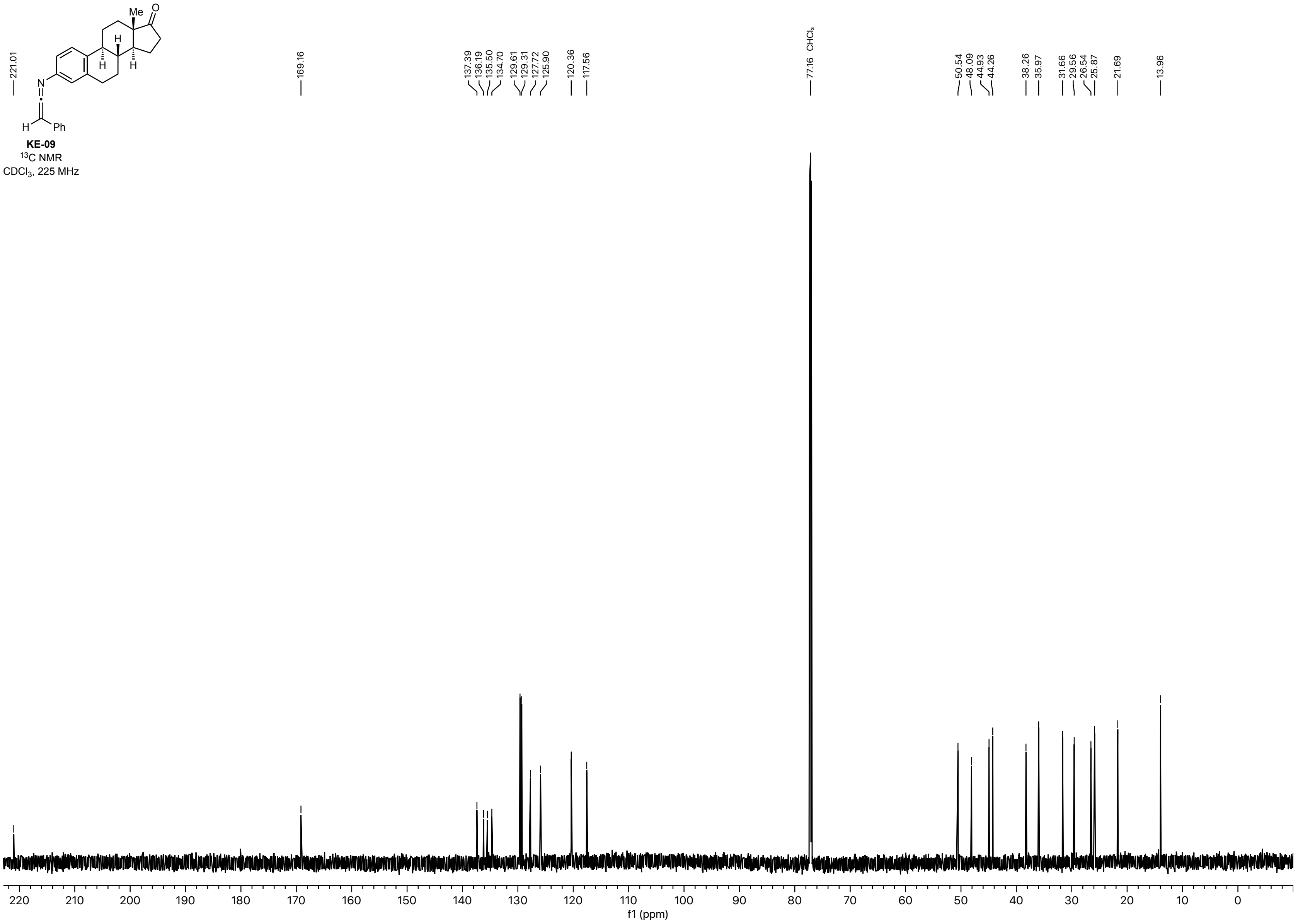

Me
O
H
H
H
N
H
Ph
KE-09
13C NMR
CDCl3, 225 MHz
221.01
169.16
137.39
136.19
135.50
134.70
129.61
129.31
127.72
125.90
120.36
117.56
77.16 CHCl3
50.54
48.09
44.93
44.26
38.26
35.97
31.66
29.56
26.54
25.87
21.69
13.96
220
210
200
190
180
170
160
150
140
130
120
110
100
90
80
70
60
50
40
30
20
10
0
f1 (ppm)

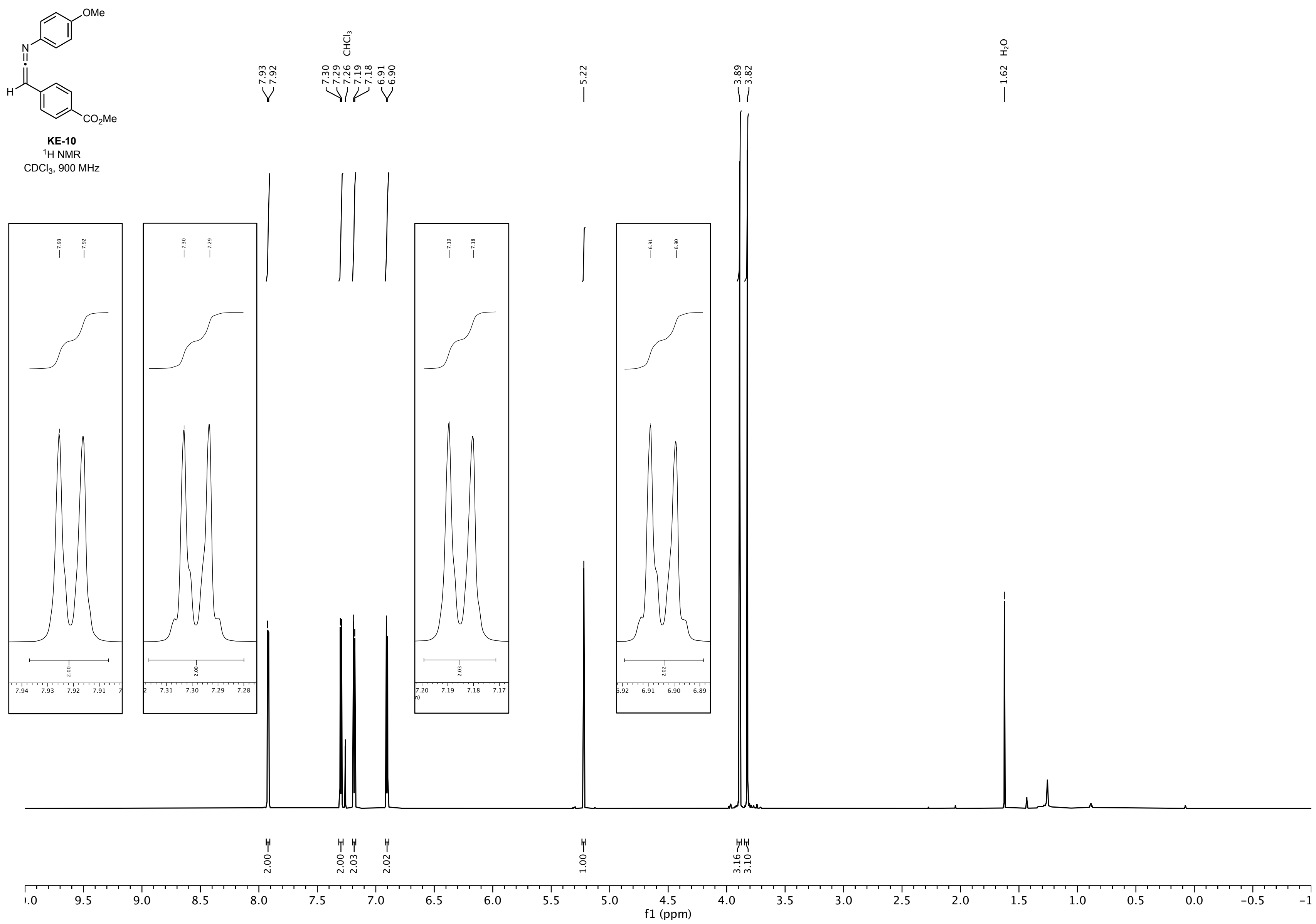

OMe
N
H
CO2Me
KE-10
1H NMR
CDCl3, 900 MHz
7.93
7.92
7.30
7.29
7.26
CHCl3
7.26
7.19
7.18
6.91
6.90
5.22
3.89
3.82
1.62 H2O
2.00
2.00
2.03
2.02
1.00
3.16
3.10
f1 (ppm)

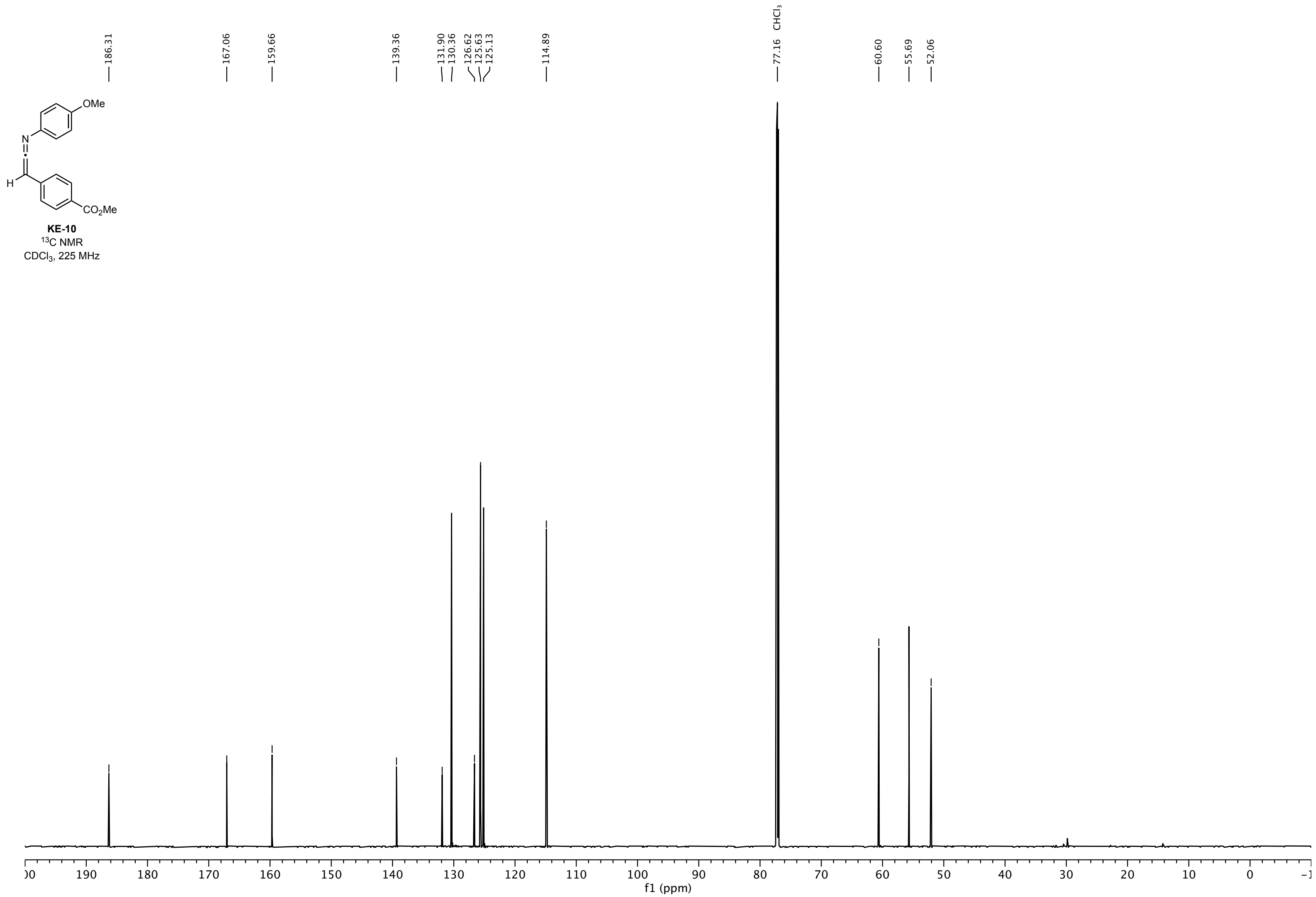

KE-10
^{13}C NMR
$CDCl_3$, 225 MHz

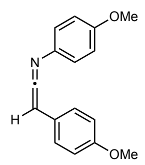

KE-11
^{1}H NMR
$(CD_3)_2CO$, 400 MHz

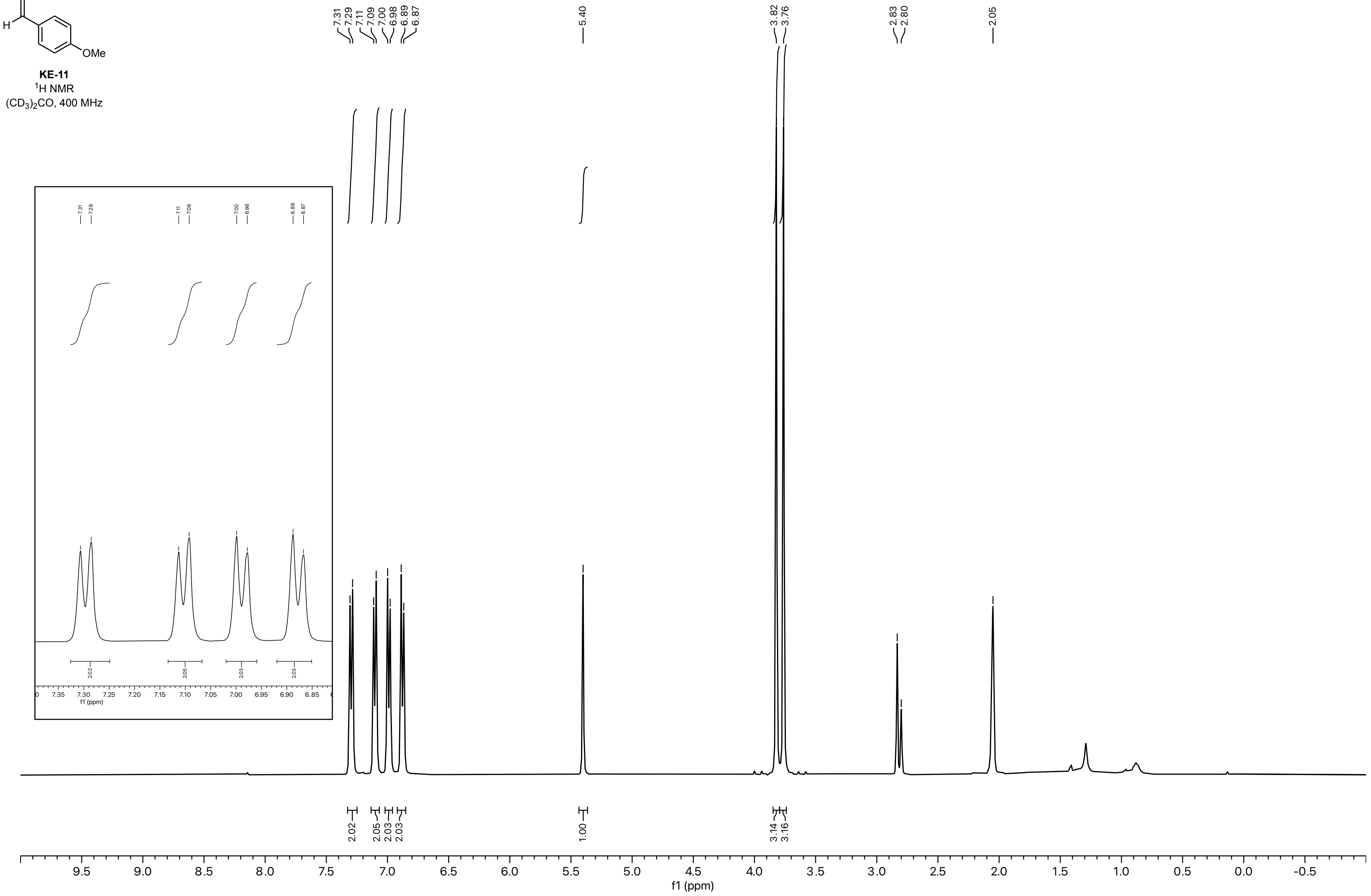

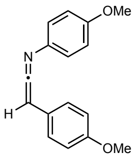

KE-11
^{13}C NMR
$(CD_3)_2CO$, 100 MHz

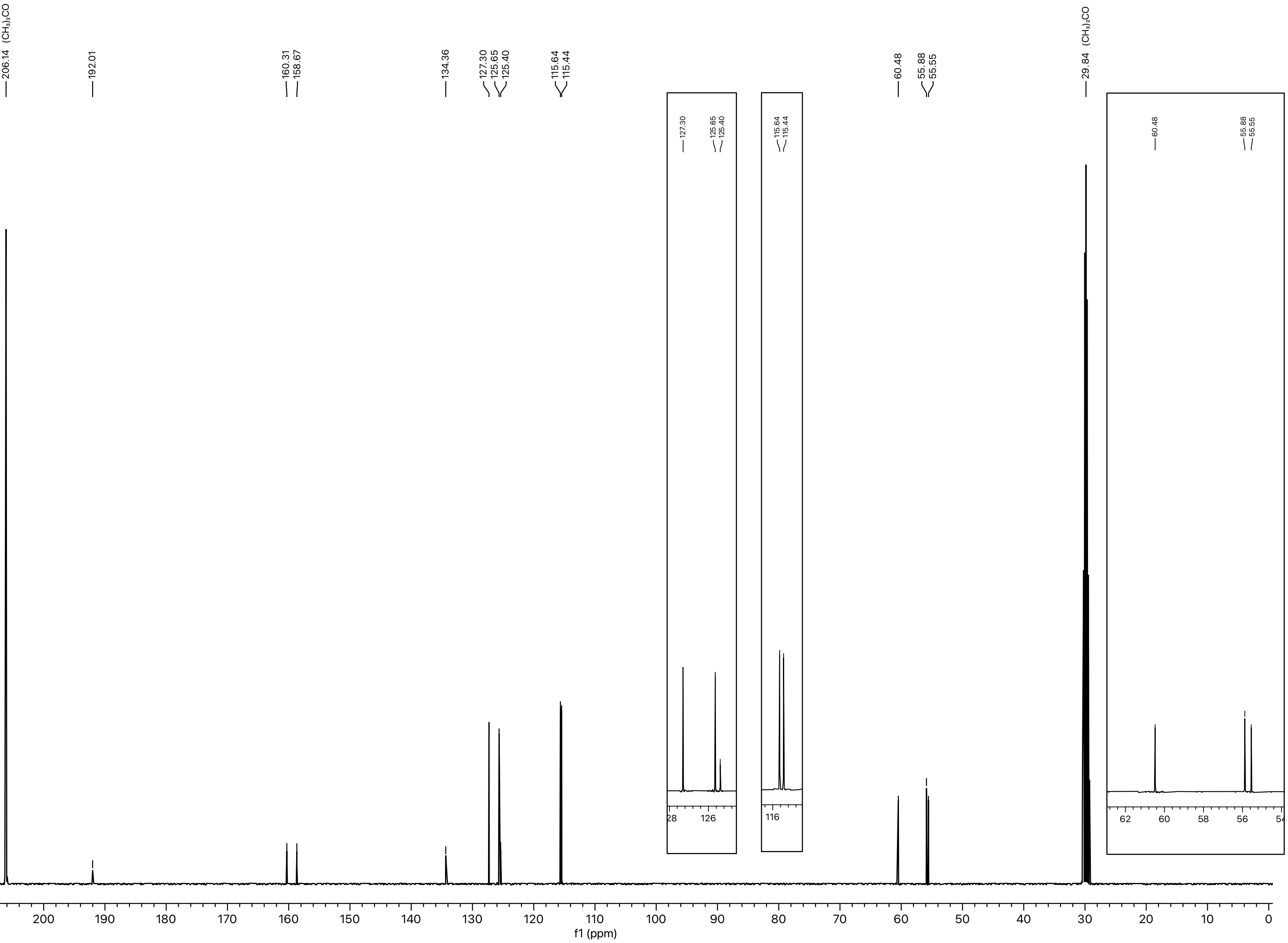

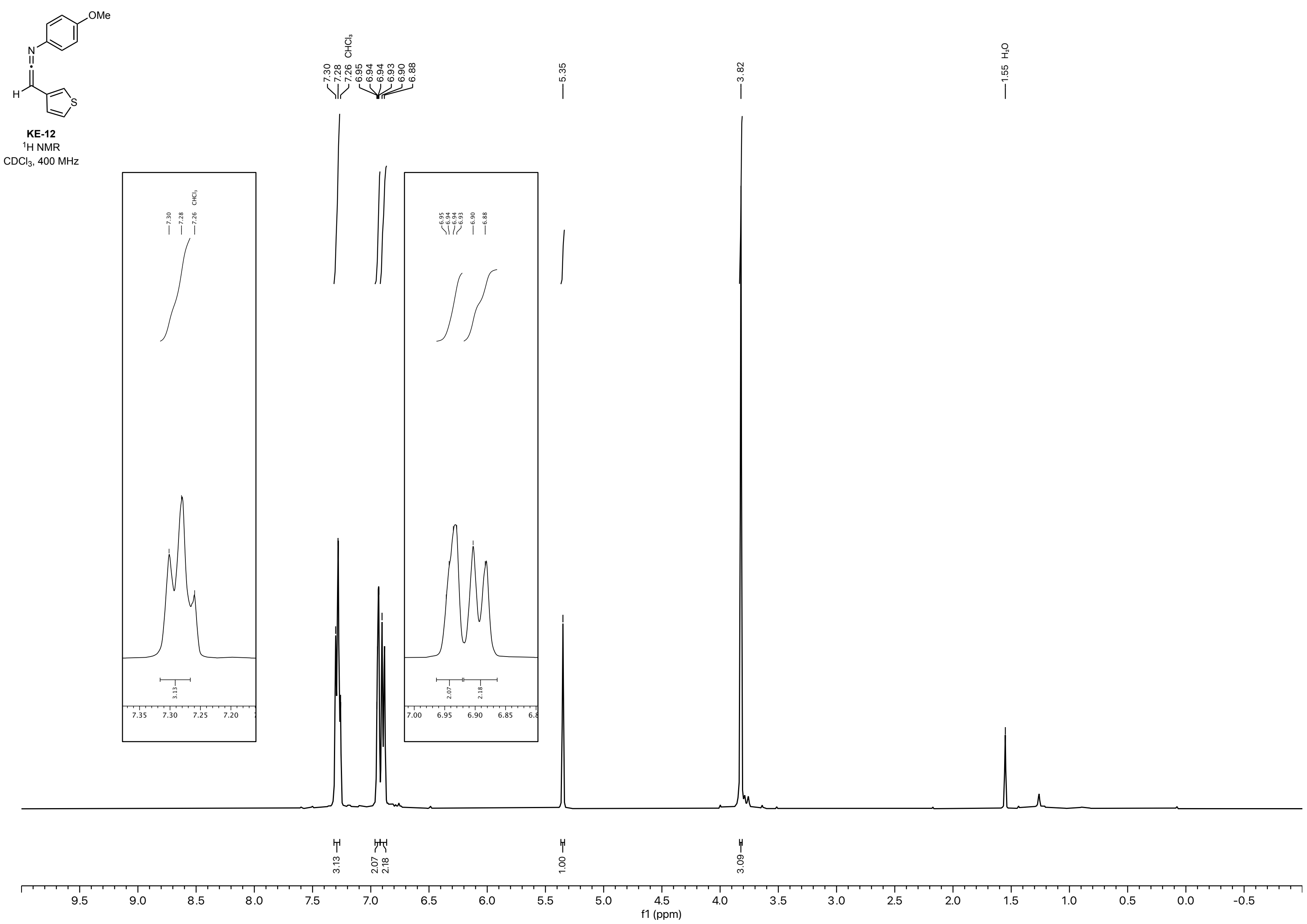
OMe
N
H
S
KE-12
^{1}H NMR
$CDCl_3$, 400 MHz
7.30
7.28
7.26 $CHCl_3$
6.95
6.94
6.94
6.93
6.90
6.88
5.35
3.82
1.55 H_2O
3.13
2.07
2.18
1.00
3.09
f1 (ppm)

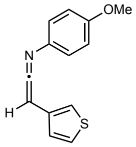

KE-12
^{13}C NMR
$CDCl_3$, 100 MHz

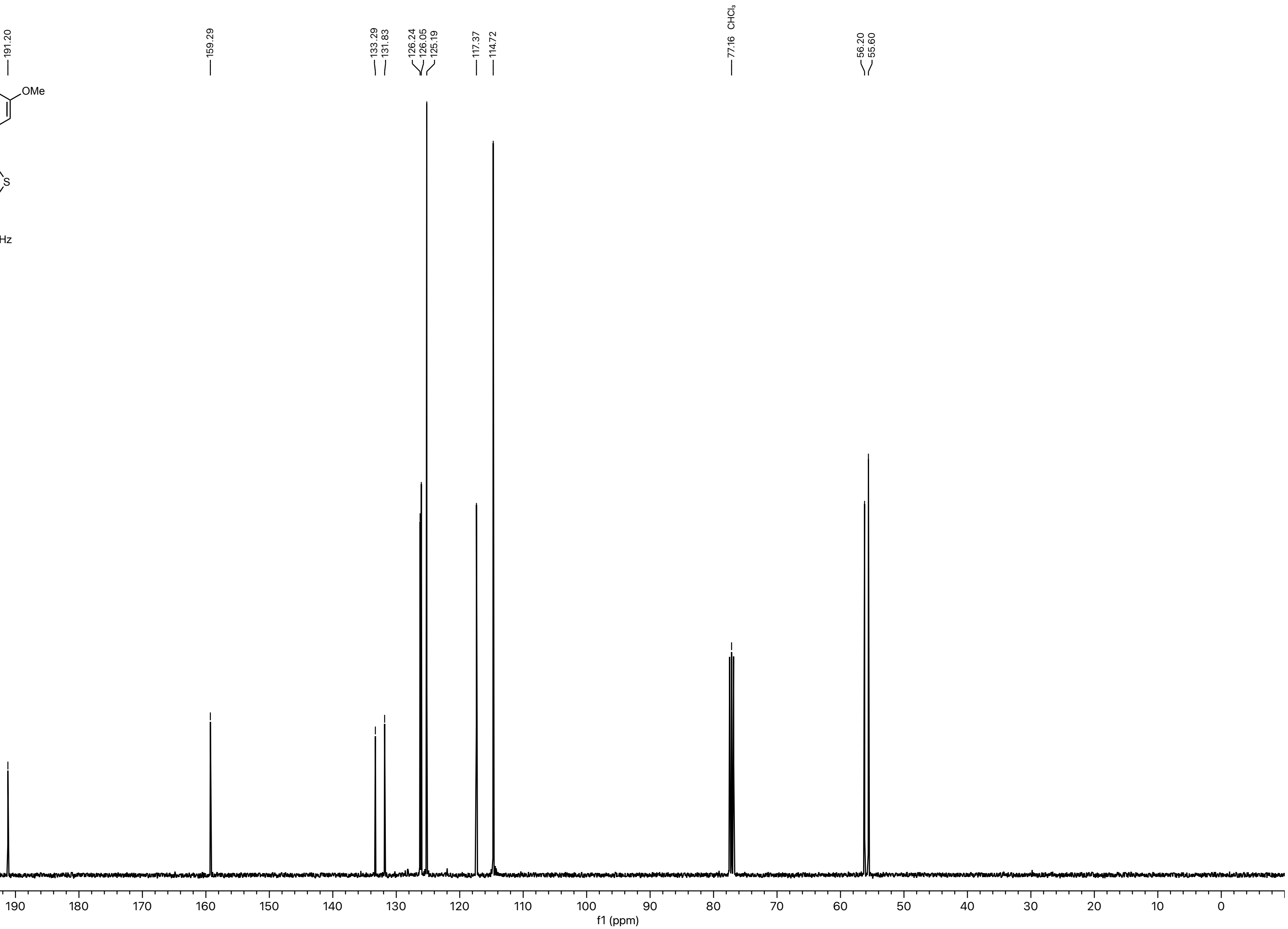

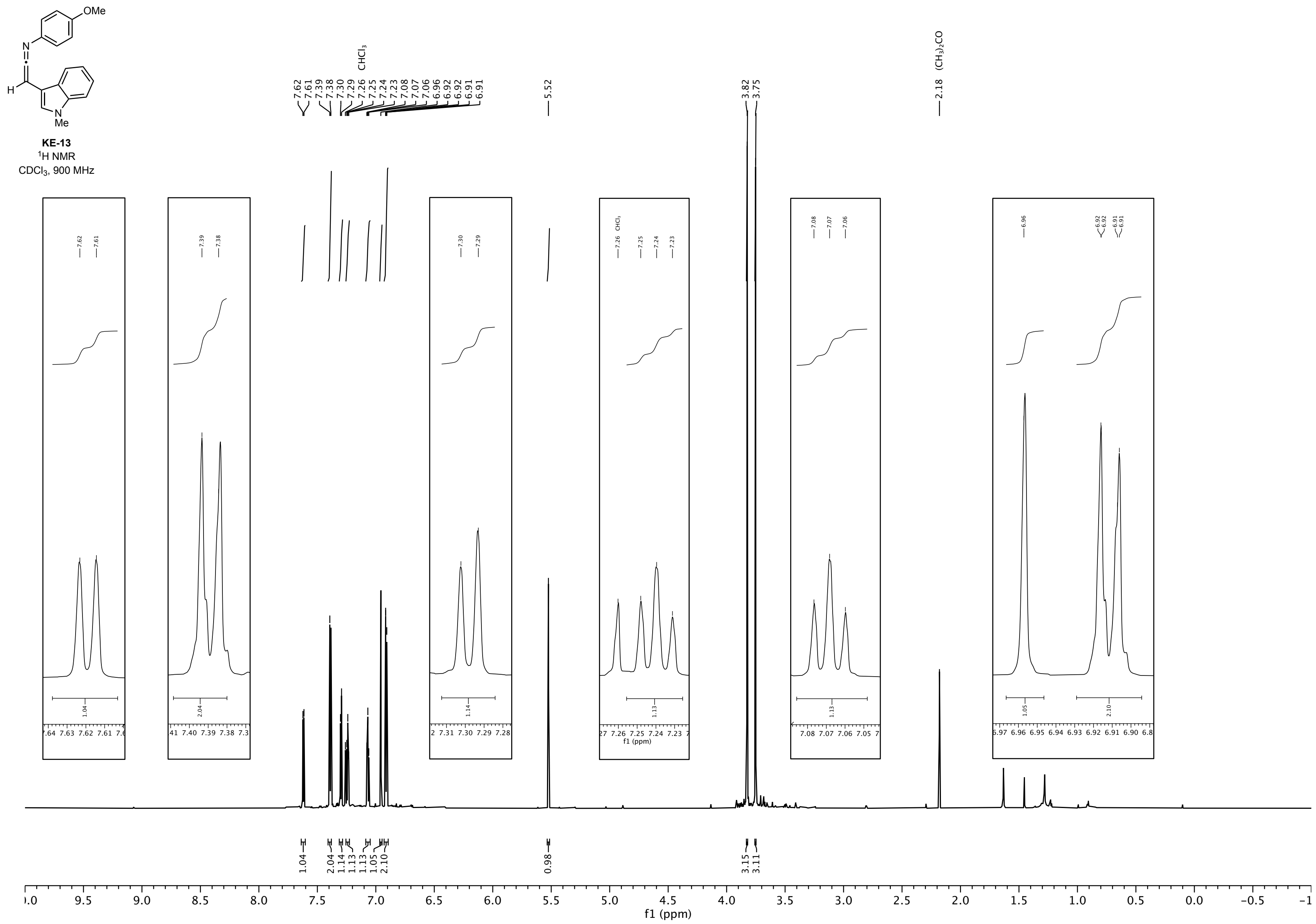
OMe
N
H
N
Me
KE-13
^{1}H NMR
$CDCl_3$, 900 MHz
7.62
7.61
7.39
7.38
7.30
7.29
7.26
7.25
7.24
7.23
7.08
7.07
7.06
6.96
6.92
6.92
6.91
6.91
CHCl3
5.52
3.82
3.75
2.18 (CH3)2CO
1.04
2.04
1.14
1.13
1.13
1.05
2.10
0.98
3.15
3.11
f1 (ppm)

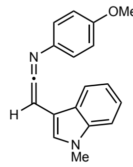

KE-13
^{13}C NMR
$CDCl_3$, 225 MHz

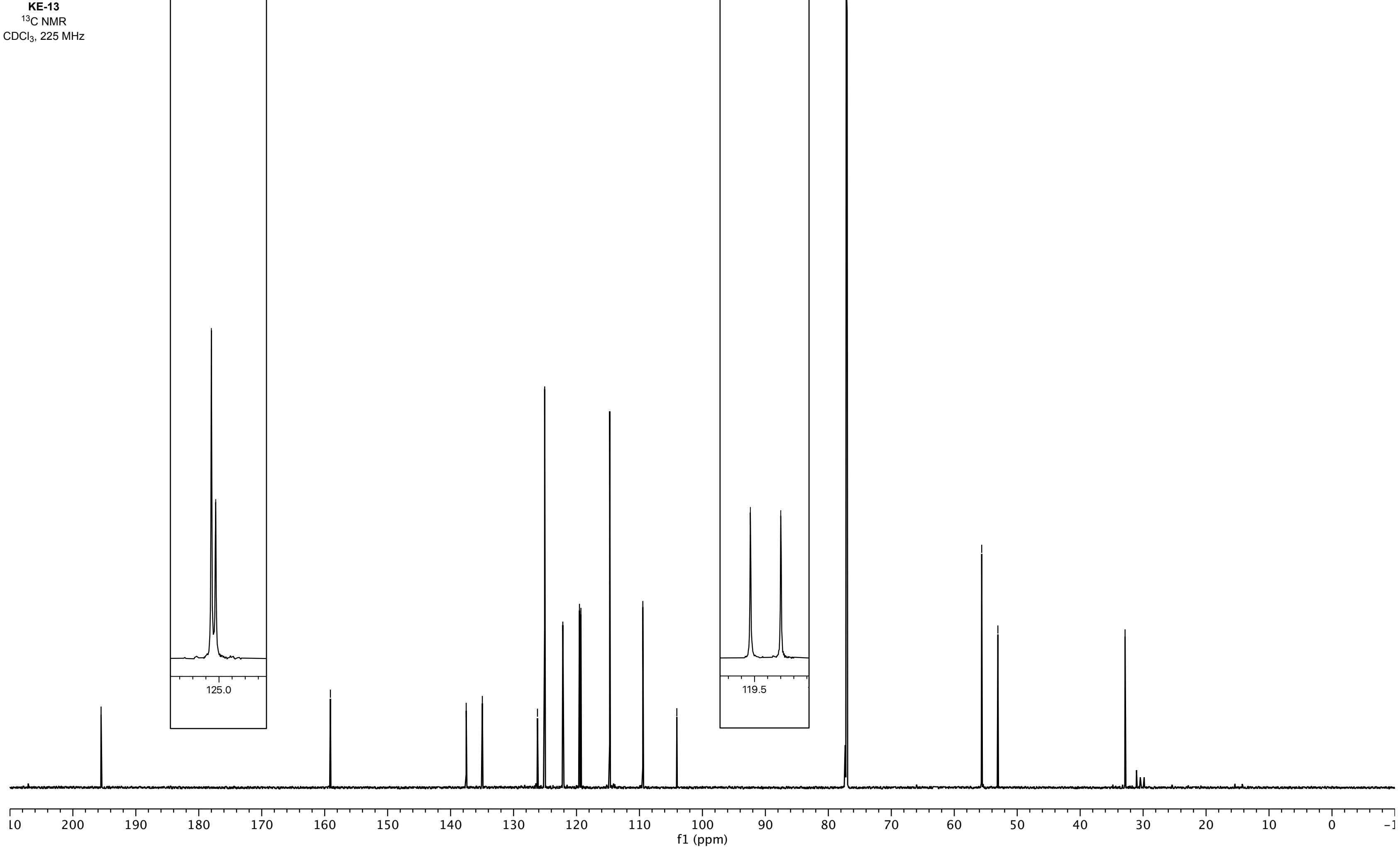

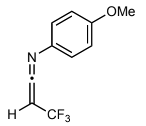

KE-14
^{1}H NMR
$CDCl_3$, 400 MHz

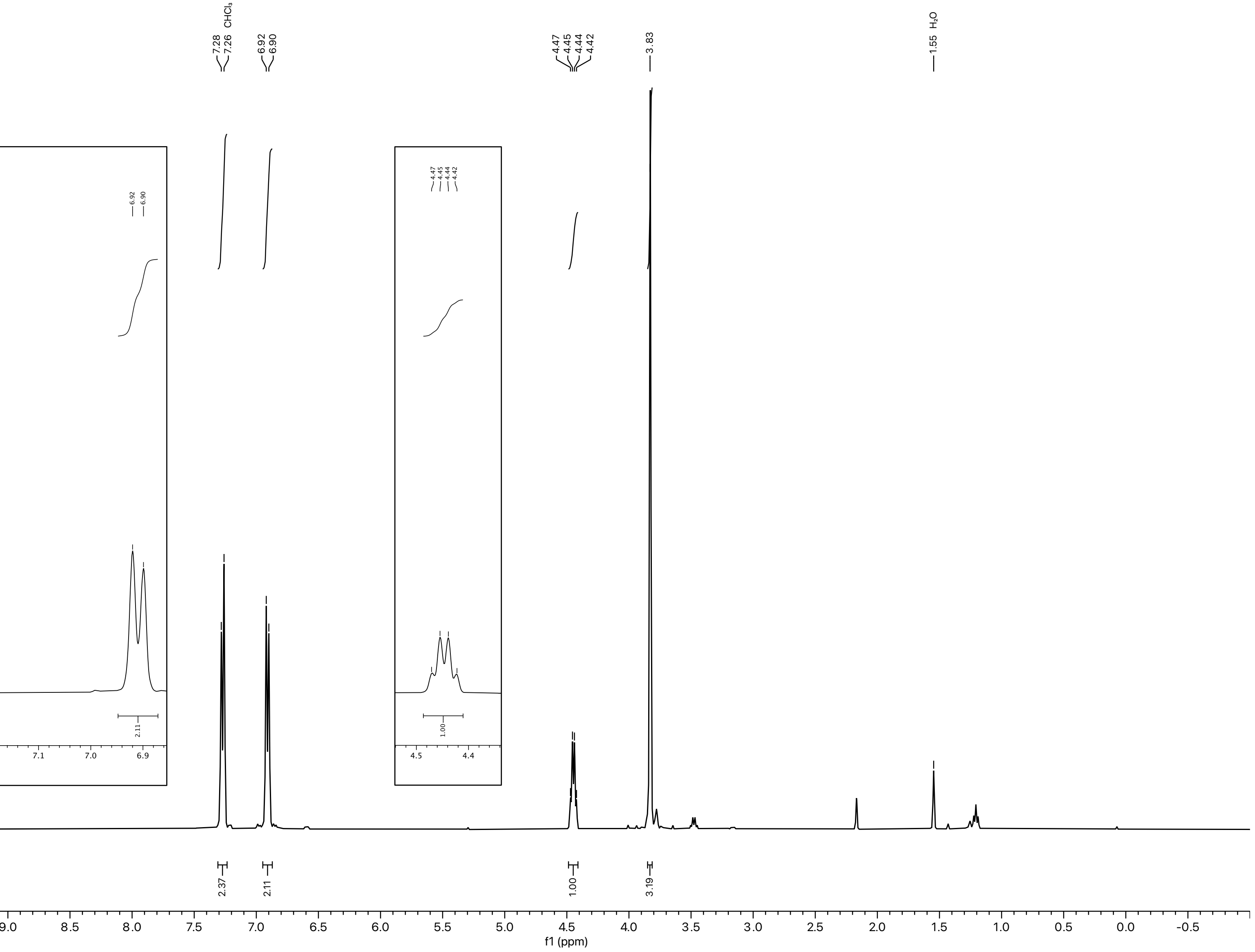

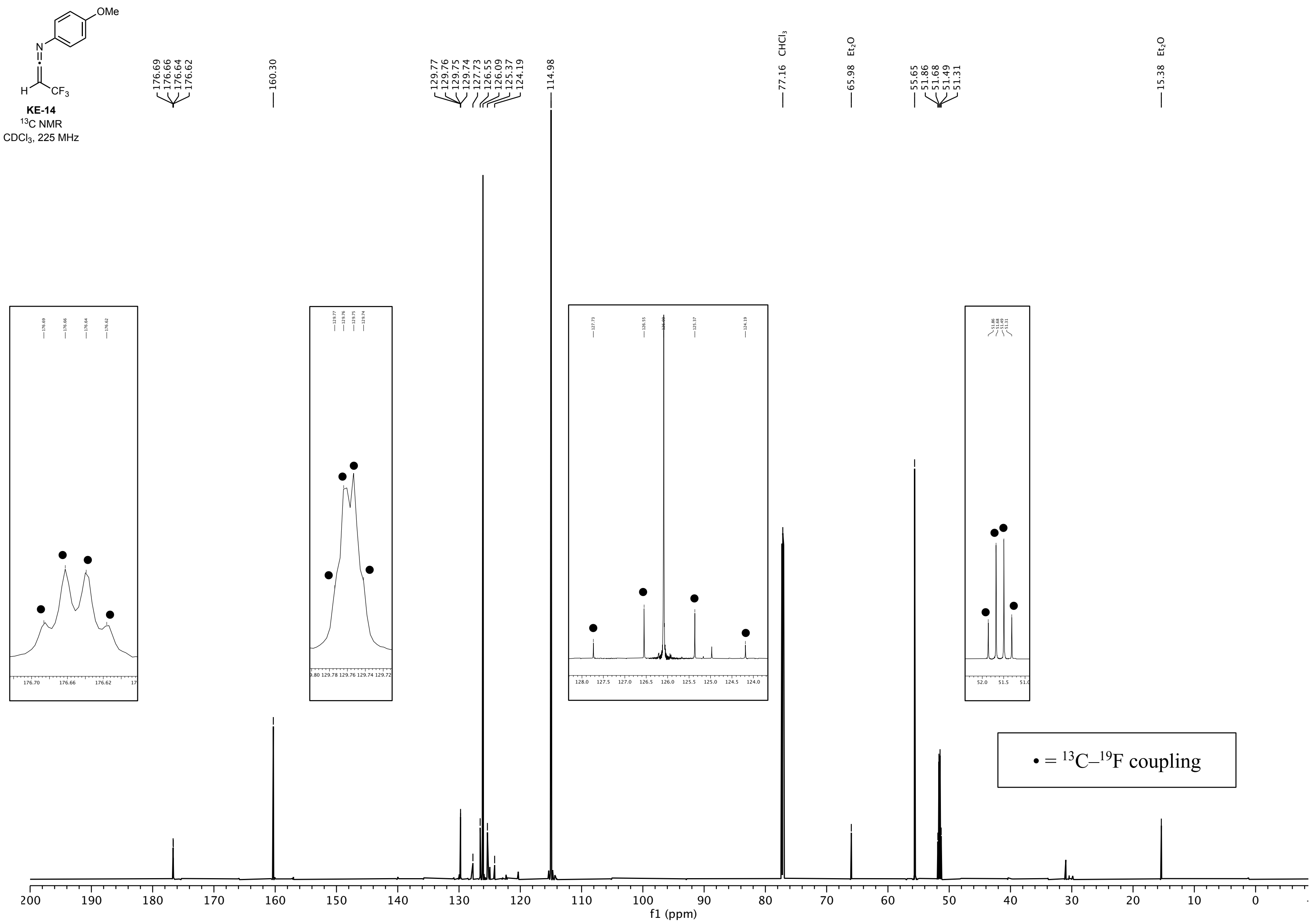

OMe
N
H
CF3
KE-14
13C NMR
CDCl3, 225 MHz
176.69
176.66
176.64
176.62
160.30
129.77
129.76
129.75
129.74
127.73
126.55
126.09
125.37
124.19
114.98
77.16 CHCl3
65.98 Et2O
55.65
51.86
51.68
51.49
51.31
15.38 Et2O
• = 13C–19F coupling
f1 (ppm)

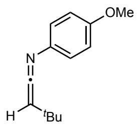

KE-15
^{1}H NMR
$CDCl_3$, 900 MHz

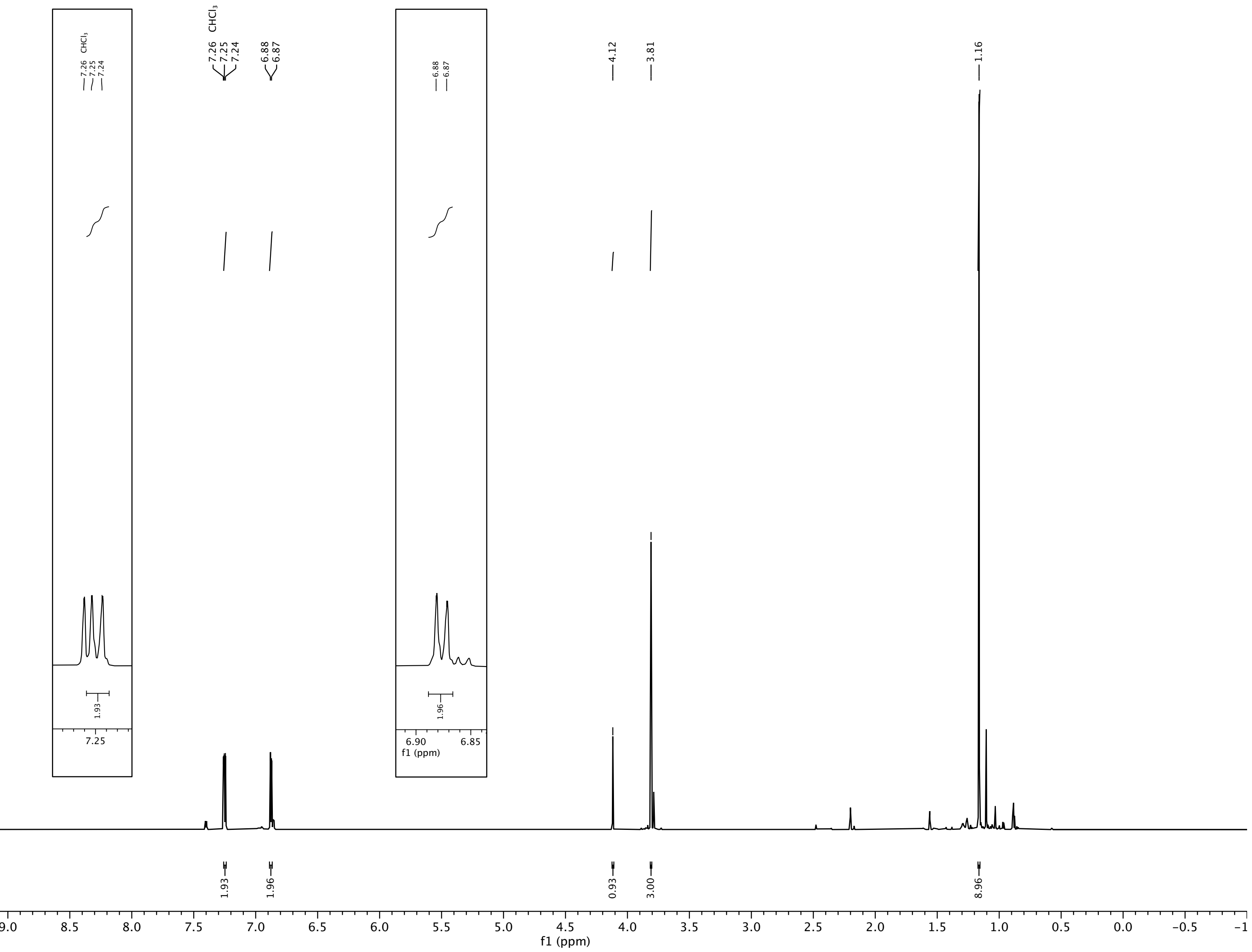

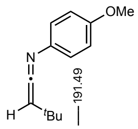

KE-15
^{13}C NMR
$CDCl_3$, 225 MHz

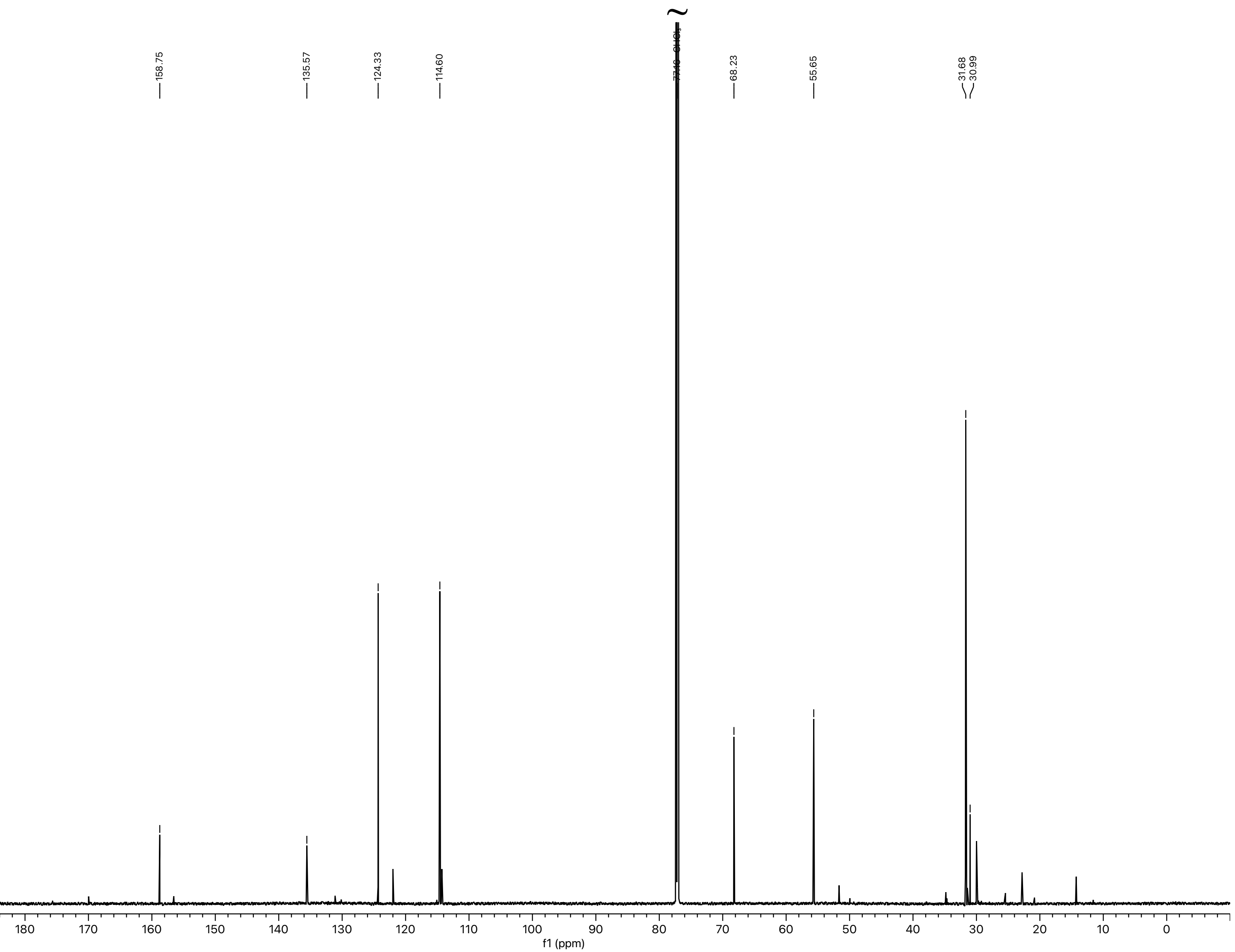

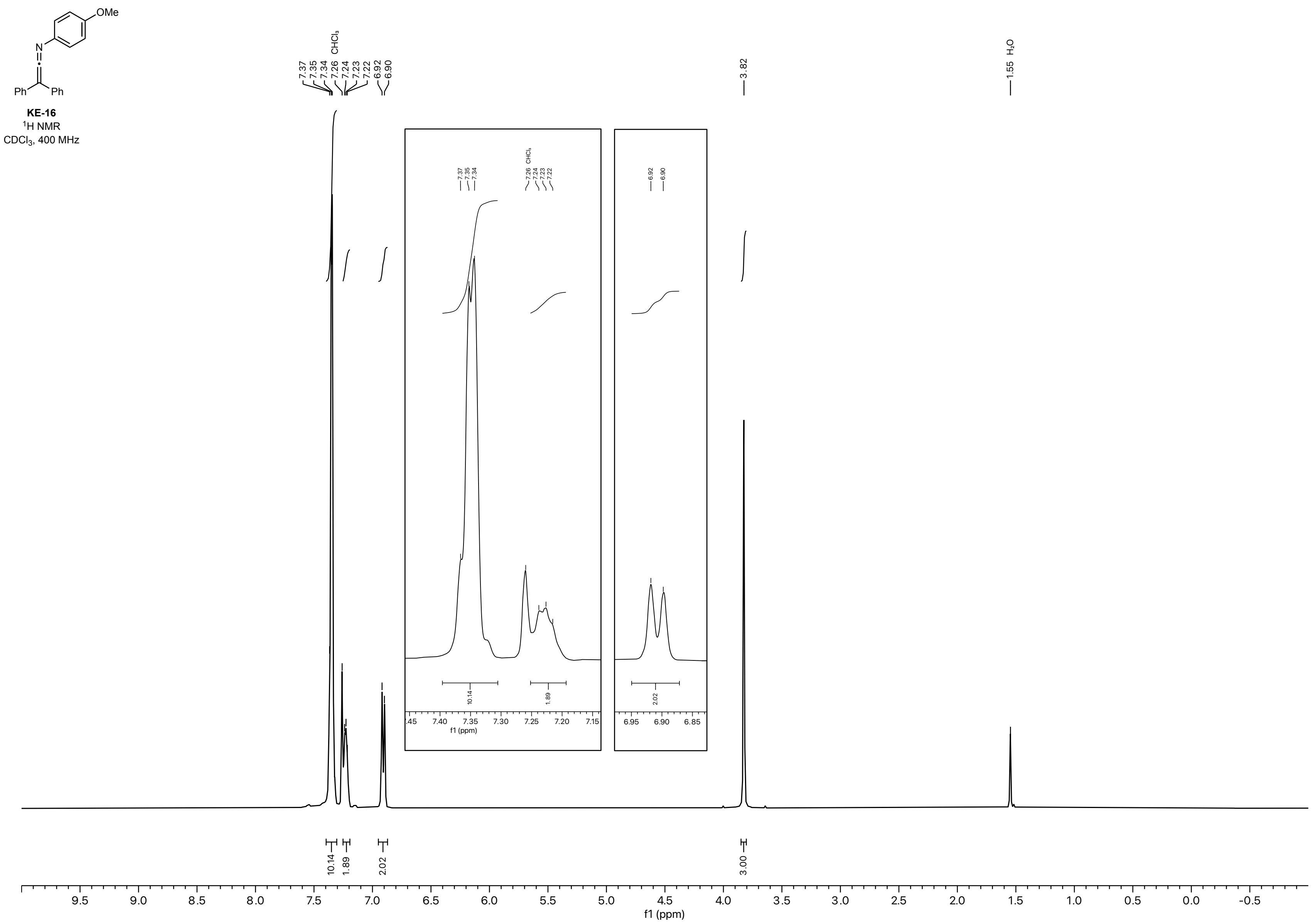

OMe
N
Ph
Ph
KE-16
^{1}H NMR
$CDCl_3$, 400 MHz
7.37
7.35
7.34
7.26 $CHCl_3$
7.24
7.23
7.22
6.92
6.90
3.82
1.55 H_2O
10.14
1.89
2.02
3.00
f1 (ppm)

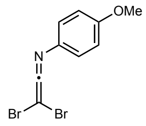

KE-17
^{1}H NMR
$CDCl_3$, 600 MHz

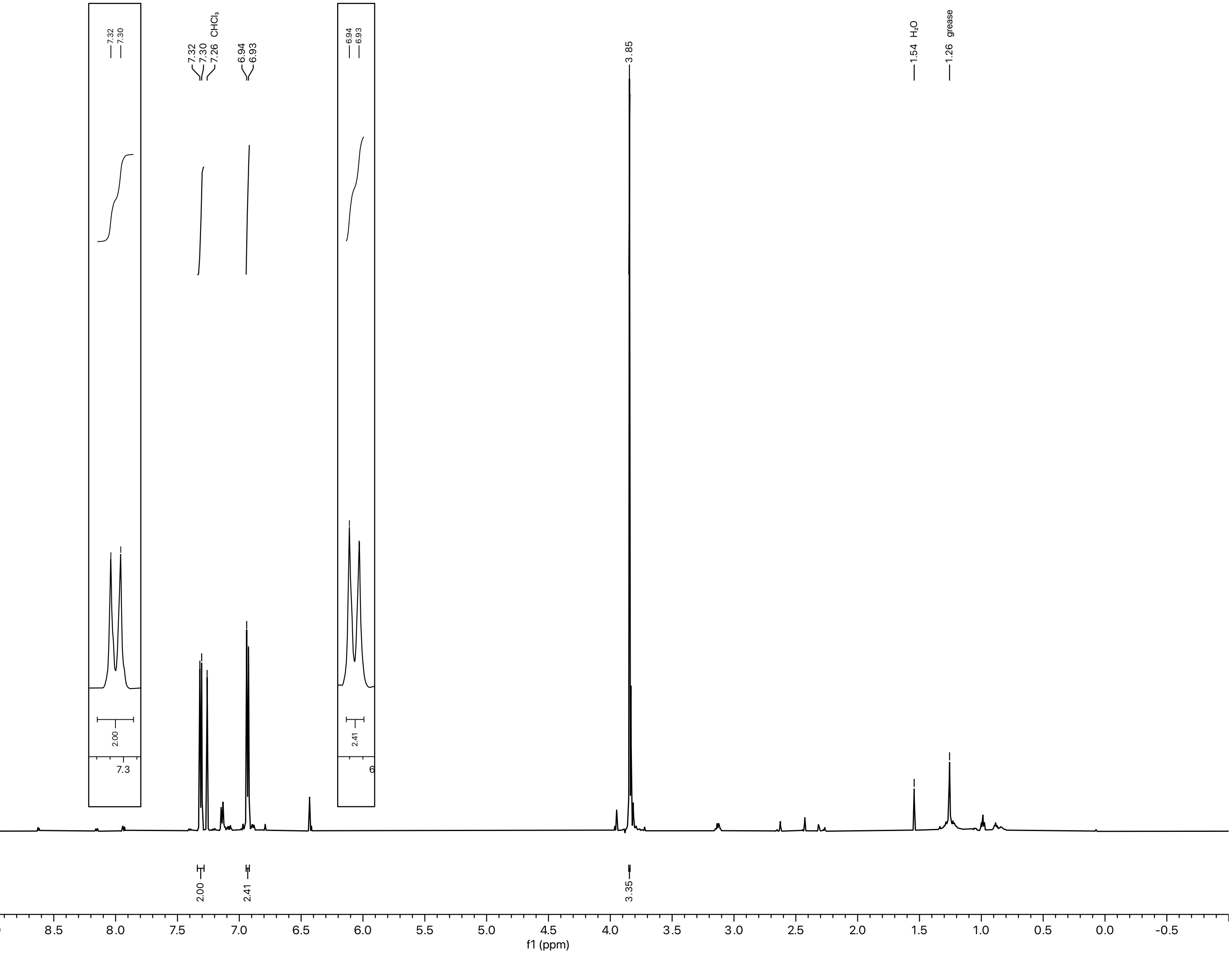

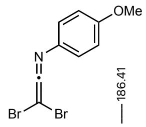

KE-17
^{13}C NMR
$CDCl_3$, 100 MHz

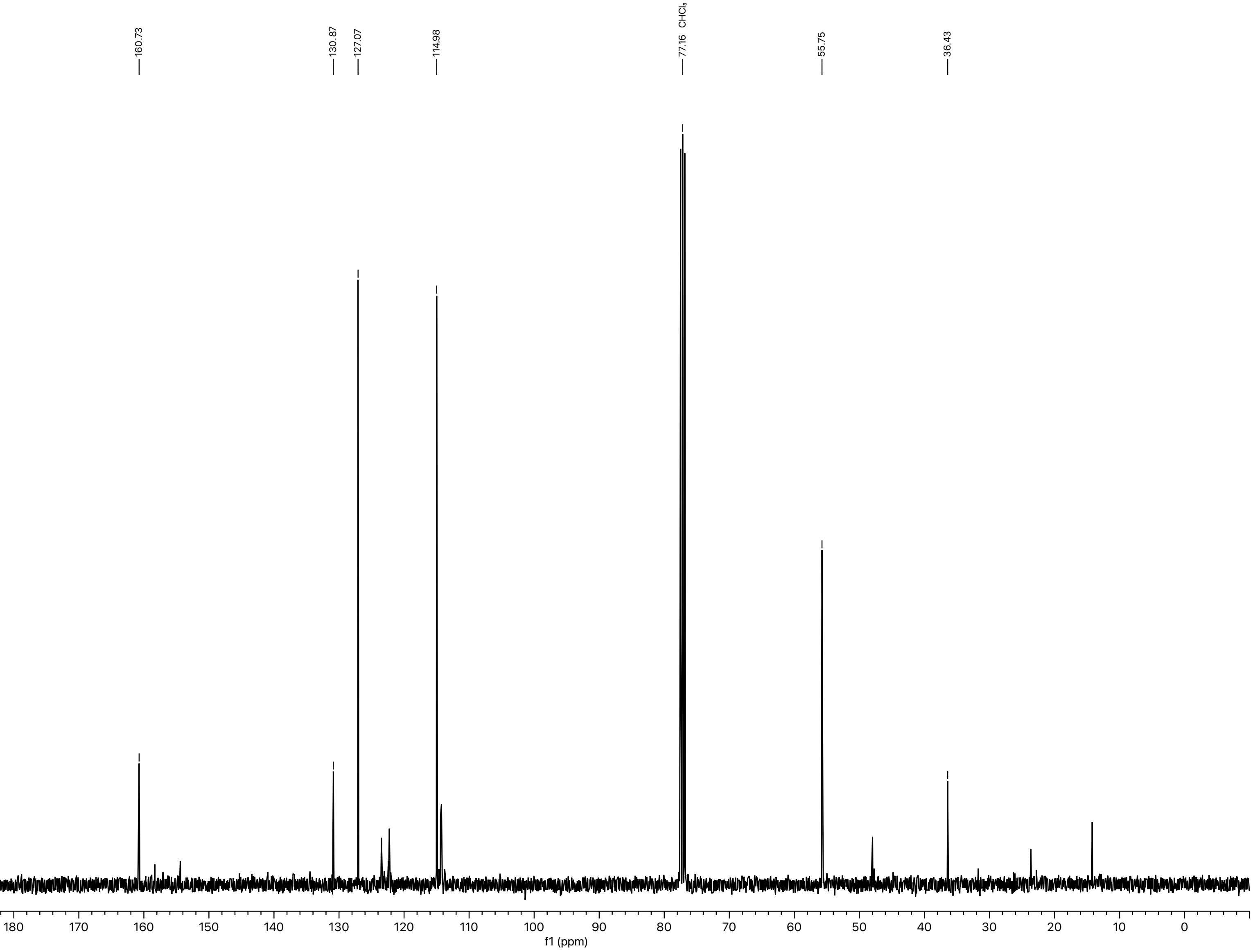

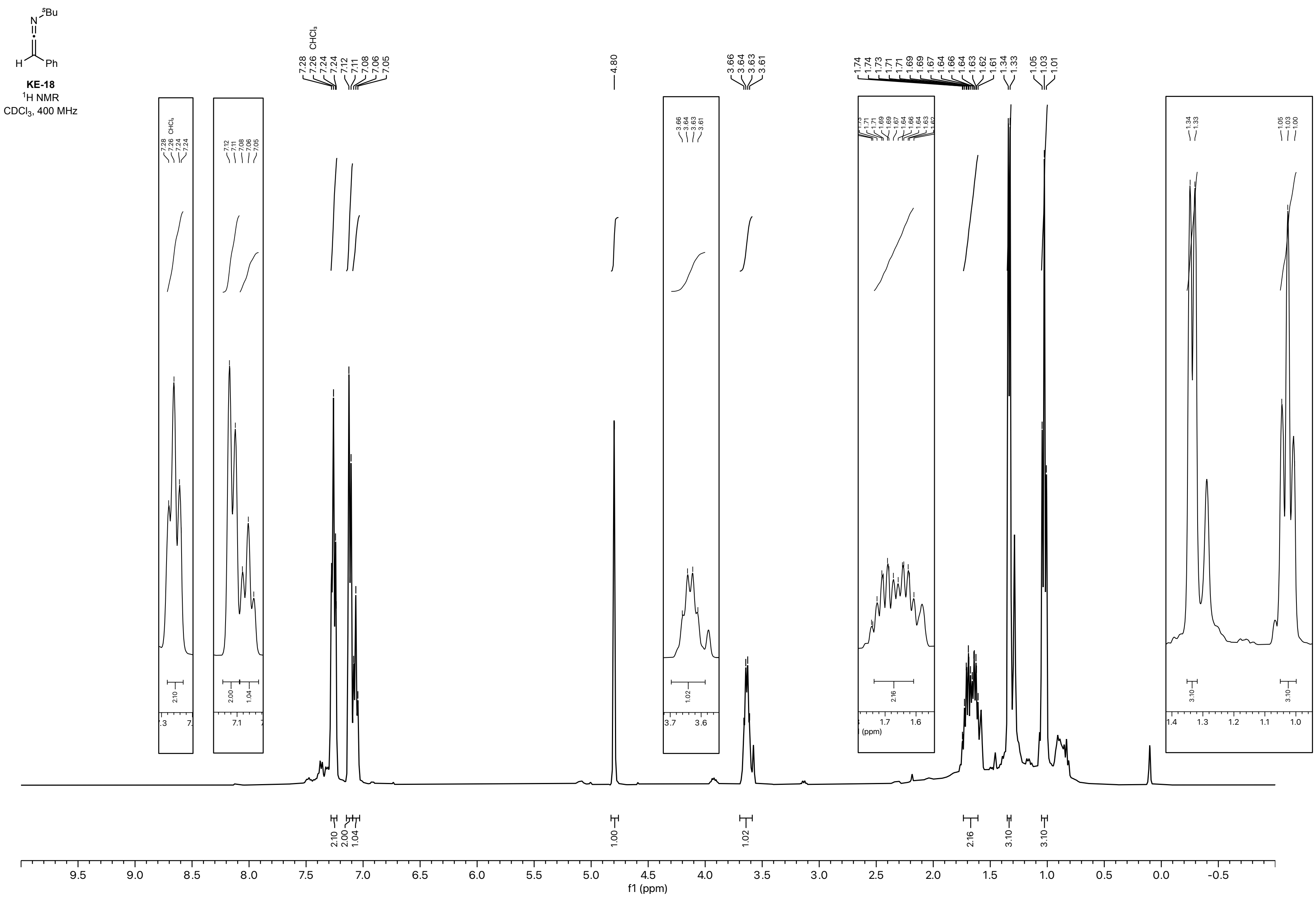
KE-18
^{1}H NMR
$CDCl_3$, 400 MHz
7.28 7.26 CHCl3 7.24 7.24 7.12 7.11 7.08 7.06 7.05
4.80
3.66 3.64 3.63 3.61
1.74 1.74 1.73 1.71 1.71 1.69 1.69 1.67 1.64 1.66 1.64 1.63 1.62 1.61 1.34 1.33
1.05 1.03 1.01
2.10 2.00 1.04 1.00 1.02 2.16 3.10 3.10
f1 (ppm)

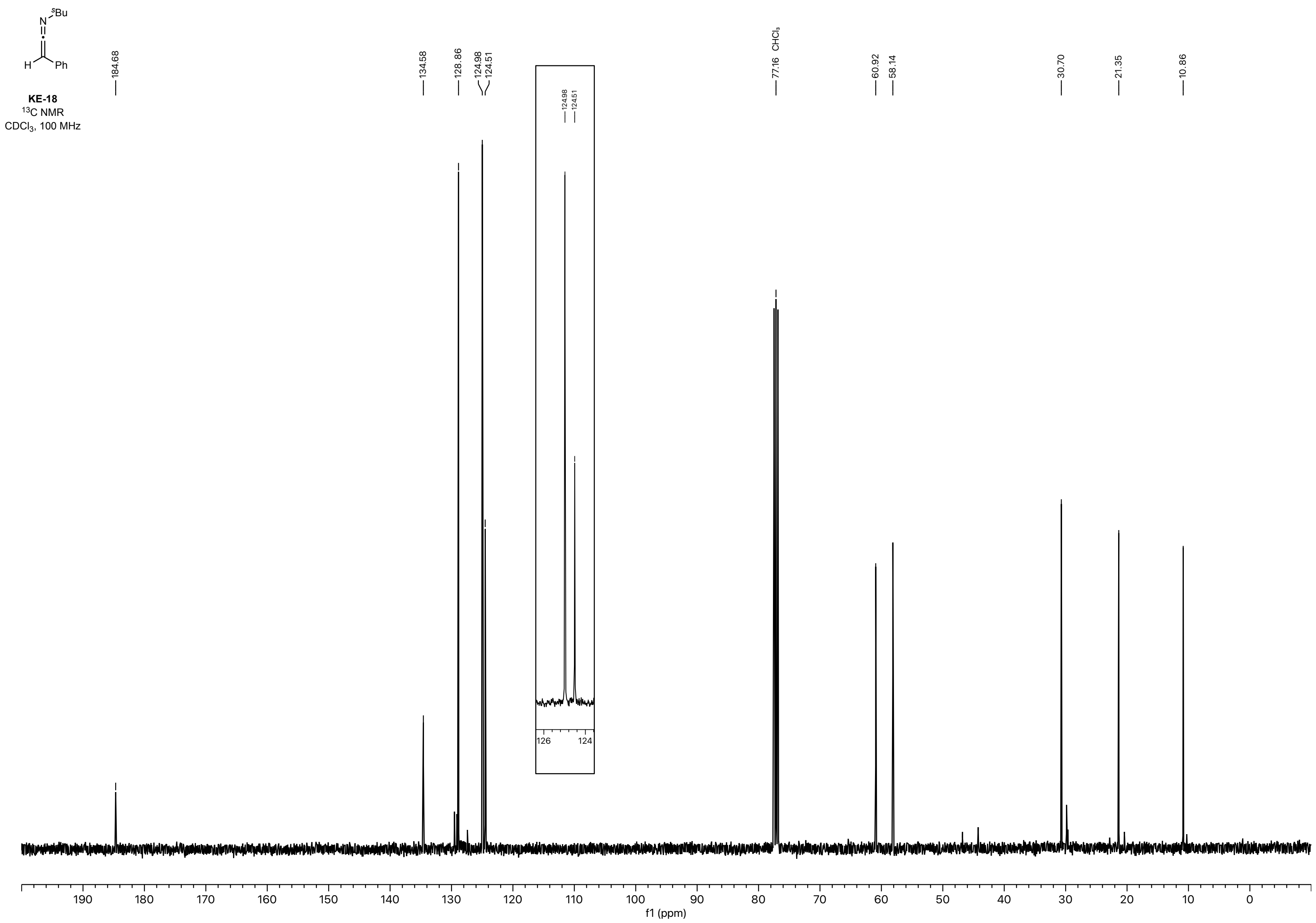
KE-18
^{13}C NMR
$CDCl_3$, 100 MHz
184.68
134.58
128.86
124.98
124.51
77.16 $CHCl_3$
60.92
58.14
30.70
21.35
10.86
f1 (ppm)

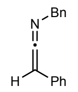

KE-19
^{1}H NMR
$CDCl_3$, 400 MHz

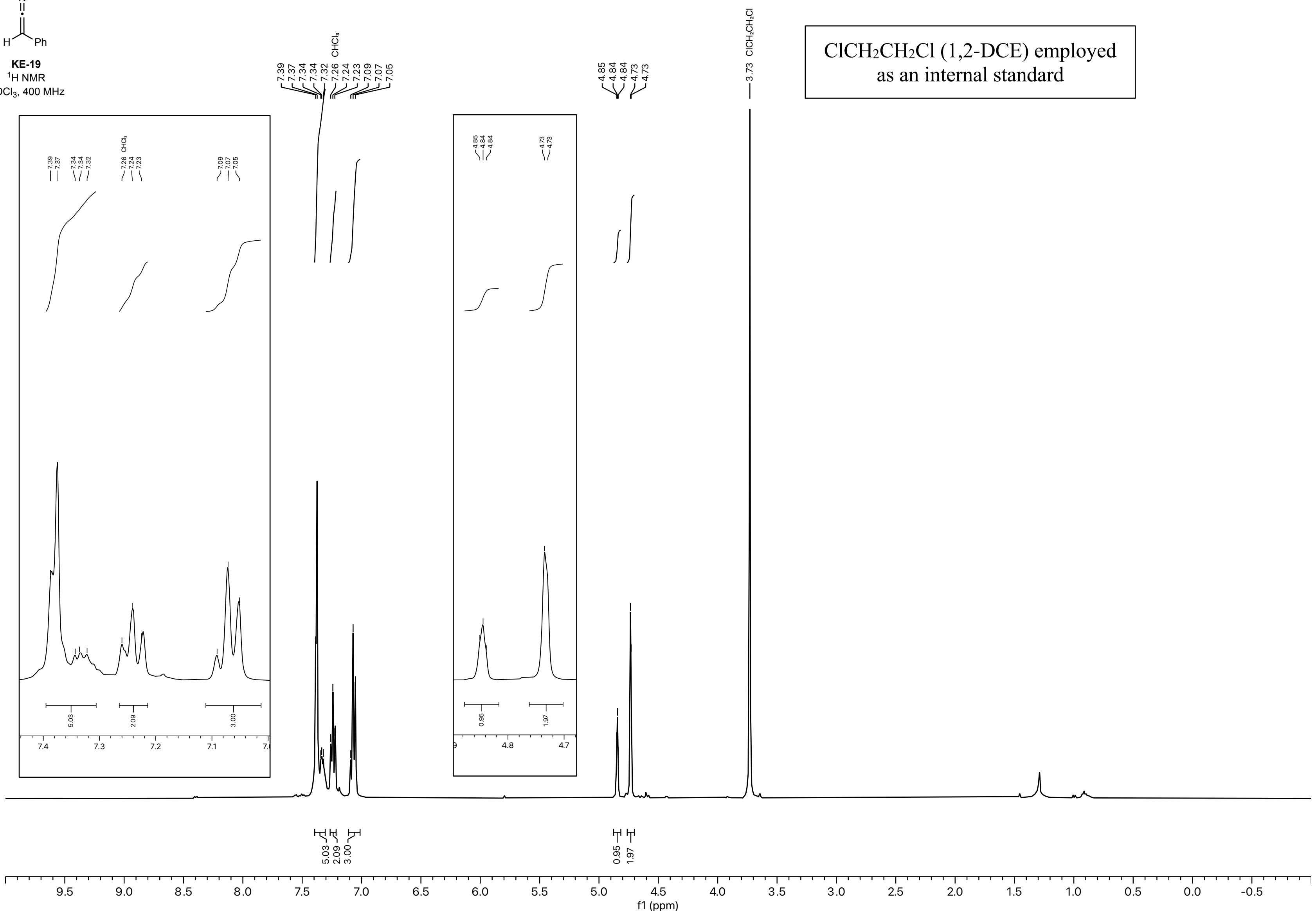

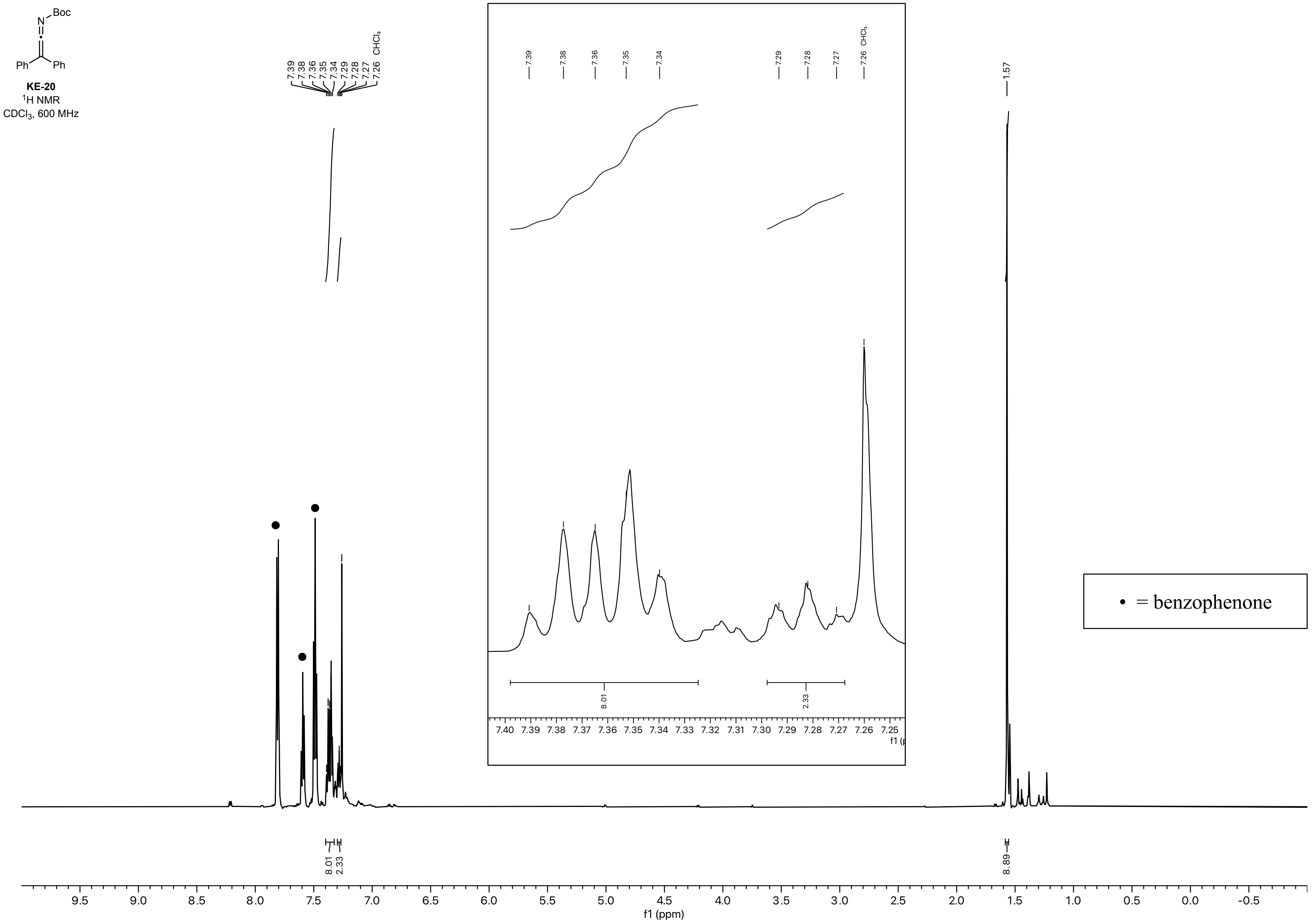

KE-20
^{1}H NMR
$CDCl_3$, 600 MHz
7.39 7.38 7.36 7.35 7.34 7.29 7.28 7.27 7.26 $CHCl_3$
1.57
8.01
2.33
8.89
• = benzophenone
f1 (ppm)

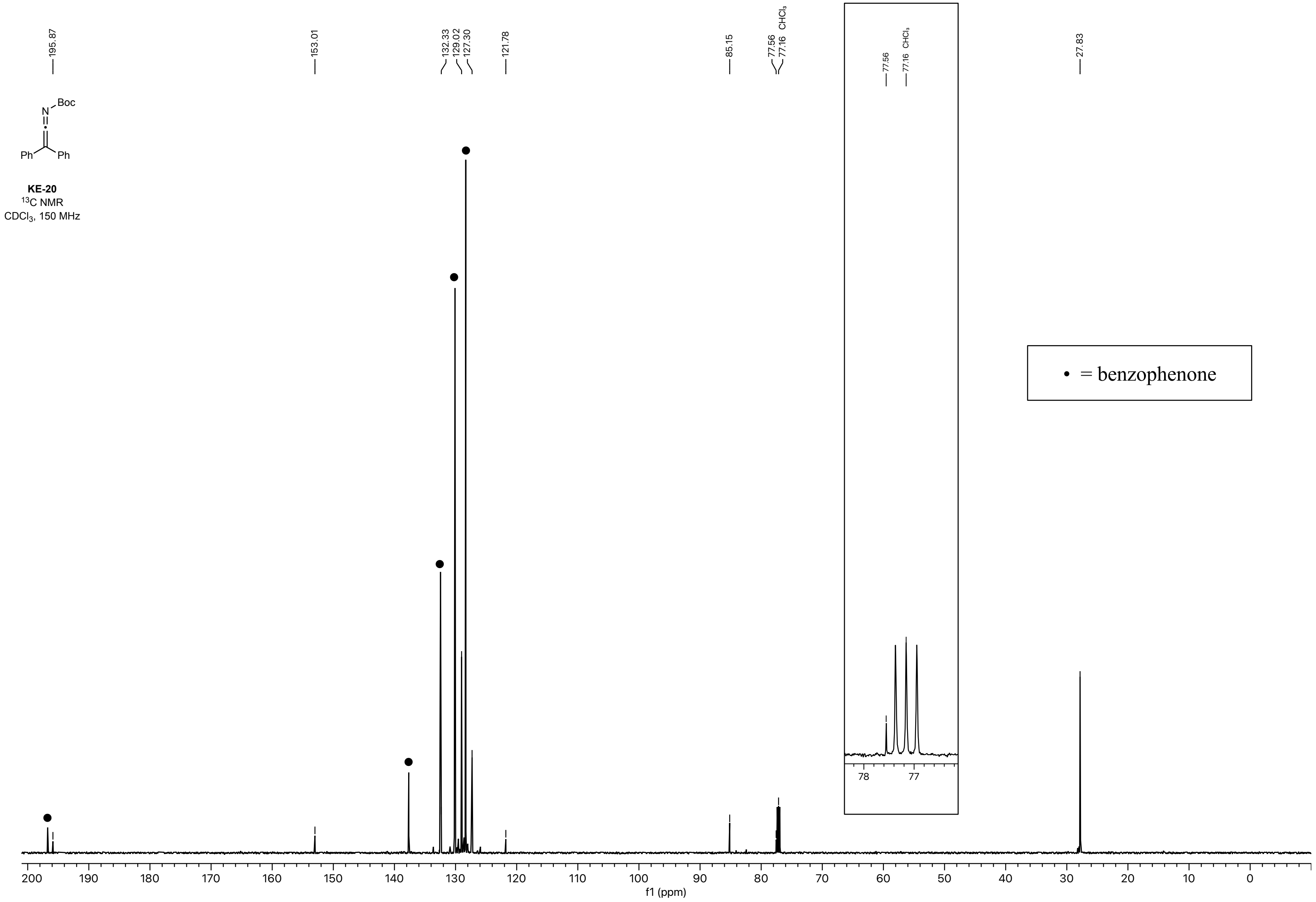
195.87
153.01
132.33
129.02
127.30
121.78
85.15
77.56
77.16 CHCl3
27.83
N Boc
Ph Ph
KE-20
13C NMR
CDCl3, 150 MHz
• = benzophenone
78
77
200 190 180 170 160 150 140 130 120 110 100 90 80 70 60 50 40 30 20 10 0
f1 (ppm)

Bis(silyloxy)furan + Ketenimine Aza-Diels–Alder Reactions

Ring-Opened Products

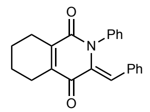

3a

^1H NMR

$CDCl_3$, 400 MHz

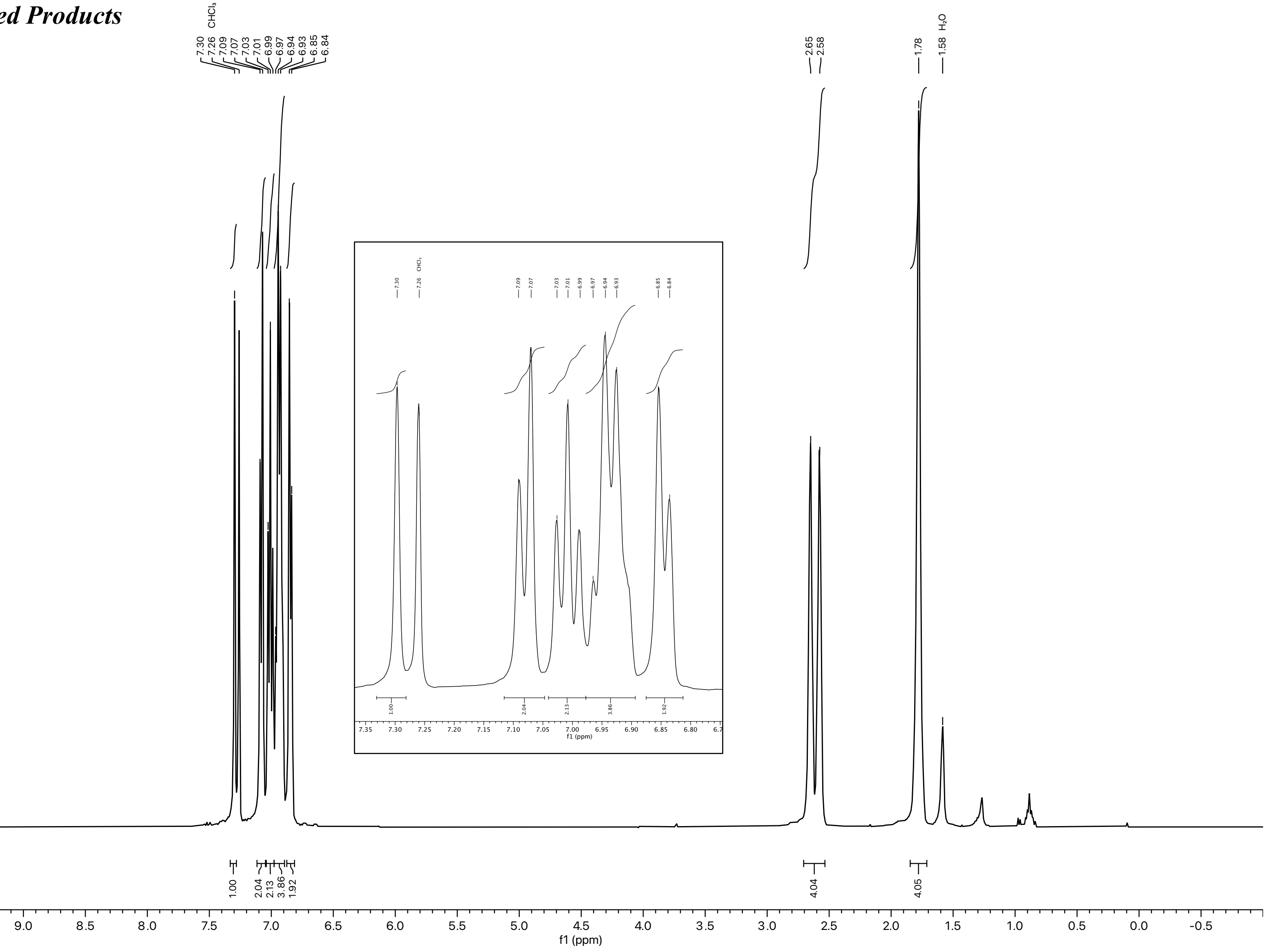

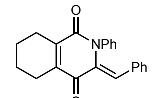

3a
^{13}C NMR
$CDCl_3$, 100 MHz

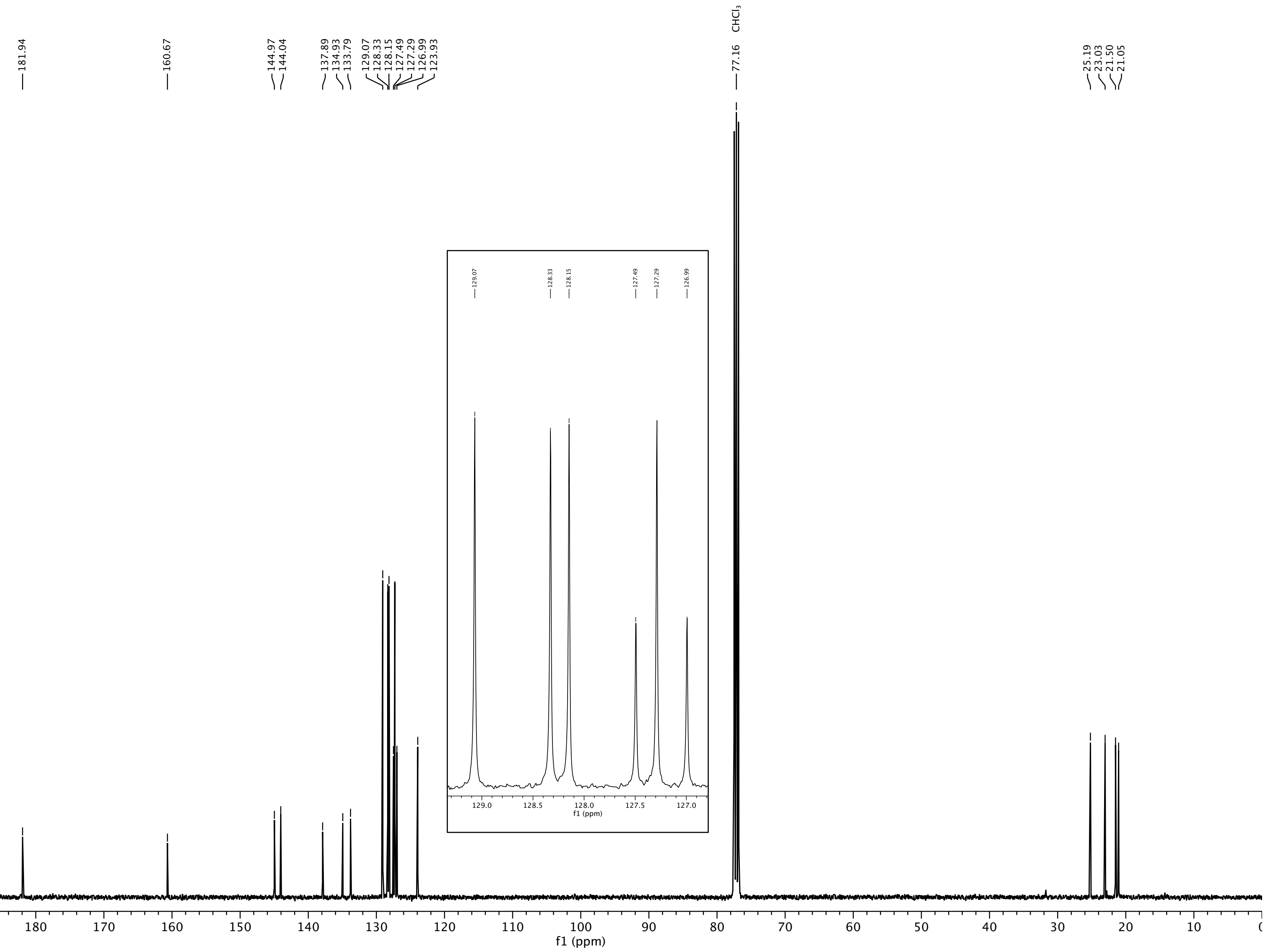

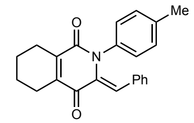

3b

^{1}H NMR

$CDCl_3$, 400 MHz

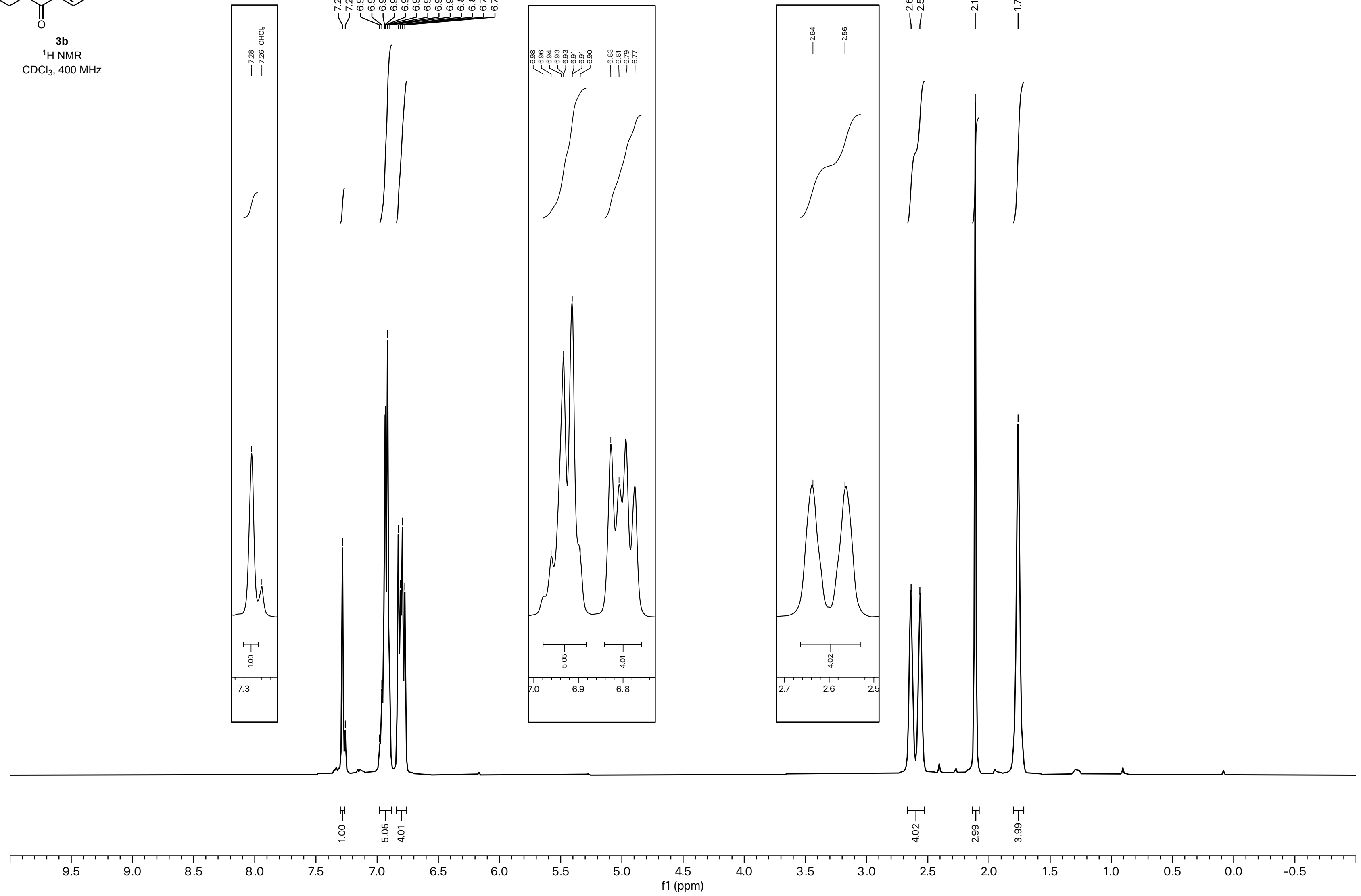

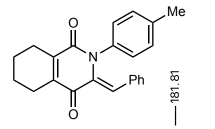

3b

^{13}C NMR

$CDCl_3$, 100 MHz

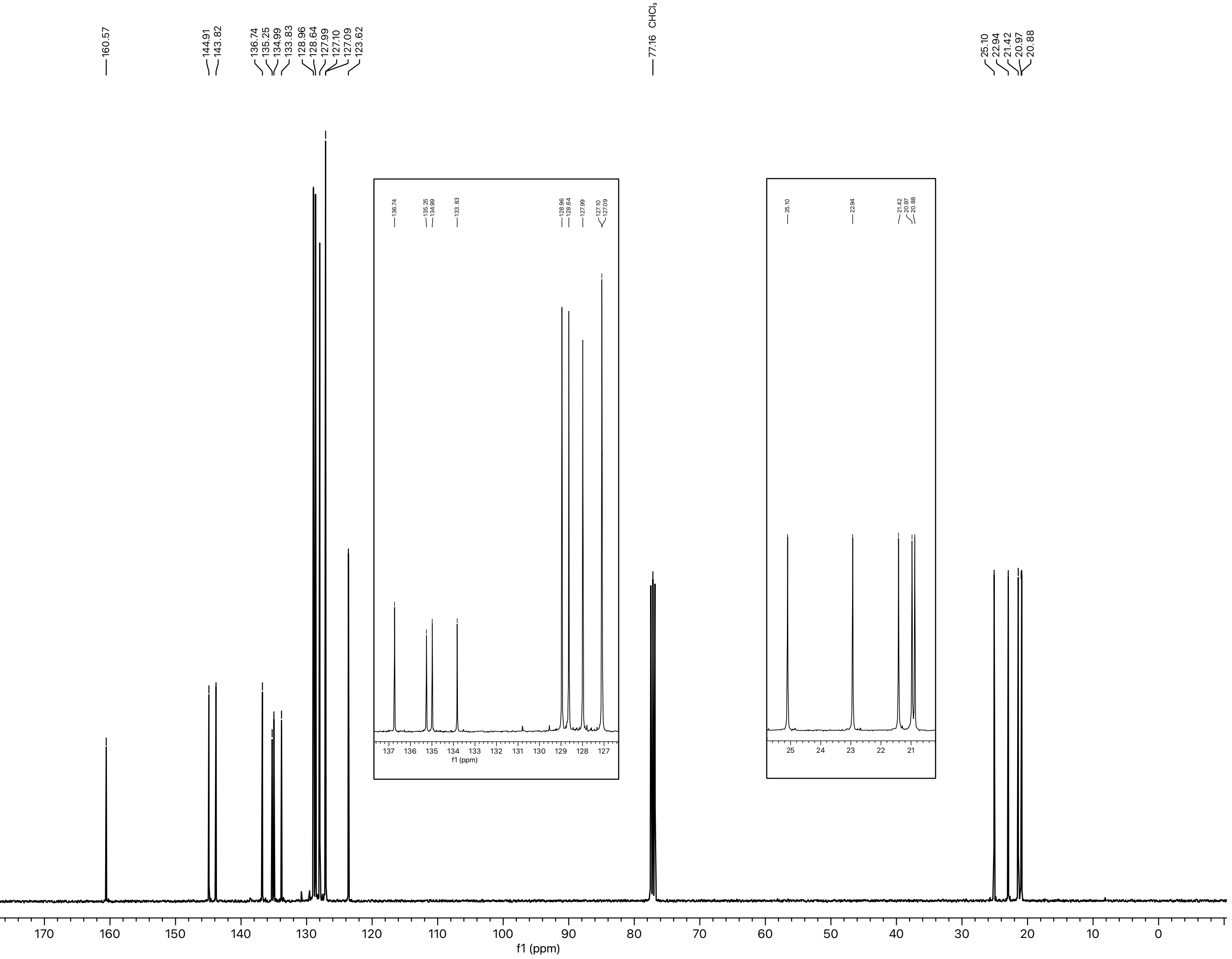

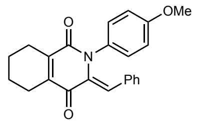

3c
^{1}H NMR
$CDCl_3$, 400 MHz

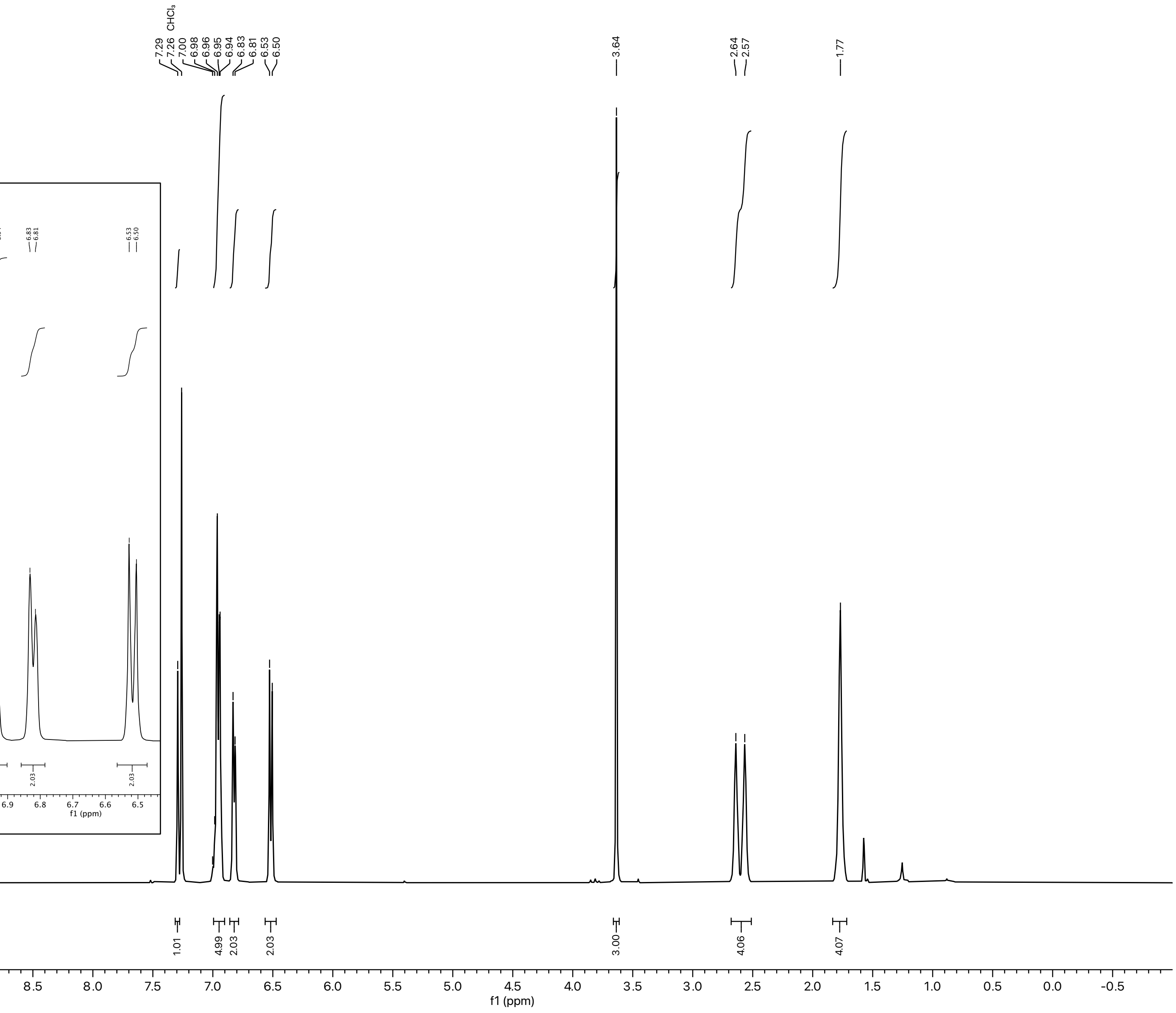

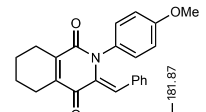

3c

^{13}C NMR

$CDCl_3$, 100 MHz

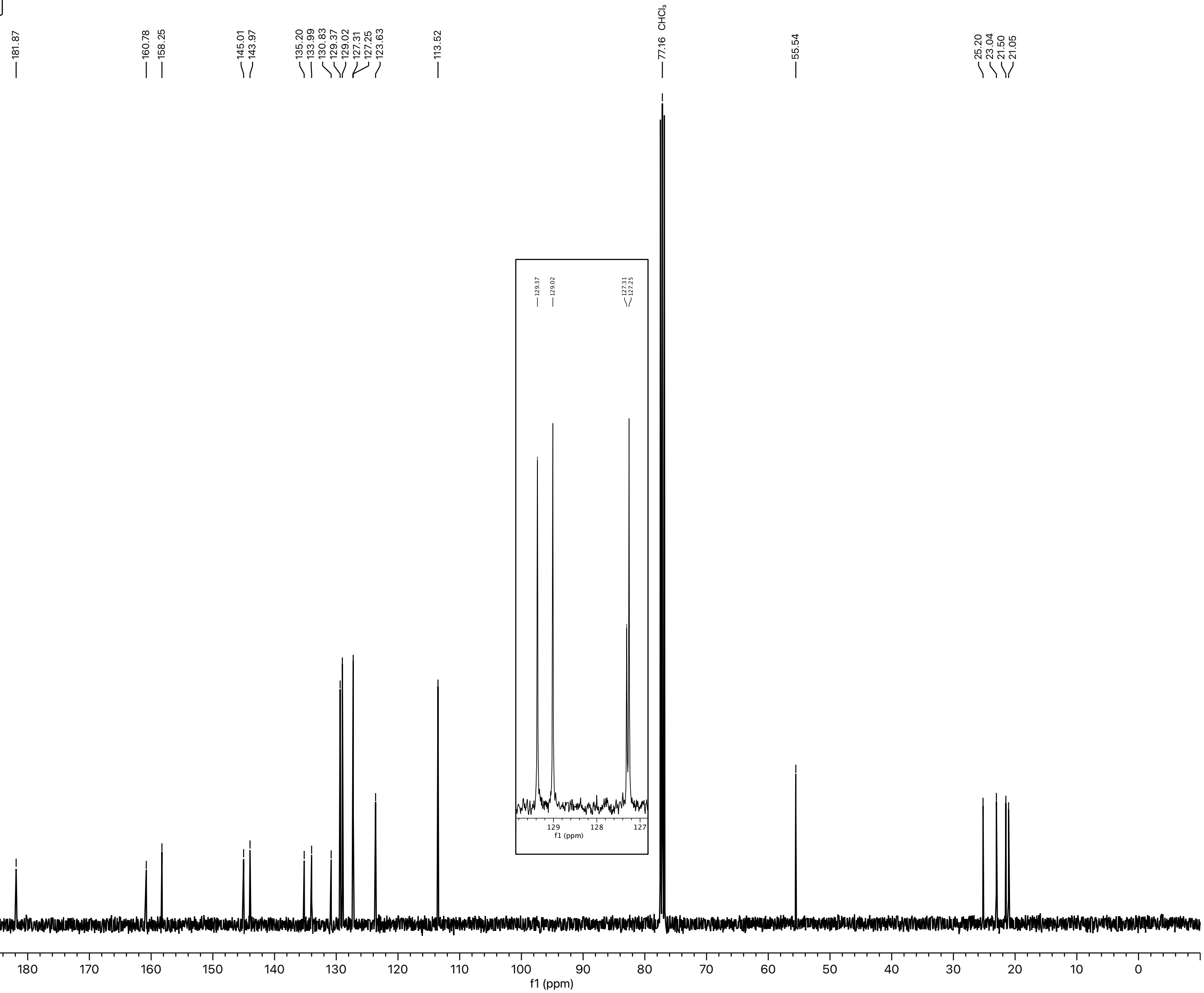

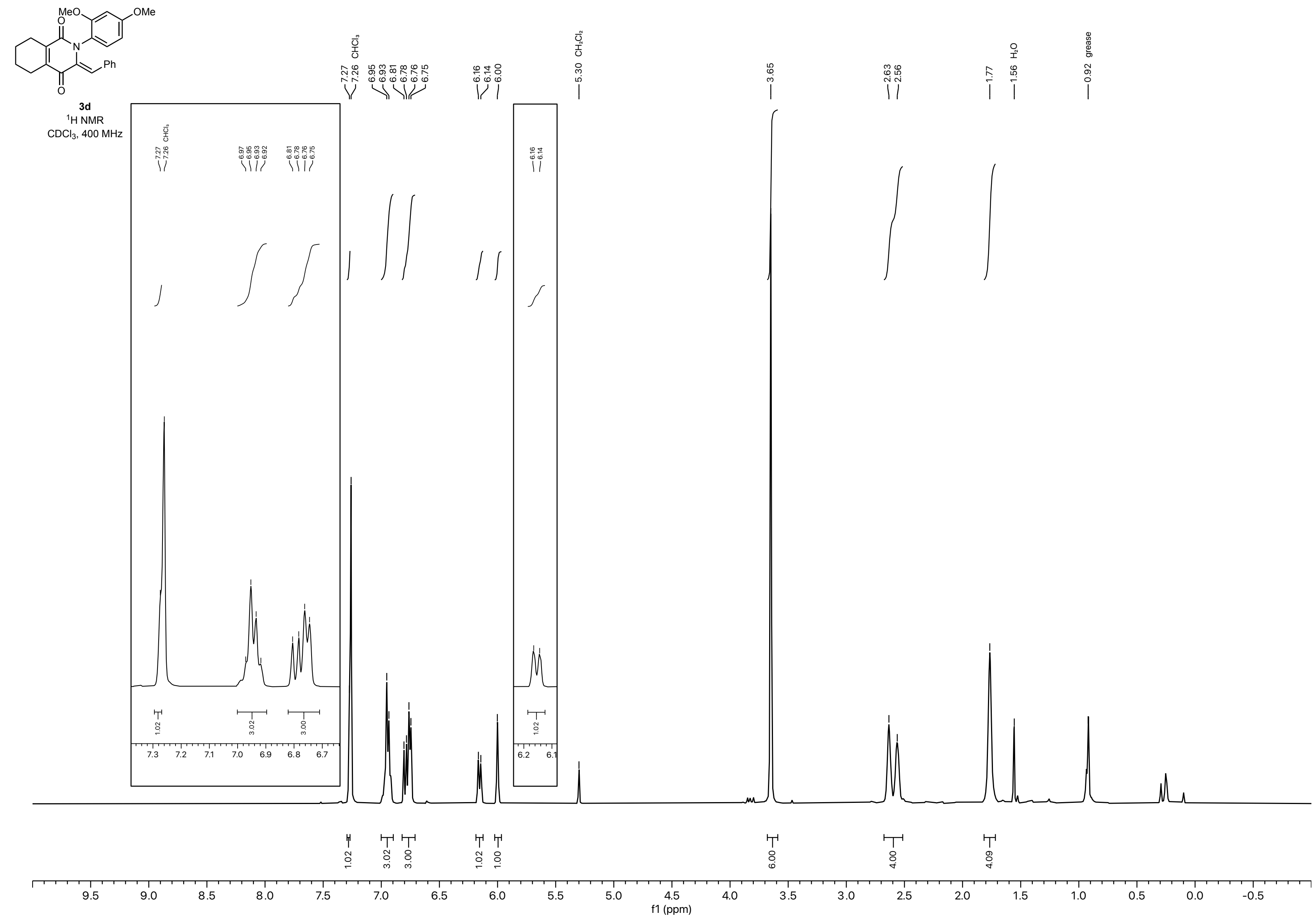
MeO
OMe
N
Ph
O
O
3d
^{1}H NMR
CDCl$_3$, 400 MHz
7.27
7.26 CHCl$_3$
6.95
6.93
6.81
6.78
6.76
6.75
6.16
6.14
6.00
5.30 CH$_2$Cl$_2$
3.65
2.63
2.56
1.77
1.56 H$_2$O
0.92 grease
1.02
3.02
3.00
1.02
1.00
6.00
4.00
4.09
f1 (ppm)

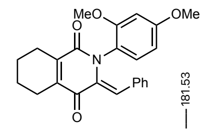

3d

^{13}C NMR

$CDCl_3$, 100 MHz

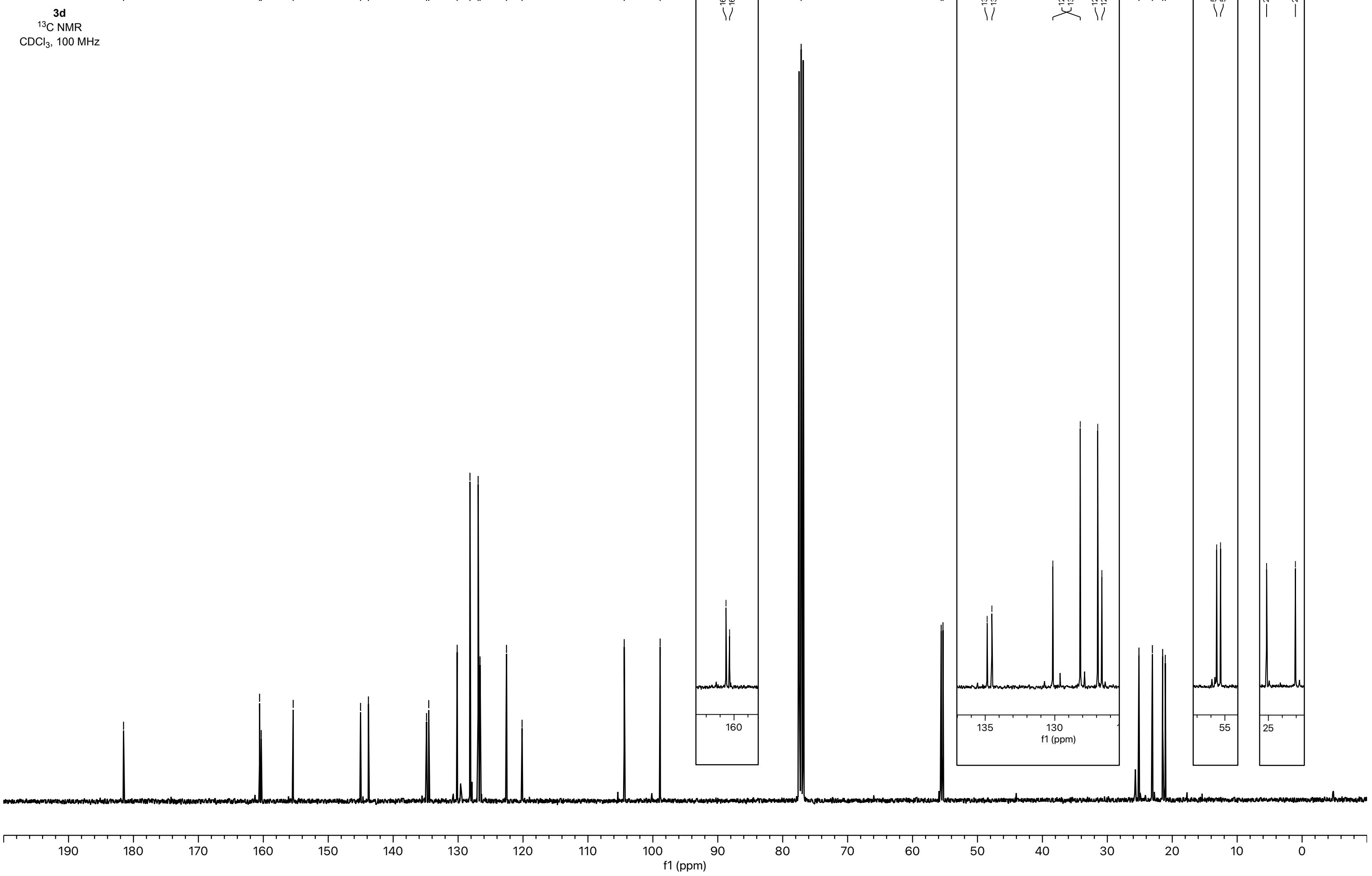

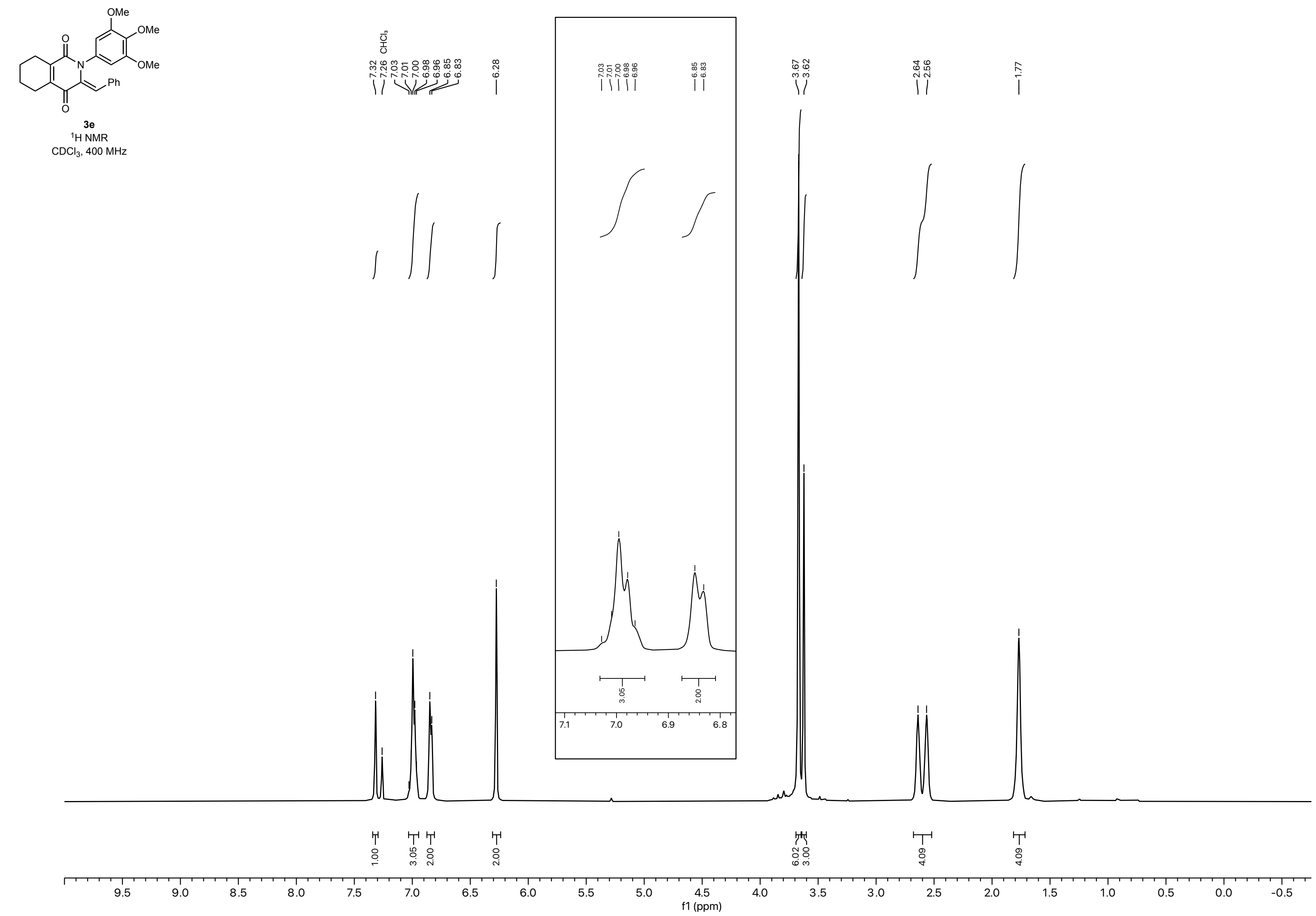

OMe
OMe
OMe
O
N
Ph
O
3e
^{1}H NMR
$CDCl_3$, 400 MHz
7.32
7.26 $CHCl_3$
7.03
7.01
7.00
6.98
6.96
6.85
6.83
6.28
3.67
3.62
2.64
2.56
1.77
1.00
3.05
2.00
2.00
6.02
3.00
4.09
4.09
f1 (ppm)

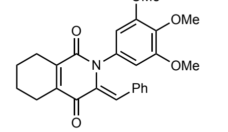

3e
^{13}C NMR
$CDCl_3$, 100 MHz

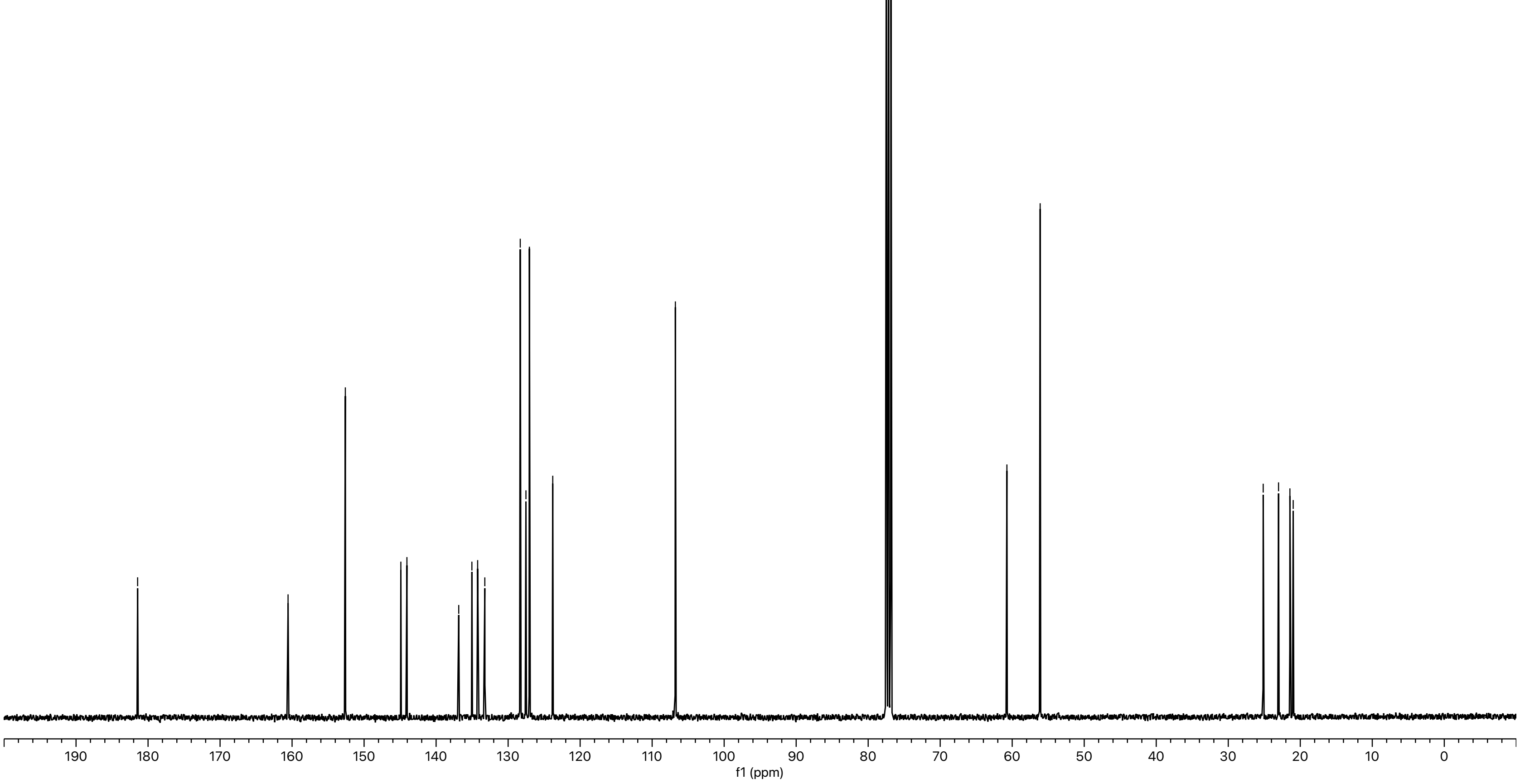

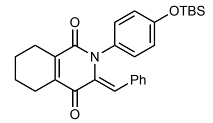

3f

^1H NMR

$CDCl_3$, 600 MHz

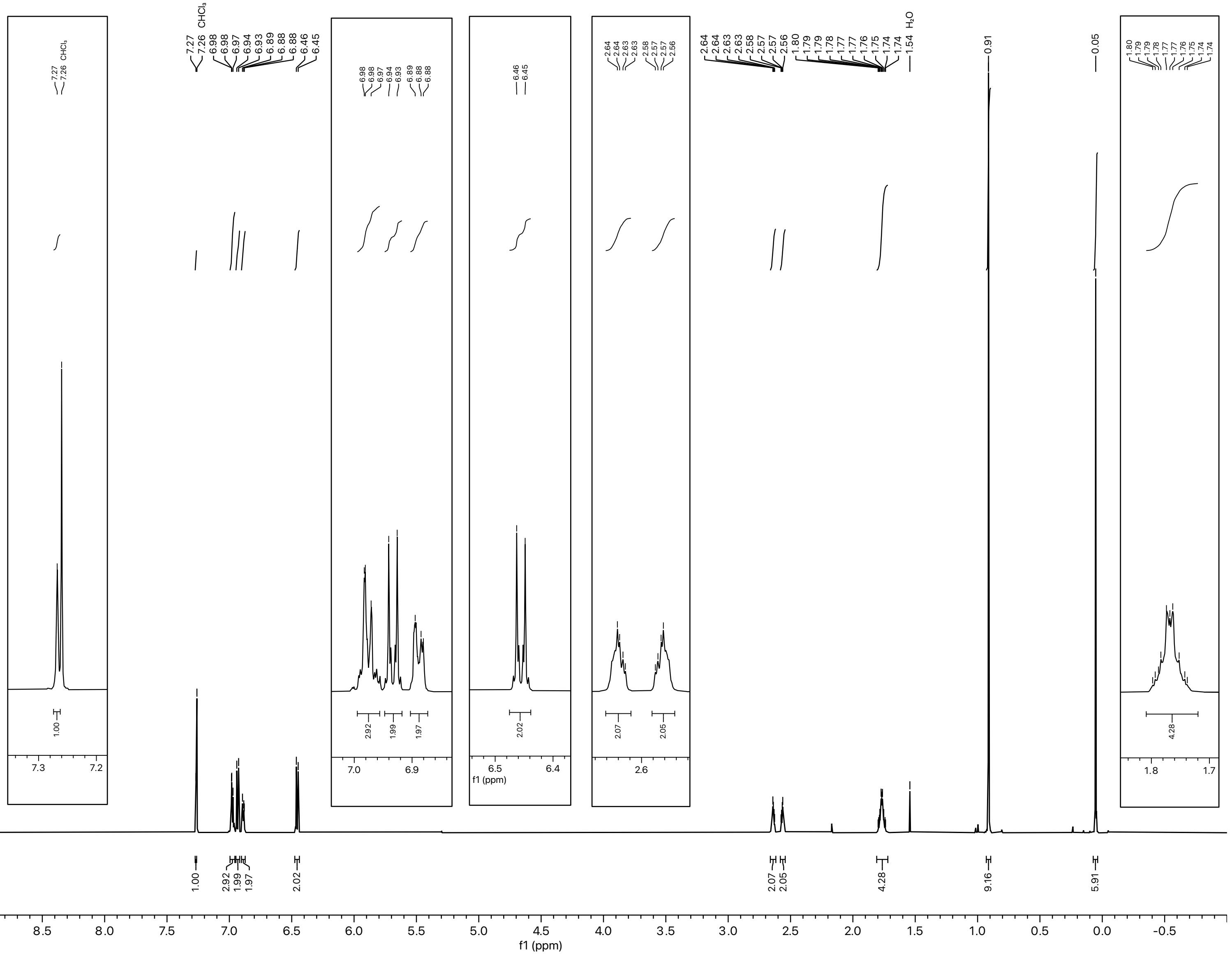

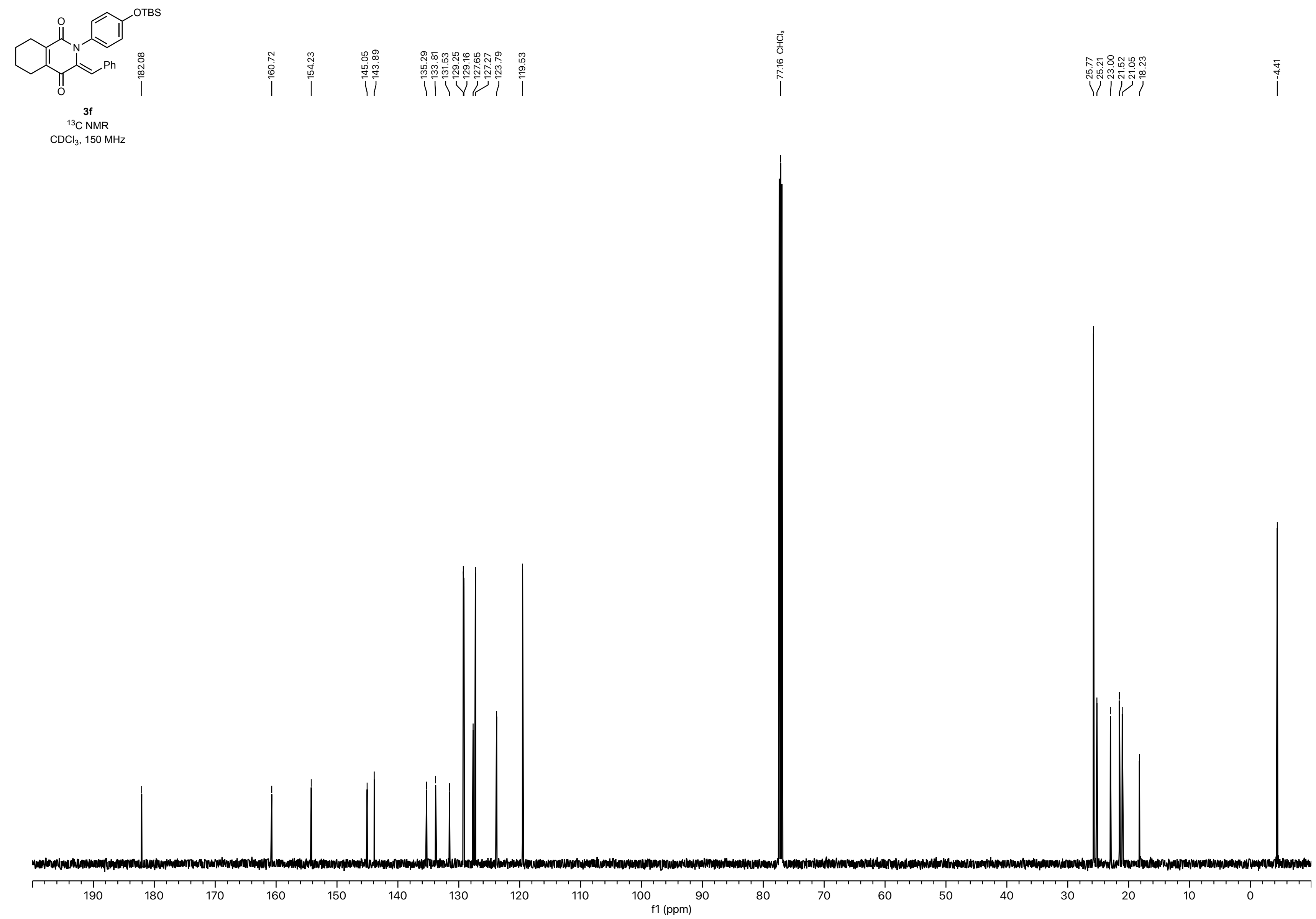

OTBS
O
N
Ph
O
3f
13C NMR
CDCl3, 150 MHz
182.08
160.72
154.23
145.05
143.89
135.29
133.81
131.53
129.25
129.16
127.65
127.27
123.79
119.53
77.16 CHCl3
25.77
25.21
23.00
21.52
21.05
18.23
-4.41
190
180
170
160
150
140
130
120
110
100
90
80
70
60
50
40
30
20
10
0
f1 (ppm)

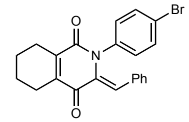

3g
^{1}H NMR
$(CD_3)_2CO$, 400 MHz

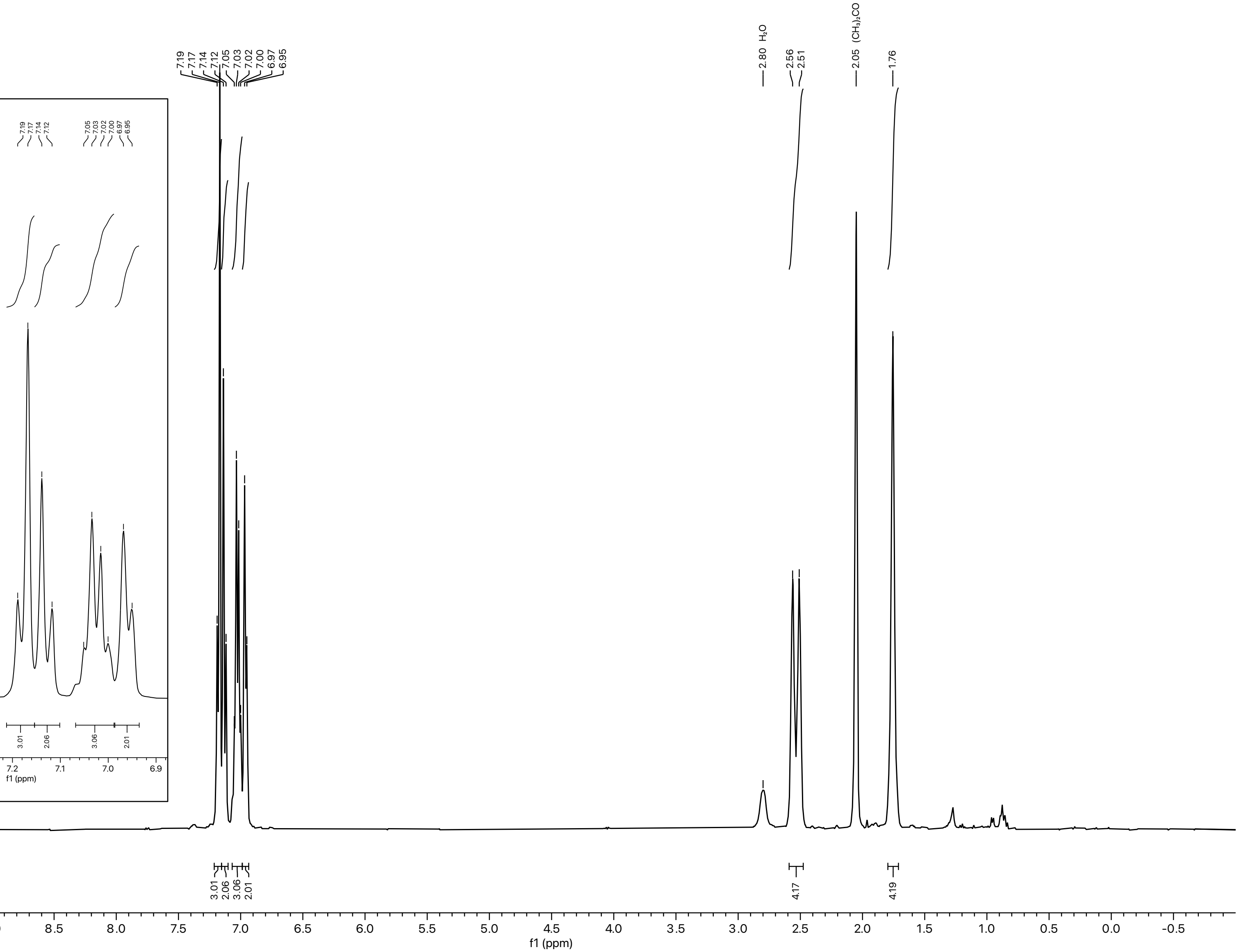

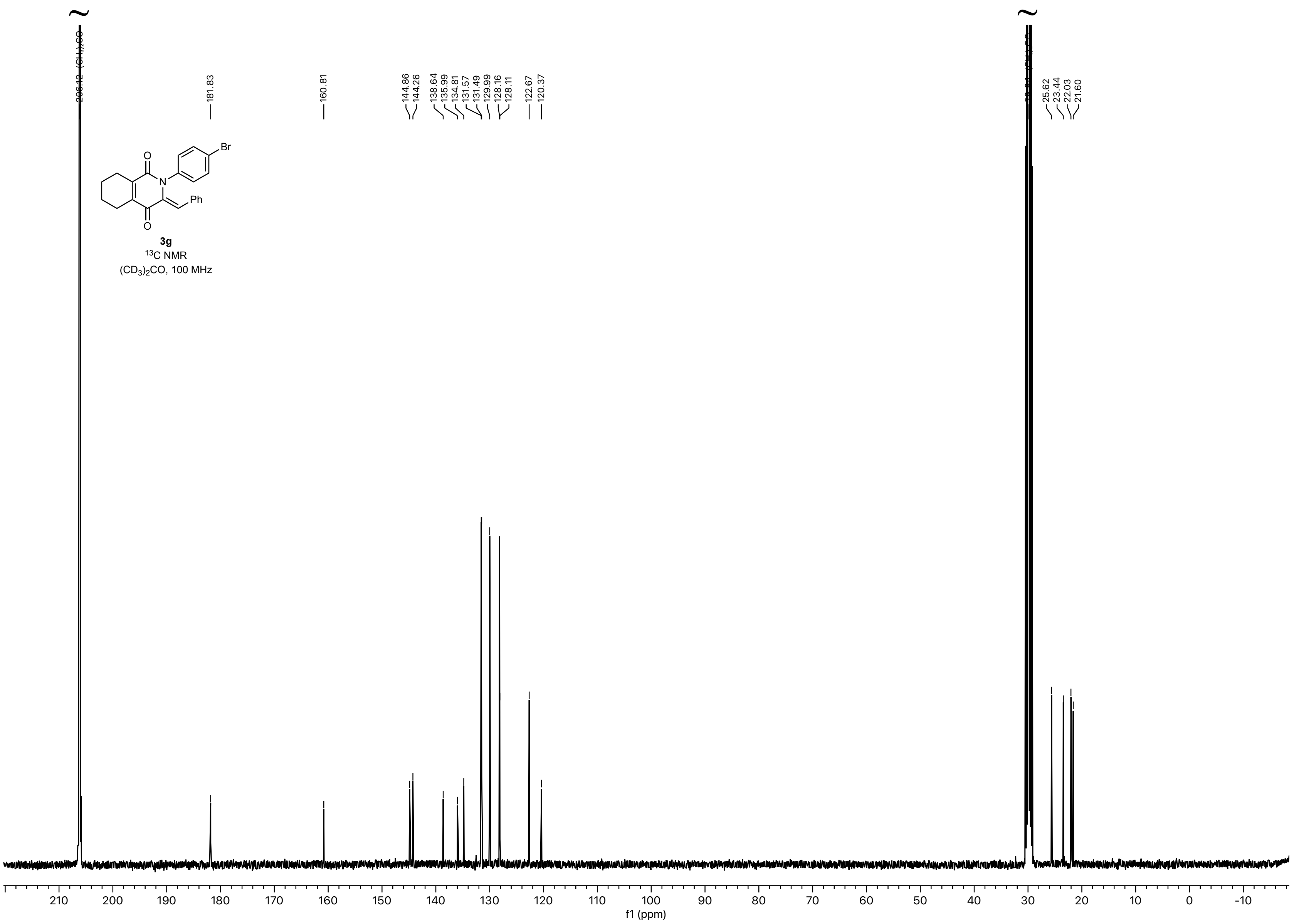

206.12 (CH3)2CO
181.83
160.81
144.86
144.26
138.64
135.99
134.81
131.57
131.49
129.99
128.16
128.11
122.67
120.37
25.62
23.44
22.03
21.60
Br
O
N
Ph
O
3g
13C NMR
(CD3)2CO, 100 MHz
210 200 190 180 170 160 150 140 130 120 110 100 90 80 70 60 50 40 30 20 10 0 -10
f1 (ppm)

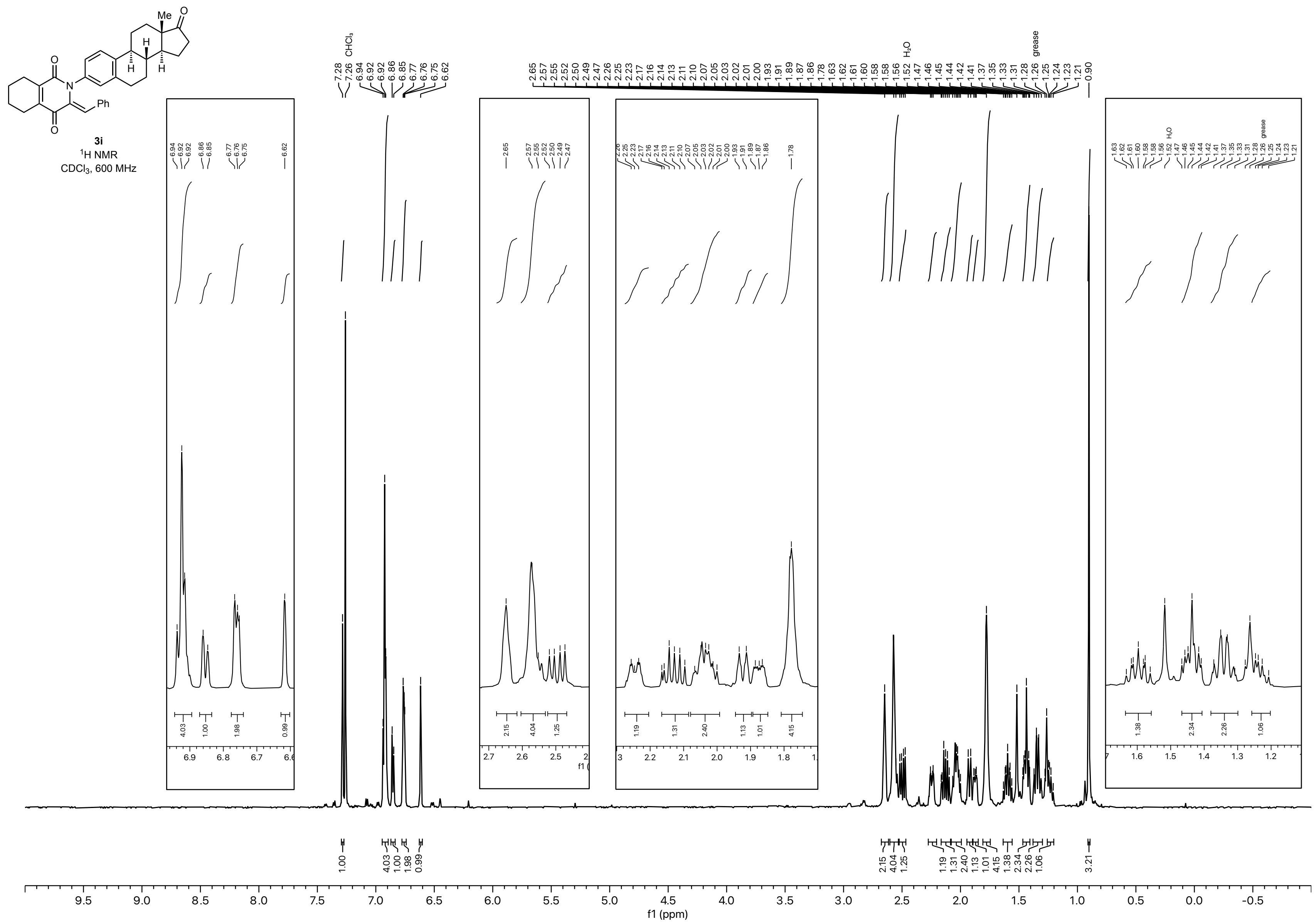

3i
^{1}H NMR
$CDCl_3$, 600 MHz
f1 (ppm)

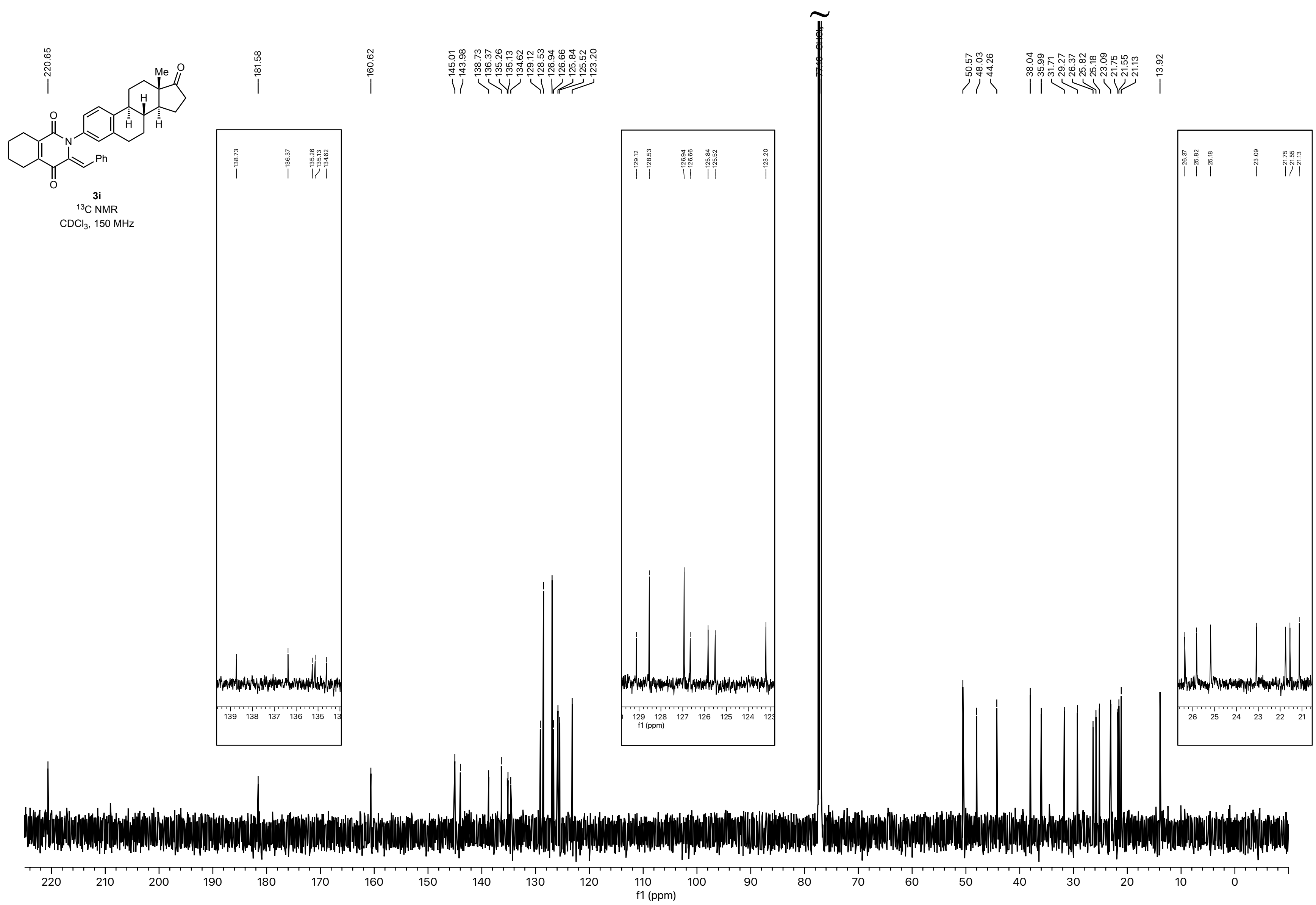

3i
^{13}C NMR
$CDCl_3$, 150 MHz

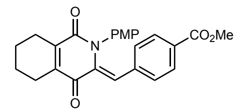

3j

^1H NMR

$CDCl_3$, 900 MHz

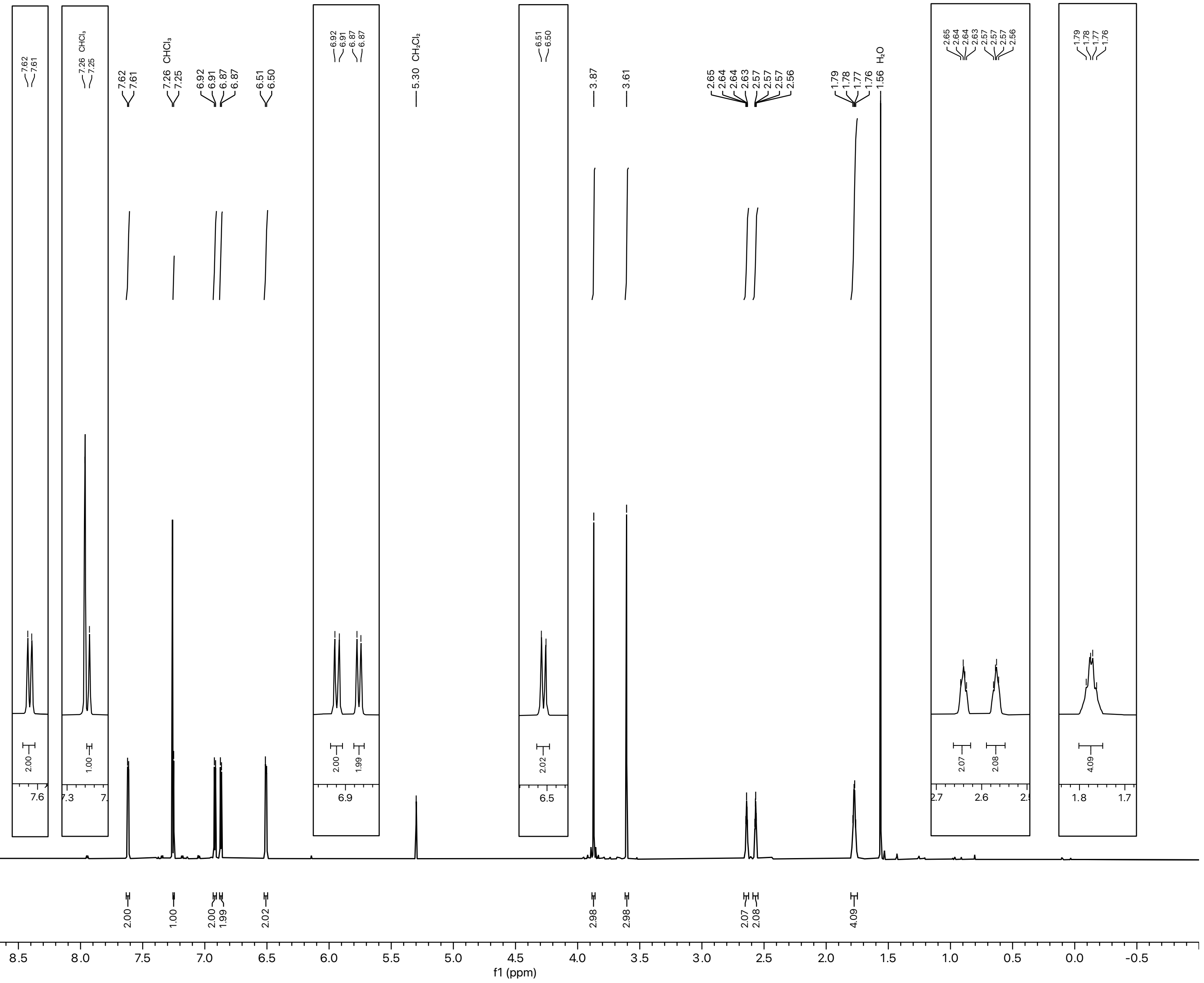

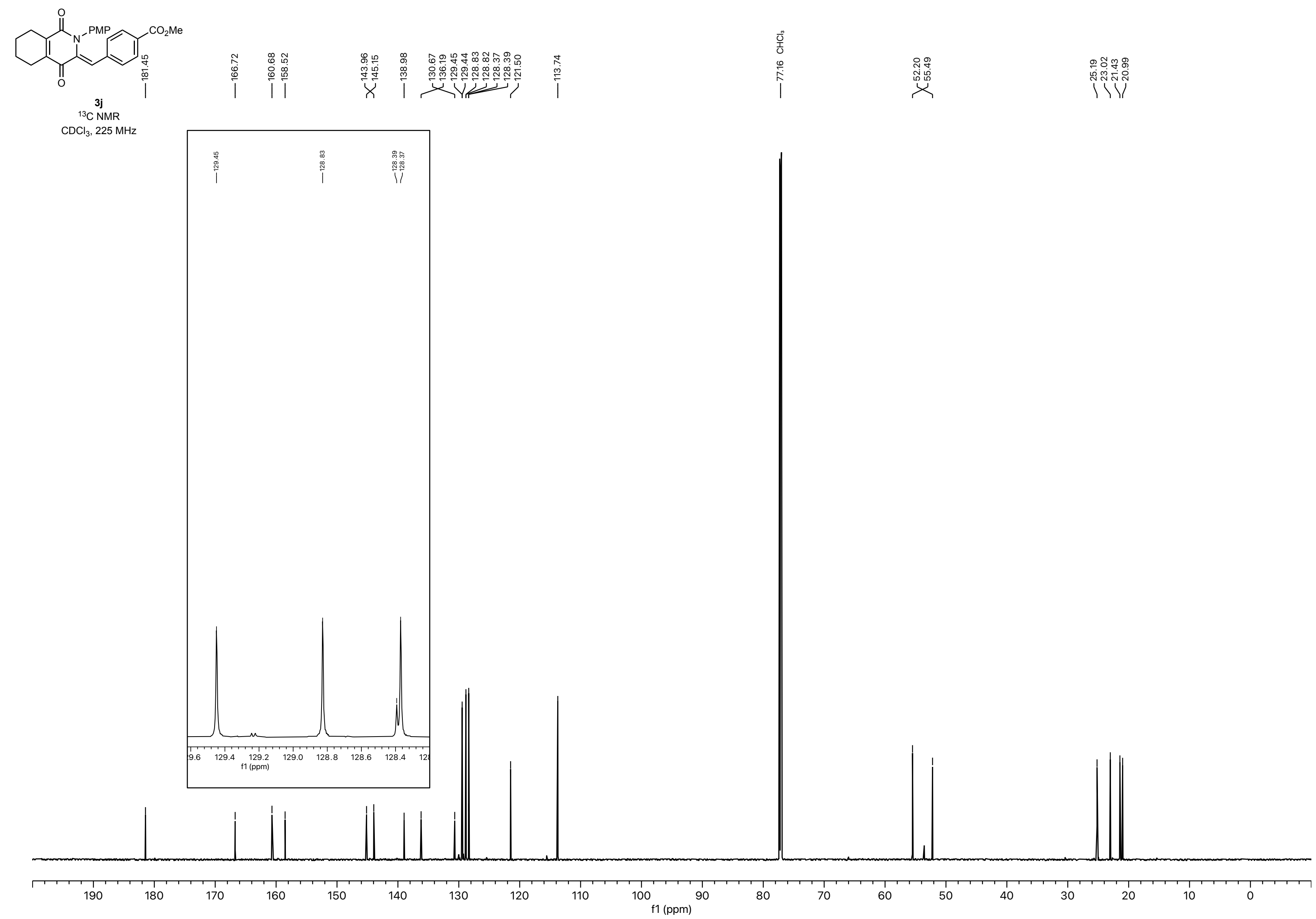

PMP
CO2Me
3j
13C NMR
CDCl3, 225 MHz
181.45
166.72
160.68
158.52
143.96
145.15
138.98
130.67
136.19
129.45
129.44
128.83
128.82
128.37
128.39
121.50
113.74
77.16 CHCl3
52.20
55.49
25.19
23.02
21.43
20.99
129.45
128.83
128.39
128.37
f1 (ppm)
f1 (ppm)

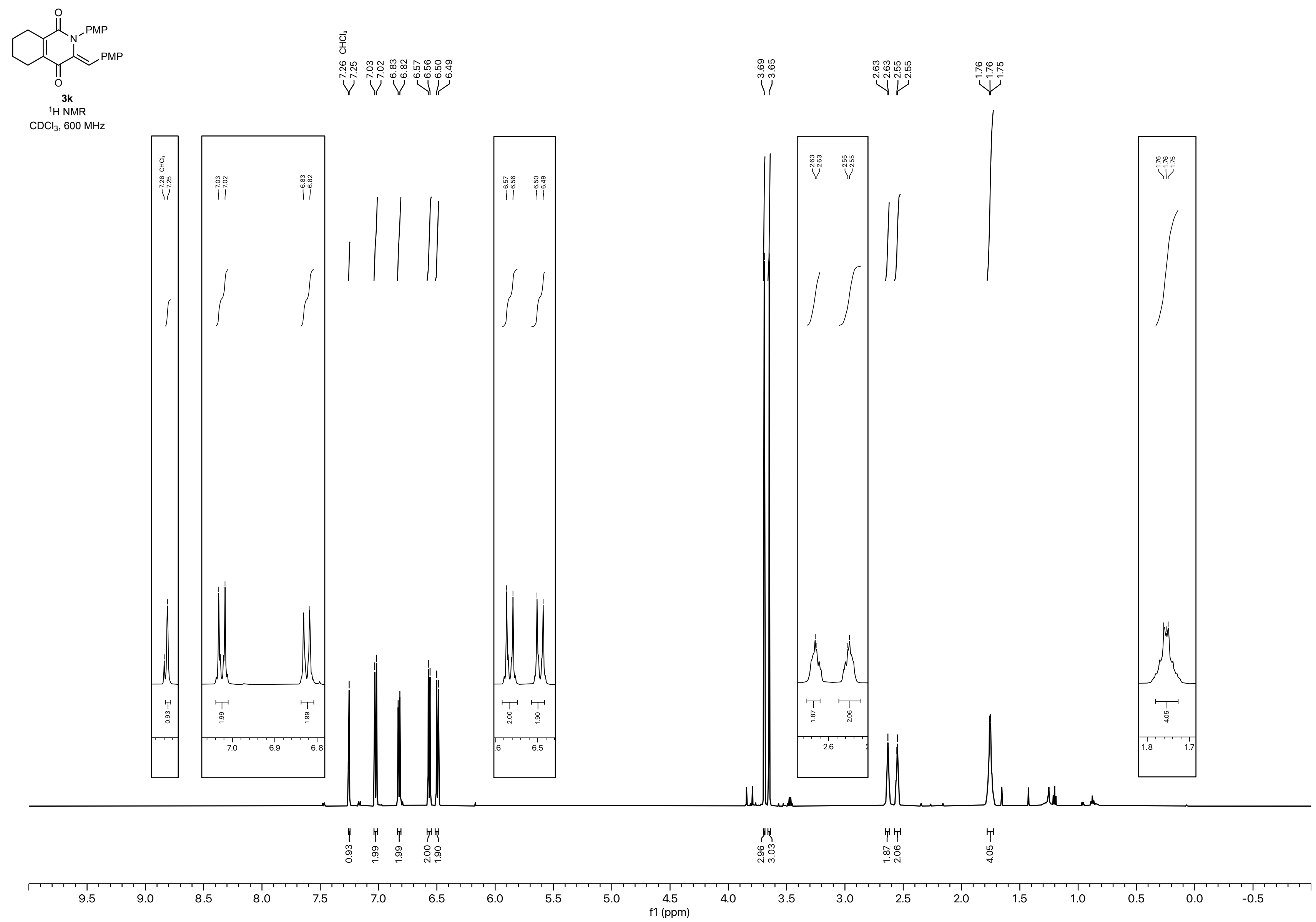

PMP
PMP
O
O
N
3k
1H NMR
CDCl3, 600 MHz
7.26 CHCl3
7.25
7.03
7.02
6.83
6.82
6.57
6.56
6.50
6.49
3.69
3.65
2.63
2.63
2.55
2.55
1.76
1.76
1.75
0.93
1.99
1.99
2.00
1.90
2.96
3.03
1.87
2.06
4.05
f1 (ppm)

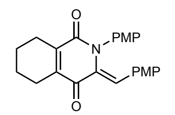

3k

^{13}C NMR

$CDCl_3$, 150 MHz

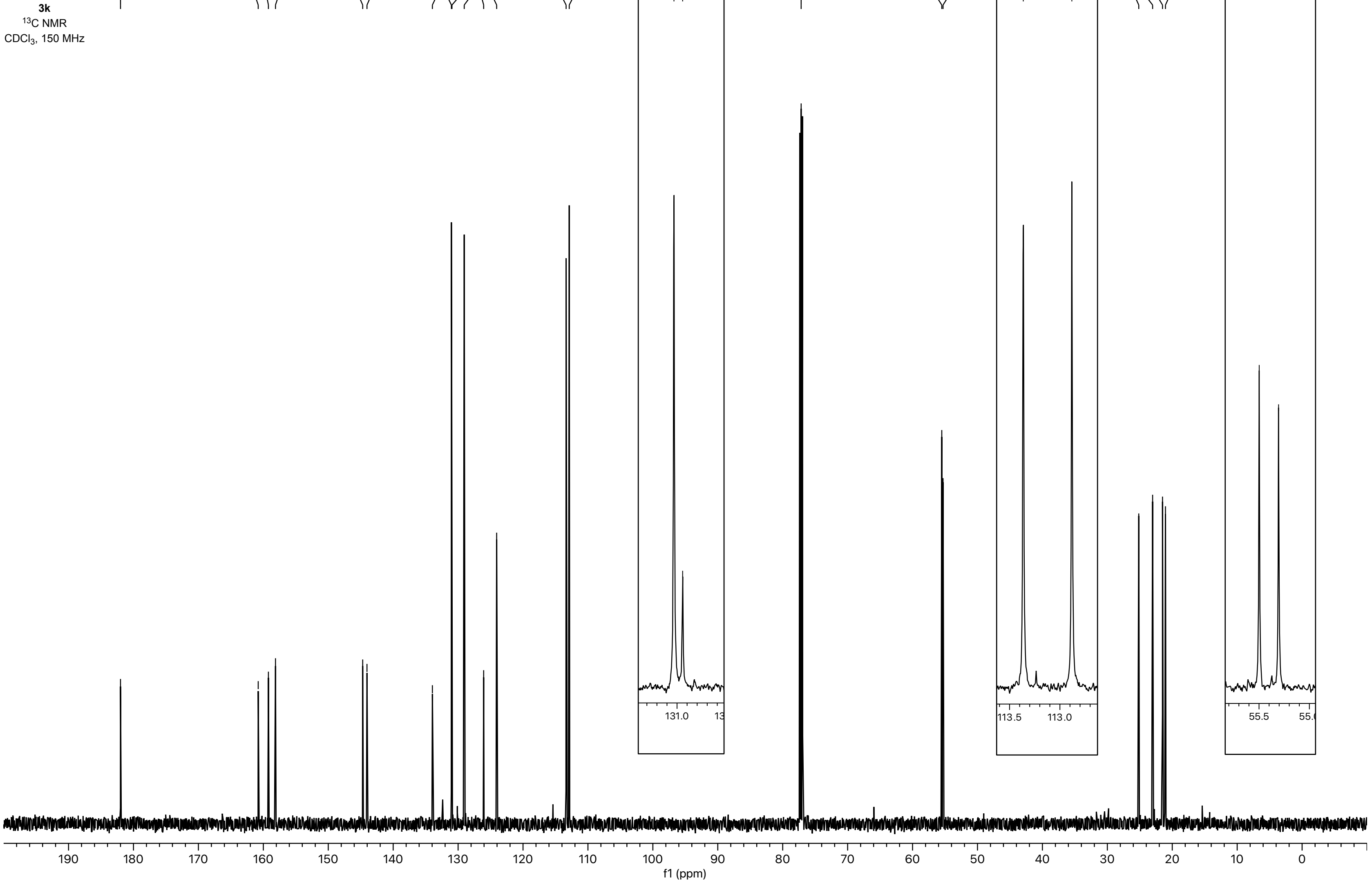

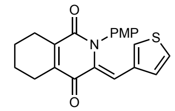

3l
^{1}H NMR
$CDCl_3$, 400 MHz

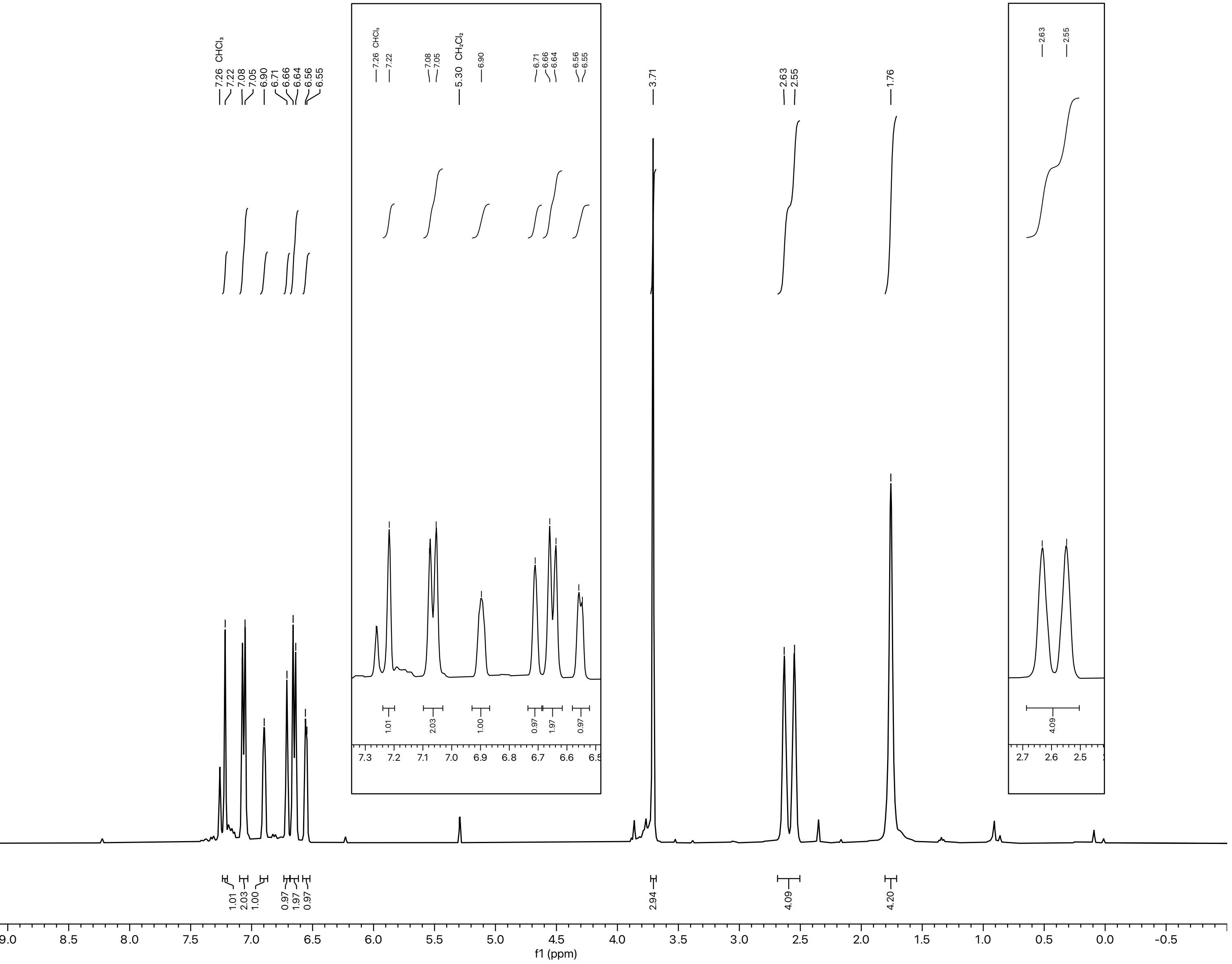

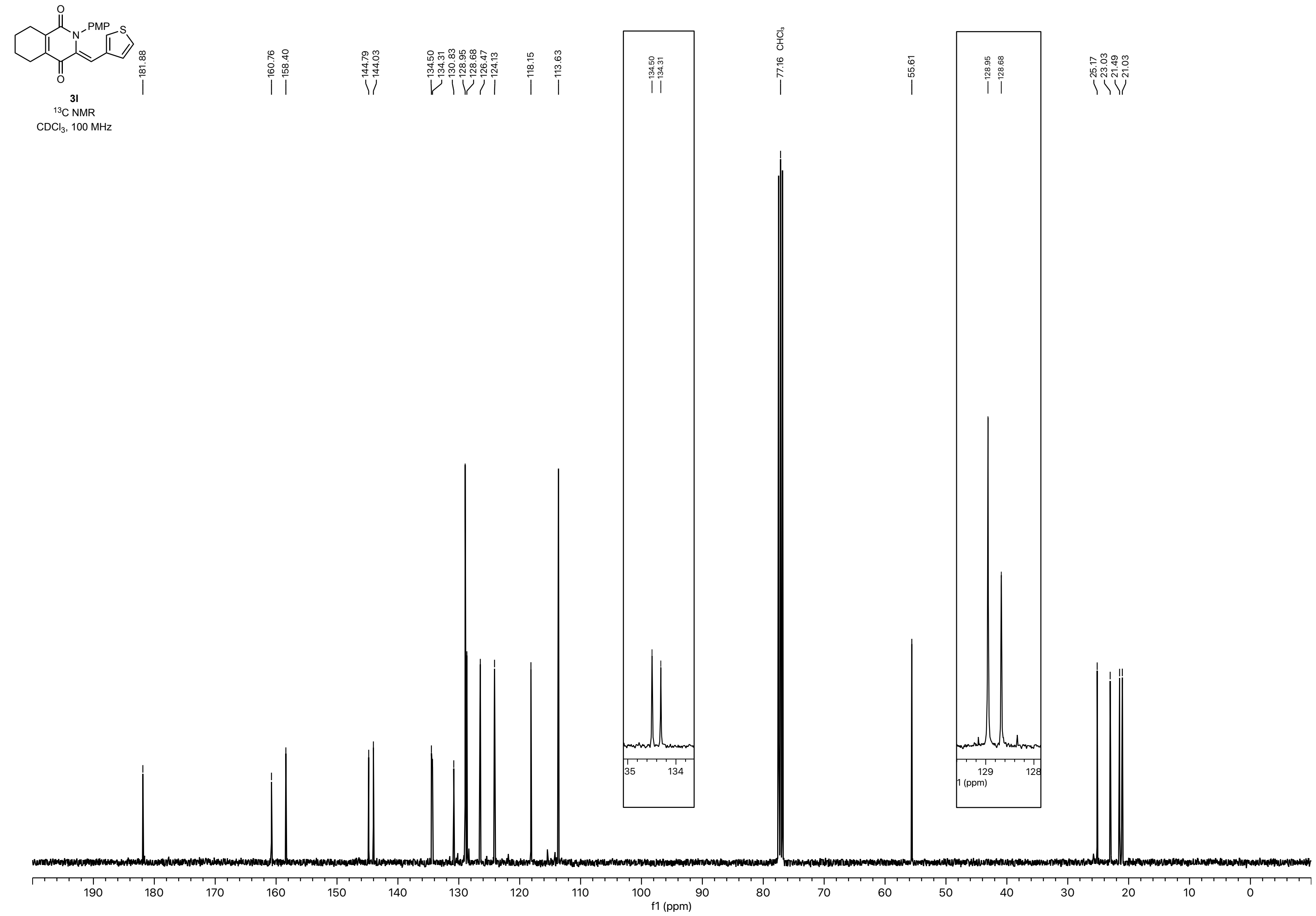

PMP
S
O
N
3l
^{13}C NMR
$CDCl_3$, 100 MHz
181.88
160.76
158.40
144.79
144.03
134.50
134.31
130.83
128.95
128.68
126.47
124.13
118.15
113.63
77.16 $CHCl_3$
55.61
25.17
23.03
21.49
21.03
f1 (ppm)

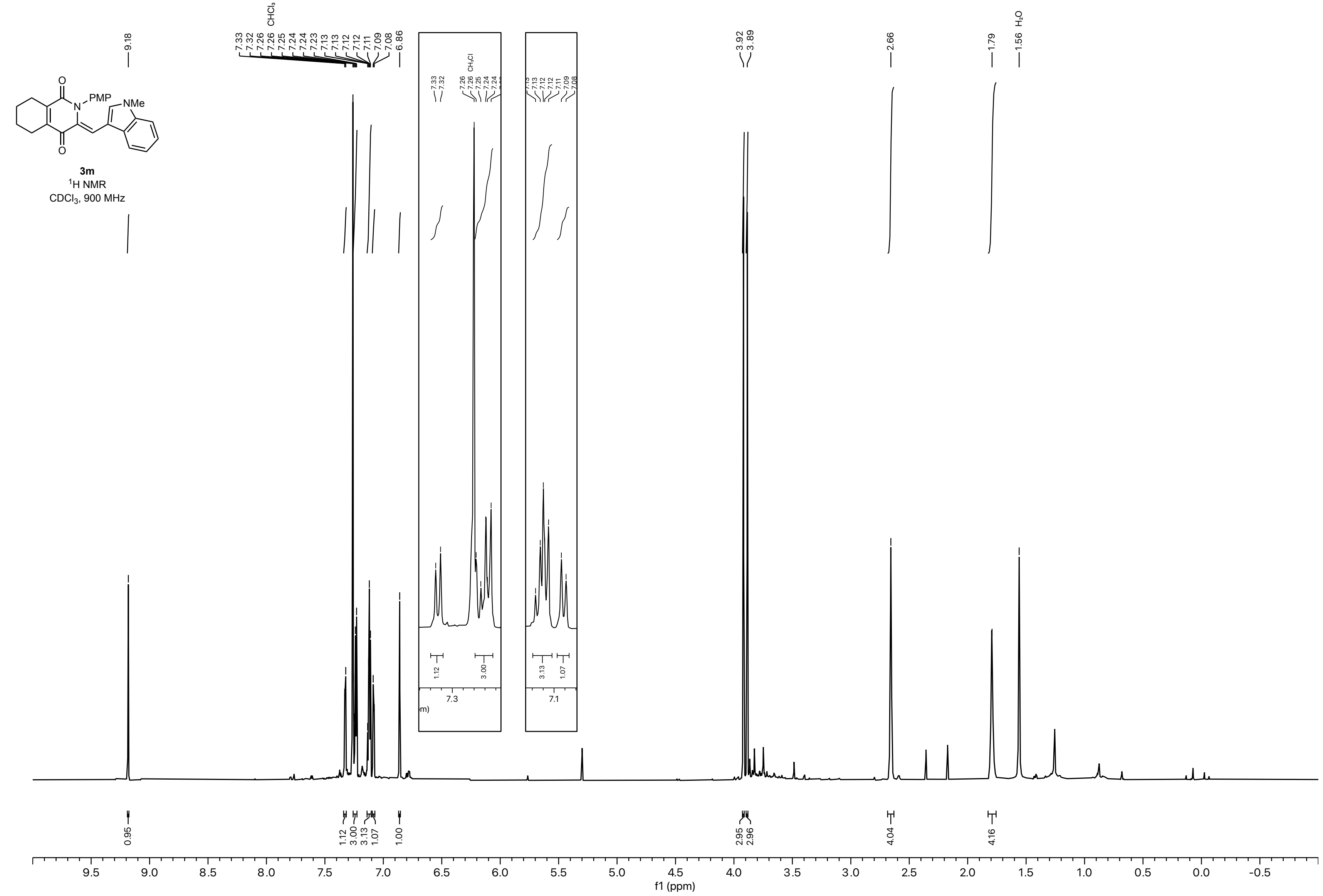

3m
1H NMR
CDCl3, 900 MHz
PMP
NMe
9.18
7.33
7.32
7.26
7.26
CHCl3
7.25
7.24
7.24
7.23
7.13
7.13
7.12
7.12
7.11
7.09
7.08
6.86
3.92
3.89
2.66
1.79
1.56 H2O
0.95
1.12
3.00
3.13
1.07
1.00
2.95
2.96
4.04
4.16
f1 (ppm)

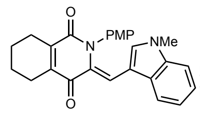

3m
^{13}C NMR
$CDCl_3$, 225 MHz

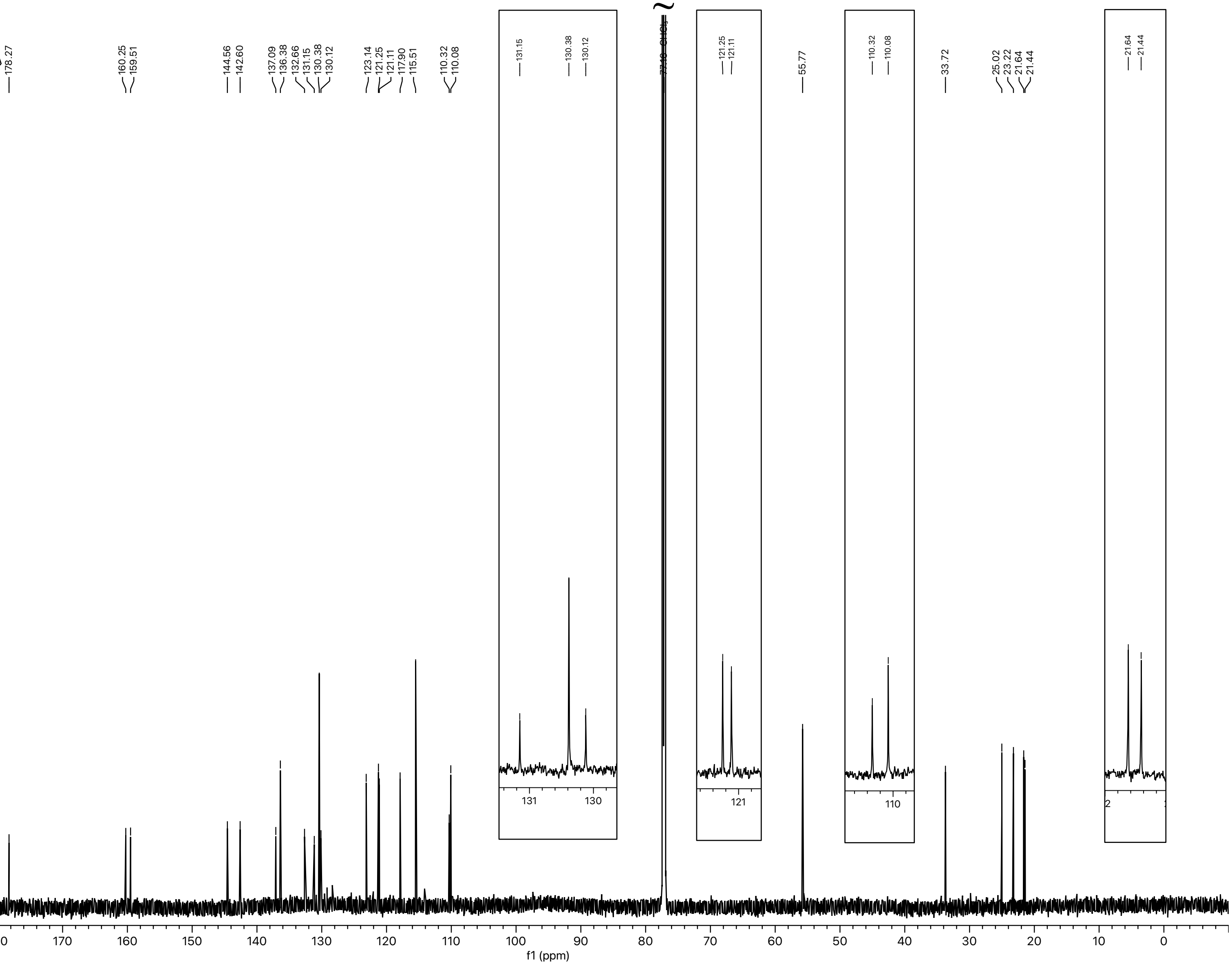

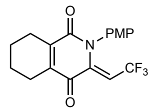

3n
^1H NMR
$(CD_3)_2CO$, 600 MHz

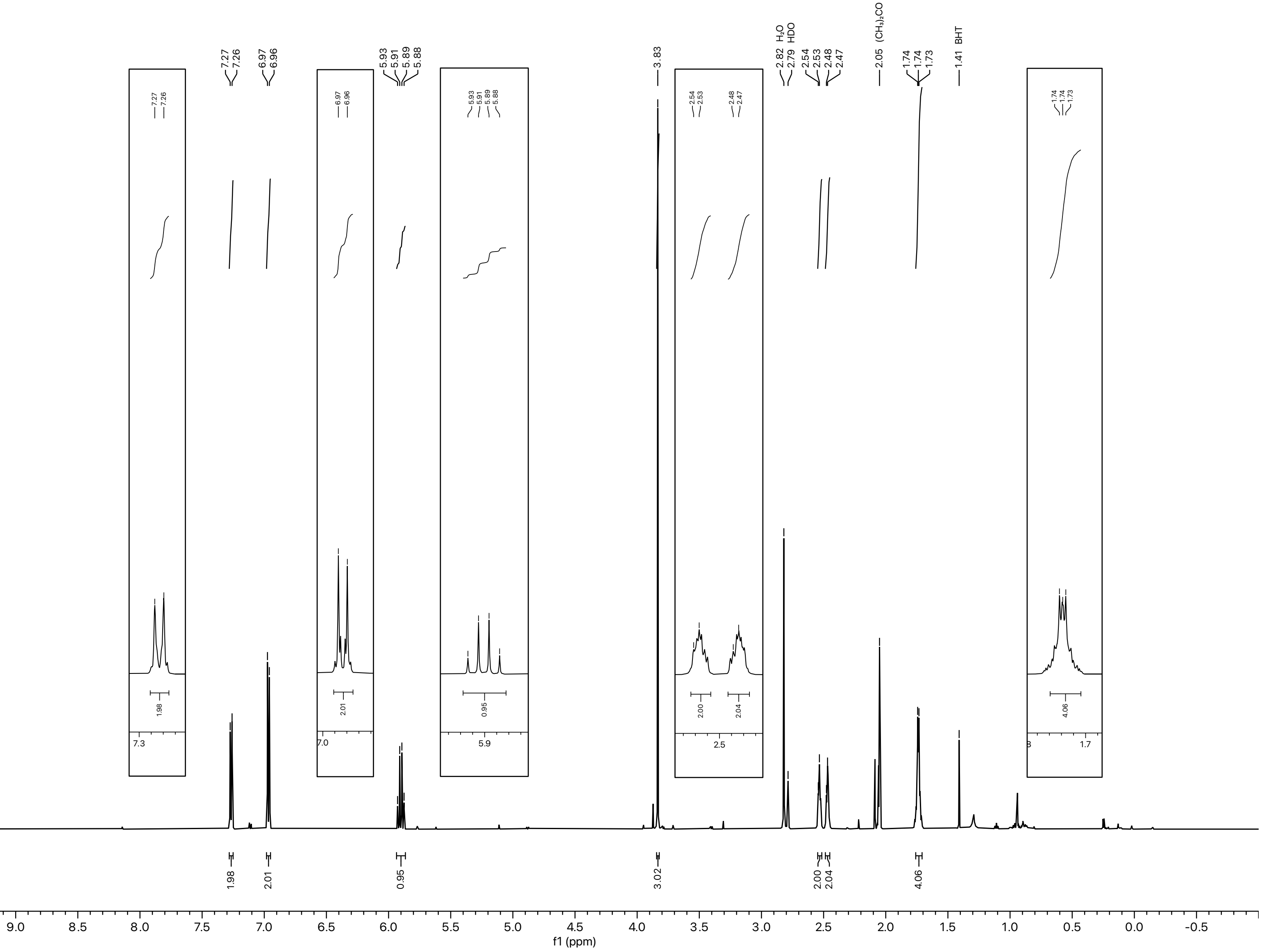

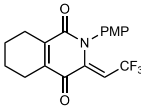

3n
^{13}C NMR
$(CD_3)_2CO$, 150 MHz

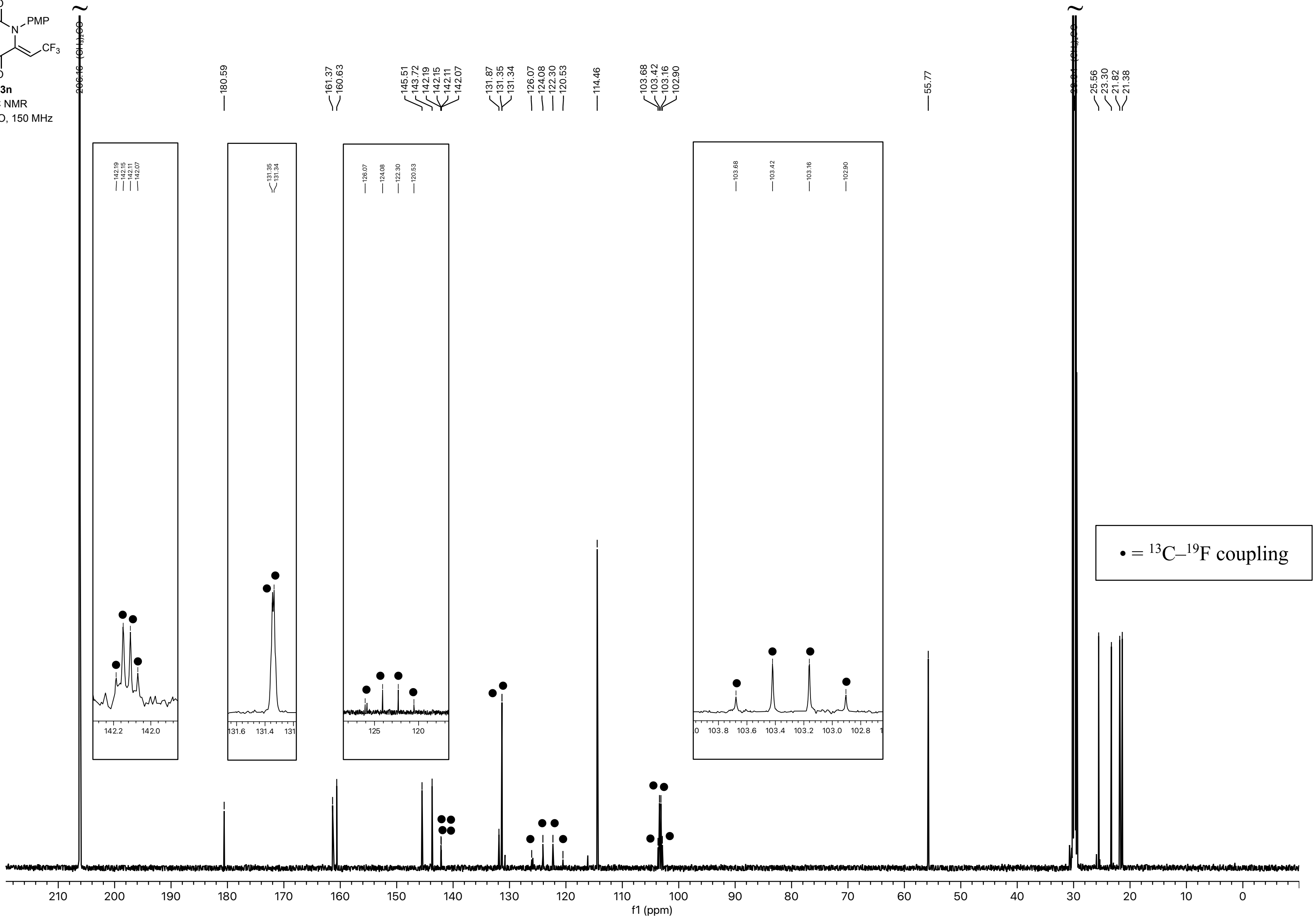

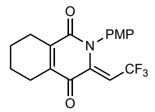

3n

^{19}F NMR

$CDCl_3$, 377 MHz

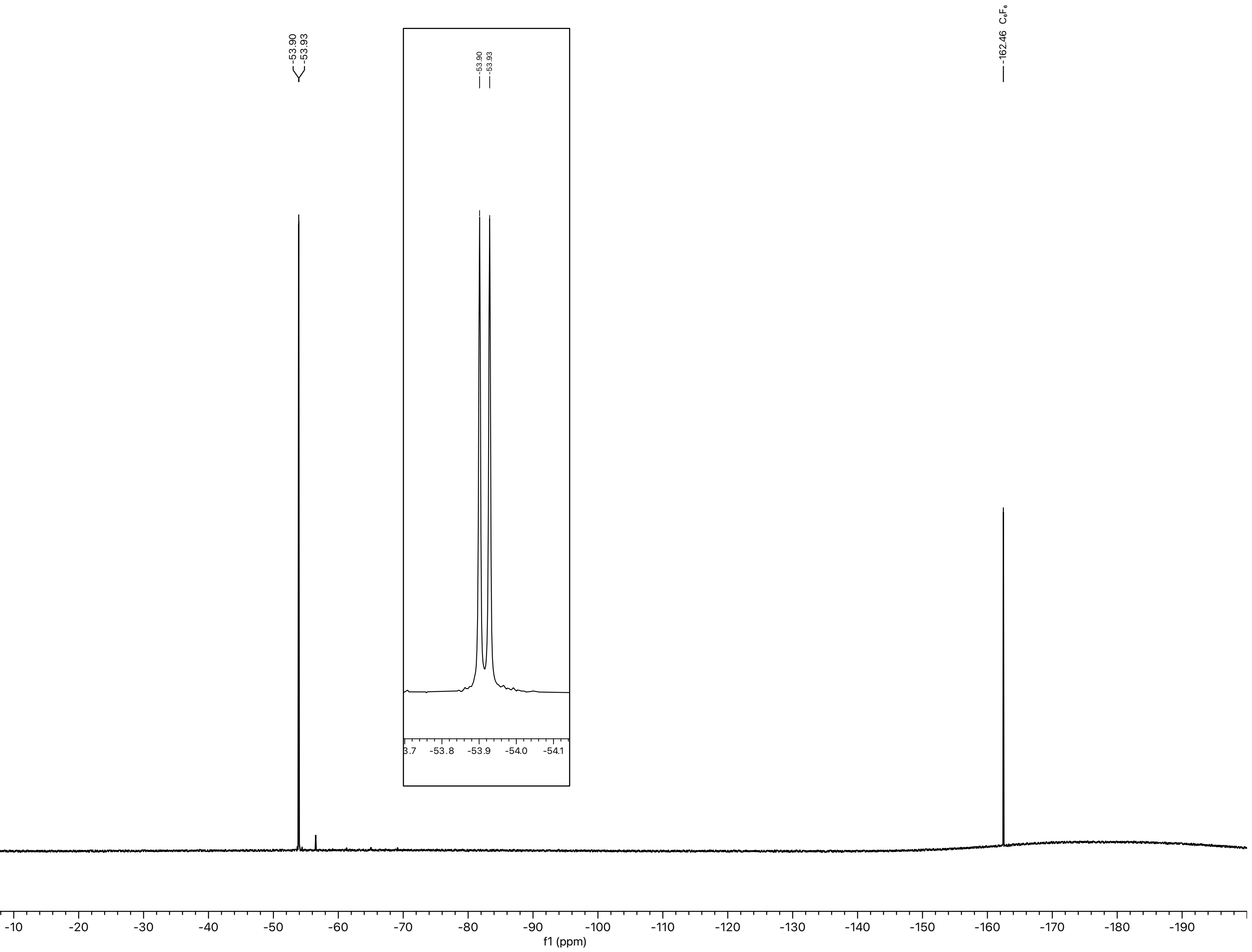

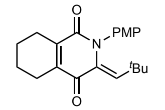

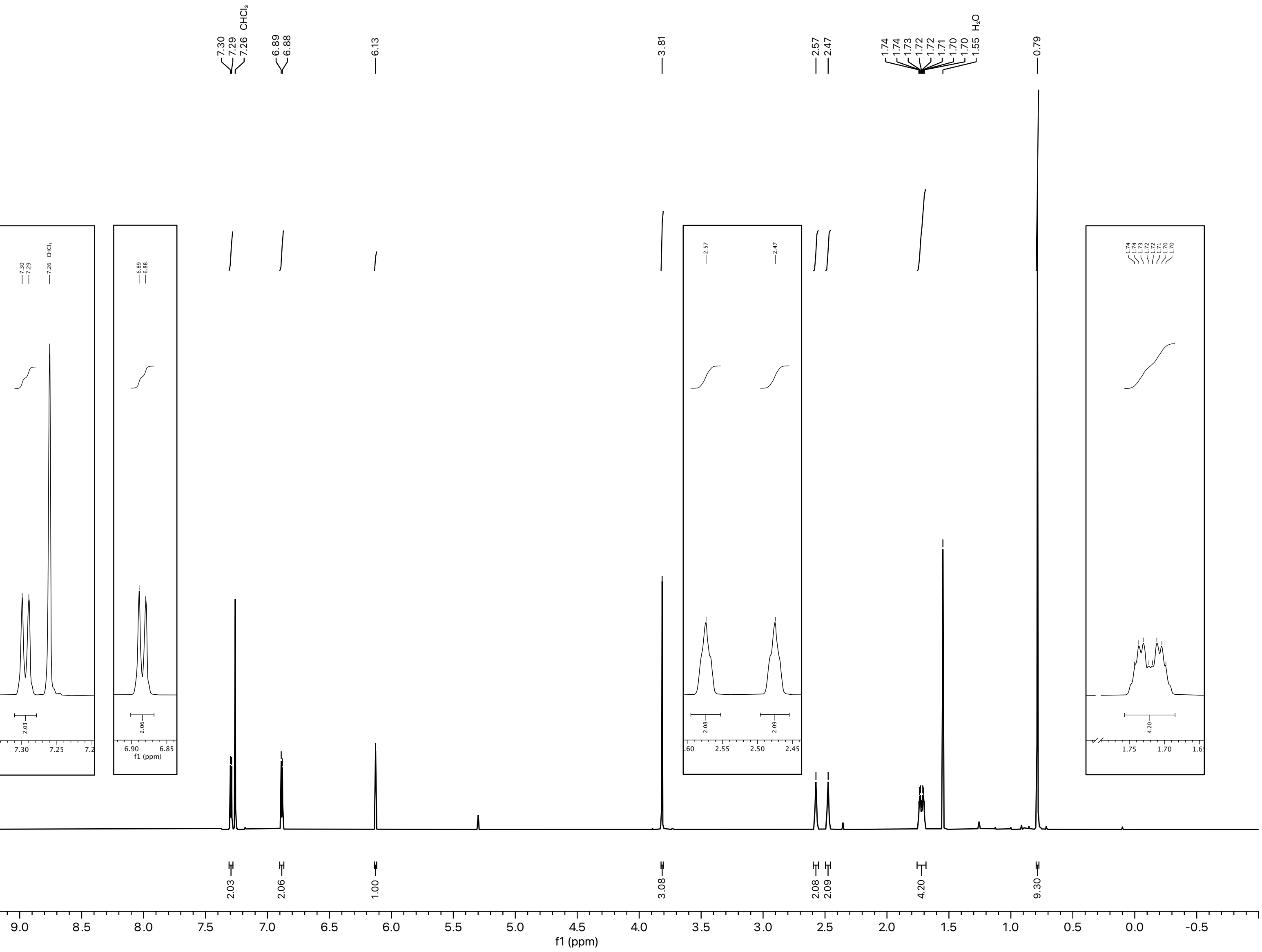

7.30
7.29
7.26
CHCl3
6.89
6.88
6.13
3.81
2.57
2.47
1.74
1.74
1.73
1.72
1.72
1.71
1.70
1.70
1.55
H2O
0.79
2.03
2.06
1.00
3.08
2.08
2.09
4.20
9.30
9.5
9.0
8.5
8.0
7.5
7.0
6.5
6.0
5.5
5.0
4.5
4.0
3.5
3.0
2.5
2.0
1.5
1.0
0.5
0.0
-0.5
f1 (ppm)

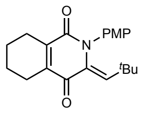

3o
^{13}C NMR
$CDCl_3$, 225 MHz

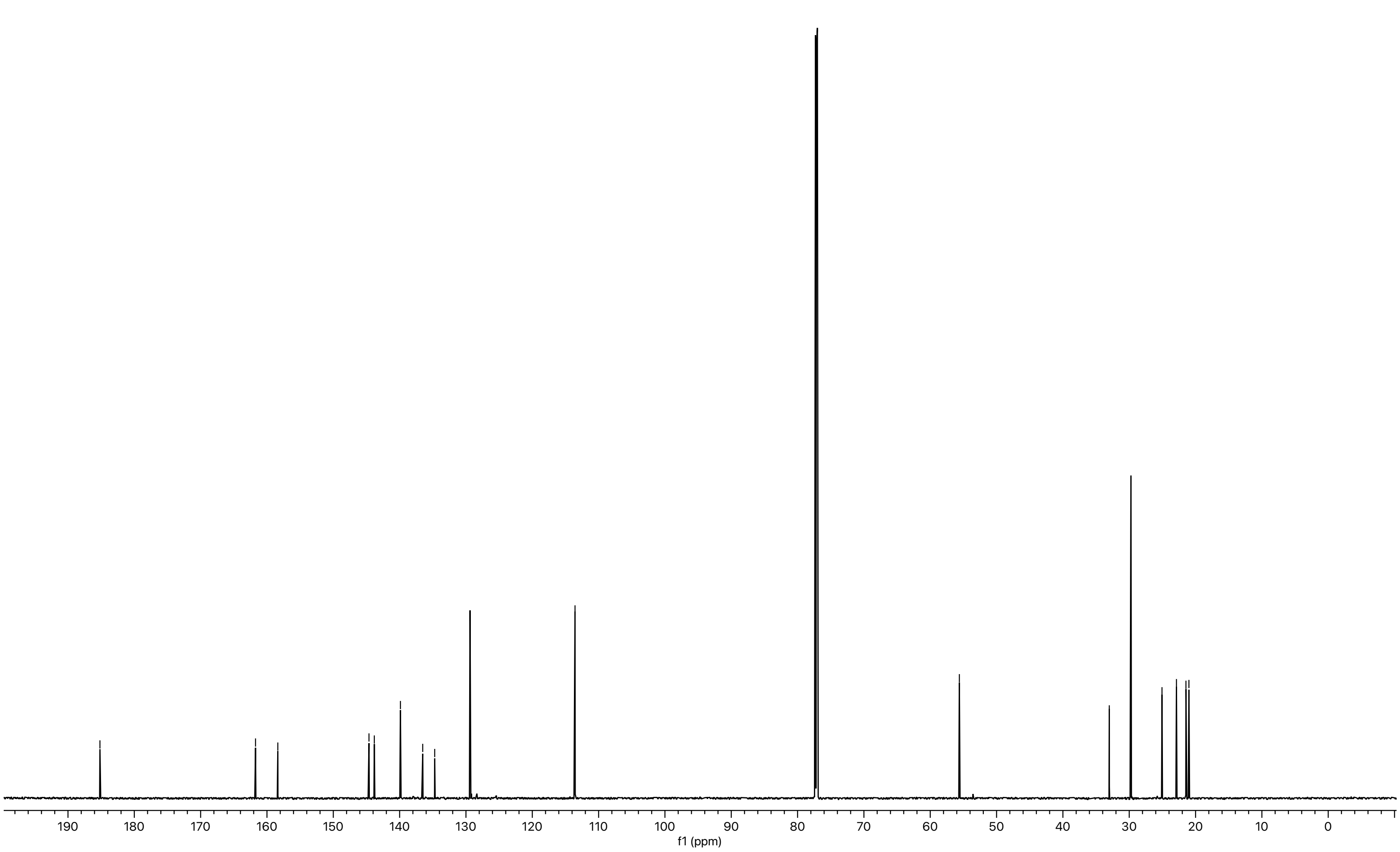

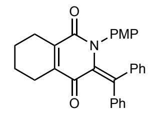

3p
^1H NMR
$CDCl_3$, 400 MHz

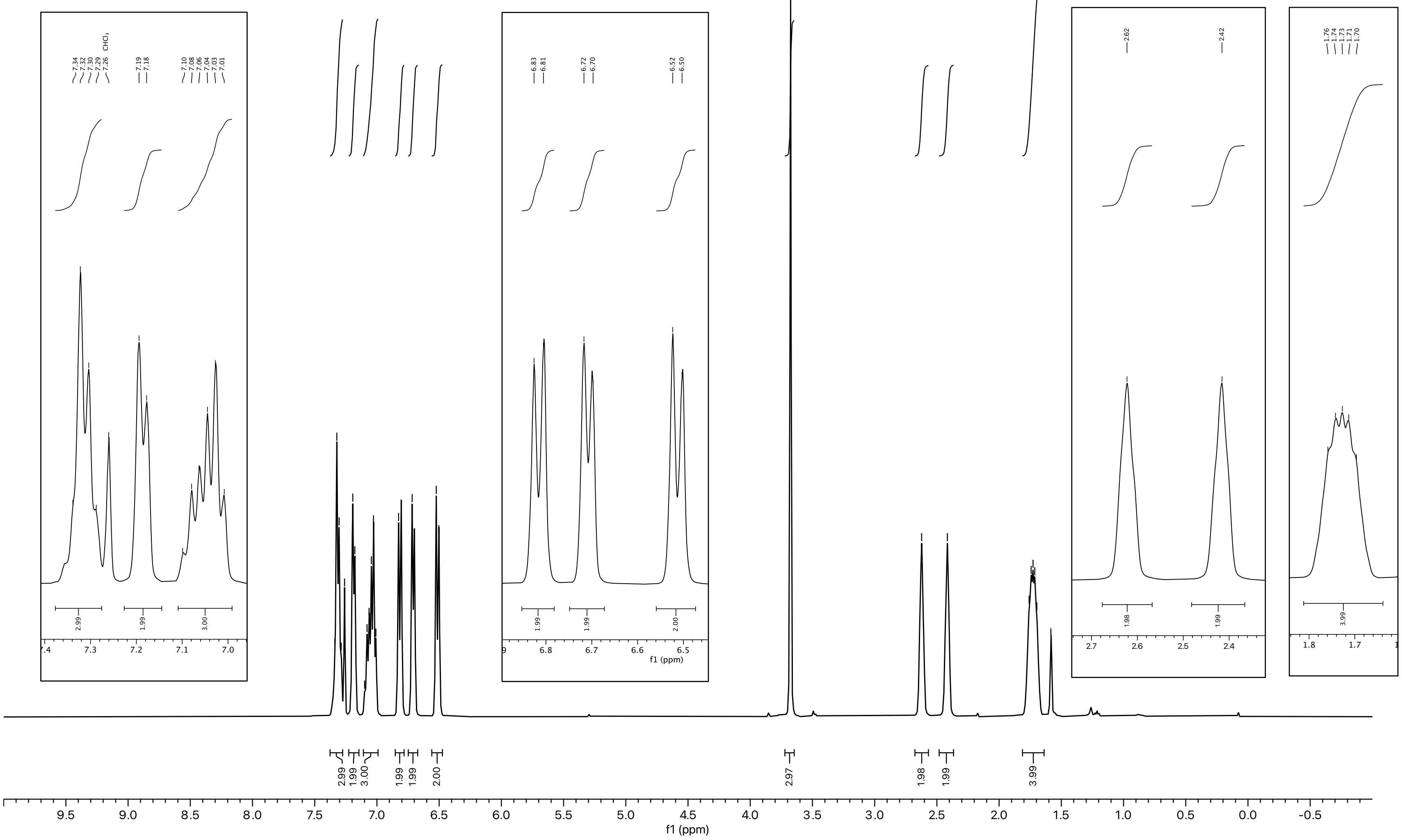

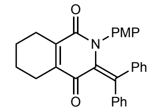

3p
^{13}C NMR
$CDCl_3$, 100 MHz

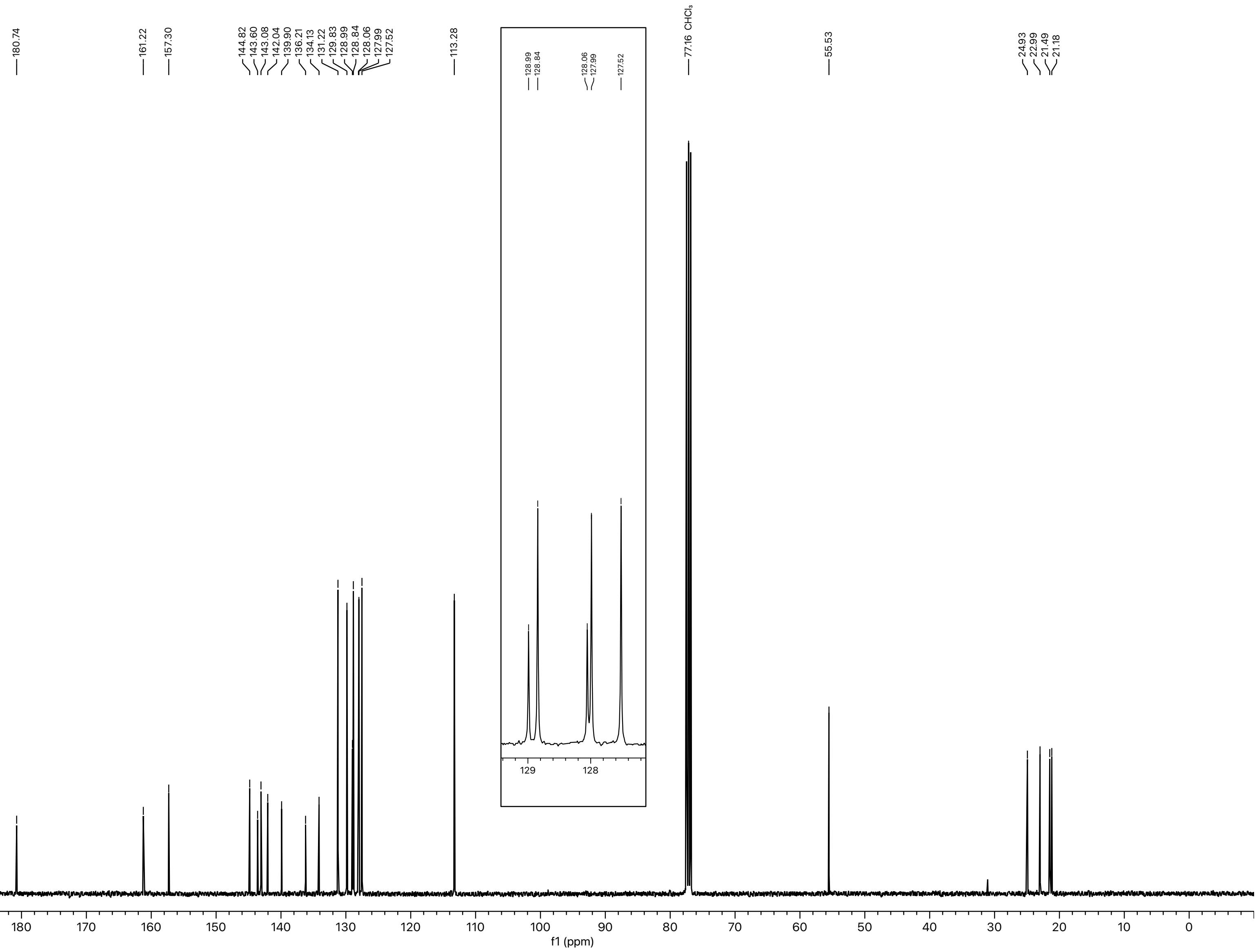

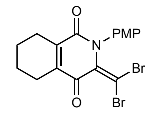

3q
^{1}H NMR
$CDCl_3$, 600 MHz

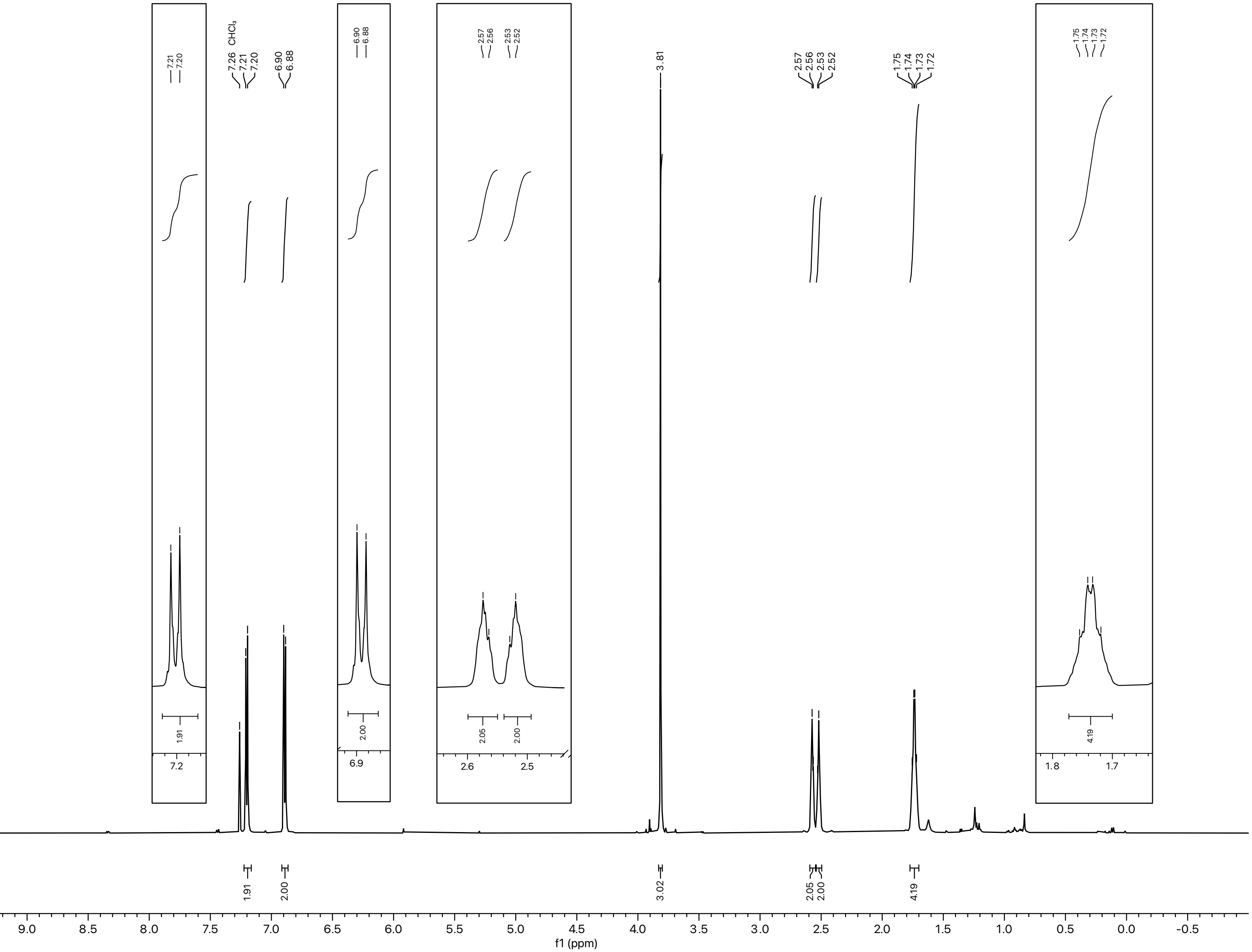

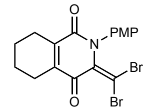

3q
^{13}C NMR
$CDCl_3$, 225 MHz

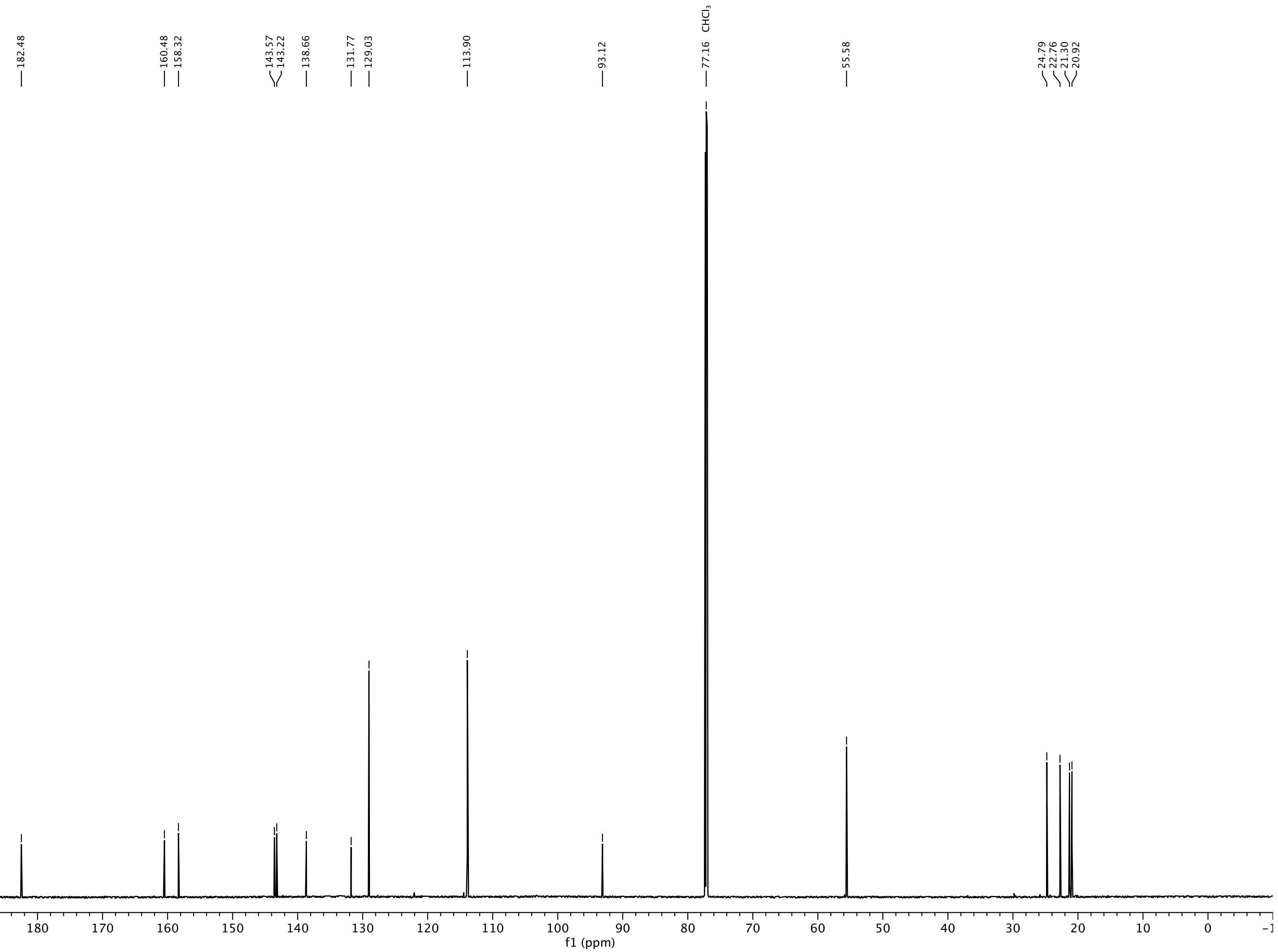

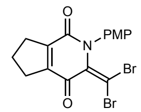

3r
^1H NMR
$CDCl_3$, 600 MHz

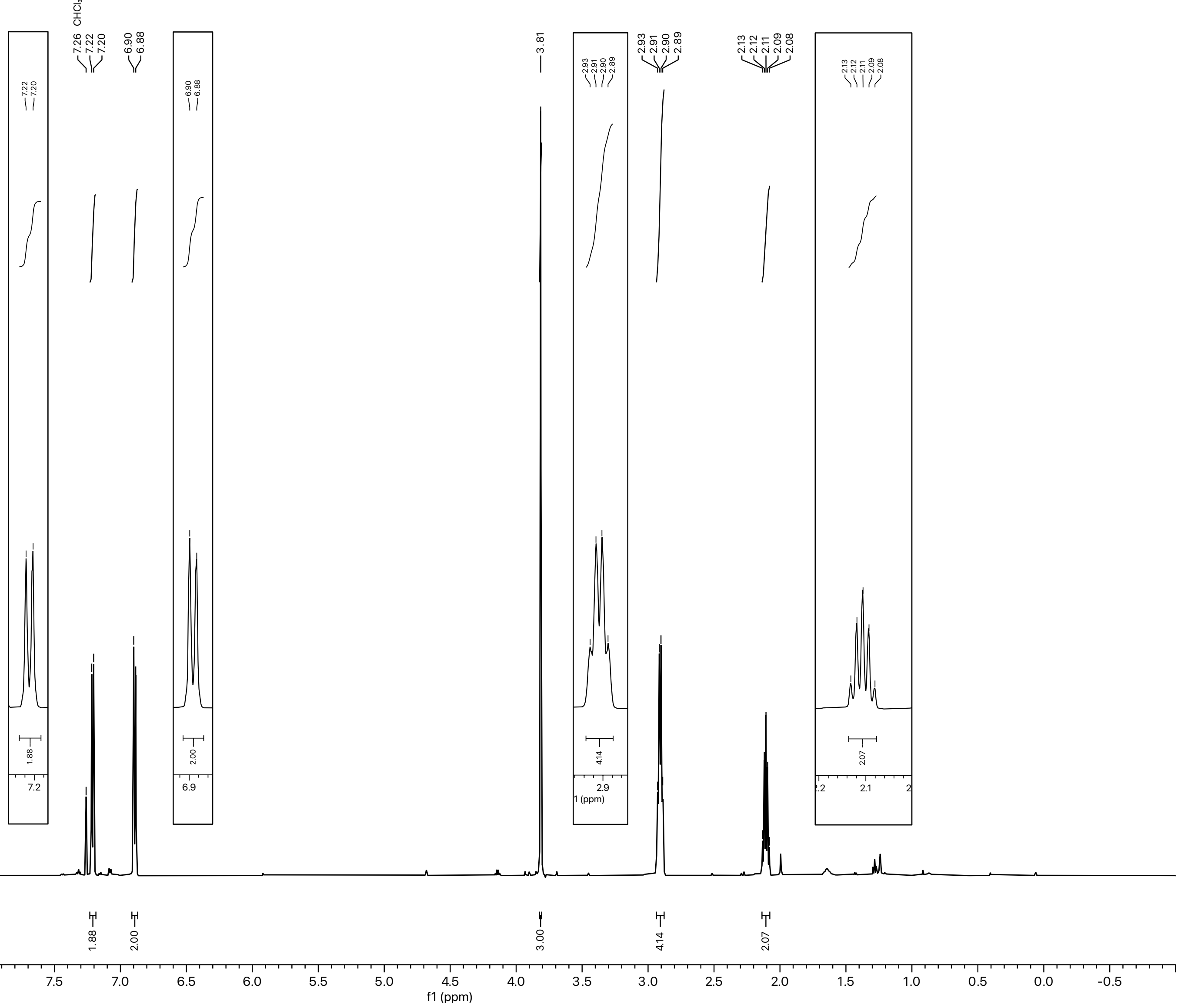

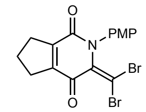

3r
^{13}C NMR
$CDCl_3$, 150 MHz

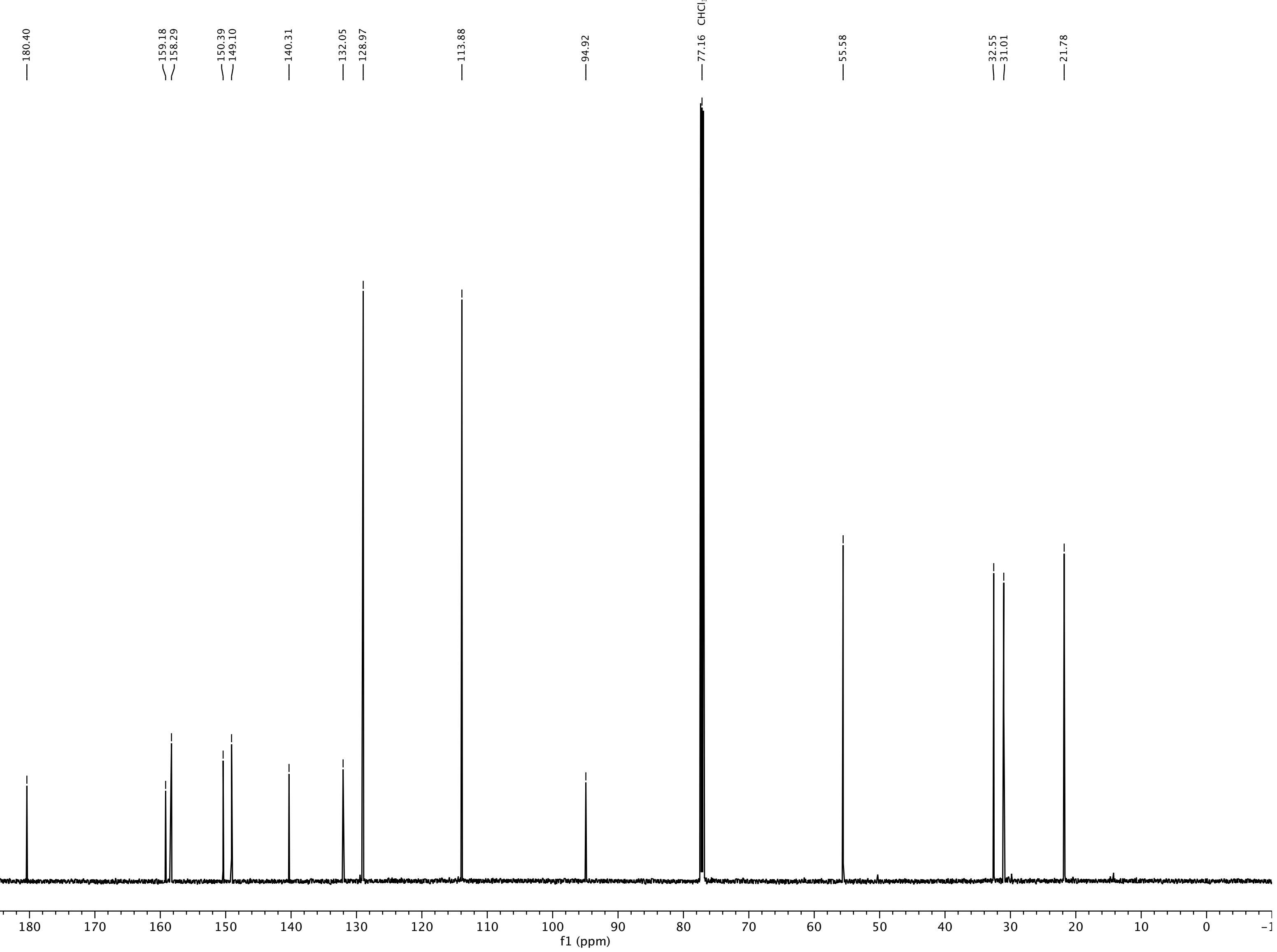

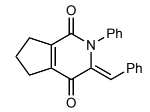

3s
^{1}H NMR
$(CD_3)_2CO$, 400 MHz

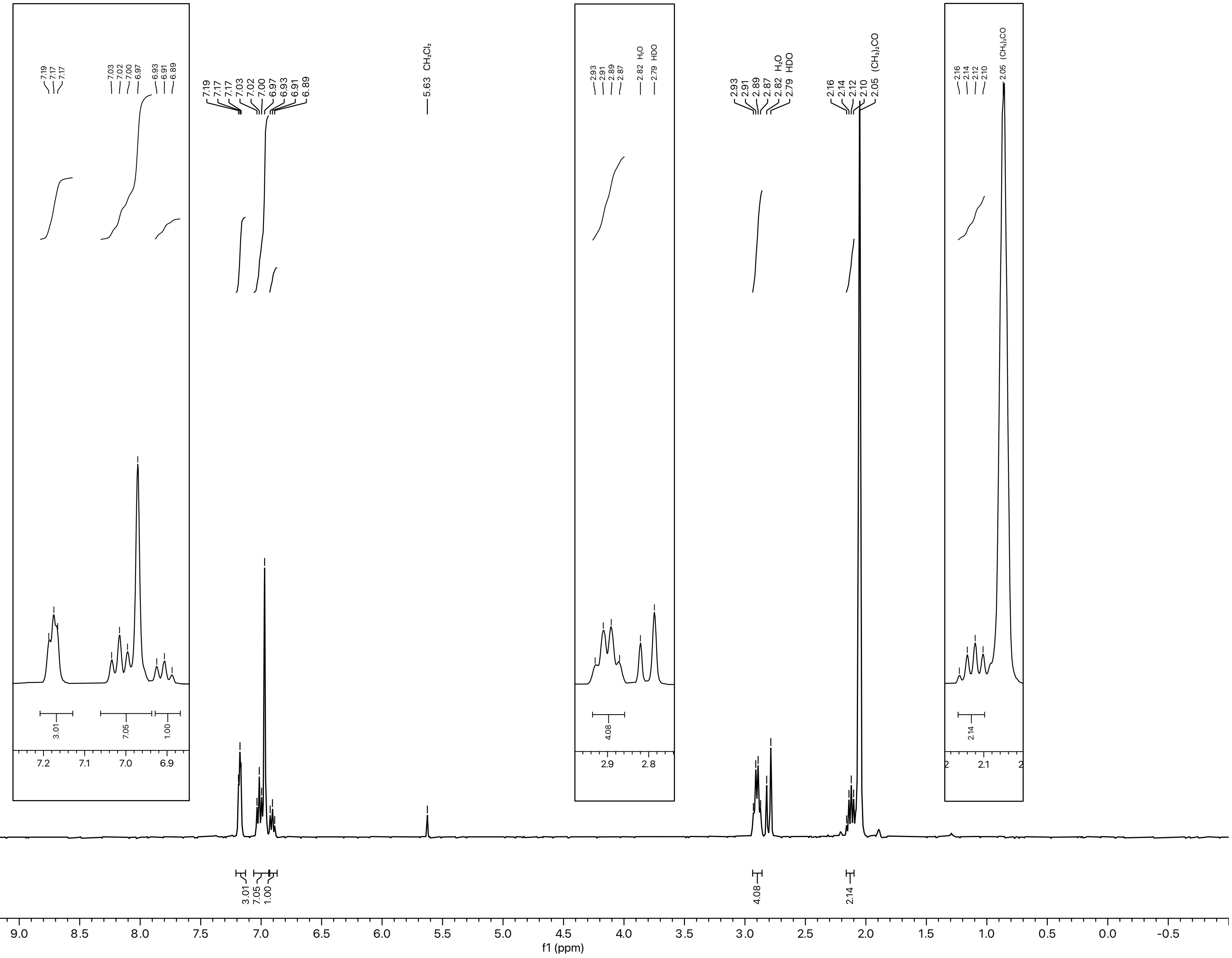

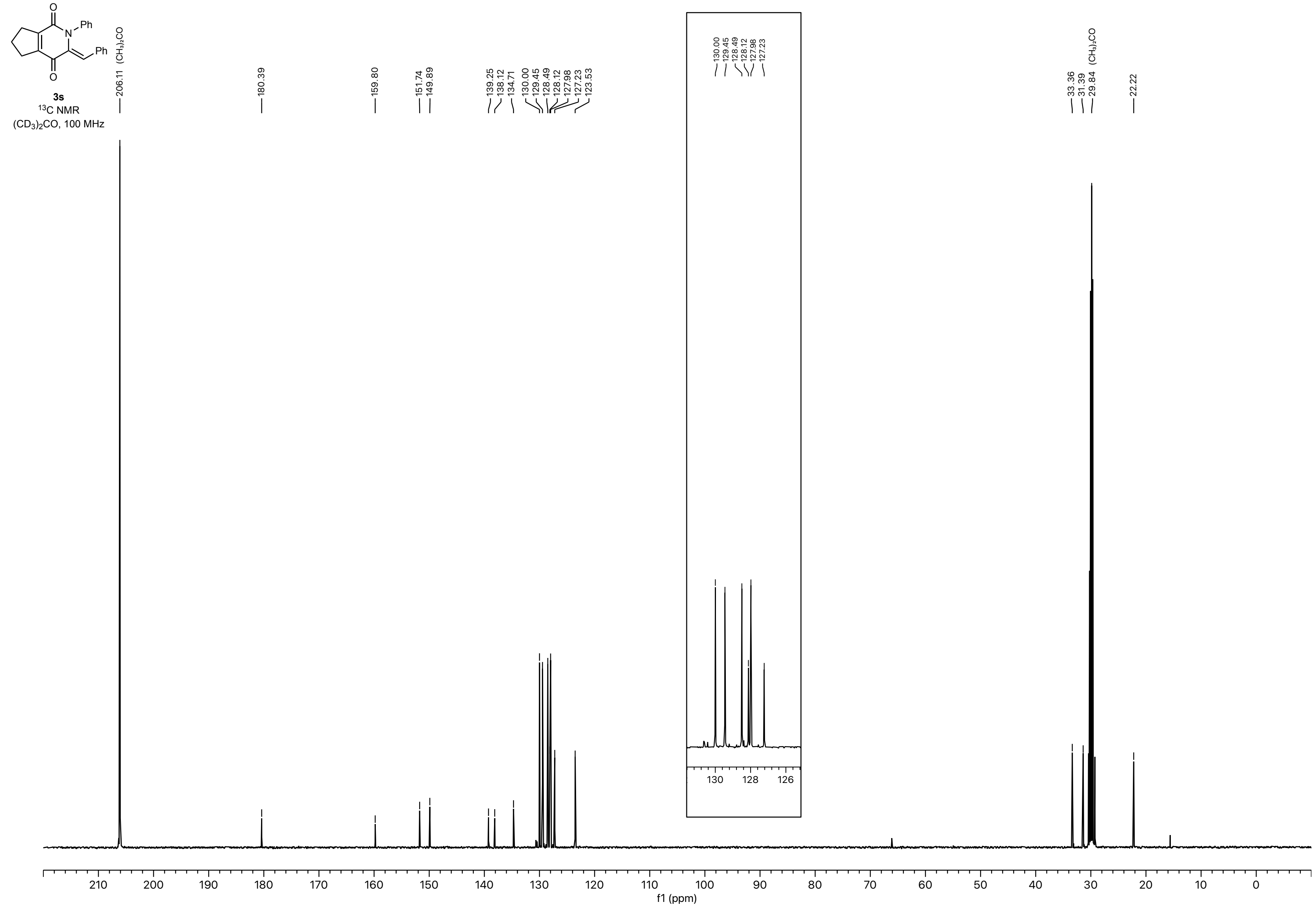

3s
^{13}C NMR
$(CD_3)_2CO$, 100 MHz
206.11 $(CH_3)_2CO$
180.39
159.80
151.74
149.89
139.25
138.12
134.71
130.00
129.45
128.49
128.12
127.98
127.23
123.53
33.36
31.39
29.84 $(CH_3)_2CO$
22.22
f1 (ppm)

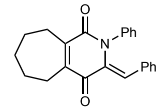

3t
^1H NMR
$CDCl_3$, 600 MHz

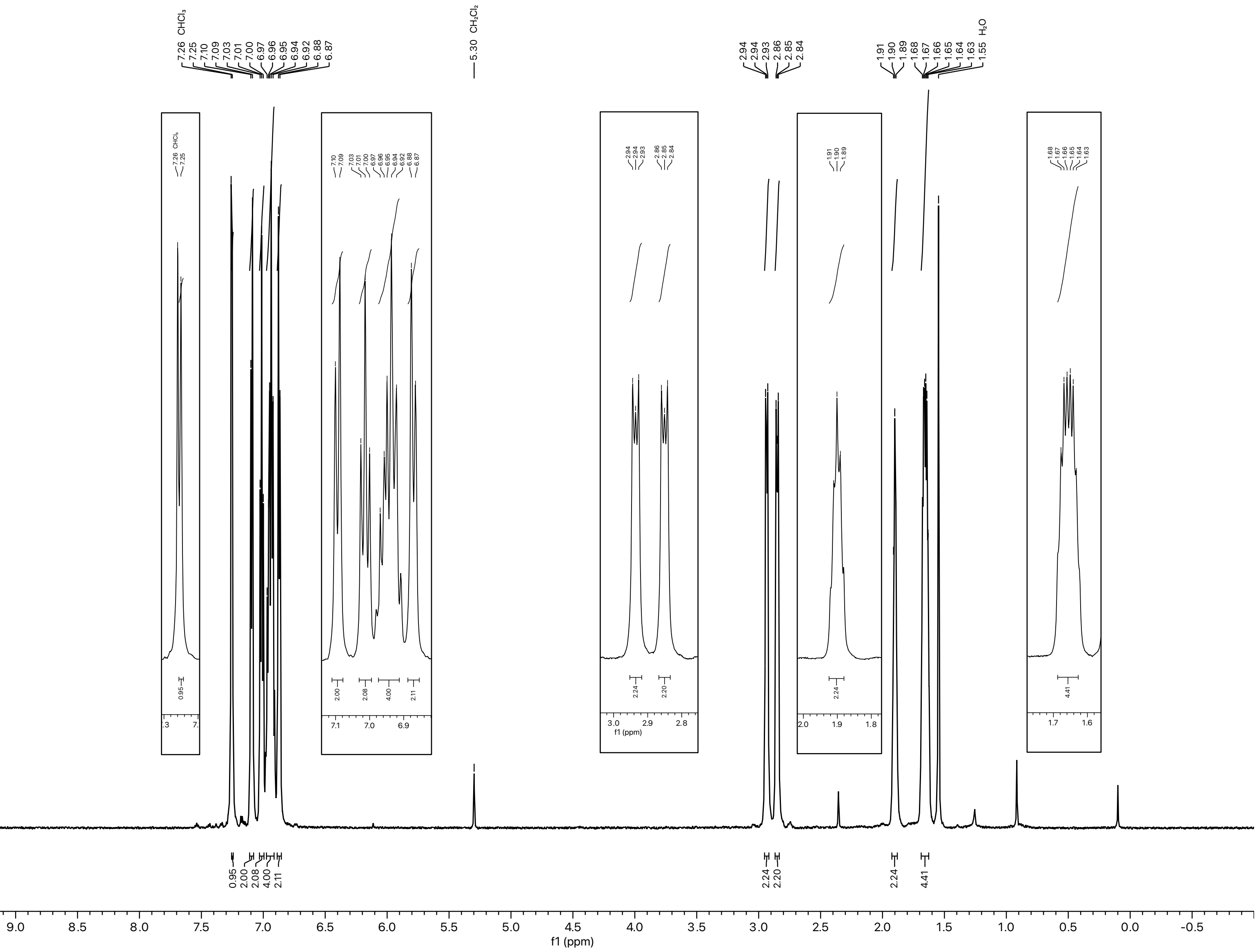

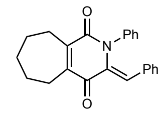

3t
^{13}C NMR
$CDCl_3$, 150 MHz

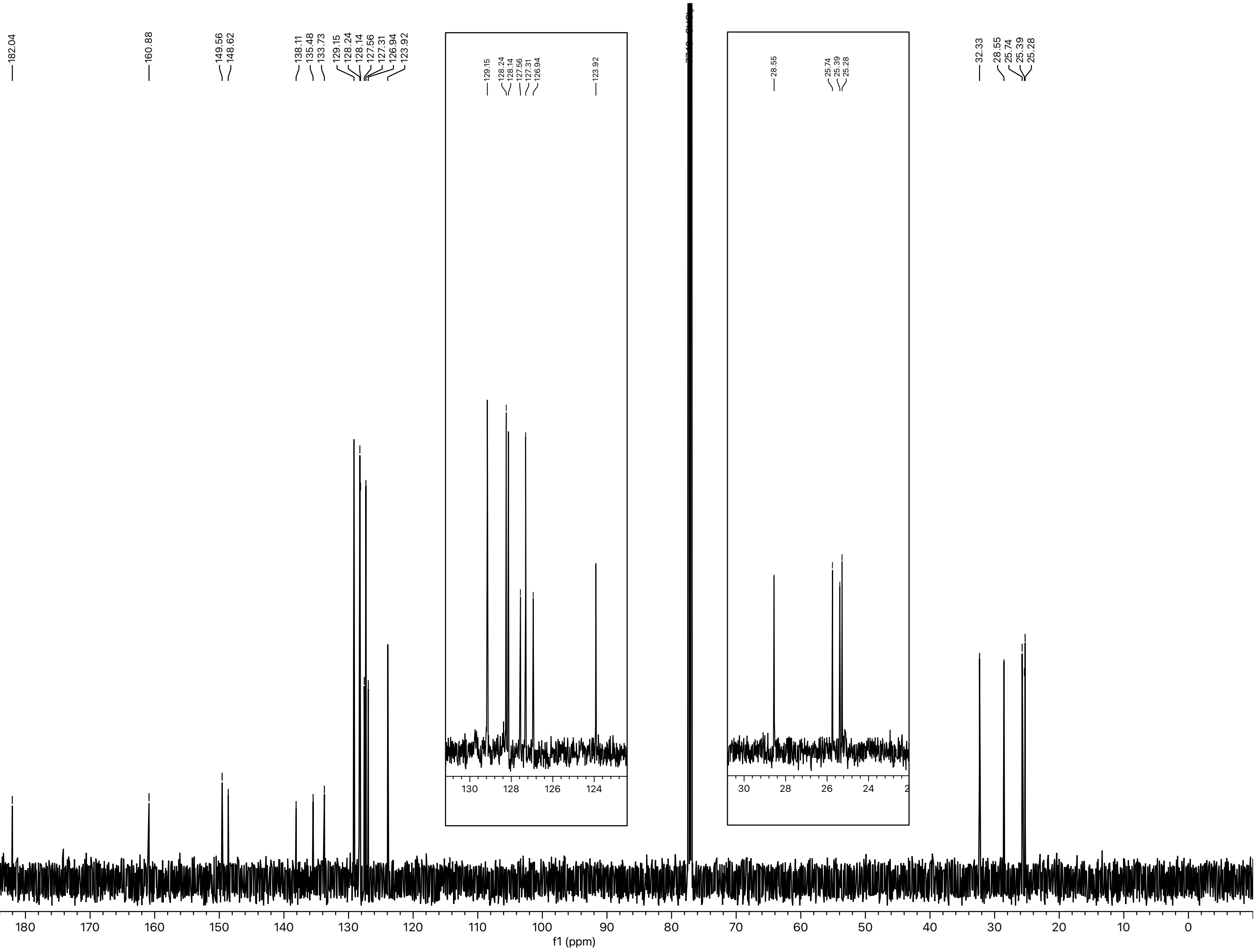

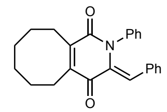

3u
^1H NMR
CD_2Cl_2, 600 MHz

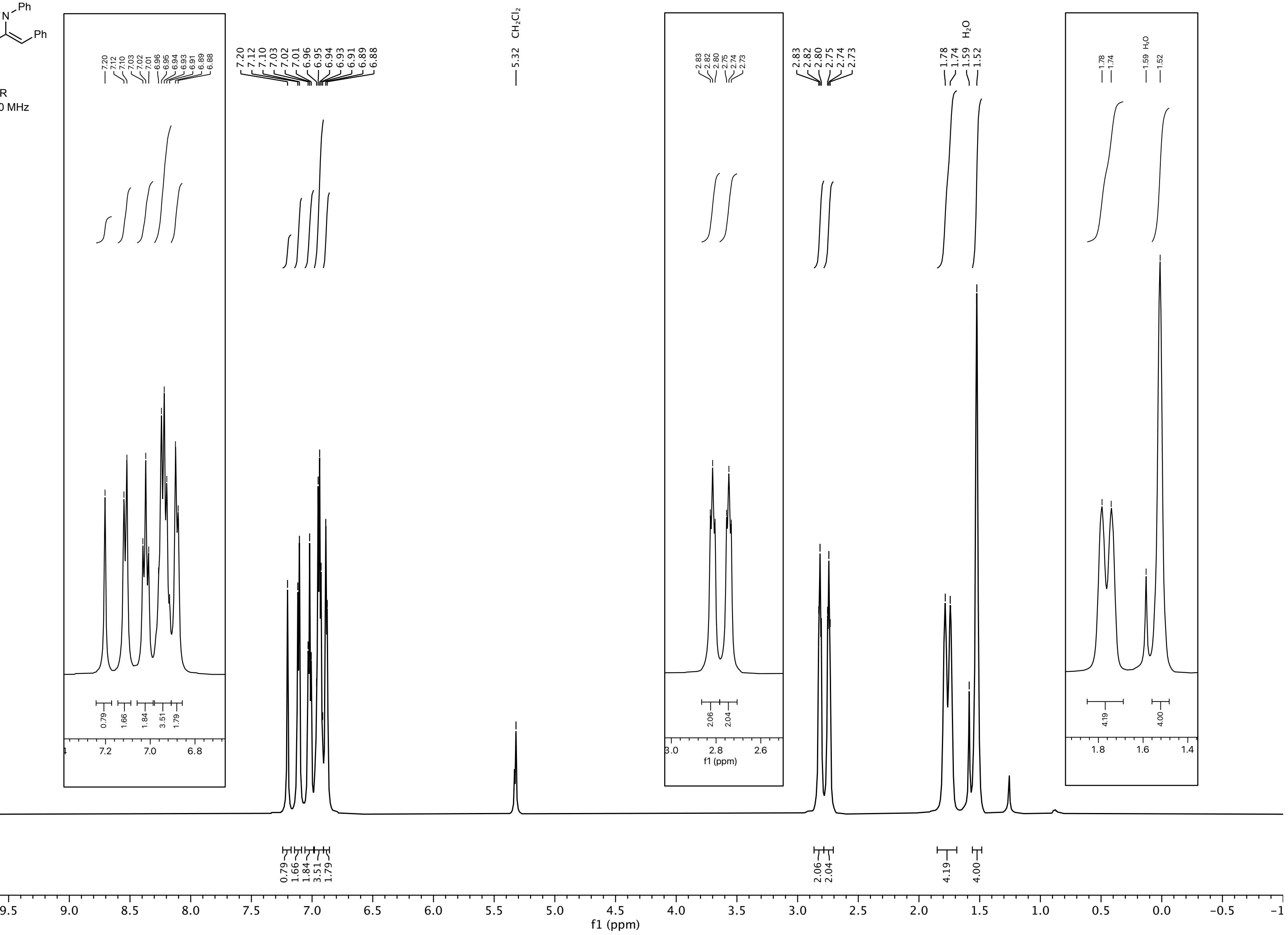

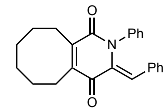
3u
^{13}C NMR
CD_2Cl_2, 100 MHz

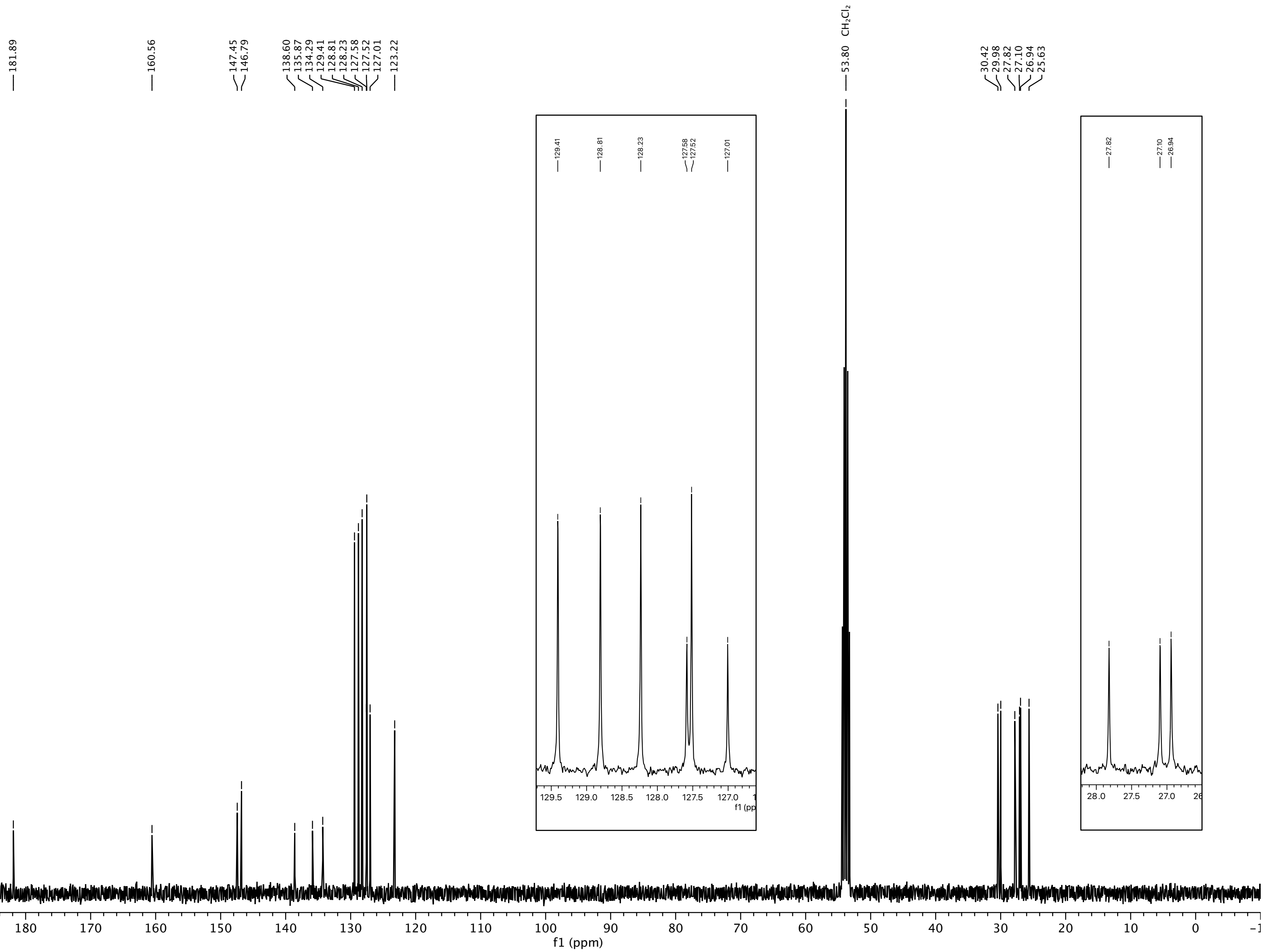

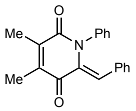

3v
^1H NMR
$CDCl_3$, 900 MHz

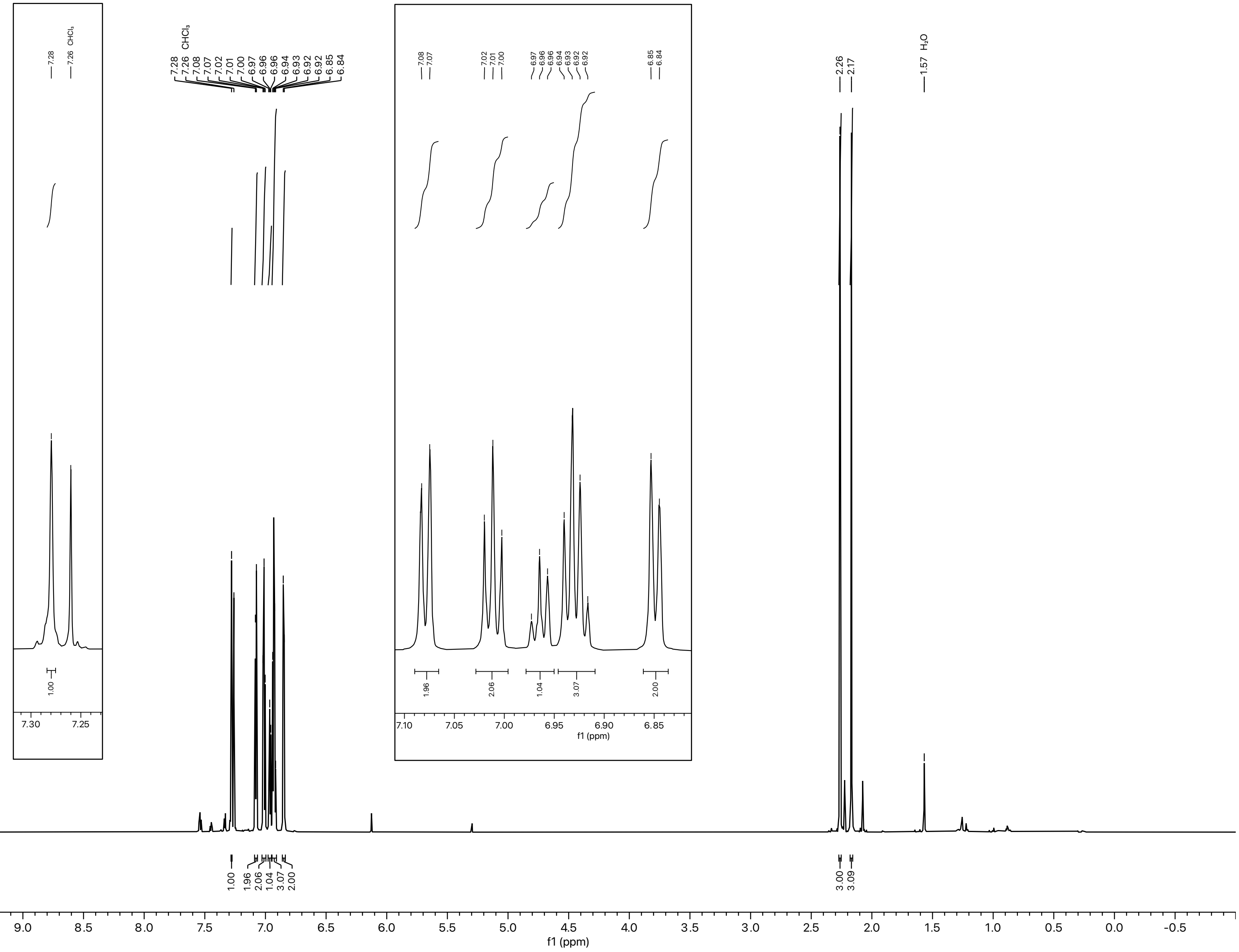

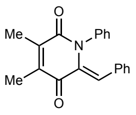

3v
^{13}C NMR
$CDCl_3$, 225 MHz

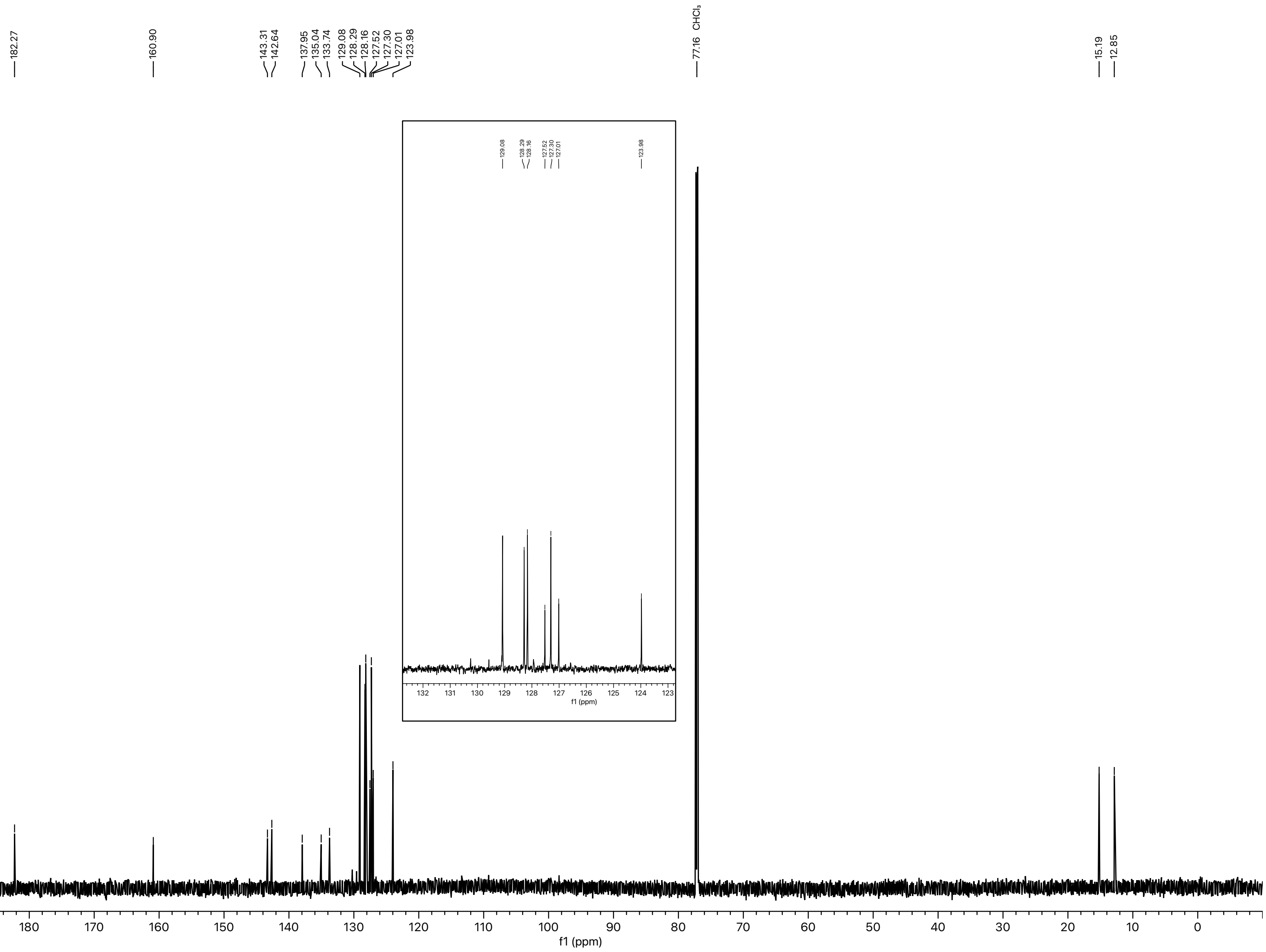

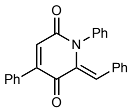

3w
^{1}H NMR
$CDCl_3$, 400 MHz

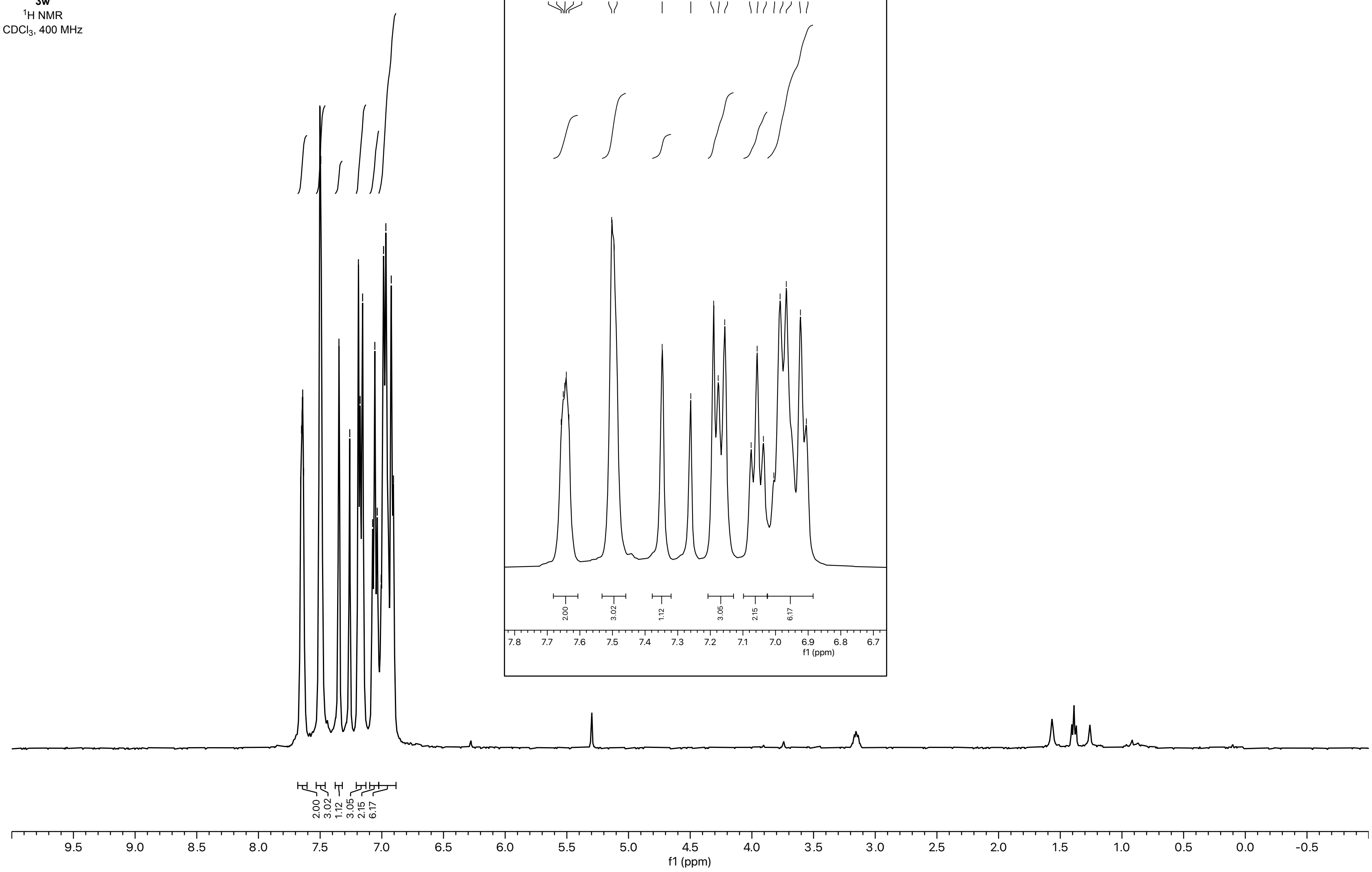

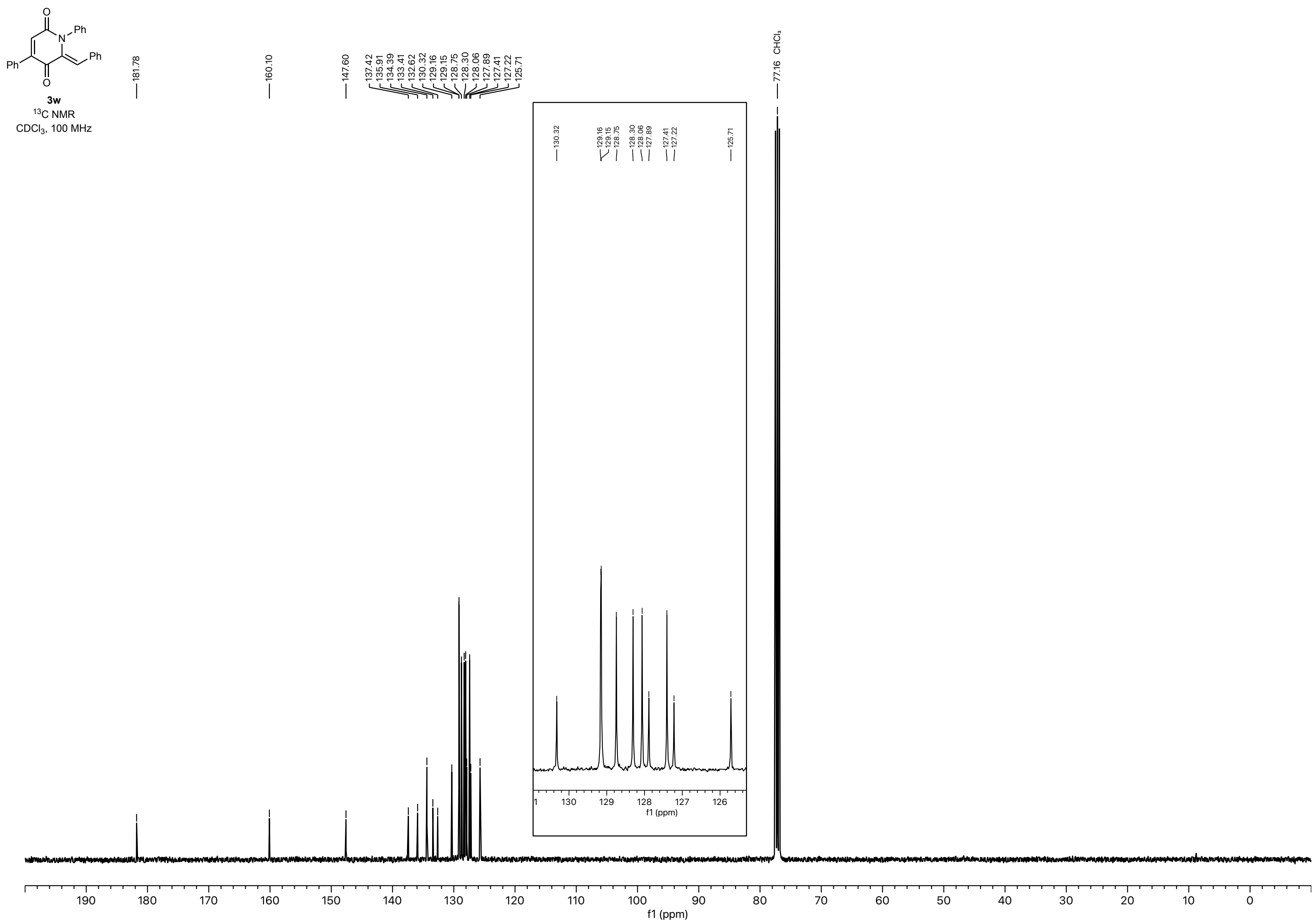
3w
^{13}C NMR
CDCl$_3$, 100 MHz
181.78
160.10
147.60
137.42
135.91
134.39
133.41
132.62
130.32
129.16
129.15
128.75
128.30
128.06
127.89
127.41
127.22
125.71
77.16 CHCl$_3$
f1 (ppm)

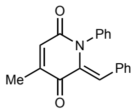

3x
^{1}H NMR
$CDCl_3$, 400 MHz

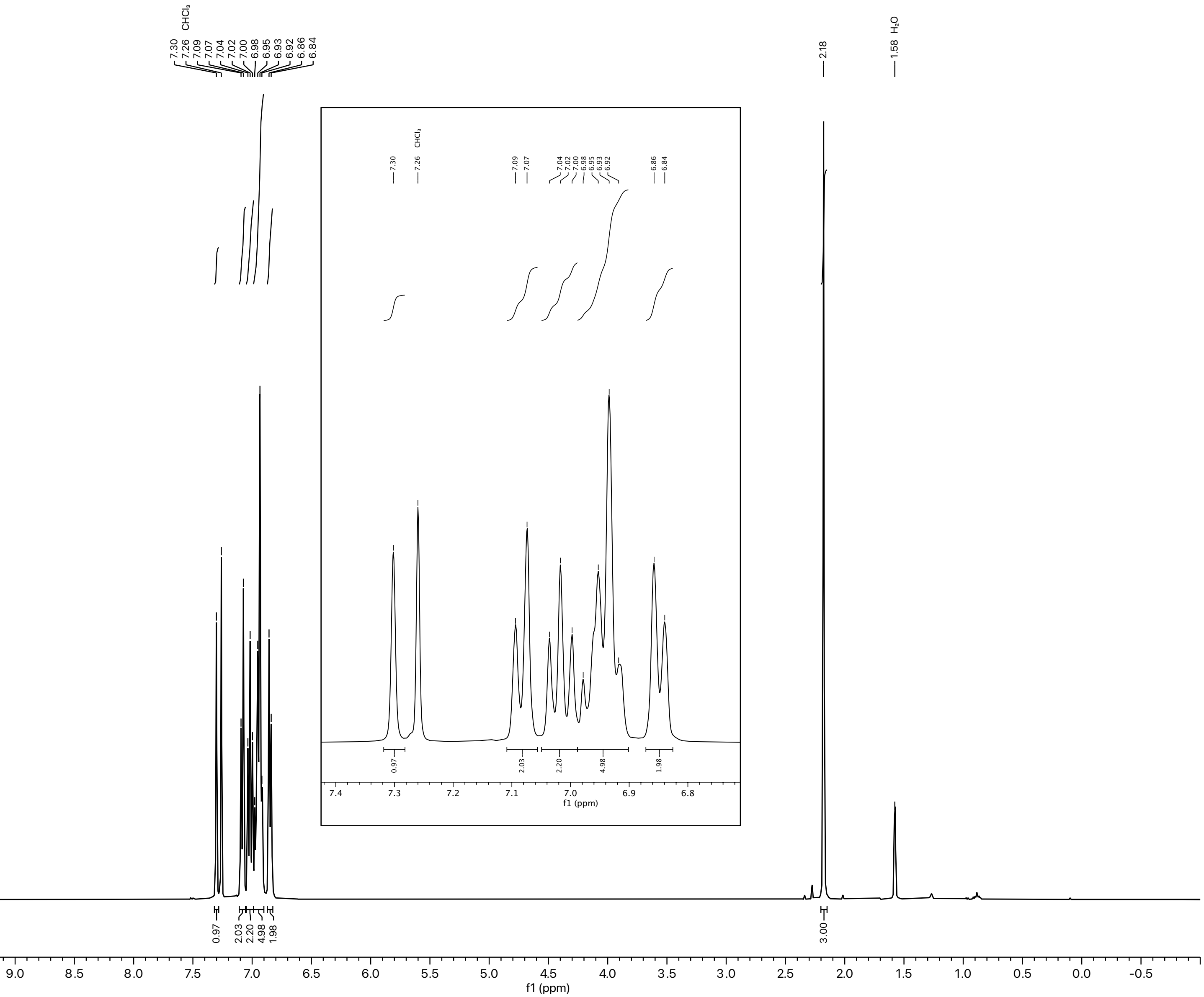

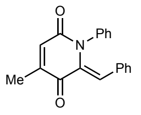

3x

^{13}C NMR

$CDCl_3$, 100 MHz

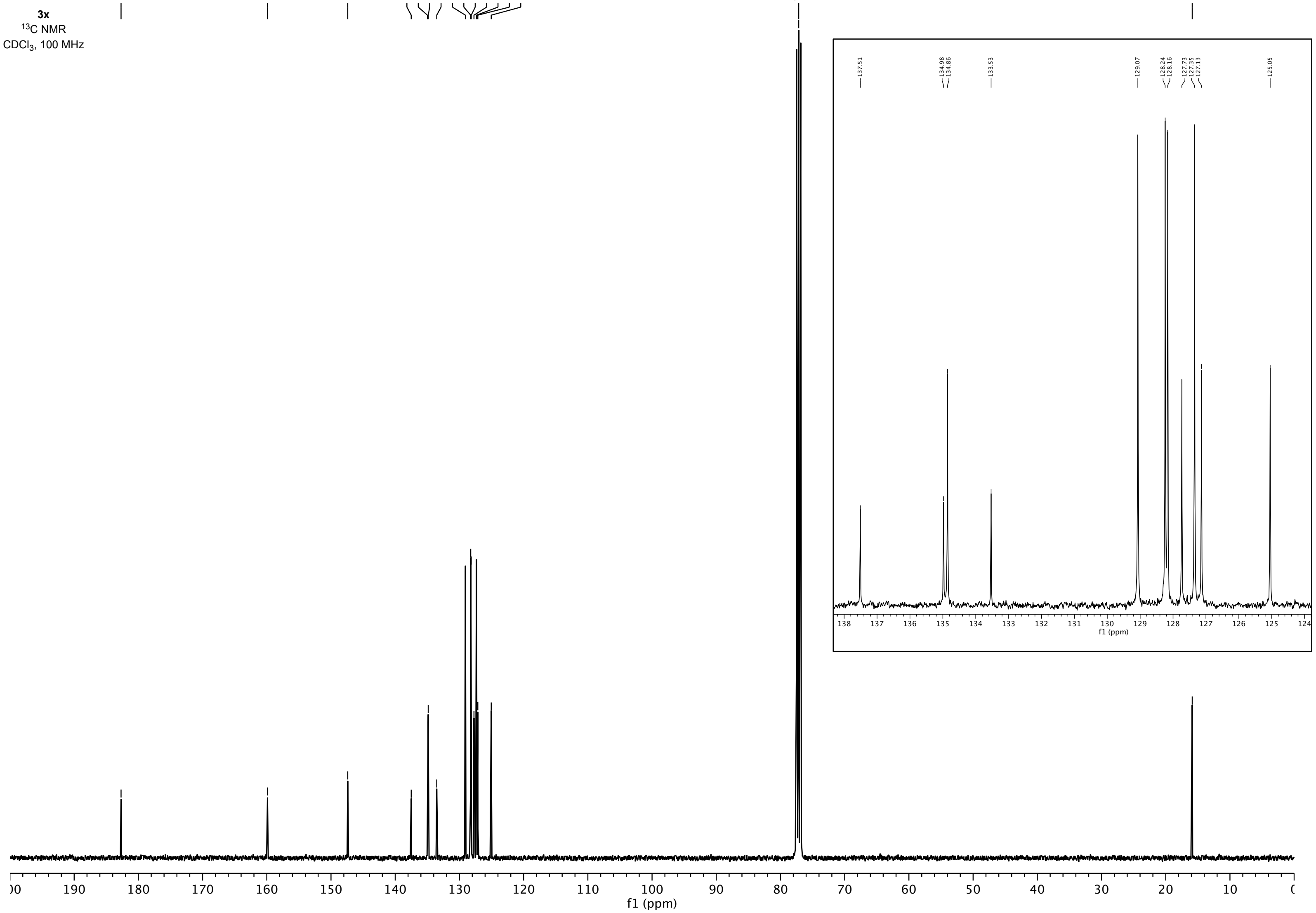

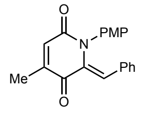

3y
^{1}H NMR
$CDCl_3$, 400 MHz

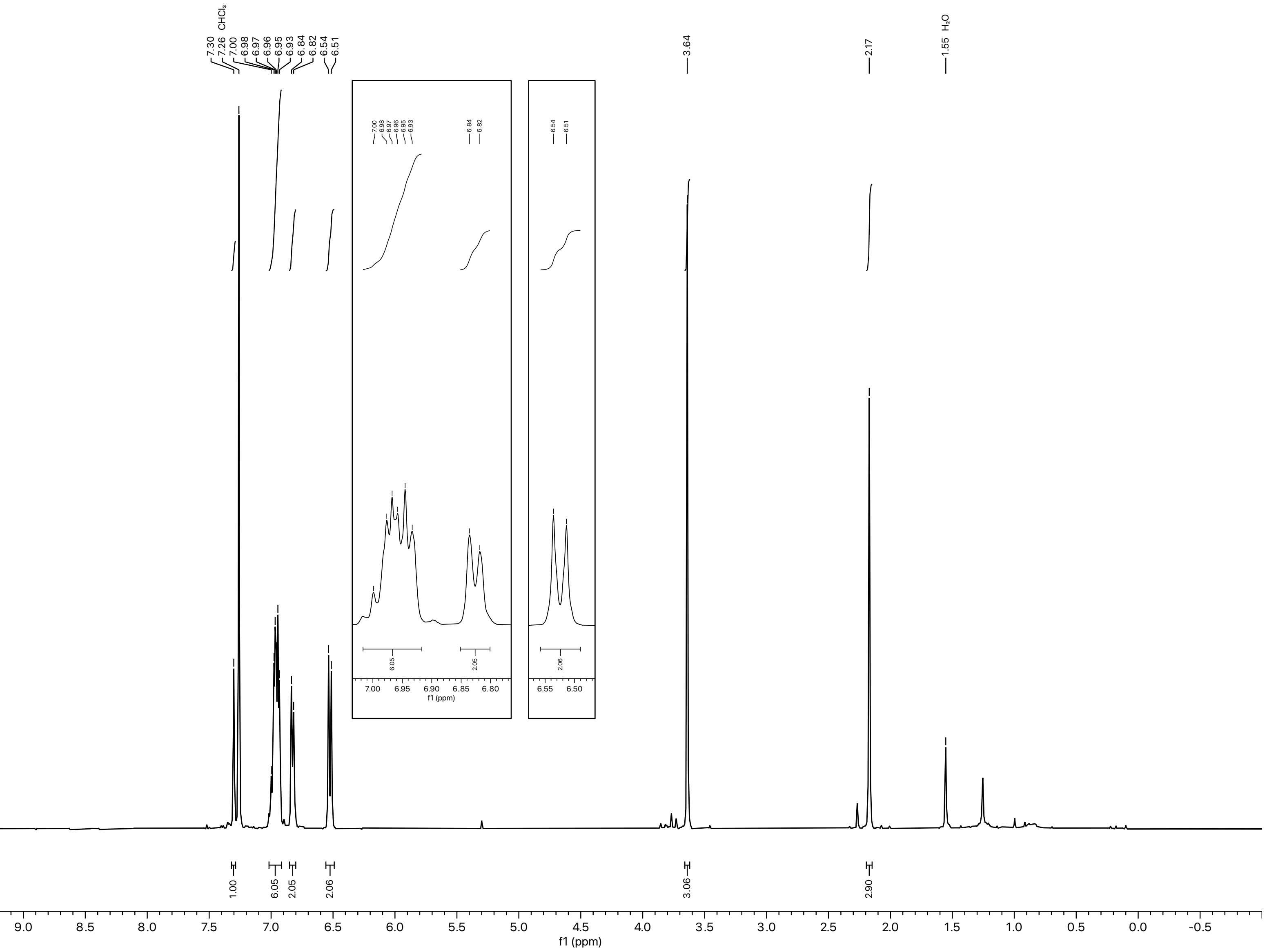

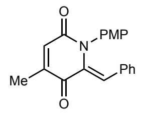

3y
^{13}C NMR
$CDCl_3$, 100 MHz

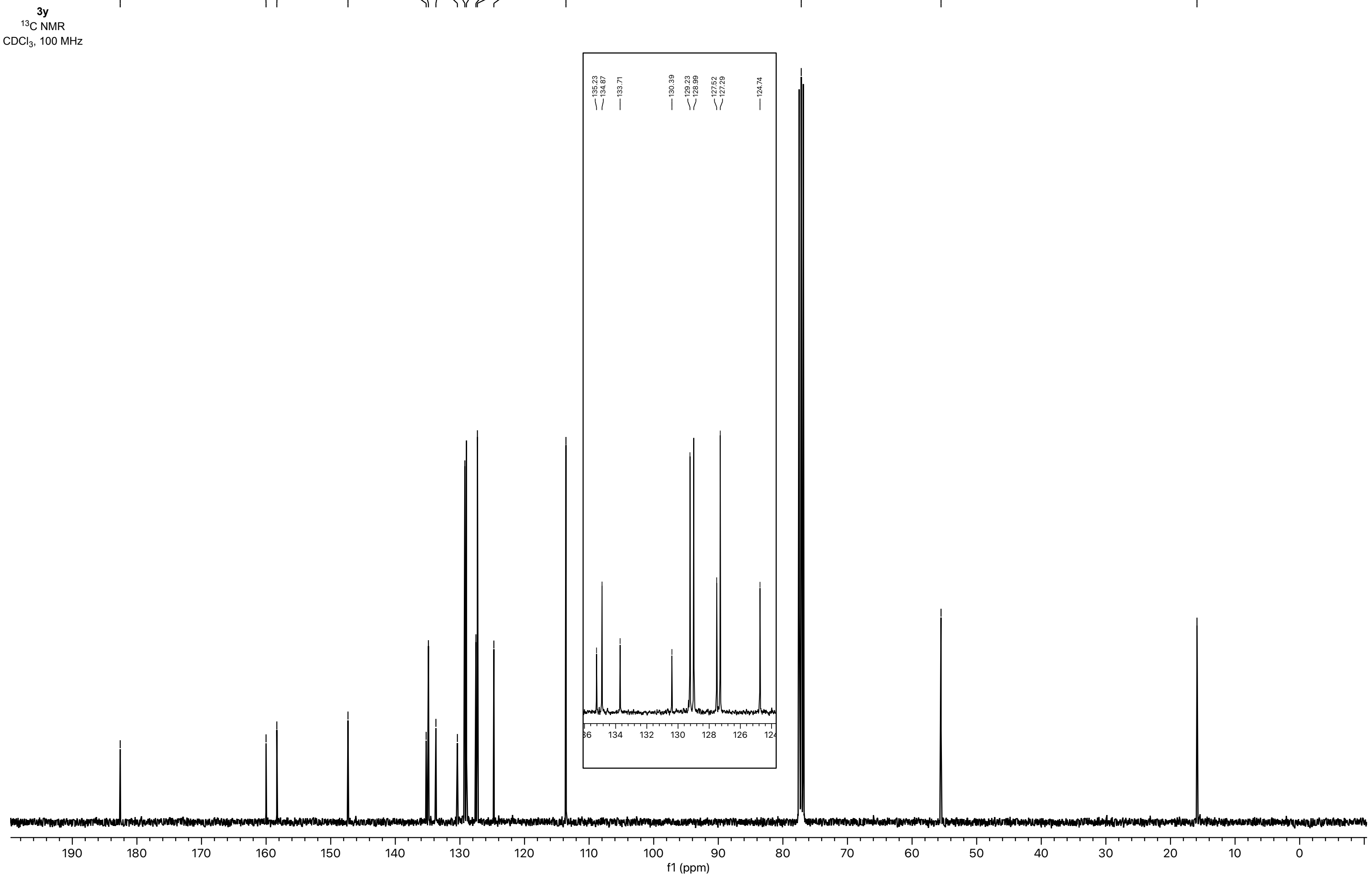

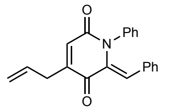

3z
^{1}H NMR
$CDCl_3$, 600 MHz

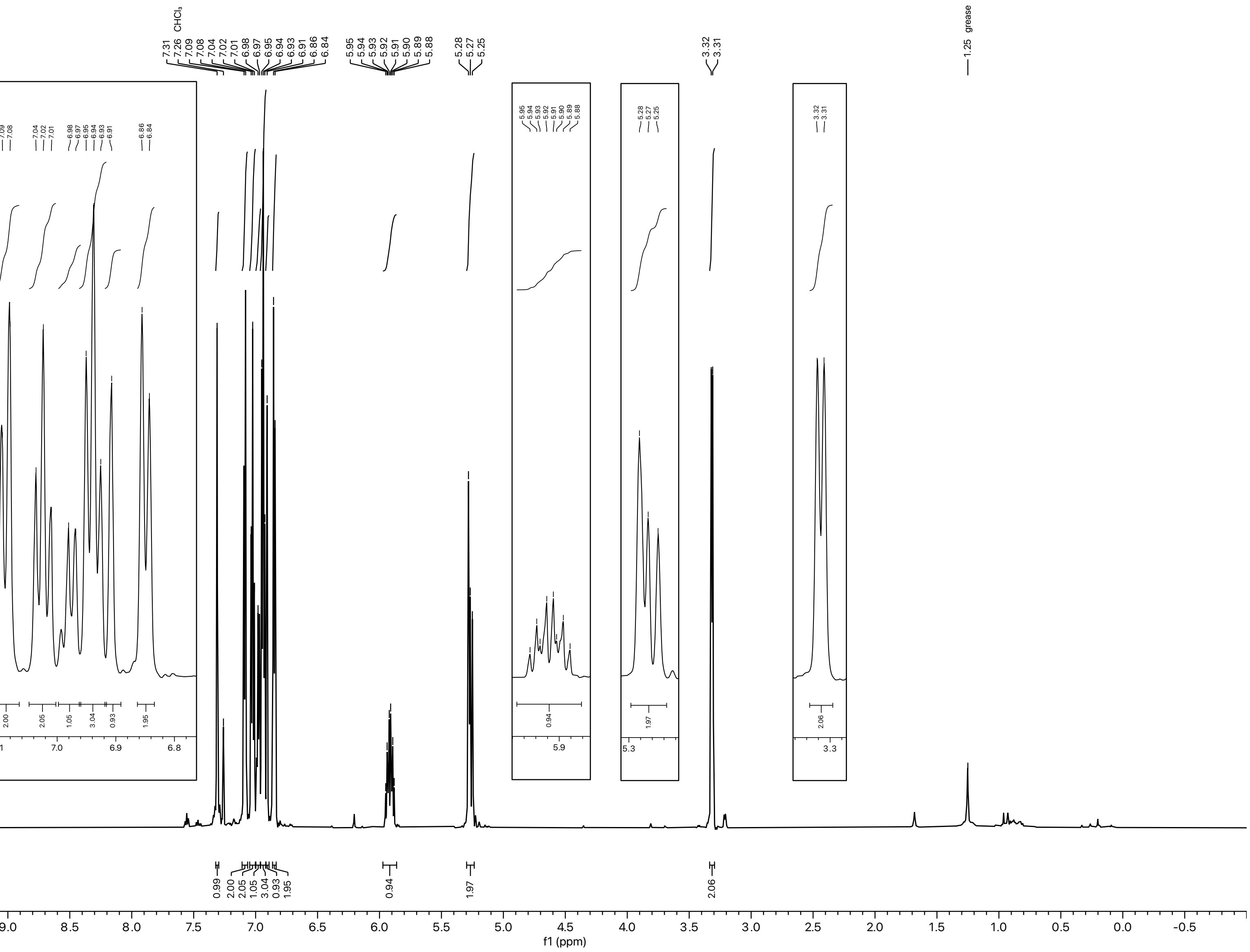

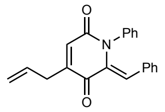

3z

^{13}C NMR

$CDCl_3$, 150 MHz

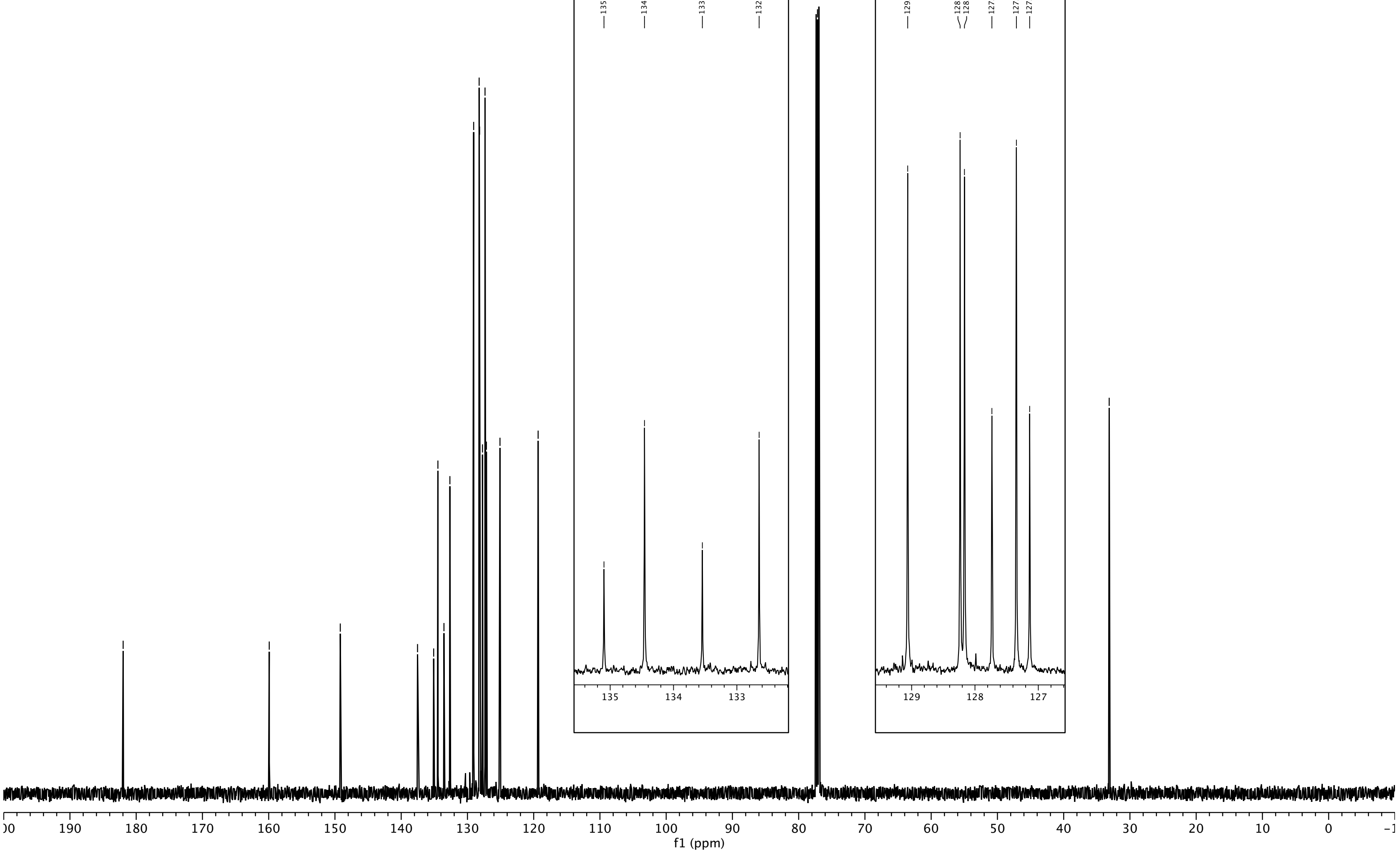

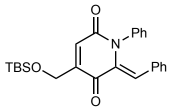

3aa
^{1}H NMR
$CDCl_3$, 600 MHz

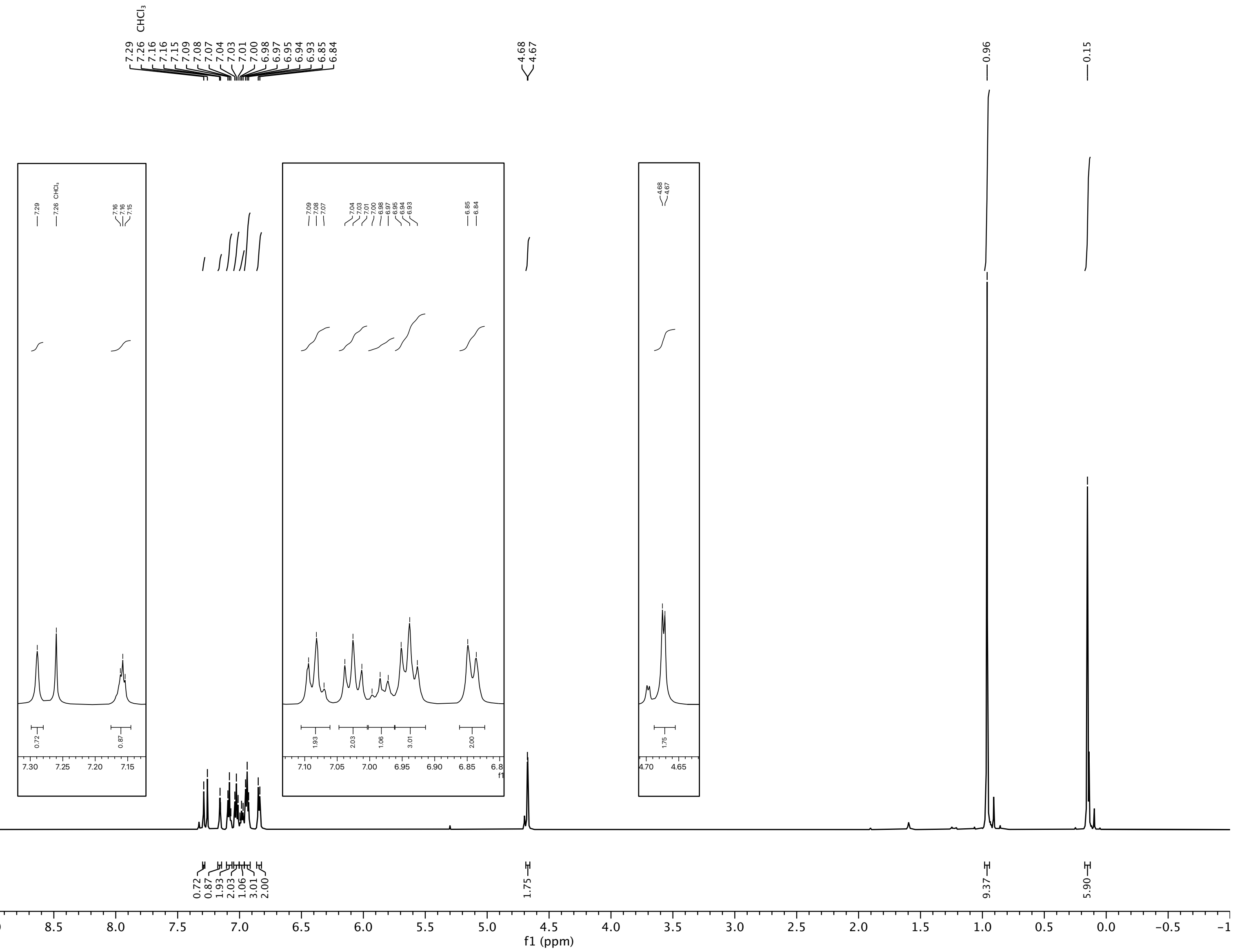

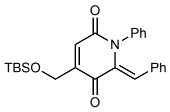

3aa
^{13}C NMR
$CDCl_3$, 100 MHz

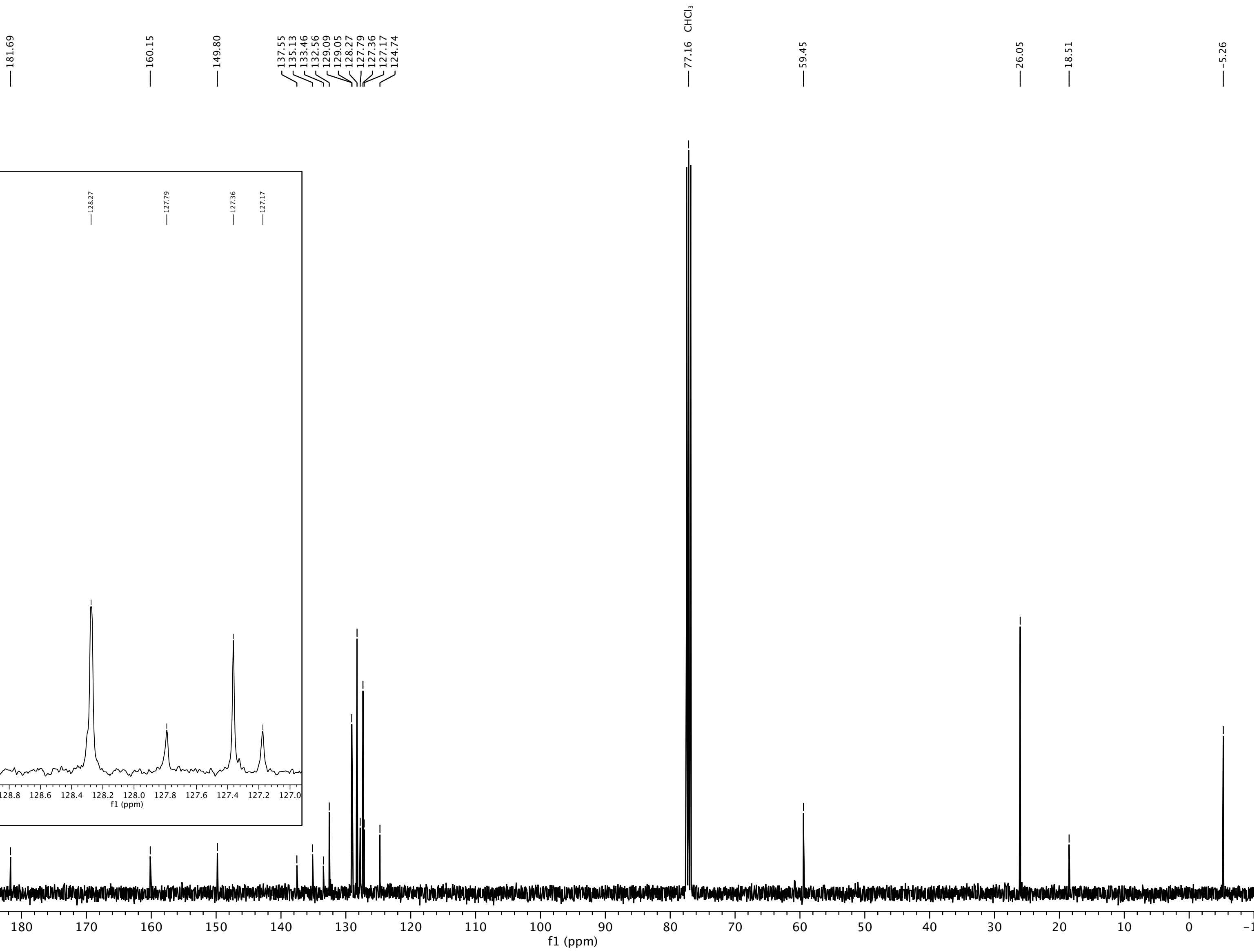

Derivatization Studies

Allylic Transposition for the Synthesis of 2-Pyridones

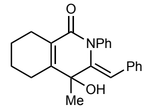

14

^1H NMR

$CDCl_3$, 600 MHz

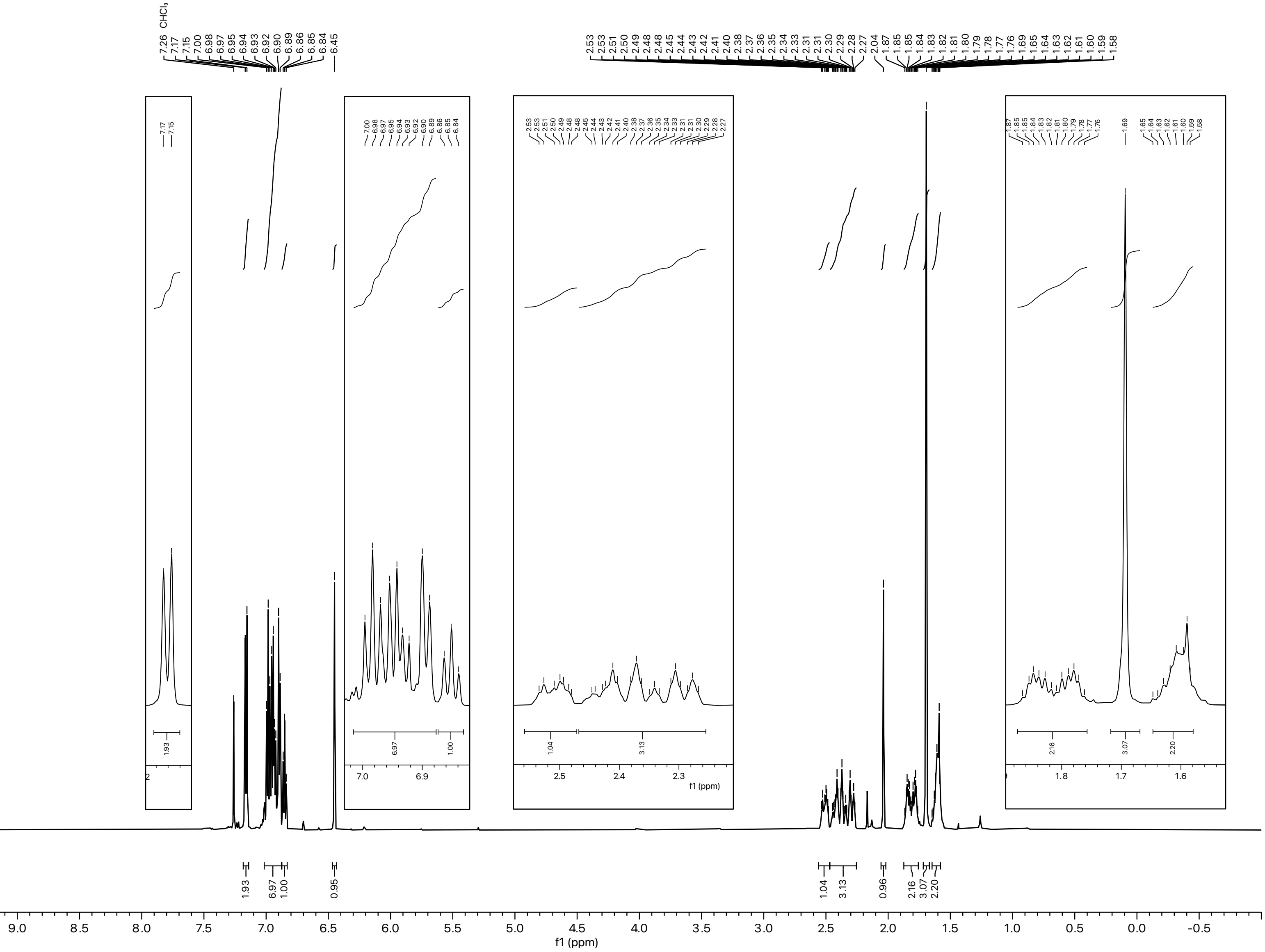

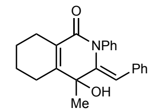

14
^{13}C NMR
$CDCl_3$, 150 MHz

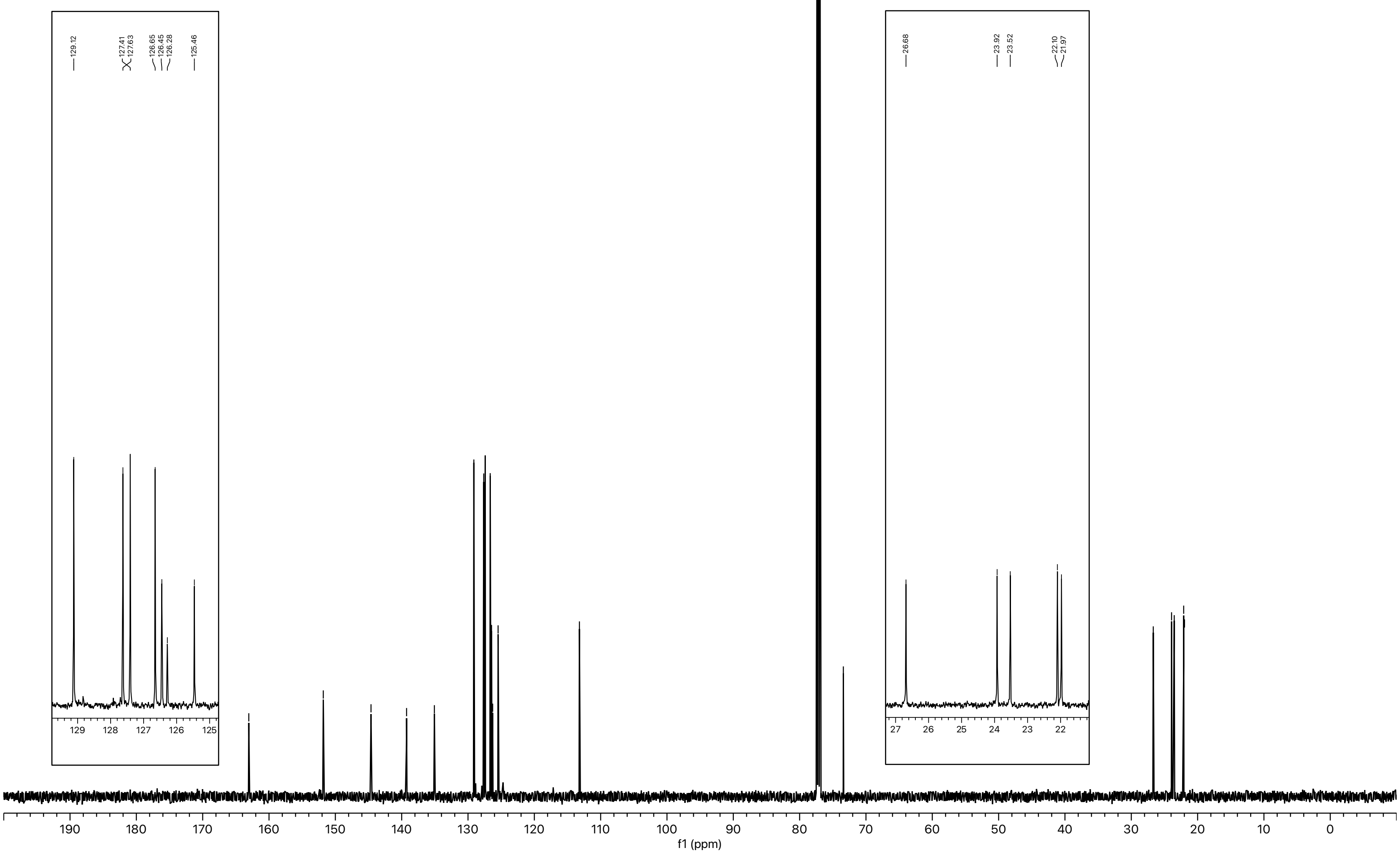

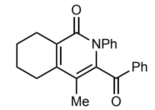

15
^{1}H NMR
$CDCl_3$, 600 MHz

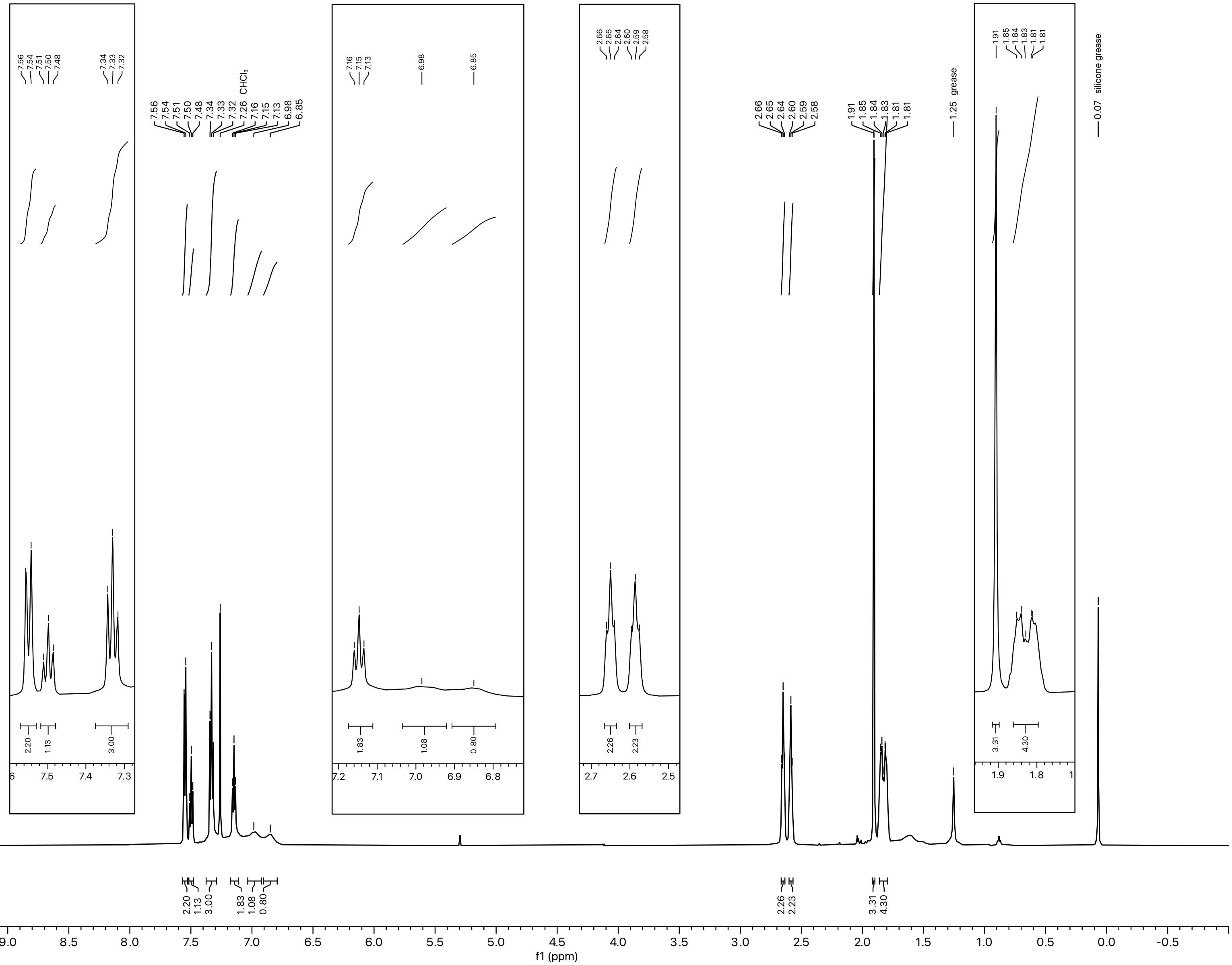

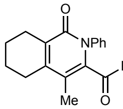

15
^{13}C NMR
$CDCl_3$, 100 MHz

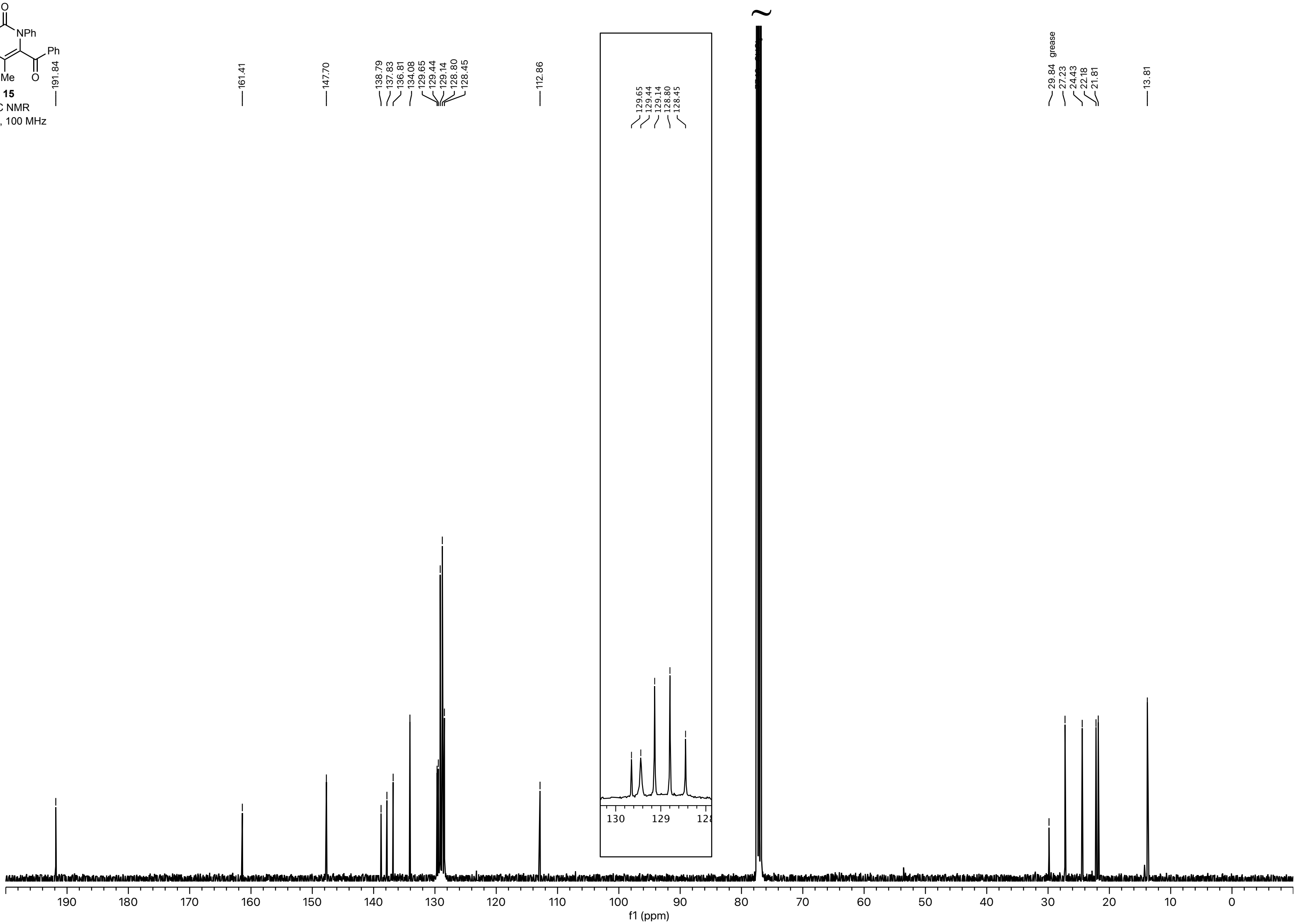

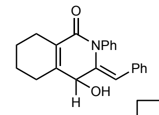

16
^1H NMR
$(CD_3)_2SO$, 600 MHz

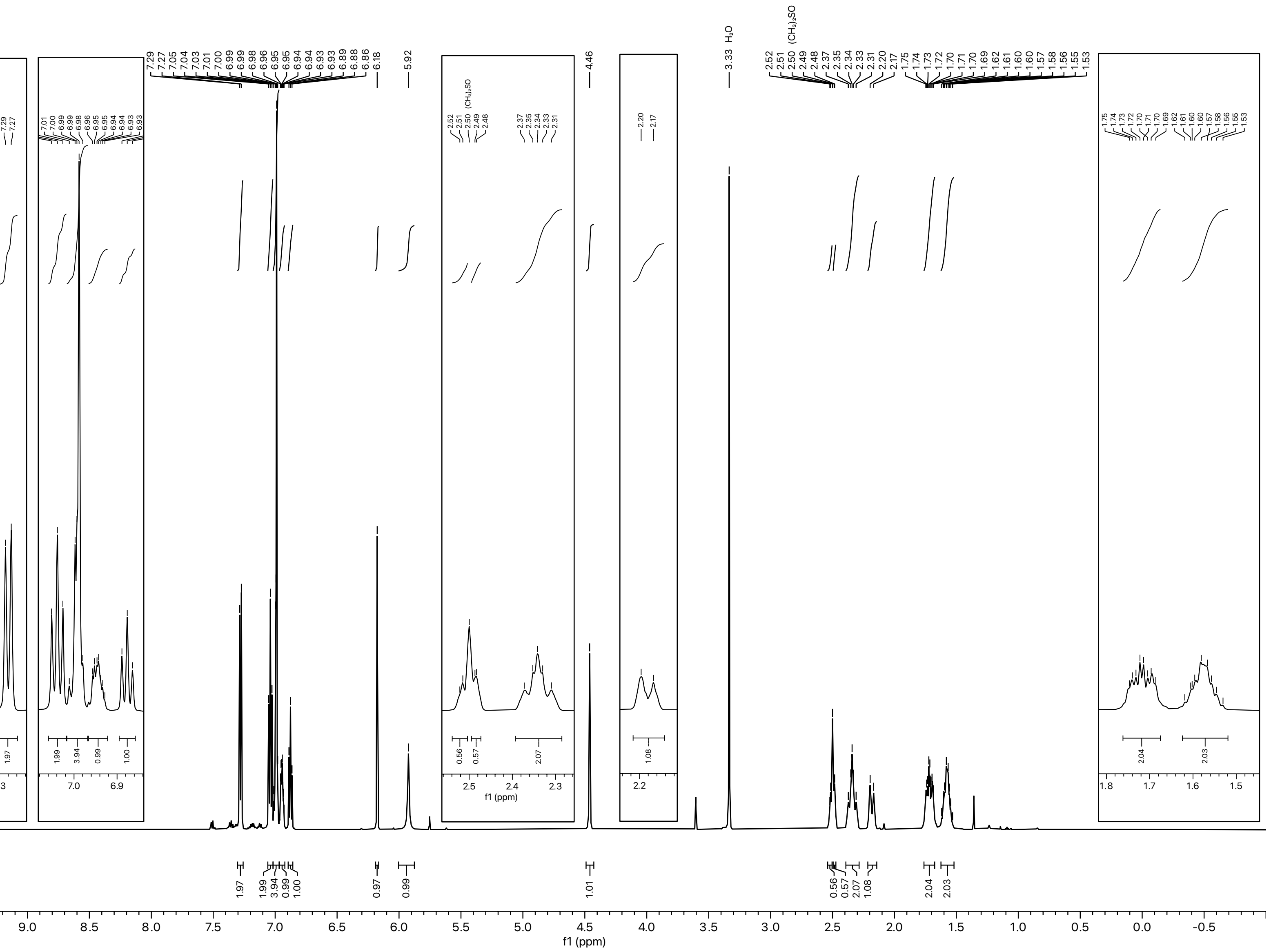

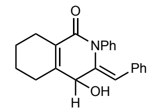

16
^{13}C NMR
$(CD_3)_2SO$, 150 MHz

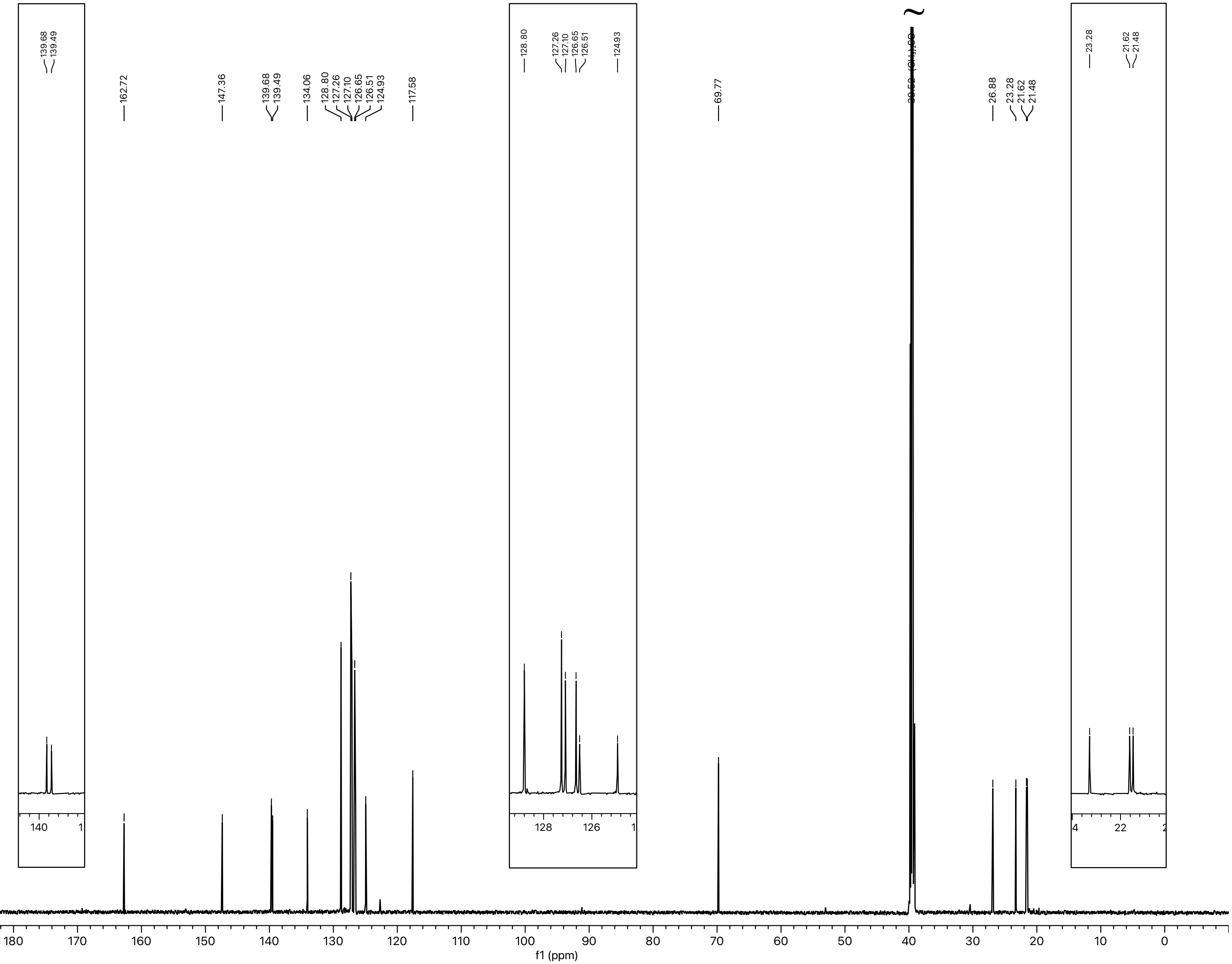

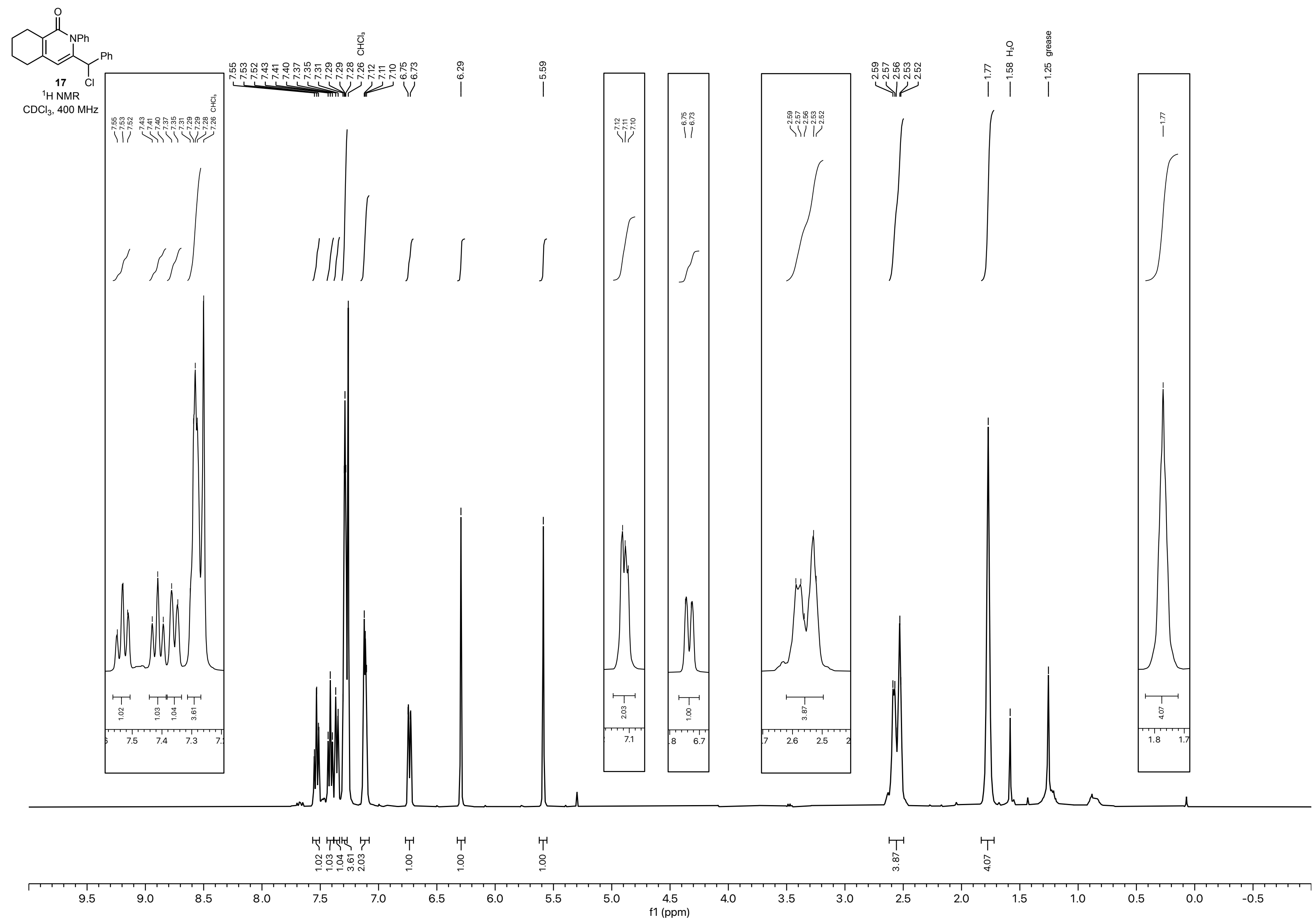

17
^{1}H NMR
$CDCl_3$, 400 MHz
NPh
Ph
Cl
O
7.55
7.53
7.52
7.43
7.41
7.40
7.37
7.35
7.31
7.29
7.29
7.28
7.26
$CHCl_3$
7.12
7.11
7.10
6.75
6.73
6.29
5.59
2.59
2.57
2.56
2.53
2.52
1.77
1.58 H_2O
1.25 grease
1.02
1.03
1.04
3.61
2.03
1.00
1.00
1.00
3.87
4.07
f1 (ppm)

17
^{13}C NMR
$CDCl_3$, 100 MHz

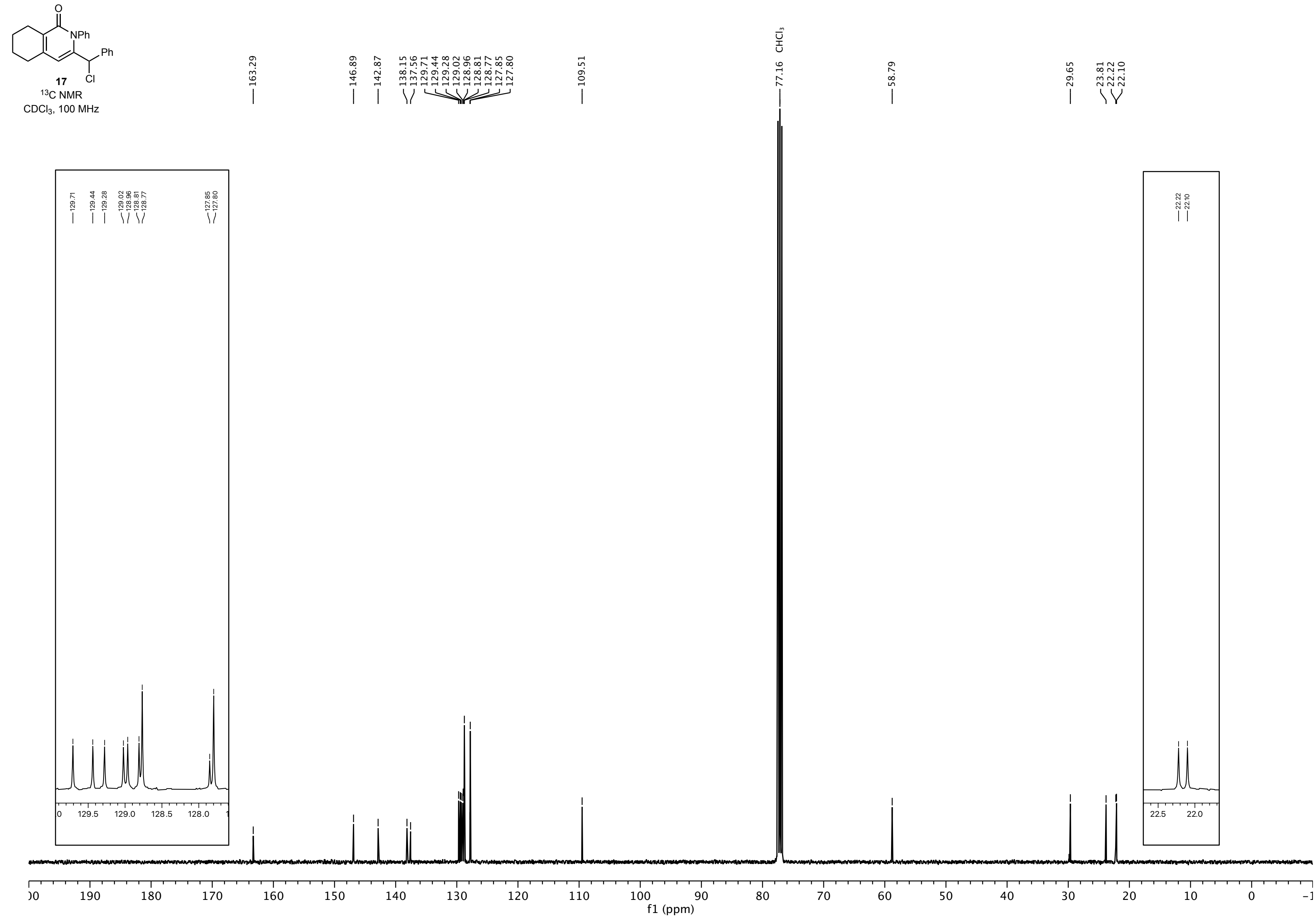

N-Aryl Ketenimines as Synthetic Equivalents of Other Aza-Dienophiles

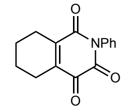

18
^{1}H NMR
$CDCl_3$, 400 MHz

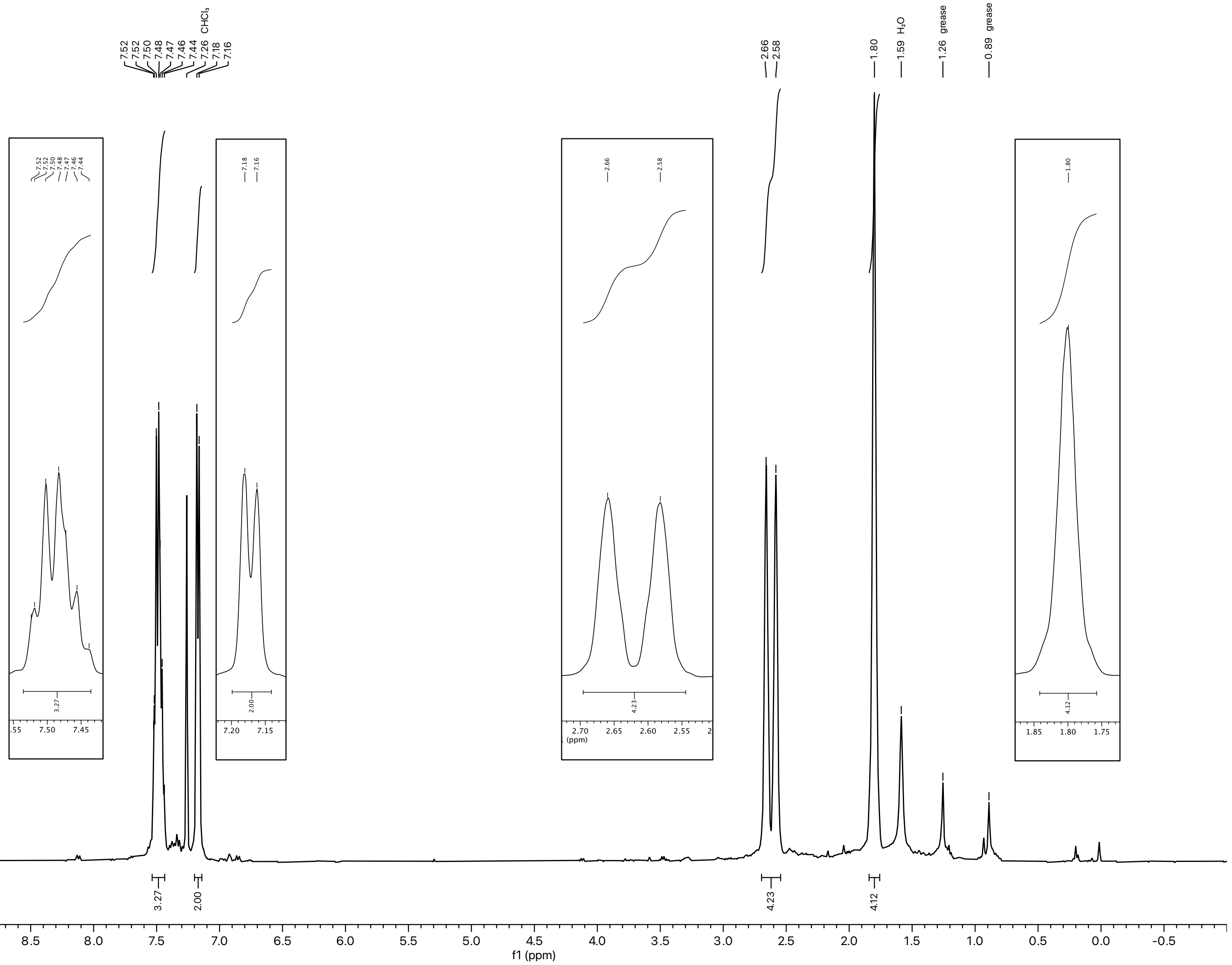

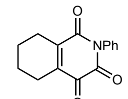

18
^{13}C NMR
$CDCl_3$, 100 MHz

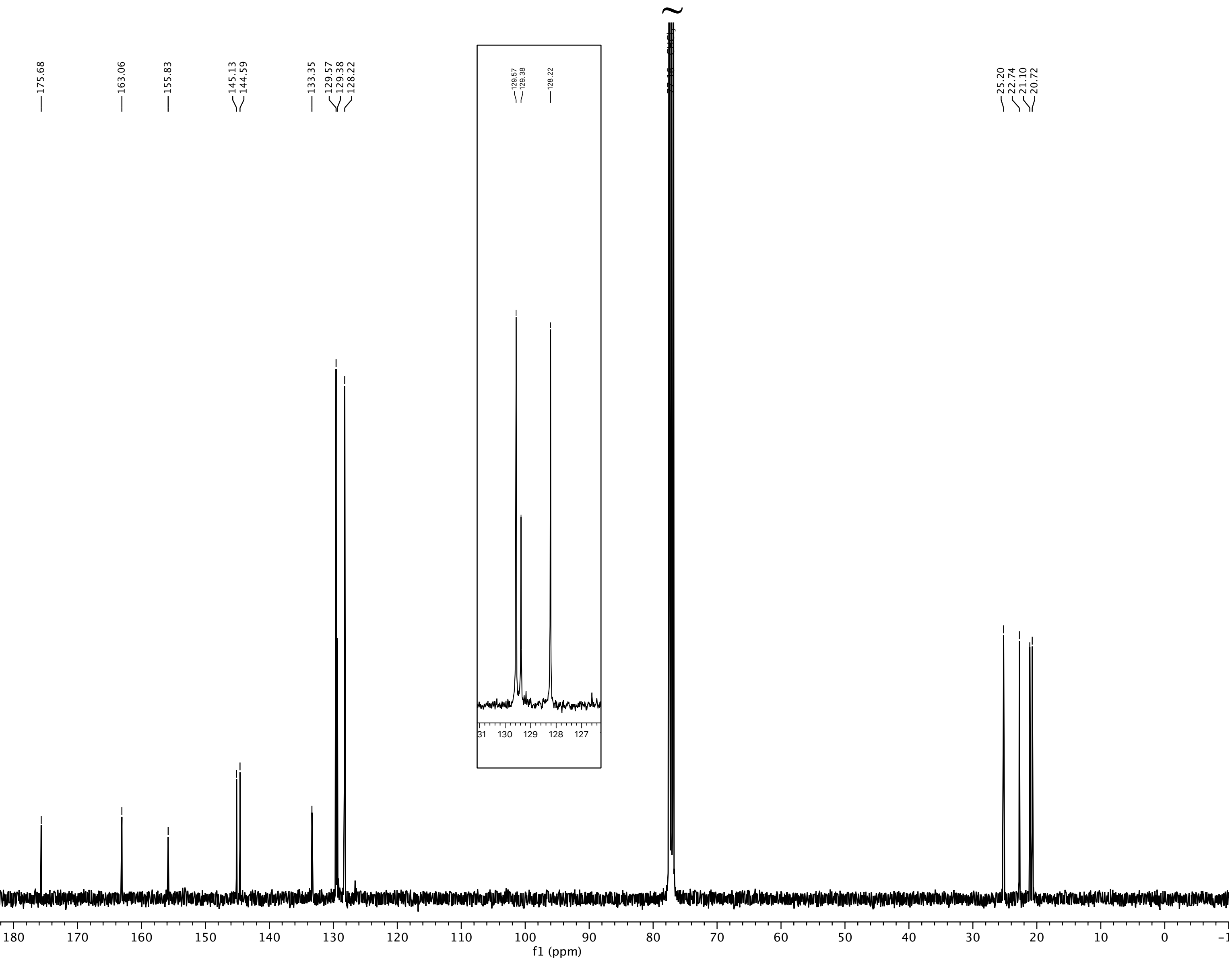

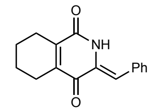

19
^{1}H NMR
$CDCl_3$, 400 MHz

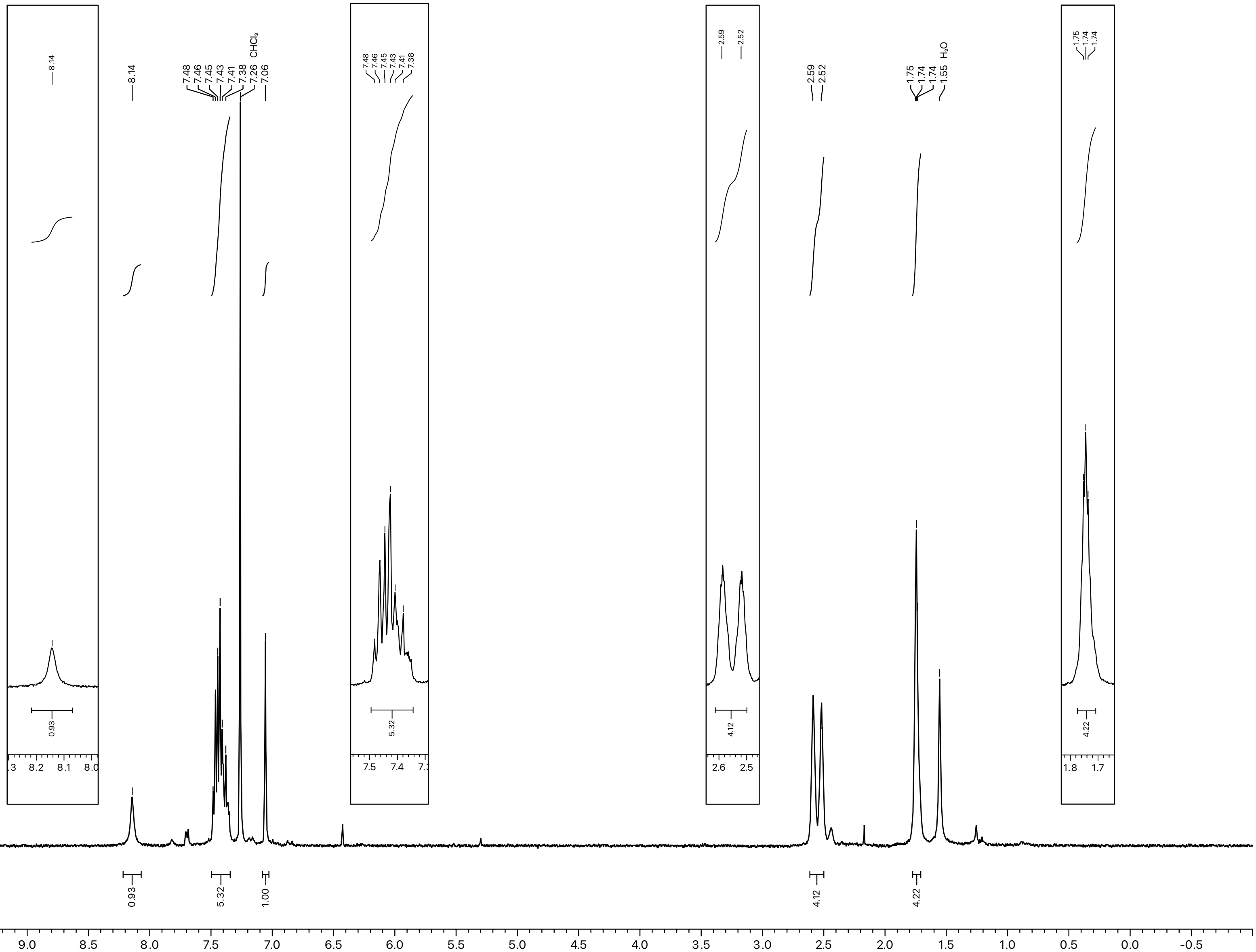

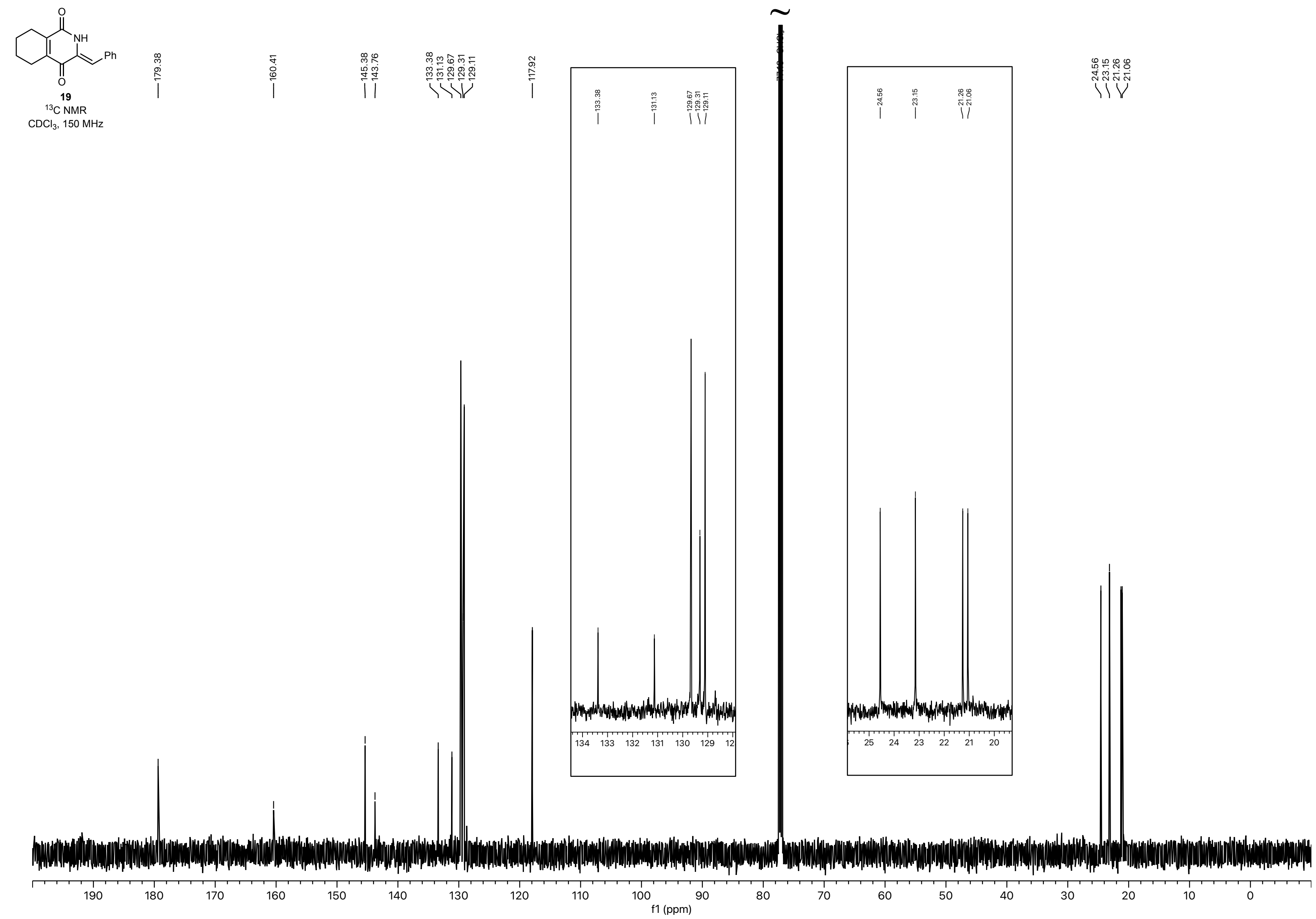
19
^{13}C NMR
$CDCl_3$, 150 MHz
179.38
160.41
145.38
143.76
133.38
131.13
129.67
129.31
129.11
117.92
24.56
23.15
21.26
21.06
f1 (ppm)

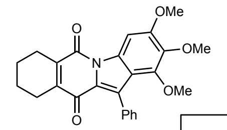

20
^1H NMR
$CDCl_3$, 900 MHz

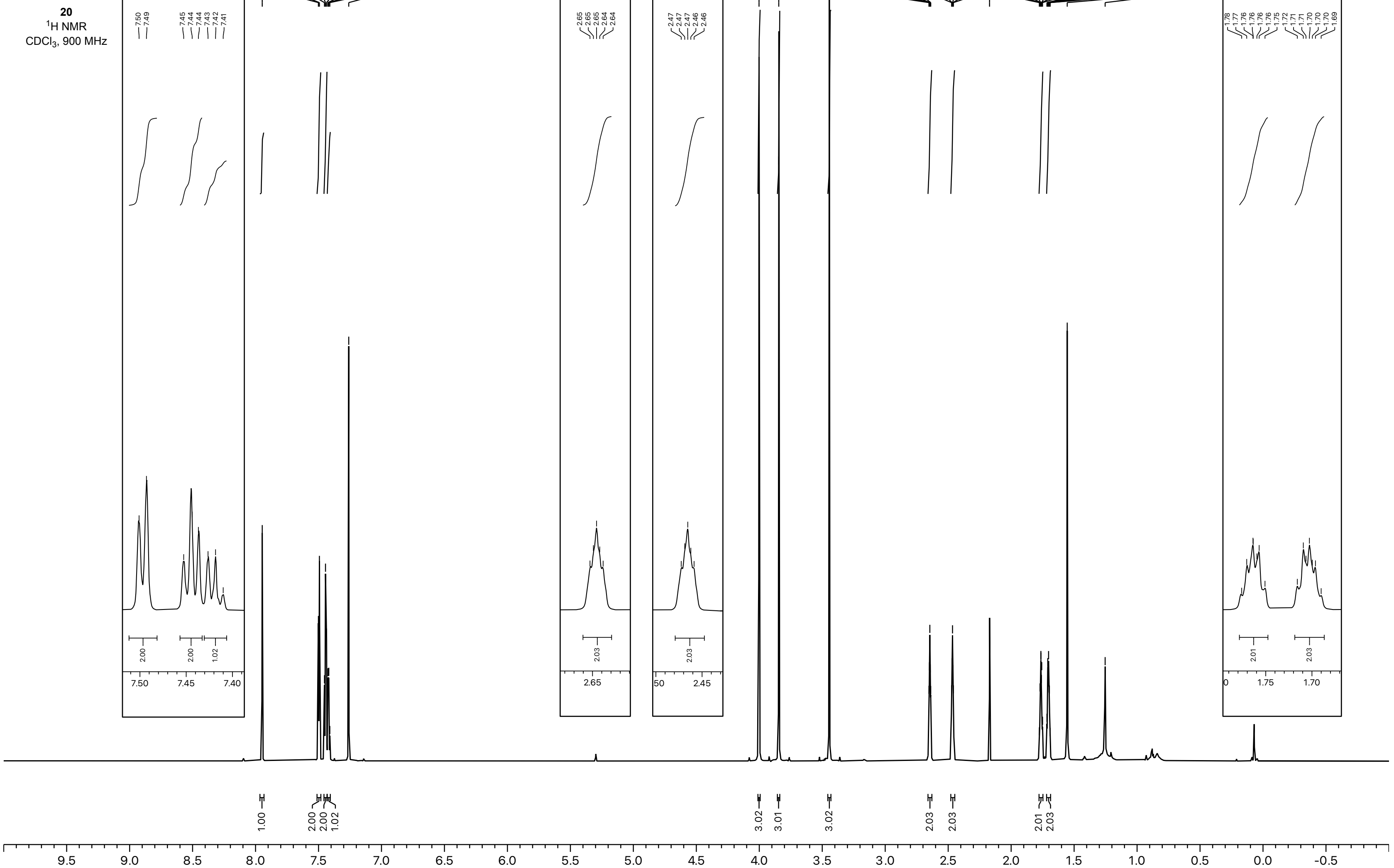

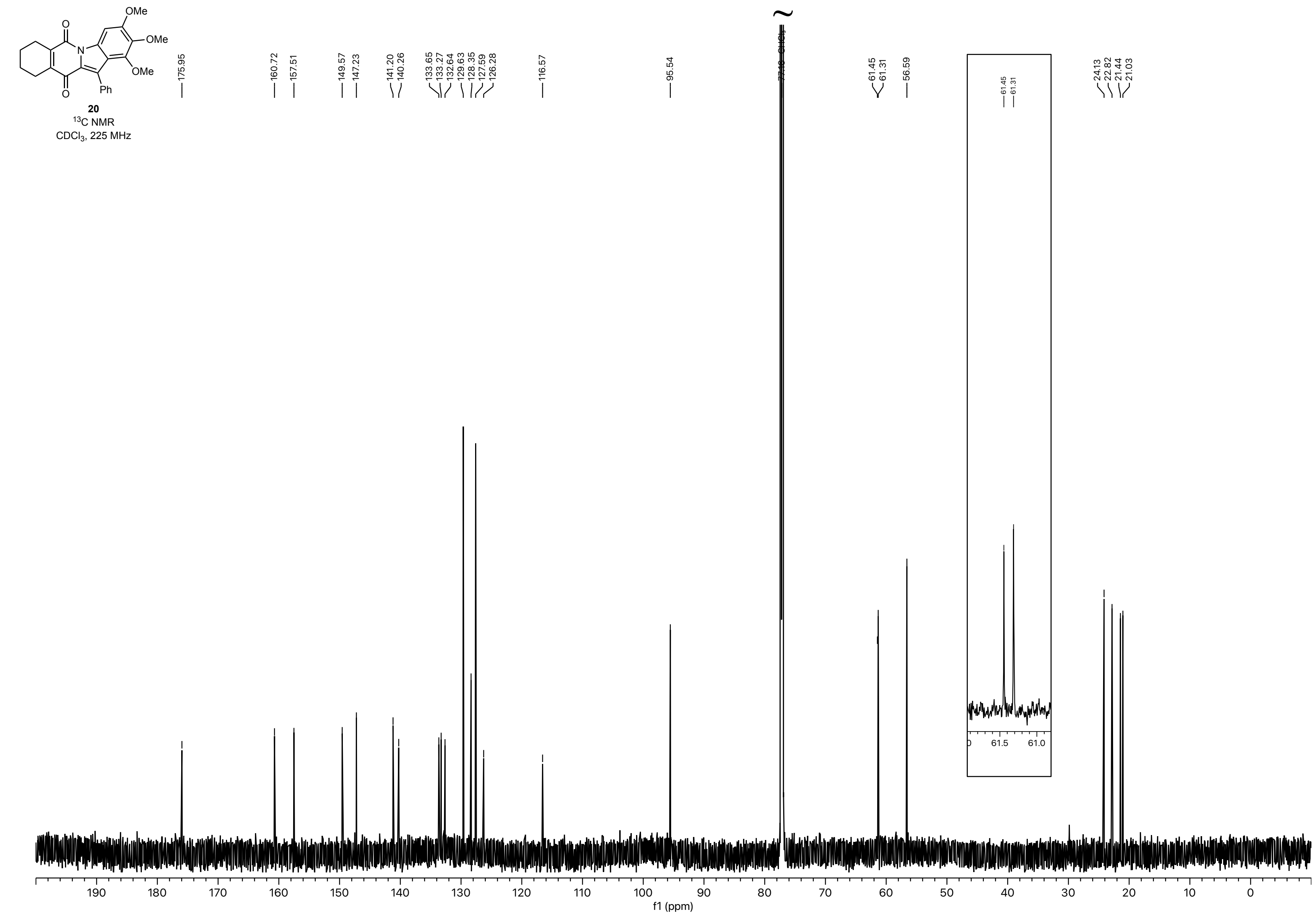

20
^{13}C NMR
$CDCl_3$, 225 MHz

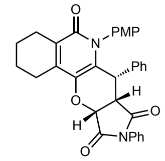

21a
^{1}H NMR
$CDCl_3$, 600 MHz

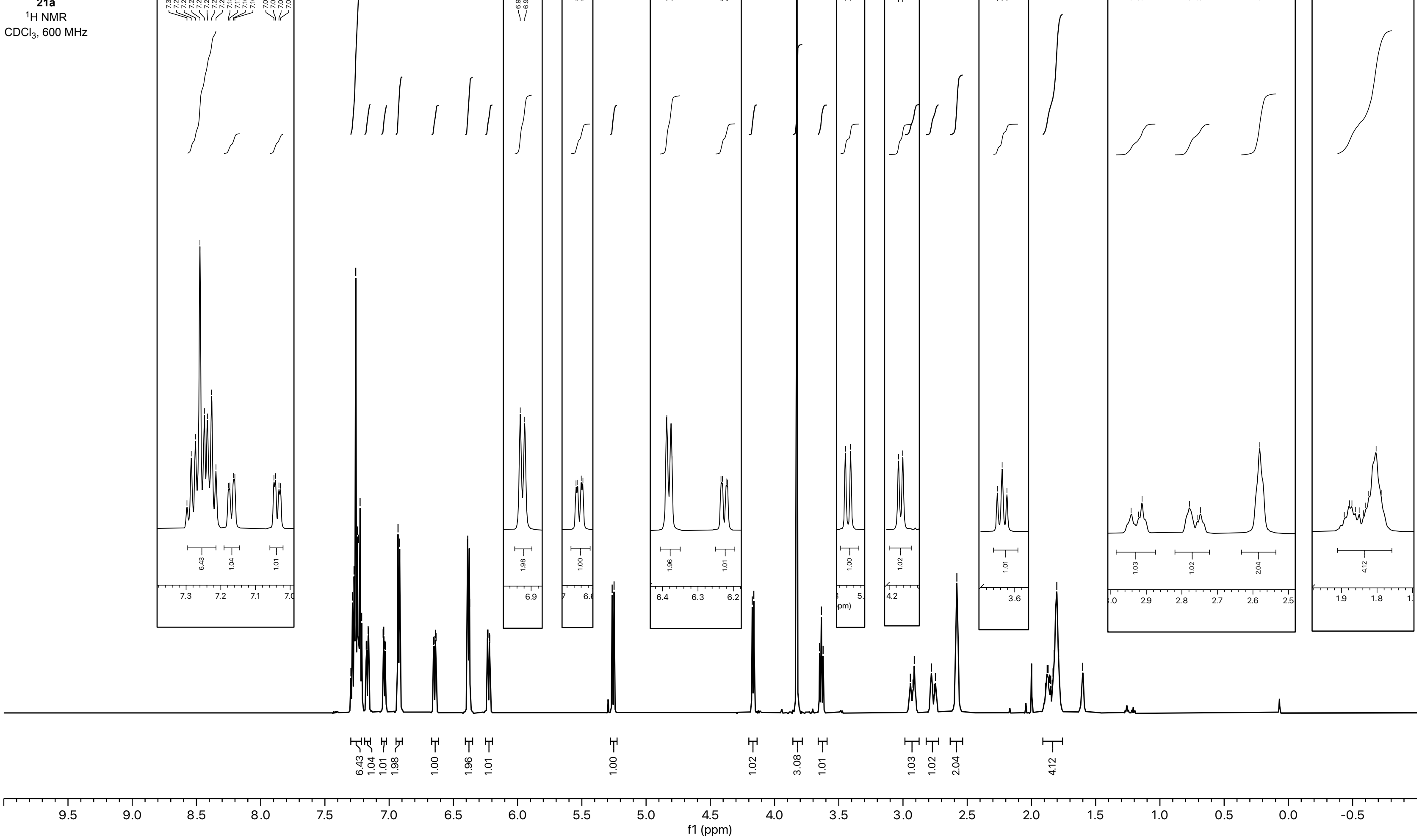

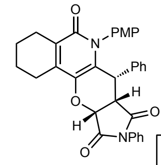

21a
^{13}C NMR
$CDCl_3$, 150 MHz

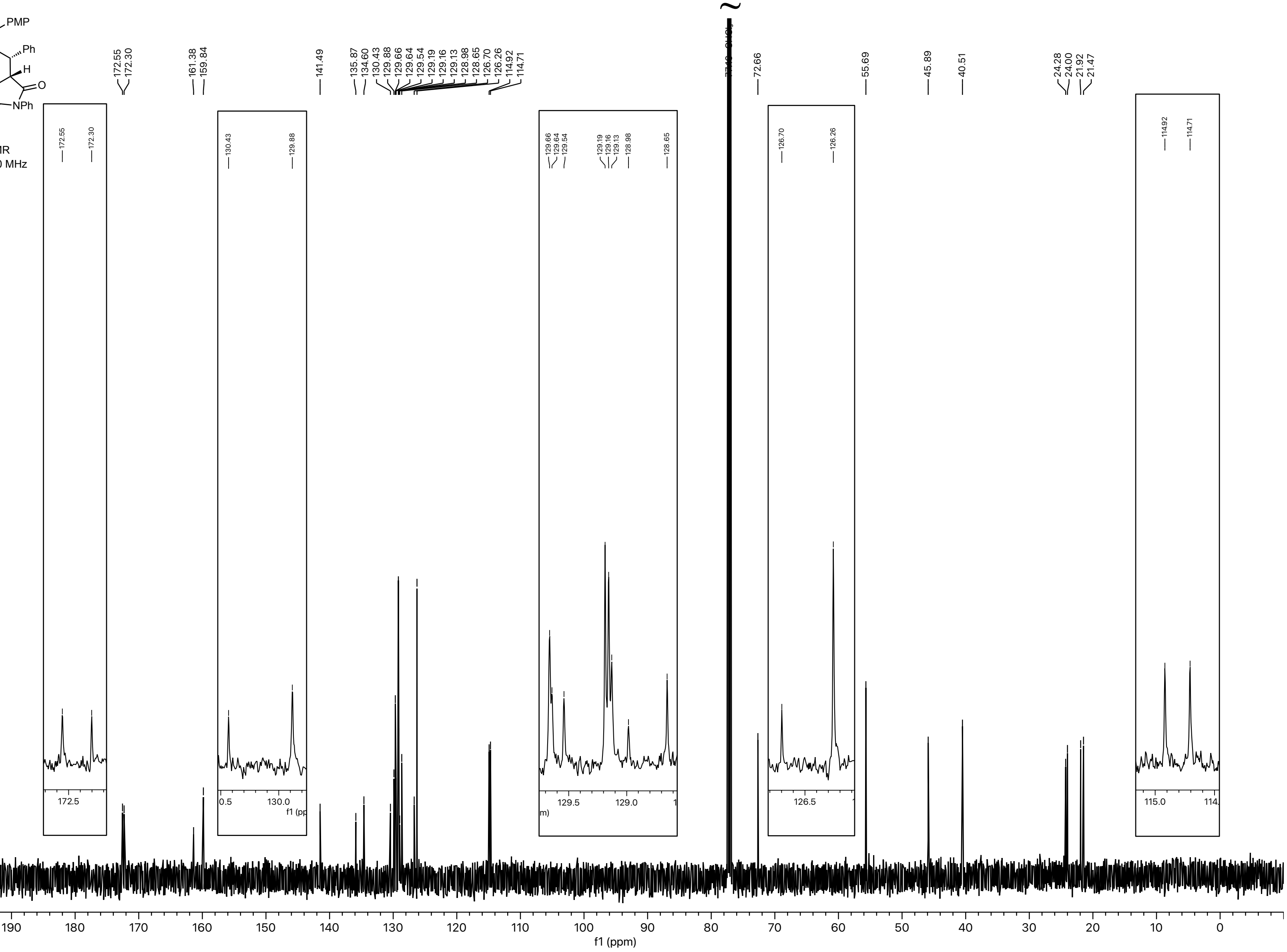

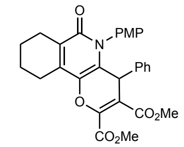

21b
^1H NMR
$CDCl_3$, 400 MHz

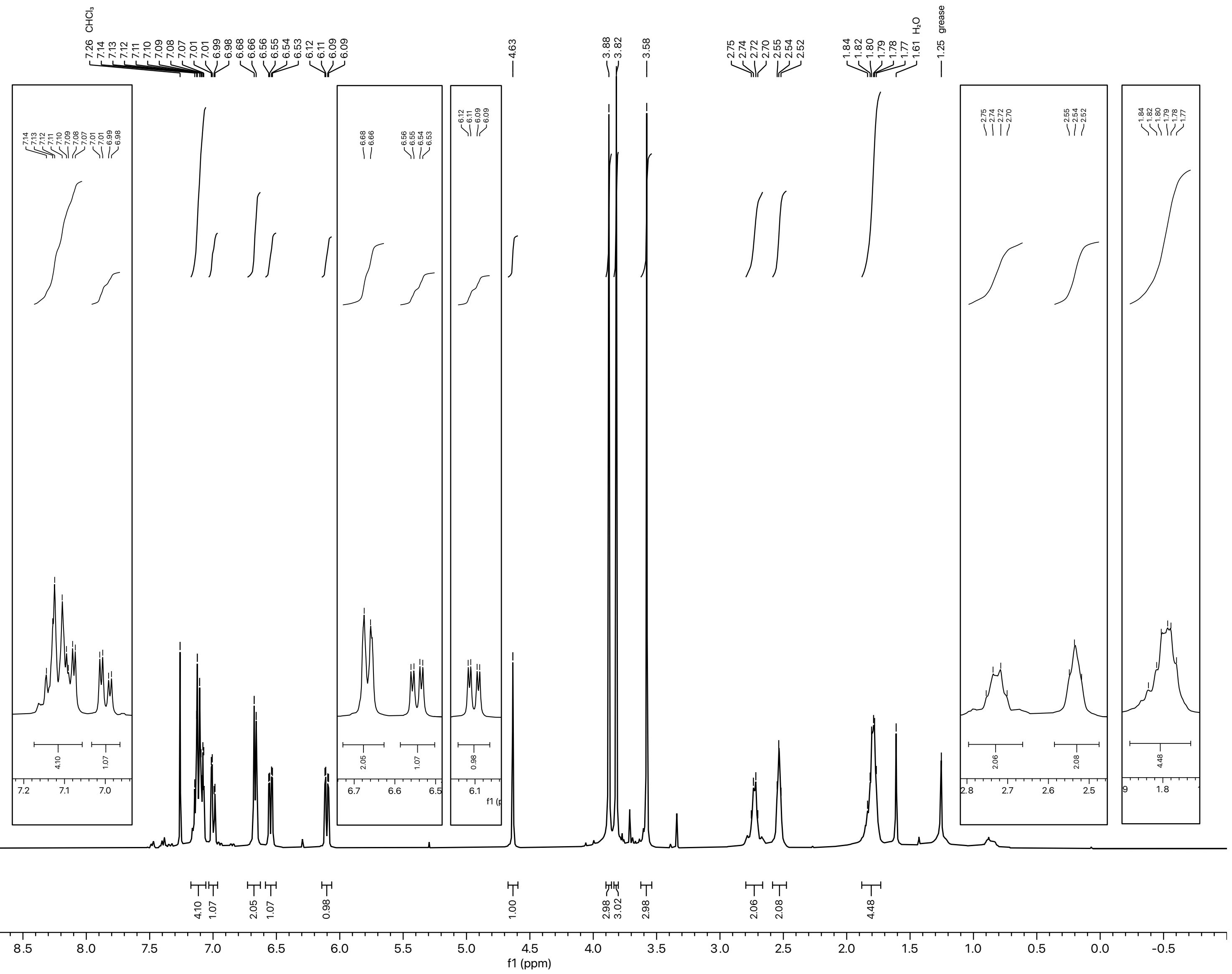

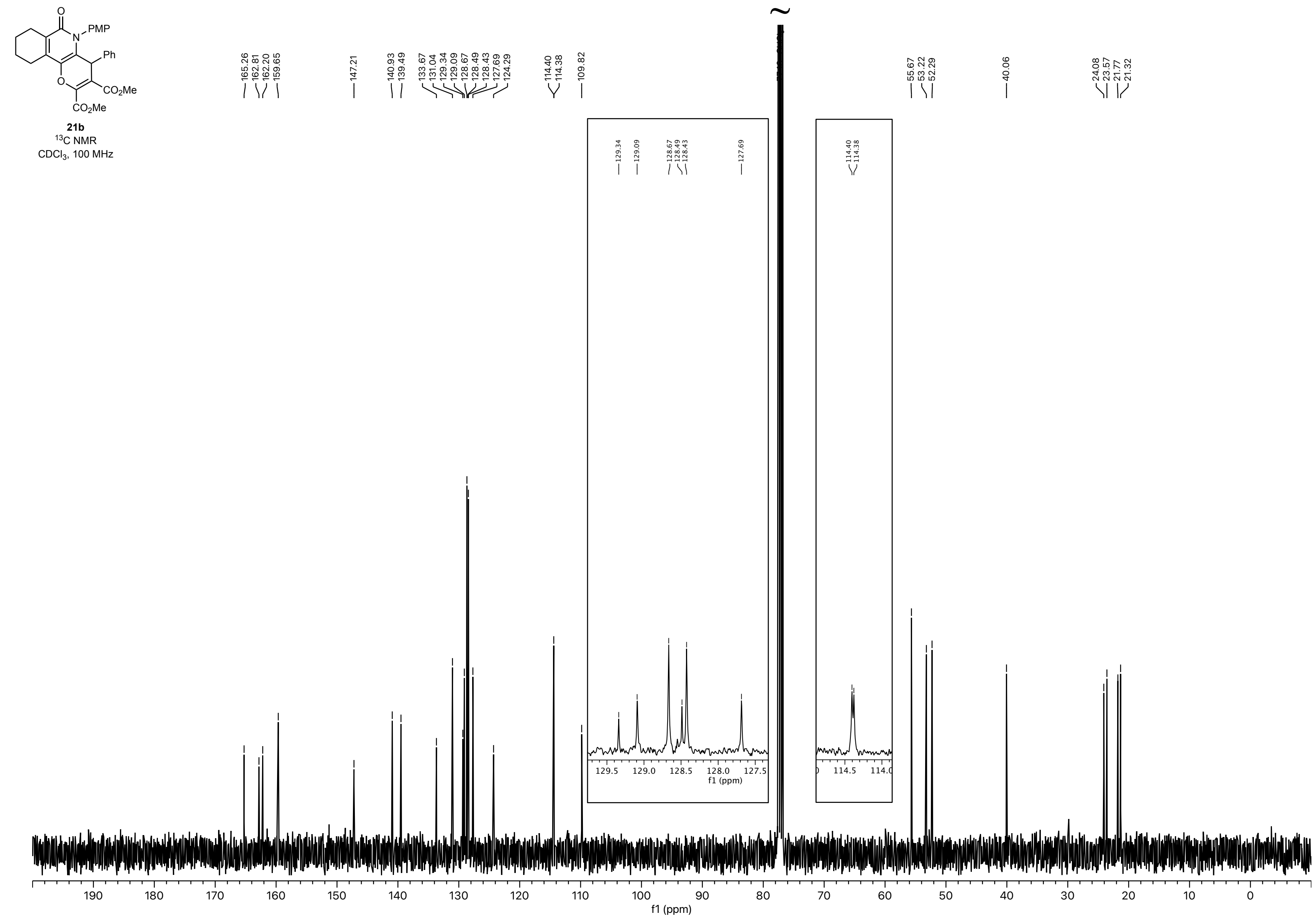

21b

^{13}C NMR

$CDCl_3$, 100 MHz

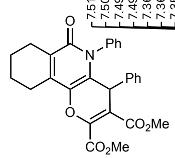

21c
^{1}H NMR
$CDCl_3$, 600 MHz

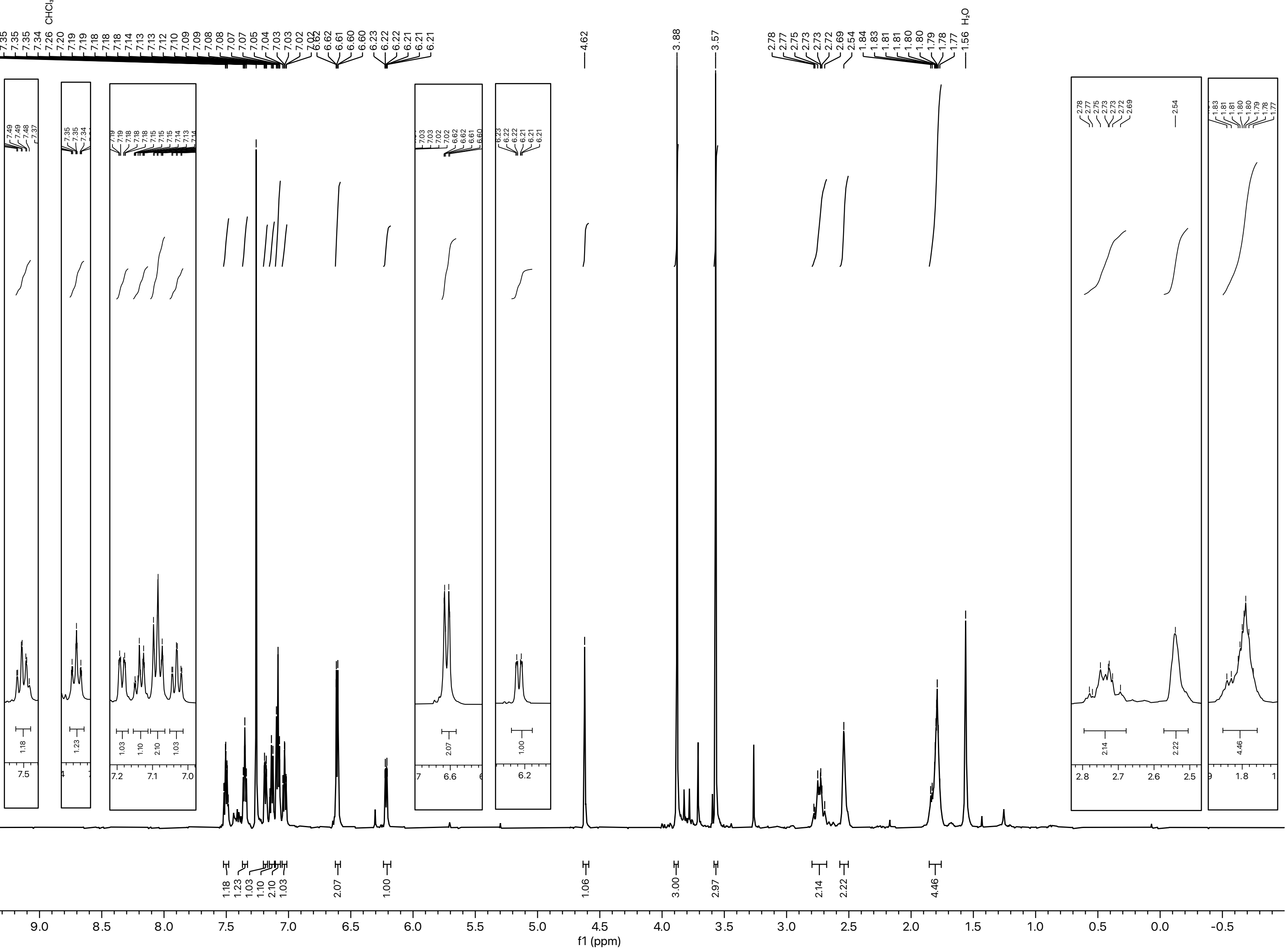

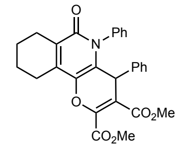

21c

^{13}C NMR

$CDCl_3$, 150 MHz

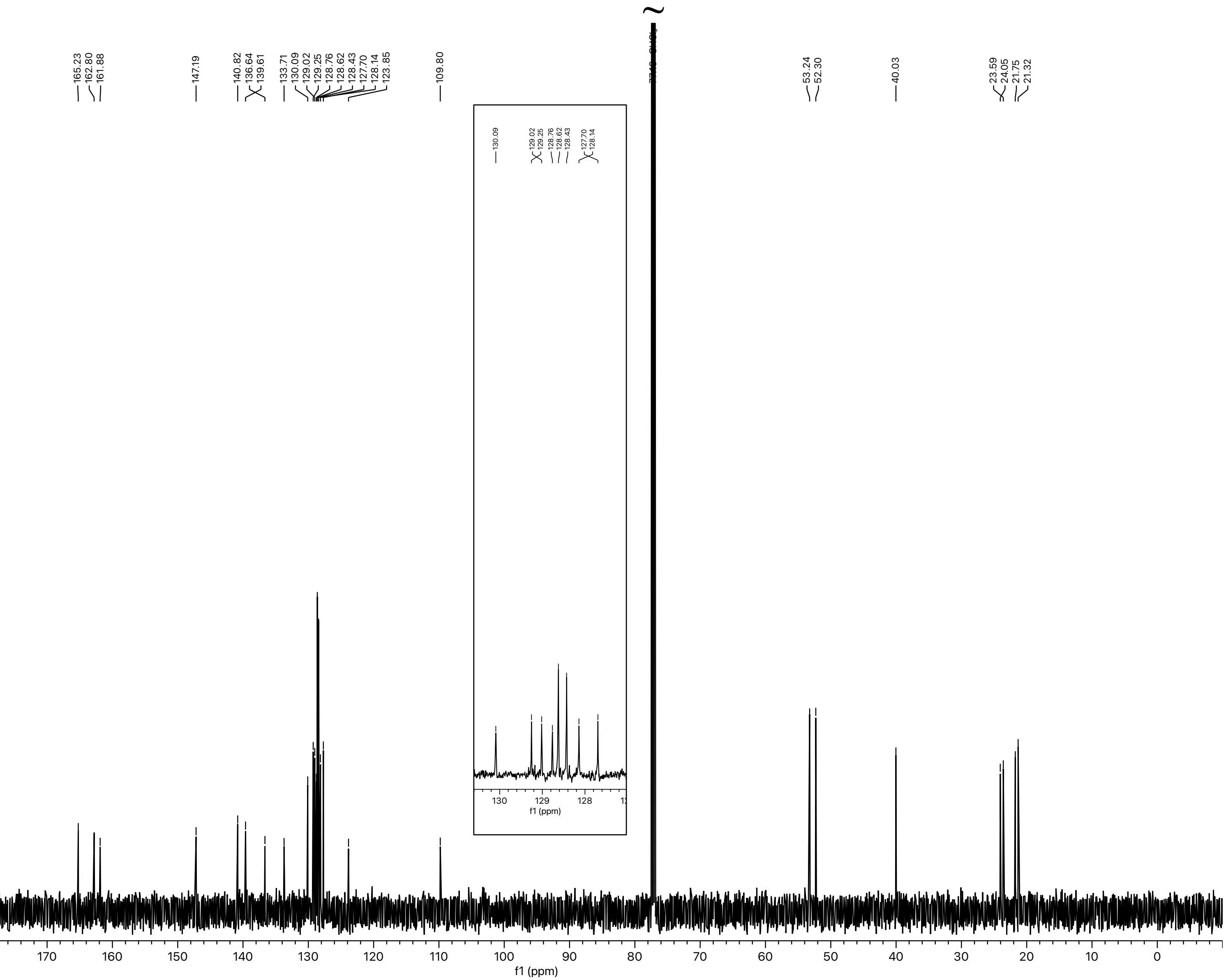

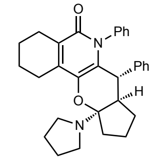

21d [major]
[isolated as an equilibrating 6:1 mixture of ring-closed hemiaminal ether (as drawn) and a ring-opened hemiaminal ether derivative]

only major isomer indicated

^{1}H NMR
$CDCl_3$, 400 MHz

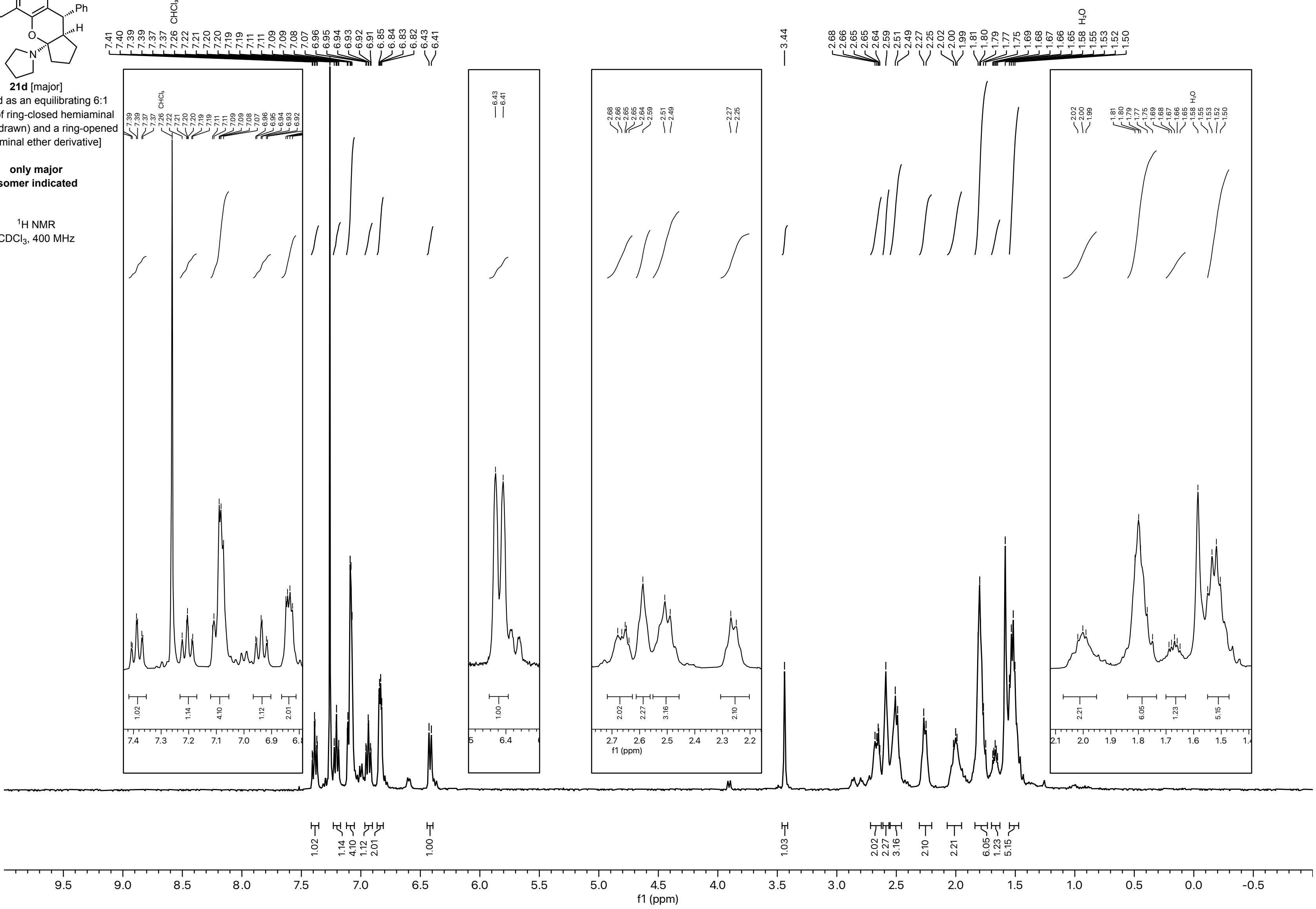

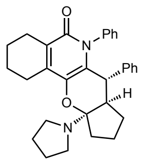

21d [major]
[isolated as an equilibrating 6:1 mixture of ring-closed hemiaminal ether (as drawn) and a ring-opened hemiaminal ether derivative]

only major isomer indicated

^{13}C NMR
$CDCl_3$, 150 MHz

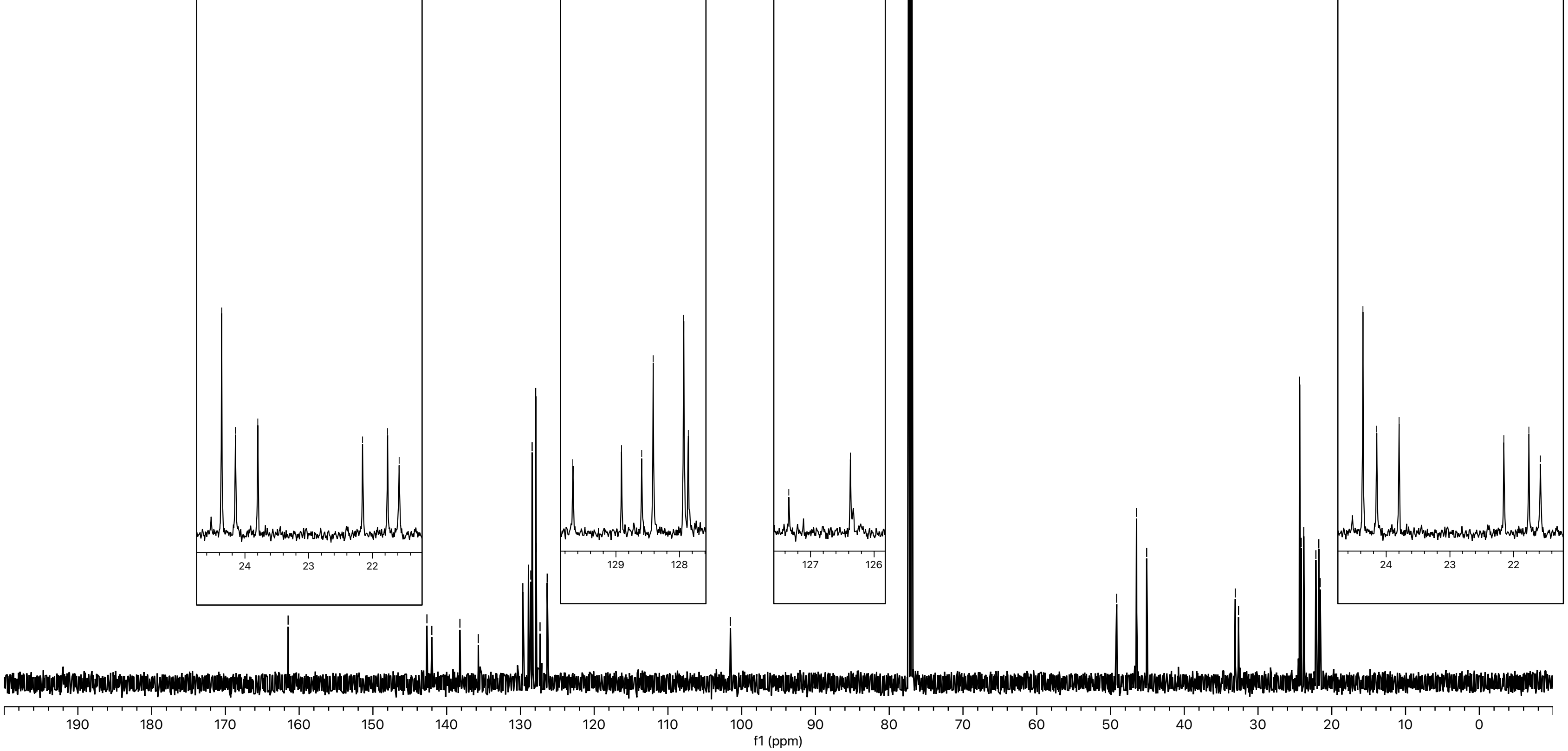

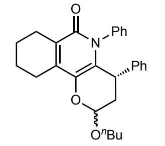

21e, dr = 2:1

both diastereoisomers indicated

^1H NMR
$CDCl_3$, 400 MHz

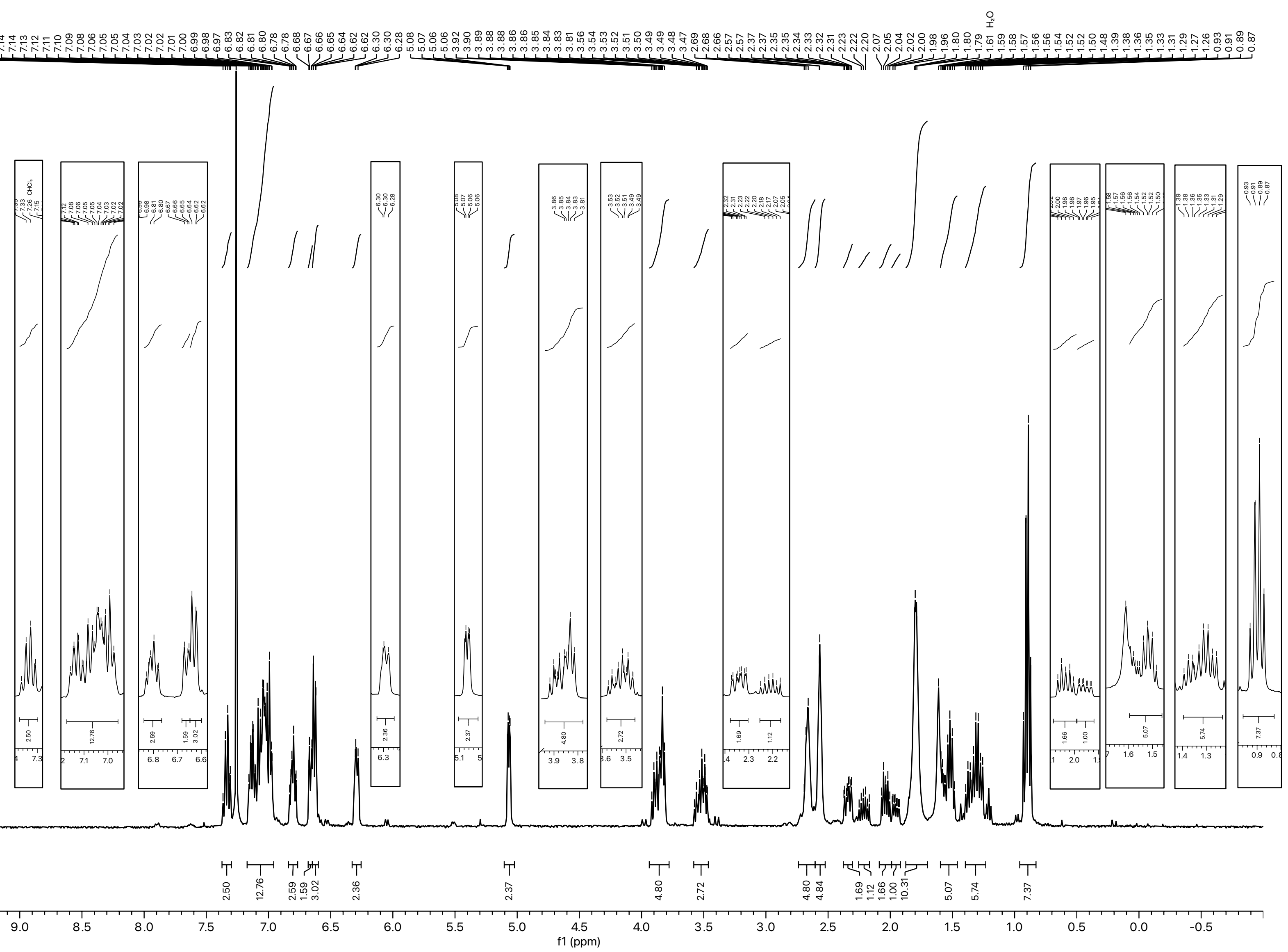

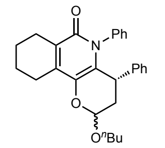

21e, dr = 2:1

both diastereoisomers indicated

^{13}C NMR
$CDCl_3$, 150 MHz

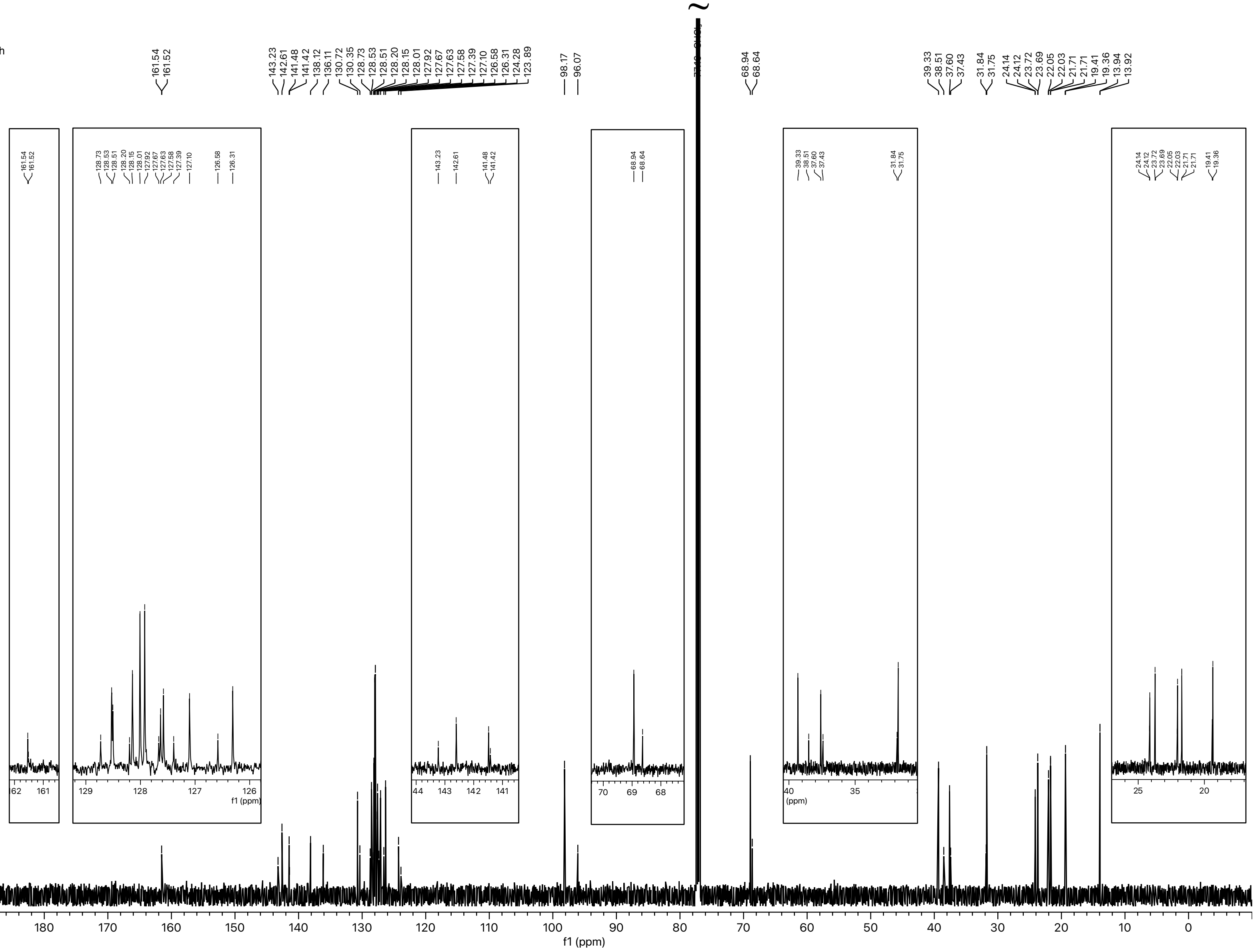

Comparing Ketenimines Against Imino Dienophiles

Dienophile Synthesis

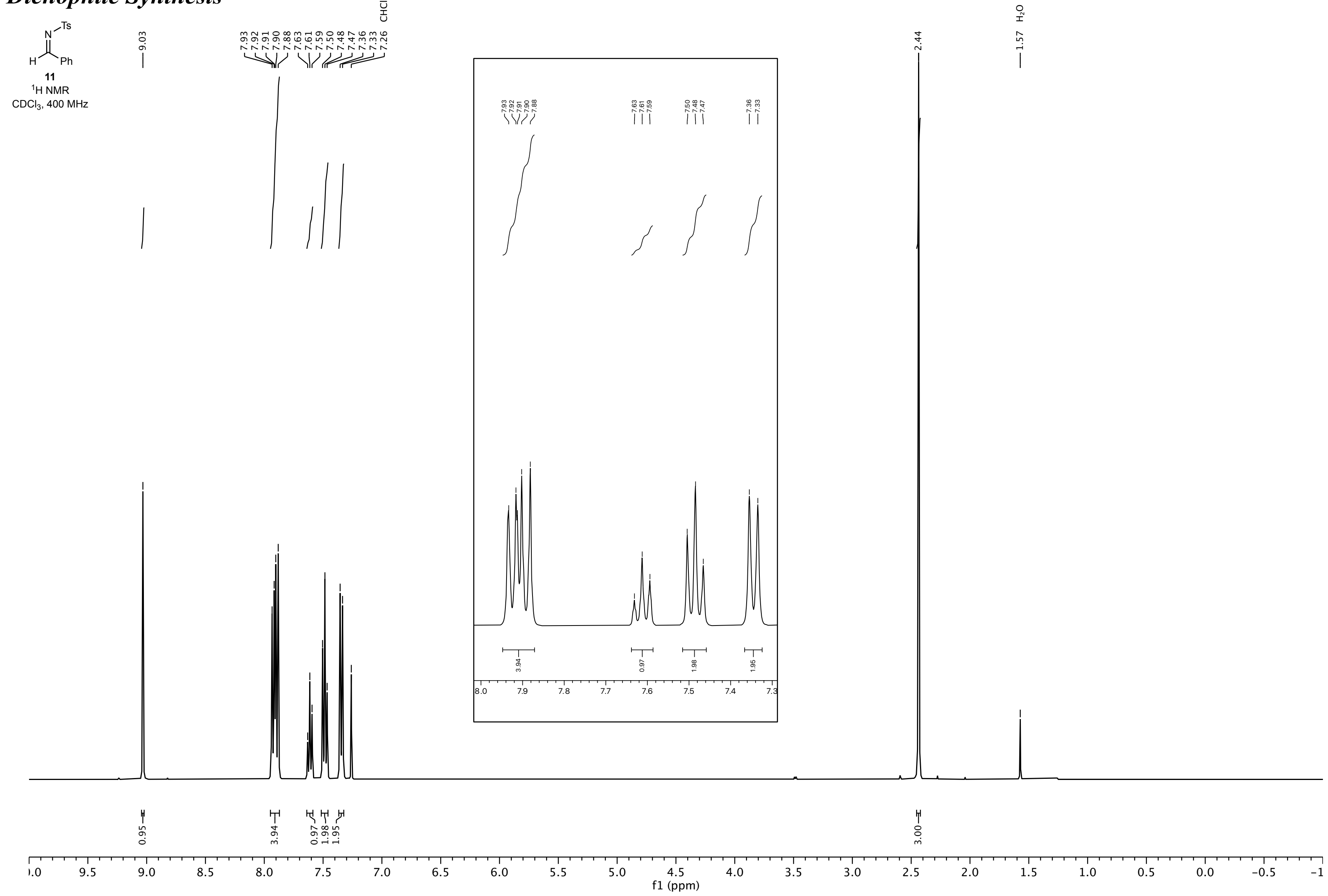

Diels–Alder Reactions

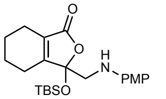

6

^{1}H NMR

$CDCl_3$, 400 MHz

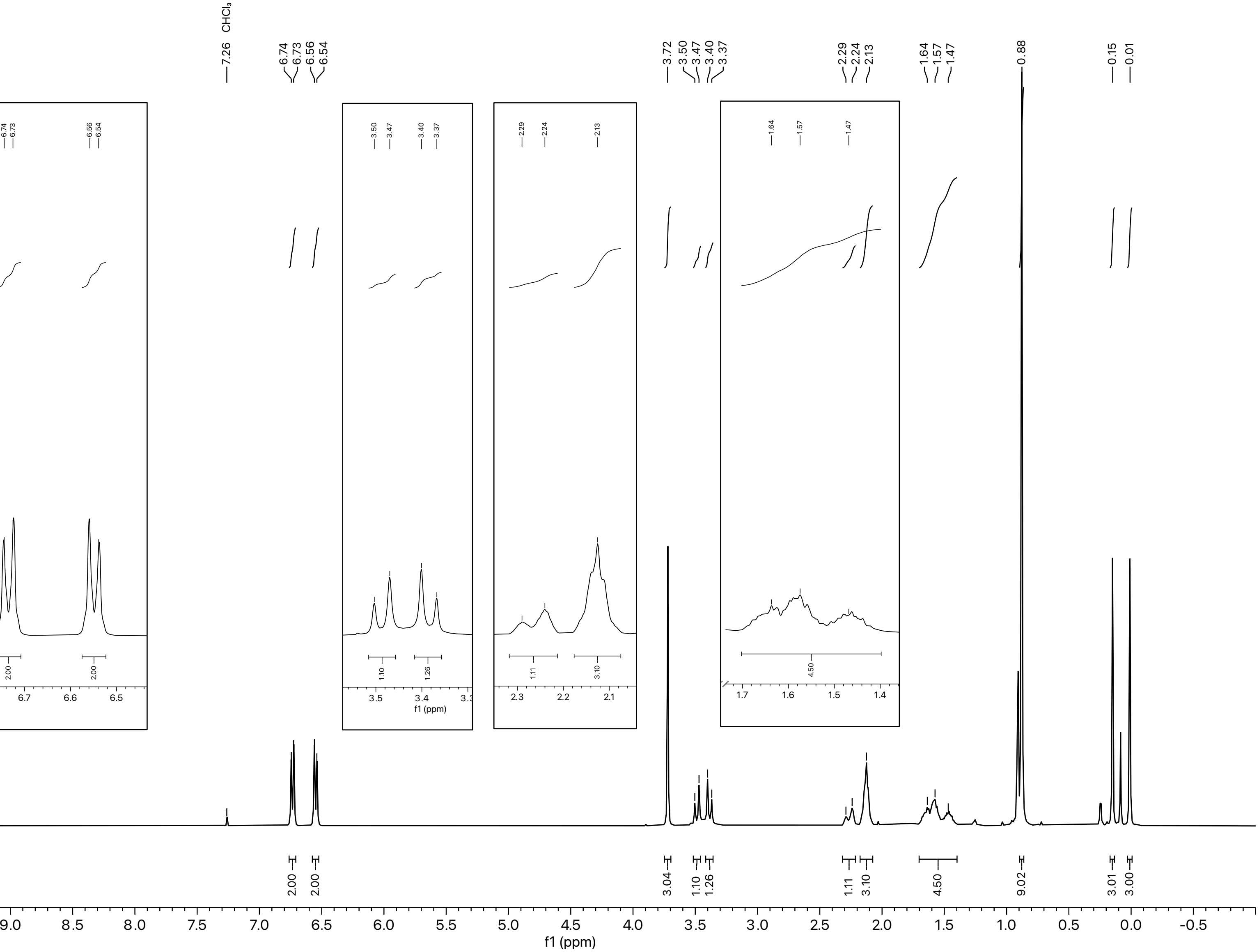

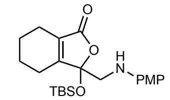

6
^{13}C NMR
$CDCl_3$, 100 MHz

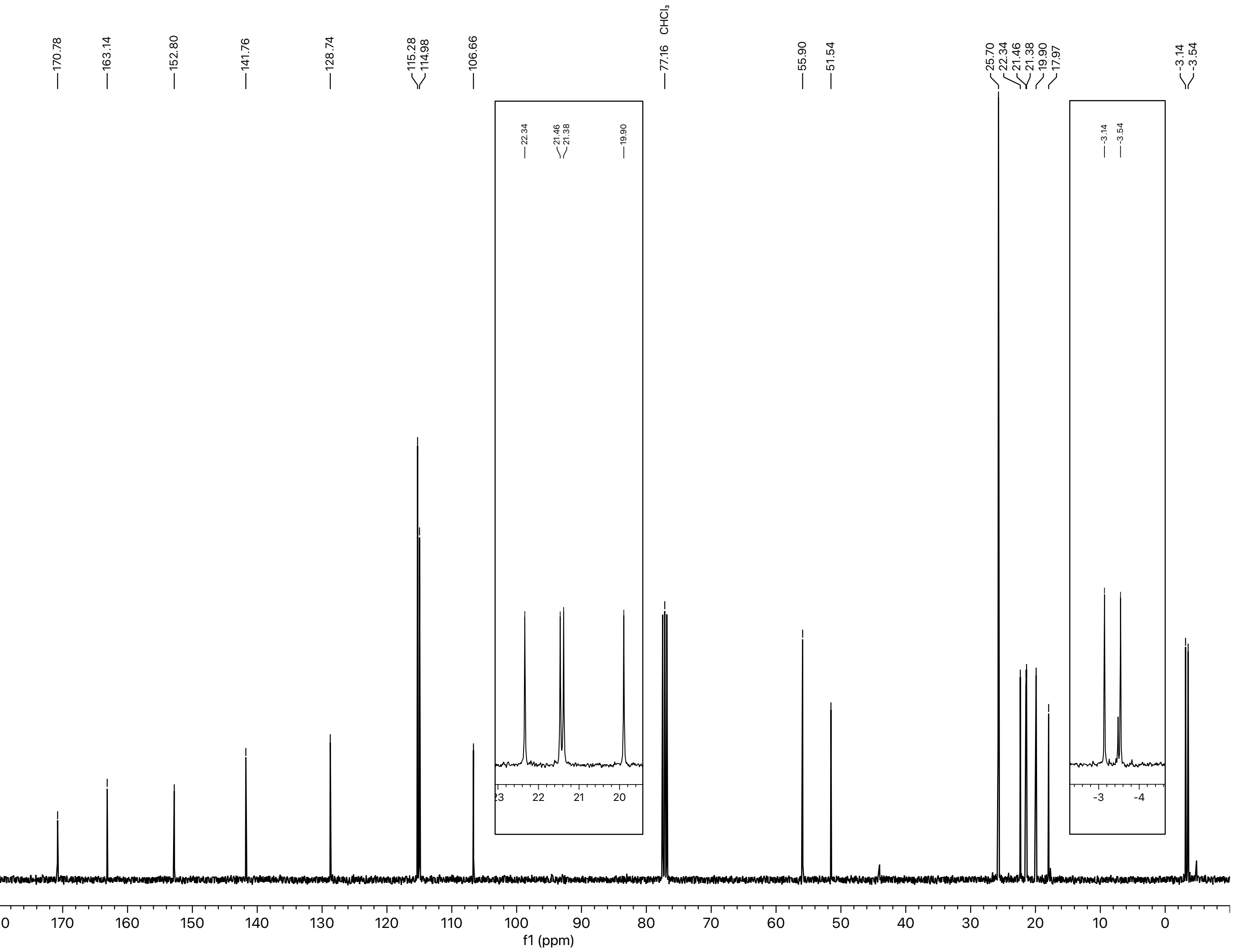

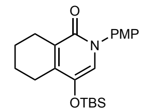

7

^1H NMR

$CDCl_3$, 600 MHz

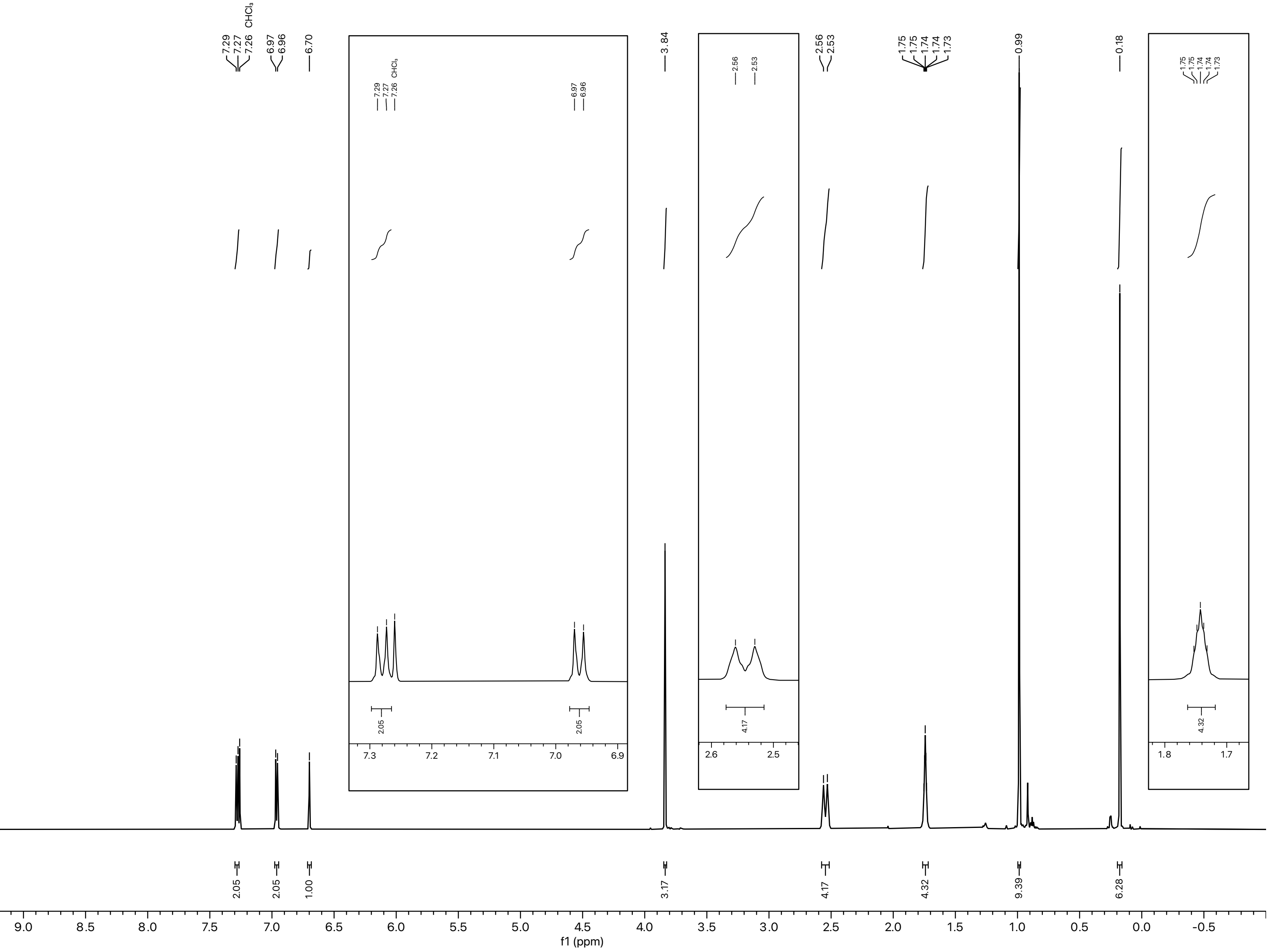

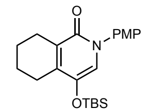

7
^{13}C NMR
$CDCl_3$, 150 MHz

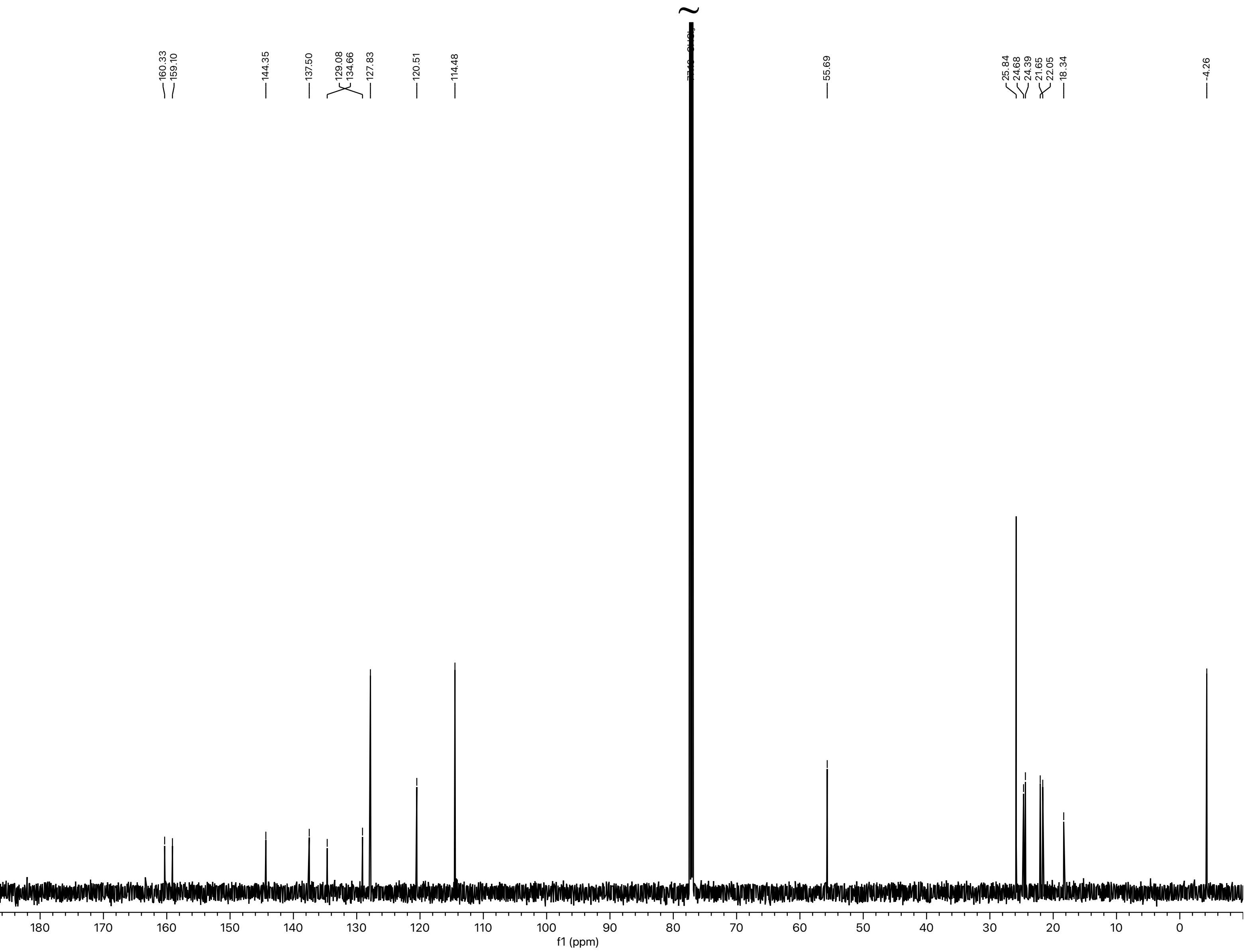

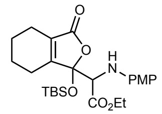

9 [dr = 1.3:1]

both diastereoisomers indicated

^{1}H NMR
$CDCl_3$, 400 MHz

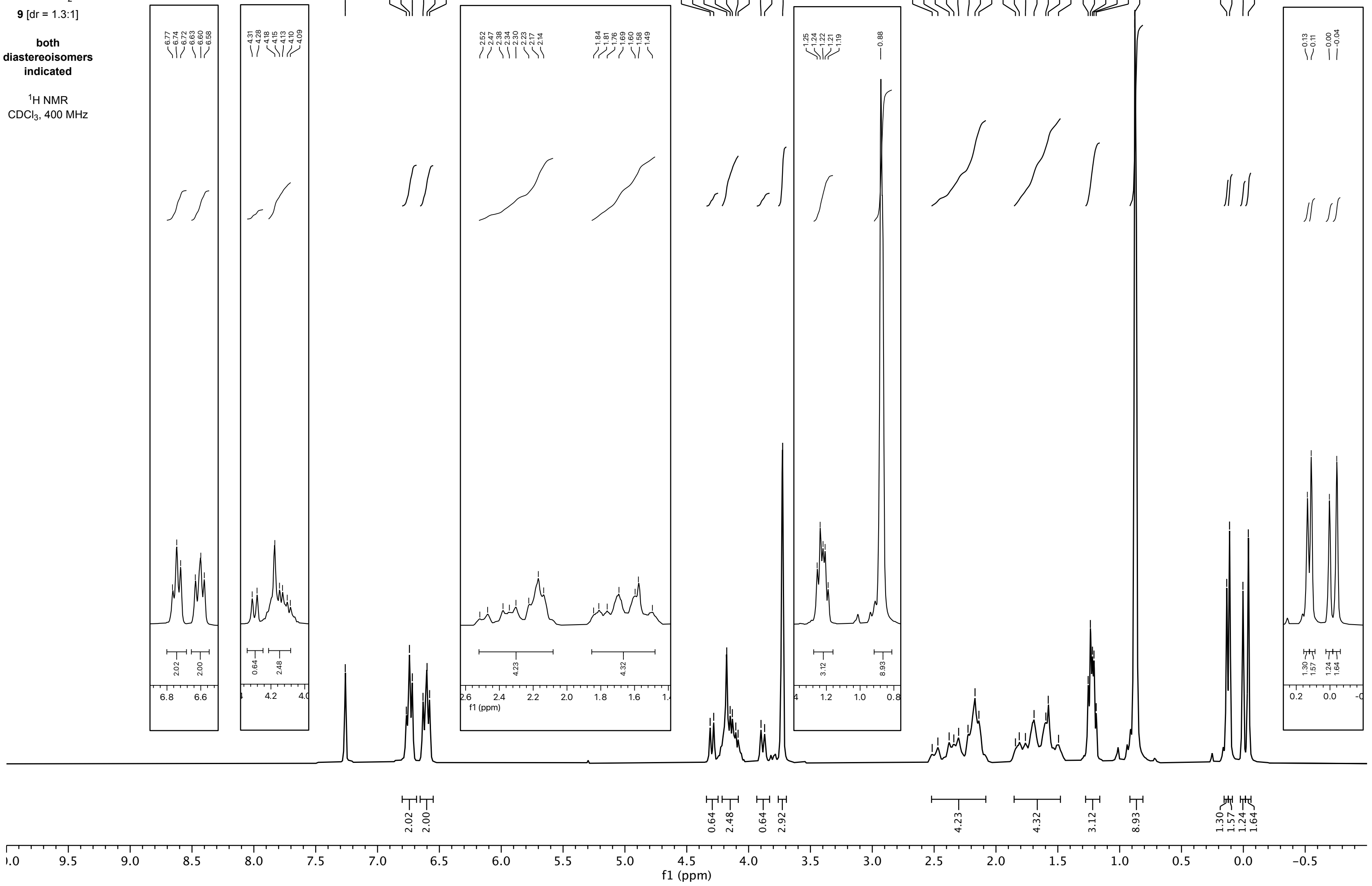

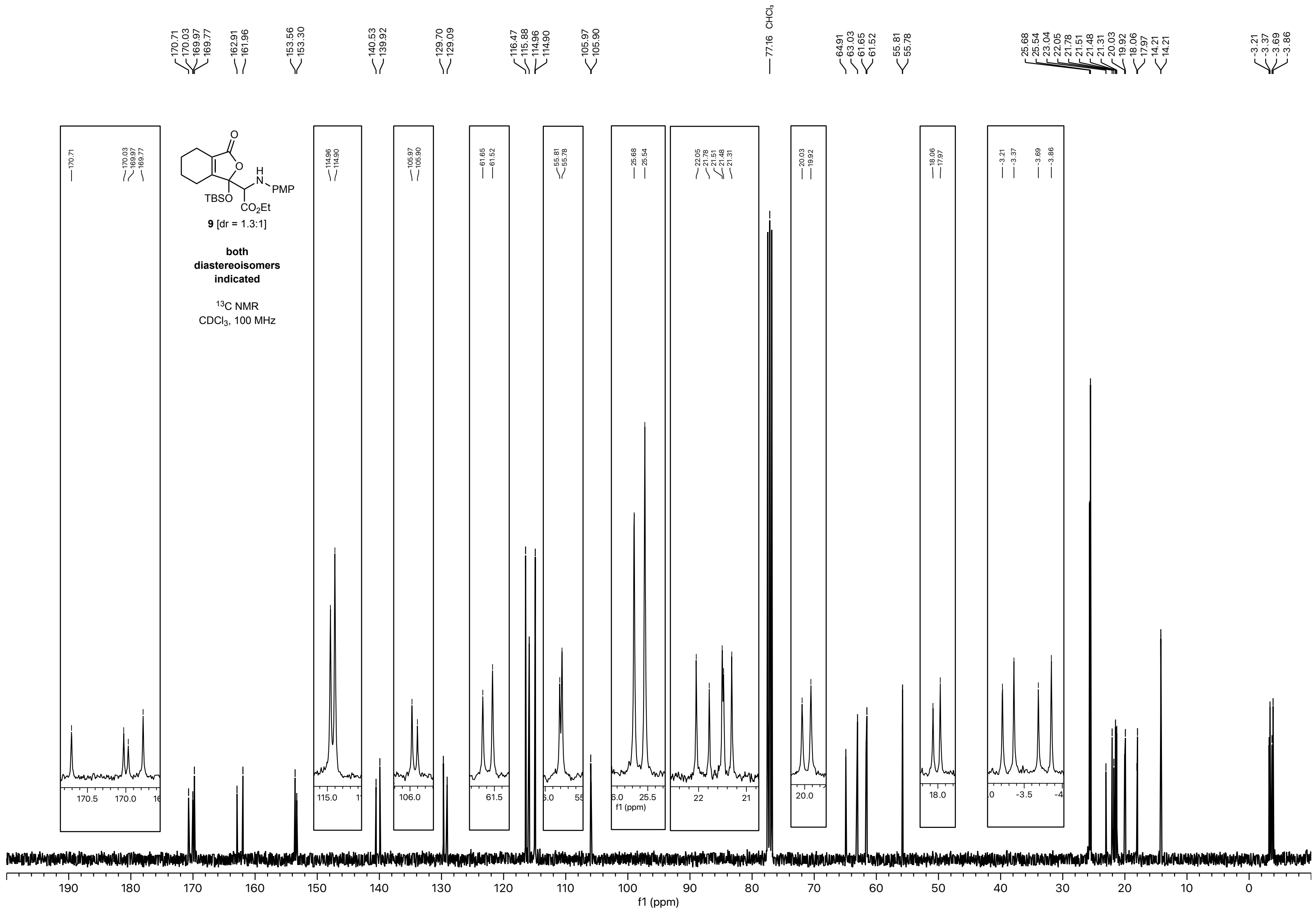

170.71
170.03
169.97
169.77
162.91
161.96
153.56
153.30
140.53
139.92
129.70
129.09
116.47
115.88
114.96
114.90
105.97
105.90
77.16 CHCl3
64.91
63.03
61.65
61.52
55.81
55.78
25.68
25.54
23.04
22.05
21.78
21.51
21.48
21.31
20.03
19.92
18.06
17.97
14.21
14.21
-3.21
-3.37
-3.69
-3.86
O
TBSO
H
N
PMP
CO2Et
9 [dr = 1.3:1]
both
diastereoisomers
indicated
13C NMR
CDCl3, 100 MHz
f1 (ppm)

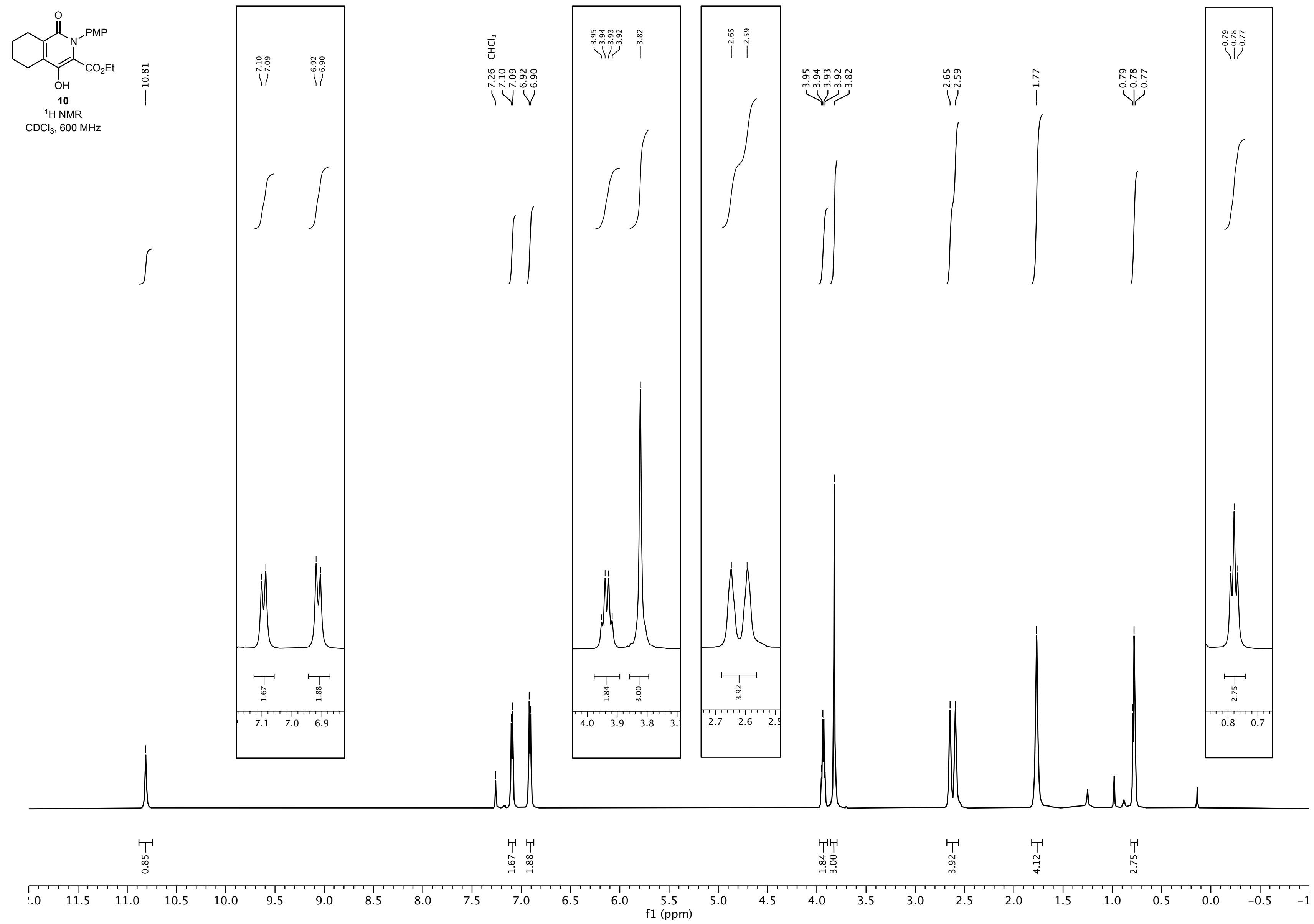
O
N
PMP
CO2Et
OH
10
1H NMR
CDCl3, 600 MHz
10.81
7.26 CHCl3
7.10
7.09
6.92
6.90
3.95
3.94
3.93
3.92
3.82
2.65
2.59
1.77
0.79
0.78
0.77
0.85
1.67
1.88
1.84
3.00
3.92
4.12
2.75
f1 (ppm)

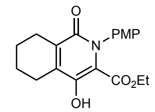

10

^{13}C NMR

$CDCl_3$, 100 MHz

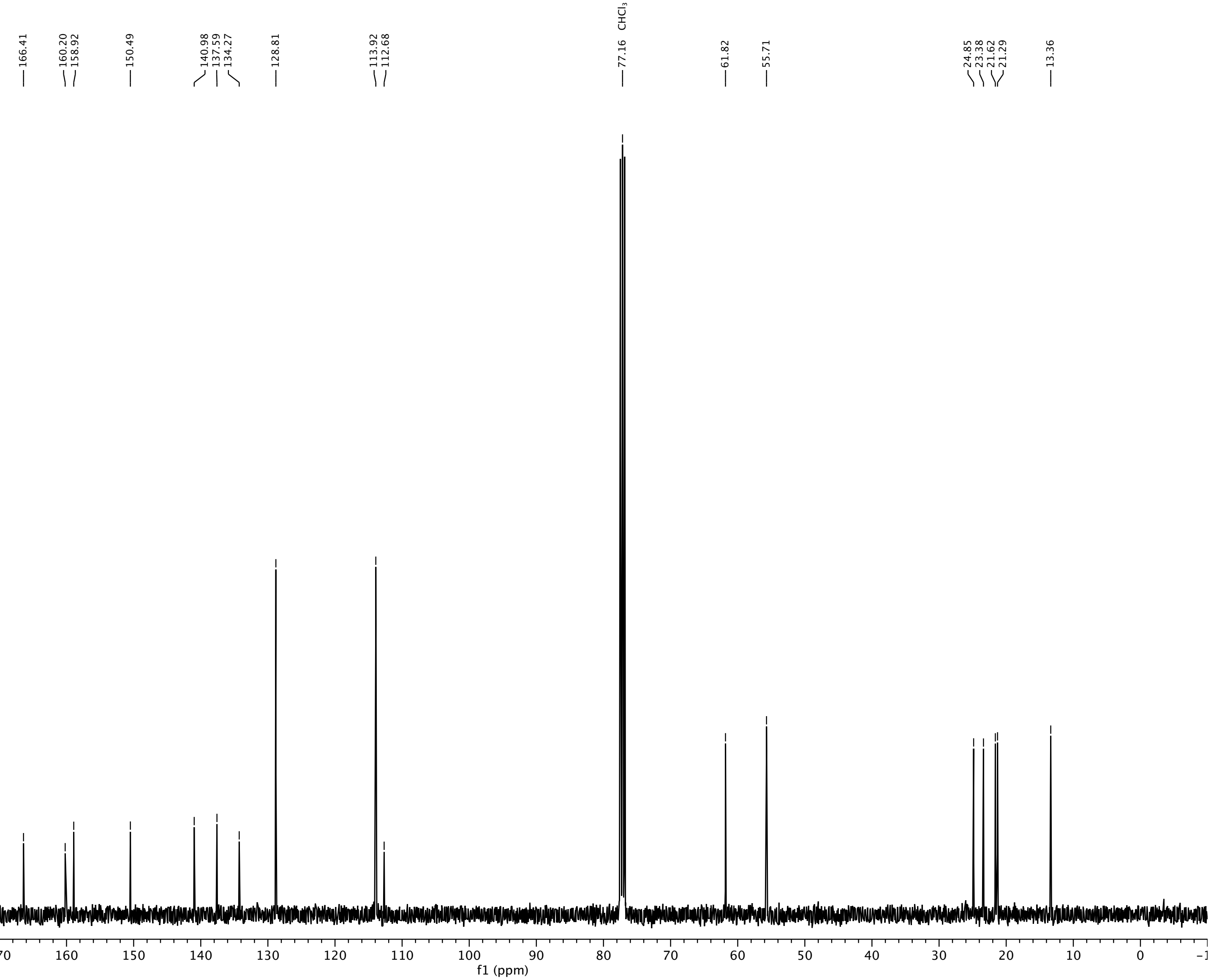

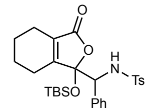

12 [dr > 20:1]
^{1}H NMR
$CDCl_3$, 400 MHz

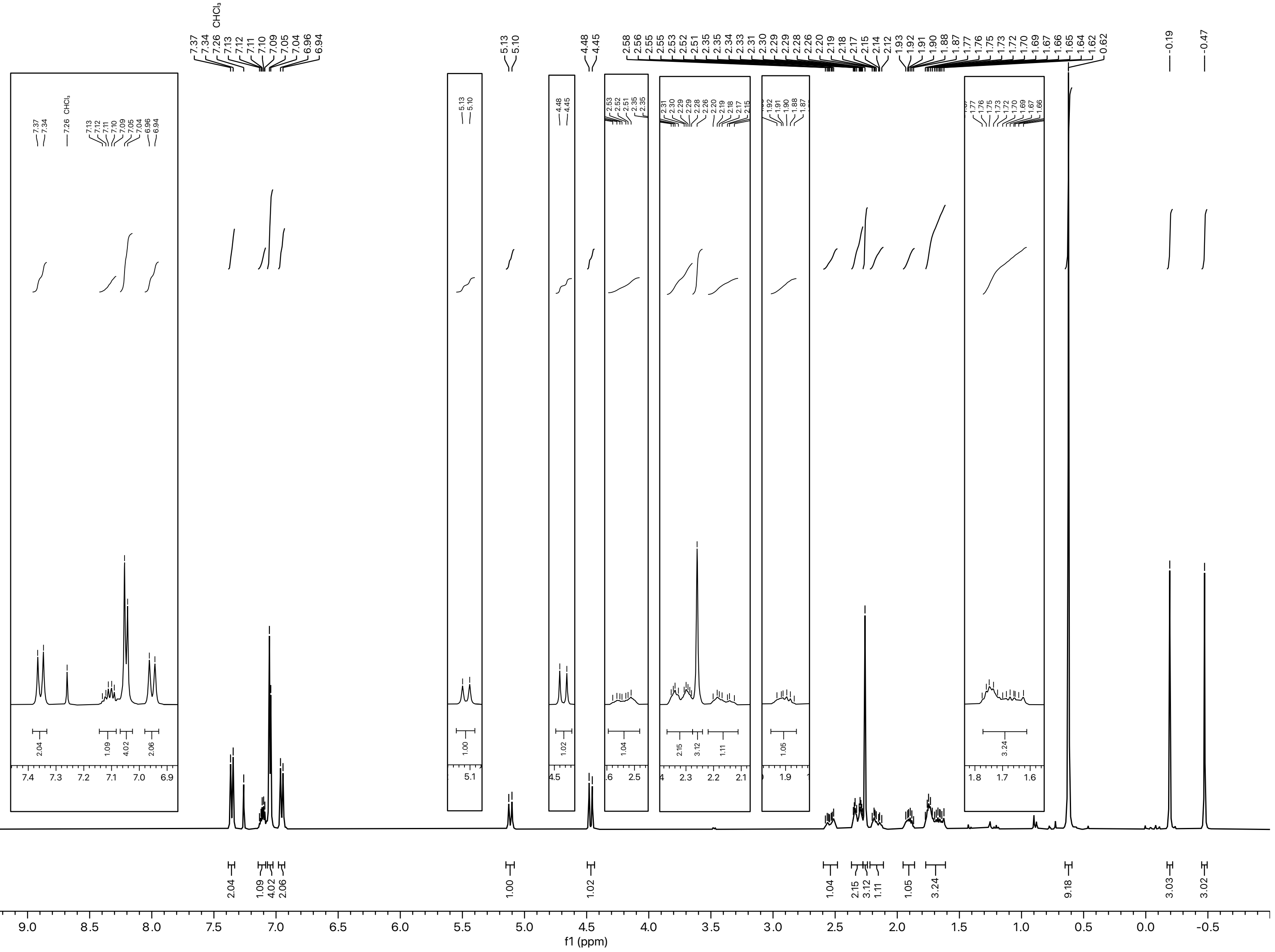

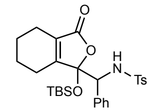

12 [dr > 20:1]
^{13}C NMR
$CDCl_3$, 100 MHz

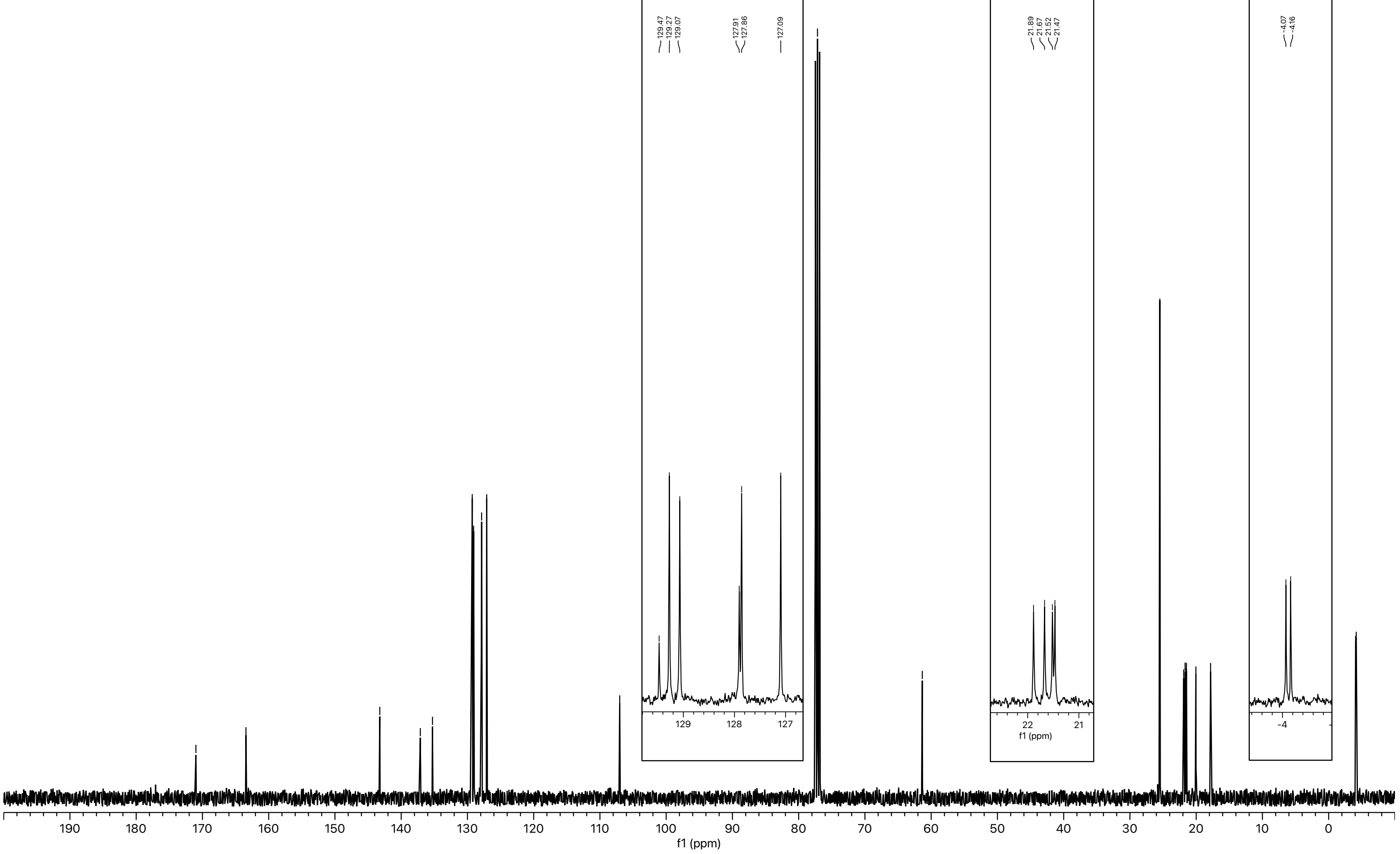

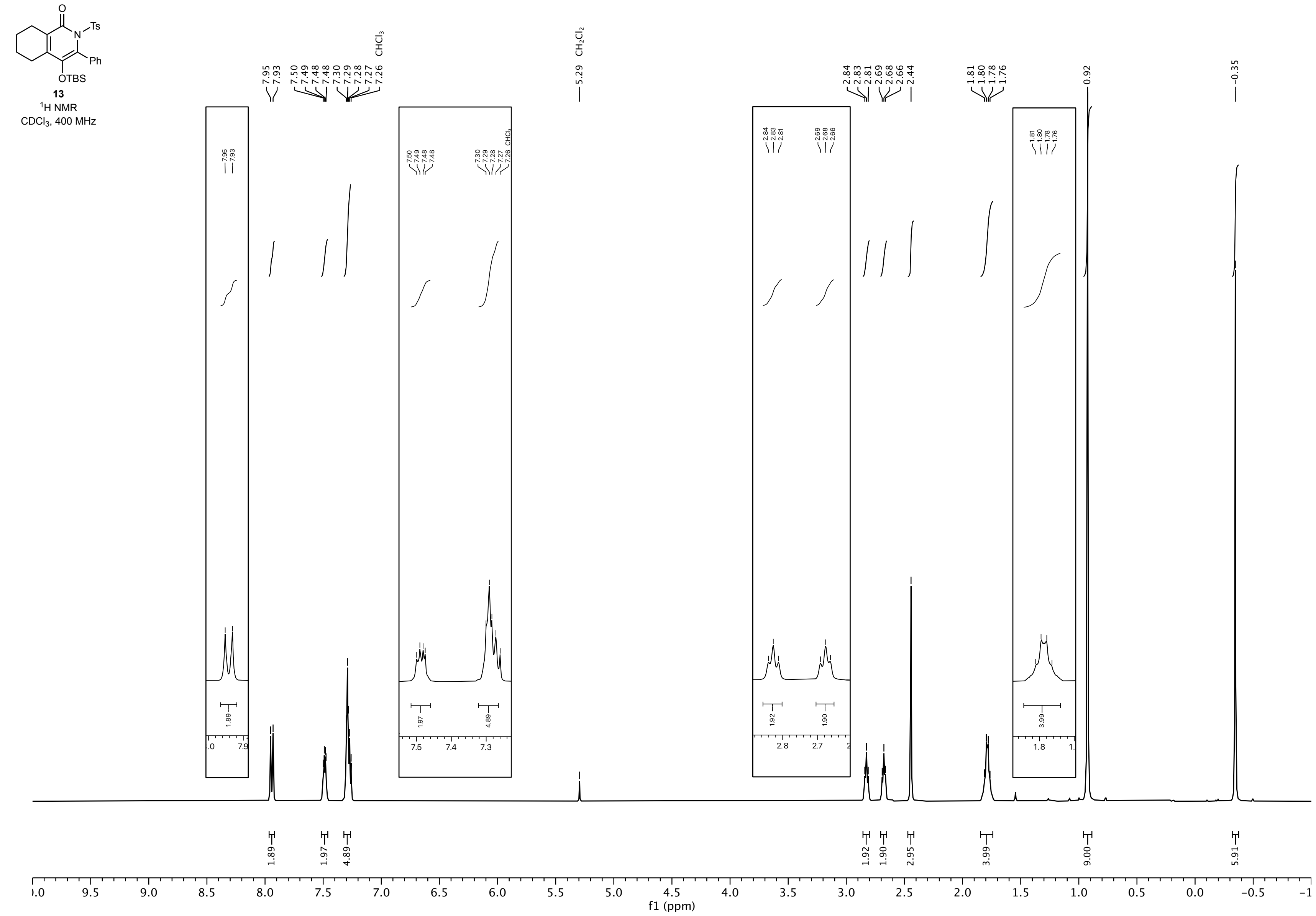

OTBS
Ts
Ph
13
^{1}H NMR
$CDCl_3$, 400 MHz
7.95
7.93
7.50
7.49
7.48
7.48
7.30
7.29
7.28
7.27
7.26
$CHCl_3$
5.29 CH_2Cl_2
2.84
2.83
2.81
2.69
2.68
2.66
2.44
1.81
1.80
1.78
1.76
0.92
-0.35
1.89
1.97
4.89
1.92
1.90
2.95
3.99
9.00
5.91
f1 (ppm)

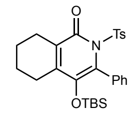

13

^{13}C NMR

$CDCl_3$, 100 MHz

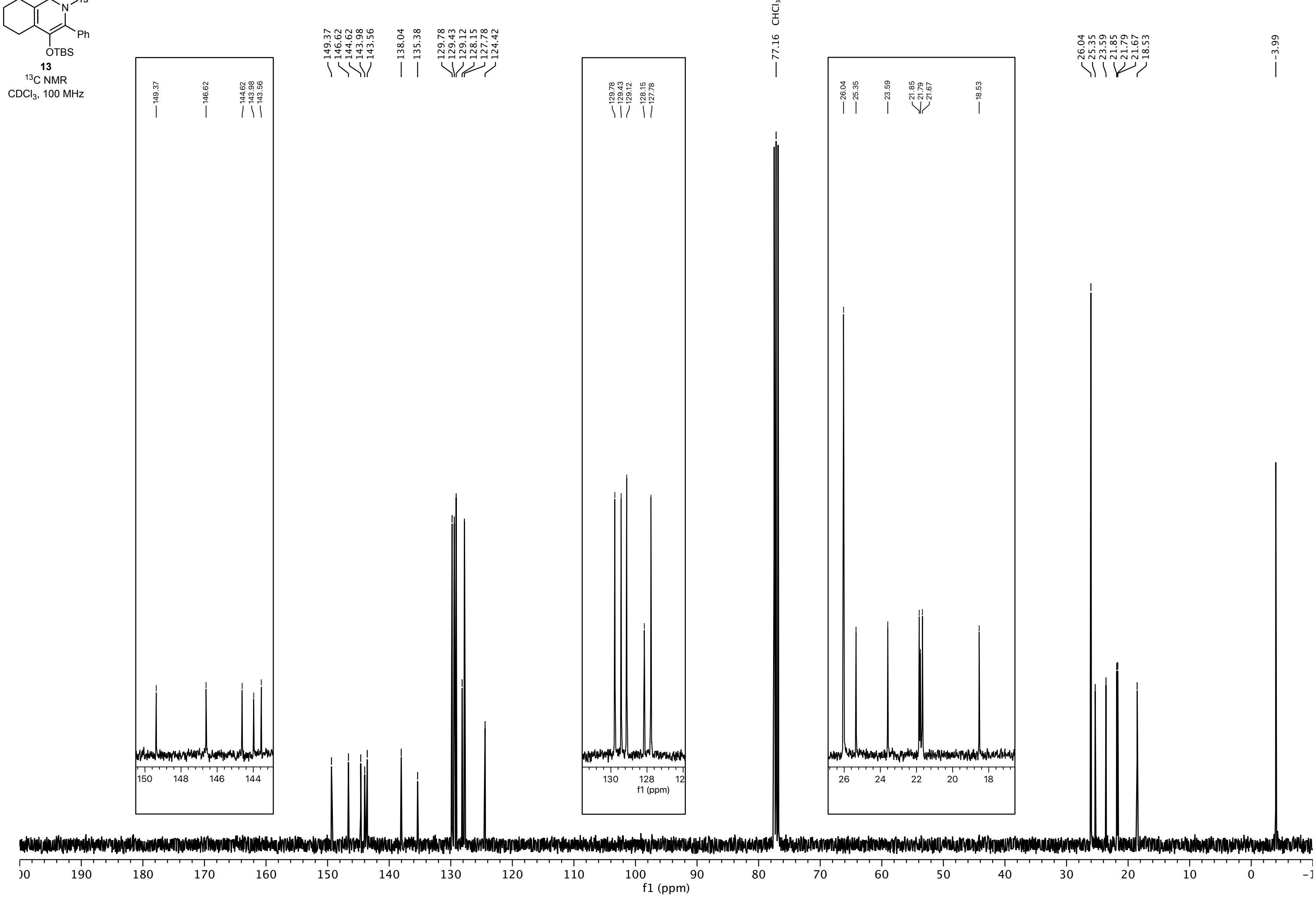

References

(1) Tagmazyan, K. T.; Mkrtchyan, R. S.; Babayan, A. T. Investigations in the Field of Amines and Ammonium Compounds. XCIX. Synthesis of Di- and Tetrahydro-5,7-*α*-Epoxyisoindolinium Salts. *Russ. J. Org. Chem.* **1974**, 1657–1662.

(2) Whitney, S. E.; Winters, M.; Rickborn, B. Benzyne-Oxazole Cycloadducts: Isolation and Retro-Diels–Alder Reactions. *J. Org. Chem.* **1990**, *55*, 929–935. DOI: 10.1021/jo00290a025.

(3) Rickborn, B. The Retro-Diels–Alder Reaction Part II. Dienophiles with One or More Heteroatom. In *Organic Reactions*, Paquette, L. A. Ed.; Vol. 53; John Wiley & Sons, Inc., New York, 1998; pp 223–629. DOI: 10.1002/0471264180.or053.02.

(4) vor der Brück, D.; Bühler, R.; Plieninger, H. Azomethin-Tricarbonsäureester als Philodien Unter Hochdruck—IV: Hochdruckversuche. *Tetrahedron* **1972**, *28*, 791–795. DOI: 10.1016/0040-4020(72)84040-7.

(5) (a) Al'bekov, V. A.; Benda, A. F.; Gontar, A. F.; Sokol'skii, G. A.; Knunyants, I. L. Реакции [2+4] Циклоприсоединения Фторолефинов И Фторазометинов С Фураном *Izv. Akad. Nauk, Ser. Khim.* **1988**, 897–900. (b) Al'bekov, V. A.; Benda, A. F.; Gontar, A. F.; Sokol'skii, G. A.; Knunyants, I. L. [2+4]-Cycloaddition of Fluoroolefins and Fluoroazomethines with Furan. *Russ. Chem. Bull.* **1988**, *37*, 777–780. DOI: 10.1007/bf01455502.

(6) (a) Krolevets, A. A.; Popov, A. G.; Adamov, A. V.; Martynov, I. V. O-силилированные Оксимы – Новые Диенофилы В Реакции Дильса – Альдера. *Izv. Akad. Nauk, Ser. Khim.* **1988**, 876–878. (b) Krolevets, A. A.; Adamov, A. V.; Popov, A. G.; Martynov, I. V. *O*-Silylated Oximes in the Diels–Alder Reaction. *Russ. Chem. Bull.* **1988**, *37*, 1737–1737. DOI: 10.1007/bf00961143.

(7) Gavina, F.; Costero, A. M.; Andreu, M. R.; Carda, M.; Luis, S. V. 2-Aza-2,4-Cyclopentadienone. Existence and Reactivity. *J. Am. Chem. Soc.* **1988**, *110*, 4017–4018. DOI: 10.1021/ja00220a047.

(8) Gavina, F.; Costero, A. M.; Andreu, M. R. Reaction of 4-Substituted 2-Azetidinone with Nucleophiles. Existence and Reactivity of 1-Azetin-4-one. *J. Org. Chem.* **1990**, *55*, 434–437. DOI: 10.1021/jo00289a011.

(9) Gavina, F.; Costero, A. M.; Andreu, M. R.; Ayet, M. D. Aza and Diazaannulenones. Influence of Nitrogen Position on their Reactivity and Stability. *J. Org. Chem.* **1991**, *56*, 5417–5421. DOI: 10.1021/jo00018a040.

(10) Alves, M. J.; Gilchrist, T. L. Methyl 2-Aryl-2*H*-Azirine-3-Carboxylates as Dienophiles. *J. Chem. Soc., Perkin Trans. 1* **1998**, 299–304. DOI: 10.1039/a705029a.

(11) Alves, M. J.; Azoia, N. G.; Bickley, J. F.; Fortes, A. G.; Gilchrist, T. L.; Mendonça, R. Diels–Alder Reactions of Alkyl 2*H*-Azirine-3-Carboxylates with Furans. *J. Chem. Soc., Perkin Trans. 1* **2001**, 2969–2976. DOI: 10.1039/b106985n.

(12) Erb, J.; Strull, J.; Miller, D.; He, J.; Lectka, T. The Diels–Alder Cyclization of Ketenimines. *Org. Lett.* **2012**, *14*, 2191–2193. DOI: 10.1021/ol300742t.

(13) Schultz, E. E.; Lindsay, V. N. G.; Sarpong, R. Expedient Synthesis of Fused Azepine Derivatives Using a Sequential Rhodium(II)-Catalyzed Cyclopropanation/1-Aza-Cope Rearrangement of Dienyltriazoles. *Angew. Chem., Int. Ed.* **2014**, *53*, 9904–9908. DOI: 10.1002/anie.201405356.

(14) Budwitz, J. E.; Newton, C. G. Synthesis of a 2,5-Bis(*tert*-Butyldimethylsilyloxy)furan and its Reaction with Benzyne. *Org. Synth.* **2023**, *100*, 159–185. DOI: 10.15227/orgsyn.100.0159.

(15) (a) Bruker AXS Inc. APEX3 Crystallography Software Suite, 2016, 5465 East Cheryl Parkway, Madison, WI 53711,USA: a. (b) Sheldrick, G. M. A Short History of SHELX. *Acta Cryst. A.* **2008**, *64*, 112–122. DOI: 10.1107/s0108767307043930. (c) Sheldrick, G. M. Crystal

Structure Refinement with SHELXL. *Acta Cryst. C.* **2015**, *71*, 3–8. DOI: 10.1107/s2053229614024218.
(16) Dissanayake, I.; Hart, J. D.; Becroft, E. C.; Sumby, C. J.; Newton, C. G. Bisketene Equivalents as Diels–Alder Dienes. *J. Am. Chem. Soc.* **2020**, *142*, 13328–13333. DOI: 10.1021/jacs.0c06306.
(17) Marcoux, D.; Bindschädler, P.; Speed, A. W. H.; Chiu, A.; Pero, J. E.; Borg, G. A.; Evans, D. A. Effect of Counterion Structure on Rates and Diastereoselectivities in α,β-Unsaturated Iminium-Ion Diels–Alder Reactions. *Org. Lett.* **2011**, *13*, 3758–3761. DOI: 10.1021/ol201448h.
(18) Kőnig, B.; Sztanó, G.; Holczbauer, T.; Soós, T. Syntheses of 2- and 3-Substituted Morpholine Congeners via Ring Opening of 2-Tosyl-1,2-Oxazetidine. *J. Org. Chem.* **2023**, *88*, 6182–6191. DOI: 10.1021/acs.joc.3c00207.
(19) Huffman, B. J.; Chen, S.; Schwarz, J. L.; Plata, R. E.; Chin, E. N.; Lairson, L. L.; Houk, K. N.; Shenvi, R. A. Electronic Complementarity Permits Hindered Butenolide Heterodimerization and Discovery of Novel cGAS/STING Pathway Antagonists. *Nat. Chem.* **2020**, *12*, 310–317. DOI: 10.1038/s41557-019-0413-8.
(20) Ishida, A.; Yamashita, S.; Takamuku, S. Hydroxyalkylation and Lactone Formation from Dialkyl Malonate by Means of a Eu^{III}/Eu^{II} Photoredox System. *Bull. Chem. Soc. Jpn.* **1988**, *61*, 2229–2231. DOI: 10.1246/bcsj.61.2229.
(21) Getzler, Y. D. Y. L.; Kundnani, V.; Lobkovsky, E. B.; Coates, G. W. Catalytic Carbonylation of β-Lactones to Succinic Anhydrides. *J. Am. Chem. Soc.* **2004**, *126*, 6842–6843. DOI: 10.1021/ja048946m.
(22) Kozuma, A.; Komatsuki, K.; Saito, K.; Yamada, T. Thermal Decarboxylative Nazarov Cyclization of Cyclic Enol Carbonates Involving Chirality Transfer. *Chem. Lett.* **2020**, *49*, 60–63. DOI: 10.1246/cl.190763.
(23) Kofron, W. G.; Baclawski, L. M. A Convenient Method for Estimation of Alkyllithium Concentrations. *J. Org. Chem.* **1976**, *41*, 1879–1880. DOI: 10.1021/jo00872a047.
(24) Cadierno, V.; García-Garrido, S. E.; Gimeno, J. Isomerization of Propargylic Alcohols into α,β-Unsaturated Carbonyl Compounds Catalyzed by the Sixteen-Electron Allyl-Ruthenium(II) Complex $[Ru(\eta^3\text{-}2\text{-}C_3H_4Me)(CO)(dppf)][SbF_6]$. *Adv. Synth. Catal.* **2006**, *348*, 101–110. DOI: 10.1002/adsc.200505294.
(25) Ayres, D. C.; Raphael, R. A. *trans-cyclo*Heptane-1:2-Dicarboxylic Acid and its Conversion into *trans*-Bicyclo[5:3:0]decan-9-One. *J. Chem. Soc.* **1958**, 1779–1789. DOI: 10.1039/jr9580001779.
(26) Lead experimentalist on the subsequent reference.
(27) Saito, F.; Gerbig, D.; Becker, J.; Schreiner, P. R. Absolute Configuration of *trans*-Perhydroazulene. *Org. Lett.* **2020**, *22*, 3895–3899. DOI: 10.1021/acs.orglett.0c01184.
(28) Ghilardi, A. F.; Yaaghubi, E.; Ferreira, R. B.; Law, M. E.; Yang, Y.; Davis, B. J.; Schilson, C. M.; Ghiviriga, I.; Roitberg, A. E.; Law, B. K.; Castellano, R. K. Anticancer Agents Derived from Cyclic Thiosulfonates: Structure-Reactivity and Structure-Activity Relationships. *ChemMedChem* **2022**, *17*, e202200165. DOI: 10.1002/cmdc.202200165.
(29) Sawamura, Y.; Nakatsuji, H.; Sakakura, A.; Ishihara, K. "Phosphite–Urea" Cooperative High-Turnover Catalysts for the Highly Selective Bromocyclization of Homogeranylarenes. *Chem. Sci.* **2013**, *4*, 4181–4186. DOI: 10.1039/c3sc51432c.
(30) Cromm, P. M.; Samarasinghe, K. T. G.; Hines, J.; Crews, C. M. Addressing Kinase-Independent Functions of Fak via PROTAC-Mediated Degradation. *J. Am. Chem. Soc.* **2018**, *140*, 17019–17026. DOI: 10.1021/jacs.8b08008.

(31) Firth, J. D.; Hammarback, L. A.; Burden, T. J.; Eastwood, J. B.; Donald, J. R.; Horbaczewskyj, C. S.; McRobie, M. T.; Tramaseur, A.; Clark, I. P.; Towrie, M.; Robinson, A.; Krieger, J. P.; Lynam, J. M.; Fairlamb, I. J. S. Light- and Manganese-Initiated Borylation of Aryl Diazonium Salts: Mechanistic Insight on the Ultrafast Time-Scale Revealed by Time-Resolved Spectroscopic Analysis. *Chem. Eur. J.* **2021**, *27*, 3979–3985. DOI: 10.1002/chem.202004568.
(32) Shen, X.-Q.; Wang, S.-Q.; Fan, D.; Zhang, X.-G.; Tu, H.-Y. Copper-Catalyzed Selective Defluorinative Sulfuration of Trifluoropropanamides Leading to α-Fluorothioacrylamides. *J. Org. Chem.* **2021**, *86*, 1591–1600. DOI: 10.1021/acs.joc.0c02436.
(33) Nguyen, T. B.; Sorres, J.; Tran, M. Q.; Ermolenko, L.; Al-Mourabit, A. Boric Acid: A Highly Efficient Catalyst for Transamidation of Carboxamides with Amines. *Org. Lett.* **2012**, *14*, 3202–3205. DOI: 10.1021/ol301308c.
(34) Lal, S.; Snape, T. J. Towards a Sustainable Synthesis of Aniline-Derived Amides Using an Indirect Chemoenzymatic Process: Challenges and Successes. *RSC Adv.* **2014**, *4*, 1609–1615. DOI: 10.1039/c3ra46499g.
(35) De, S.; Yin, J.; Ma, D. Copper-Catalyzed Coupling Reaction of (Hetero)Aryl Chlorides and Amides. *Org. Lett.* **2017**, *19*, 4864–4867. DOI: 10.1021/acs.orglett.7b02326.
(36) Joseph, B.; Darro, F.; Béhard, A.; Lesur, B.; Collignon, F.; Decaestecker, C.; Frydman, A.; Guillaumet, G.; Kiss, R. 3-Aryl-2-Quinolone Derivatives: Synthesis and Characterization of In Vitro and In Vivo Antitumor Effects with Emphasis on a New Therapeutical Target Connected with Cell Migration. *J. Med. Chem.* **2002**, *45*, 2543–2555. DOI: 10.1021/jm010978m.
(37) Lundberg, H.; Tinnis, F.; Adolfsson, H. Direct Amide Coupling of Non-Activated Carboxylic Acids and Amines Catalysed by Zirconium(IV) Chloride. *Chem. Eur. J.* **2012**, *18*, 3822–3826. DOI: 10.1002/chem.201104055.
(38) Chen, Z.-W.; Jiang, H.-F.; Pan, X.-Y.; He, Z.-J. Practical Synthesis of Amides from Alkynyl Bromides, Amines, and Water. *Tetrahedron* **2011**, *67*, 5920–5927. DOI: 10.1016/j.tet.2011.06.045.
(39) Pampana, V. K. K.; Sagadevan, A.; Ragupathi, A.; Hwang, K. C. Visible Light-Promoted Copper Catalyzed Regioselective Acetamidation of Terminal Alkynes by Arylamines. *Green Chem.* **2020**, *22*, 1164–1170. DOI: 10.1039/c9gc03608c.
(40) Cheung, C. W.; Shen, N.; Wang, S.-P.; Ullah, A.; Hu, X.; Ma, J.-A. Manganese-Mediated Reductive Amidation of Esters with Nitroarenes. *Org. Chem. Front.* **2019**, *6*, 756–761. DOI: 10.1039/c8qo01405a.
(41) Ji, X.; Zhang, Z.; Wang, Y.; Han, Y.; Peng, H.; Li, F.; Liu, L. Catalyst-Free Synthesis of α,α-Disubstituted Carboxylic Acid Derivatives Under Ambient Conditions via a Wolff Rearrangement Reaction. *Org. Chem. Front.* **2021**, *8*, 6916–6922. DOI: 10.1039/d1qo01265g.
(42) Meng, F.; Cheng, S.; Ding, H.; Liu, S.; Liu, Y.; Zhu, K.; Chen, S.; Lu, J.; Xie, Y.; Li, L.; Liu, R.; Shi, Z.; Zhou, Y.; Liu, Y.-C.; Zheng, M.; Jiang, H.; Lu, W.; Liu, H.; Luo, C. Discovery and Optimization of Novel, Selective Histone Methyltransferase SET7 Inhibitors by Pharmacophore- and Docking-Based Virtual Screening. *J. Med. Chem.* **2015**, *58*, 8166–8181. DOI: 10.1021/acs.jmedchem.5b01154.
(43) Ke, Z.; Lam, Y.-P.; Chan, K.-S.; Yeung, Y.-Y. Zwitterion-Catalyzed Deacylative Dihalogenation of β-Oxo Amides. *Org. Lett.* **2020**, *22*, 7353–7357. DOI: 10.1021/acs.orglett.0c02701.
(44) Wippich, J.; Truchan, N.; Bach, T. Rhodium-Catalyzed *N-tert*-Butoxycarbonyl (Boc) Amination by Directed C–H Bond Activation. *Adv. Synth. Catal.* **2016**, *358*, 2083–2087. DOI: 10.1002/adsc.201600410.

(45) da Silva, C. D. G.; Katla, R.; dos Santos, B. F.; Tavares Junior, J. M. C.; Albuquerque, T. B.; Kupfer, V. L.; Rinaldi, A. W.; Domingues, N. L. C. Cobalt Used as a Novel and Reusable Catalyst: A New and One-Pot Synthesis of Isatin-Derived *N,S*-Acetals Using Substituted Isatins and Thiols. *Synthesis* **2019**, *51*, 4014–4022. DOI: 10.1055/s-0037-1611913.
(46) McCarthy, D. G.; Hegarty, A. F. Rapid Acid-Catalysed and Uncatalysed Hydration of Ketenimines. *J. Chem. Soc., Perkin Trans. 2* **1980**, 579–591. DOI: 10.1039/p29800000579.
(47) Sung, K.; Wu, S.-H.; Wu, R.-R.; Sun, S.-Y. NMR and ab Initio Studies of Amination of Ketenimine: Direct Evidence for a Mechanism Involving a Vinylidenediamine as an Intermediate. *J. Org. Chem.* **2002**, *67*, 4298–4303. DOI: 10.1021/jo025523z.
(48) Sung, K.; Huang, P.-M.; Chiang, S.-M. Kinetic Studies for Amination of Ketenimines: Change of Rate-Determining Step by Electron-Withdrawing *N*-Substituents through Electronic Effects. *Tetrahedron* **2006**, *62*, 4795–4799. DOI: 10.1016/j.tet.2006.03.022.
(49) Sung, K.; Chen, F.-L.; Huang, P.-M.; Chiang, S.-M. Mechanistic Studies of Amination of Ketenimines: Change of Rate-Determining Step by *N*-Substituents Through Electronic Effects. *Tetrahedron* **2006**, *62*, 171–181. DOI: 10.1016/j.tet.2005.09.119.
(50) Katagiri, T.; Handa, M.; Asano, H.; Asanuma, T.; Mori, T.; Jukurogi, T.; Uneyama, K. Preparations and Reactions of 2-Trifluoromethylketenimines. *J. Fluor. Chem.* **2009**, *130*, 714–717. DOI: 10.1016/j.jfluchem.2009.05.020.
(51) Moderhack, D.; Stolz, K. Cycloaddition von *N*-Aryl-*tert*-Butylketeniminen an Nitrosobenzol sowie 2-Methyl-2-Nitrosopropan. *Chem. Ber.* **1986**, *119*, 3411–3421. DOI: 10.1002/cber.19861191118.
(52) Zhou, X.; Jiang, Z.; Xue, L.; Lu, P.; Wang, Y. Preparation of 1,2,5-Trisubstituted 1*H*-Imidazoles from Ketenimines and Propargylic Amines by Silver-Catalyzed or Iodine-Promoted Electrophilic Cyclization Reaction of Alkynes. *Eur. J. Org. Chem.* **2015**, 5789–5797. DOI: 10.1002/ejoc.201500704.
(53) Shimizu, M.; Gama, Y.; Takagi, T.; Shibakami, M.; Shibuya, I. A Convenient Synthesis of Ketenimines from Thioamides with Haloiminium Salts. *Synthesis* **2000**, *2000*, 517–520. DOI: 10.1055/s-2000-6362.
(54) Taylor, E. C.; McKillop, A.; Hawks, G. H. Diphenylketene. *Org. Synth.* **1972**, *52*, 36. DOI: 10.15227/orgsyn.052.0036.
(55) Goll, J. M.; Fillion, E. Tuning the Reactivity of Palladium Carbenes Derived from Diphenylketene. *Organometallics* **2008**, *27*, 3622–3625. DOI: 10.1021/om800390w.
(56) Meier, S.; Würthwein, E. U. Acetylierung von *N*-(Trimethylsilyl)keteniminen: *N*-Acylketenimine und α-Cyanketone. *Chem. Ber.* **1990**, *123*, 2339–2347. DOI: 10.1002/cber.19901231218.
(57) Horvath, K. L.; Magann, N. L.; Sowden, M. J.; Gardiner, M. G.; Sherburn, M. S. Unlocking Acyclic π-Bond Rich Structure Space with Tetraethynylethylene–Tetravinylethylene Hybrids. *J. Am. Chem. Soc.* **2019**, *141*, 19746–19753. DOI: 10.1021/jacs.9b08885.
(58) Lin, H.-S.; Paquette, L. A. A Convenient Method for Determining the Concentration of Grignard Reagents. *Synth. Commun.* **1994**, *24*, 2503–2506. DOI: 10.1080/00397919408010560.
(59) Shibuya, M.; Ito, S.; Takahashi, M.; Iwabuchi, Y. Oxidative Rearrangement of Cyclic Tertiary Allylic Alcohols with IBX in DMSO. *Org. Lett.* **2004**, *6*, 4303–4306. DOI: 10.1021/ol048210u.
(60) Di Mola, A.; Tedesco, C.; Massa, A. Metal-Free Air Oxidation in a Convenient Cascade Approach for the Access to Isoquinoline-1,3,4(2*H*)-triones. *Molecules* **2019**, *24*, 2177. DOI: 10.3390/molecules24112177.

(61) Nakazaki, A.; Mori, A.; Kobayashi, S.; Nishikawa, T. Diastereoselective Synthesis of 3,3-Disubstituted Oxindoles from Atropisomeric *N*-Aryl Oxindole Derivatives. *Tetrahedron Lett.* **2012**, *53*, 7131–7134. DOI: 10.1016/j.tetlet.2012.10.092.
(62) Kendall, P. M.; Johnson, J. V.; Cook, C. E. Synthetic Route to an Aromatic Analog of Strigol. *J. Org. Chem.* **1979**, *44*, 1421–1424. DOI: 10.1021/jo01323a013.
(63) Zhao, X.; Liu, D.; Xie, F.; Zhang, W. Enamines: Efficient Nucleophiles for the Palladium-Catalyzed Asymmetric Allylic Alkylation. *Tetrahedron* **2009**, *65*, 512–517. DOI: 10.1016/j.tet.2008.11.003.
(64) Li, J.; Kong, M.; Qiao, B.; Lee, R.; Zhao, X.; Jiang, Z. Formal Enantioconvergent Substitution of Alkyl Halides via Catalytic Asymmetric Photoredox Radical Coupling. *Nat. Commun.* **2018**, *9*, 2445. DOI: 10.1038/s41467-018-04885-3.
(65) Retich, C.; Bräse, S. Asymmetric Organocatalytic Synthesis of Bisindoles – Scope and Derivatizations. *Eur. J. Org. Chem.* **2018**, *2018*, 60–77. DOI: 10.1002/ejoc.201701502.
(66) Morita, Y.; Yamamoto, T.; Nagai, H.; Shimizu, Y.; Kanai, M. Chemoselective Boron-Catalyzed Nucleophilic Activation of Carboxylic Acids for Mannich-Type Reactions. *J. Am. Chem. Soc.* **2015**, *137*, 7075–7078. DOI: 10.1021/jacs.5b04175.
(67) Reed-Berendt, B. G.; Morrill, L. C. Manganese-Catalyzed *N*-Alkylation of Sulfonamides Using Alcohols. *J. Org. Chem.* **2019**, *84*, 3715–3724. DOI: 10.1021/acs.joc.9b00203.
(68) *Gaussian 16 Rev. A.03*; Wallingford, CT, 2016.
(69) Grimme, S. Supramolecular Binding Thermodynamics by Dispersion-Corrected Density Functional Theory. *Chem. Eur. J.* **2012**, *18*, 9955–9964. DOI: 10.1002/chem.201200497.
(70) Ingman, V. M.; Schaefer, A. J.; Andreola, L. R.; Wheeler, S. E. QChASM: Quantum Chemistry Automation and Structure Manipulation. *WIREs Comput. Mol. Sci.* **2021**, *11*, e1510. DOI: 10.1002/wcms.1510.
(71) Liang, J.; Feng, X.; Hait, D.; Head-Gordon, M. Revisiting the Performance of Time-Dependent Density Functional Theory for Electronic Excitations: Assessment of 43 Popular and Recently Developed Functionals from Rungs One to Four. *J. Chem. Theory Comput.* **2022**, *18*, 3460–3473. DOI: 10.1021/acs.jctc.2c00160.
(72) (a) Chai, J.-D.; Head-Gordon, M. Long-Range Corrected Hybrid Density Functionals with Damped Atom–Atom Dispersion Corrections. *Phys. Chem. Chem. Phys.* **2008**, *10*, 6615–6620. DOI: 10.1039/b810189b. (b) Ditchfield, R.; Hehre, W. J.; Pople, J. A. Self-Consistent Molecular-Orbital Methods. IX. An Extended Gaussian-Type Basis for Molecular-Orbital Studies of Organic Molecules. *J. Chem. Phys.* **1971**, *54*, 724–728. DOI: 10.1063/1.1674902. (c) Hehre, W. J.; Ditchfield, R.; Pople, J. A. Self—Consistent Molecular Orbital Methods. XII. Further Extensions of Gaussian—Type Basis Sets for Use in Molecular Orbital Studies of Organic Molecules. *J. Chem. Phys.* **1972**, *56*, 2257–2261. DOI: 10.1063/1.1677527.
(73) (a) Hariharan, P. C.; Pople, J. A. The Influence of Polarization Functions on Molecular Orbital Hydrogenation Energies. *Theor. Chim. Acta* **1973**, *28*, 213–222. DOI: 10.1007/bf00533485. (b) Marenich, A. V.; Cramer, C. J.; Truhlar, D. G. Universal Solvation Model Based on Solute Electron Density and on a Continuum Model of the Solvent Defined by the Bulk Dielectric Constant and Atomic Surface Tensions. *J. Phys. Chem. B.* **2009**, *113*, 6378–6396. DOI: 10.1021/jp810292n. (c) Tomasi, J.; Mennucci, B.; Cammi, R. Quantum Mechanical Continuum Solvation Models. *Chem. Rev.* **2005**, *105*, 2999–3094. DOI: 10.1021/cr9904009.
(74) (a) Grimme, S. Exploration of Chemical Compound, Conformer, and Reaction Space with Meta-Dynamics Simulations Based on Tight-Binding Quantum Chemical Calculations. *J. Chem. Theory Comput.* **2019**, *15*, 2847–2862. DOI: 10.1021/acs.jctc.9b00143. (b) Pracht, P.; Bohle, F.;

Grimme, S. Automated Exploration of the Low-Energy Chemical Space with Fast Quantum Chemical Methods. *Phys. Chem. Chem. Phys.* **2020**, *22*, 7169–7192. DOI: 10.1039/c9cp06869d.
(75) (a) Fukui, K. Formulation of the Reaction Coordinate. *J. Phys. Chem.* **1970**, *74*, 4161–4163. DOI: 10.1021/j100717a029. (b) Fukui, K. The Path of Chemical Reactions - The IRC Approach. *Acc. Chem. Res.* **1981**, *14*, 363–368. DOI: 10.1021/ar00072a001.
(76) Luchini, G.; Alegre-Requena, J. V.; Funes-Ardoiz, I.; Paton, R. S. GoodVibes: Automated Thermochemistry for Heterogeneous Computational Chemistry Data. *F1000Research* **2020**, *9*. DOI: 10.12688/f1000research.22758.1.
(77) Li, Y.-P.; Gomes, J.; Mallikarjun Sharada, S.; Bell, A. T.; Head-Gordon, M. Improved Force-Field Parameters for QM/MM Simulations of the Energies of Adsorption for Molecules in Zeolites and a Free Rotor Correction to the Rigid Rotor Harmonic Oscillator Model for Adsorption Enthalpies. *J. Phys. Chem. C.* **2015**, *119*, 1840–1850. DOI: 10.1021/jp509921r.
(78) Schaefer, A. J.; Ingman, V. M.; Wheeler, S. E. SEQCROW: AChimeraXbundle to Facilitate Quantum Chemical Applications to Complex Molecular Systems. *J. Comput. Chem.* **2021**, *42*, 1750–1754. DOI: 10.1002/jcc.26700.
(79) Meng, E. C.; Goddard, T. D.; Pettersen, E. F.; Couch, G. S.; Pearson, Z. J.; Morris, J. H.; Ferrin, T. E. UCSF ChimeraX: Tools for Structure Building and Analysis. *Protein Sci.* **2023**, *32*, e4792. DOI: 10.1002/pro.4792.
